# Effectiveness of Journal Ranking Schemes as a Tool for Locating Information

**DOI:** 10.1371/journal.pone.0001683

**Published:** 2008-02-27

**Authors:** Michael J. Stringer, Marta Sales-Pardo, Luís A. Nunes Amaral

**Affiliations:** 1 Department of Physics and Astronomy, Northwestern University, Evanston, Illinois, United States of America; 2 Department of Chemical and Biological Engineering, Northwestern University, Evanston, Illinois, United States of America; 3 Northwestern Institute on Complex Systems (NICO), Northwestern University, Evanston, Illinois, United States of America; University of East Piedmont, Italy

## Abstract

**Background:**

The rise of electronic publishing [1], preprint archives, blogs, and wikis is raising concerns among publishers, editors, and scientists about the present day relevance of academic journals and traditional peer review [2]. These concerns are especially fuelled by the ability of search engines to automatically identify and sort information [1]. It appears that academic journals can only remain relevant if acceptance of research for publication within a journal allows readers to infer *immediate*, reliable information on the value of that research.

**Methodology/Principal Findings:**

Here, we systematically evaluate the effectiveness of journals, through the work of editors and reviewers, at evaluating unpublished research. We find that the distribution of the number of citations to a paper published in a given journal in a specific year converges to a steady state after a journal-specific transient time, and demonstrate that in the steady state the logarithm of the number of citations has a journal-specific typical value. We then develop a model for the asymptotic number of citations accrued by papers published in a journal that closely matches the data.

**Conclusions/Significance:**

Our model enables us to quantify both the typical impact and the range of impacts of papers published in a journal. Finally, we propose a journal-ranking scheme that maximizes the efficiency of locating high impact research.

## Introduction

As de Solla Price observed [3], the number of scientific journals and the number of papers published in those journals is increasing at an approximately exponential rate. The size and growth of the research literature places a tremendous burden on researchers—how are they to select what to browse, what to read, and what to cite from a large and quickly growing body of literature?

This burden does not only affect researchers. Funding agencies, university administrators, and reviewers are called on to evaluate the productivity of researchers and institutions, as well as the impact of their work. Typically, these agents have neither the time nor the financial resources to obtain an in-depth evaluation of the actual research and must instead use indirect indicators of quality such as number of publications, *h*-index, number of citations, or journal rank [4]–[8].

Despite the oversimplification of using just a few numbers to quantify the scientific merit of a body of research, the entire science and technology community is relying more and more on citation-based statistics as a tool for evaluating the research quality of individuals and institutions [9]. An example of this trend is the widespread use of the Institute of Scientific Information (ISI) Journal Impact Factor (JIF) to rate scientific journals. This practice is pervasive enough that, despite evidence that the JIF can be misleading [10], [11], some countries pay researchers per paper published with the amount being determined by the JIF of the journal in which the paper is published [12].

This act of “judging a book by its cover” has caused researchers to note that we should judge a paper not by the number of citations that the journal in which it is published receives, but by the number of citations the paper itself receives [13]. This seemingly obvious fact is countered by one major challenge—administrators often want an estimate of the impact of a paper long before it has finished accumulating citations, which, as we show later, might take as long as 26 years.

The need for an estimate of the ultimate impact of recently published articles is the reason that the JIF is often used as a proxy for quality of the research. Indeed, the premise of the peer-reviewing process is that reviewers are in fact able to assess the quality of a paper. Thus, the heuristic that the journal in which a paper is published is a good proxy for the ultimate impact of a paper is likely to be an adaptive one [14].

Like any heuristic, the evaluation of research using citation analysis has weaknesses. These weaknesses have been extensively explored in the literature [15], [16], however, as reviewed by Nicolaisen [17], there are plausible assumptions underlying the use of citation analysis as a heuristic. Here, we assume that the quality of a paper bears *significant correlation* with the ultimate impact of the paper, that is, the asymptotic total number of citations to that paper. We further assume that the actual relation between total number of citations and quality is uncertain, and may be field- and even journal-dependent. This latter assumption is prompted by the observation that many extrinsic factors for which we have no data can influence the number of citations that the paper receives. For example, because social influence may affect the citations to a paper, small differences in quality may lead to large differences in the number of citations [18].

In this article, we investigate two fundamental aspects concerning the prediction of the ultimate impact of a published research paper: (i) the time scale *τ* for the full impact of papers published in a given journal to become apparent, and (ii) the typical impact of papers published in a given journal. We find that *τ* varies from less than 1 year to 26 years, depending on the journal. Additionally, we find that there is a typical value and a well-defined range for the eventual impact of papers published in a given journal, which enables us to develop a model for the distribution of paper impacts that matches the data. These findings lead us to propose a method of ranking journals based on a natural criterion: the higher a journal is ranked, the higher the probability of finding a high impact paper published in that journal.

## Results

We obtained the number of citations accrued by December 31, 2006 for 22,951,535 papers tracked in Thomson Scientific's Web of Science® (WoS) database. This database comprises information on papers published in ∼5,800 science and engineering journals, ∼1,700 social science journals, and ∼1,100 arts and humanities journals. Journals are typically covered from their inception or from the beginning of the WoS coverage for the research area (whichever is later) until the present date or until their demise (whichever is earlier). The beginning of WoS coverage for science and engineering, social science, and arts and humanities is 1955, 1956, and 1975 respectively. In this study, we restrict our analysis to journals publishing at least 50 articles per year for at least 15 years. This condition restricts our analysis to 19,372,228 articles published in 2,267 journals, and enables us to ensure good statistics on the journals that we include in the analysis. More information about the data is included in Appendix S1.

Because the citation history of a paper may be field- and even journal-dependent, we first investigate 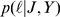, the probability distribution of *ℓ*, the logarithm of the number of citations accrued by each paper by December 31st of 2006, for articles published in journal *J* during year *Y*. We define *ℓ* as
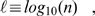
(1)where *n* is the number of accrued citations.

**Figure 1:**
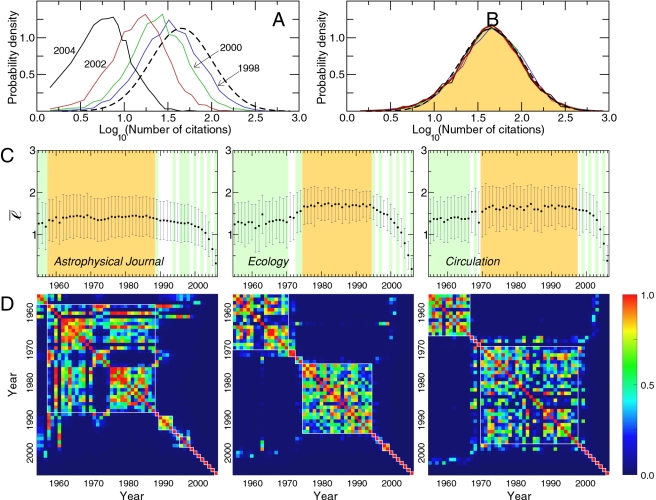
Time evolution of the distribution of number of citations of the papers published in a given academic journal. (A) Probability density function 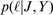, where *Y* is a year in the period 1998–2004, *J* is the *Journal of Biological Chemistry*, and *ℓ*≡log_10_(*n*) where *n* is the number of citations accrued by a paper between its publication date and December 31, 2006. Because the papers published in those years are still accruing citations by December 2006, the distributions are not stationary, but instead “drift” to higher values of *ℓ*. (B) 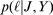 for the *Journal of Biological Chemistry* and for *Y* in the period 1991–1993. For this period, the distributions are essentially identical, indicating that 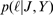 has converged to its steady-state form 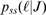. The steady-state distribution is well described by a normal with mean 1.65 and standard deviation 0.35 (black dashed curve). (C) Time dependence of 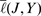 for three journals: *Astrophysical Journal*, *Ecology*, and *Circulation*. As for the *Journal of Biological Chemistry*, we find that after some transient period, 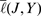 reaches a stationary value 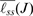 (see Methods). The orange region highlights the set of years for which we consider that 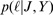 is stationary. The time scale *τ*(*J*) for reaching the steady-state strongly depends on the journal: *τ*(*Astrophysical Journal*) = 18 years, *τ*(*Ecology*) = 12 years, and *τ*(*Circulation*) = 9 years. Significantly, we find no correlations between *τ*(*J*) and 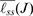, whose values are 1.44 for *Astrophysical Journal*, 1.70 for *Ecology*, and 1.66 for *Circulation*. (D) Pairwise comparison of citation distributions for different years for a given journal. We show the matrices of *p*-values obtained using the Kolmogorov-Smirnov test [29] for the *Astrophysical Journal*, *Ecology*, and *Circulation*. We color the matrix elements following the color code on the right. *p*-values close to one mean that it is likely that both distributions come from a common underlying distribution; *p*-values close to zero mean that is it very unlikely that both distributions come from a common underlying distribution. We then use a box-diagonal model [28] to identify contiguous blocks of years for which the *p*-value is large enough that the null hypothesis cannot be rejected. The white lines in the matrices indicate the best fit of a box-diagonal model. We identify the first box with more than 2 years for which 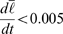 to be the steady-state period (see Methods).

Figures **1A,B** display estimates of the probability density function 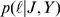 for the *Journal of Biological Chemistry* for different years. Two patterns are apparent from the data. First, the distribution for each of the years considered shows a tendency to peak around a central value, that is, there is a characteristic value for *ℓ*. Second, after about 10 years, the distribution has converged to a steady-state functional form, 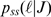. The explanation for this apparently counter-intuitive observation is that papers with a small number of citations have stopped accruing citations, while the trickle of citations to the most highly-cited papers is small when compared to the already accrued citations, and thus does not significantly change the value of the *logarithm* of the number of citations.

These results are not restricted to the *Journal of Biological Chemistry*; 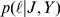 displays these two characteristics for nearly all journals we analyzed (see Appendix S2). However, as illustrated in Figure **1C**, the mean value of *ℓ* in the steady state,
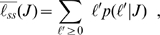
(2)and the time *τ*(*J*) needed to reach the steady state depend on the journal—for example, *τ*(*Astrophysical Journal*) is more than twice *τ*(*Circulation*), yet 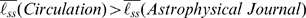.

The existence of a steady state for 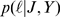 prompts us to investigate: (i) the functional form of 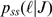, and (ii) whether there is a universal functional form for all journals. As others have noted [19], many papers remain uncited even decades after their publication. For those papers that do get cited, the total number of citations varies over *five* orders of magnitude (the most highly-cited paper in the data [20] had received 196,452 citations by the end of 2006). Nevertheless, *ℓ* follows a distribution that is approximately normal (Figures **1A,B**).

In order to explain our empirical findings, we develop a model for the asymptotic number of citations a paper published in journal *J* will receive. Our first assumption is that the papers published in journal *J* have a normal distribution of “quality”, *q*∈*N*(*μ*,*σ*), where *μ* and *σ* depend on *J*. The simplest model is to equate the ultimate impact with quality, *ℓ*≈*q*, so that *n*≈10*^q^*. However, since 10*^q^* is a continuous random variable, whereas *n* is integer-valued, the model needs further refinement. In particular, the model must also specify how the continuous values of *q* map onto the discrete values of *n*. For generality, we introduce an additional parameter *γ* to the model, such that
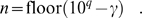
(3)


**Figure 2:**
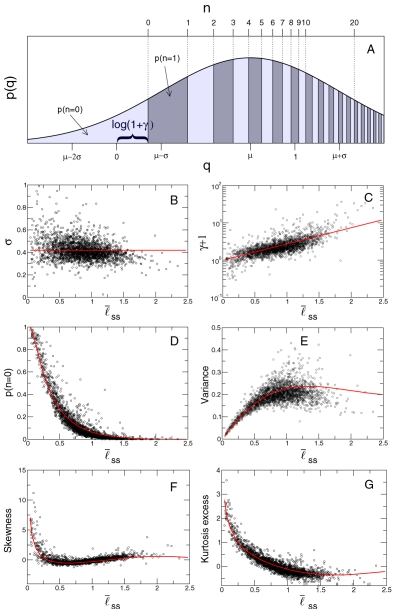
Modeling the steady-state distributions of the number of citations for papers published in a given journal. (A) Our model assumes that the “quality” of the papers published by a journal obeys a normal distribution with mean *μ* and standard deviation *σ*. The number of citations of a paper with quality *q*∈*N*(*μ*,*σ*) is given by Eq. (3). Because the quality is a continuous variable whereas the number of citations is an integer quantity, the same number of citations will occur for papers with qualities spanning a certain range of *q*. In particular, all papers for which *q*<log_10_(1+*γ*) will receive no citations. In the panel, the areas of differently shaded regions yield the probability of a paper accruing a given number of citations. (B) Scatter plot of the estimated value of *σ* versus 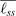 for all 2,267 journals considered in our analysis (see Methods and Appendices S1 and S4 for details on the fits). Notice that *σ* is almost independent of 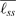. The solid line corresponds to *σ* = 0.419, the mean of the estimated values of *σ* for all journals (see Methods). (C) Scatter plot of the estimated value of *γ*+1 for versus 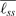. Notice the strong correlation between the two variables. The solid line corresponds to 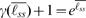 (see Methods for details on the fit). (D) Fraction of uncited papers as a function of 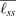. For this and all subsequent panels, solid lines show the predictions of the model using 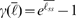, *σ* = 0.419, and a value of *μ* for each 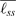 (see Methods). (E) Variance of *ℓ* as a function of 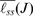. (F) Skewness of *ℓ* as a function of 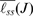. The skewness of the normal distribution is zero. (G) Kurtosis excess of *ℓ* as a function of 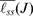. The kurtosis excess of the normal distribution is zero. Note how, for the case of 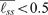, the moments of the distribution of citations for cited papers deviate significantly from those expected for a normal distribution. In contrast, for 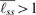, only a small fraction of papers remains uncited, so deviations from the expectations for a normal distribution are small.

One can interpret *γ* as the value of *q* at which one can expect a paper to get cited once (Figure **2A**). More generally, one could write *n* = floor(10*^q+ε^*−*γ*), where *ε*∈*N*(0,*σ_ε_*), to account for external influences to the number of citations. For example, assuming *γ* = 0 and *q* = 3, one would get *n* = 794 for *ε* = −0.1 and *n* = 1258 for *ε* = 0.1. However, if *ε* is independent of *J*, 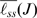 will not be significantly affected by *ε*. Thus, even though the number of citations to individual papers may change, the mean for a journal will not. To demonstrate the agreement between our model and the data, in Figure 2 we plot the moments of the empirical distributions for each journal together with the predictions of our model for those quantities. It is visually apparent that the model provides a close description of the data.

## Discussion

Our finding that the distribution of number of citations is log-normal is in agreement with recent generative models of the citation network [21], [22] that predict a log-normal distribution for subsets of papers related by content similarity. Note that this result is not in disagreement with prior claims about the power-law behavior of the citation distribution [23], as the convolution of many log-normal distributions with different means can yield a distribution that can be hard to distinguish from a power law.

The findings reported in Figures 1 and 2 demonstrate that there is a quantity, related to the ultimate impact of a paper, which for papers published in a given journal is normally distributed. For all papers published in journal *J*, that quantity has a well-defined mean, *q̅*(*J*) = *μ*, implying that the average *q* of the papers is *representative* of the *q* of all the papers published in the journal and, thus, of the *q* of the journal.

Our findings thus suggest the possibility of ranking journals according to *q̅*(*J*). To this end, we turn to a heuristic used in information retrieval called the Probability Ranking Principle [24]. This principle dictates that the optimal ranking of a set of journals will be the one that maximizes the probability that given a pair of papers (*a,b*) from journals A and B, respectively, *q*(*a*)>*q*(*b*) if A is above B in that ranking. This probability is also known as the multi-class “area under curve” (AUC) statistic [25]–[27] (see Methods and Appendix S1 for details).

**Figure 3:**
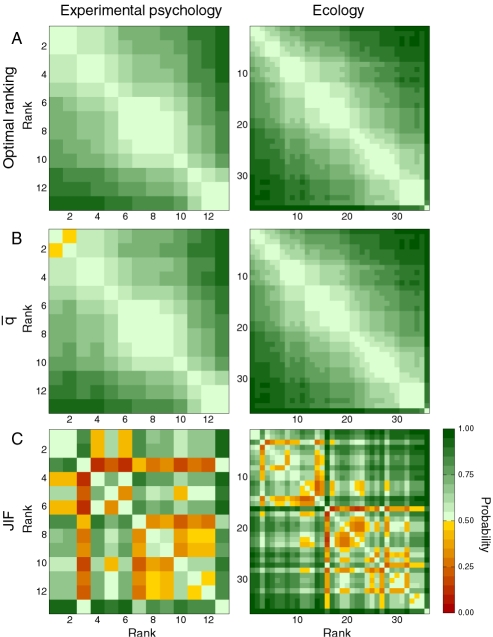
Comparison of citation-based journal ranking schemes. We present results for 13 journals that the ISI classifies primarily in experimental psychology, and 36 journals that the ISI classifies primarily in ecology (see Appendix S3 for other fields). For every pair of journals, *J_i_* and *J_j_*, belonging to the same field, we obtain the probability *p_ij_* that a randomly selected paper published in *J_i_* has received more citations than a randomly selected paper published in *J_j_*. We rank the journals in each field according to three schemes: (A) optimal ranking R^AUC^, that is, the ranking that maximizes *p_ij_* for R(*i*)<R(*j*); (B) ranking according to decreasing *q̅*(*J*); (C) ranking according to decreasing JIF. We plot {*p_ij_*} matrices for each of the fields and ranking schemes using the color scheme on the right. Green indicates adequate ranking, whereas red indicates inadequate ranking. It is visually apparent that the ranking according to decreasing *q̅*(*J*) provides nearly optimal ranking, whereas ranking according to decreasing JIF does not. As an example, consider the journals *Brain and Cognition* and *Journal of Experimental Psychology: Learning, Memory, and Cognition*. The JIF ranks *Brain Cogn.* third and *J. Exp. Psy.* fourth. However, the median number of cumulative citations to the papers published in the latter is 34, and only 3 for papers published in the former. Not surprisingly, the probability of a randomly selected paper published in *J. Exp. Psy.* to have received more cumulative citations than a randomly selected paper published in *Brain Cogn.* is 0.88.

We rank journals in different fields according to both *q̅*(*J*) and the JIF. Figure 3 illustrates the effectiveness of these two ranking schemes for separating papers into different journals based on their impact. In Appendix S3, we provide rankings and the value of the multi-class AUC statistic for all fields. Our analysis demonstrates that the ranking scheme defined by *q̅*(*J*) is very similar to the optimal ranking.

**Figure 4:**
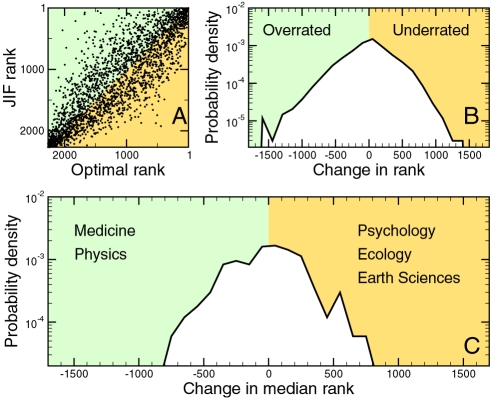
Effect of JIF biases on the ranking of journals. (A) Comparison of the rankings of journals obtained using the JIF and the AUC statistic. Though there are clear correlations between the two rankings, deviations can be extremely large. (B) Probability density function of ΔR(*i*) = R^JIF^(*i*)−R^AUC^(*i*). Positive values of ΔR indicate under-rating of the journal. (C) Probability density function of change in the median ranking of the journals primarily classified in a given field, for fields with at least two journals. The papers published in journals classified in fields that are over-rated tend to get cited quickly (probably because of faster publication times), whereas papers published in journals in under-rated fields take longer to start accruing citations. Table S1 lists the median change of rank for each field.

Our analysis also demonstrates that the *mean* number of citations and the JIF provide particularly inaccurate ranking schemes. This finding is particularly important because some journals and some fields benefit greatly in reputation from the biases in the JIF, while others are at a disadvantage (see Figure 4 and Tables 1 and 2).

**Table 1:** Rankings for the field of ecology.

Rank			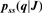		n			Steady state
AUC	JIF	Journal abbreviation	q̅	*σ*	n̅	Q2	JIF	period
1	1	ECOLOGY	1.75	0.33	71.1	52	4.782	1974–1994
2	2	AM NAT	1.72	0.40	80.4	48	4.660	1967–1992
3	4	EVOLUTION	1.67	0.35	69.8	43	4.292	1973–1993
4	14	BEHAV ECOL SOCIOBIOL	1.60	0.31	44.4	36	2.316	1978–1990
5	8	J ANIM ECOL	1.57	0.34	47.5	33	3.390	1954–1996
6	5	J ECOL	1.55	0.35	45.1	32	4.239	1973–1996
7	15	MAR ECOL-PROG SER	1.47	0.31	33.6	26	2.286	1991–1995
8	6	CONSERV BIOL	1.42	0.42	37.5	23	3.762	1988–1998
9	7	FUNCT ECOL	1.42	0.32	29.6	23	3.417	1989–1996
10	9	OIKOS	1.41	0.35	34.2	22	3.381	1974–1995
11	10	OECOLOGIA	1.40	0.29	27.5	22	3.333	1994–1997
12	17	J EXP MAR BIOL ECOL	1.31	0.30	23.5	18	1.919	1988–1995
13	3	J APPL ECOL	1.31	0.36	25.6	17	4.527	1965–2000
14	23	BIOTROPICA	1.30	0.38	25.5	17	1.391	1975–1994
15	13	J VEG SCI	1.28	0.36	22.4	16	2.382	1989–1999
16	22	POLAR BIOL	1.27	0.33	20.8	16	1.502	1981–1994
17	28	ENVIRON BIOL FISH	1.24	0.42	21.0	15	0.934	1981–1990
18	12	BIOL CONSERV	1.21	0.38	22.1	14	2.854	1988–1996
19	11	J BIOGEOGR	1.21	0.36	20.4	13	2.878	1976–1998
20	21	J WILDLIFE MANAGE	1.19	0.34	18.3	13	1.538	1984–1995
21	18	J CHEM ECOL	1.11	0.31	14.4	10	1.896	1995–1998
22	32	AM MIDL NAT	1.07	0.36	14.5	9	0.667	1964–1995
23	26	WILDLIFE RES	1.05	0.32	11.6	9	1.032	1990–1997
24	24	PEDOBIOLOGIA	0.99	0.41	12.8	8	1.347	1965–1997
25	20	AGR ECOSYST ENVIRON	0.97	0.44	11.4	7	1.832	1982–2001
26	19	ECOL MODEL	0.91	0.40	11.3	6	1.888	1977–1998
27	30	J RANGE MANAGE	0.90	0.37	9.9	6	0.859	1966–1995
28	29	BIOCHEM SYST ECOL	0.89	0.36	9.2	6	0.906	1980–1994
29	31	WILDLIFE SOC B	0.87	0.40	8.7	6	0.843	1983–1998
30	25	J ARID ENVIRON	0.83	0.38	7.8	5	1.238	1989–2000
31	34	SOUTHWEST NAT	0.72	0.38	5.9	4	0.309	1980–1994
32	33	J NAT HIST	0.72	0.38	6.4	4	0.631	1966–2000
33	35	CAN FIELD NAT	0.69	0.40	5.8	3	0.073	1983–1993
34	16	LANDSCAPE URBAN PLAN	0.67	0.41	5.3	3	2.029	1985–2004
35	27	J SOIL WATER CONSERV	0.65	0.49	7.0	3	0.949	1966–2002
36	36	NAT HIST	-0.32	0.44	0.3	0	0.059	1989–2005

We consider the 36 journals that are primarily classified in the field of ecology according to the ISI. We rank journals according to: (i) the maximization of the multi-class AUC statistic for the steady-state distributions 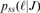 and (ii) the JIF; *q̅* and *σ* are the model parameters obtained using 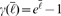; *n̅* and *Q*2 are the mean and median number of citations in the steady state. We also show the steady-state period.

**Table 2:** Rankings for the field of experimental psychology.

Rank			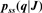		n			Steady state
AUC	JIF	Journal abbreviation	q̅	*σ*	n̅	Q2	JIF	period
1	4	J EXP PSYCHOL LEARN	1.55	0.35	47.5	34	2.601	1992–1995
2	6	J EXP PSYCHOL HUMAN	1.56	0.38	52.1	32	2.261	1974–1995
3	2	PSYCHOPHYSIOLOGY	1.47	0.36	41.8	27	3.159	1985–1995
4	1	NEUROPSYCHOLOGIA	1.48	0.41	48.6	27	3.924	1964–1995
5	10	MEM COGNITION	1.38	0.40	34.0	21	1.512	1977–1997
6	5	BRAIN LANG	1.25	0.33	22.9	16	2.317	1992–1997
7	12	J EXP ANAL BEHAV	1.22	0.38	23.8	14	1.221	1970–1991
8	11	PERCEPT PSYCHOPHYS	1.20	0.41	23.5	13	1.482	1965–1996
9	8	J EXP CHILD PSYCHOL	1.15	0.39	20.0	12	2.062	1963–1999
10	9	PERCEPTION	1.07	0.45	17.7	9	1.585	1973–1995
11	7	ACTA PSYCHOL	0.84	0.55	13.2	5	2.094	1955–2001
12	3	BRAIN COGNITION	0.73	0.61	9.1	3	2.858	1995–1999
13	13	PERCEPT MOTOR SKILL	0.54	0.42	4.5	2	0.333	1970–1995

We consider the 13 journals that are primarily classified in the field of experimental psychology according to the ISI. We rank journals according to: (i) the maximization of the multi-class AUC statistic for the steady-state distributions 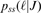 and (ii) the JIF; *q̅* and *σ* are the model parameters obtained using 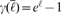; *n̅* and *Q*2 are the mean and median number of citations in the steady state. We also show the steady-state period.

The bias introduced by the JIF arises directly from the major methodological problems raised against using citation analysis to evaluate journals. First, the mean number of citations to papers published in a journal is not representative of the number of citations to each individual paper [11], a point that our analysis systematically confirms. However, we show that *q̅*(*J*) is representative of the *q* of the papers published in journal *J*, that being the reason why ranking according to *q̅*(*J*) is efficient. Second, citation behavior varies by field [11]. Our analysis again confirms this. Nevertheless, we show that by comparing the steady-state behavior of a set of journals and keeping comparisons to within fields, one can accurately rank a set of journals.

Our findings provide a quantitative measure of the efficacy of academic journals, through the work of editors and reviewers, at organizing research based on their prediction of the ultimate impact of that research. Even though far from perfect, the journal system and the ranking of journals provides a powerful heuristic with which to locate the research that will ultimately have the largest impact.

## Methods

### Identifying steady-state regions

We use the time evolution of 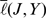 to identify transient and steady-state periods. (Figures 1**C,D**) In the steady state, 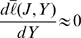 whereas in the transient period 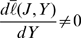. Because of the noisy fluctuations in the time series, we use a moving average considering the five previous years of the derivative. We define the duration of the transient regime as *τ* = 2006−*Y*_0_, where *Y*_0_ is largest value of *Y* for which the moving average is <0.005.

We also determine the periods during which the citation distribution is stable. To this end, we compare the citation distribution for all pairs of years using the Kolmogorov-Smirnov test and fit a box-diagonal model to the matrix of *p*-values. We then identify the periods for which we cannot reject the hypothesis that the citation distribution is stationary [28]. The distribution that we use for comparison is the most recent stationary period before *Y*_0_.

### Estimating μ, σ, and γ for a journal

For each steady-state citation distribution, our model (Eq. 3) has three parameters that must be estimated: *μ*, *σ*, and *γ*. To the best of our knowledge, no maximum likelihood estimation procedures exist for the parameters of this model, so we estimate the parameters by minimizing the *χ* ^2^ statistic (see Appendix S4 for plots of all the fits)
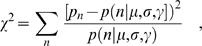
(4)where *p_n_* is the fraction of papers with *n* citations, and 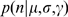 is the probability of having a paper with *n* citations according to our model (Eq. 3) 
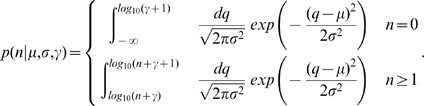
(5)


In practice, we bin the empirical data so that we have at least ten data points in each bin. This is especially important for the tails of the distribution. Then, the contribution to *χ* ^2^ is
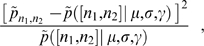
(6)where 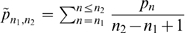, and 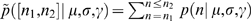.

The fitting parameters suggest that *σ* has a slight dependency on 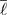 (Figure **2B**). In contrast, we find that there is a strong dependency of *γ* on 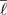 (Figure **2C**)
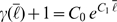
(7)with *C*_0_ = 0.91±0.02 and *C*_1_ = 1.03±0.02. For simplicity, when comparing properties of the empirical distributions to model predictions (Figures **2D–G**), we assume that *σ* = 0.419 and that 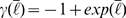. Assuming these two dependencies, one can then obtain a relationship between *μ* and 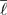 as
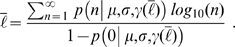
(8)


As shown in Figure **2C**, the estimated value of *γ* displays large fluctuations to which the remaining parameters in the fit (*μ*,*σ*) are very sensitive. In order to obtain a less noisy estimate for those parameters, we fix *γ* using the relationship in Eq. 7, and estimate *μ* and *σ* by minimizing *χ* ^2^. The estimate we obtain for *μ* = *q̅* is the one we use for ranking journals (Figure **3** and Tables **1**, **2**).

### Calculating multi-class AUC

We define the best ordering as the one that maximizes the value of the multi-class AUC statistic. For a set of journals 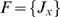 and a journal ranking **R**, we define the multi-class AUC statistic *M*(*F*,**R**) as [27]

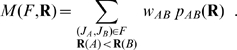
(9)


We denote as *p_AB_*(**R**) the probability that given a pair of papers (*a,b*) from journals *J_A_* and *J_B_* such that **R**(*A*)<**R**(*B*), then *q*(*a*)>*q*(*b*). We denote as *w_AB_* the weight we assign to each probability, which depends on the number of papers *N_A_* and *N_B_* published in journals *J_A_* and *J_B_* during the steady-state period, as follows
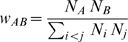
(10)


In principle, one could calculate the multi-class AUC statistic for every permutation of the ordering of journal citation distributions, and choose the ordering that gives the highest value. However, the number of permutations of a sequence of even modest size is unwieldy. Fortunately, in almost all cases, the distributions obey the property of transitivity, that is, if *a>b* and *b>c*, then *a>c*, which simplifies the optimization task. In the few cases where the transitivity condition does not hold, we resort to brute-force optimization, and resolve the ambiguity in the ordering by permuting the order of each distribution and finding the permutation that maximizes the multi-class AUC statistic.

## Supporting Information

Appendix S1 Supporting information text, and description of other supporting information files.
(0.06 MB PDF)

Appendix S2 Citation history for the 2,266 journals included in our analysis in alphabetical order. For a detailed description of the plots see the caption of panel C in Figure 1.
(19.10 MB PDF)

Appendix S3 Comparison of ranking schemes for all the fields listed in the WoS.
(12.95 MB PDF)

Appendix S4 Fit to the steady-state citation distribution for the 2,266 journals included in our analysis in alphabetical order.
(21.06 MB PDF)

Table S1 Median change of rank from JIF to optimal ranking for all fields with at least two journals with more than 50 articles published during the steady-state period.
(0.00 MB TXT)
