# Supplementary material for: Effectiveness of Journal Ranking Schemes as a Tool for Locating Information
Source: PLoS One. 2008 Feb 27;3(2):e1683. doi: 10.1371/journal.pone.0001683 (PMC2244807; doi:10.1371/journal.pone.0001683)
Supplement: Appendix S1 — Supporting information text, and description of other supporting information files. (0.06 MB PDF) [file pone.0001683.s001.pdf]

Supplementary Material

Michael J. Stringer, Marta Sales-Pardo, Luís A. Nunes Amaral

Data

We study the citation data associated with approximately 23 million papers $(22,951,535)$ from the Institute for Scientific Information (ISI) Web of Science (WoS) database. We downloaded the data using a web interface available to those with a subscription to the service from Thomson Scientific (http://www.isiwebofknowledge.com). We used all of the following subsets from the WoS database:

- Science Citation Index (SCI) from 1955 – 2006
- Social Science Citation Index (SSCI) from 1956 – 2006
- Arts & Humanities Citation Index (AHCI) from 1975 – 2006

The ISI classifies research publications based on the content type, for example editorial material, meeting abstract, book review, paper, etc... We restrict the citation analysis to the publications marked as *Article*. Further descriptions of classification types and descriptions can be obtained in the help section of the ISI Web of Science website, under the heading *Document Types*.

Within each of the citation indexes, the ISI classifies journals as belonging to subfields. We used the subfield classification available in the 2006 editions of both the Science Edition and the Social Sciences Edition of the ISI Journal Citation Reports (JCR). Detailed information about the subfield classifications are available on the ISI Journal Citation Reports website, under the heading *Scope Notes*.

Several authors have pointed out that the WoS coverage has biases toward science and engineering and toward English language publications. Concerning these issues, we make two observations. First, citations across fields, such as a science and engineering paper citing an arts and humanities paper, are rare, so the former bias is likely negligible. Second, English has become the *lingua franca* of science and engineering, such as Greek or Latin were centuries

ago. Research not published in English does not get read and thus has little chance of making an impact.

Probability Ranking Principle

This principle is used in the context of ranking documents on relevance to a query. However, to our knowledge this principle has not been used to rank a collection of documents, rather than a single document. In such case, one can extend this principle in one of two ways. The first one is to interpret the optimal ranking $\mathbf{R}$ as the one that maximizes the probability of picking a "good" paper, where good is defined as a paper from the top α-quantile from the global distribution of papers. Thus, different rankings could be obtained depending on the value of α used. The second way to realize the Probability Ranking Principle for journals is to maximize the probability that, given two papers a and b published in journals J_A and J_B, respectively, $q_a > q_b$ given that $\mathbf{R}(A) < \mathbf{R}(B)$. This latter probability is known as the multi-class AUC statistic and is used as a non-parametric way of evaluating how good a classification rule is in machine learning tasks where a fitness function for the classification performance is difficult (or undesirable) to define (*1*). Because $p_{ss}(\ell|J)$ is roughly Gaussian with constant variance, these two forms of ranking are similar, however we choose the latter for simplicity, and because the lack of any parameters eliminates the possibility of introducing bias in this step. This ranking scheme is appropriate to use as a standard because it does not depend on any particular form of the function that maps quality onto number of citations, as long as it is monotonically increasing (it is better to have more citations).

Ranking according to this principle ensures that it will always be more probable to select a paper with higher number of citations from a journal that is ranked higher, i.e. the AUC statistic is always $>$ 0.5. In Fig. 3 of the main text, we show the results of calculating the AUC statistic for every pair of journals to demonstrate the efficiency of different ranking schemes. Ordering by $\overline{q}(J)$ gives results that are very close to the optimal ordering, while the mean is the least effective way of ordering the distributions that we included in our comparison. Another feature of the data illustrated in Fig. 3 of the main text is that the most significant source of error in the ranking arises from failing to take into account the difference in time scale for citations to journals to reach the steady state. The fact that the mean is a poor estimator of central tendency for the distributions, while still important, accounts for much less of the error.

Description of supplementary material files

- **Appendix S2** Time dependence of $\bar{\ell}(Y, J)$ for the 2,266 journals included in our analysis in alphabetical order. For a detailed description of the plots see the caption of panel **C** in Fig. 1. **This file is 91 pages long.**

- **Appendix S3** Fit to the steady-state citation distribution for the 2,266 journals included in our analysis in alphabetical order. We show the fits and estimated parameters for: (1) minimising χ^2 and estimating μ_1, σ_1, and γ_1 (solid line); (ii) minimising χ^2 using the relationship $\gamma_2 = \gamma(\bar{\ell}) = e^{\bar{\ell}} - 1$ and estimating μ_2 and σ_2 (dashed line). For details on the fitting procedure see Methods. **This file is 91 pages long.**

- **Appendix S4** Comparison of ranking schemes for all the fields listed in the WoS. We consider all the fields with at least two journals primarily classified in that field, and that have more than 50 papers published in the steady-state period. For each field, we compare the optimal journal ranking $\mathbf{R}^{\text{AUC}}$ (that is, the ranking that maximises the multi-class AUC statistic) to $\mathbf{R}^{\bar{q}}$, the ranking obtained according to the estimate of $\bar{q}$ from the fit with fixed γ (see Methods), and $\mathbf{R}^{\text{JIF}}$. For each ranking, we show the matrix of probabilities p_{ij}, in which p_{ij} is the probability that if one picks a pair of papers (i, j) from journals J_i and J_j, $q(i) > q(j)$ given that $\mathbf{R}(i) < \mathbf{R}(j)$. For each ranking scheme, we give the value of the multi-class AUC statistic $M(\mathcal{F}, \mathbf{R})$ (Eq. 10 in main manuscript). The table below the matrices lists the journals in that field, the optimal ranking, and the ranking obtained from the JIF. We also show: the parameters $\bar{q}$ and σ estimated from the fit of $p_{ss}(q, J)$ to the model (Eq. 3) using $\gamma(\bar{\ell}) = e^{\bar{\ell}} - 1$; $\bar{\mathbf{n}}$ and **Q2**, the mean and median number of citations in the steady-state distribution; and the years that correspond to the steady state. **This file is 248 pages long.**

- **Table S1** Spreadsheet showing the median change of rank from JIF to optimal ranking for all fields with at least two journals with more than 50 articles published during the steady-state period.

References and Notes

1. Hand DJ, Till RJ (2001) A simple generalisation of the area under the ROC curve for multiple class classification problems. Mach Learn 45: 171–186.
