# Supplementary figures and images for: Effectiveness of Journal Ranking Schemes as a Tool for Locating Information
Source: PLoS One. 2008 Feb 27;3(2):e1683. doi: 10.1371/journal.pone.0001683 (PMC2244807; doi:10.1371/journal.pone.0001683)

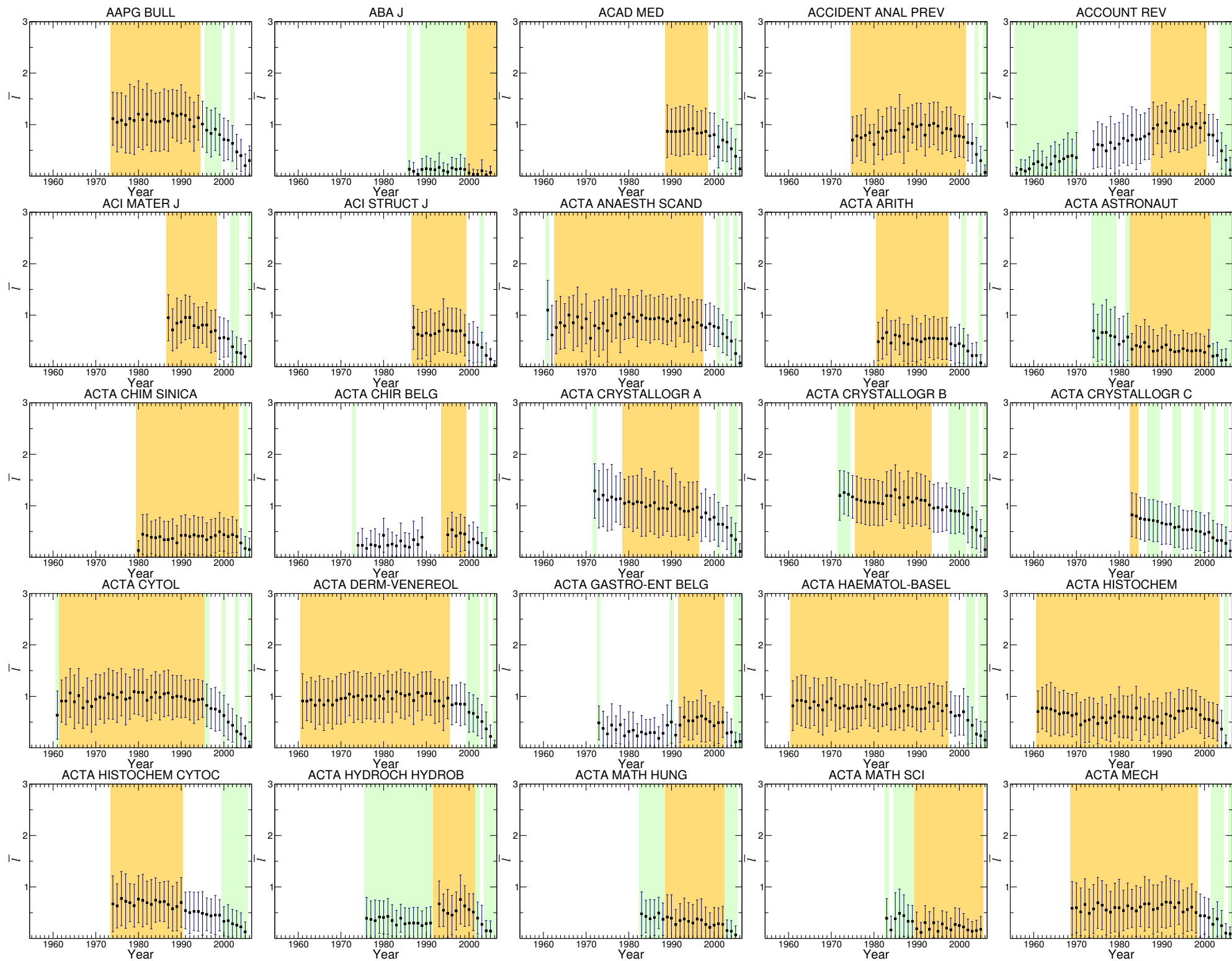

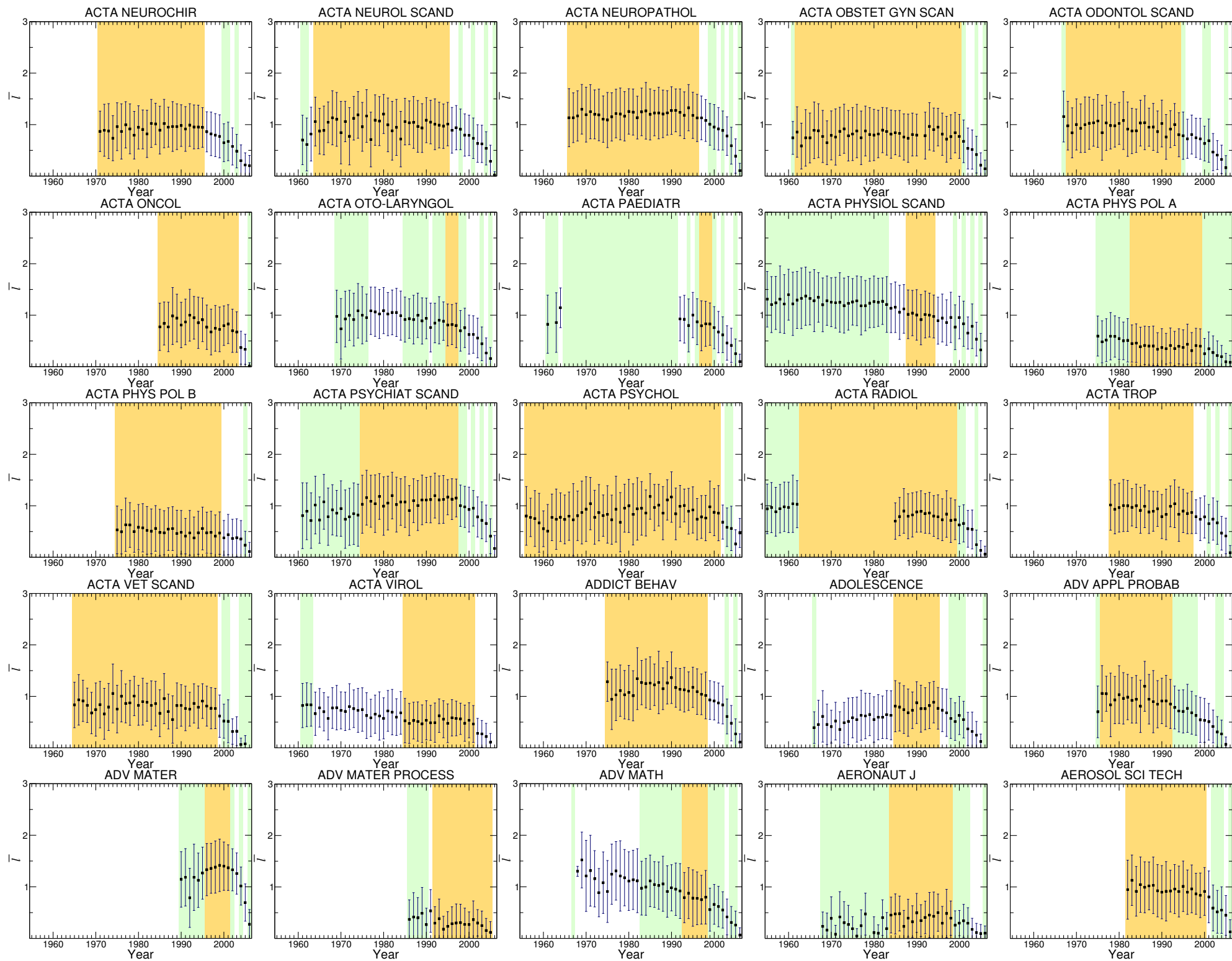

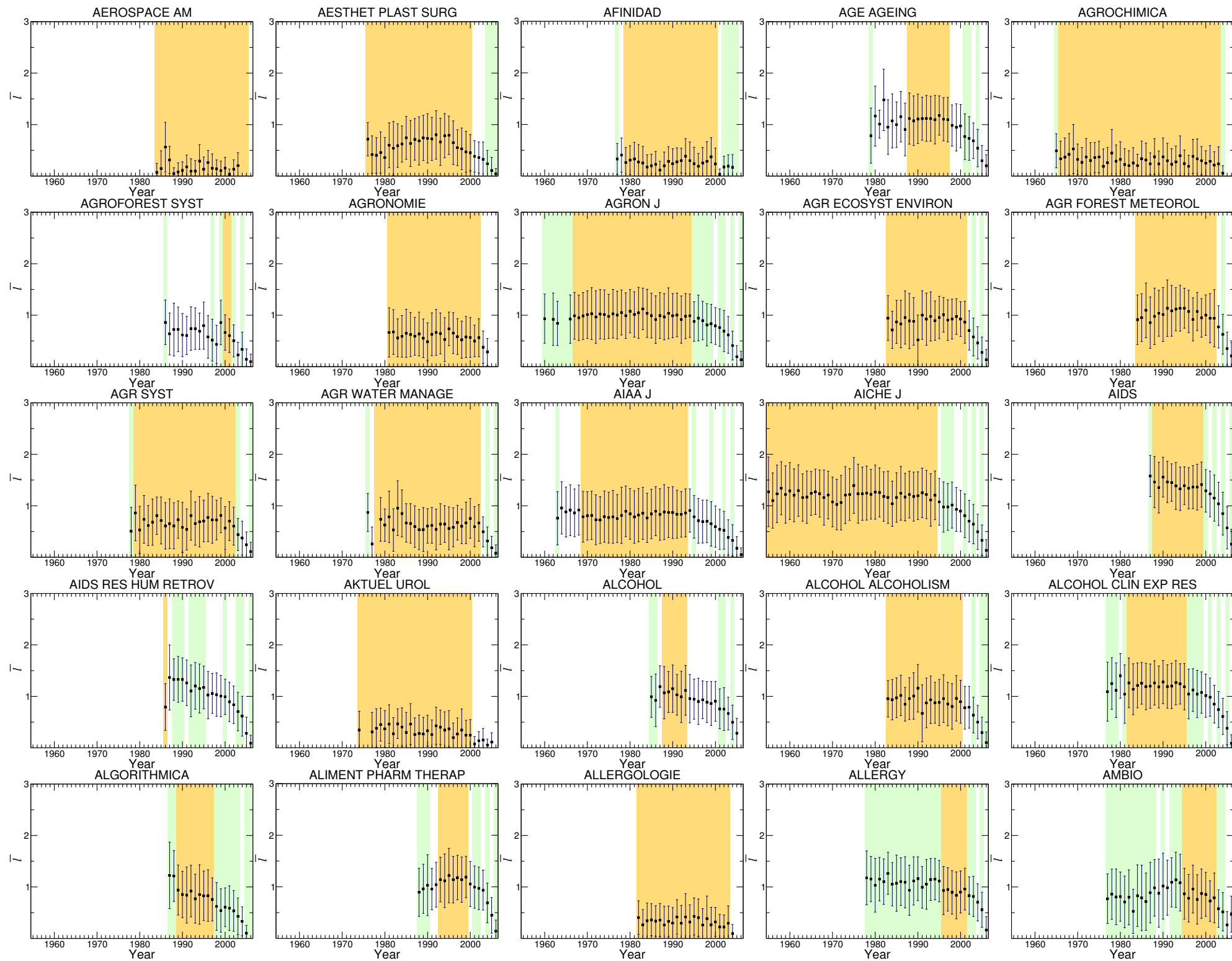

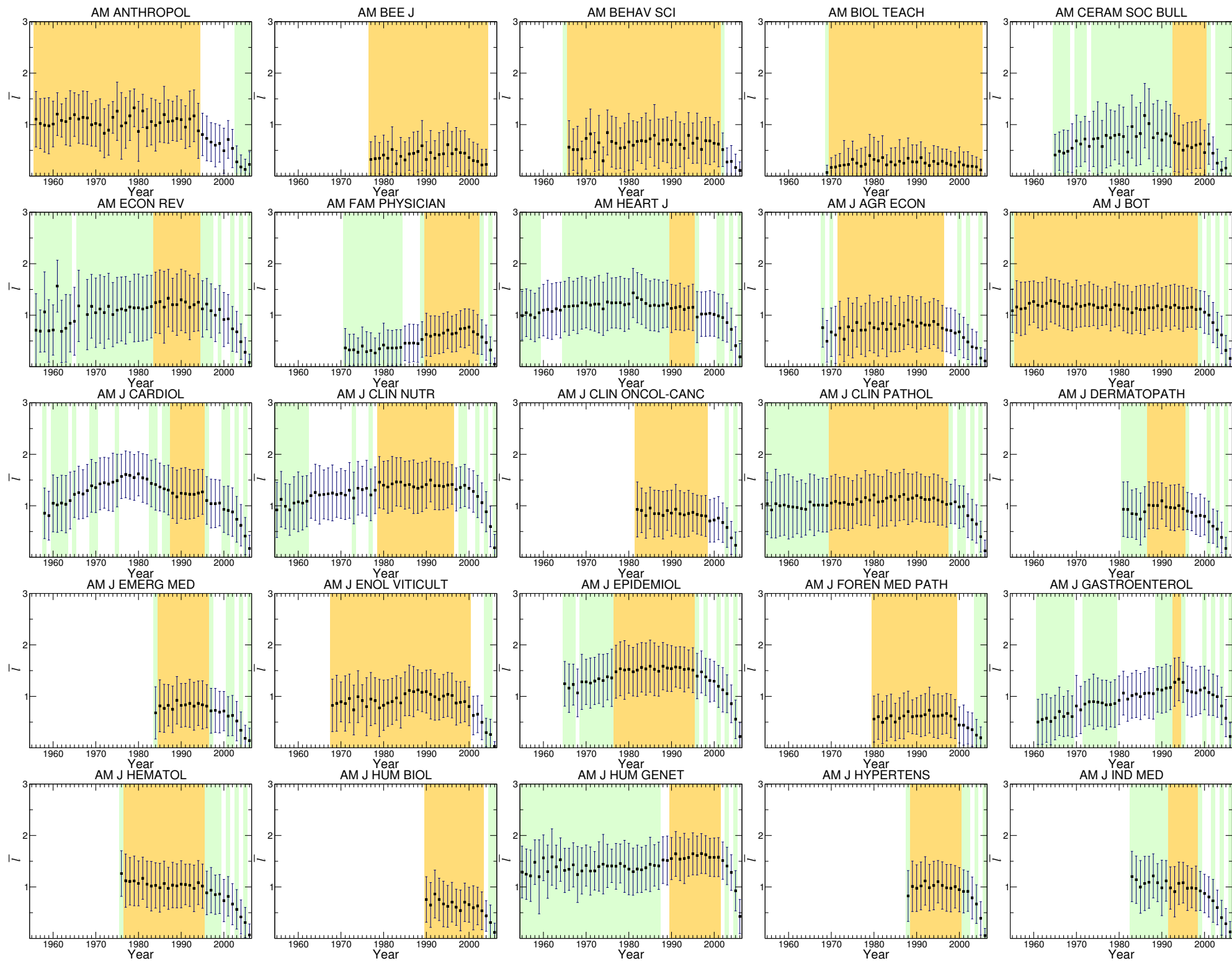

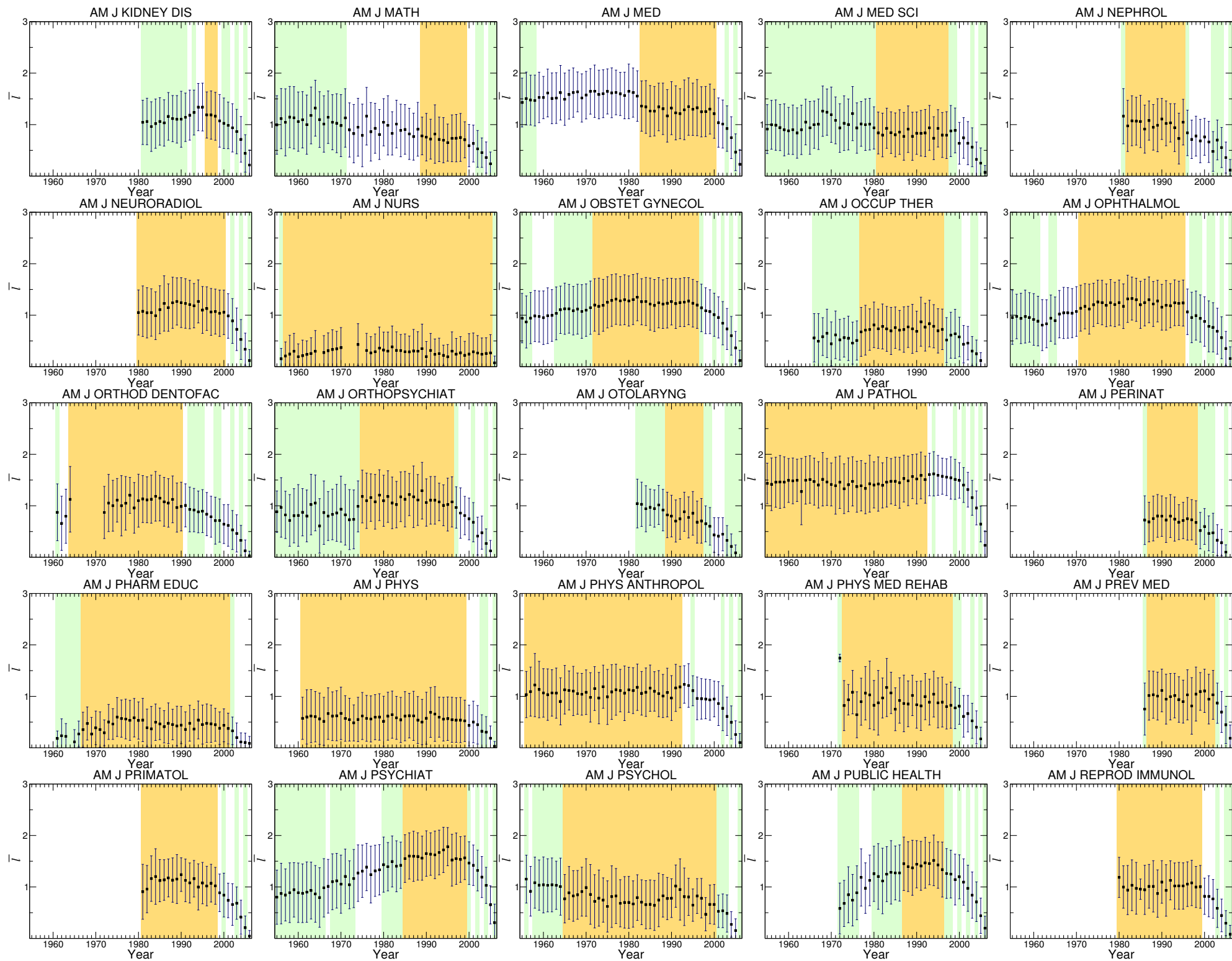

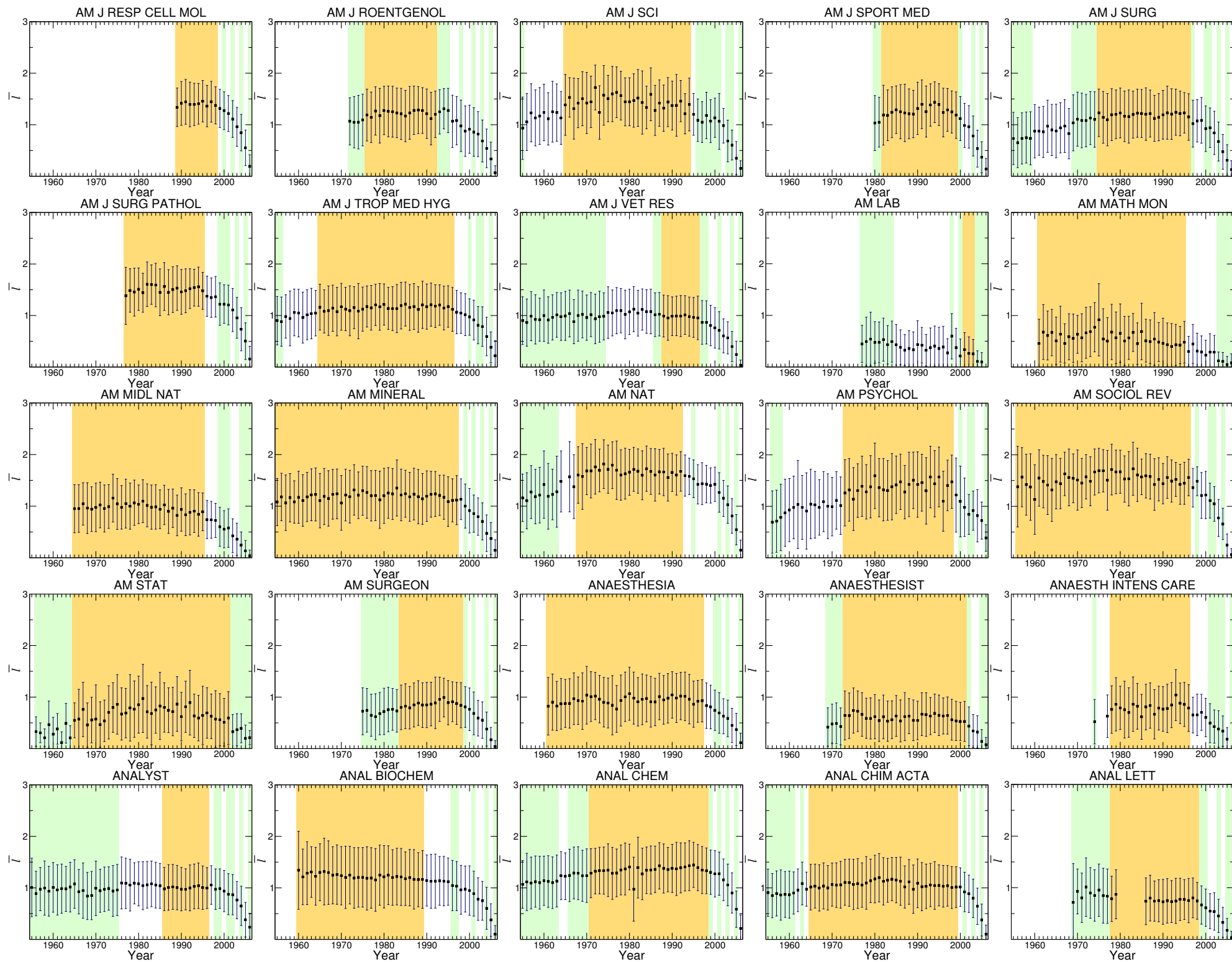

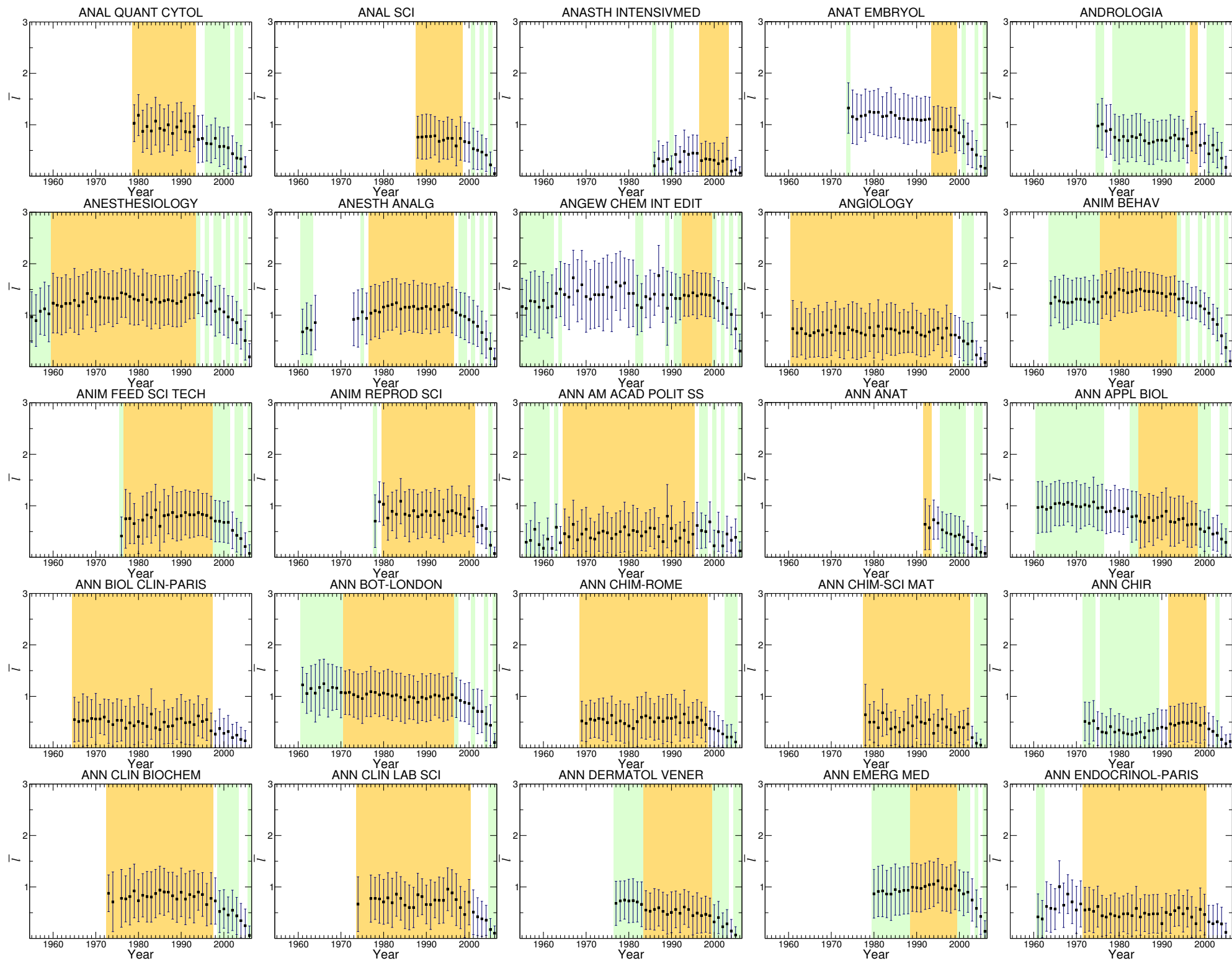

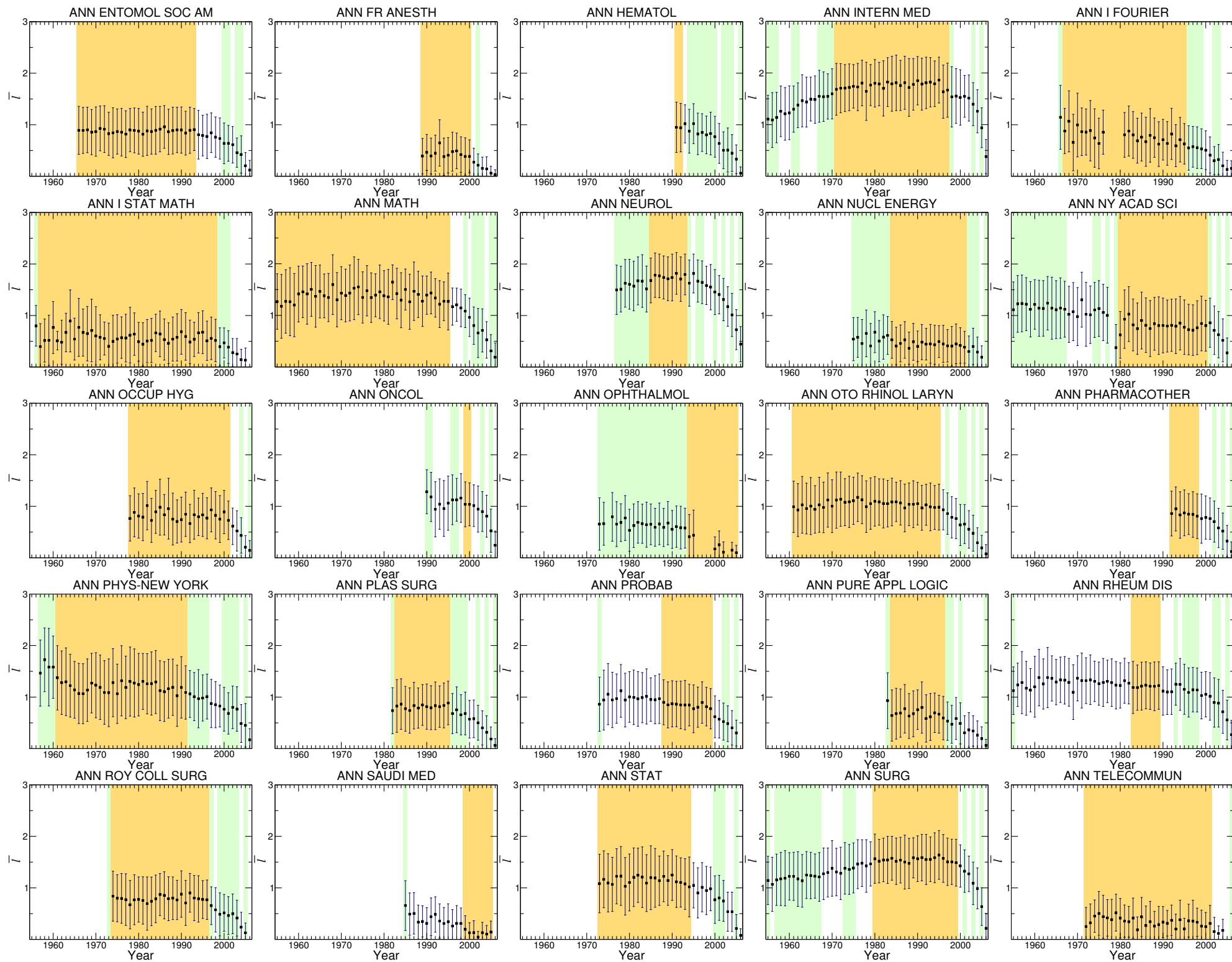

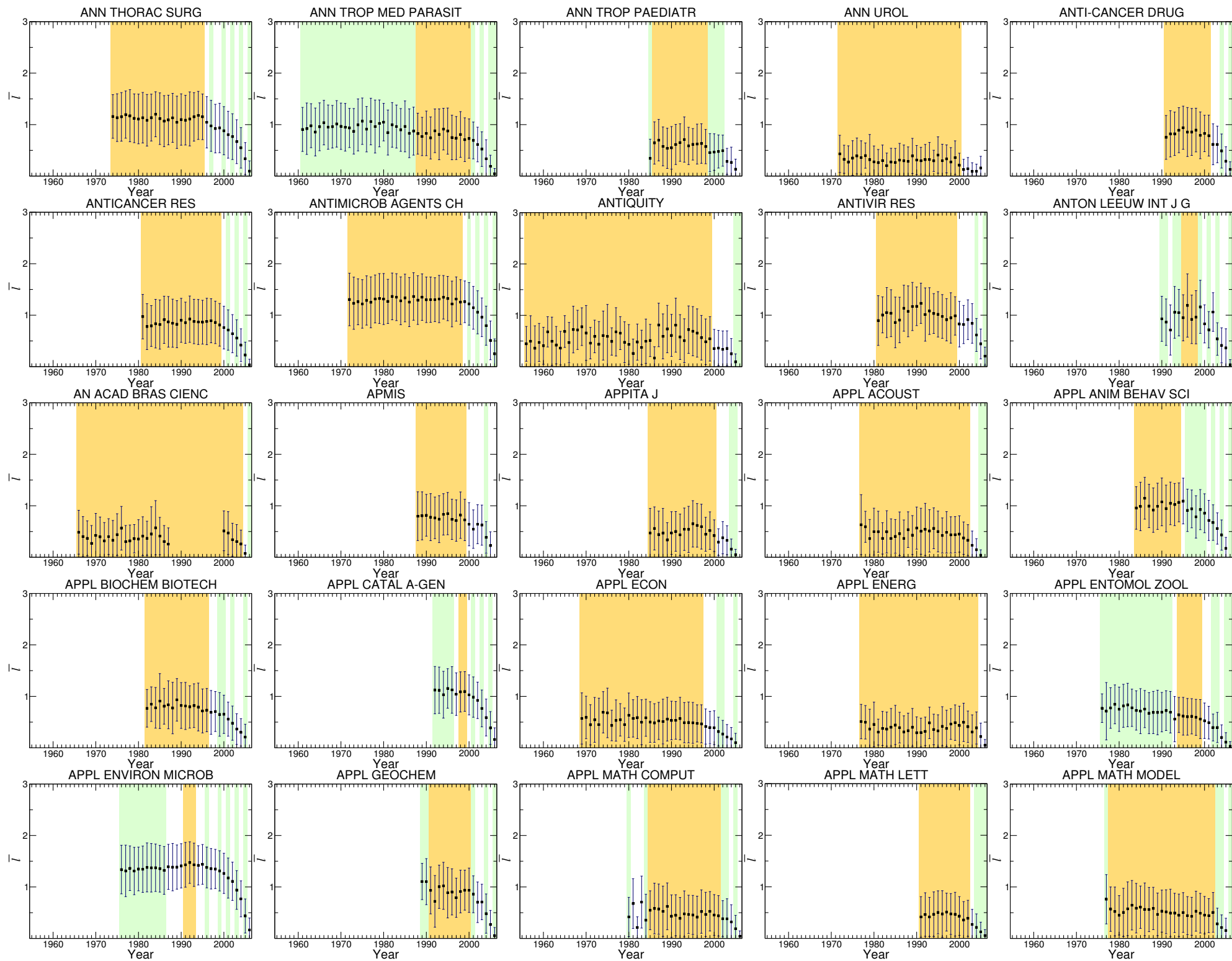

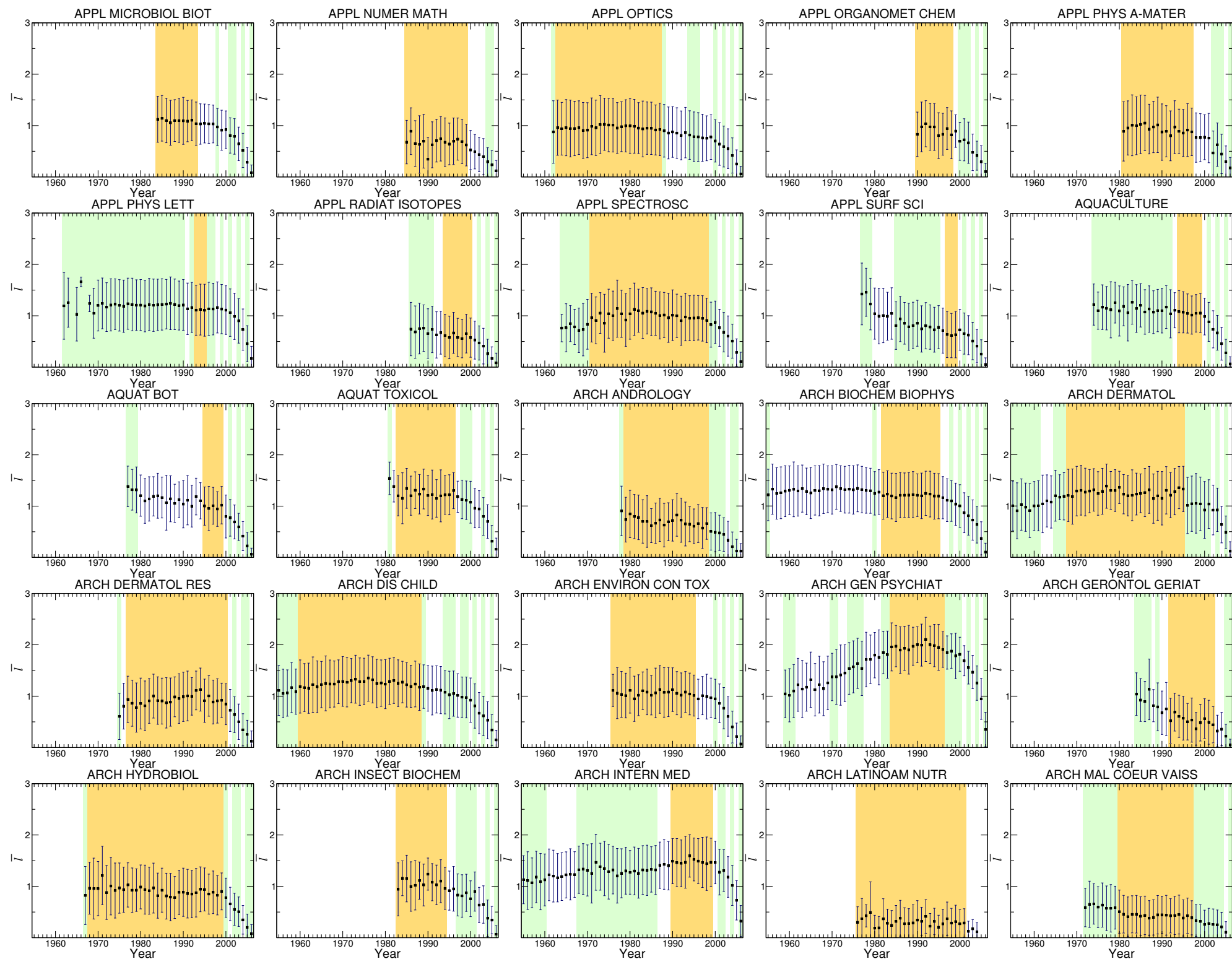

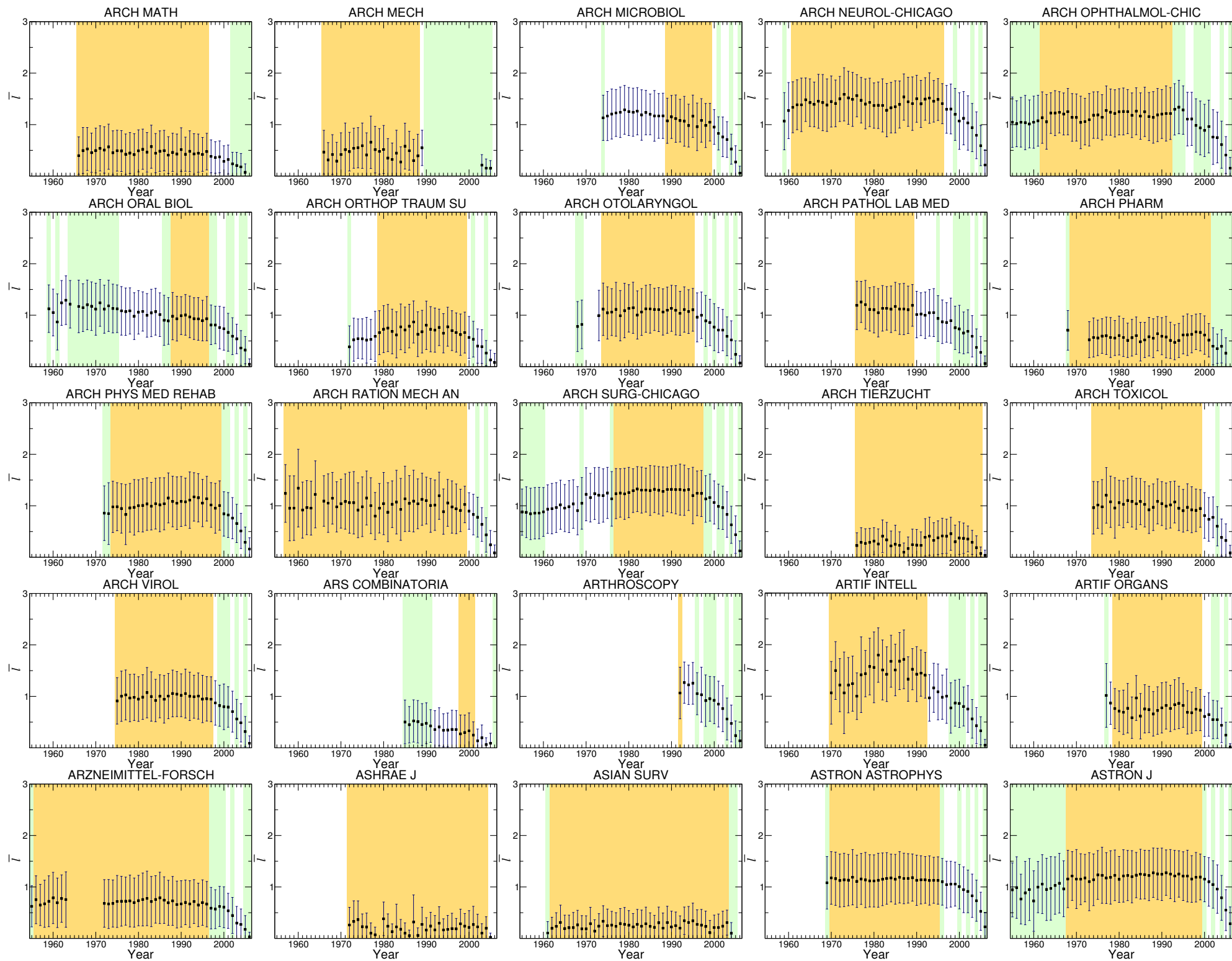

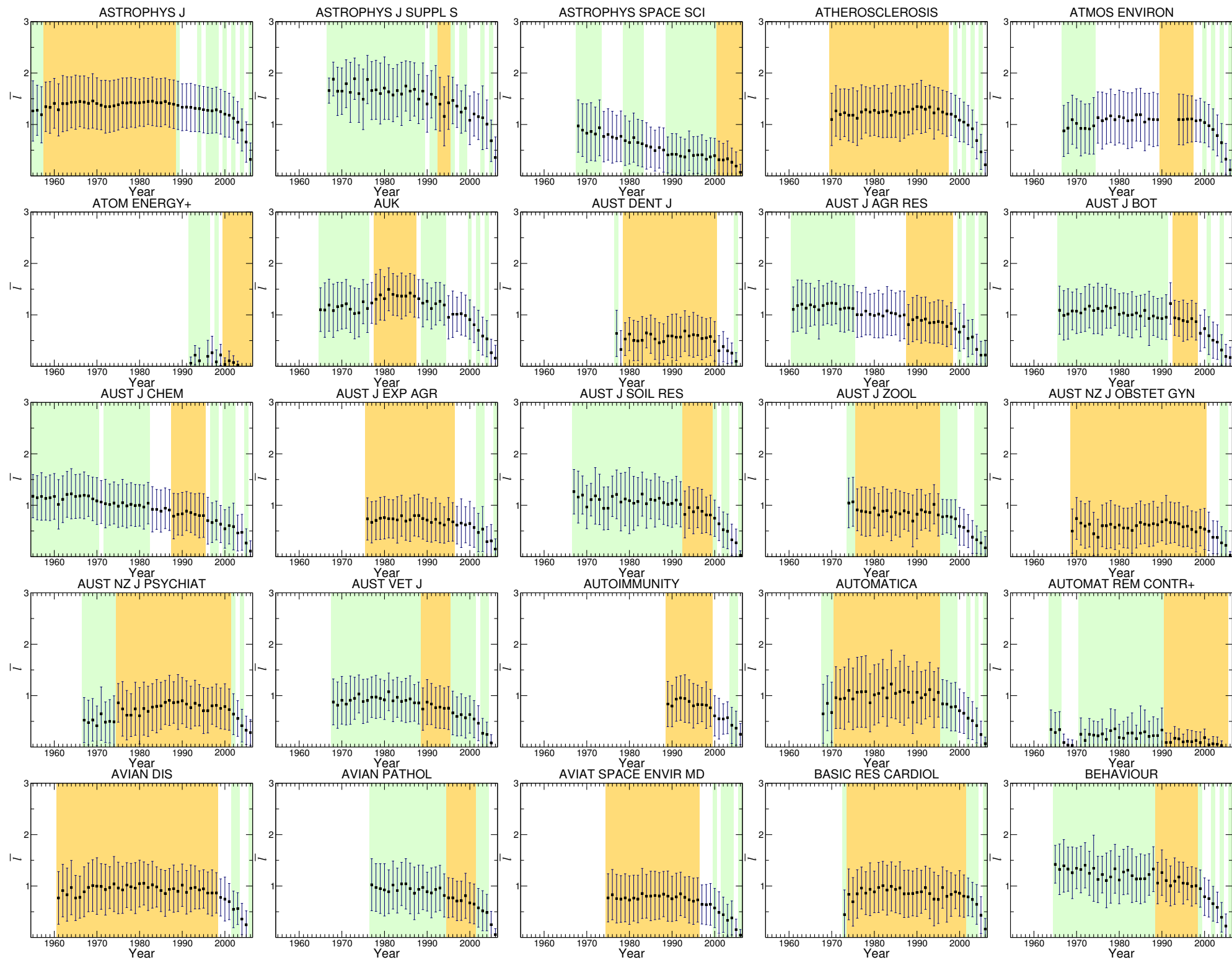

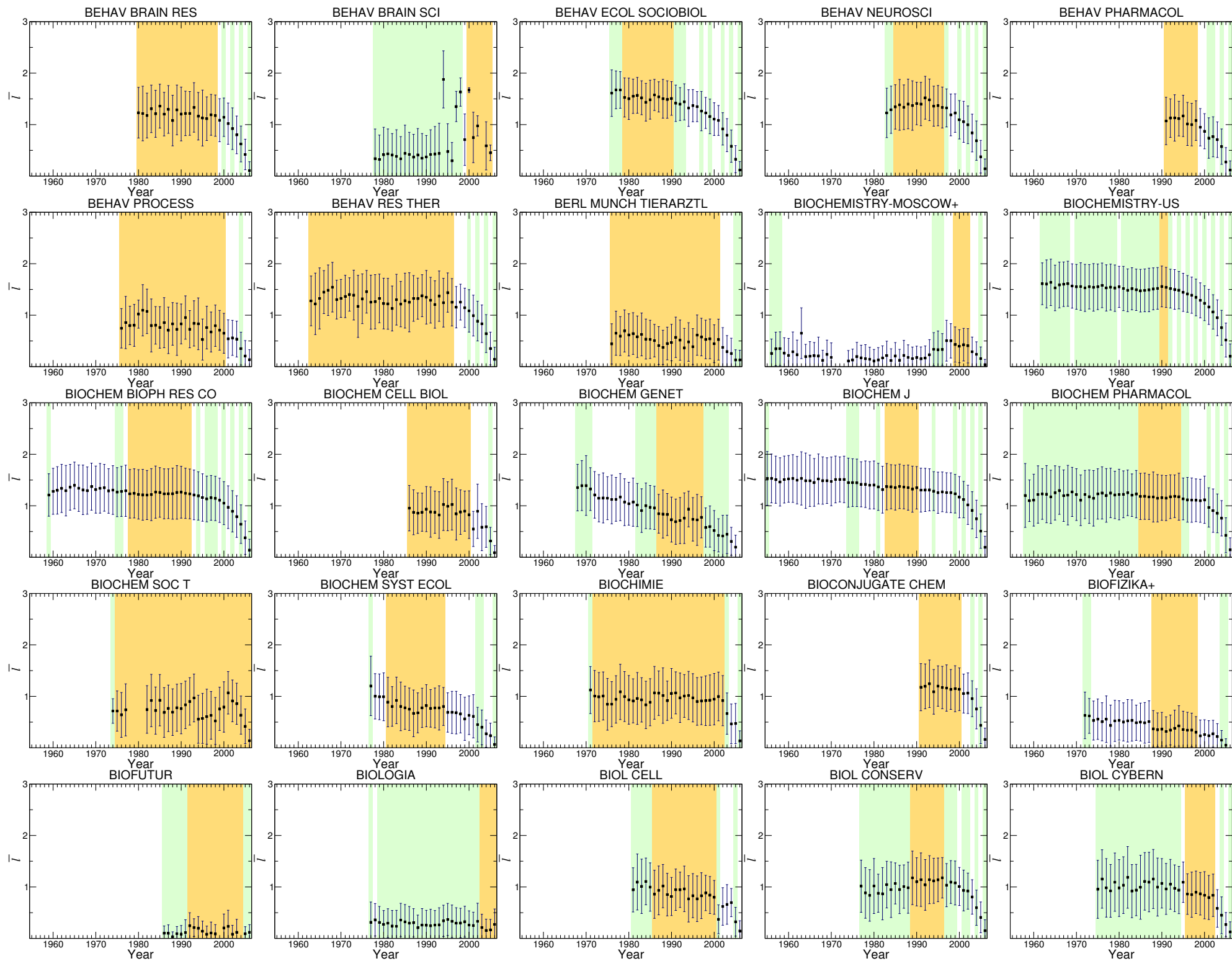

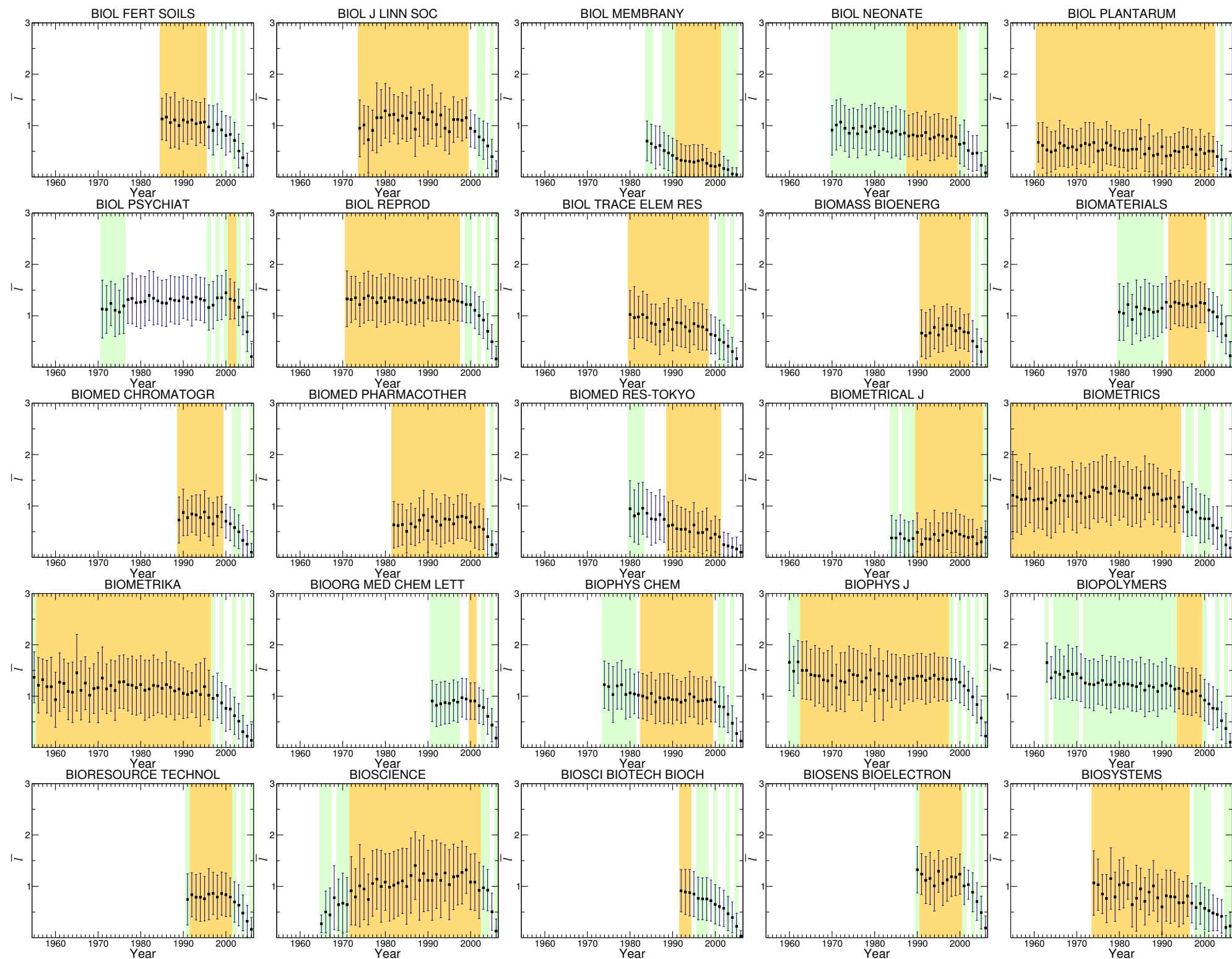

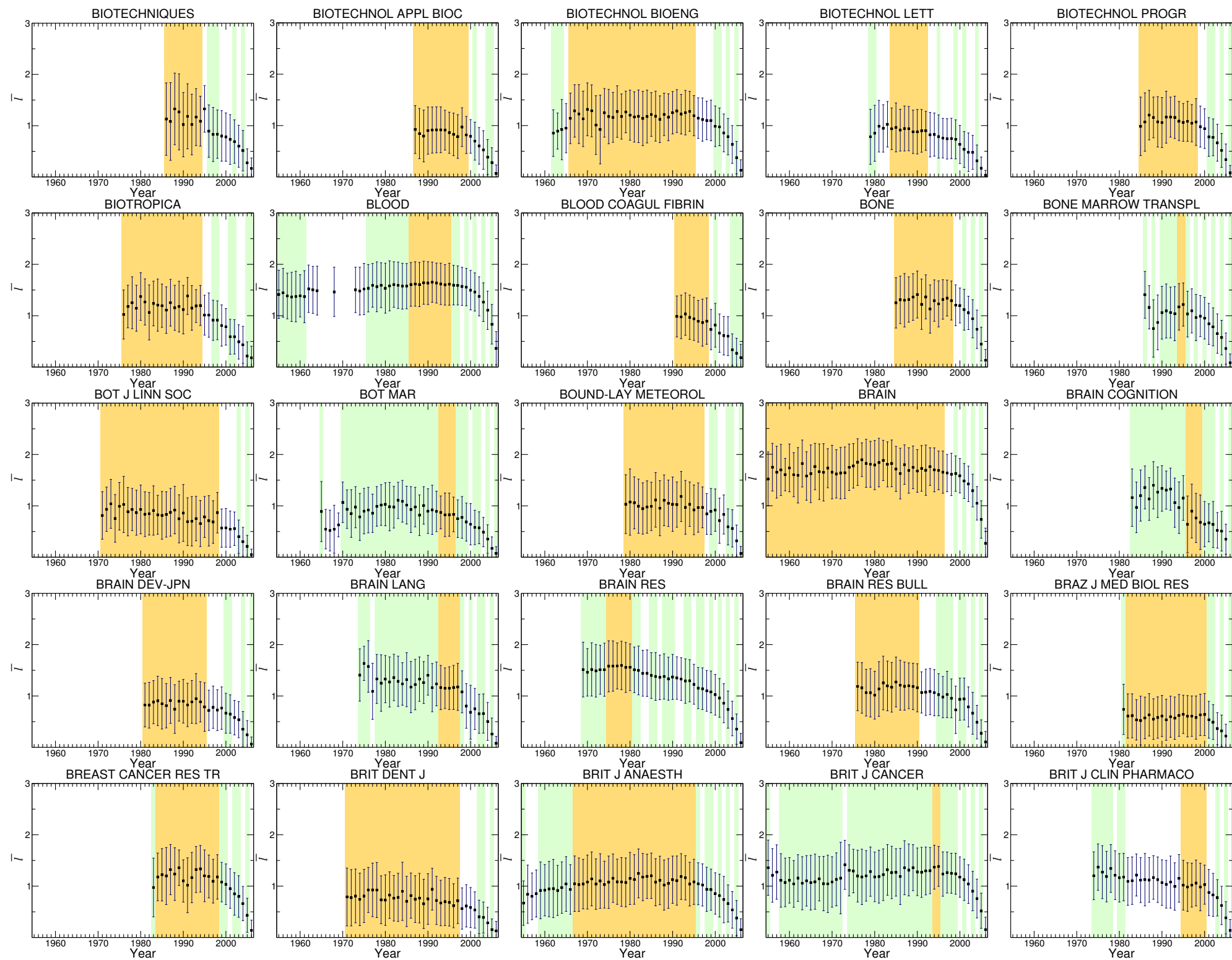

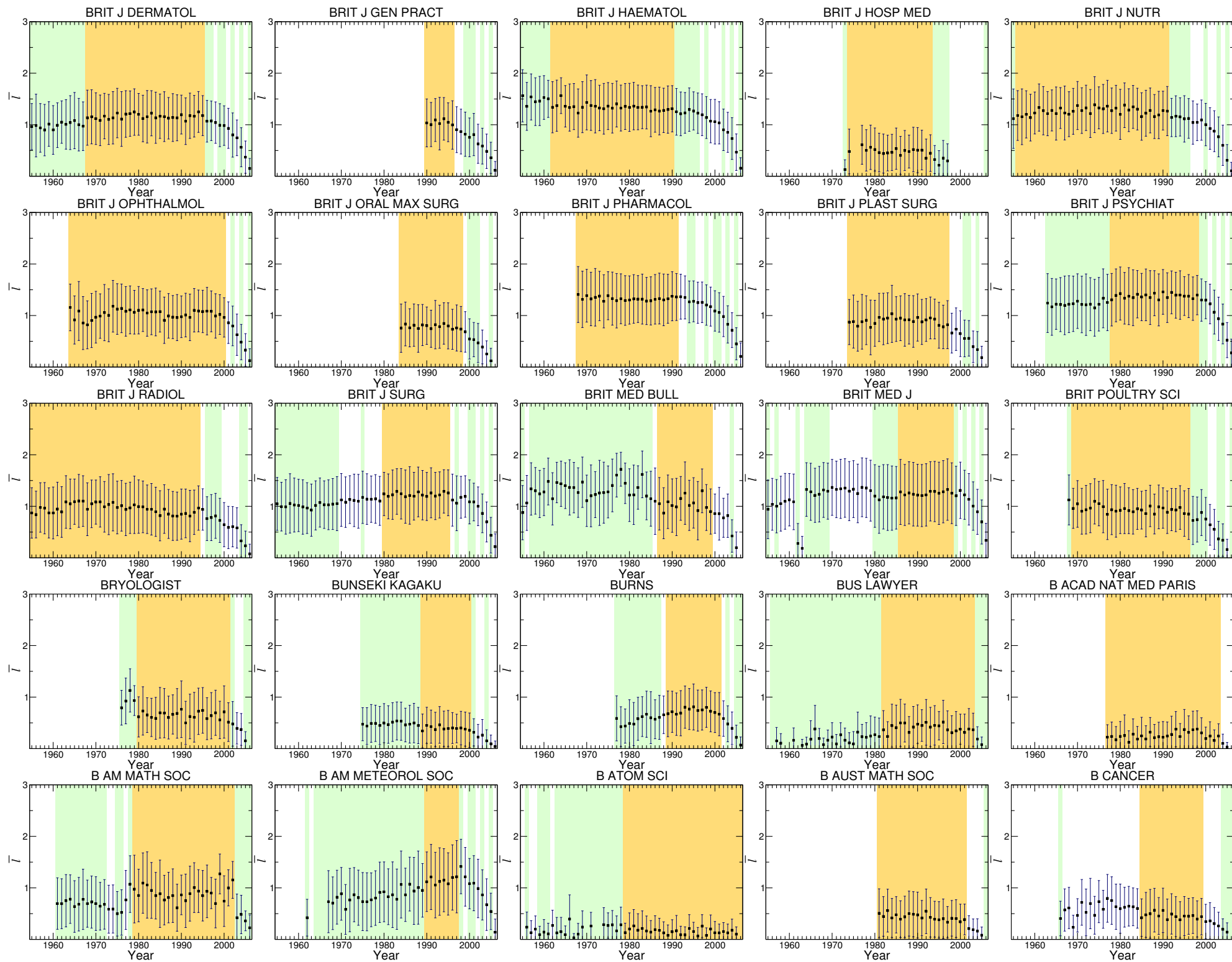

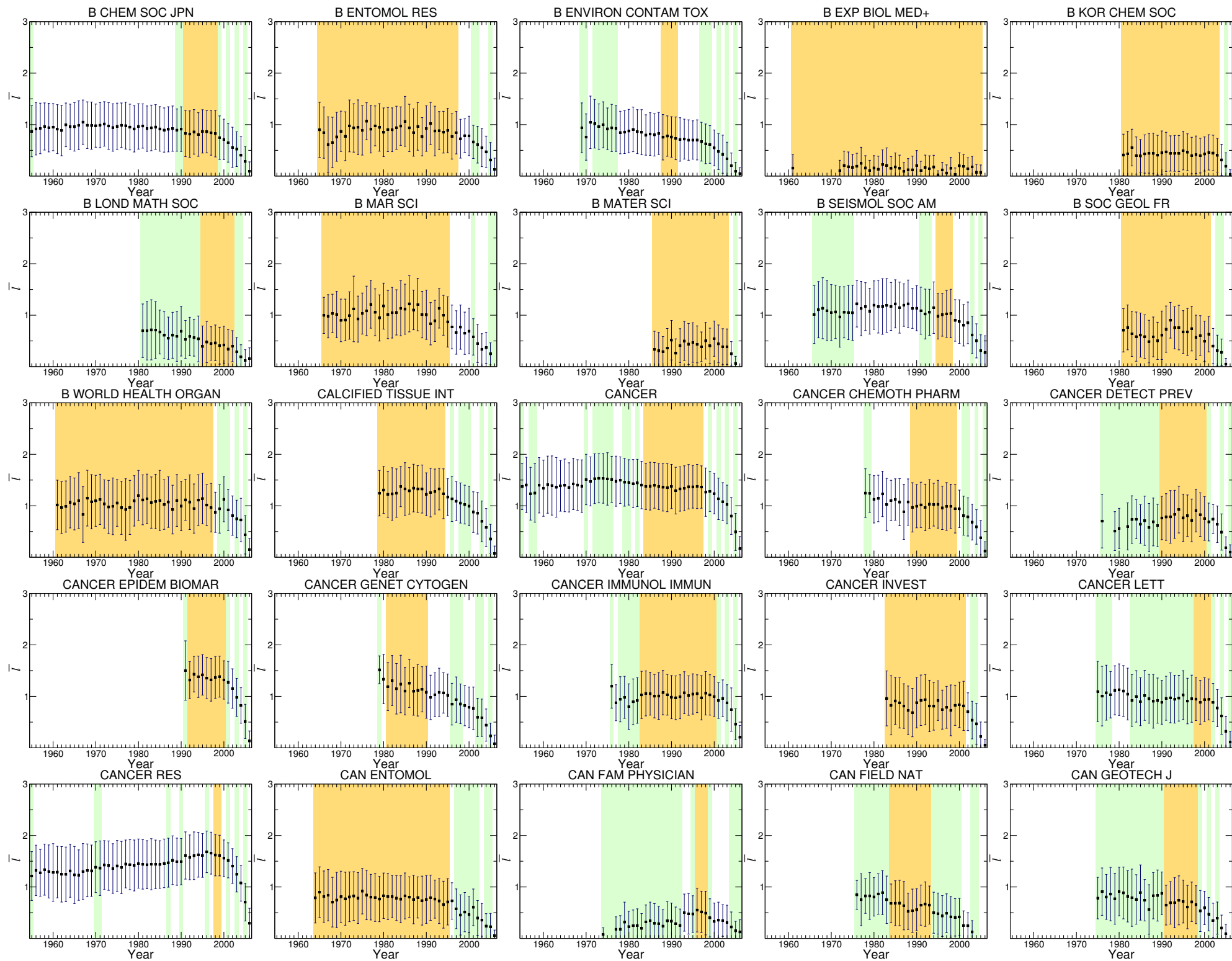

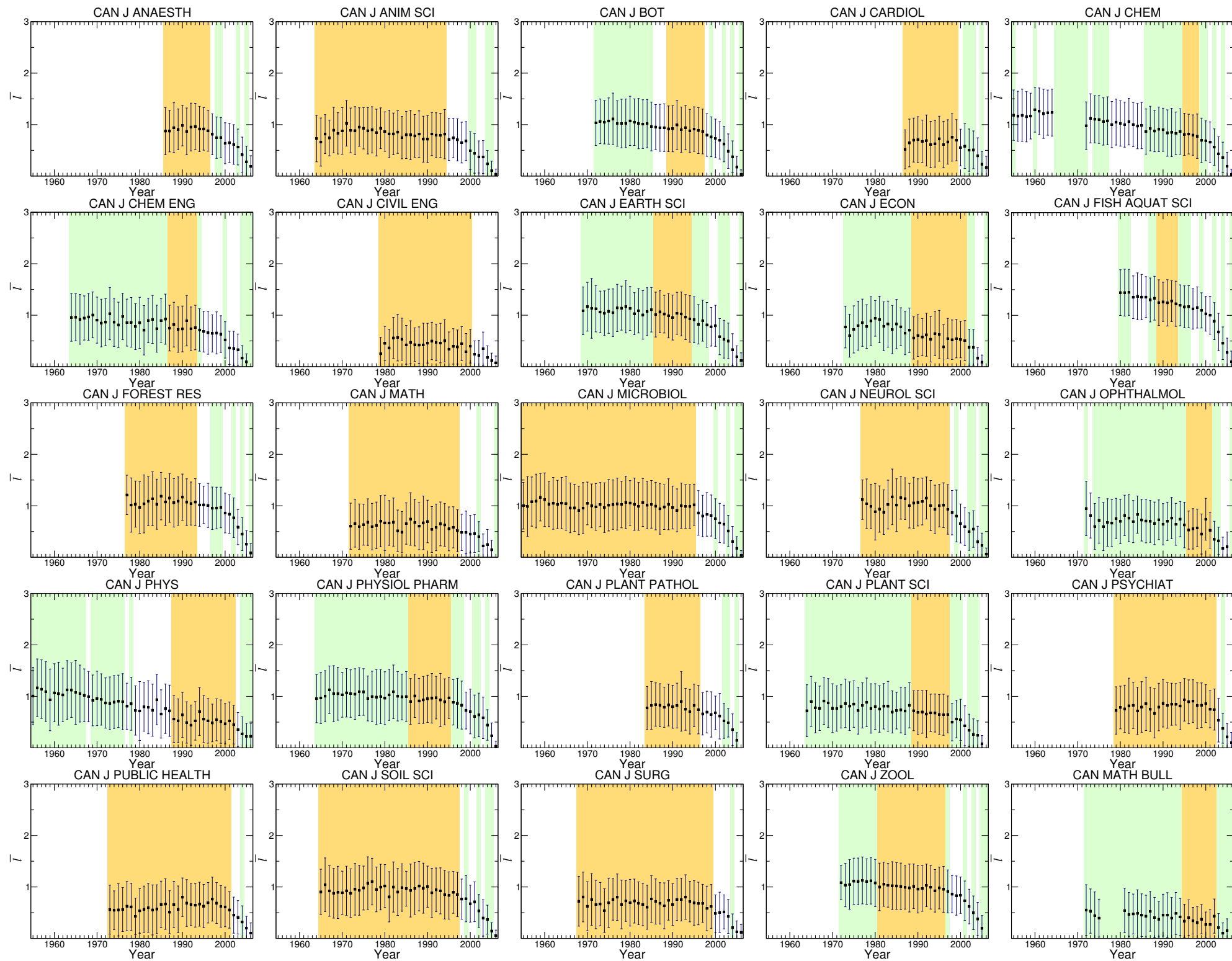

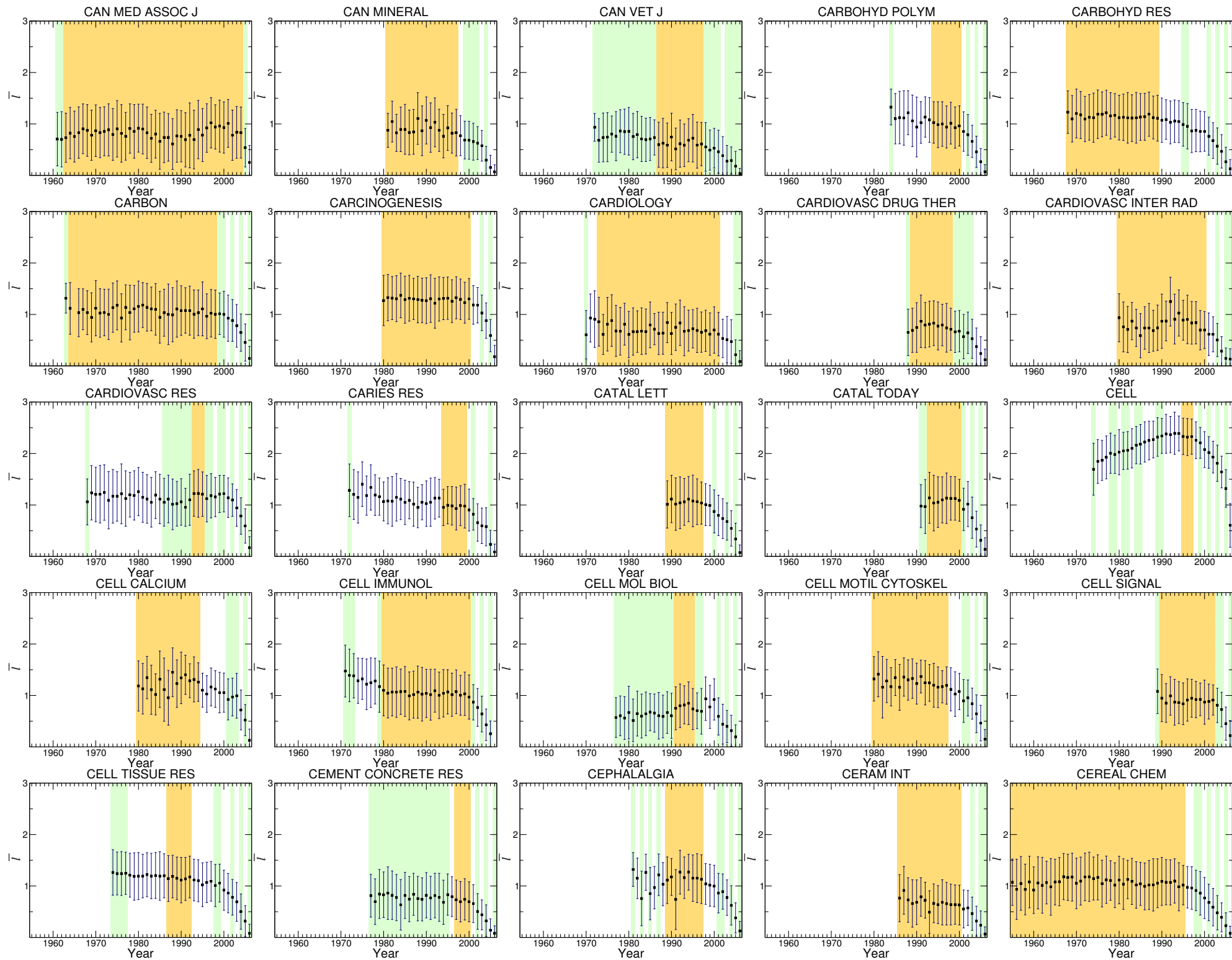

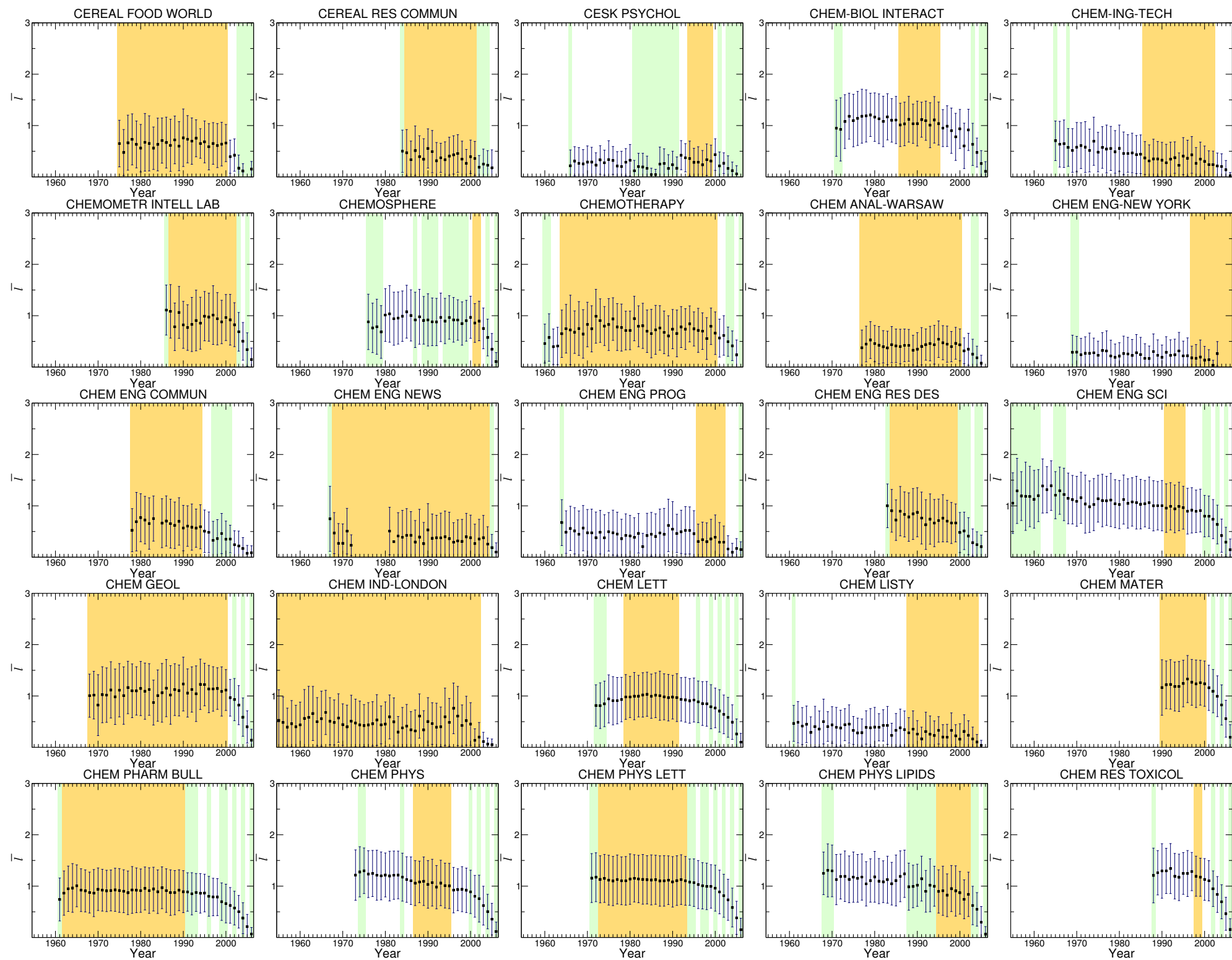

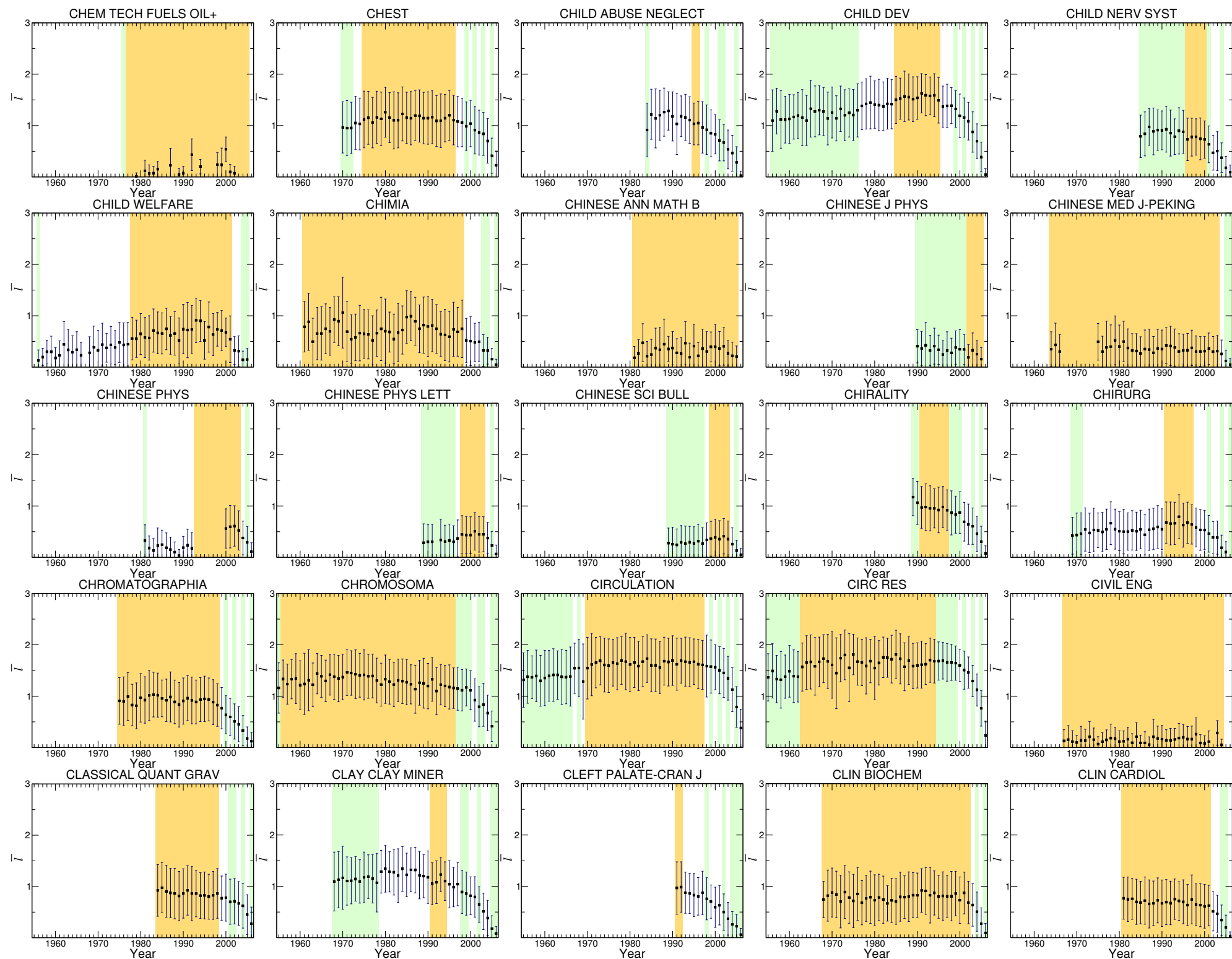

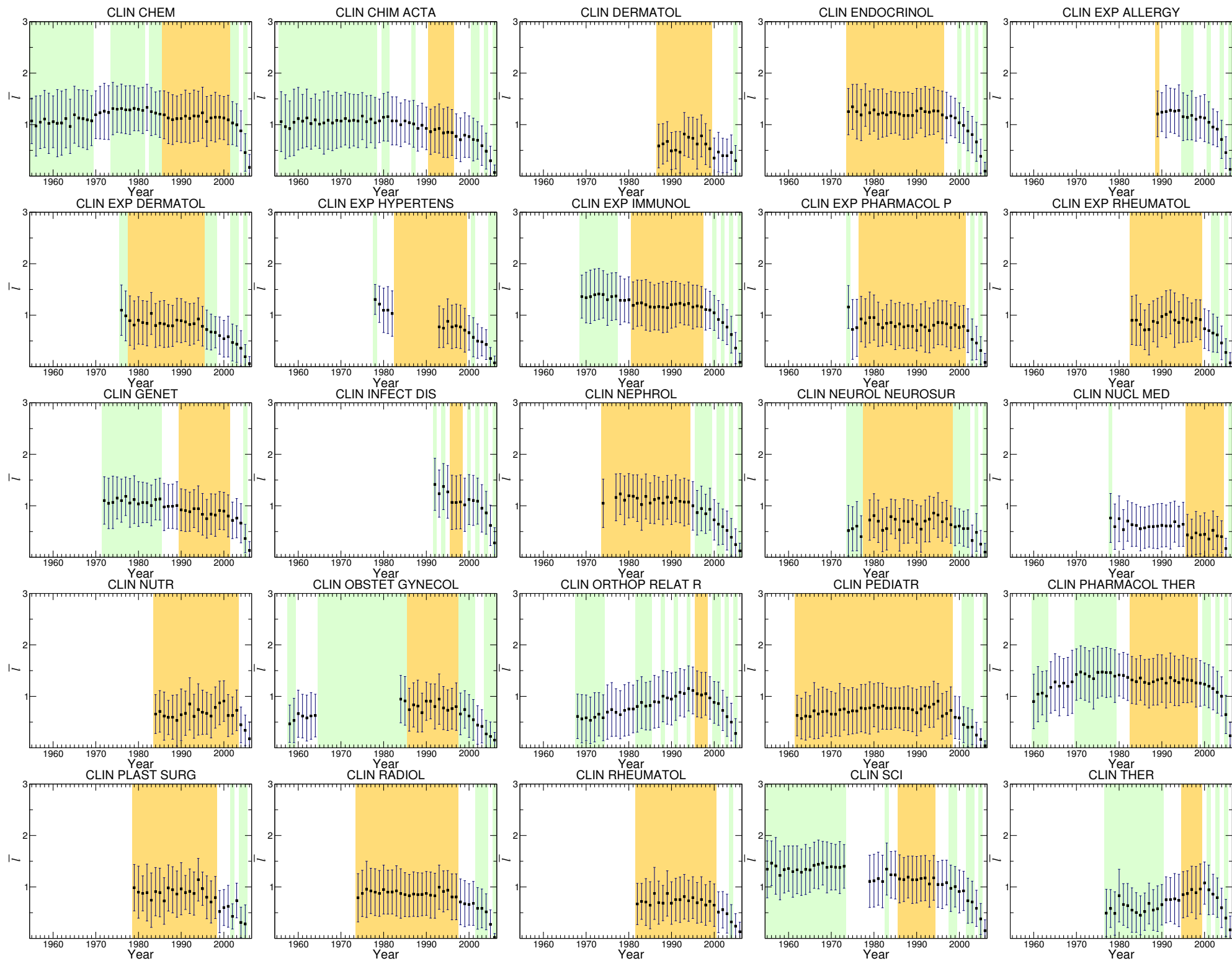

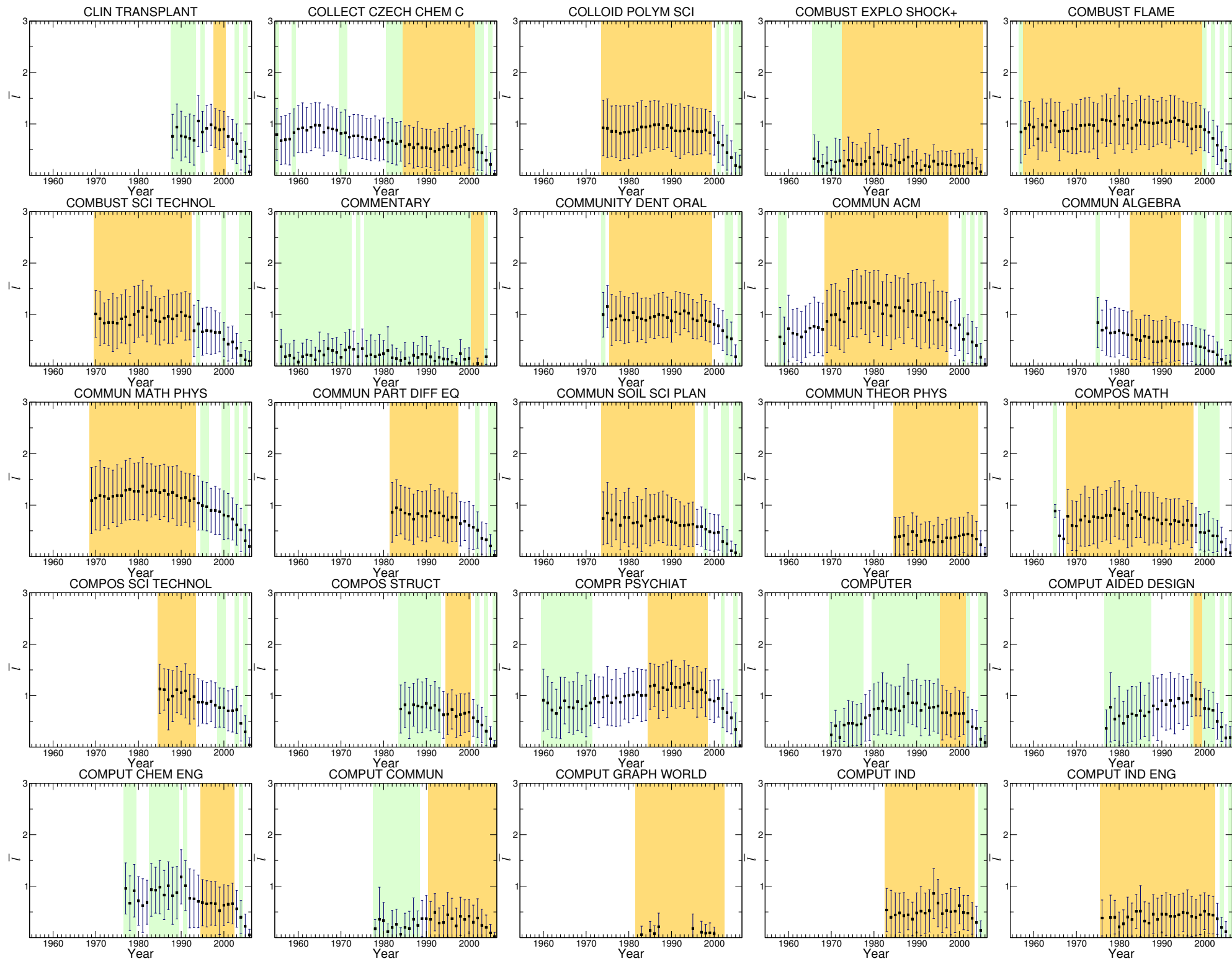

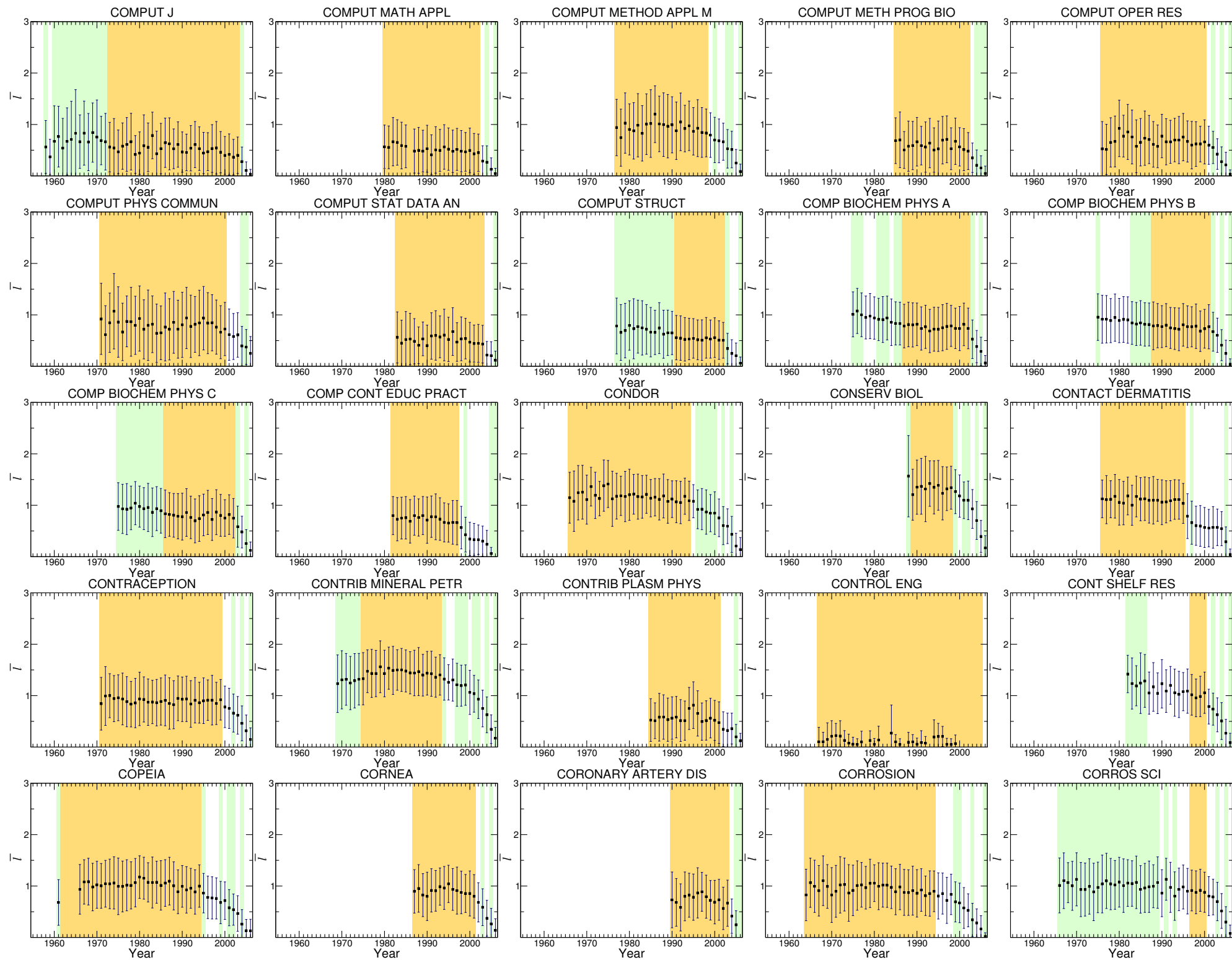

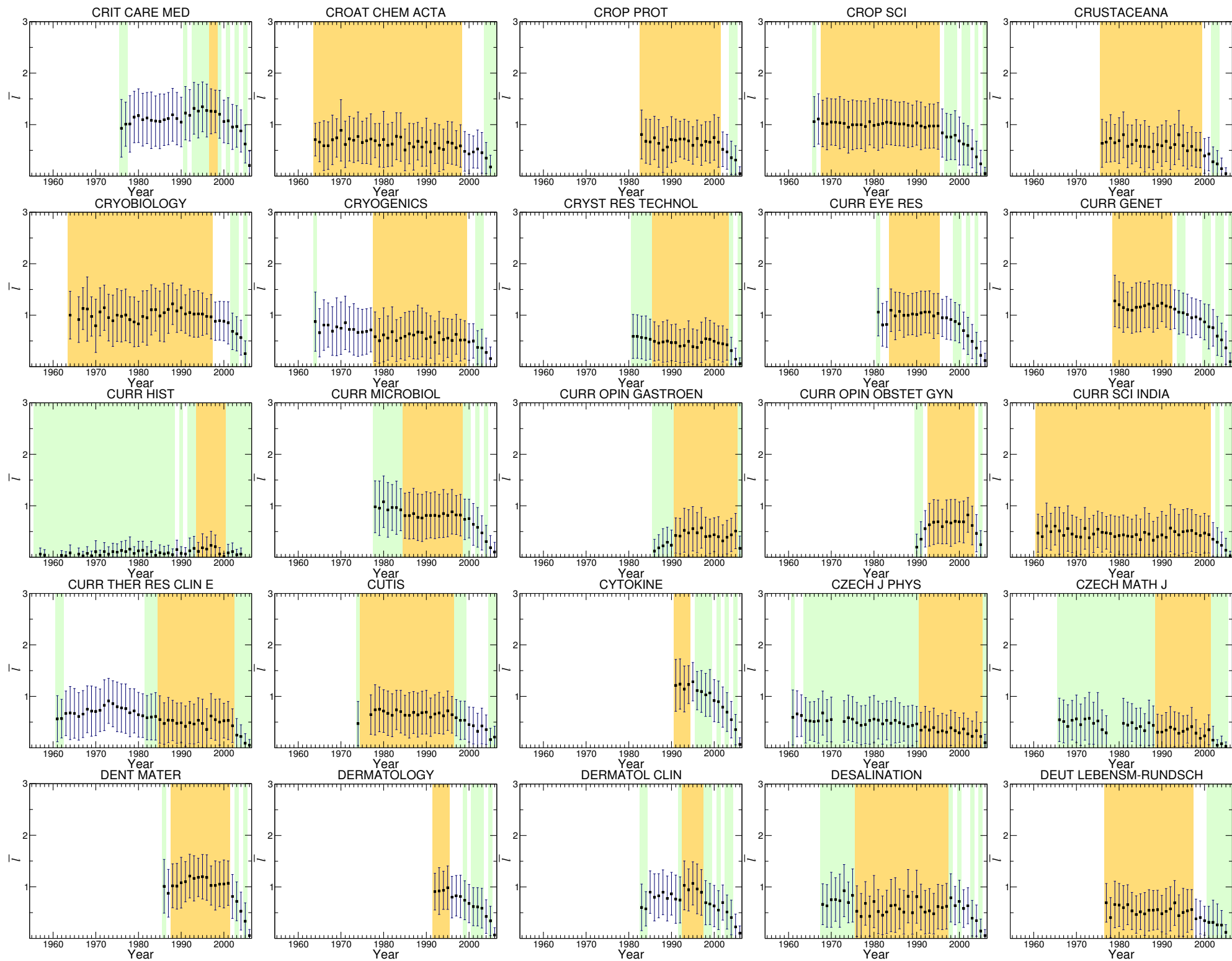

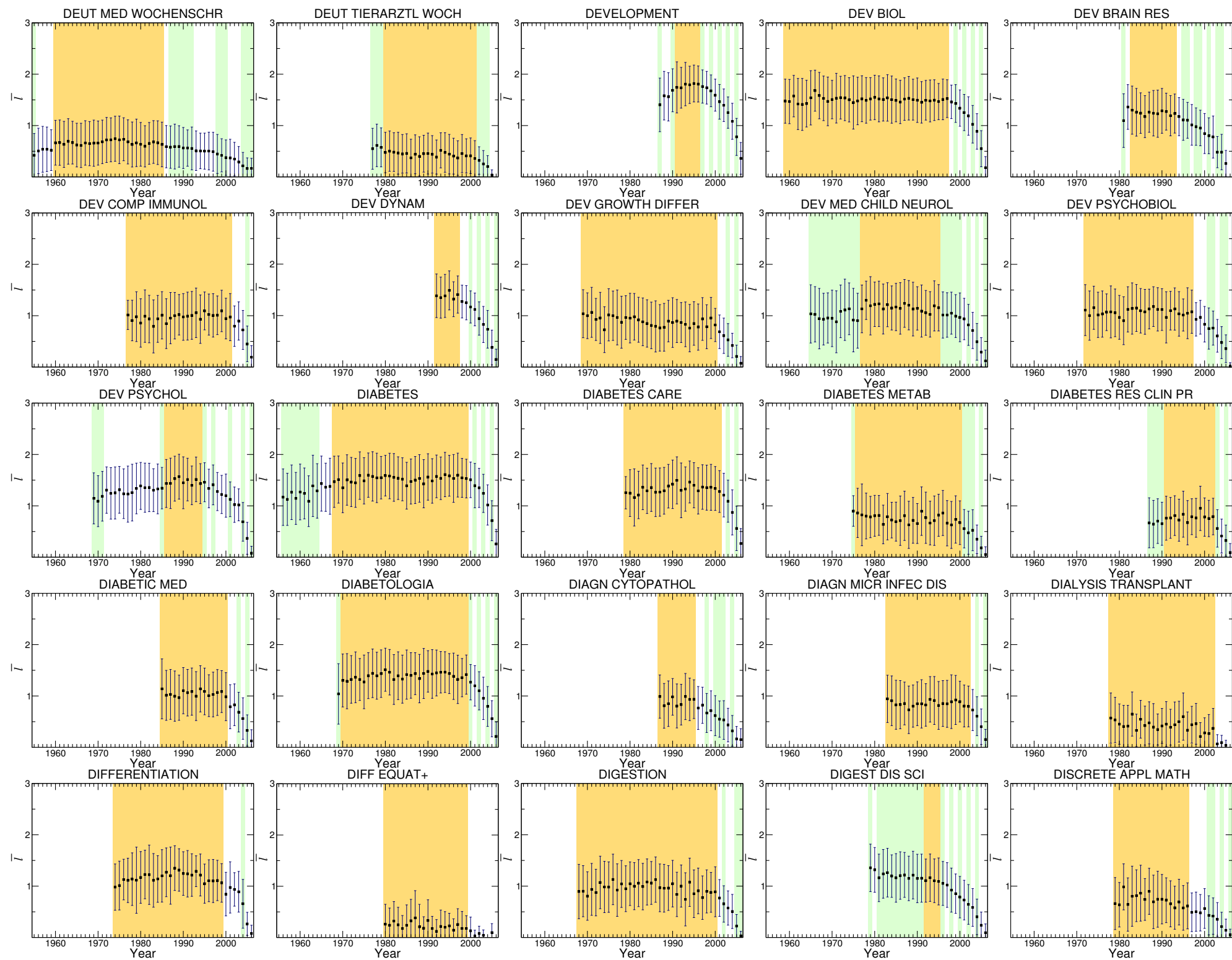

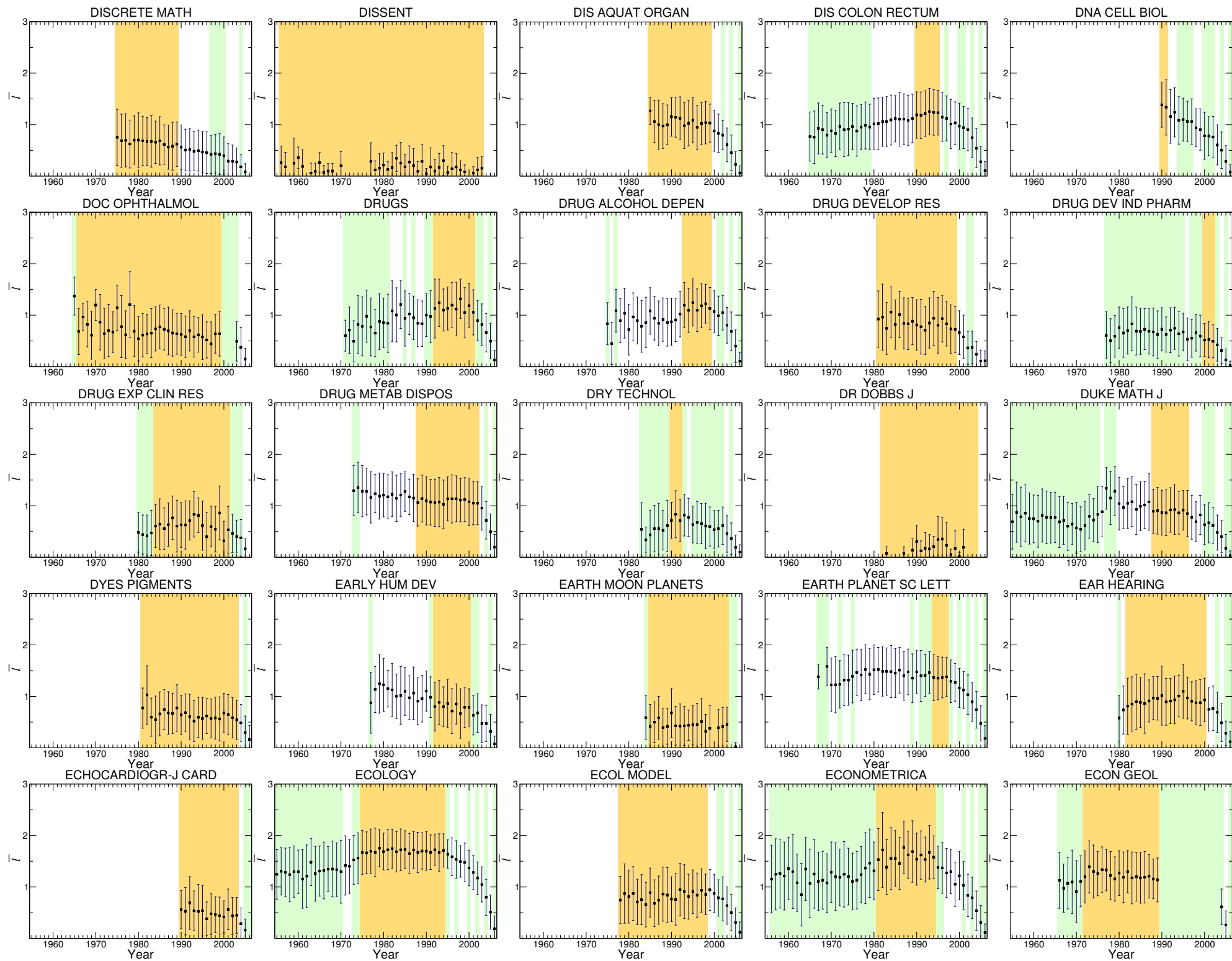

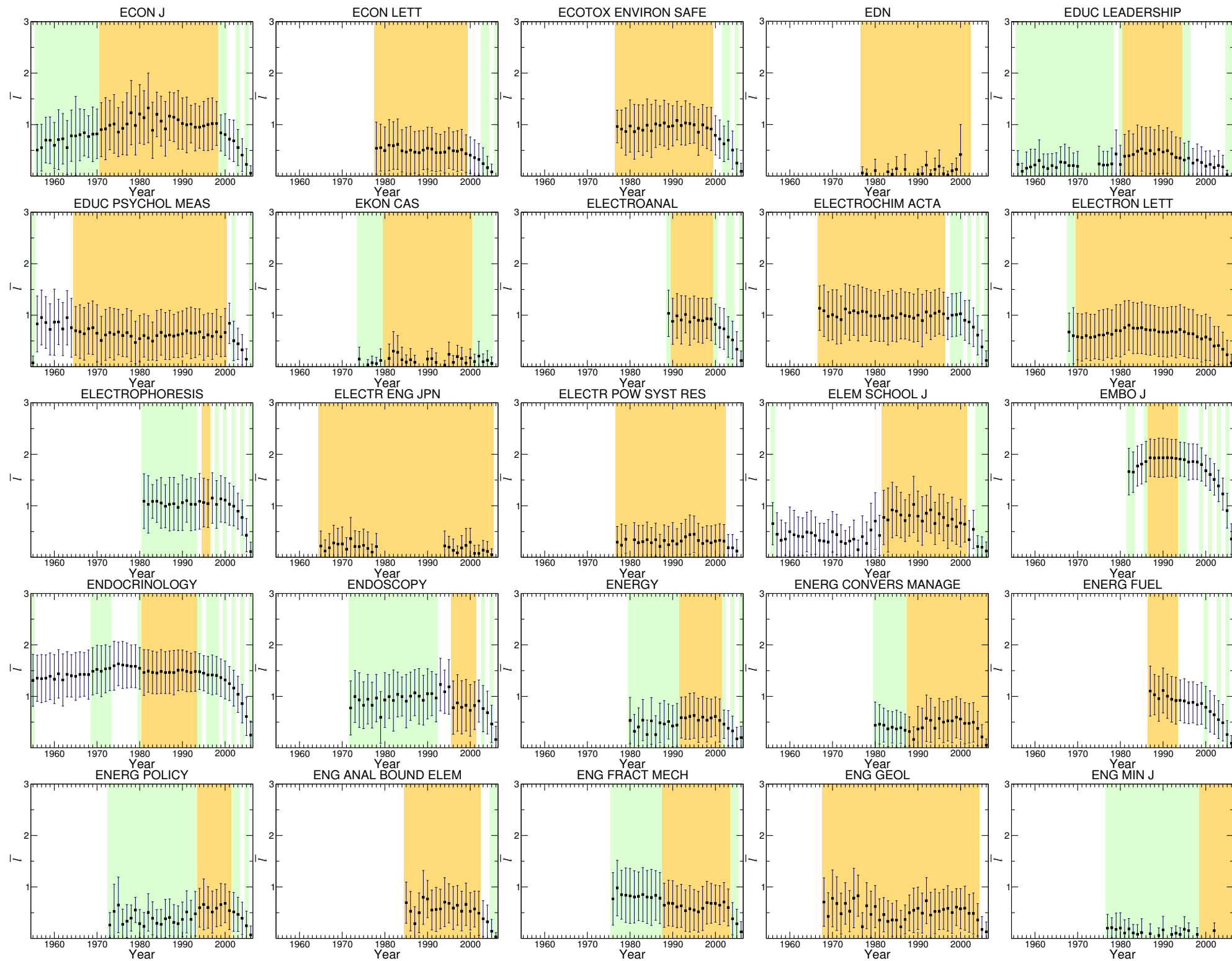

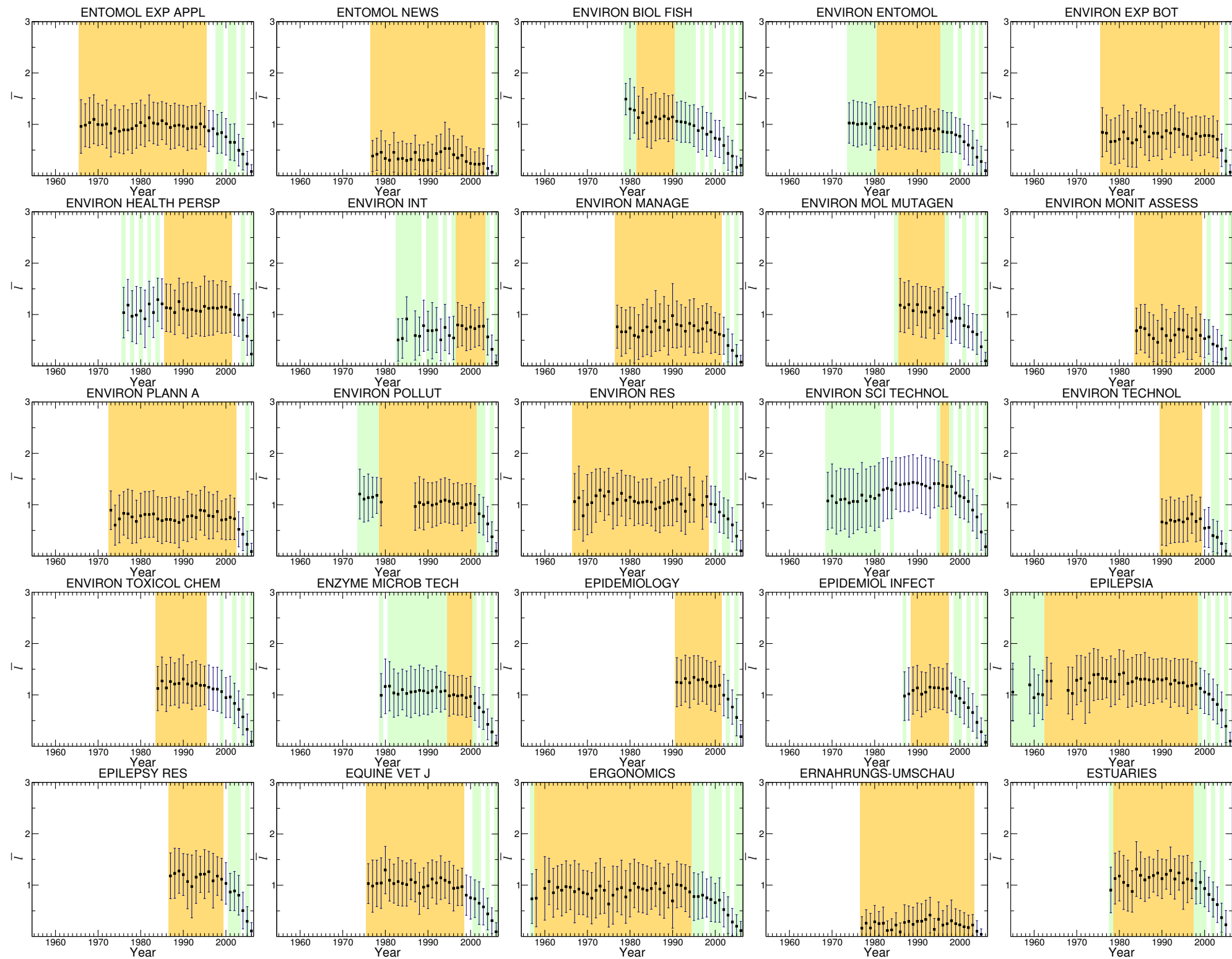

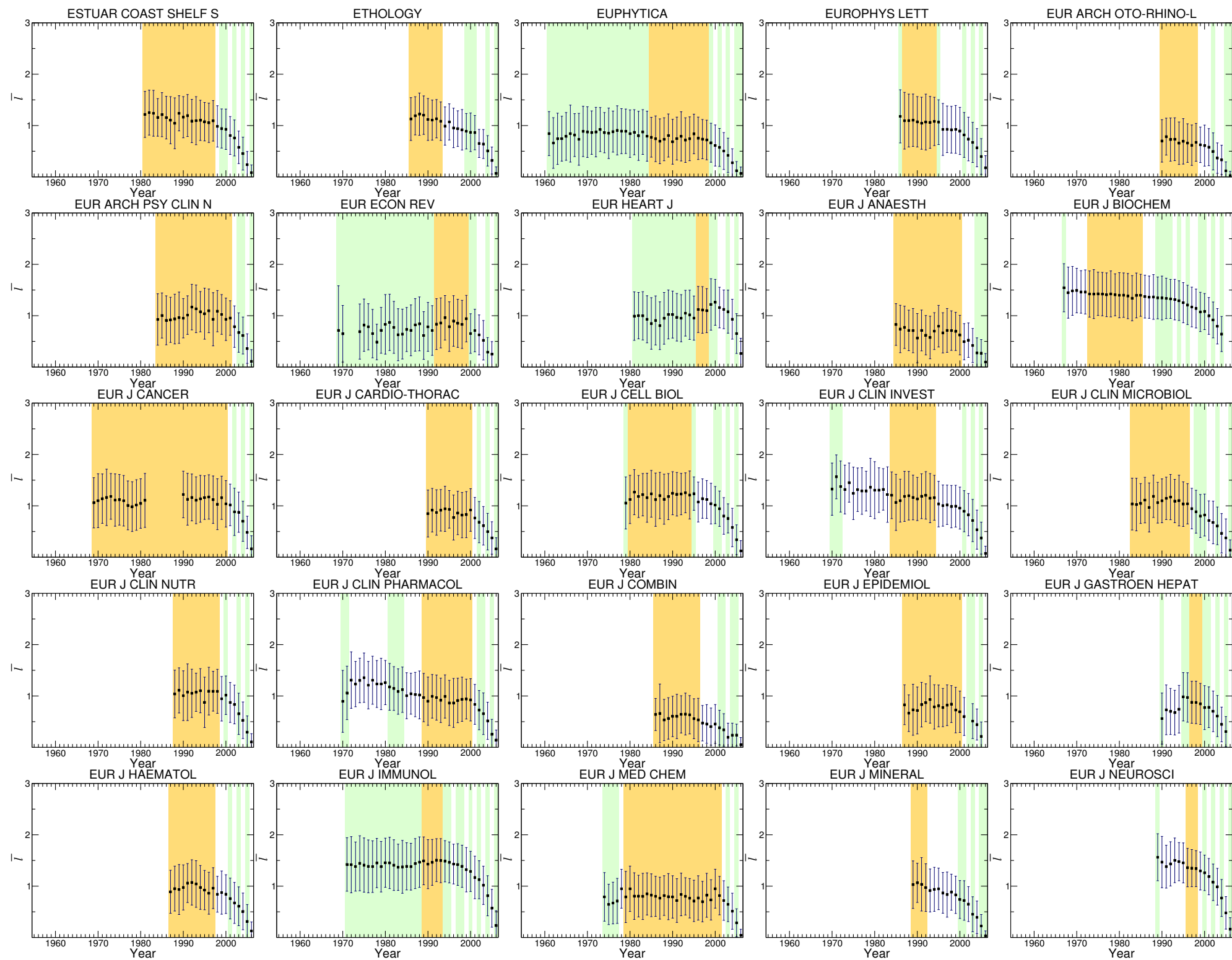

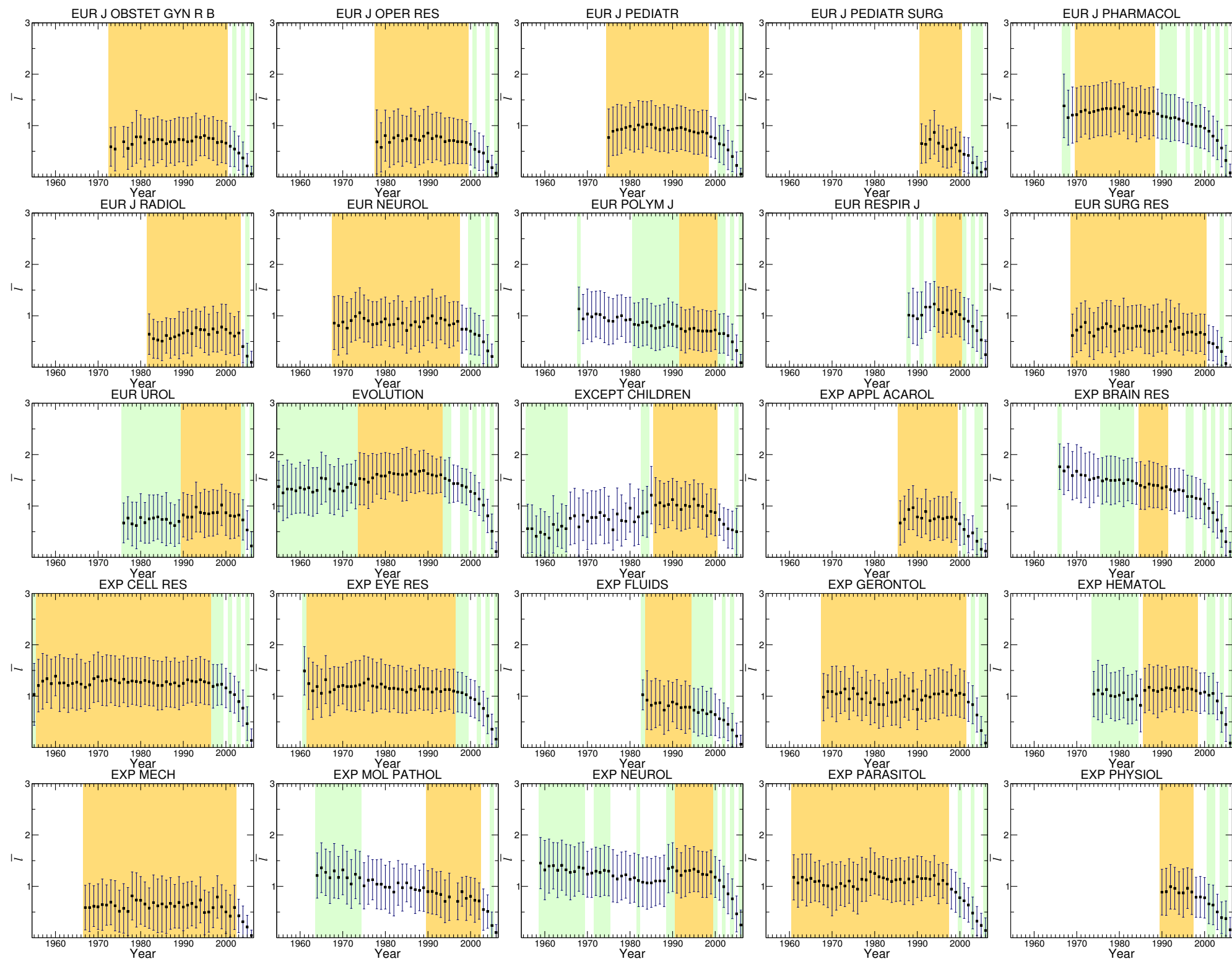

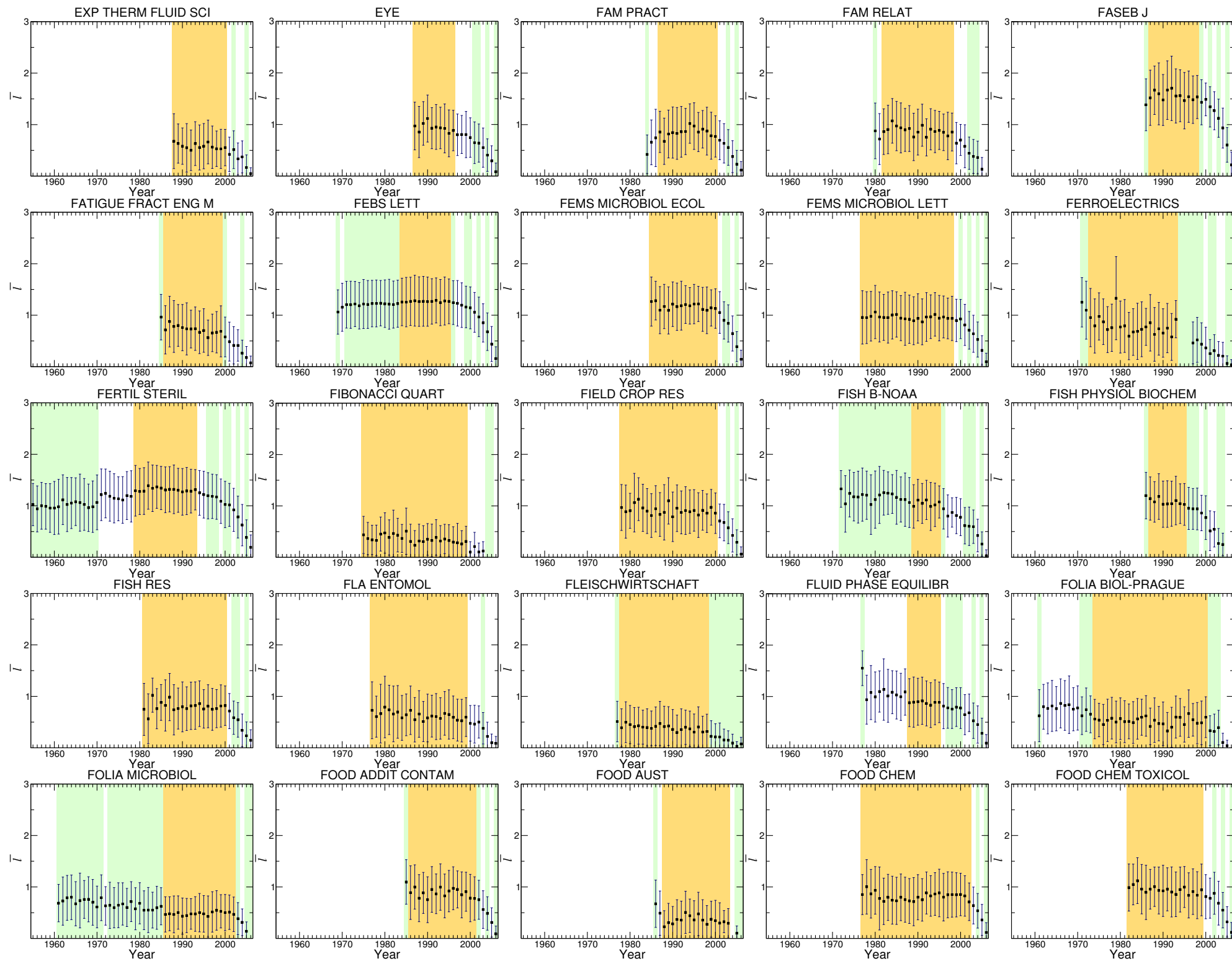

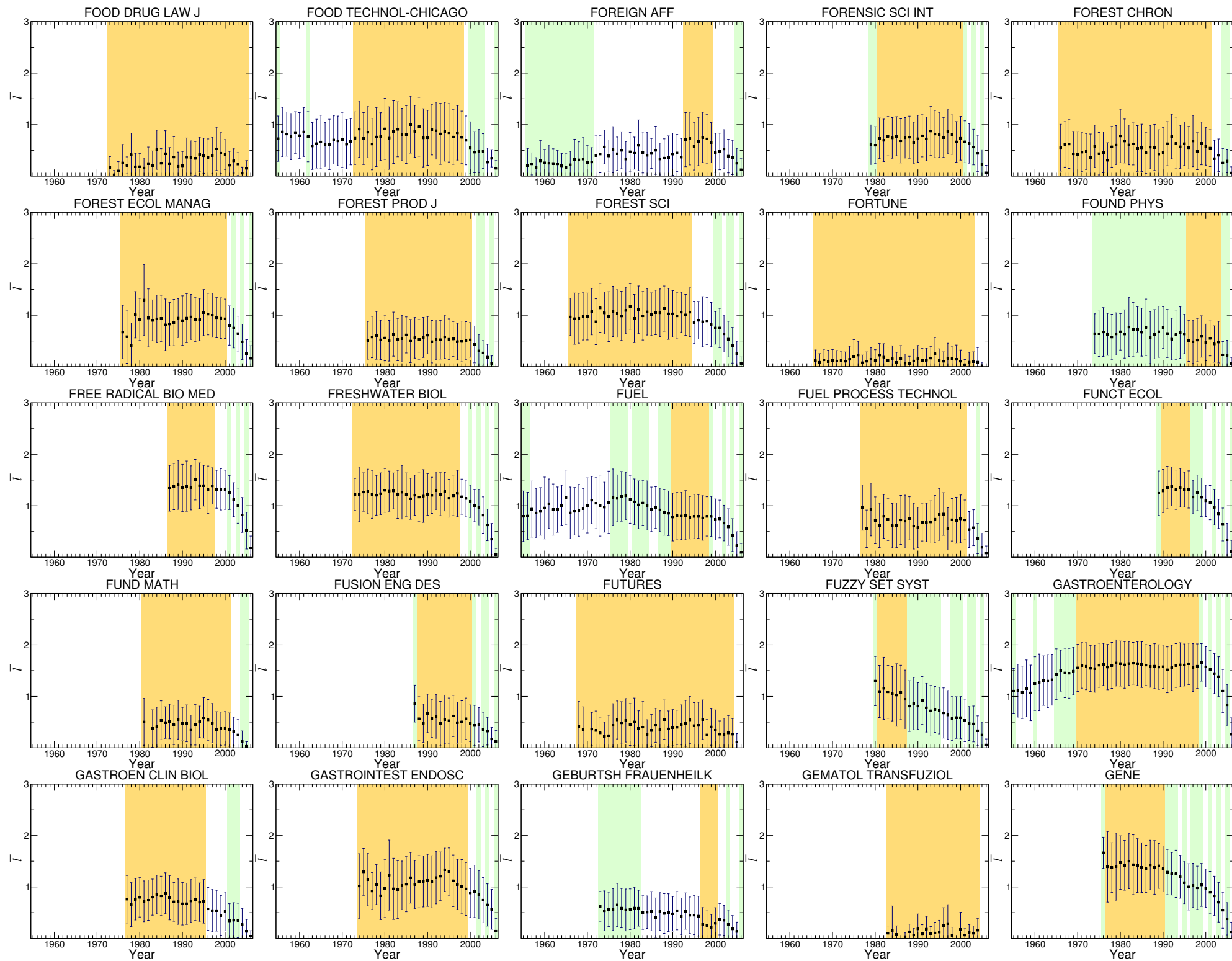

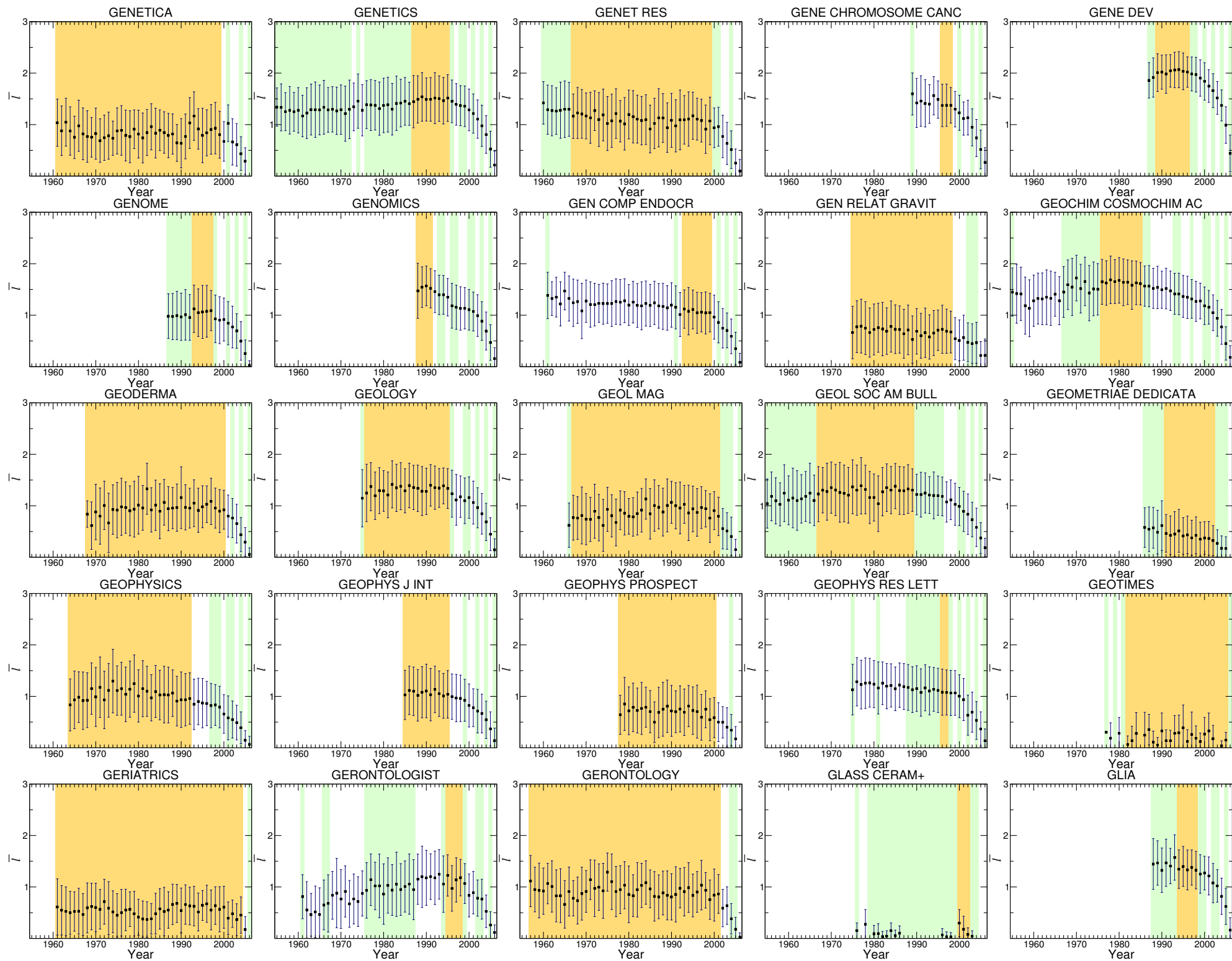

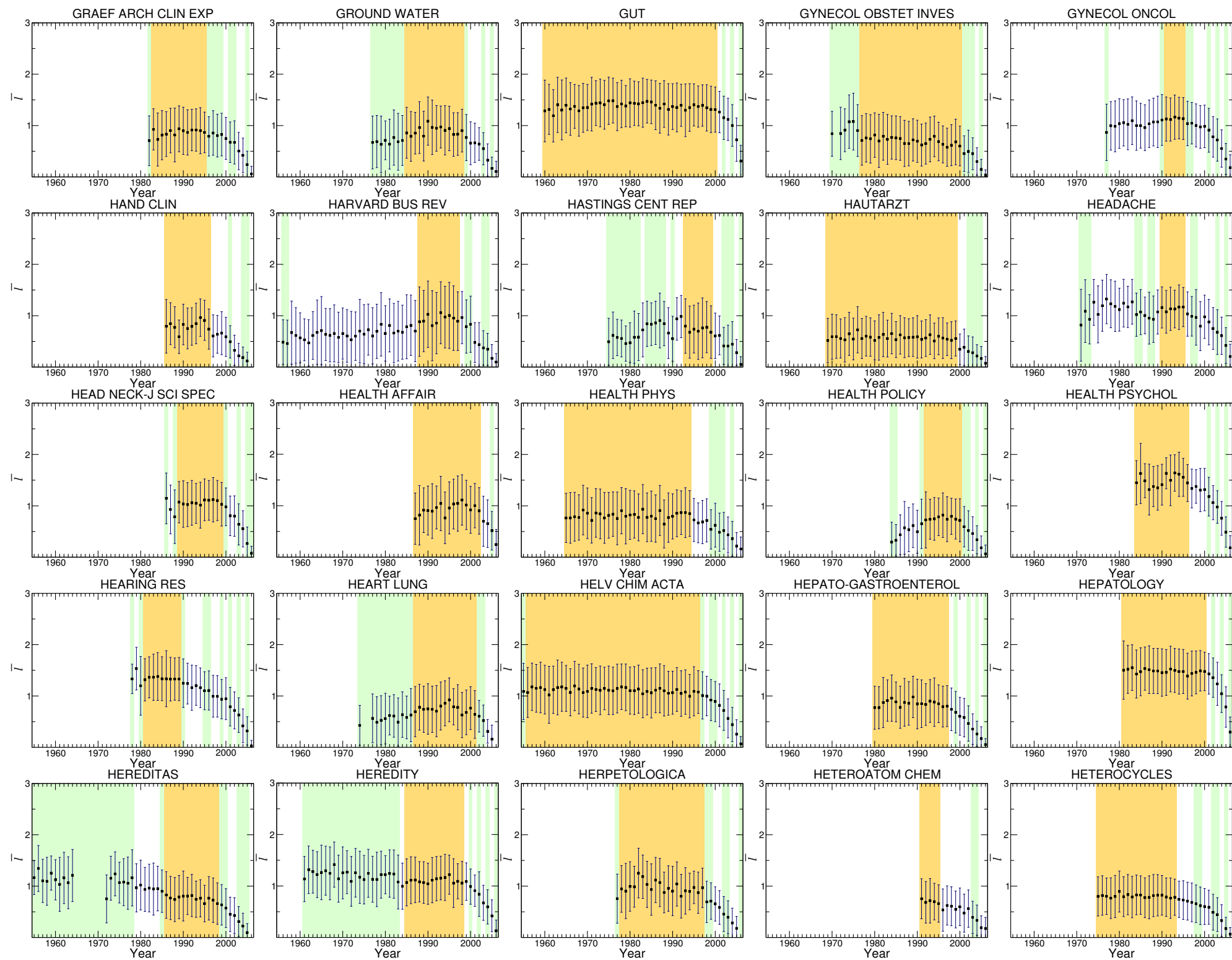

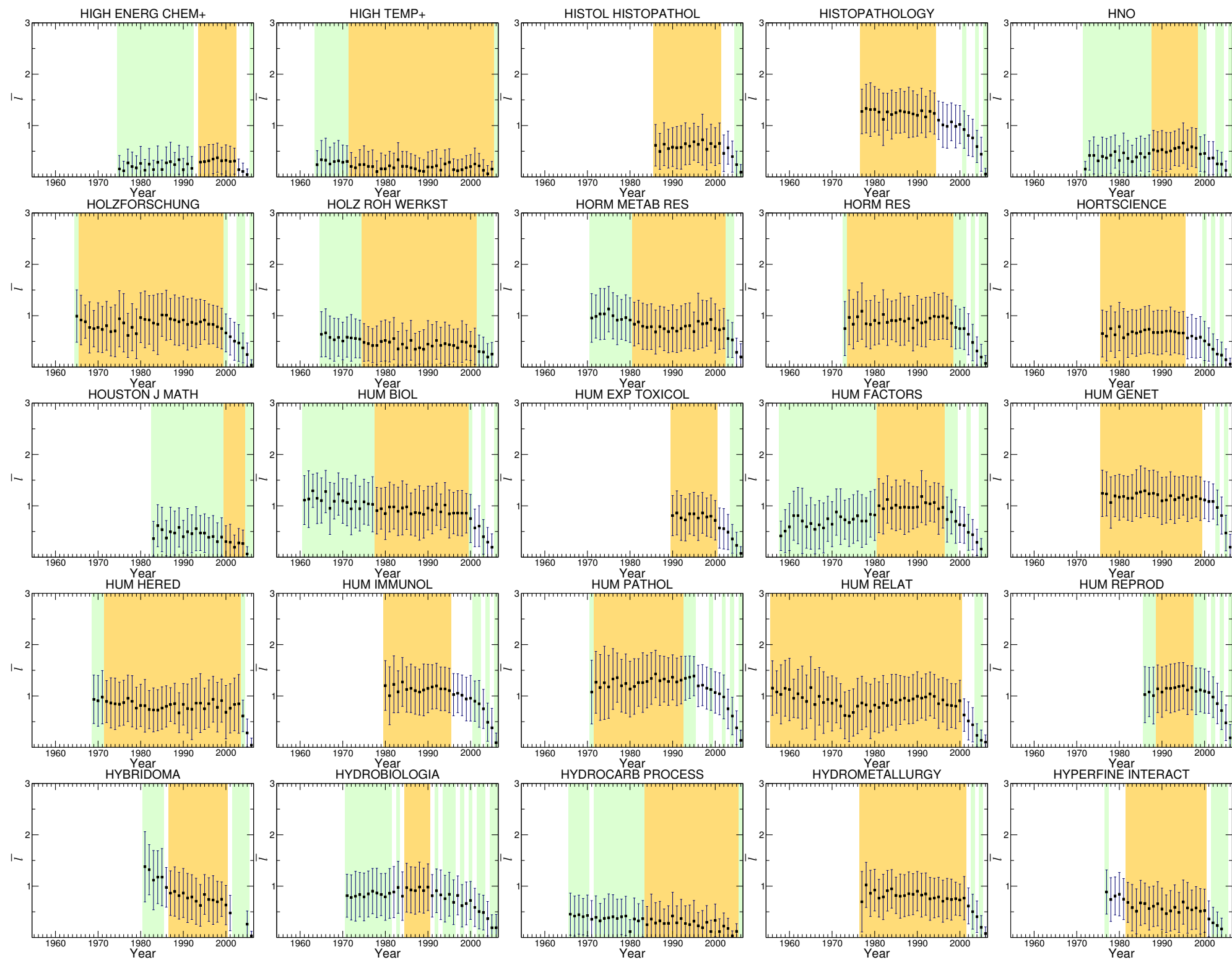

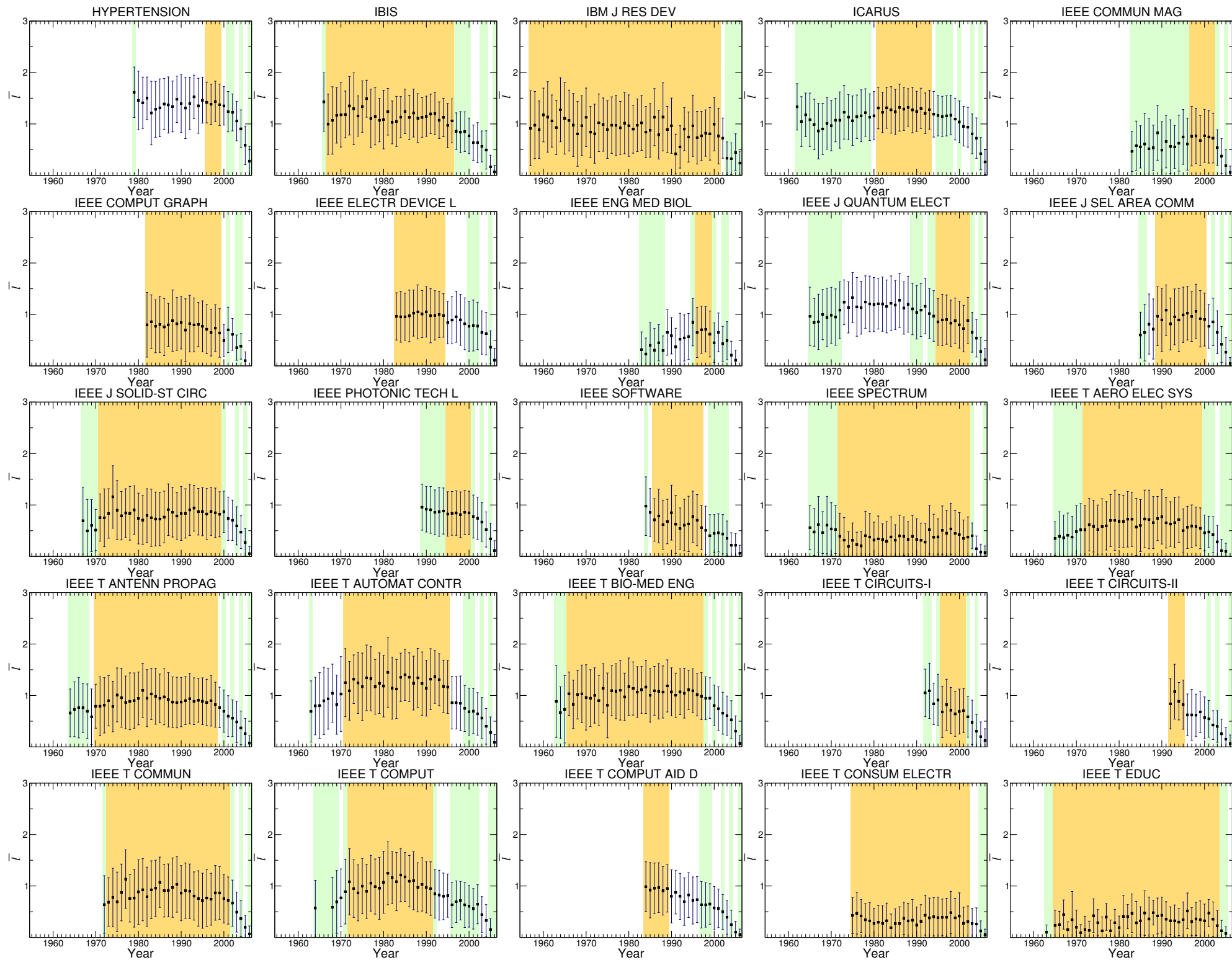

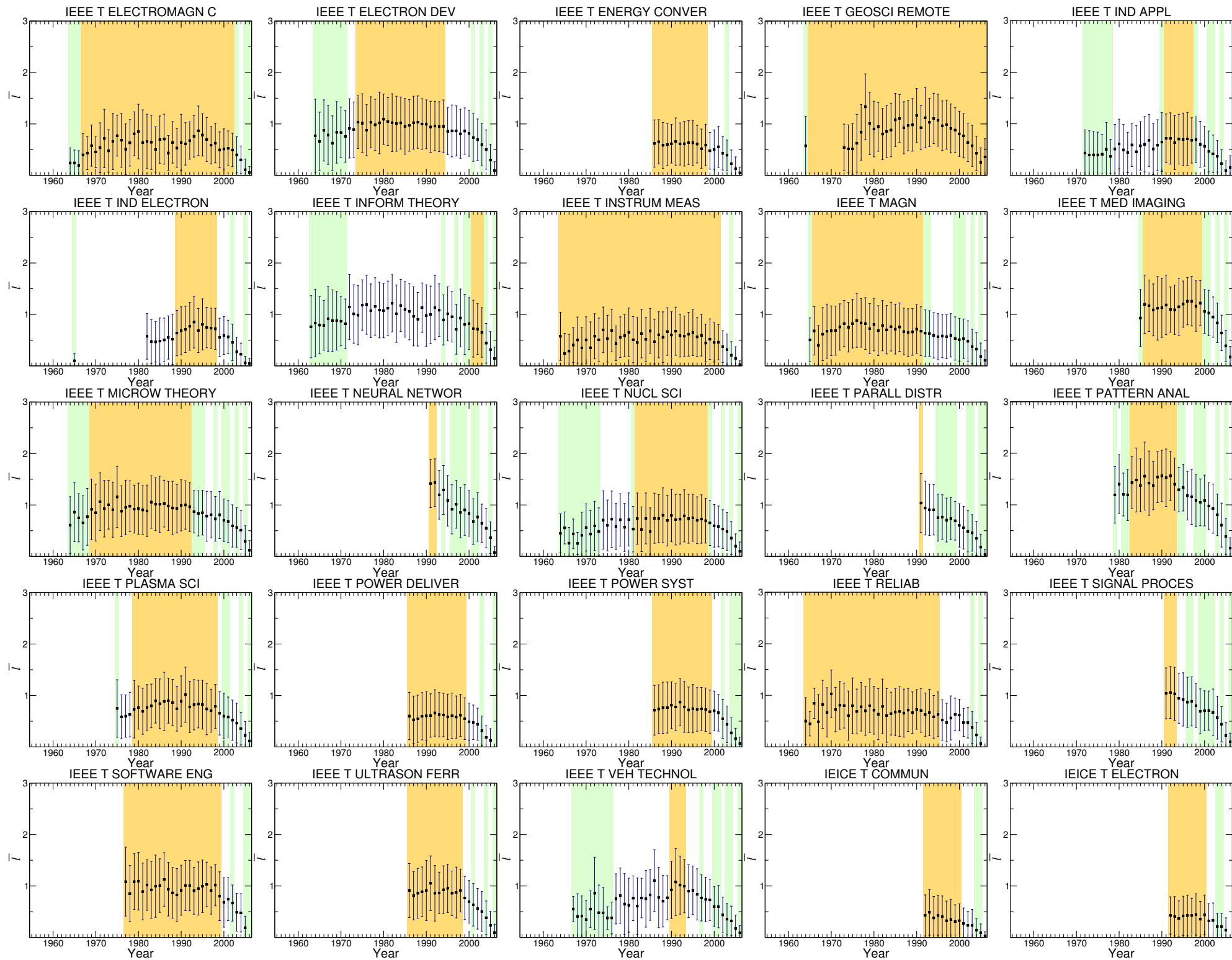

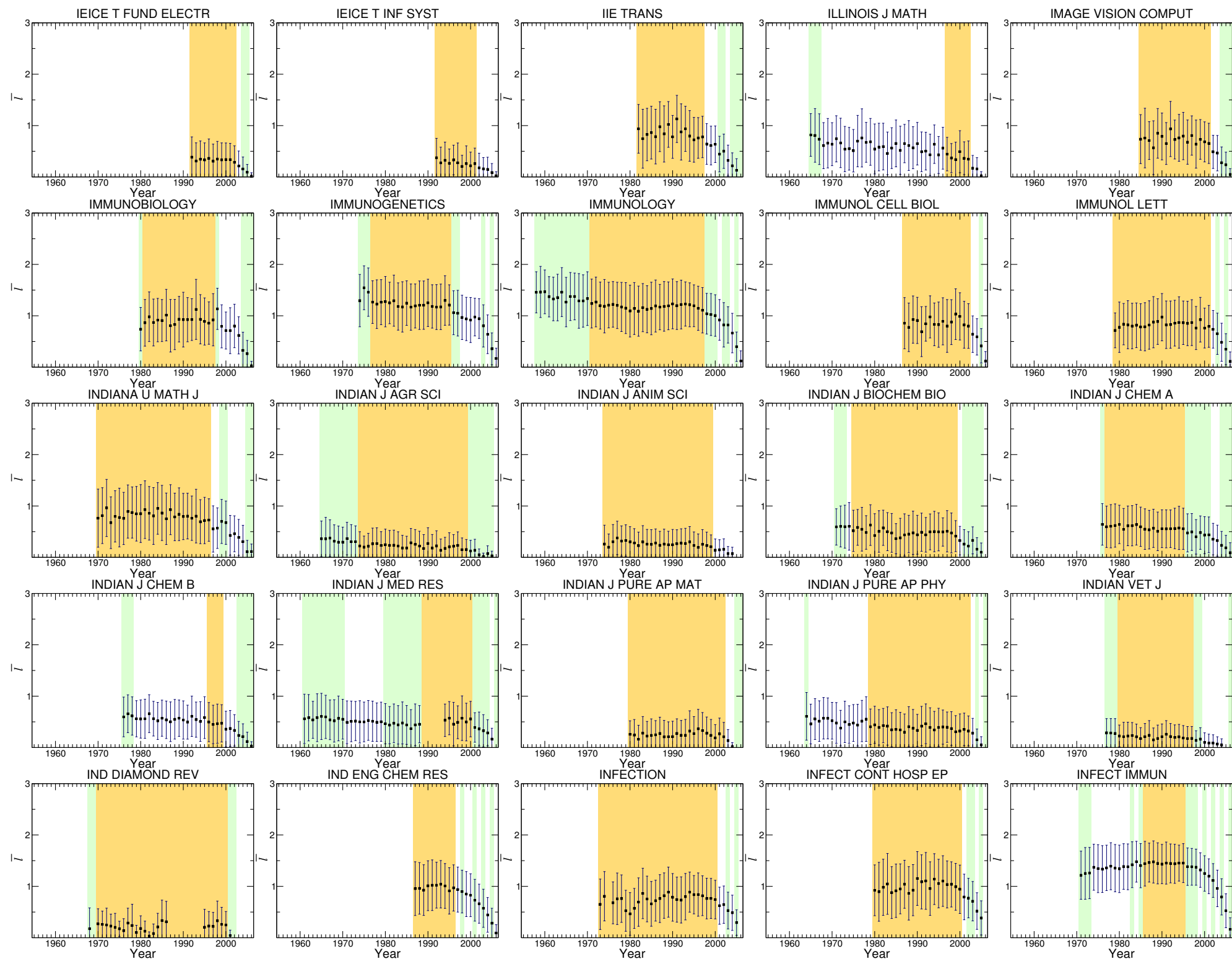

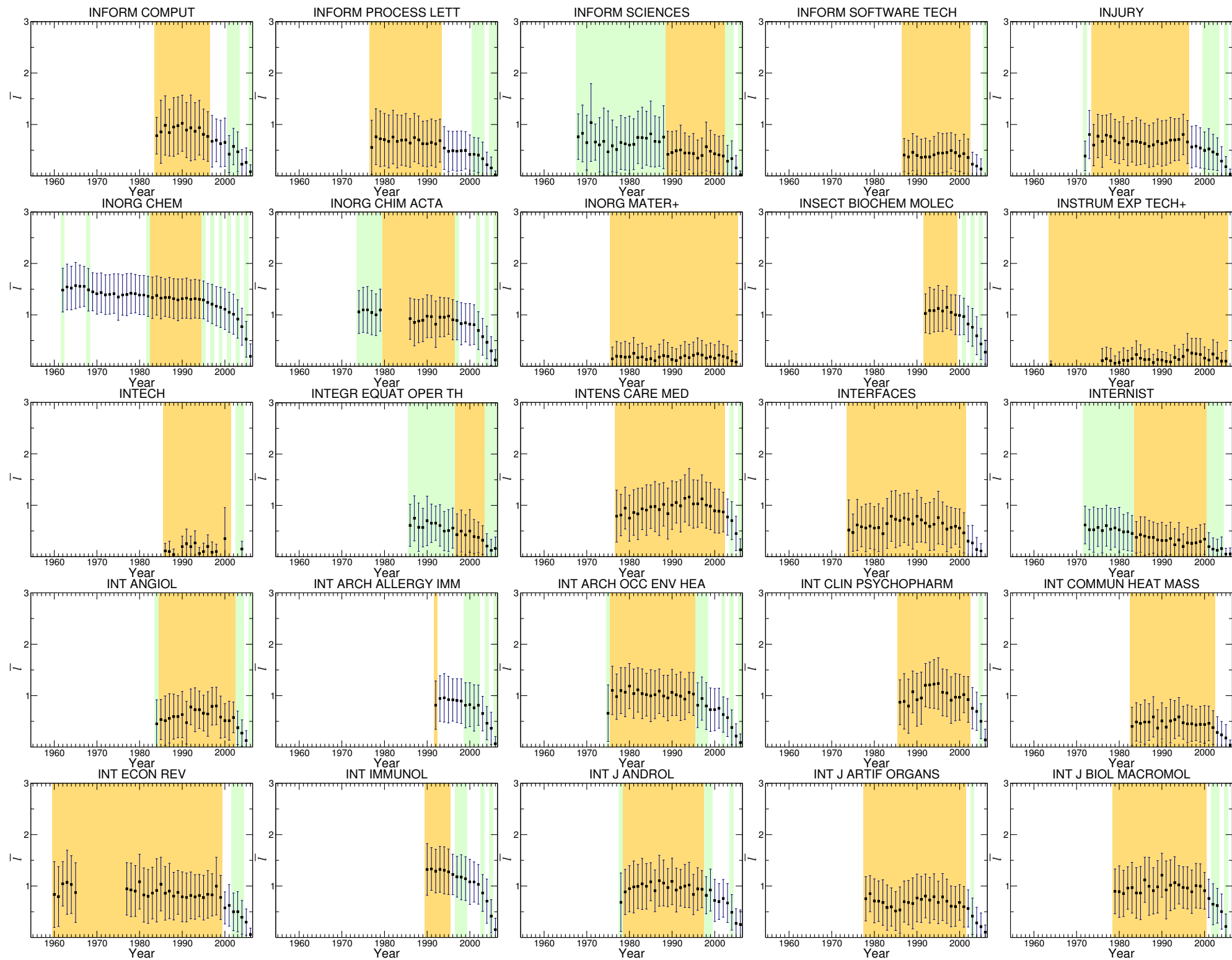

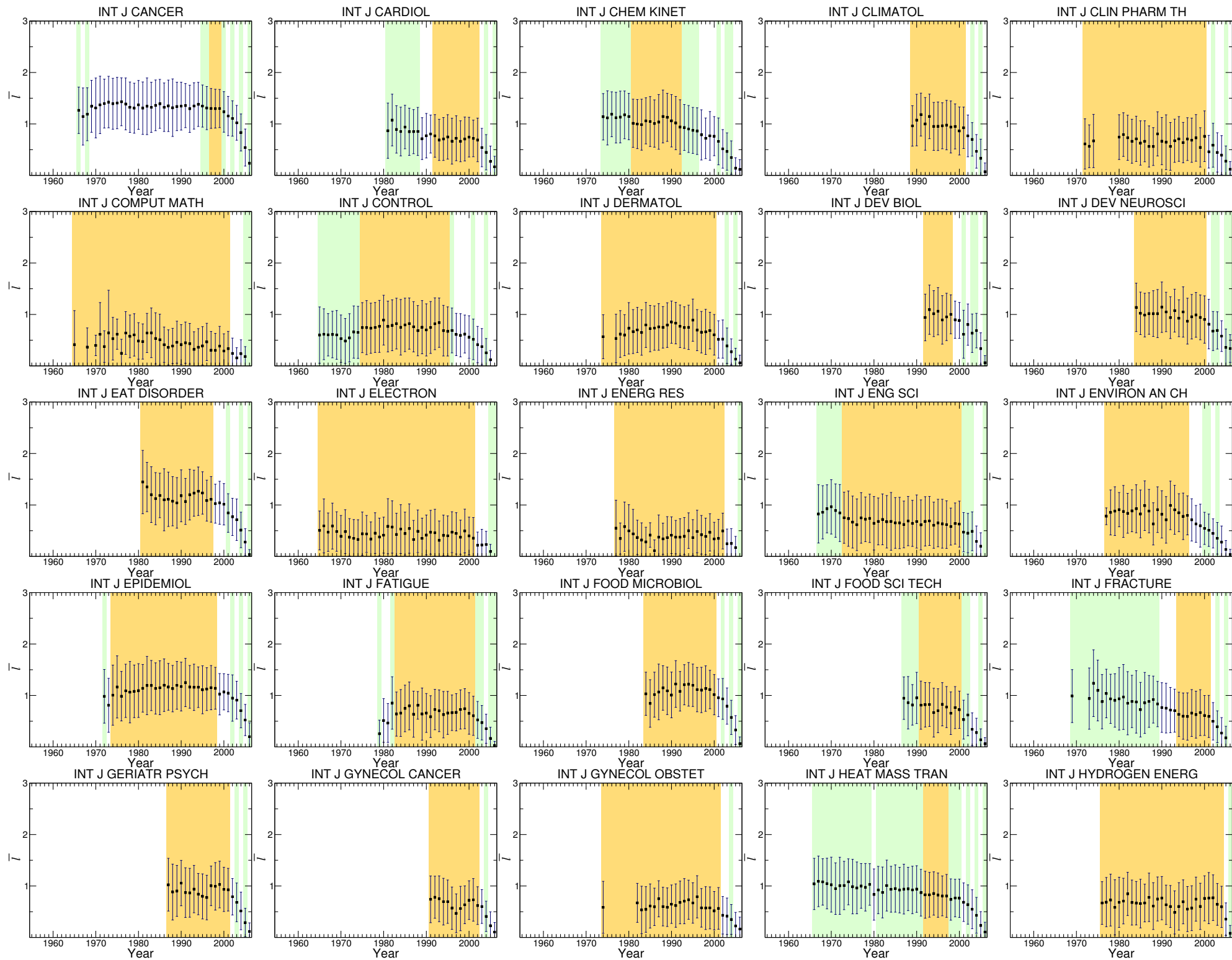

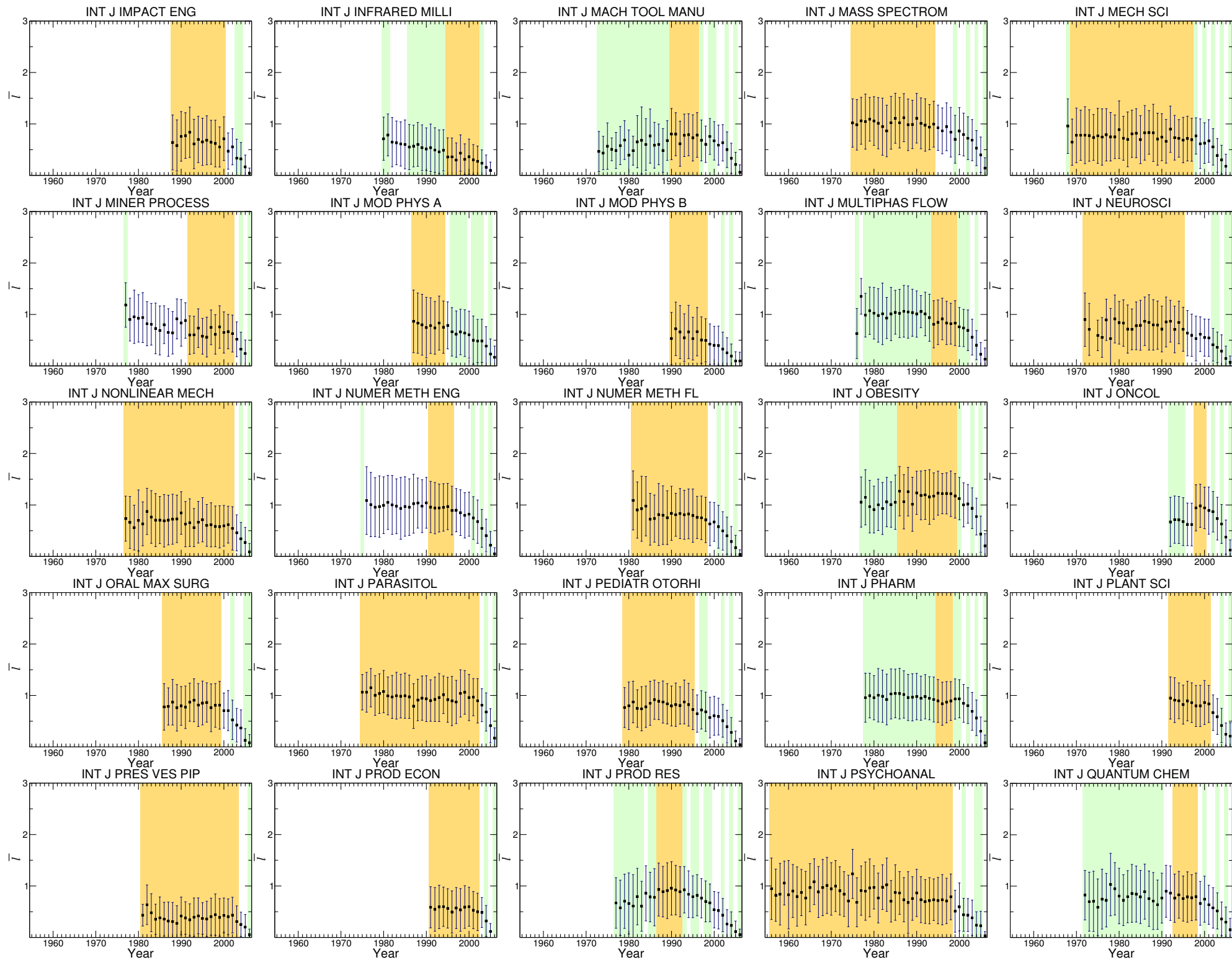

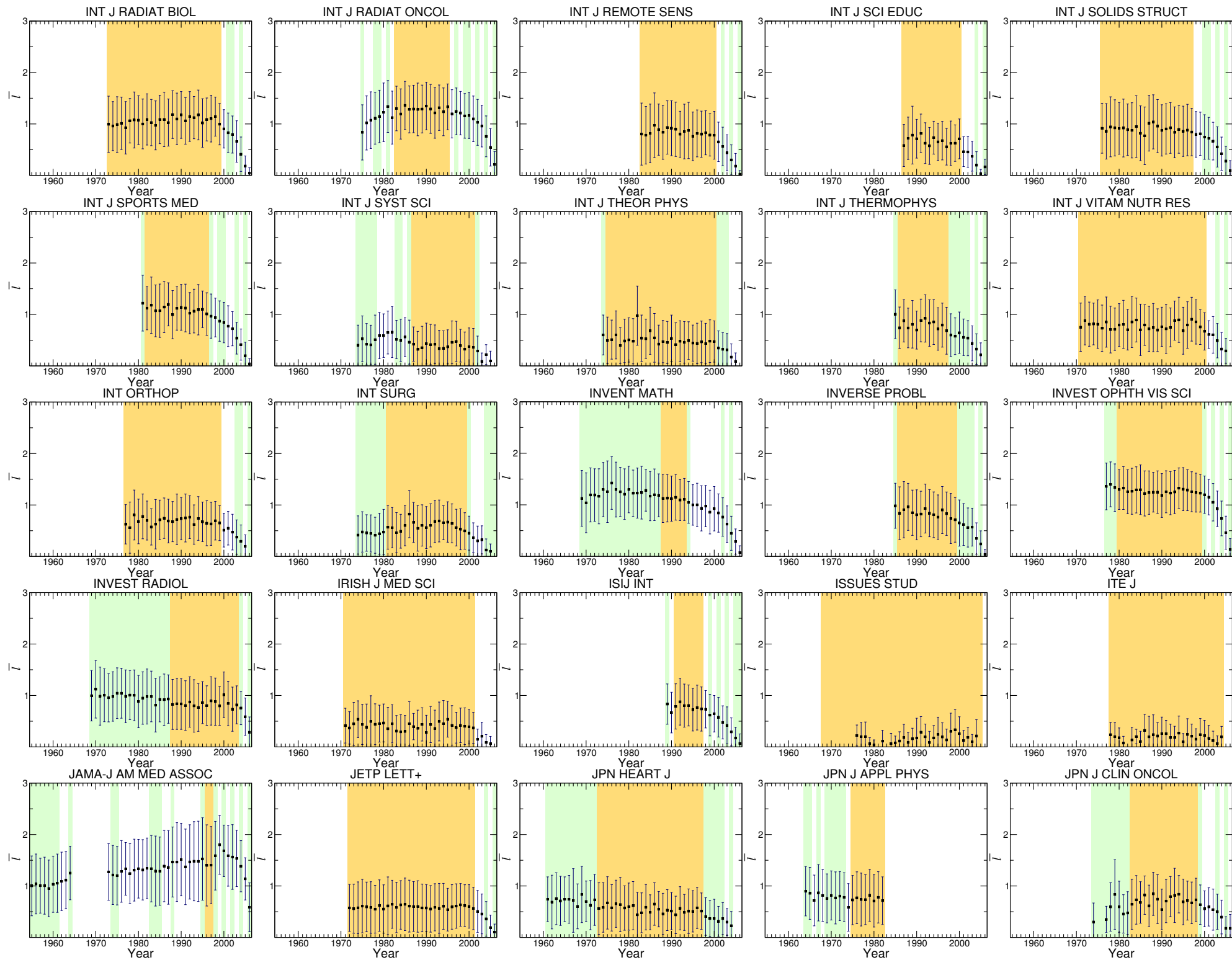

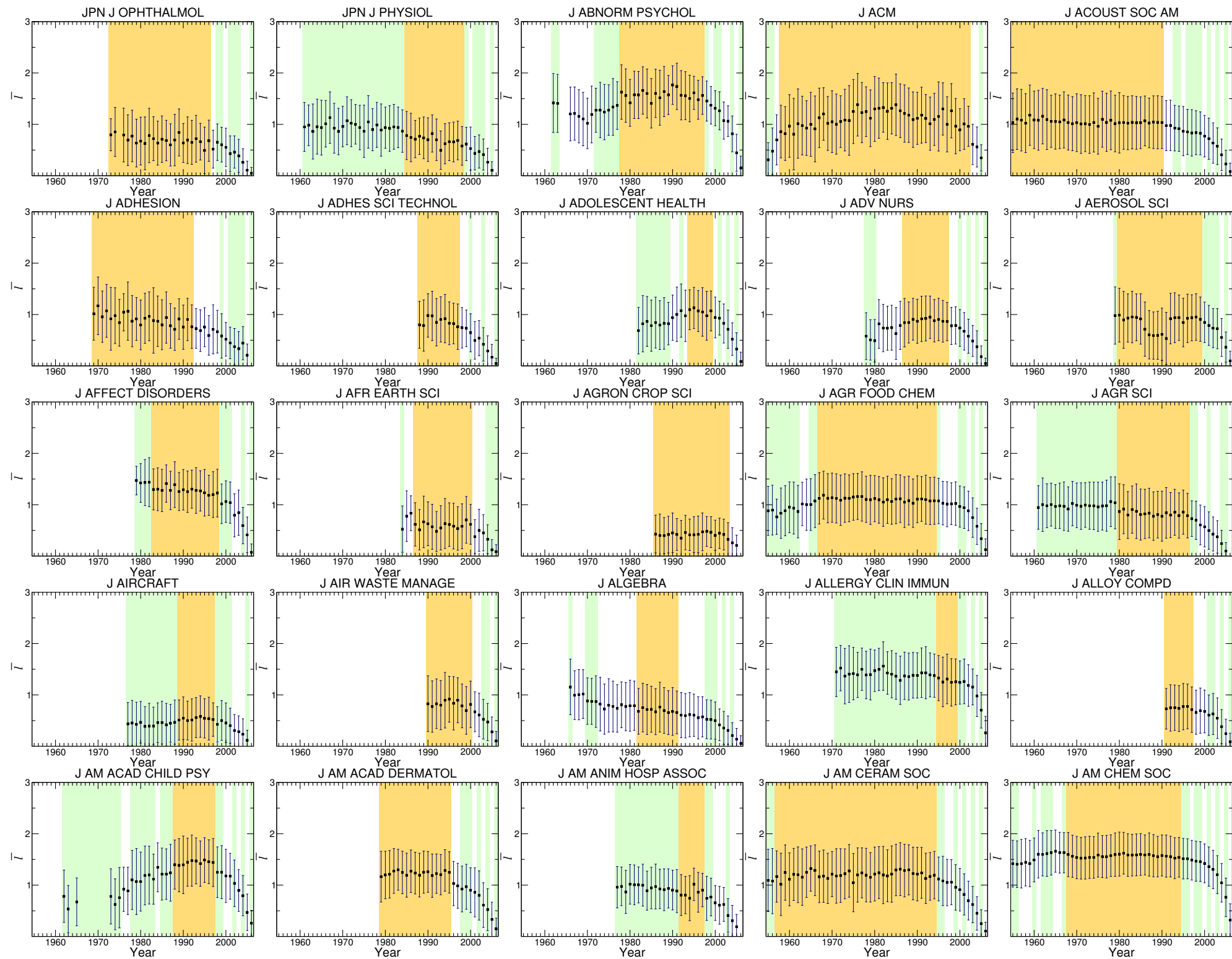

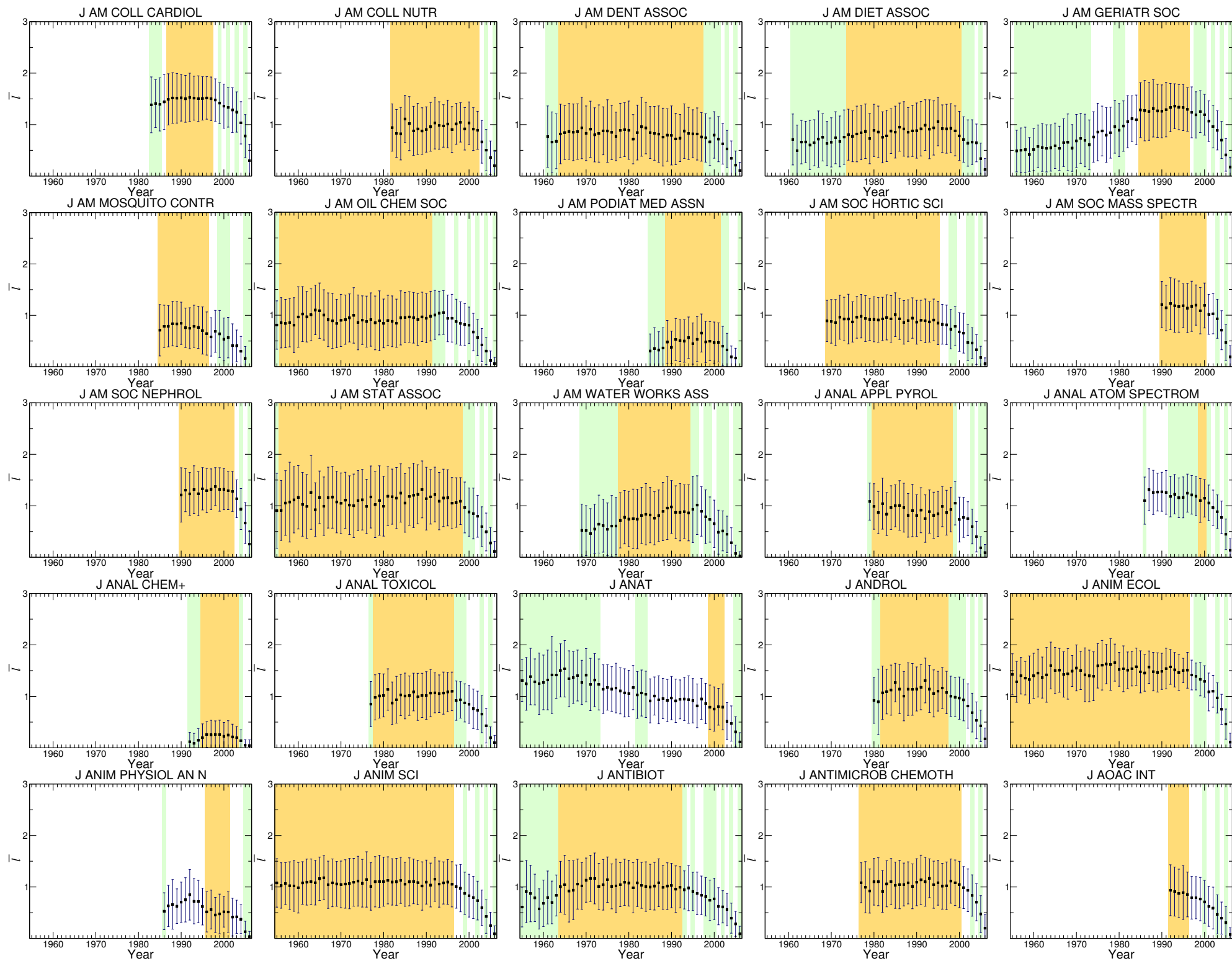

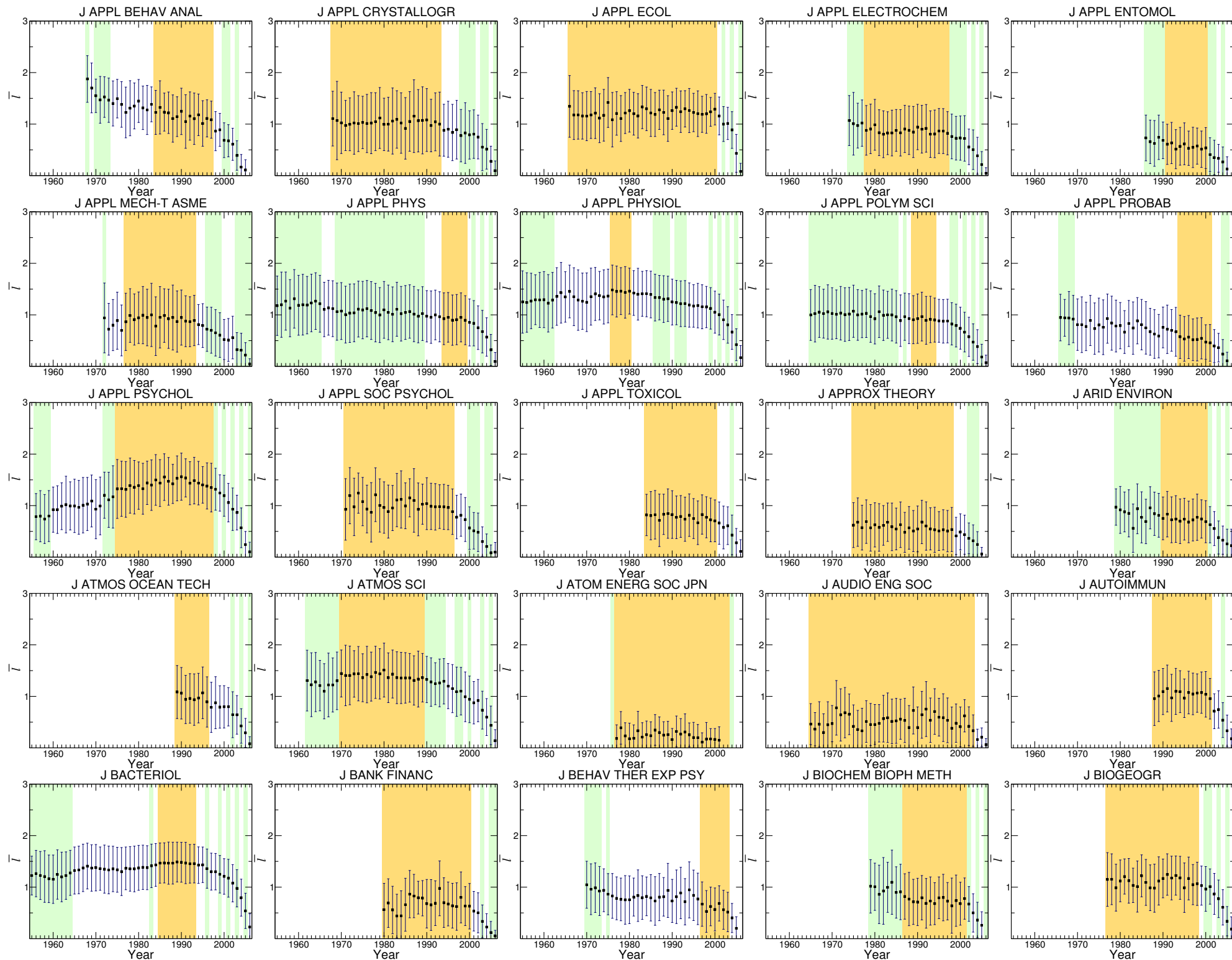

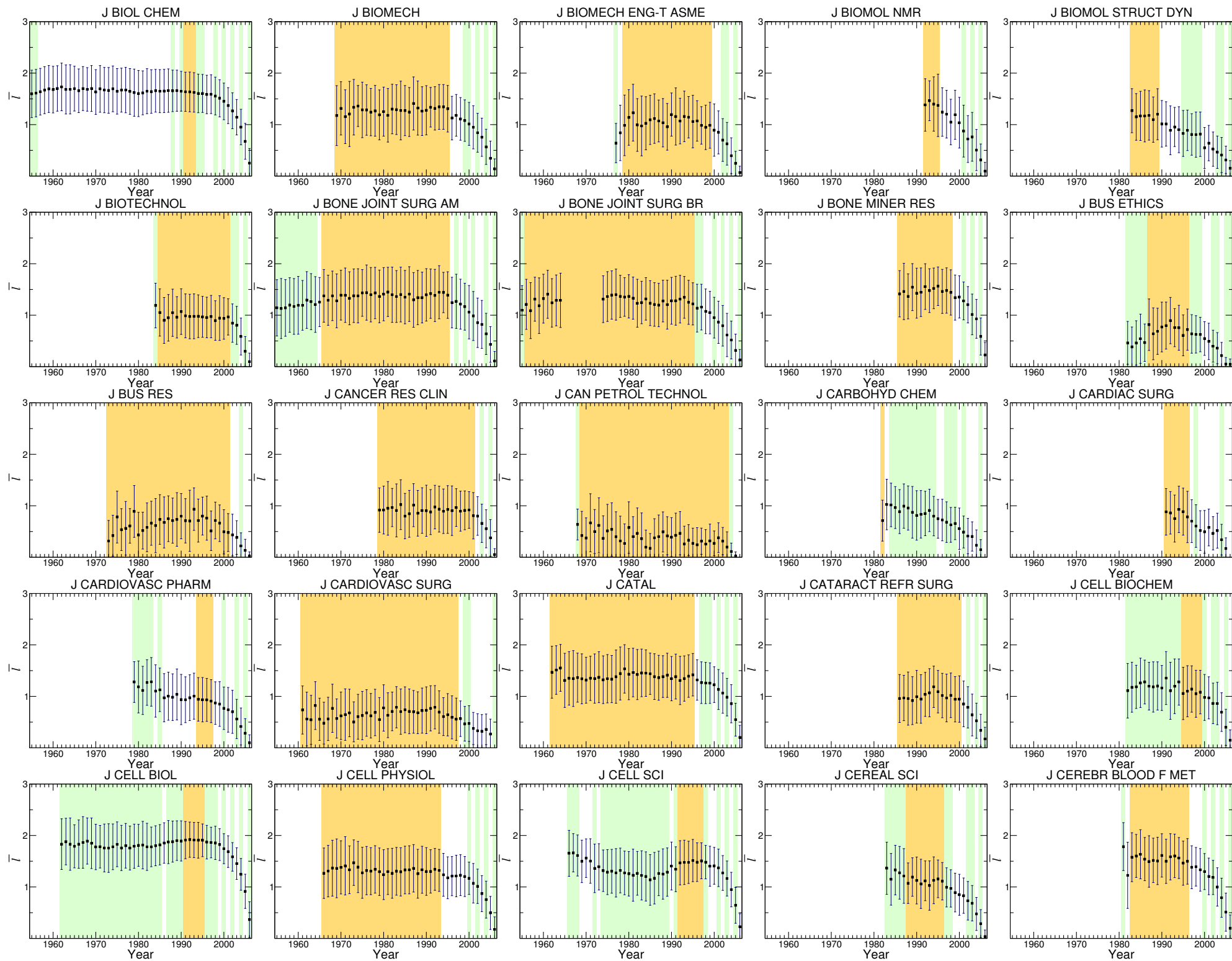

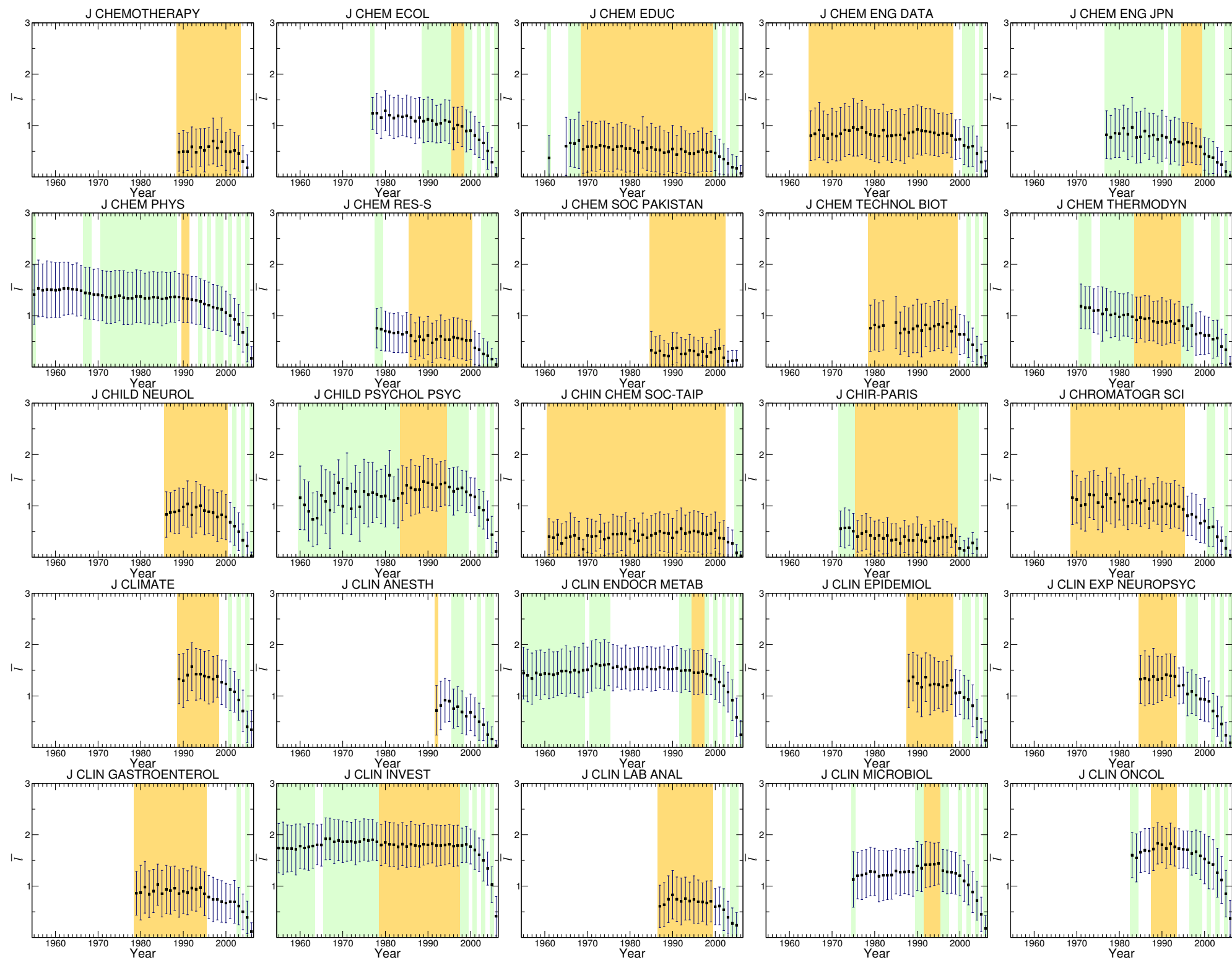

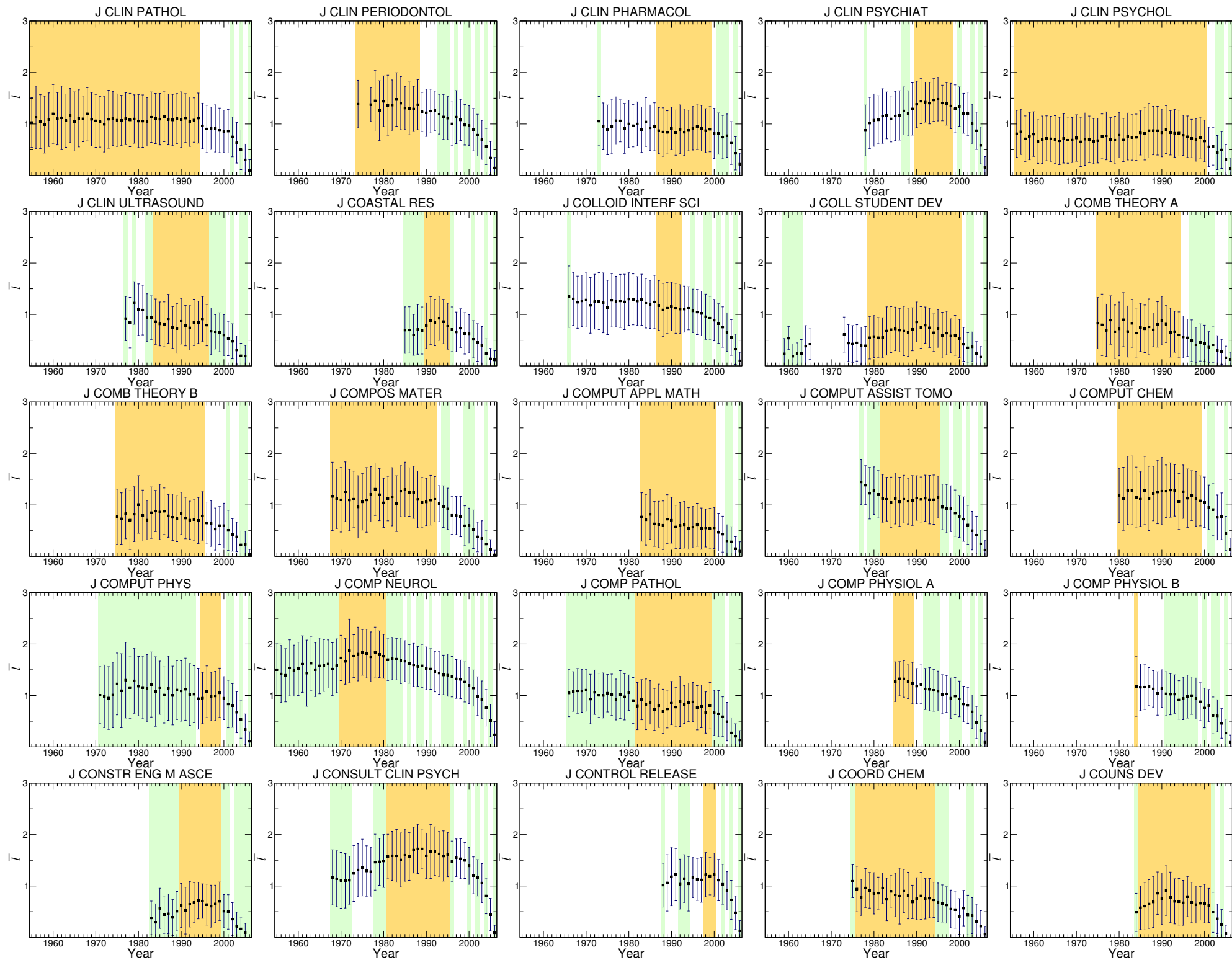

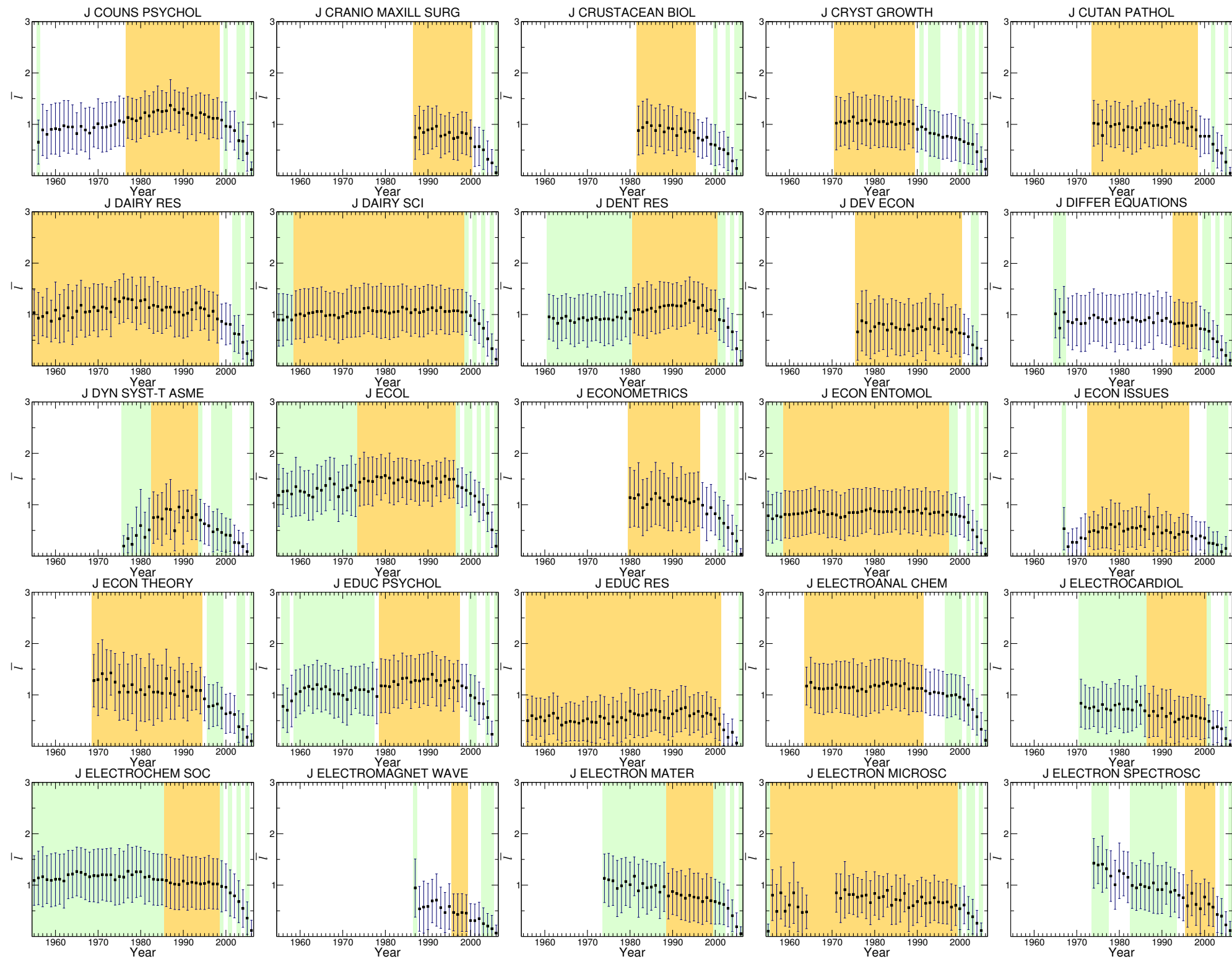

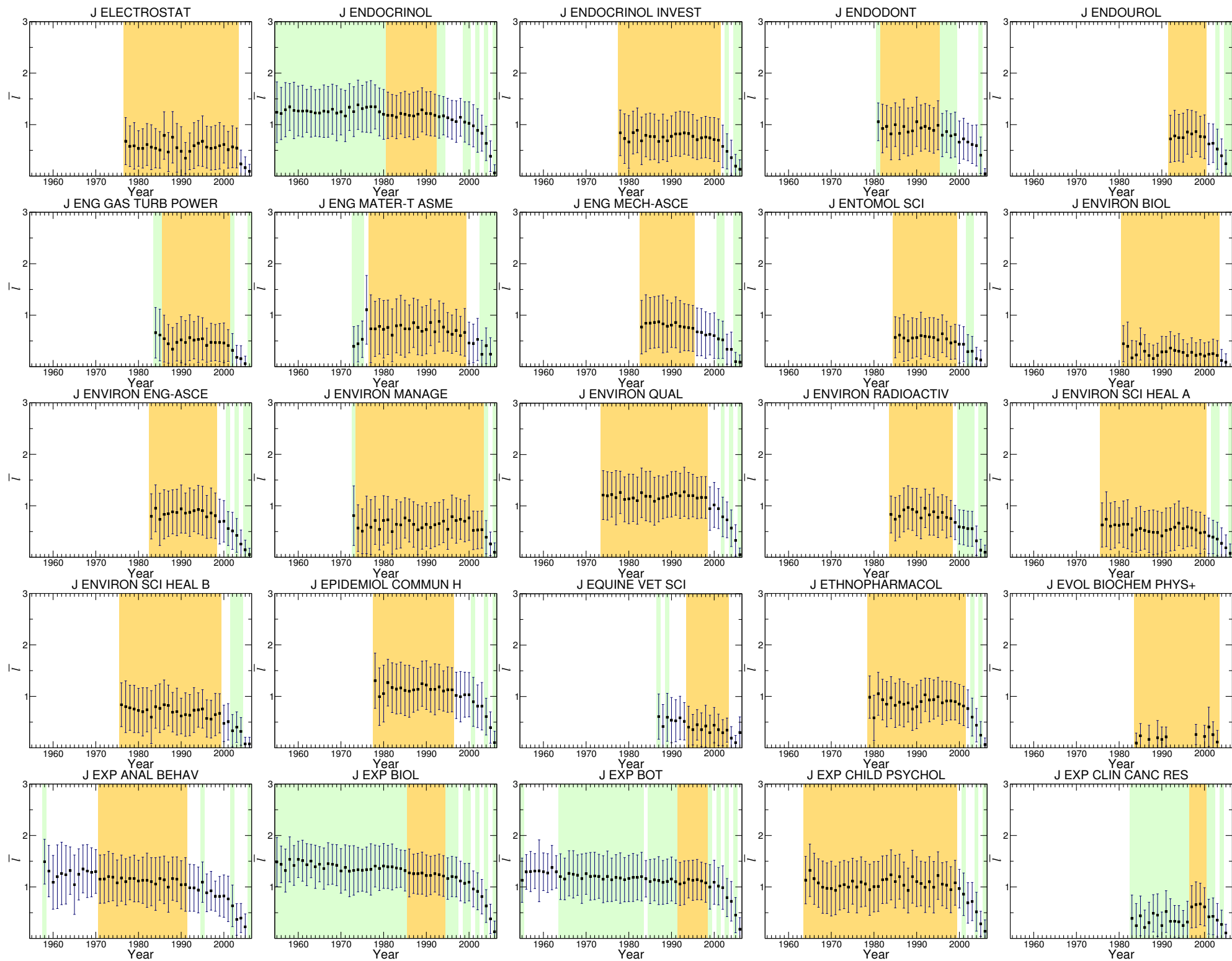

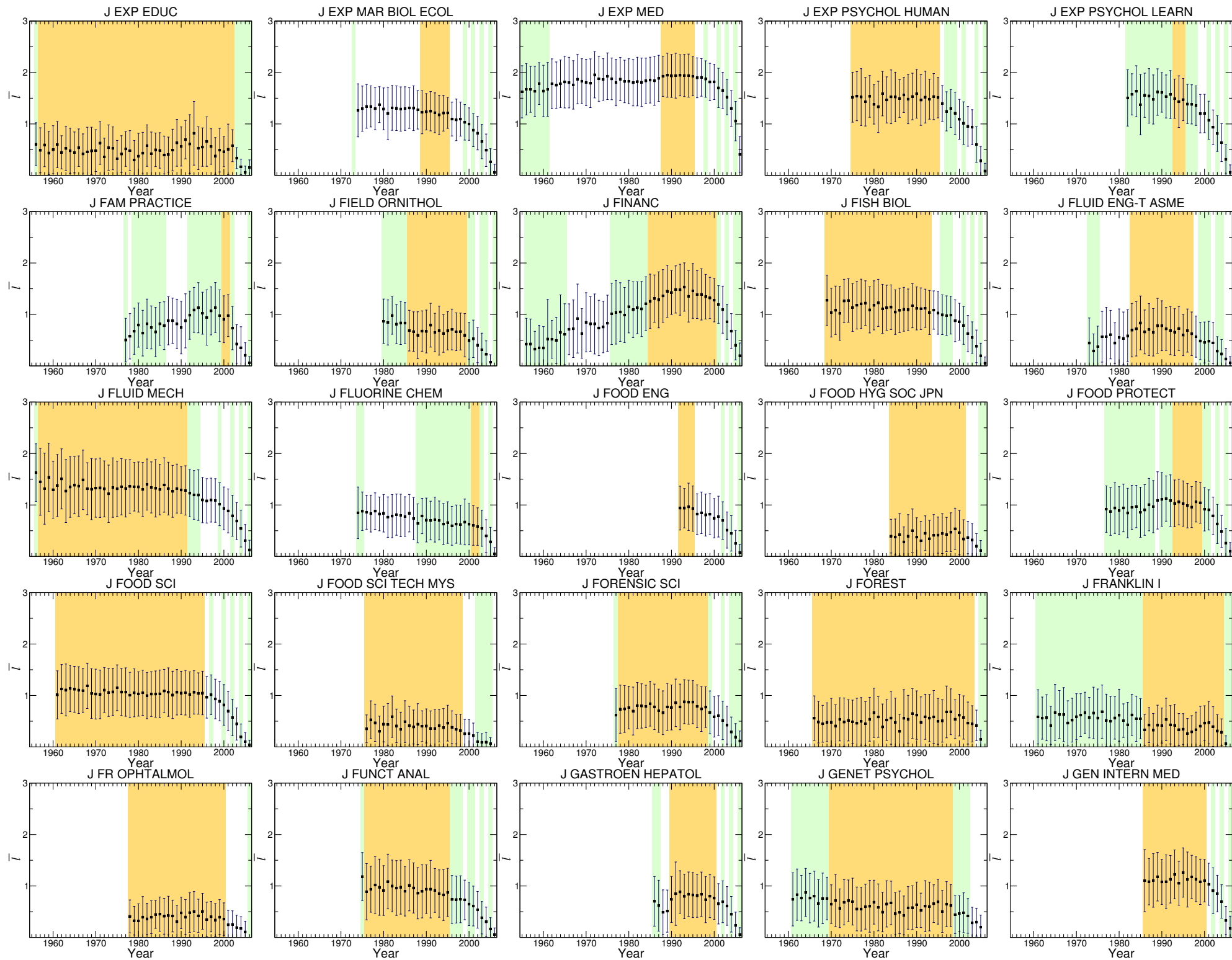

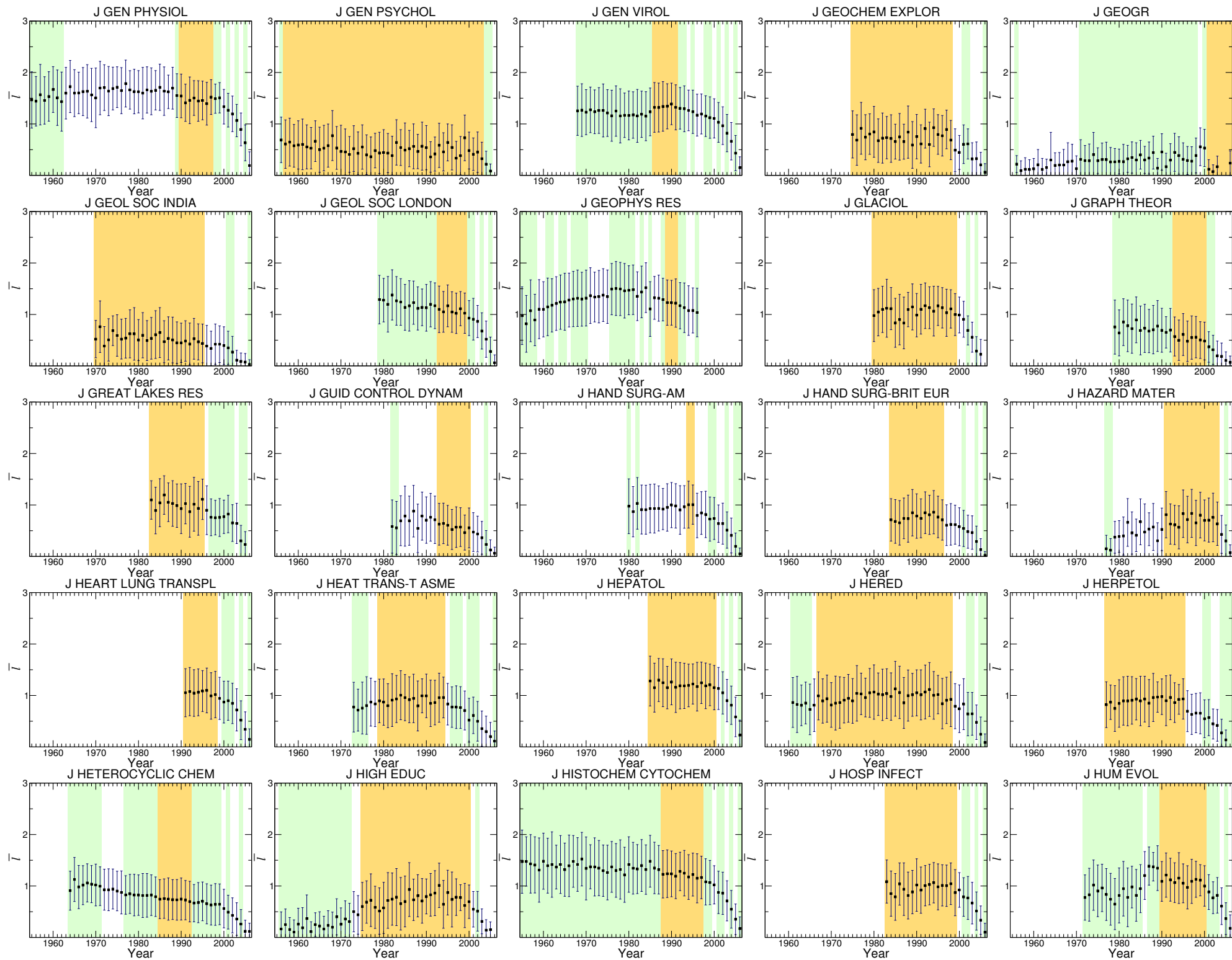

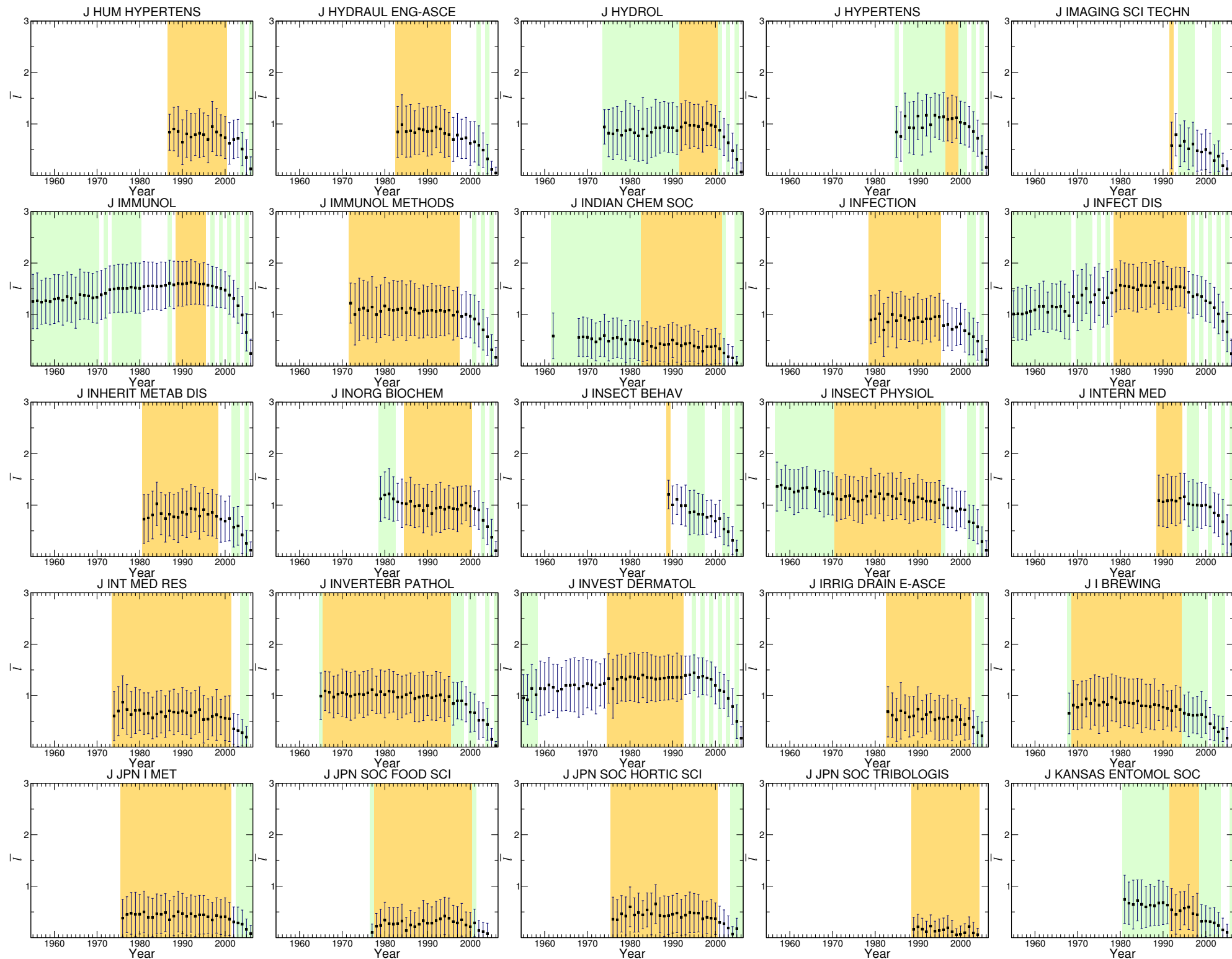

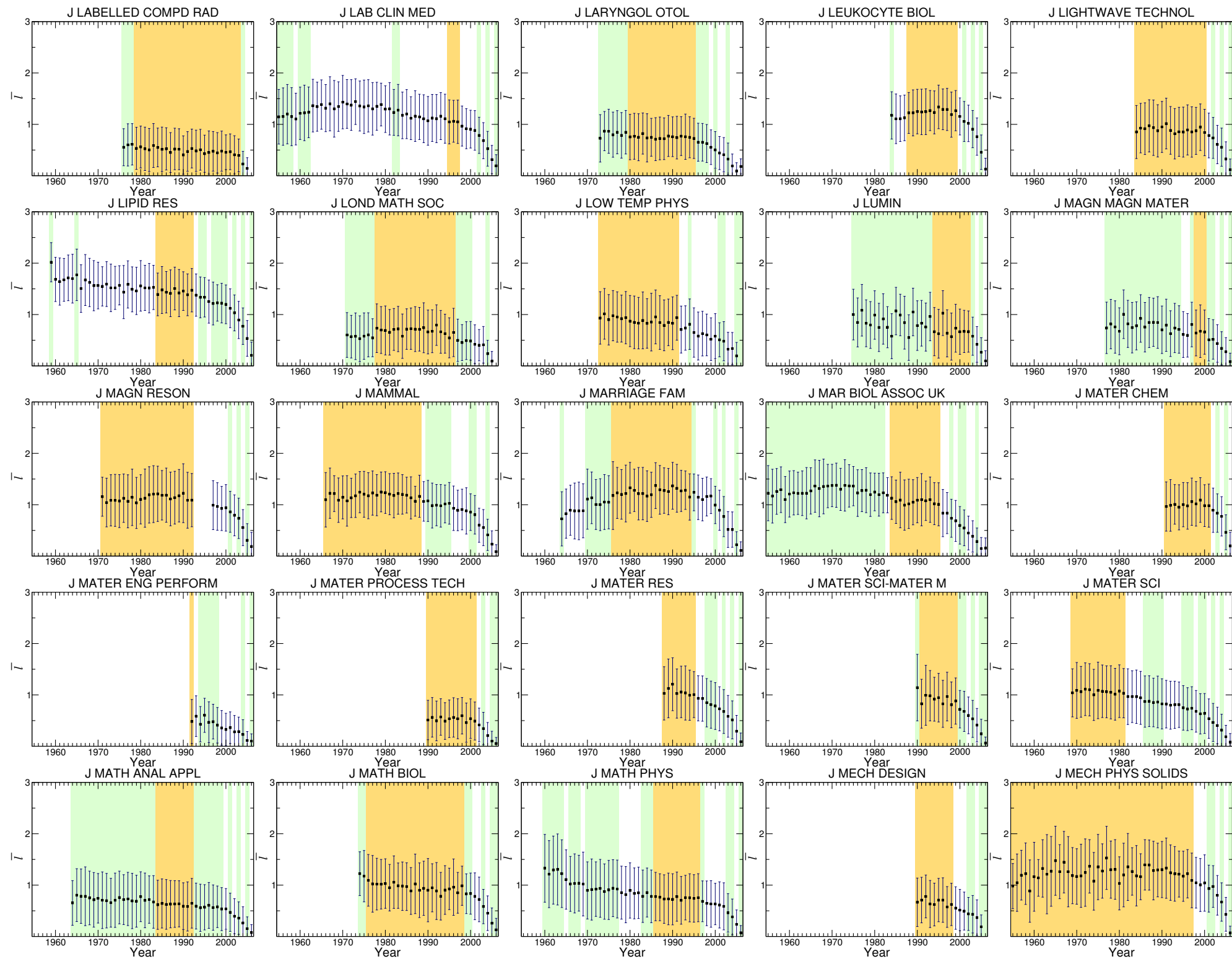

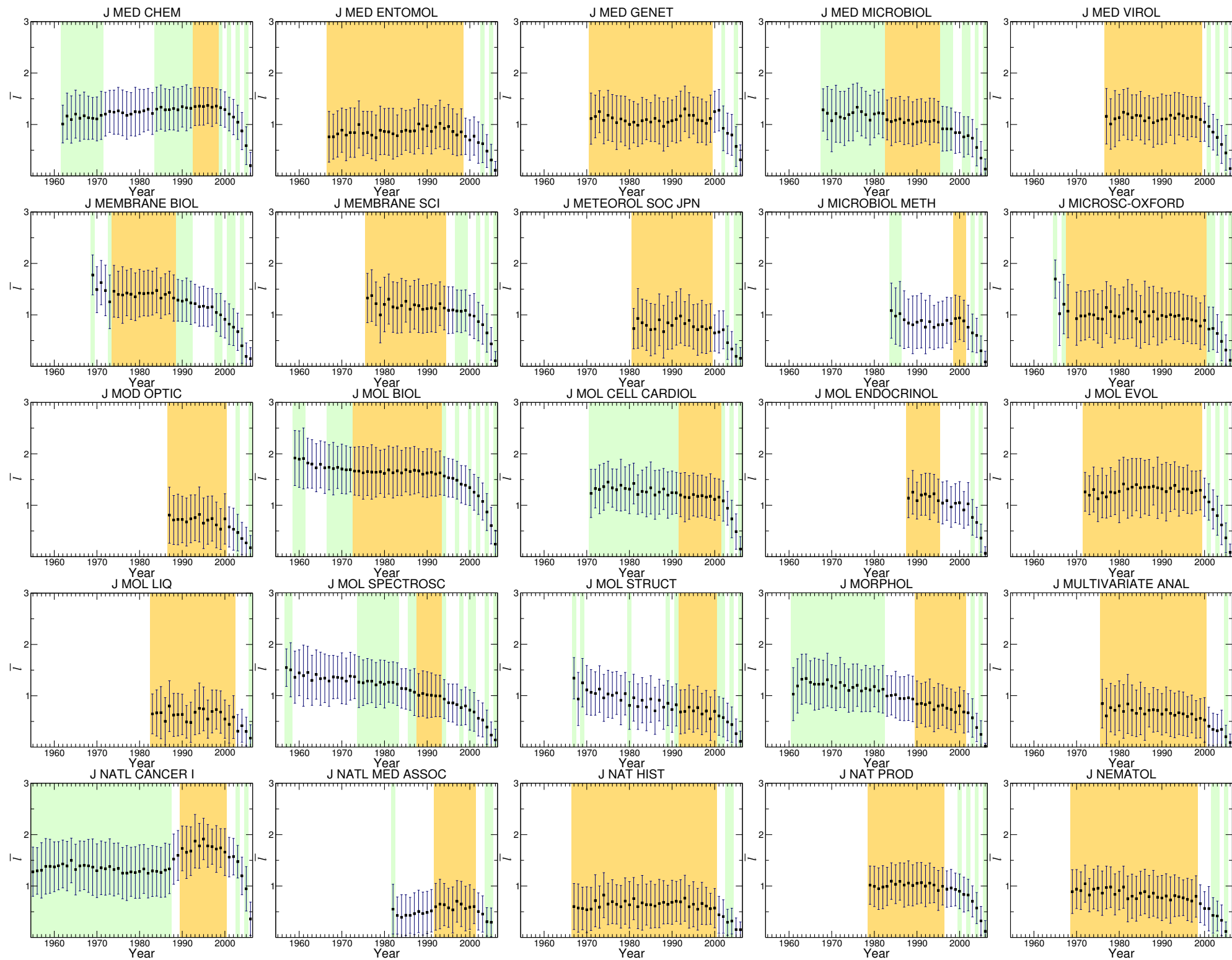

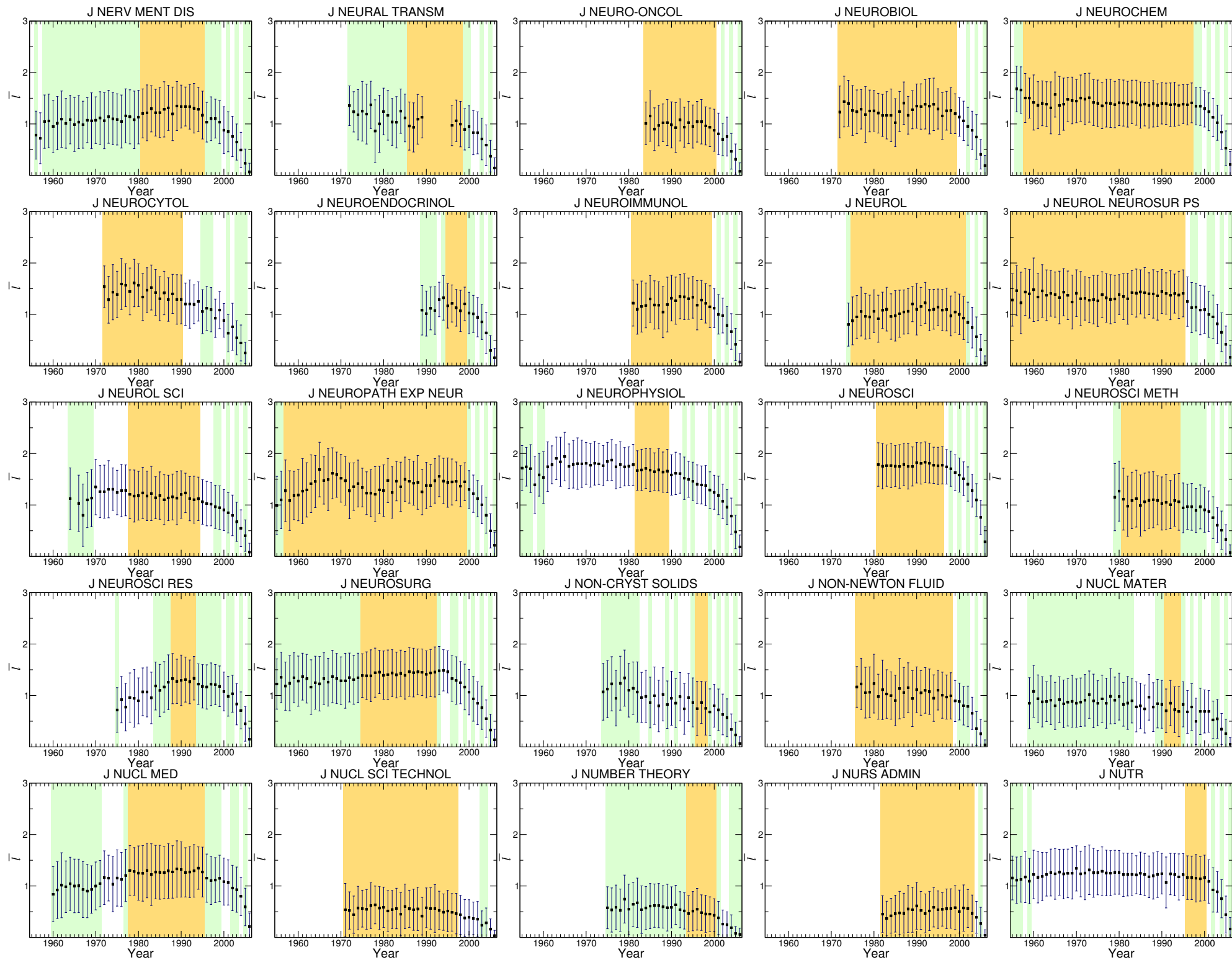

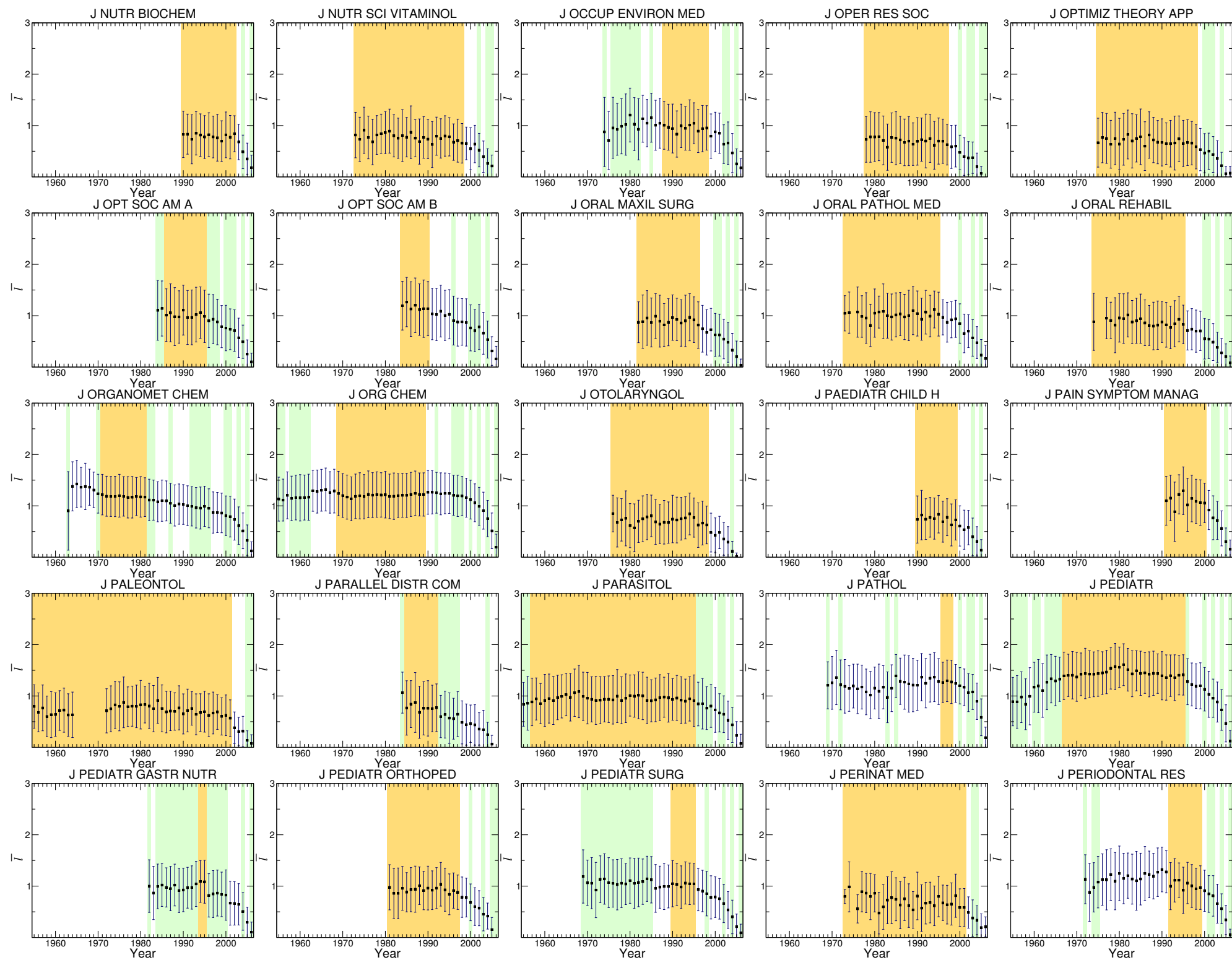

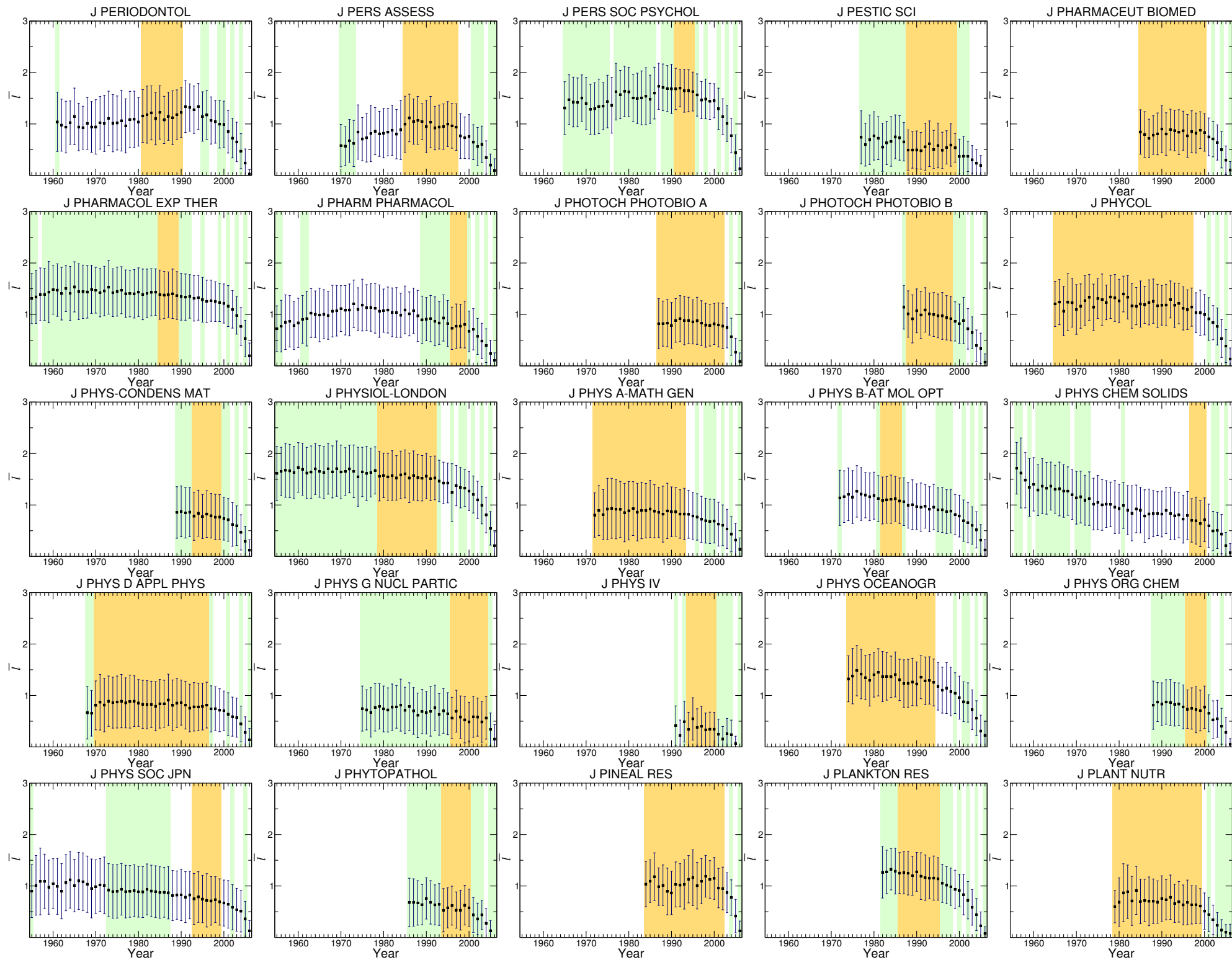

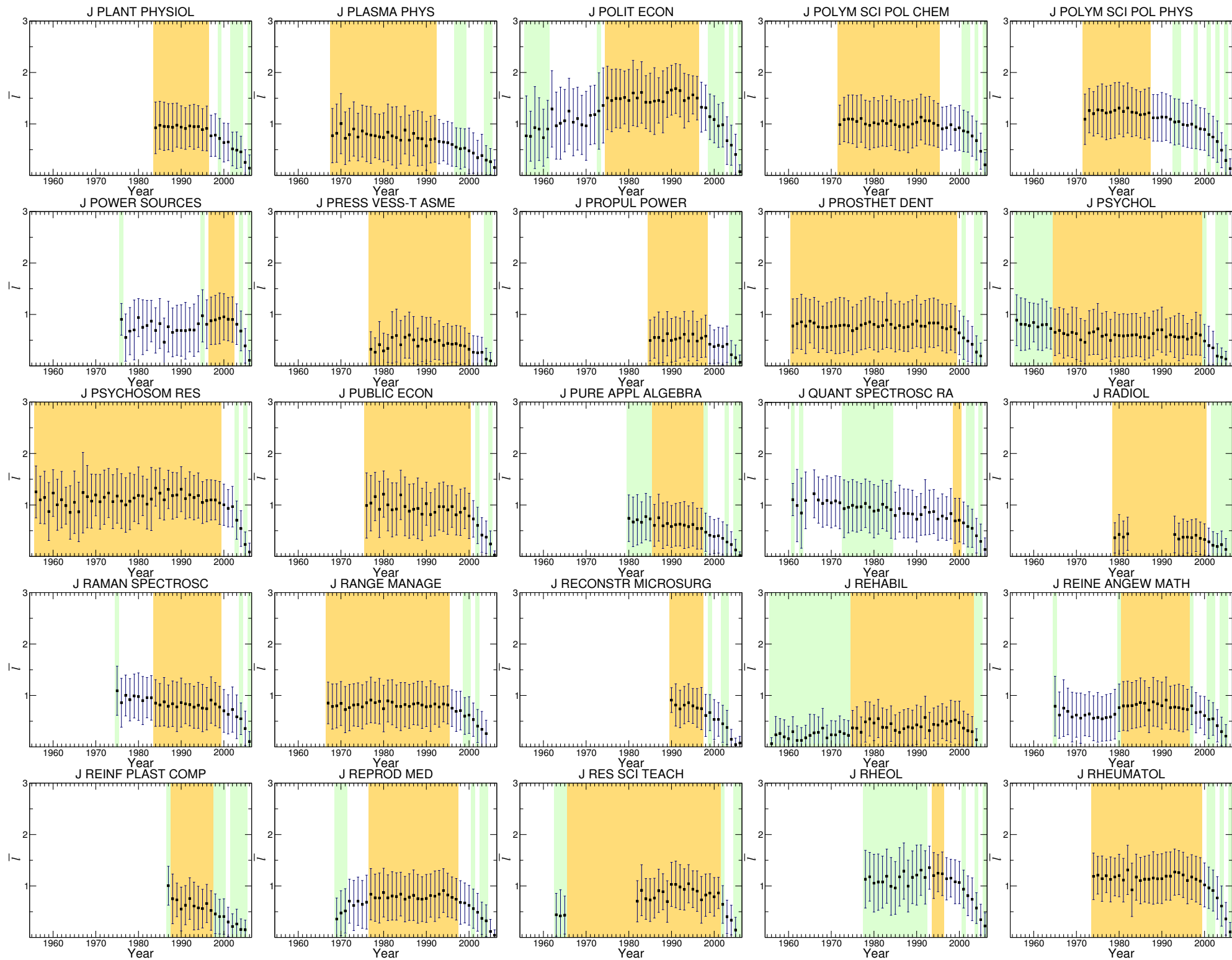

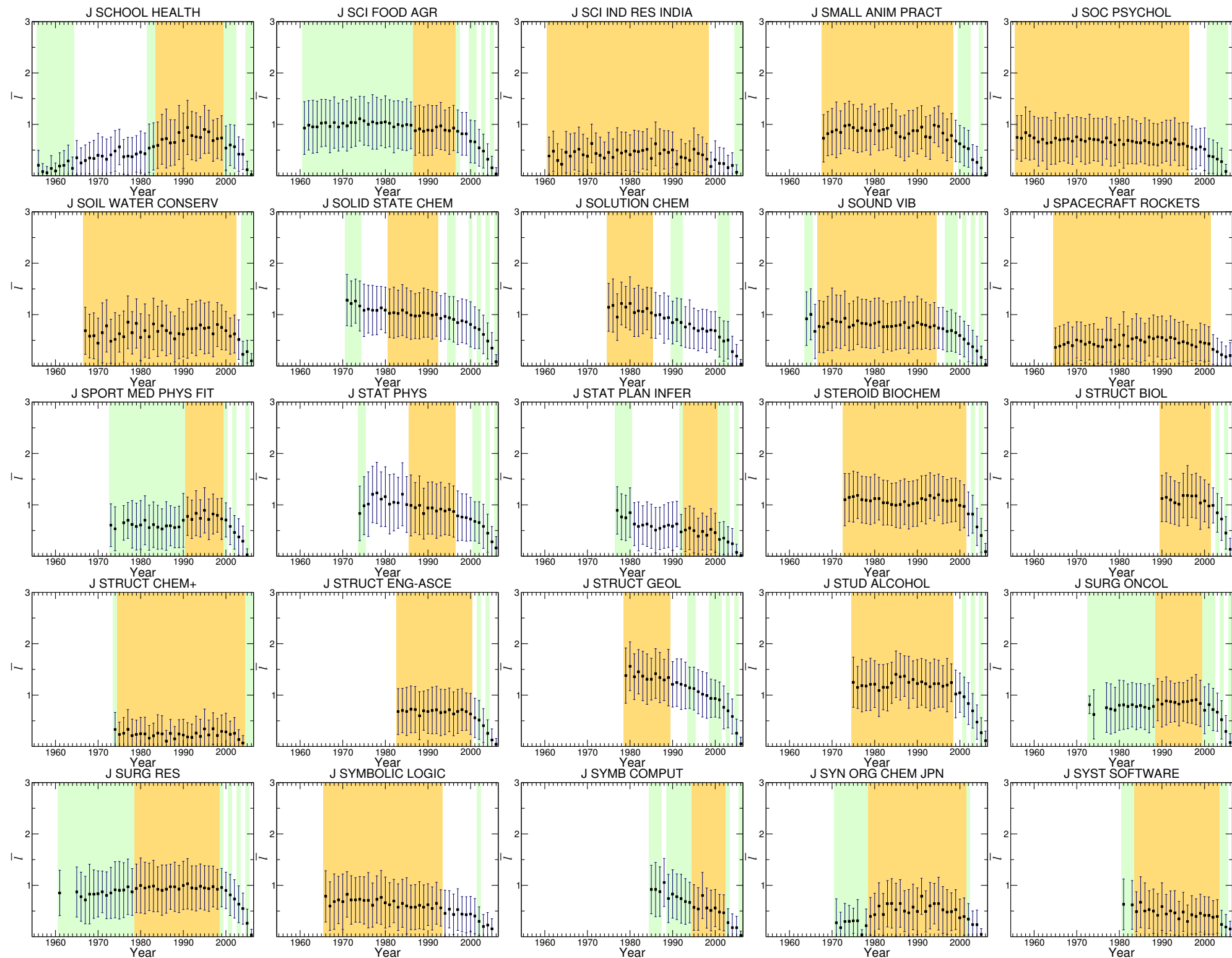

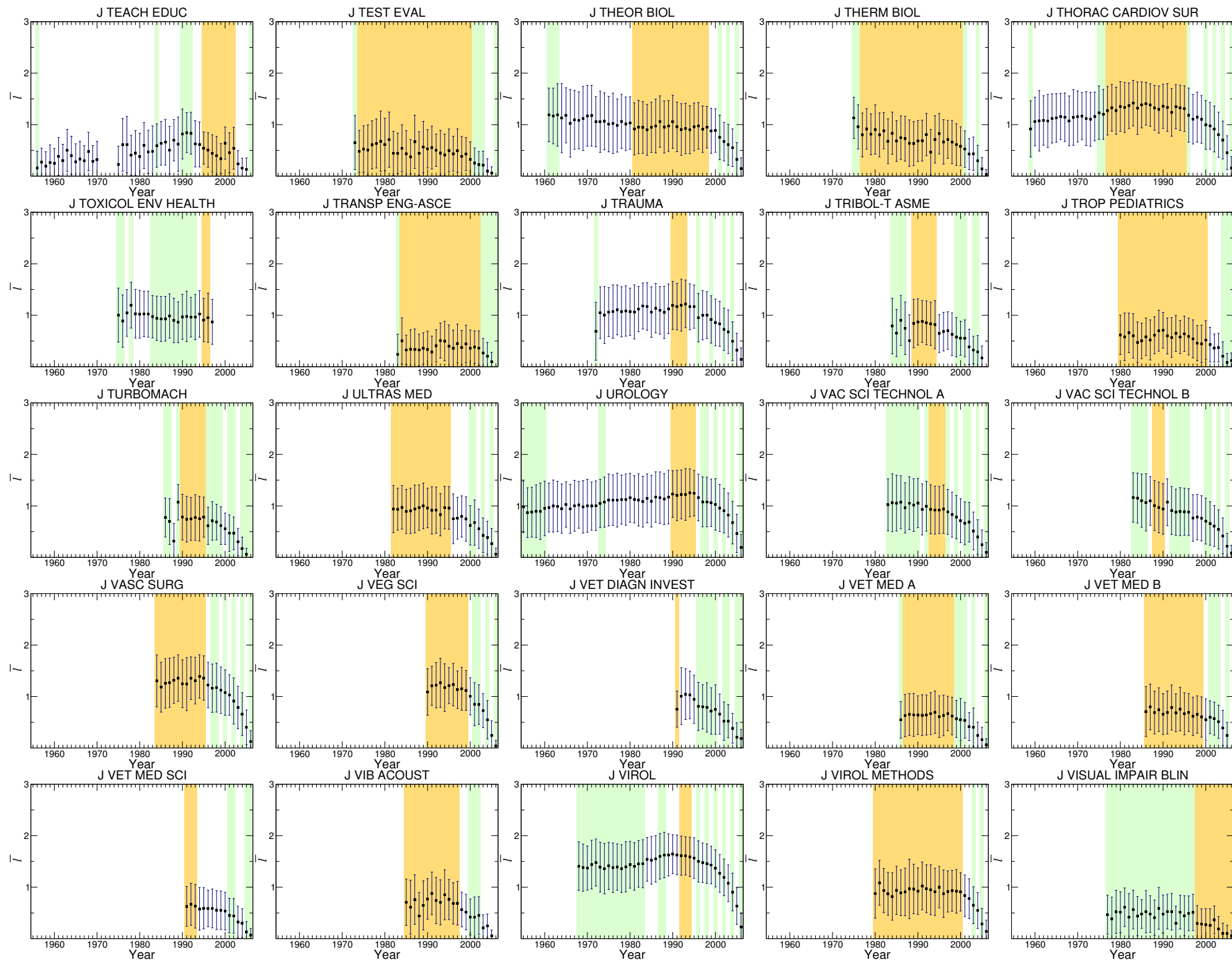

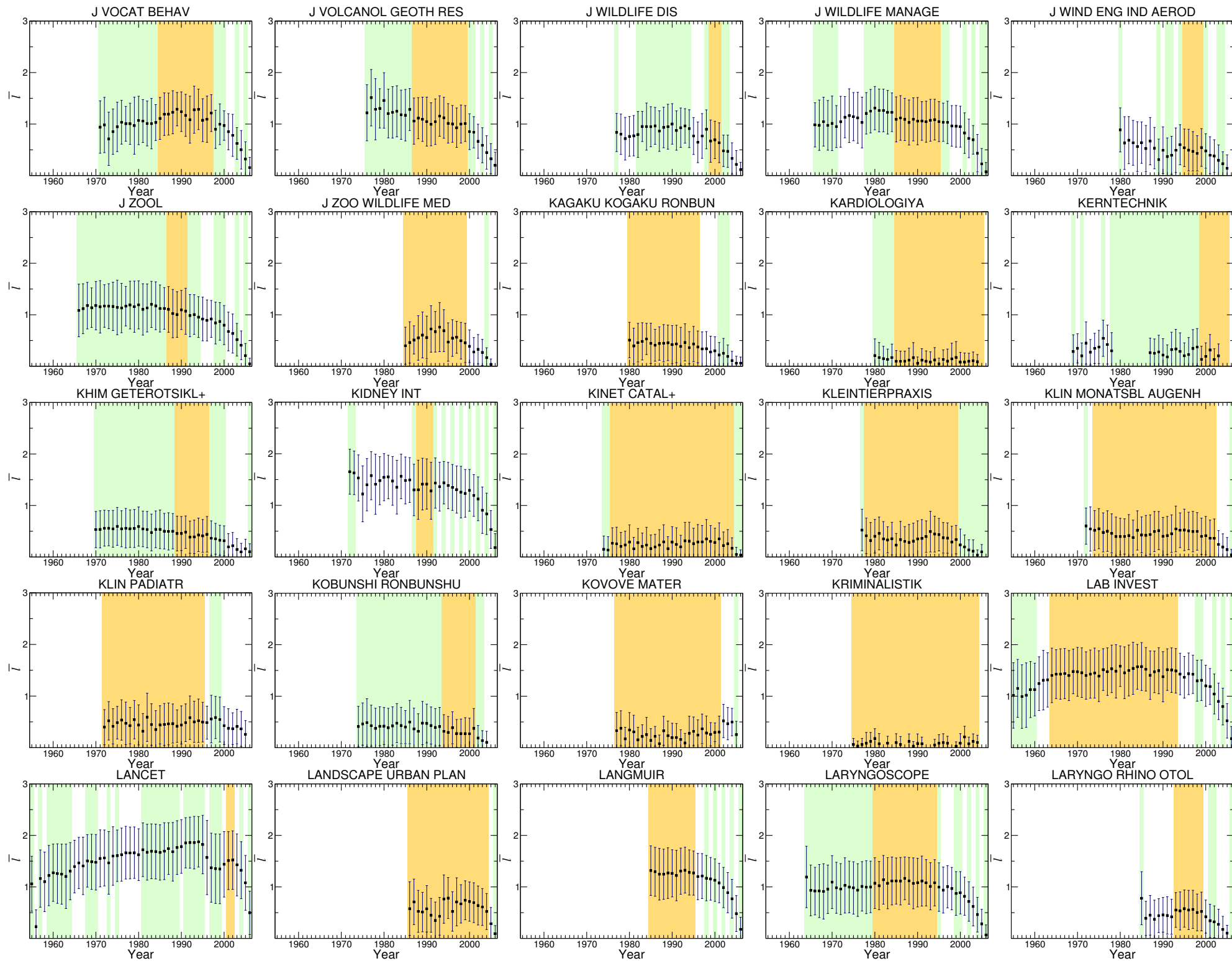

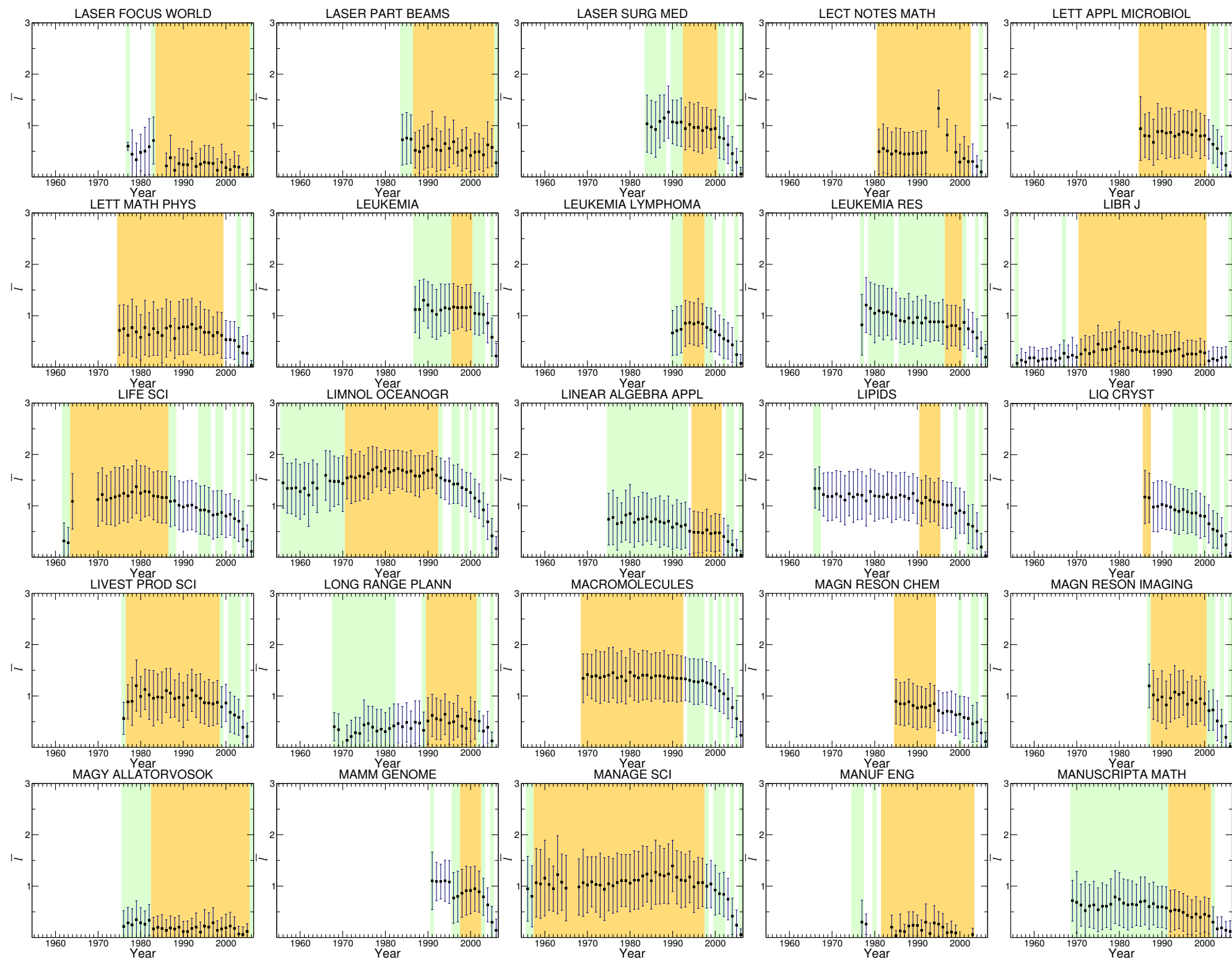

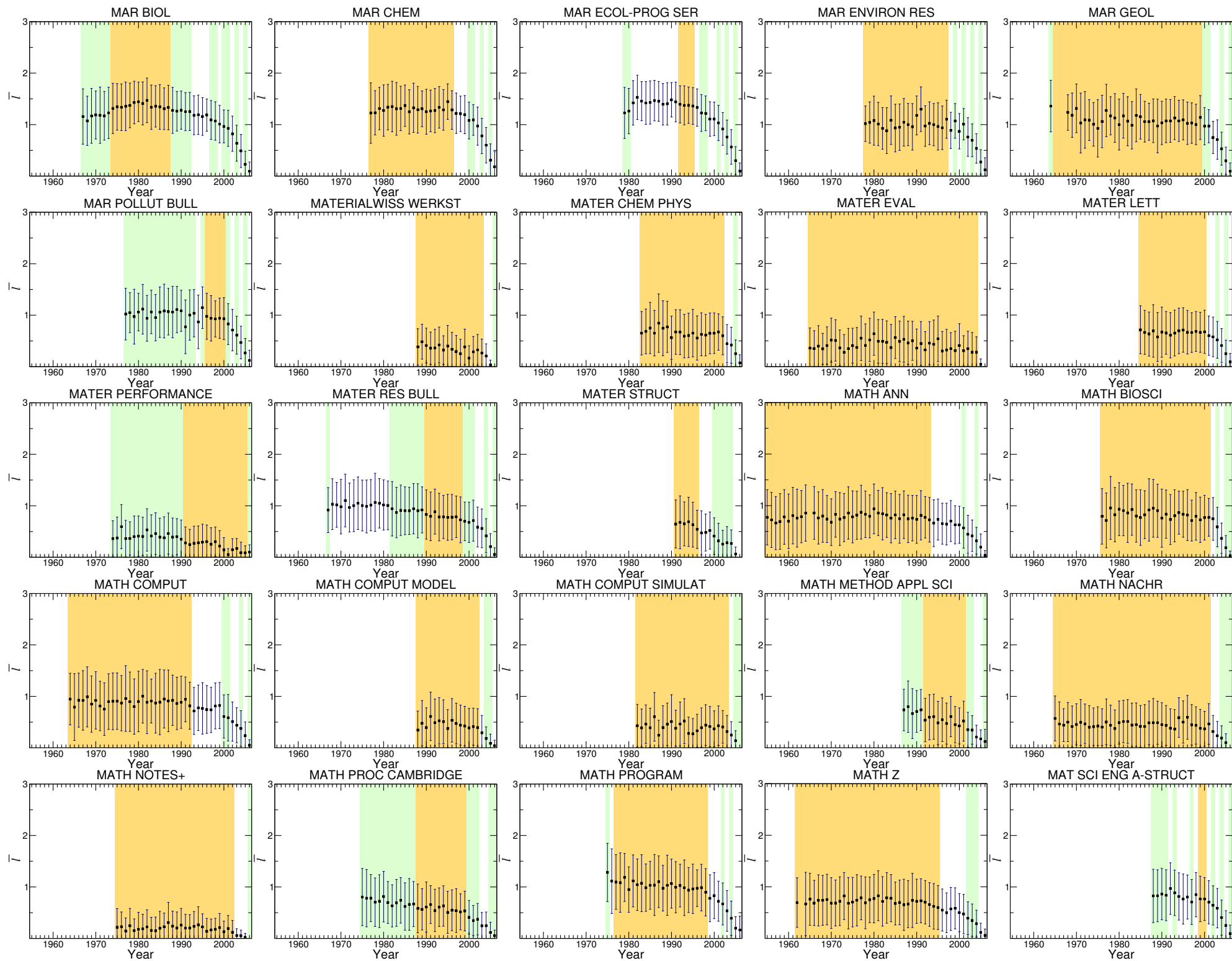

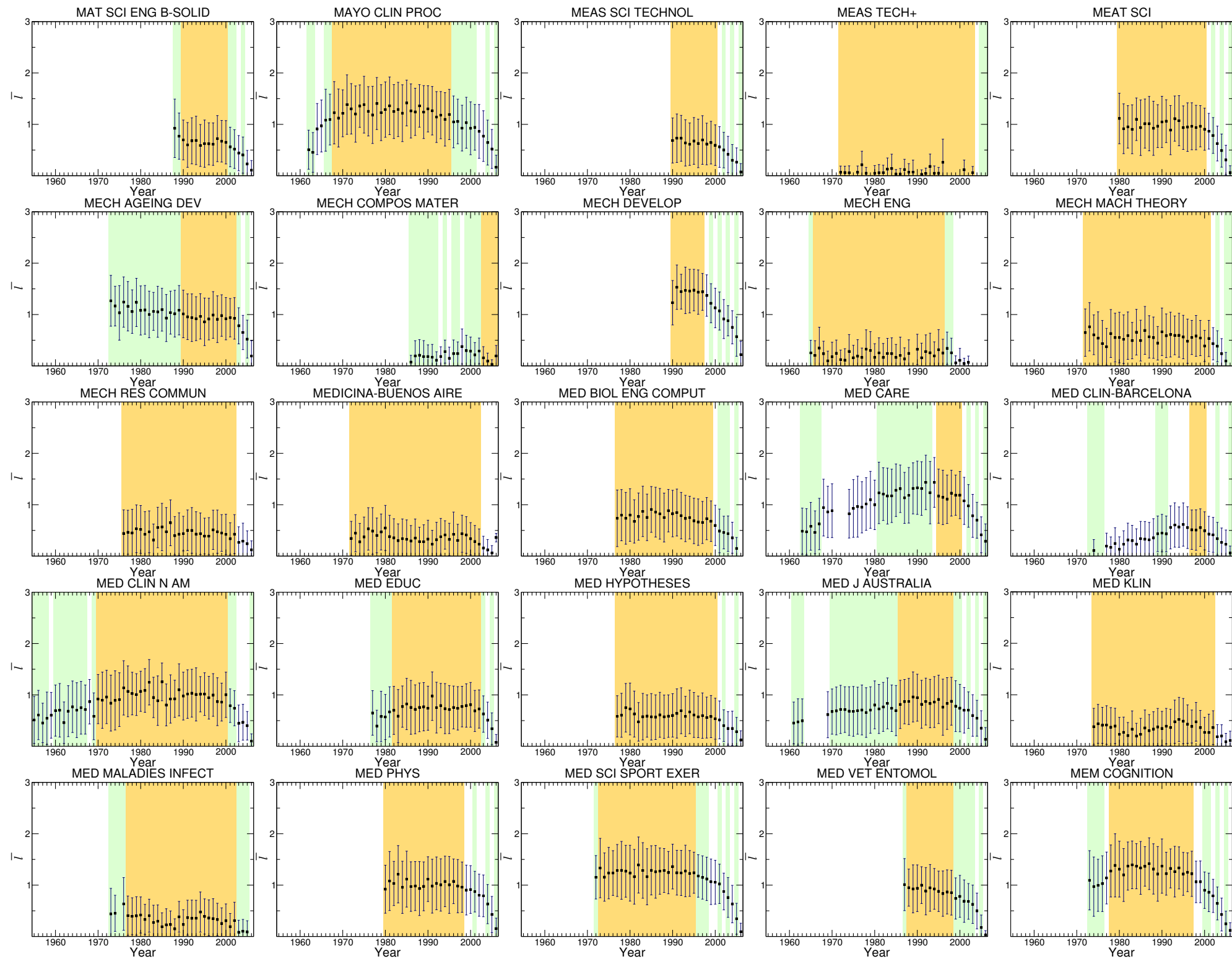

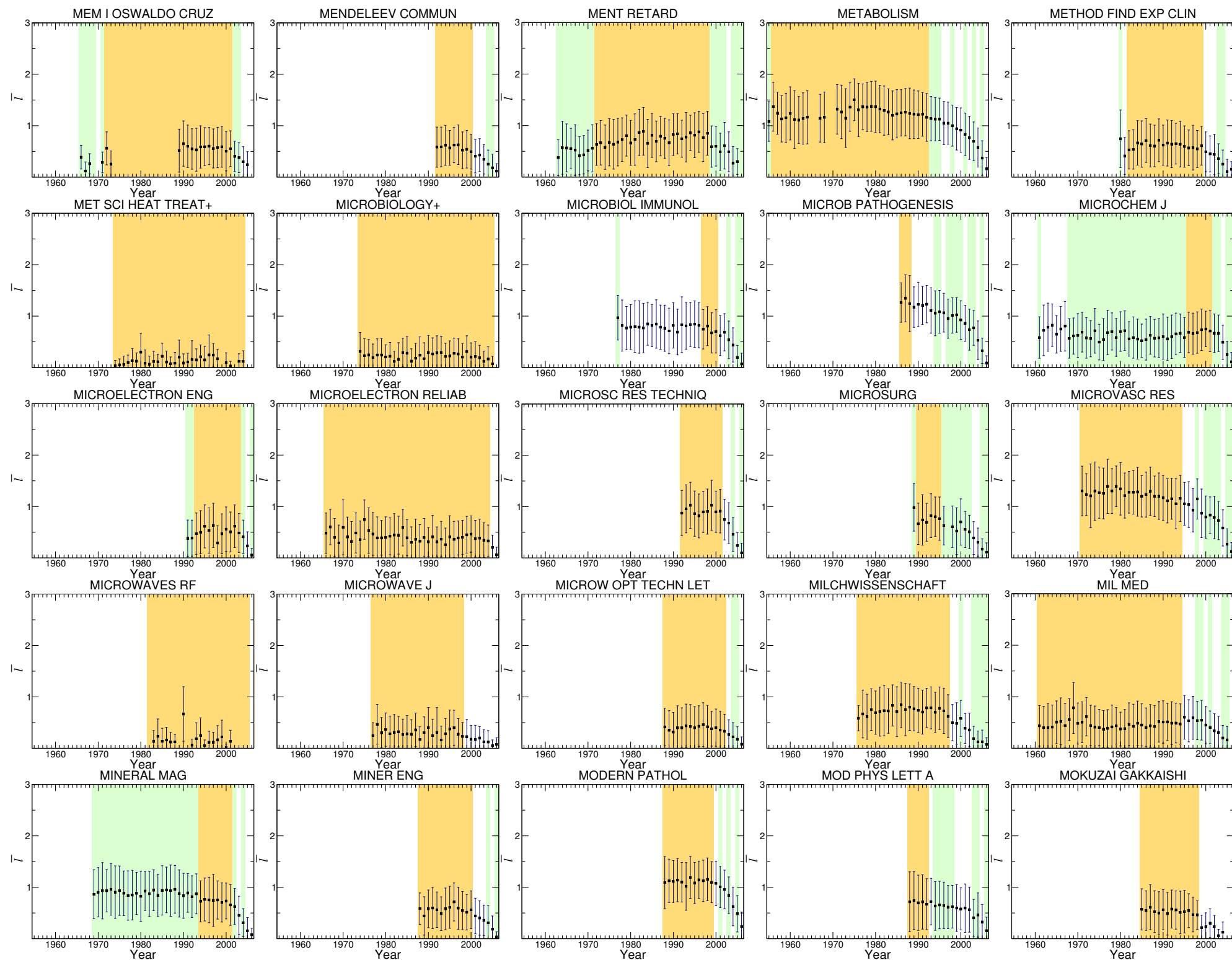

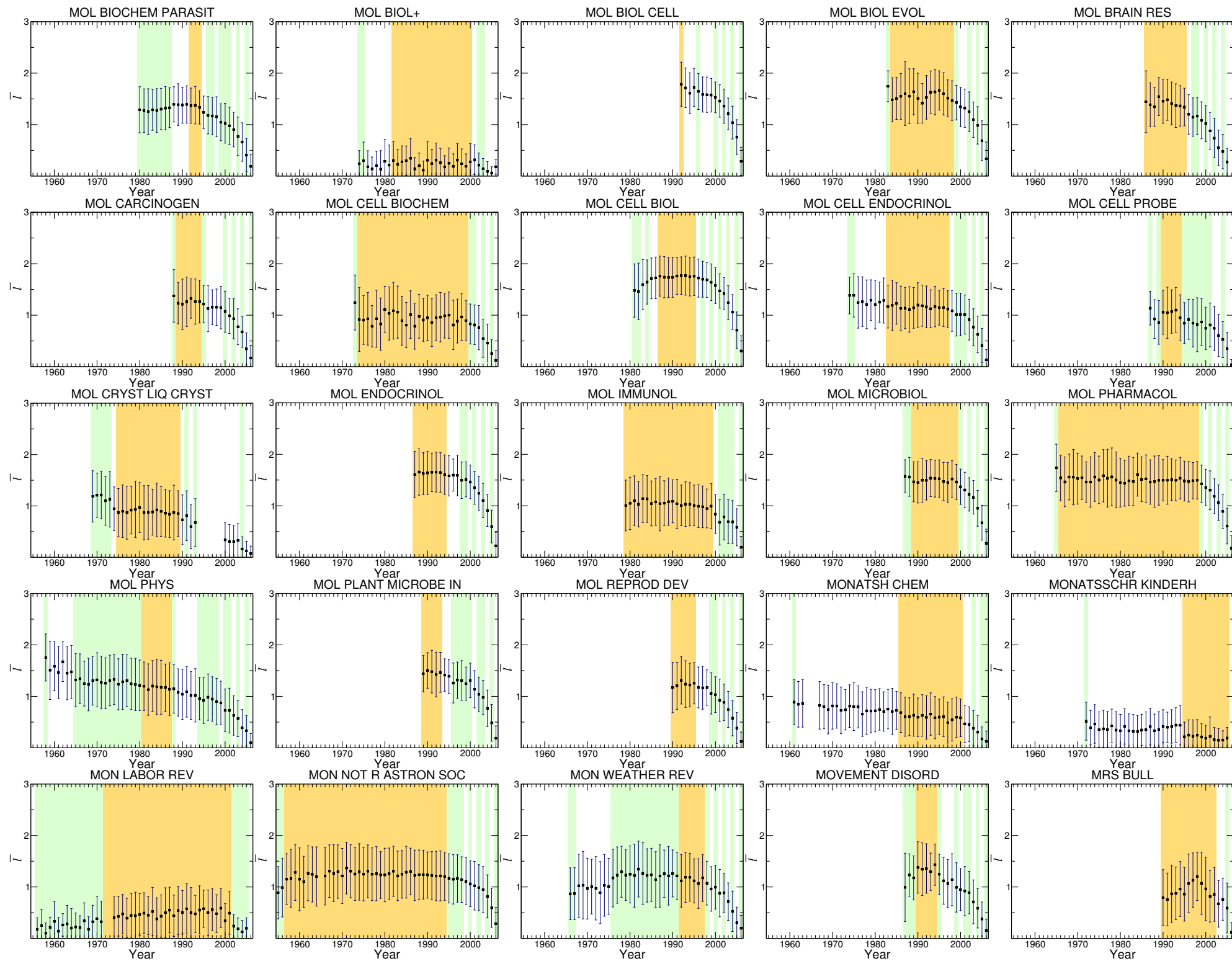

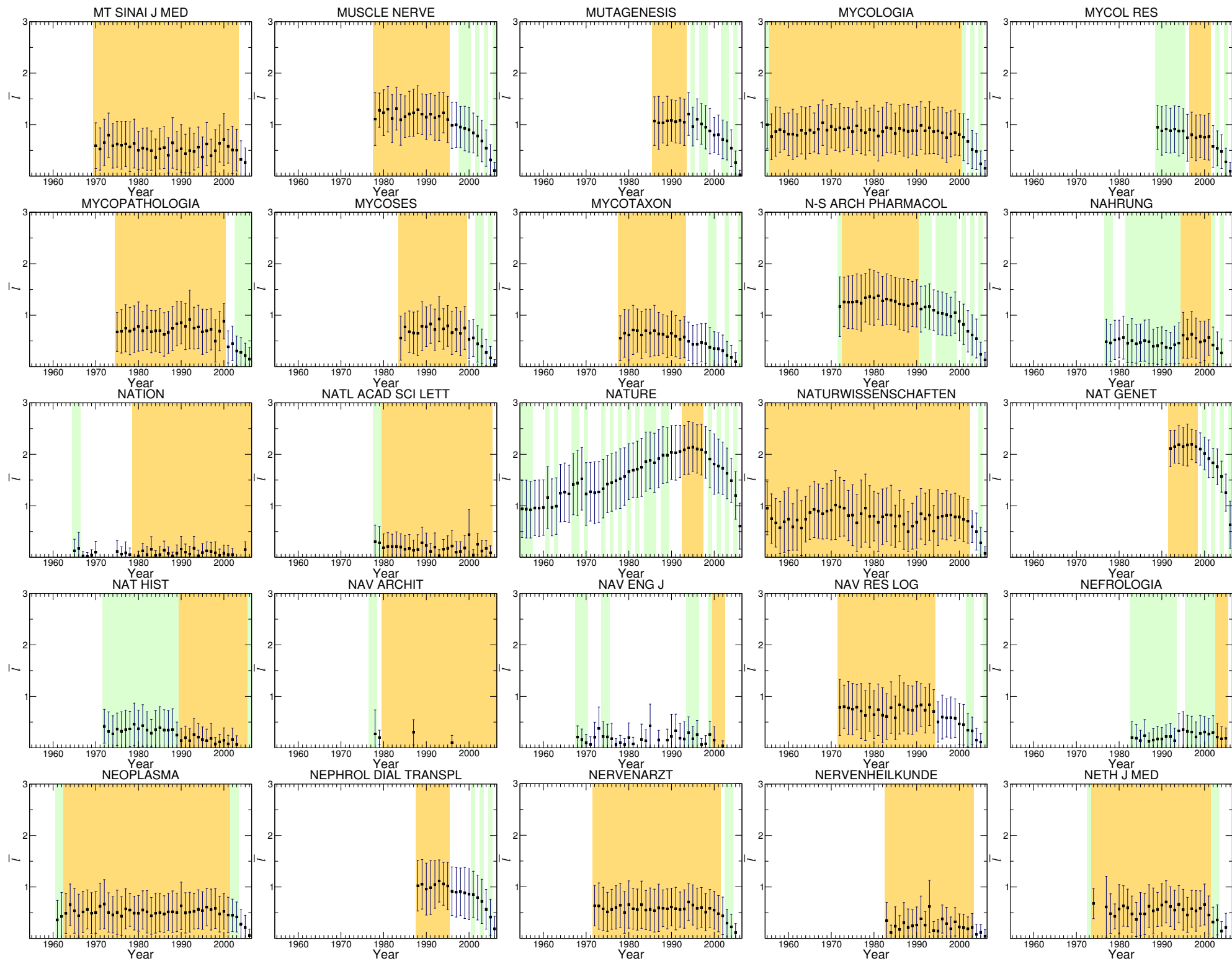

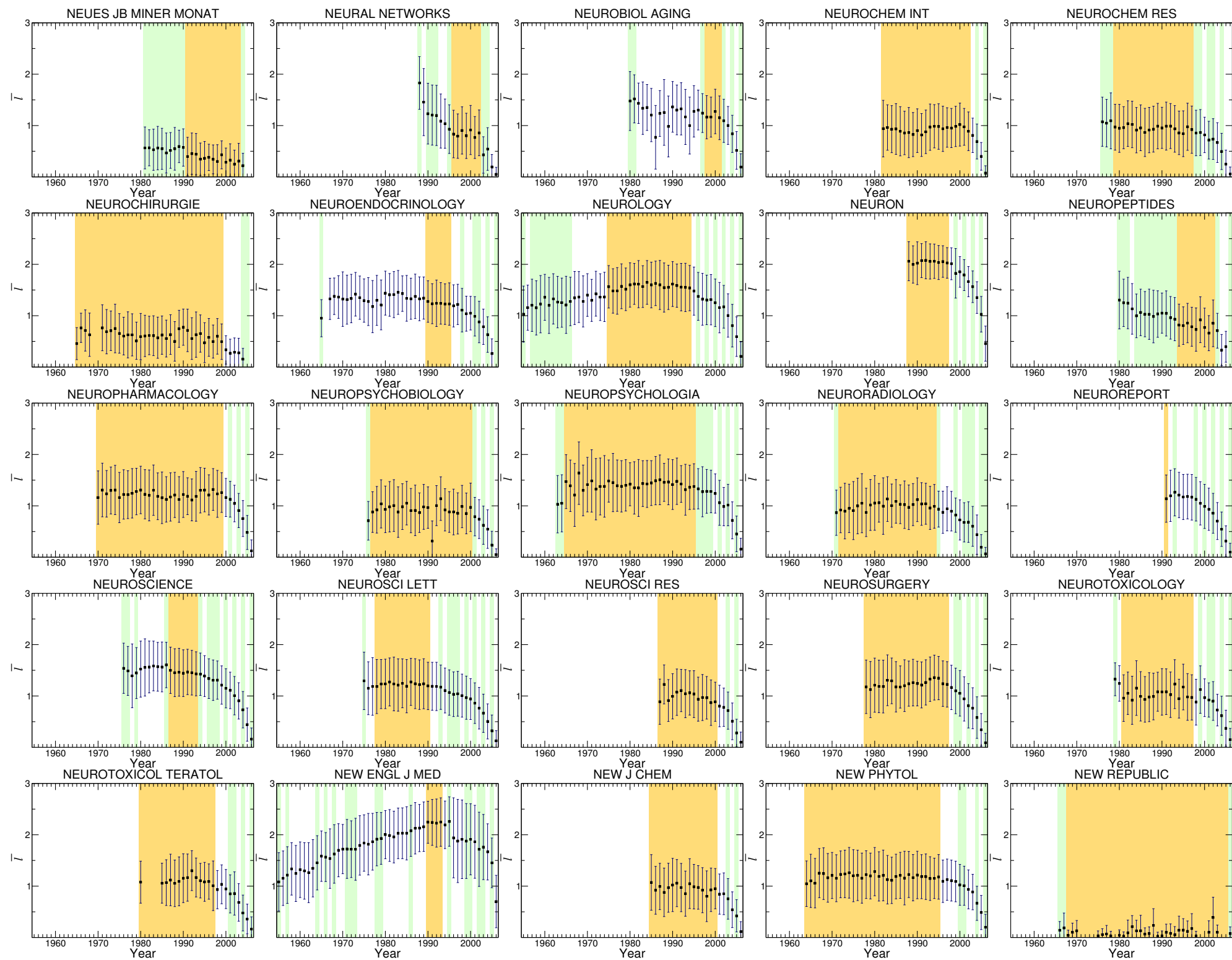

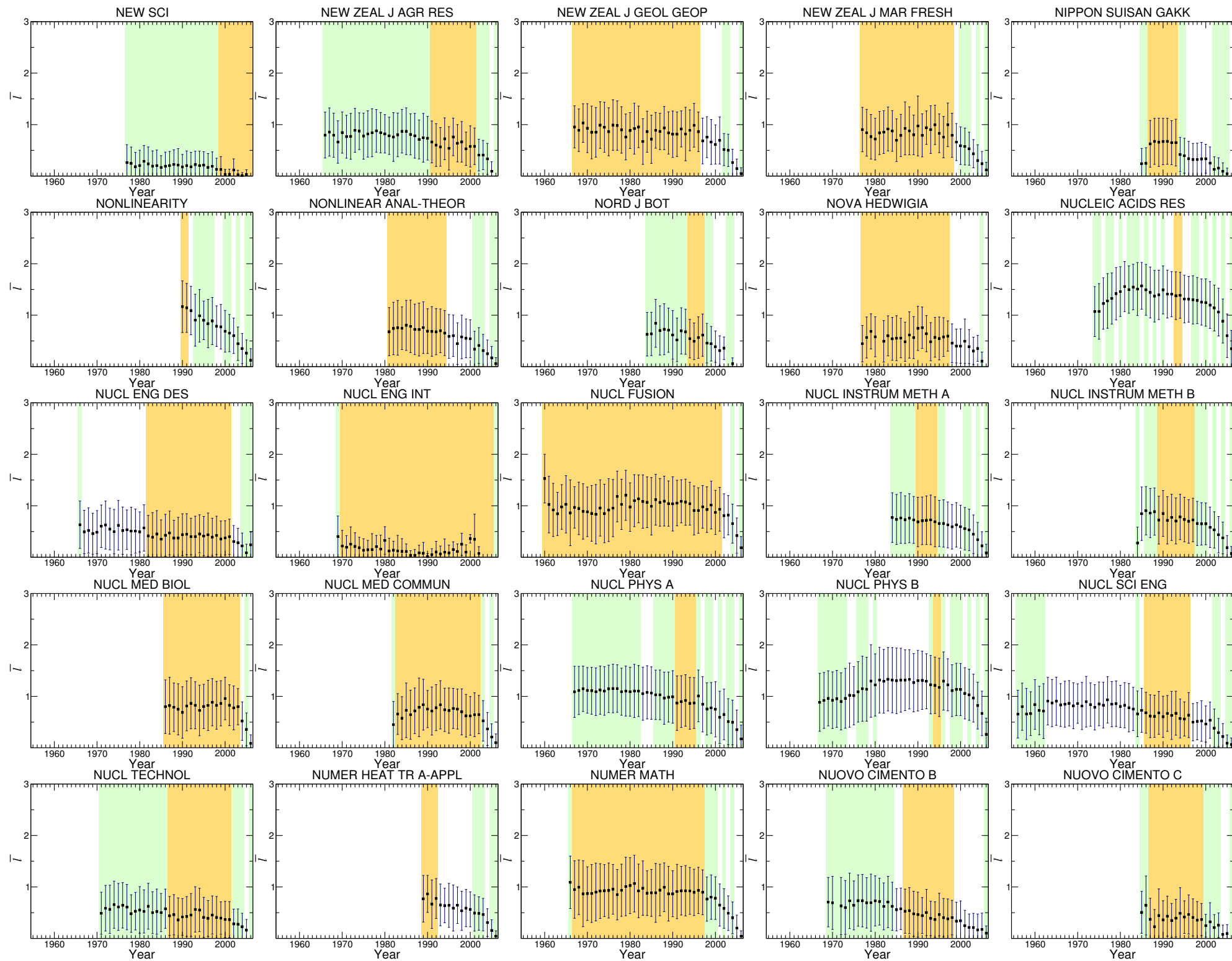

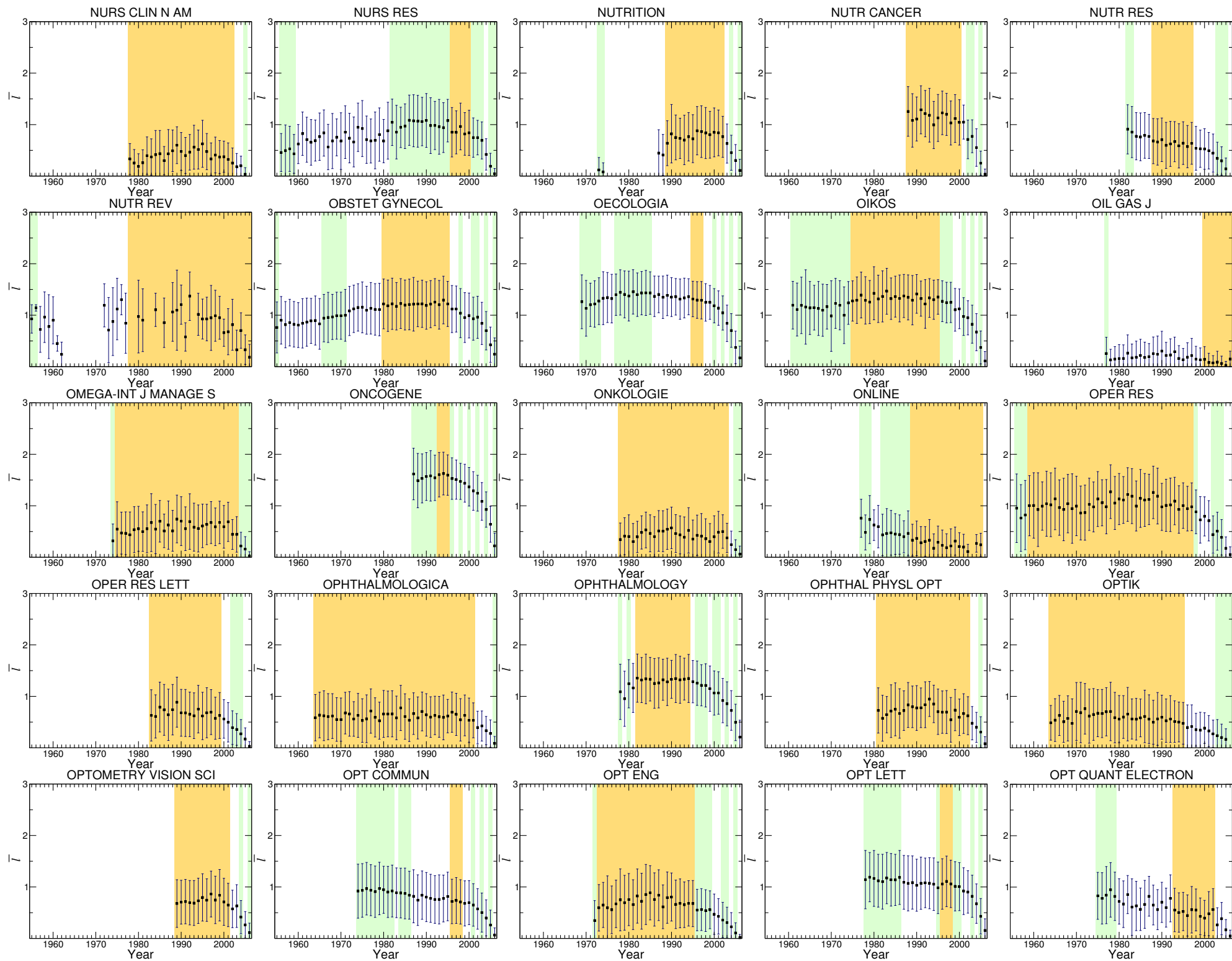

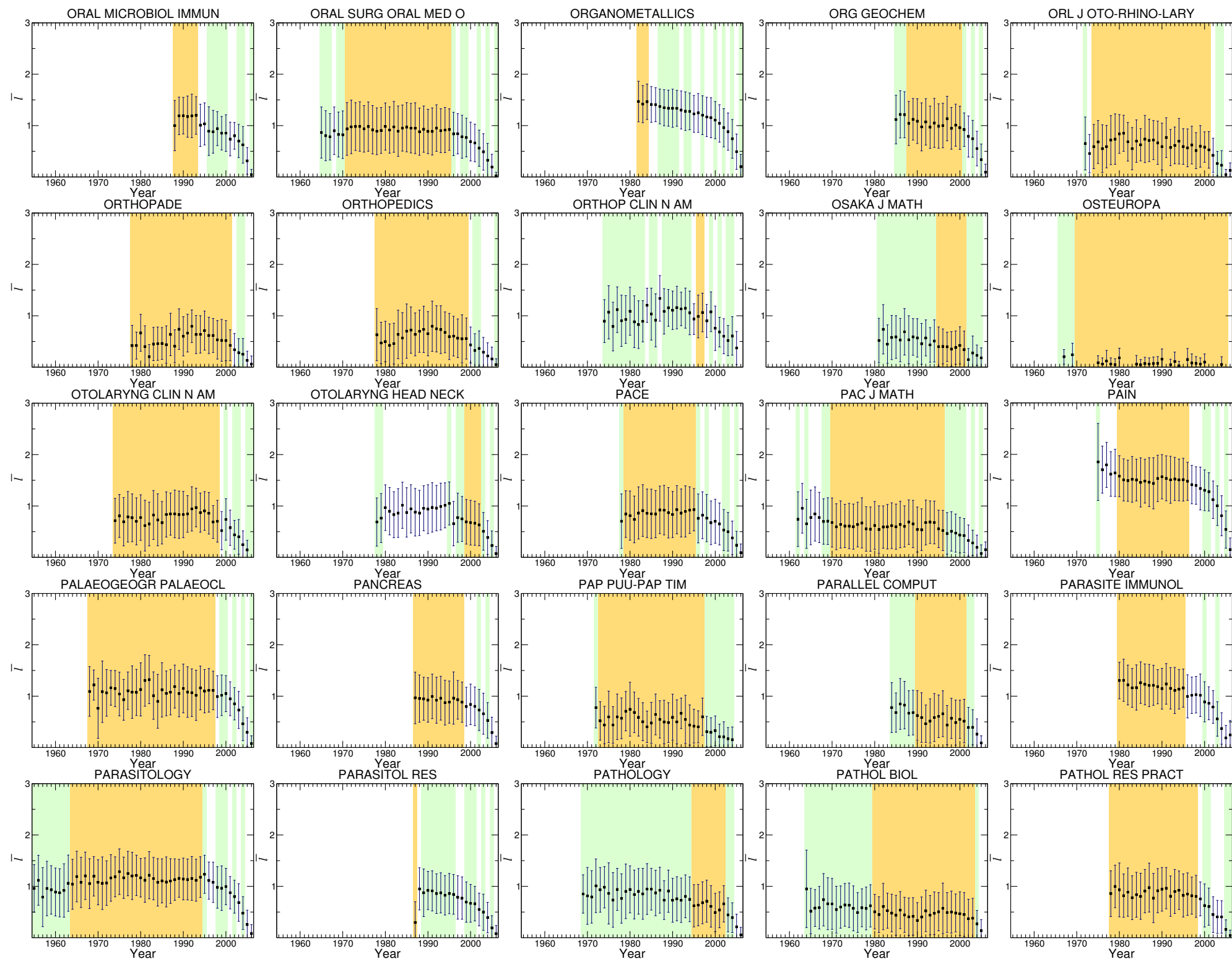

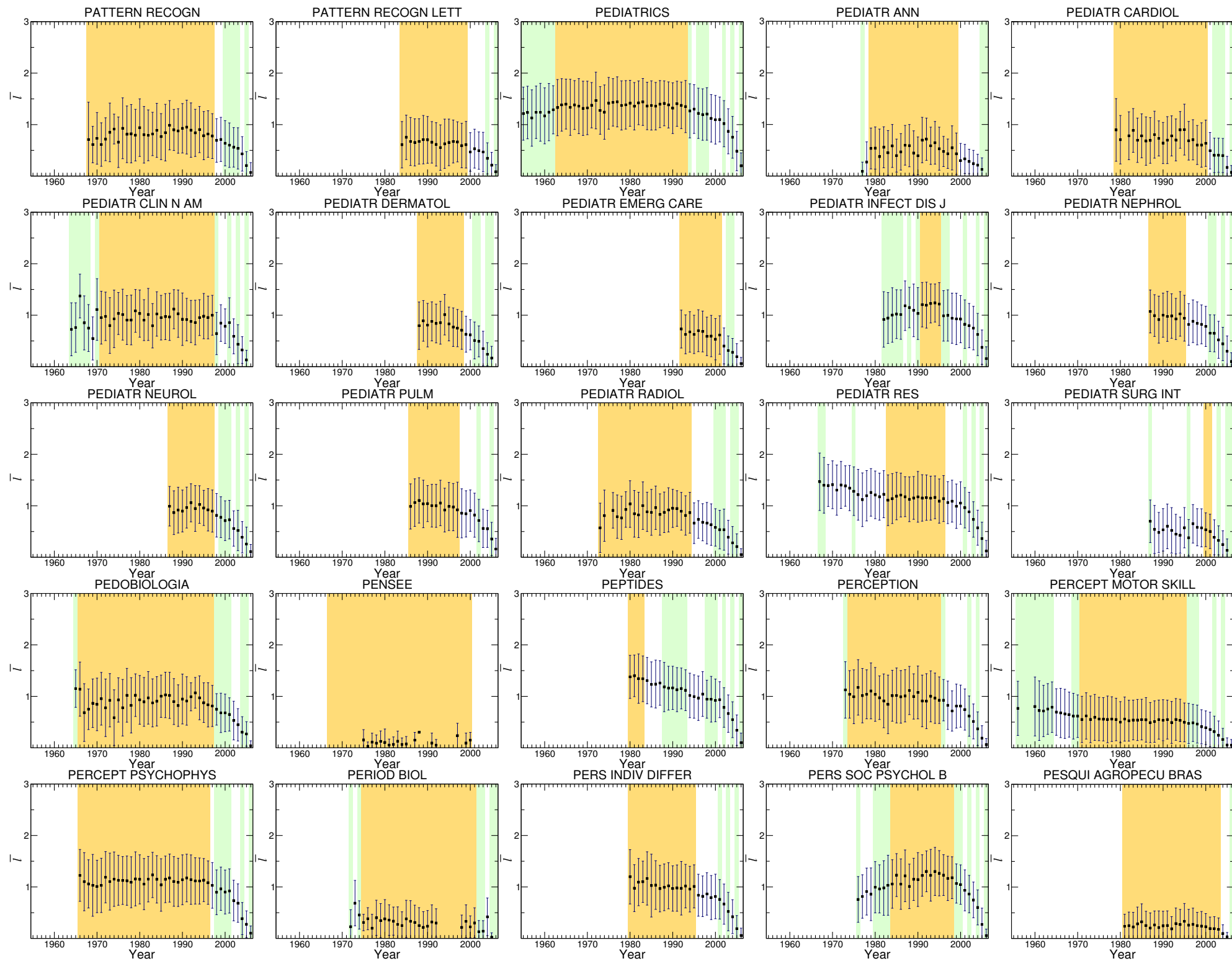

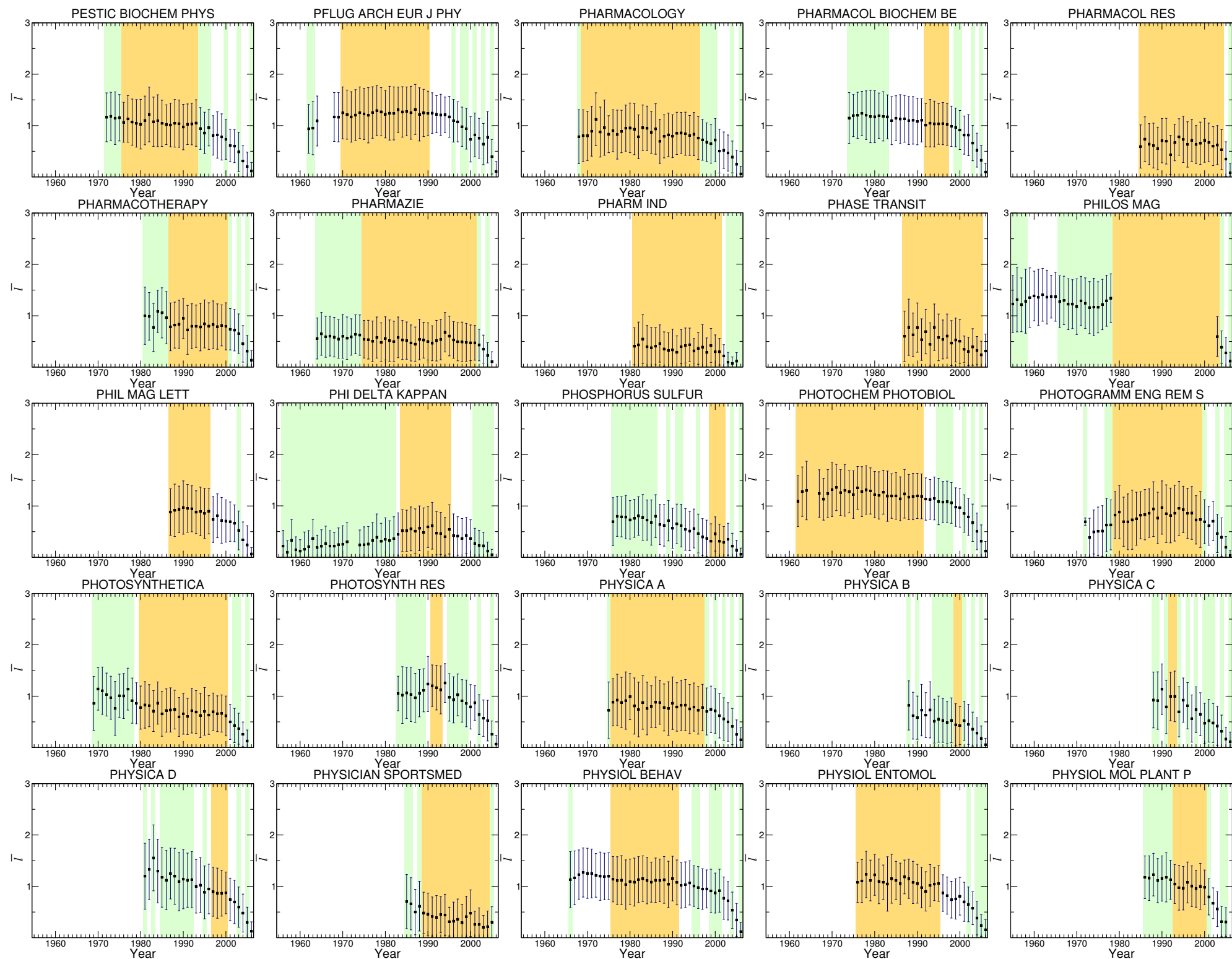

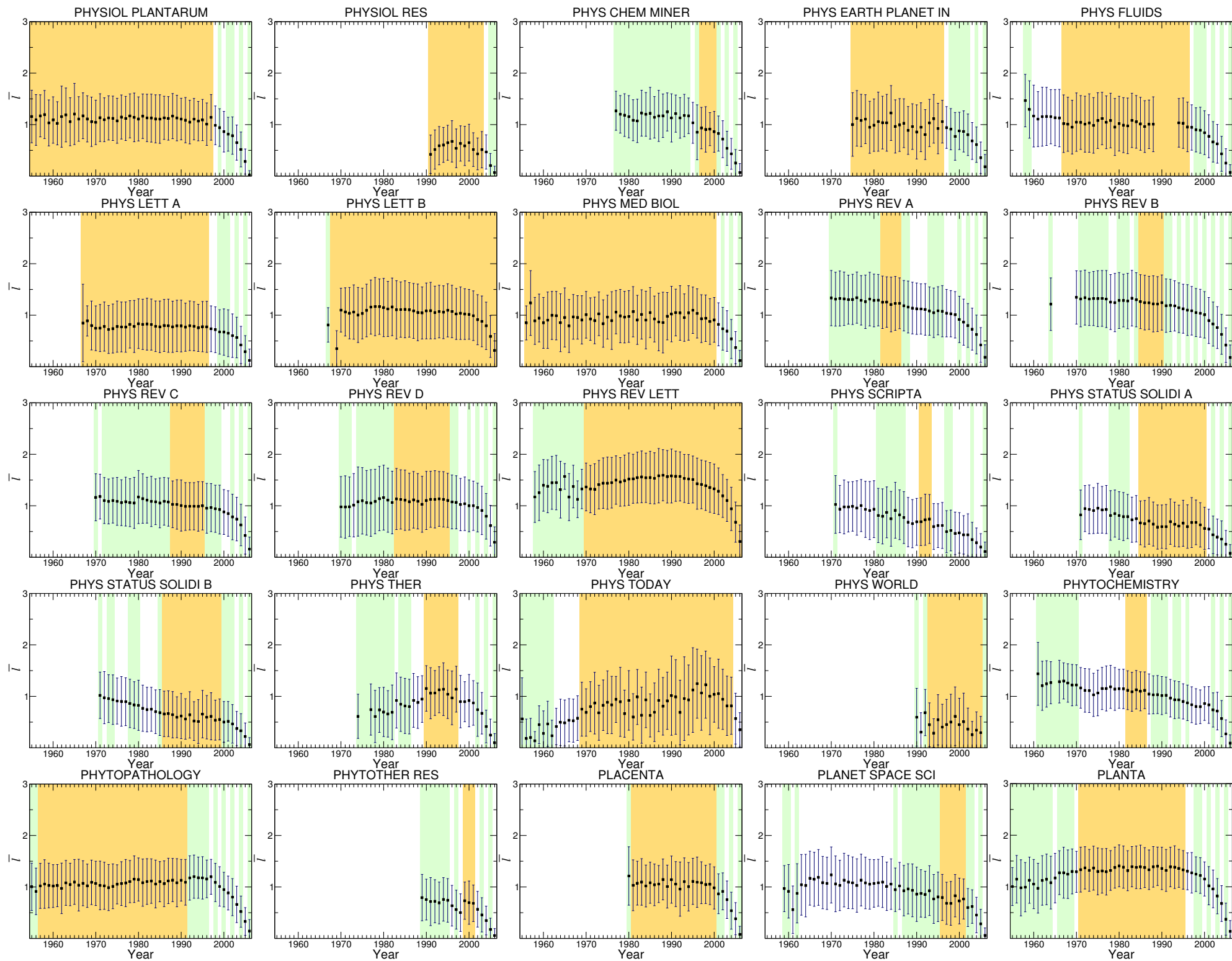

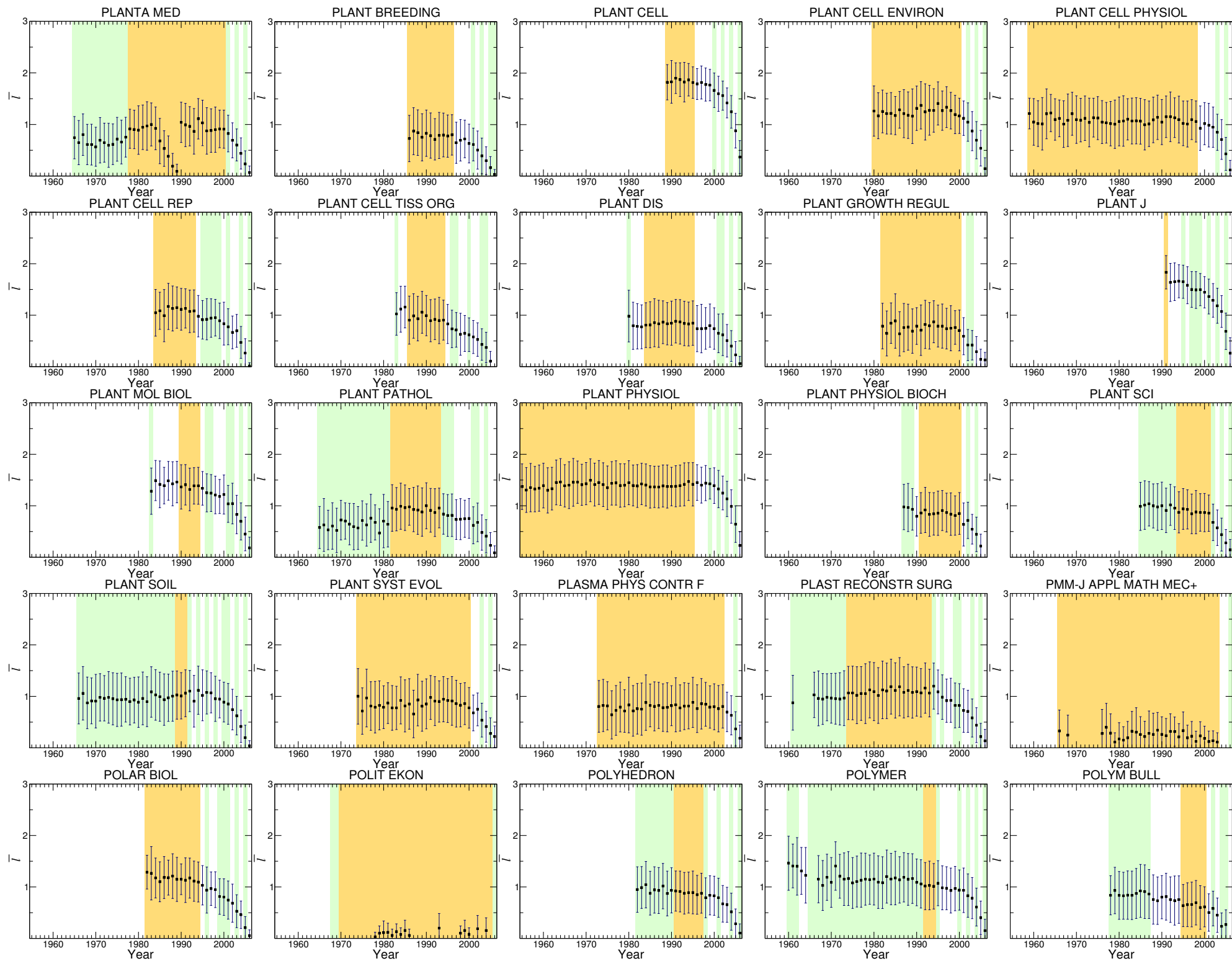

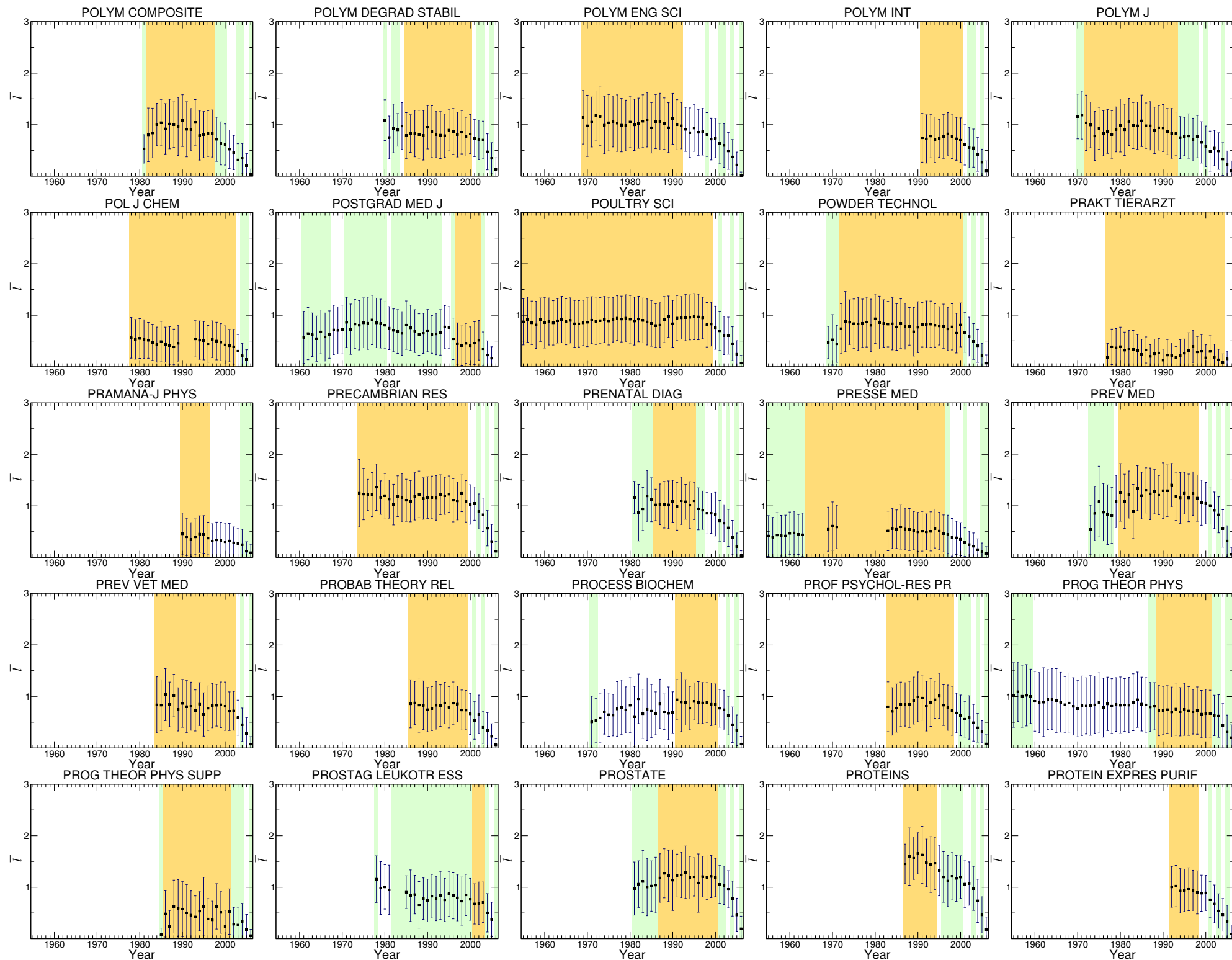

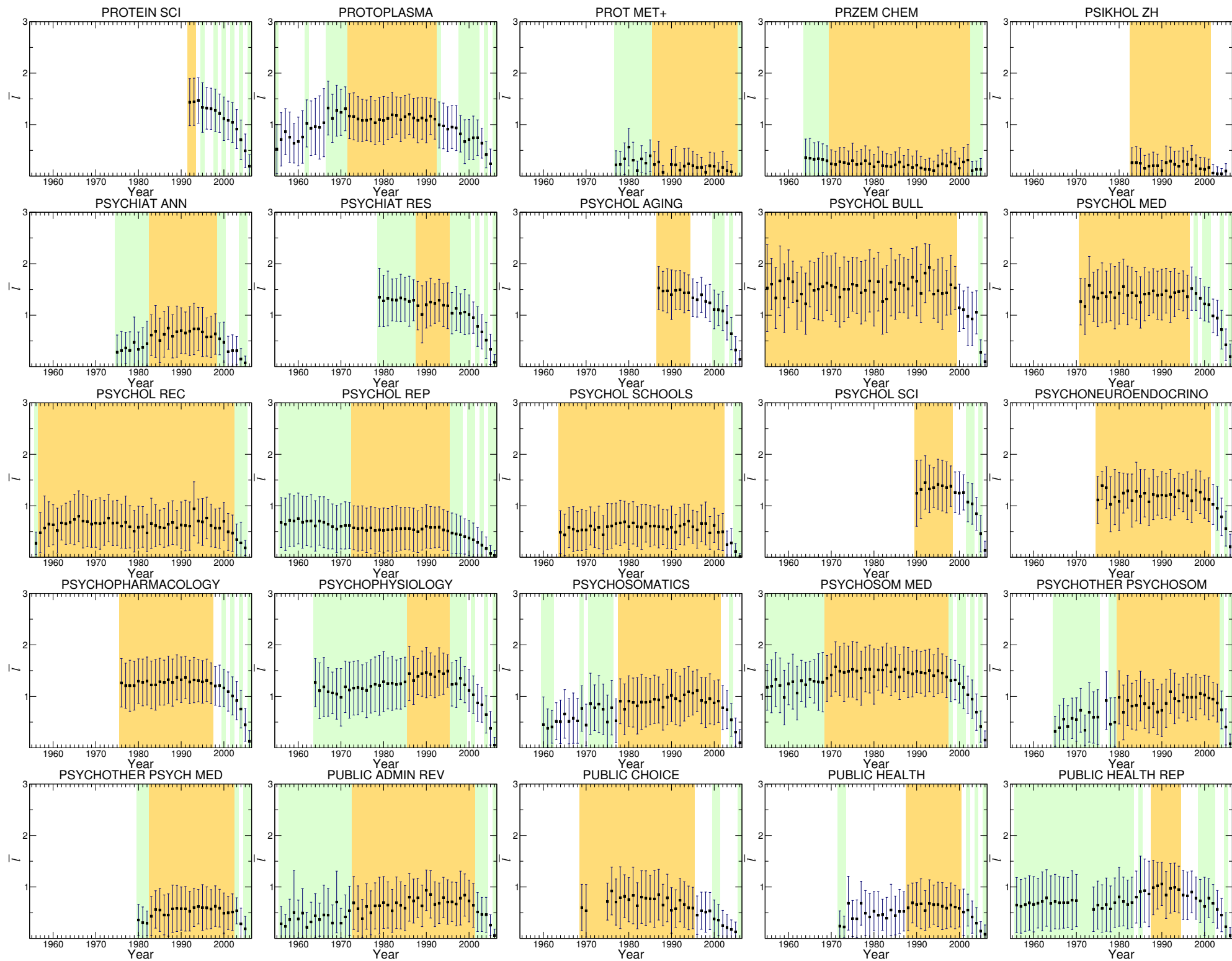

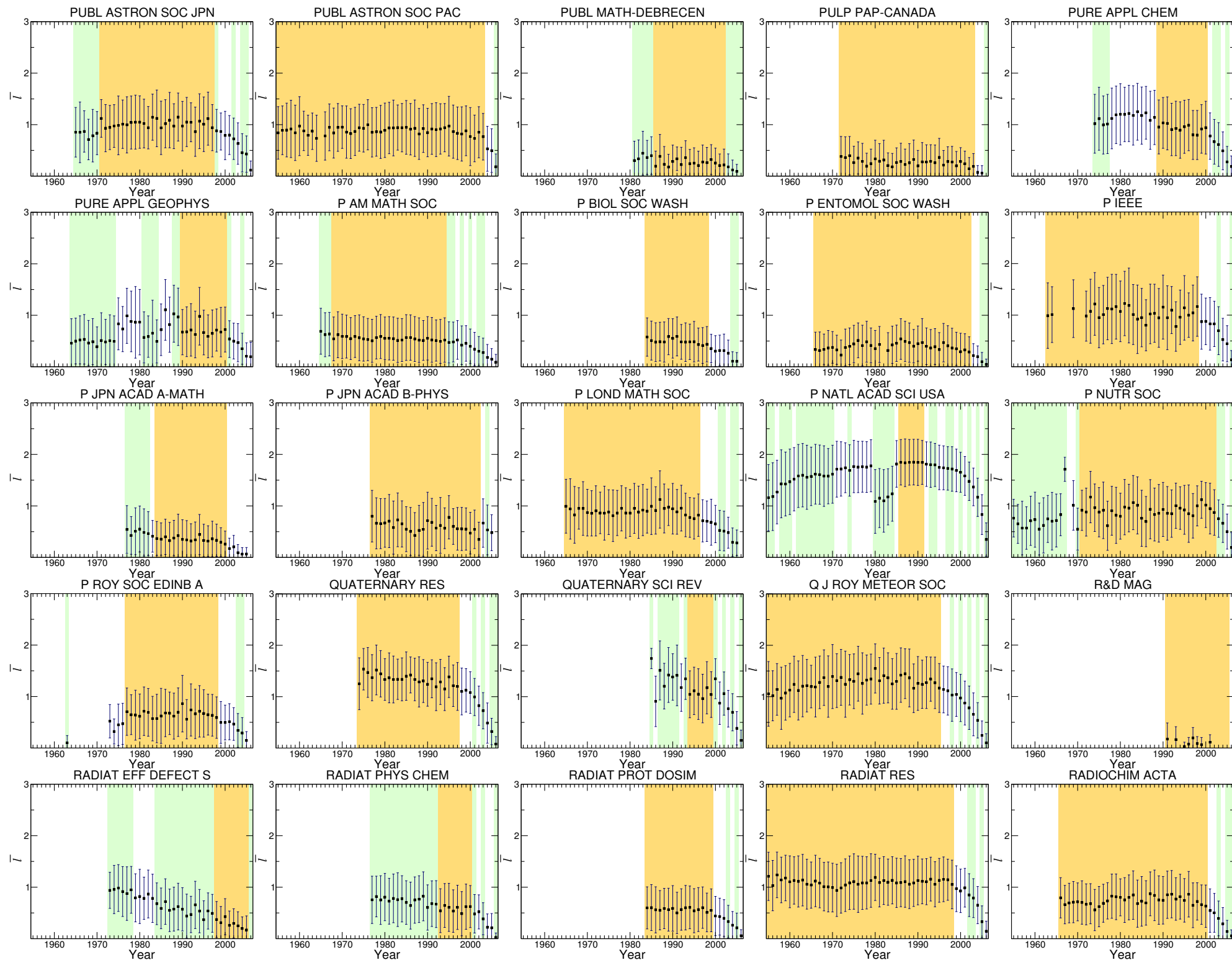

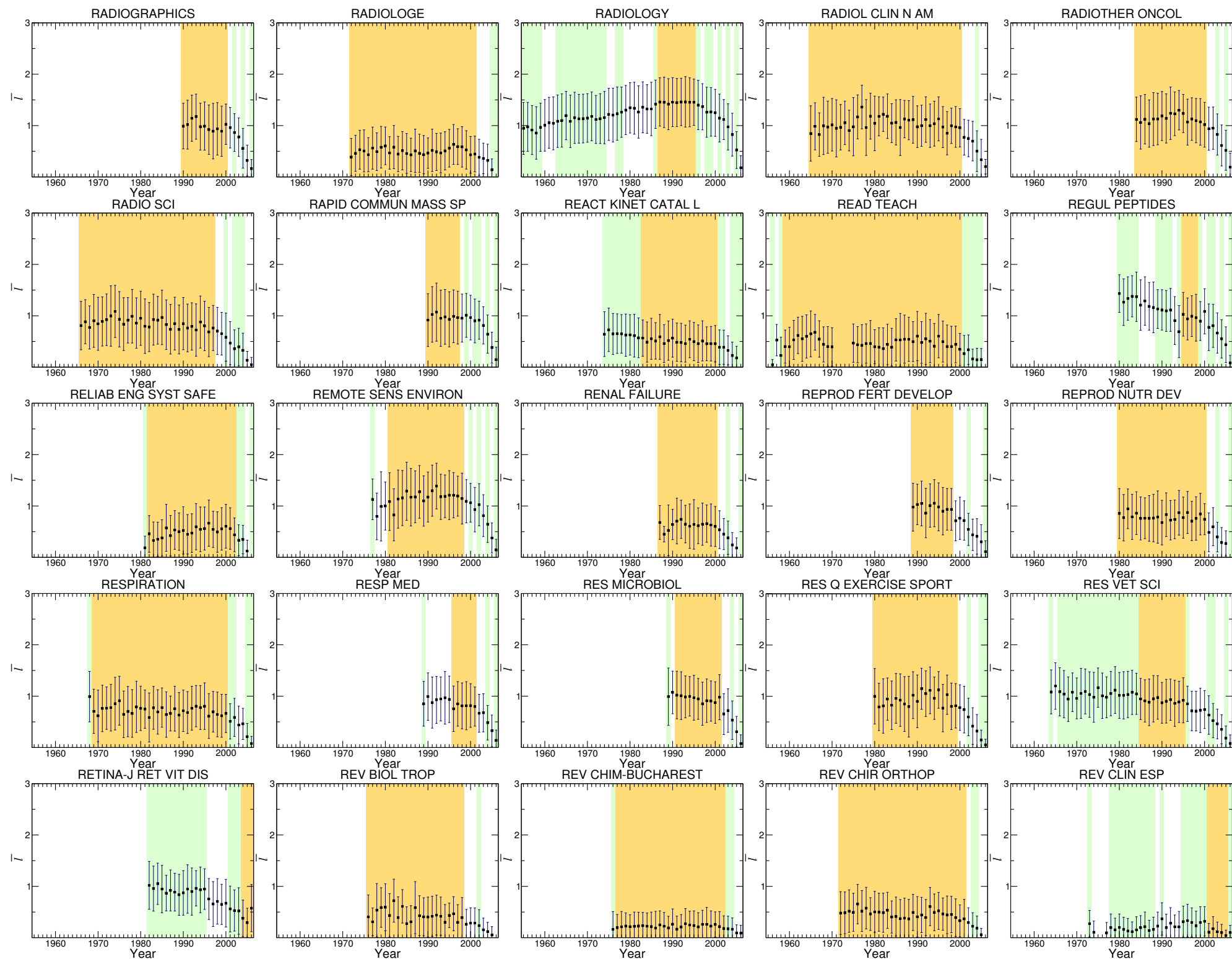

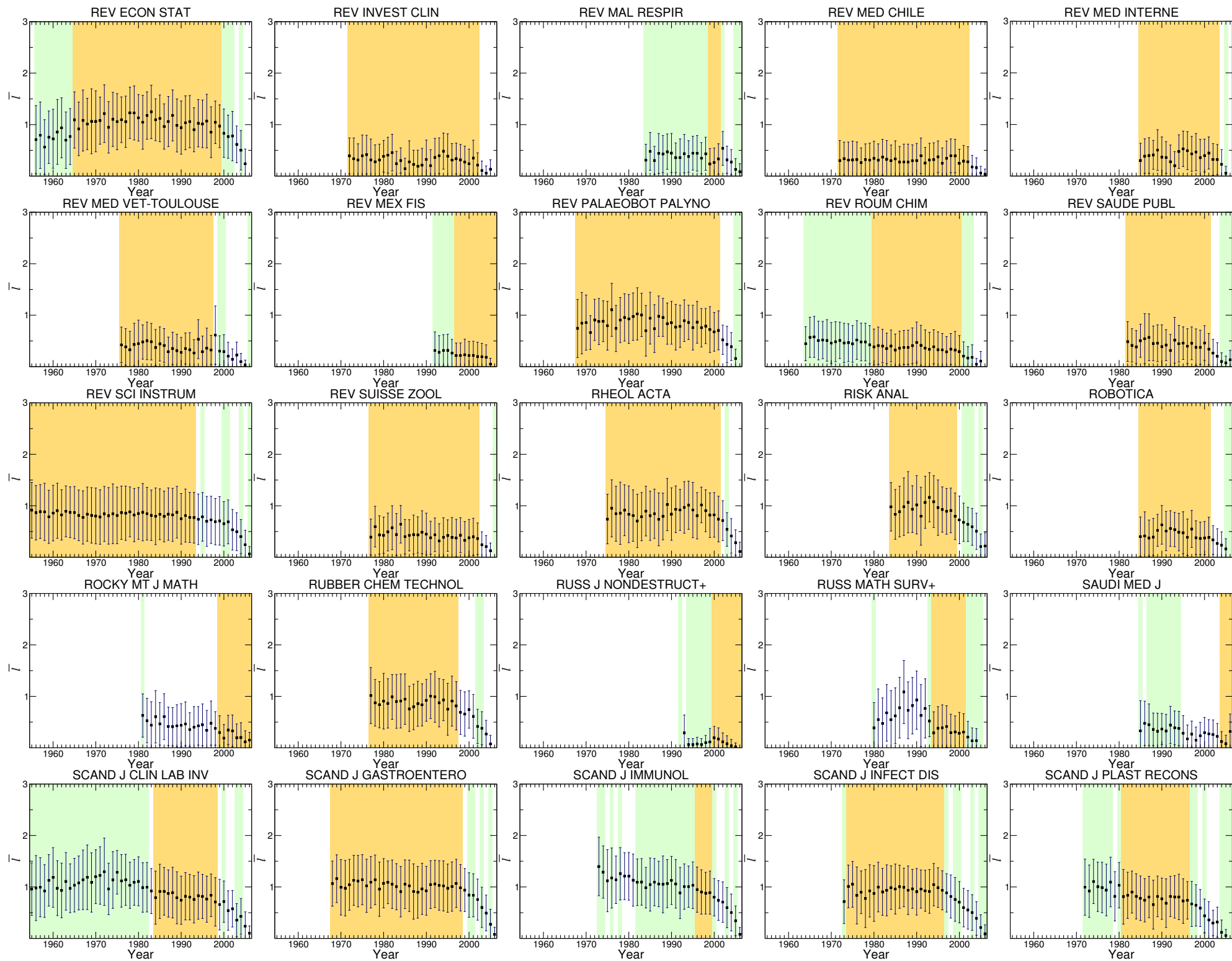

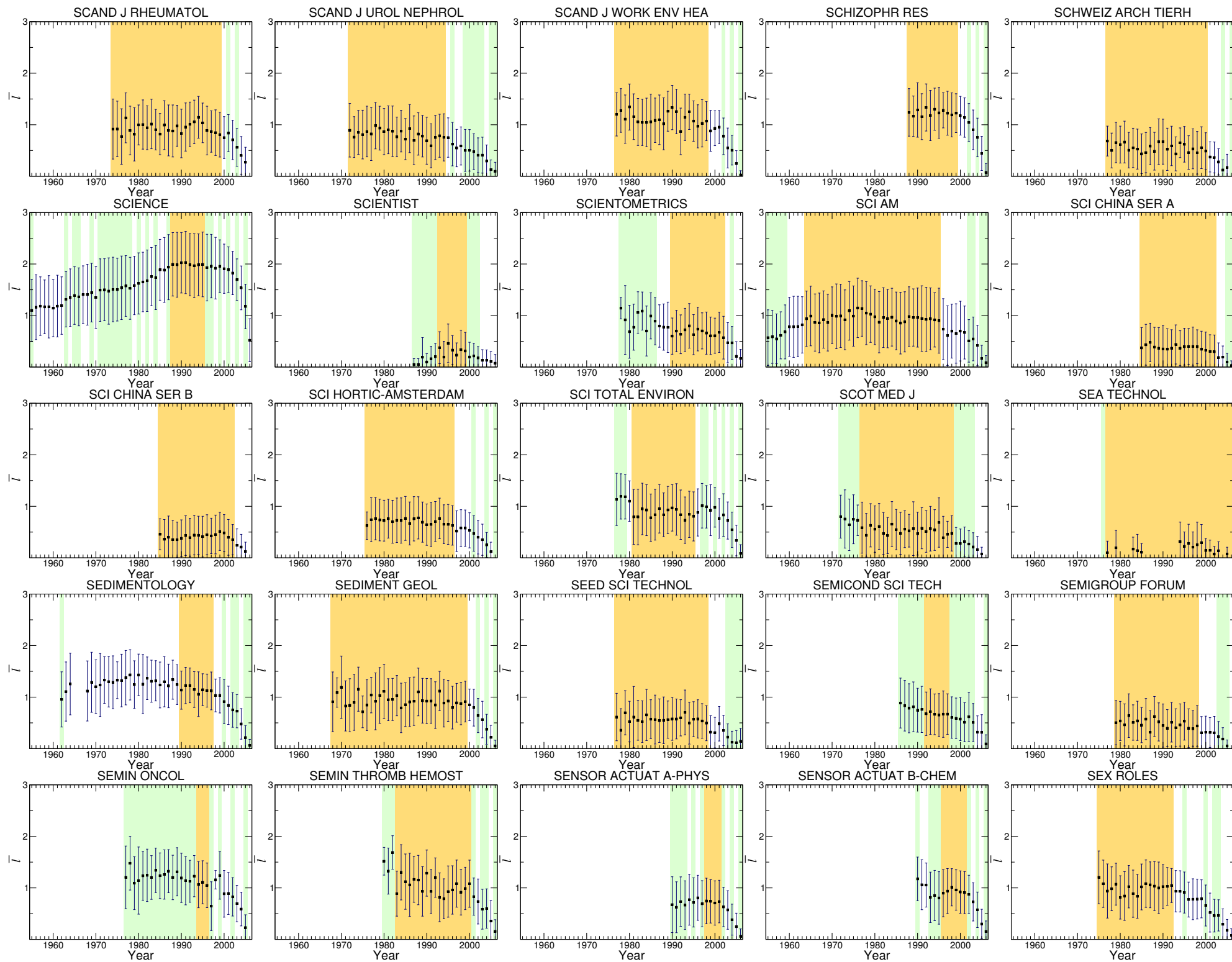

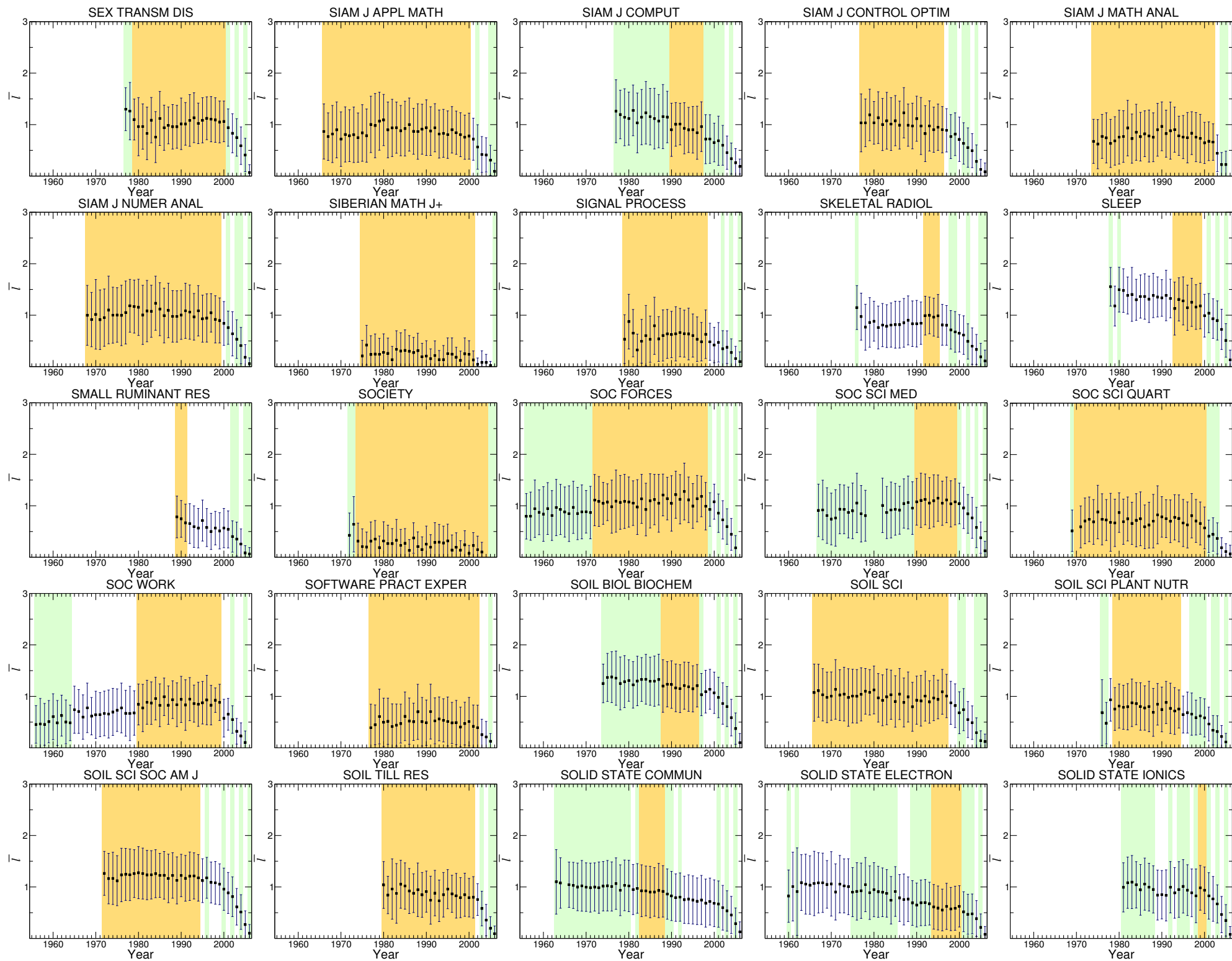

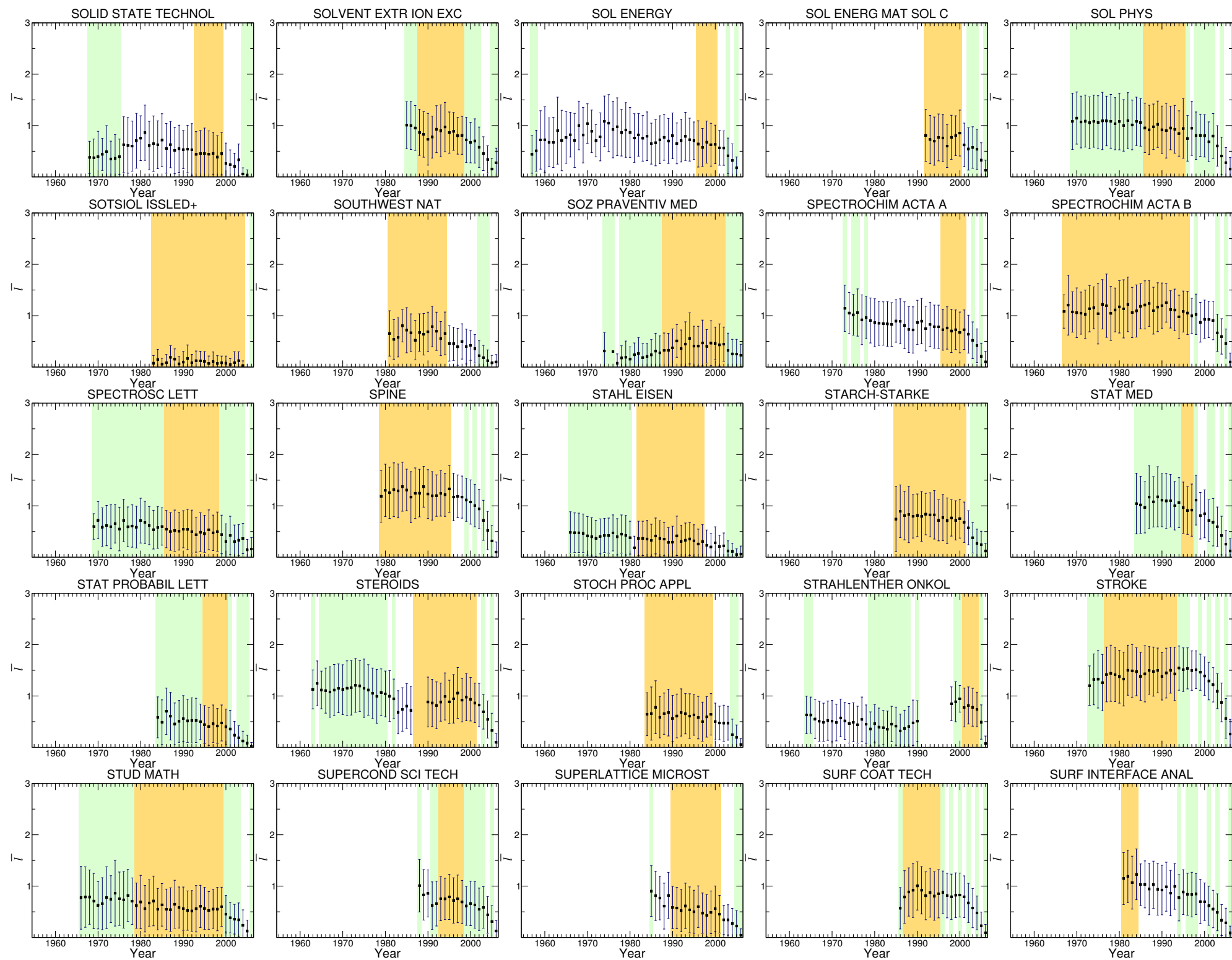

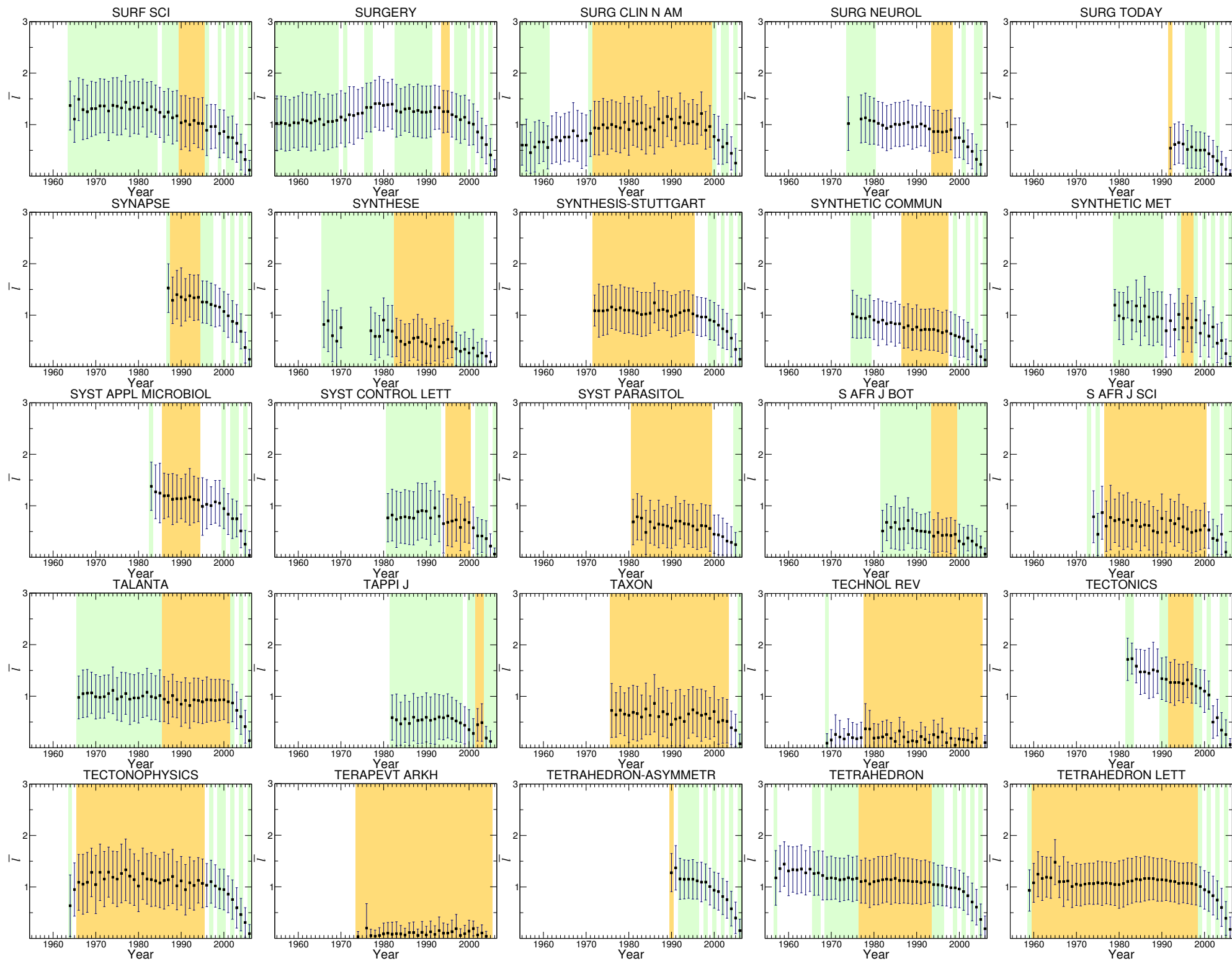

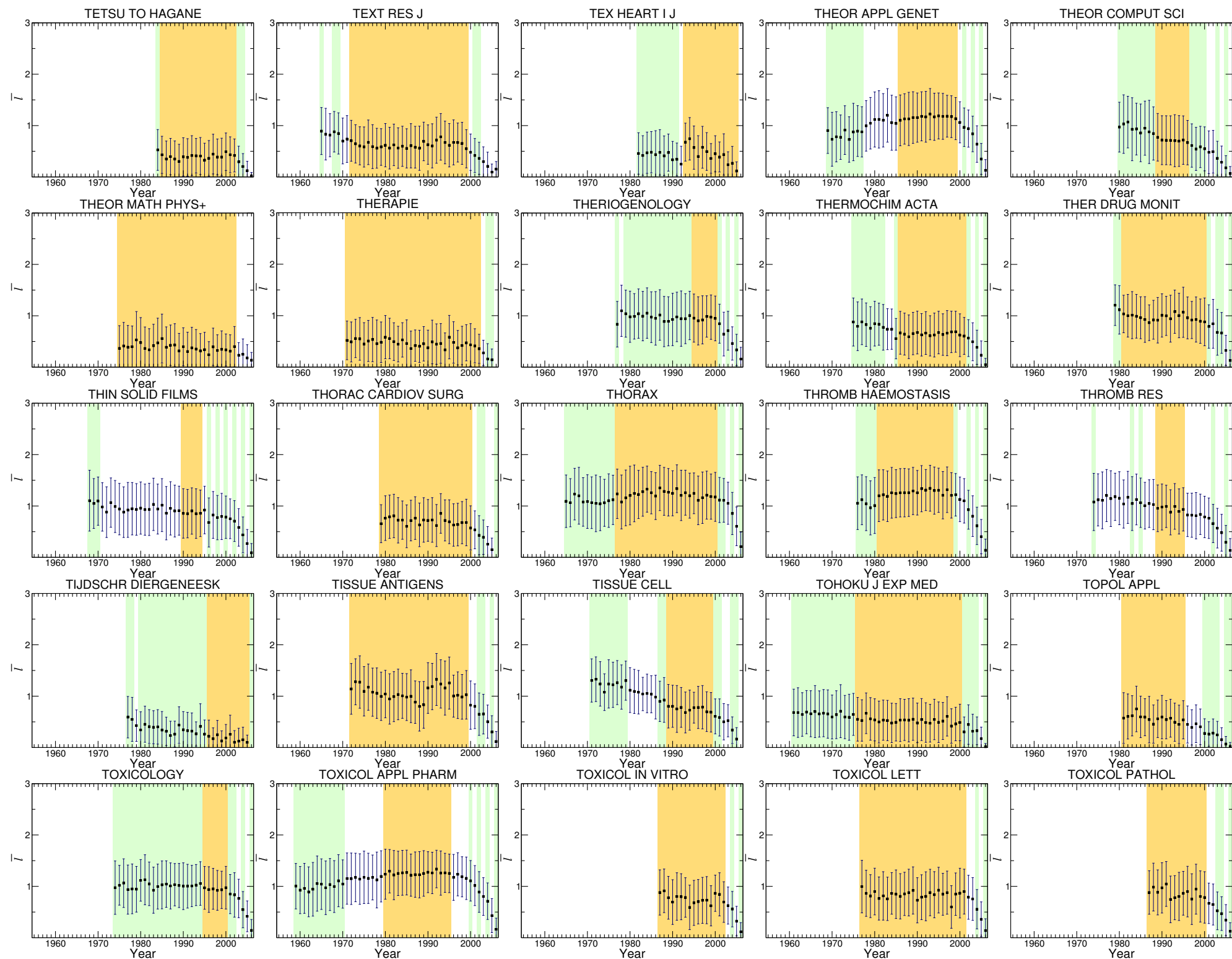

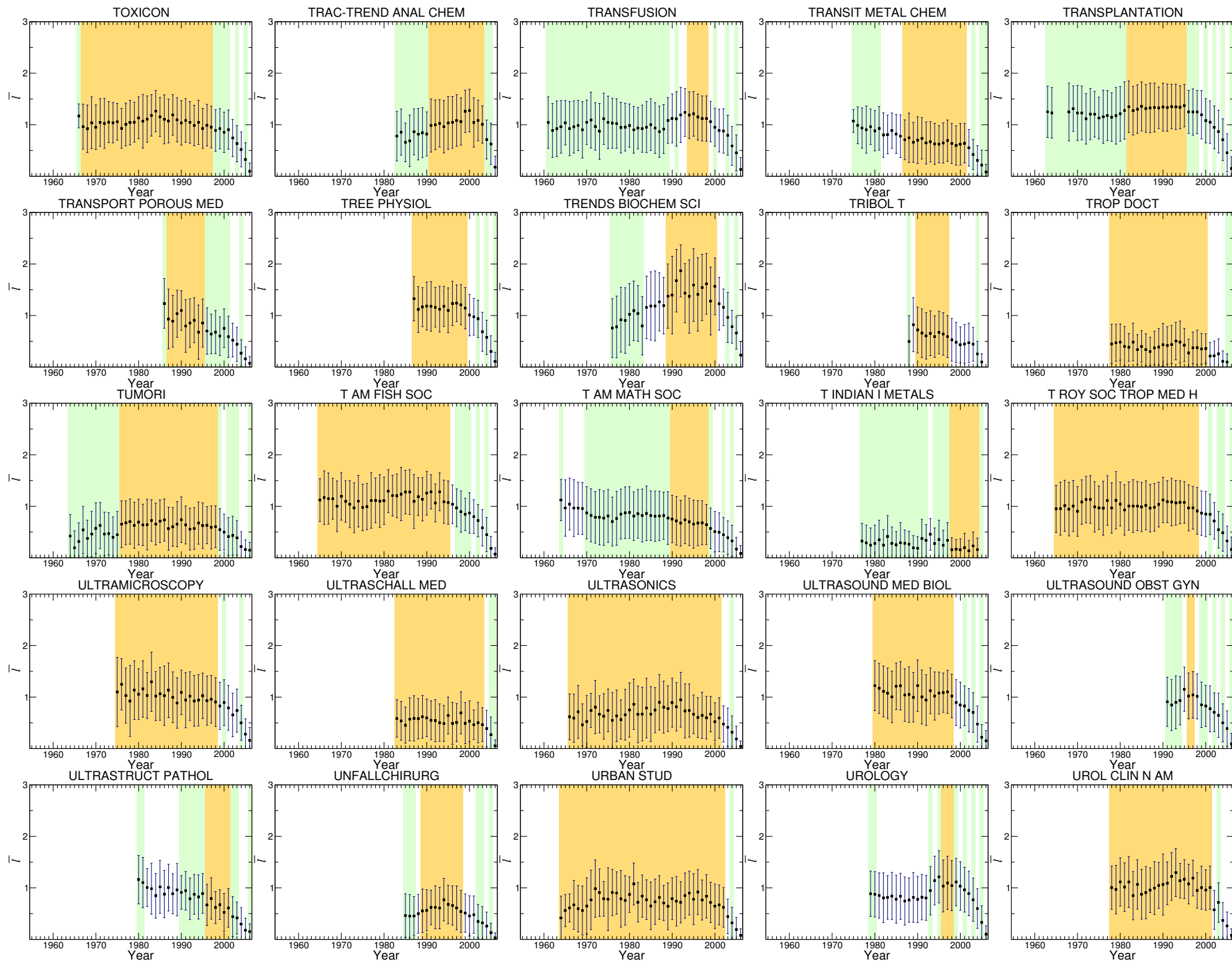

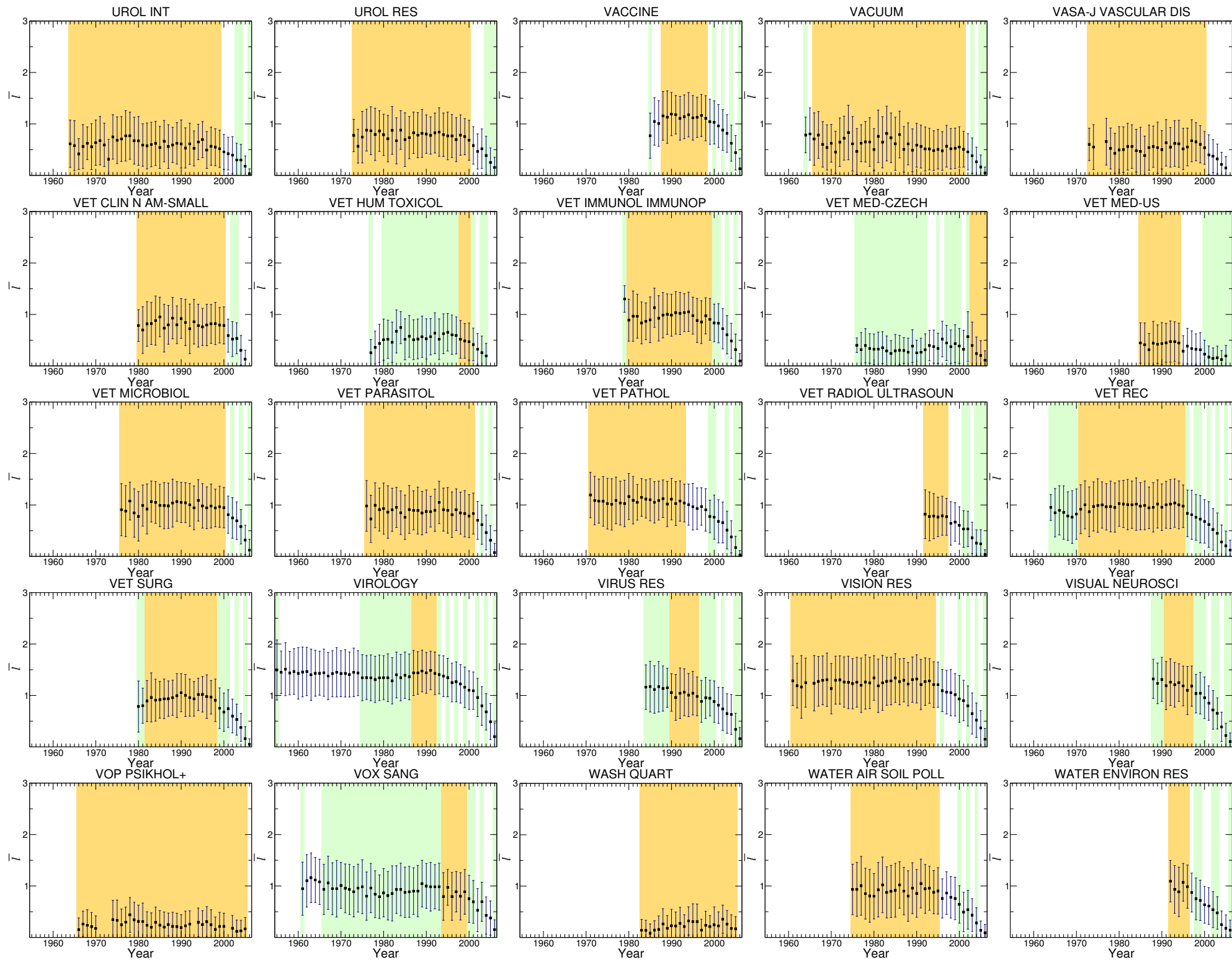

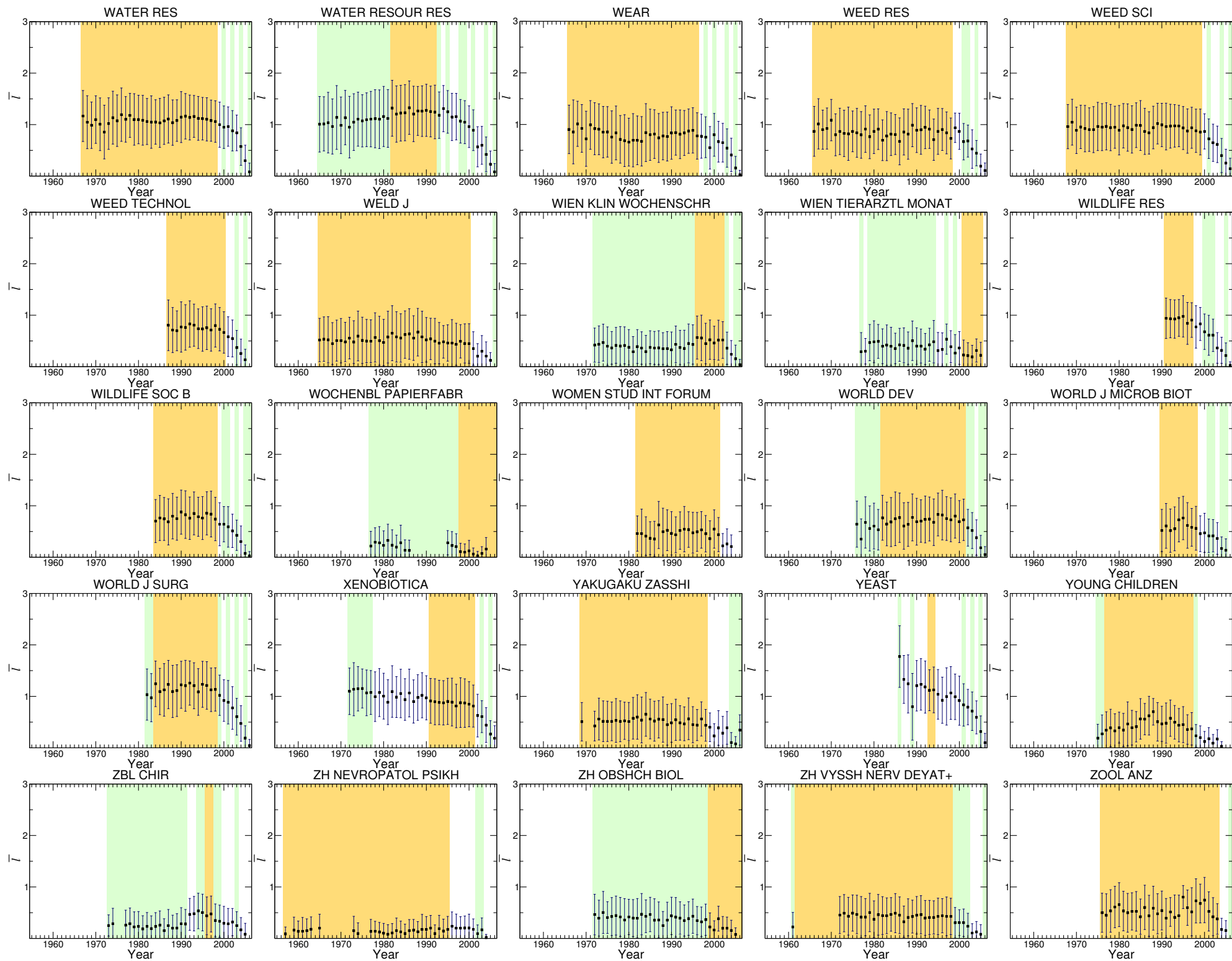

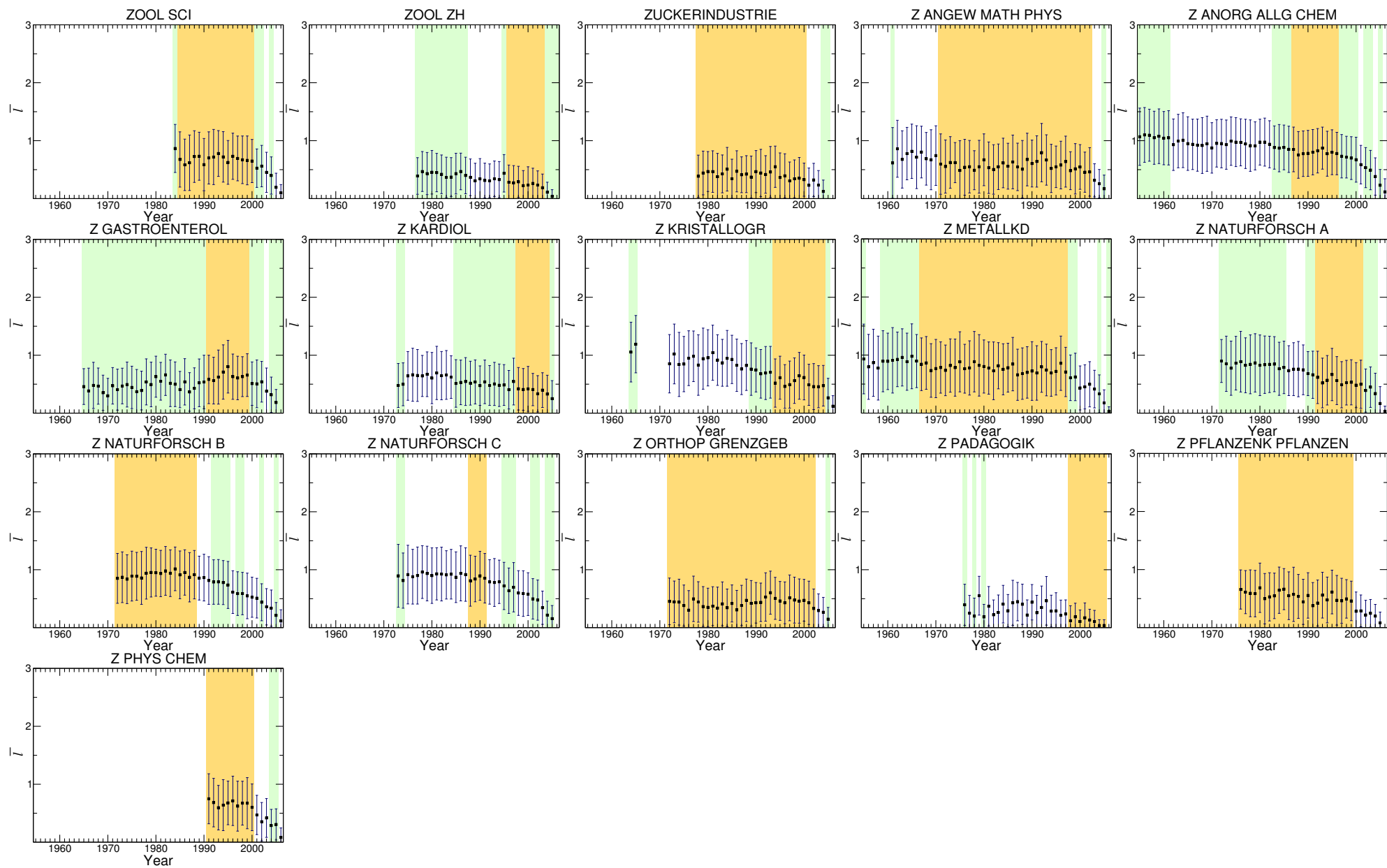

Supplement: Appendix S2 — Citation history for the 2,266 journals included in our analysis in alphabetical order. For a detailed description of the plots see the caption of panel C in Figure 1. (19.10 MB PDF) [file pone.0001683.s002.pdf]

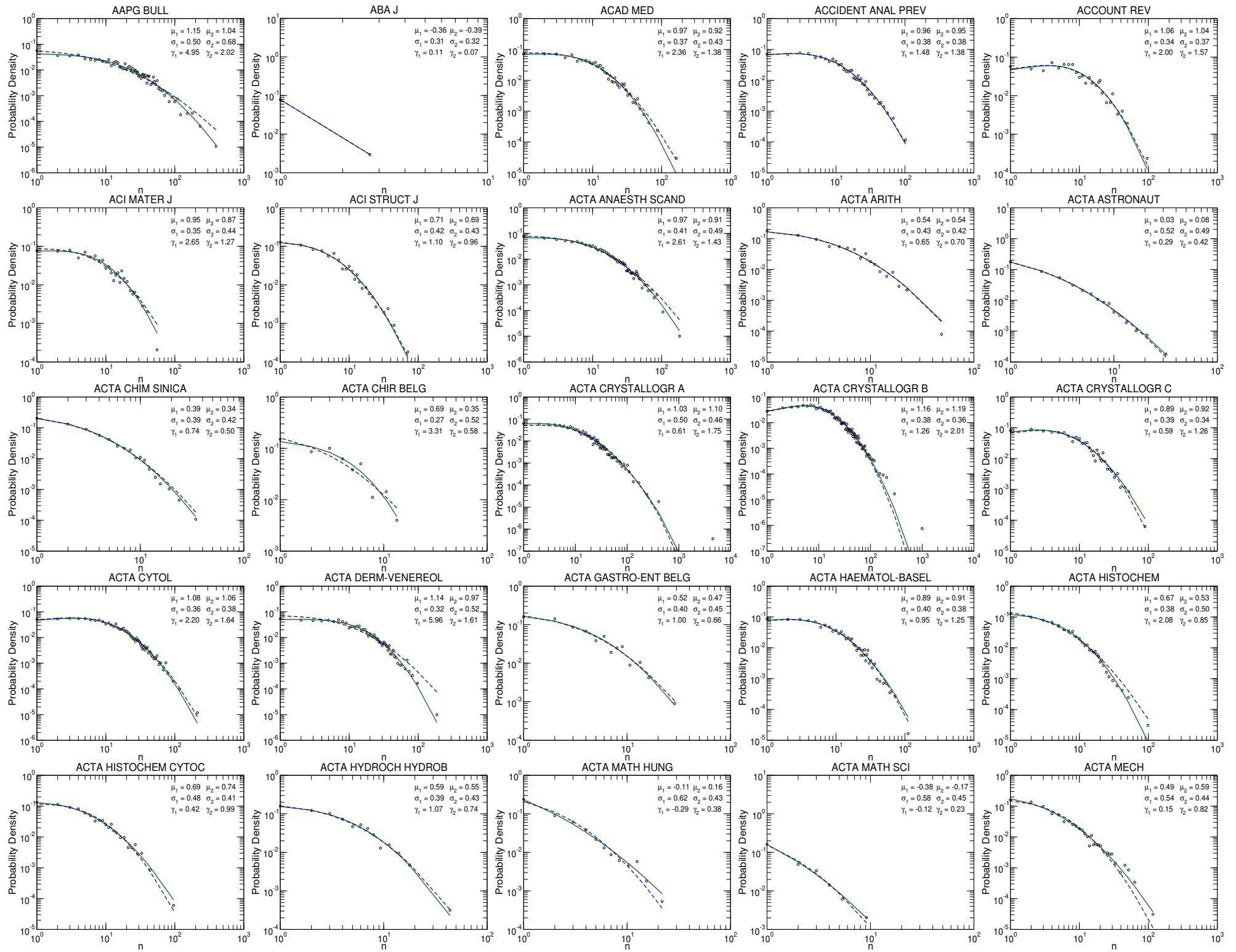

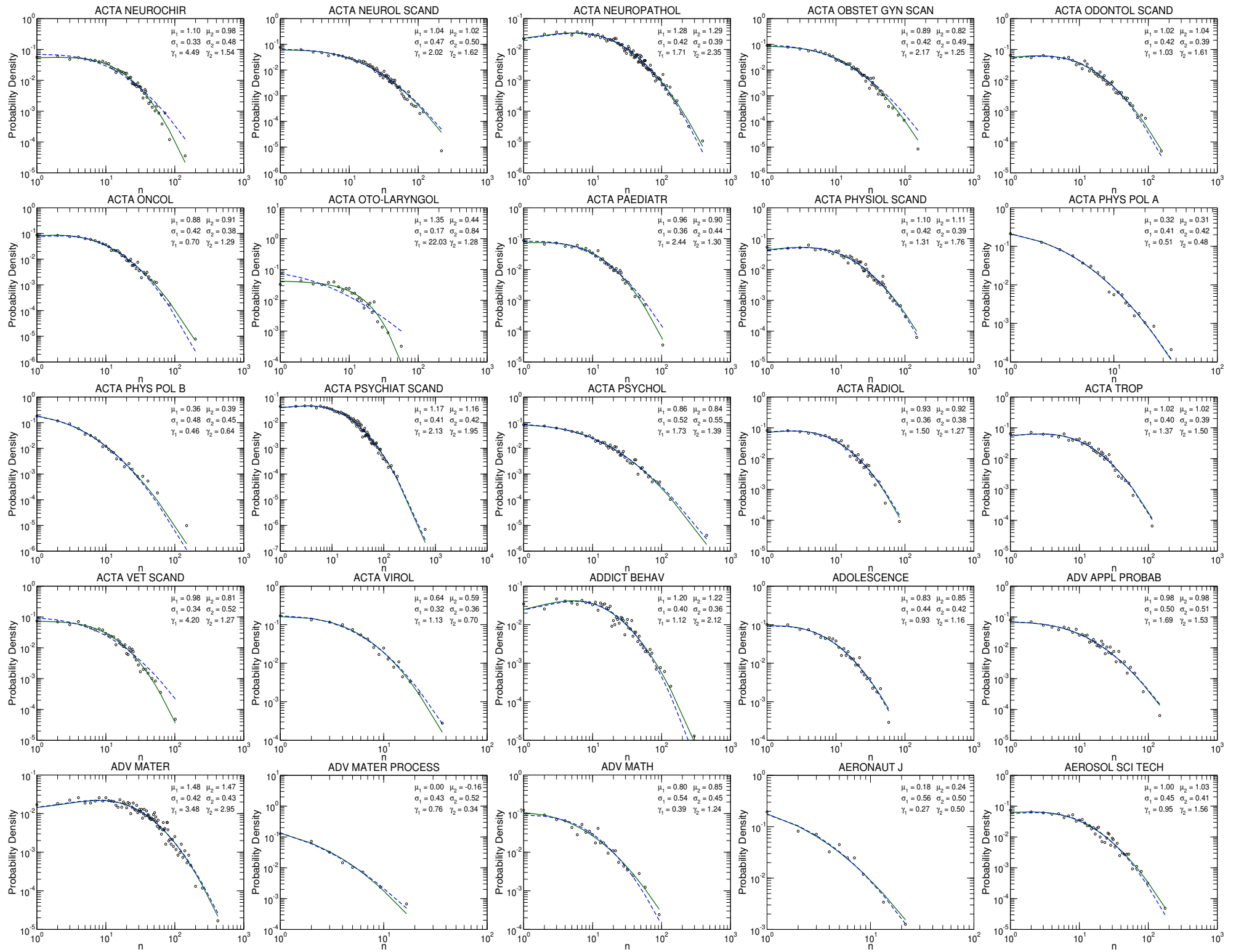

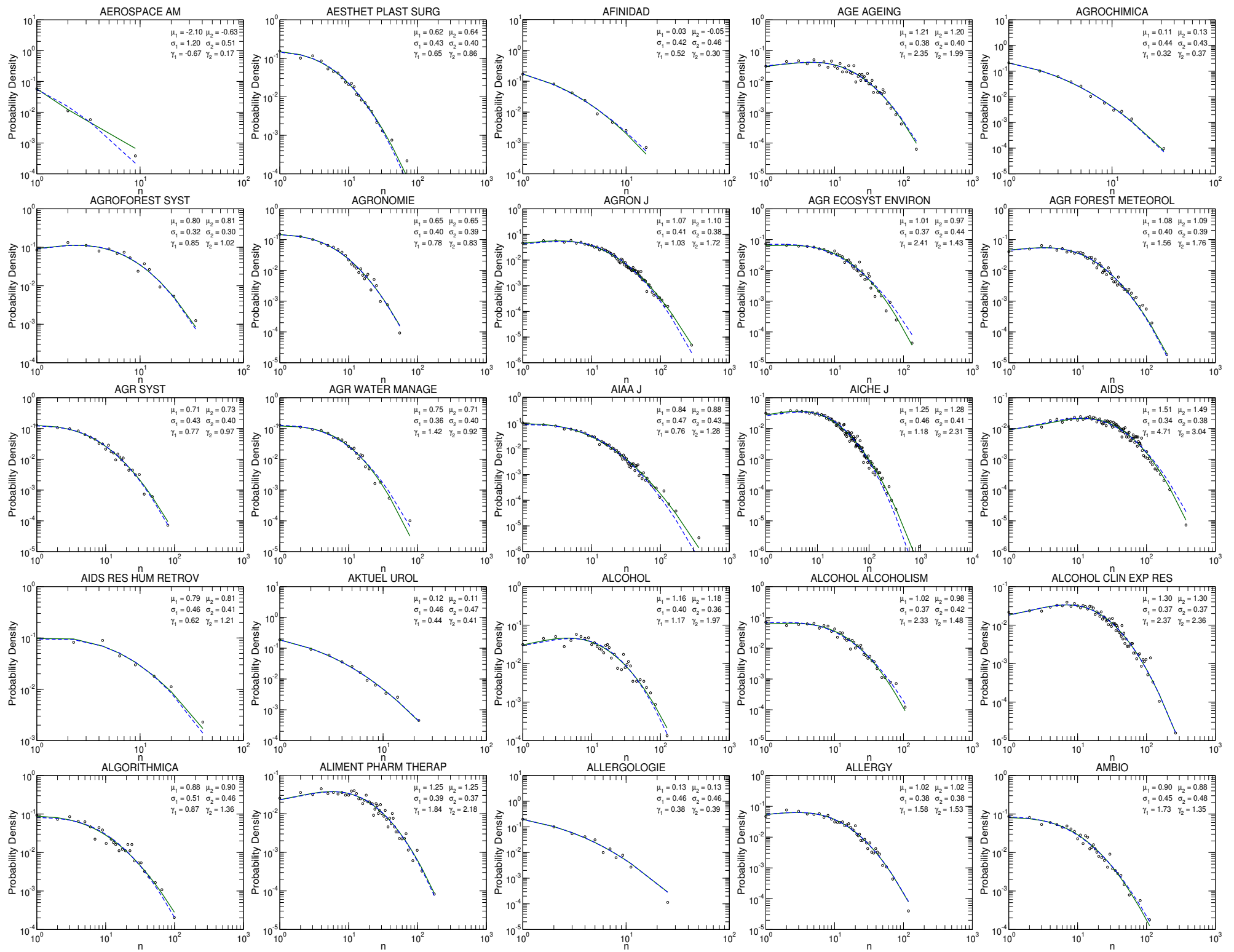

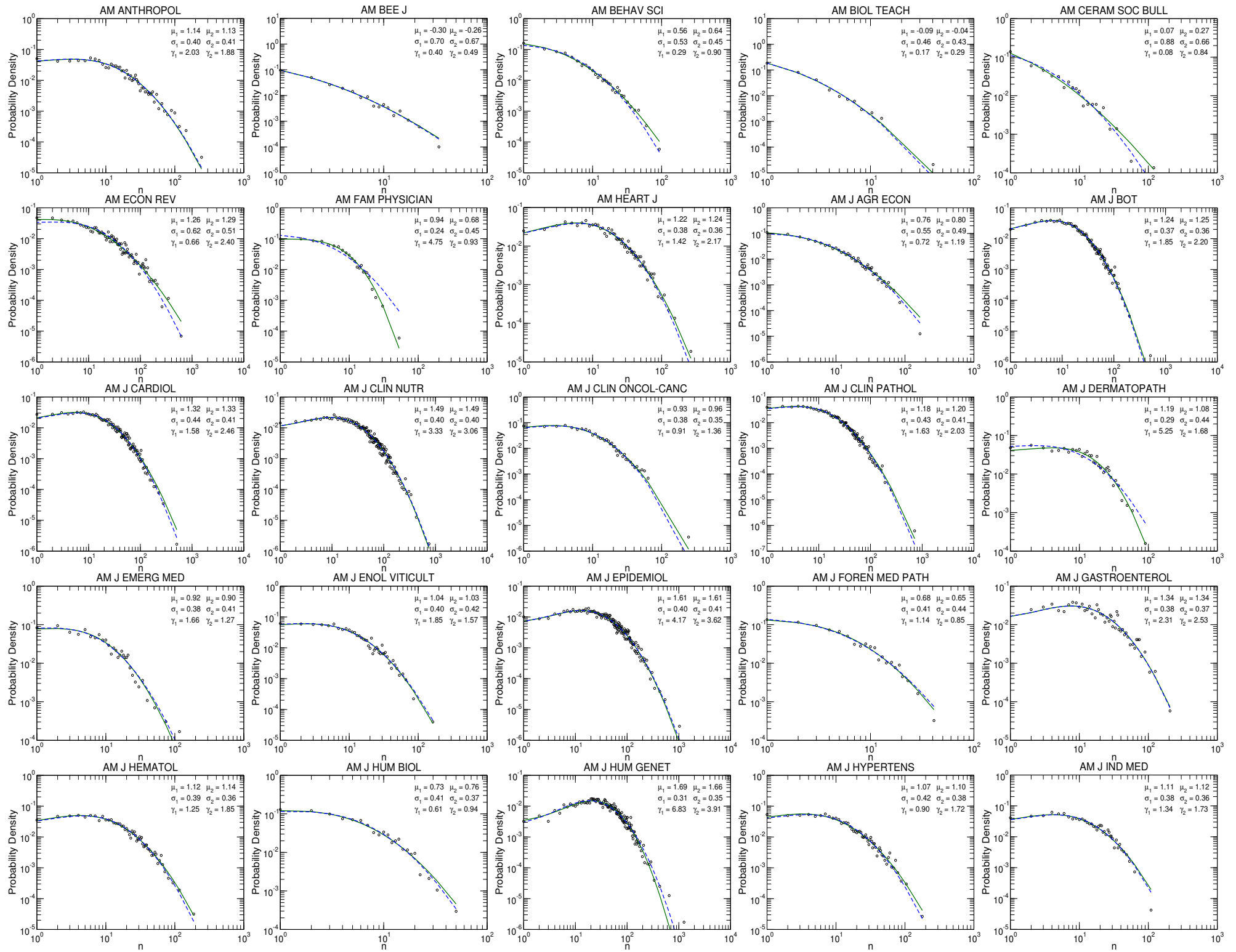

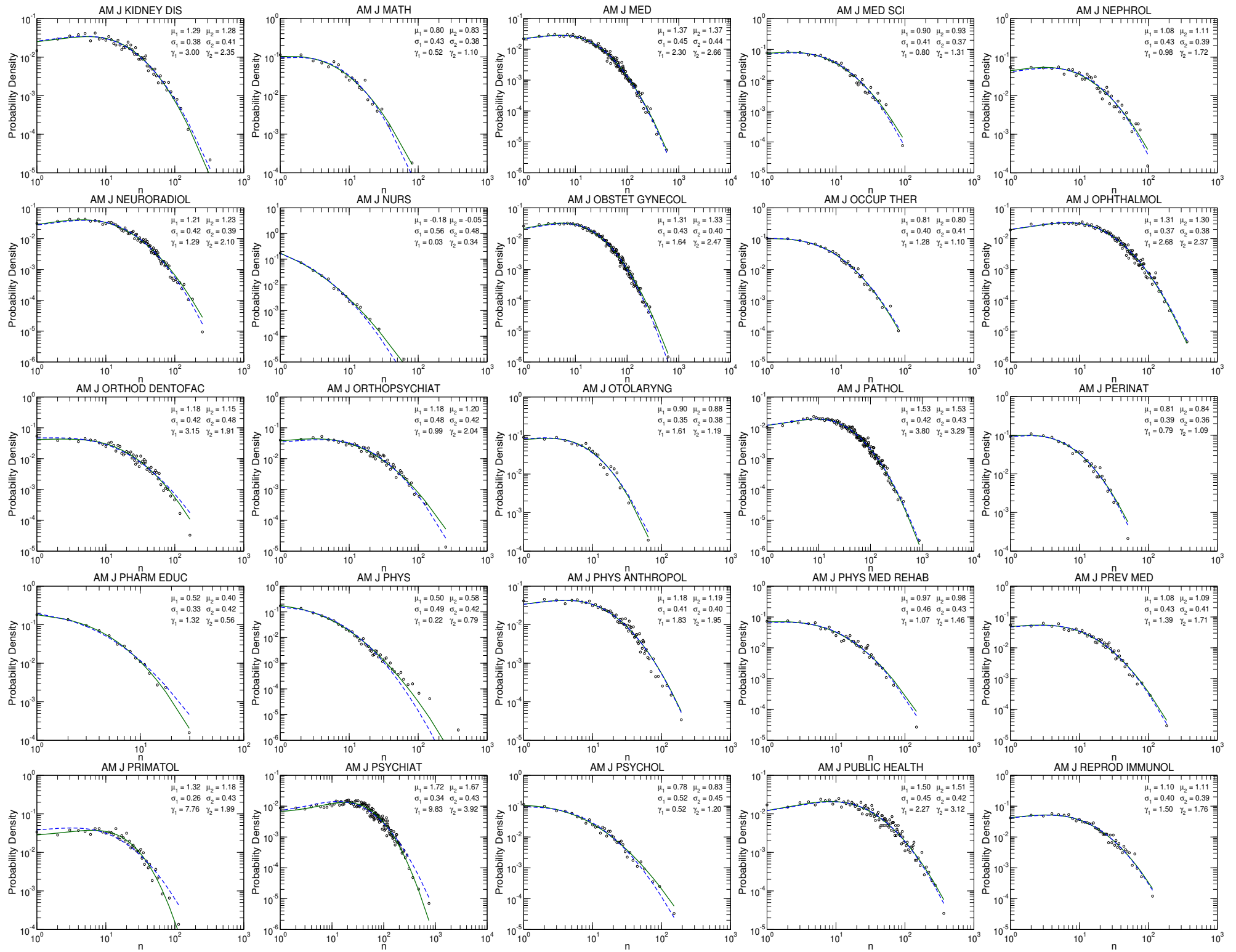

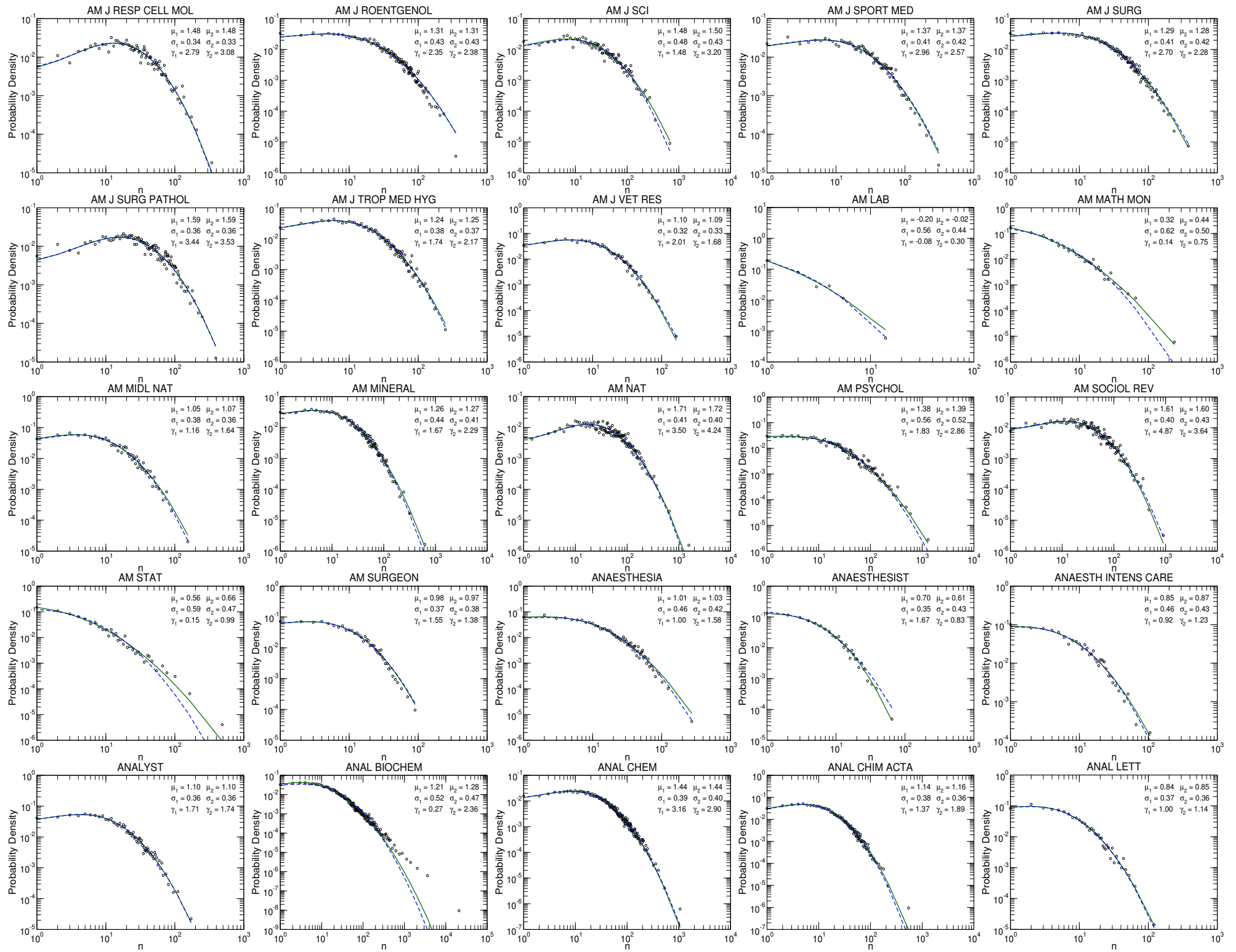

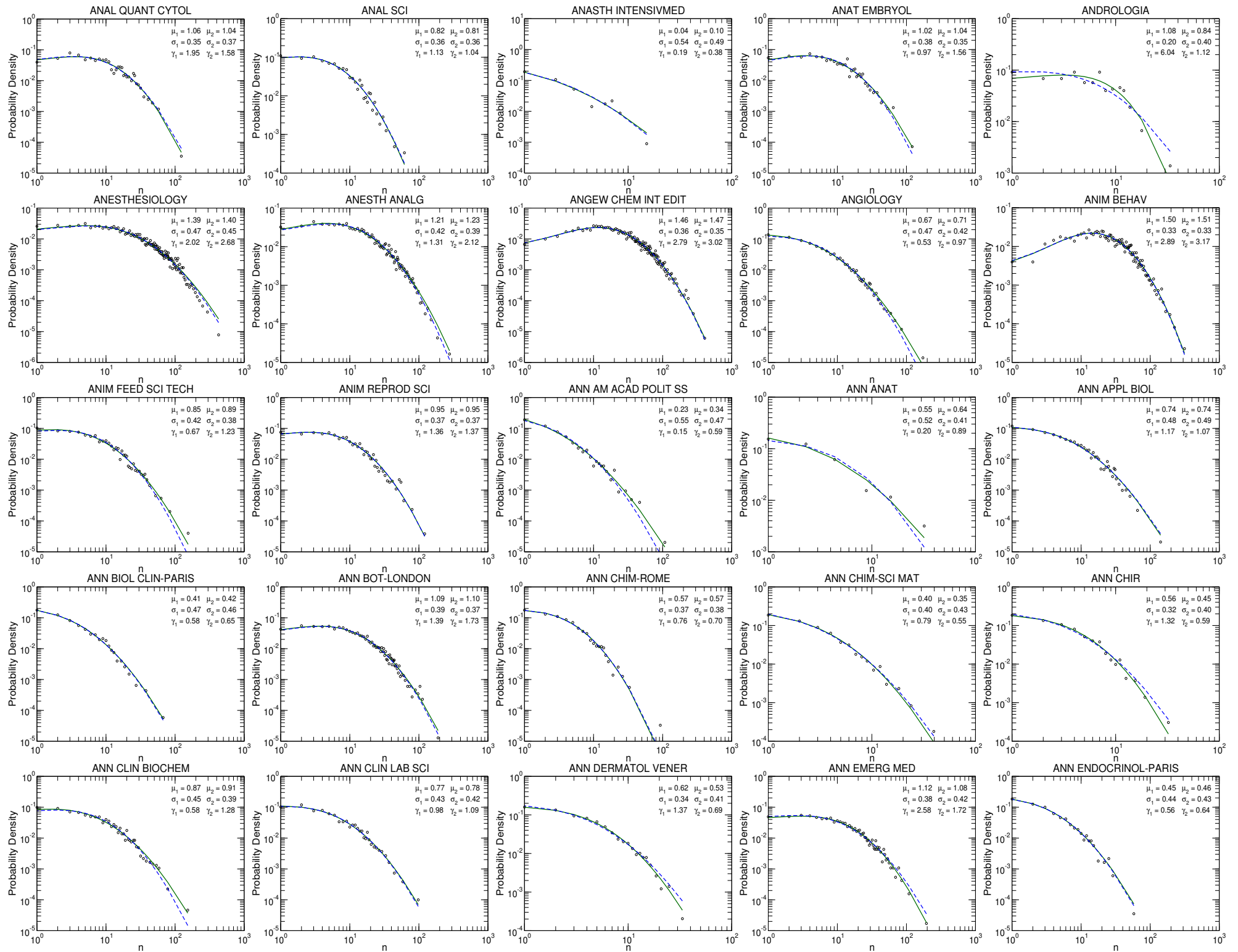

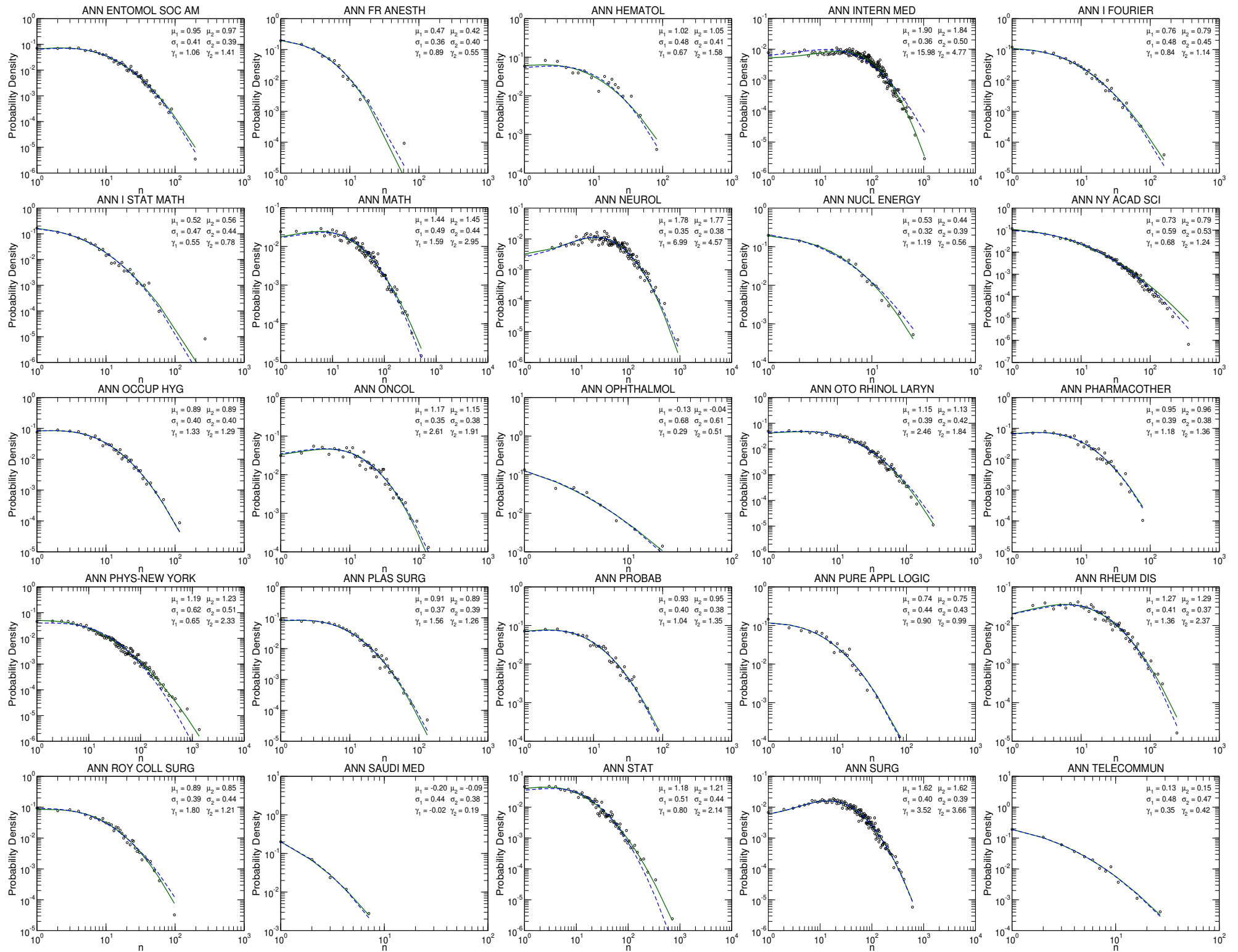

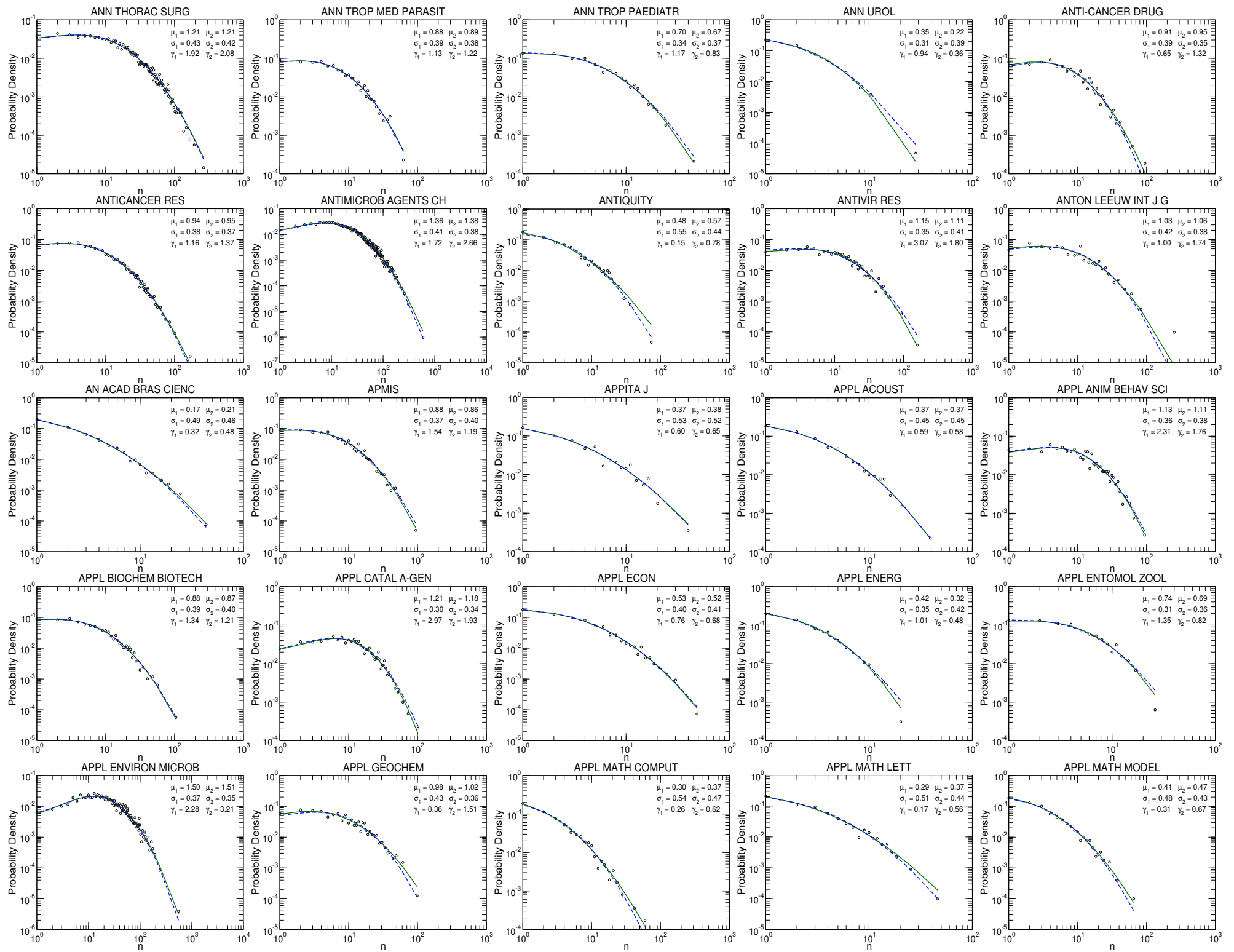

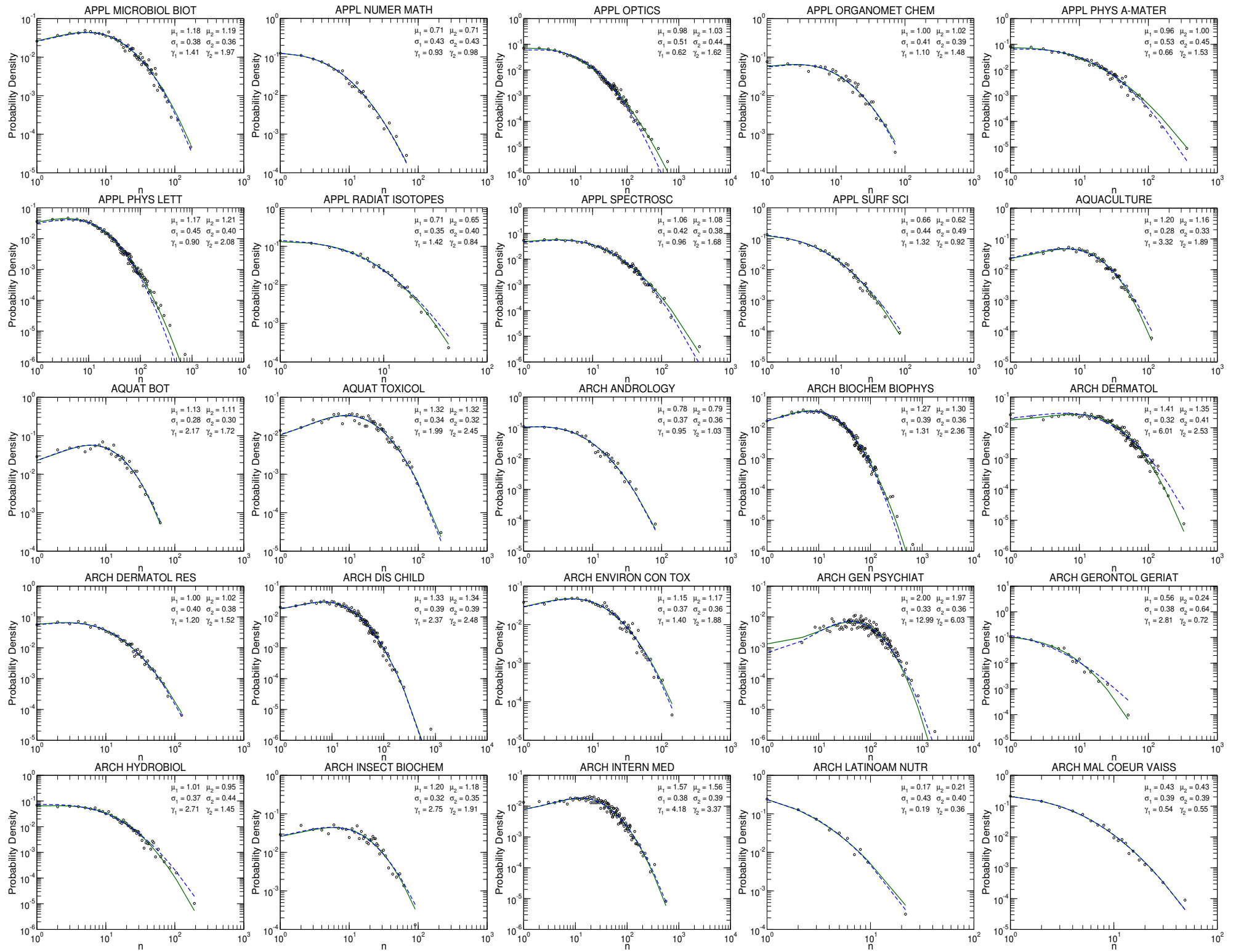

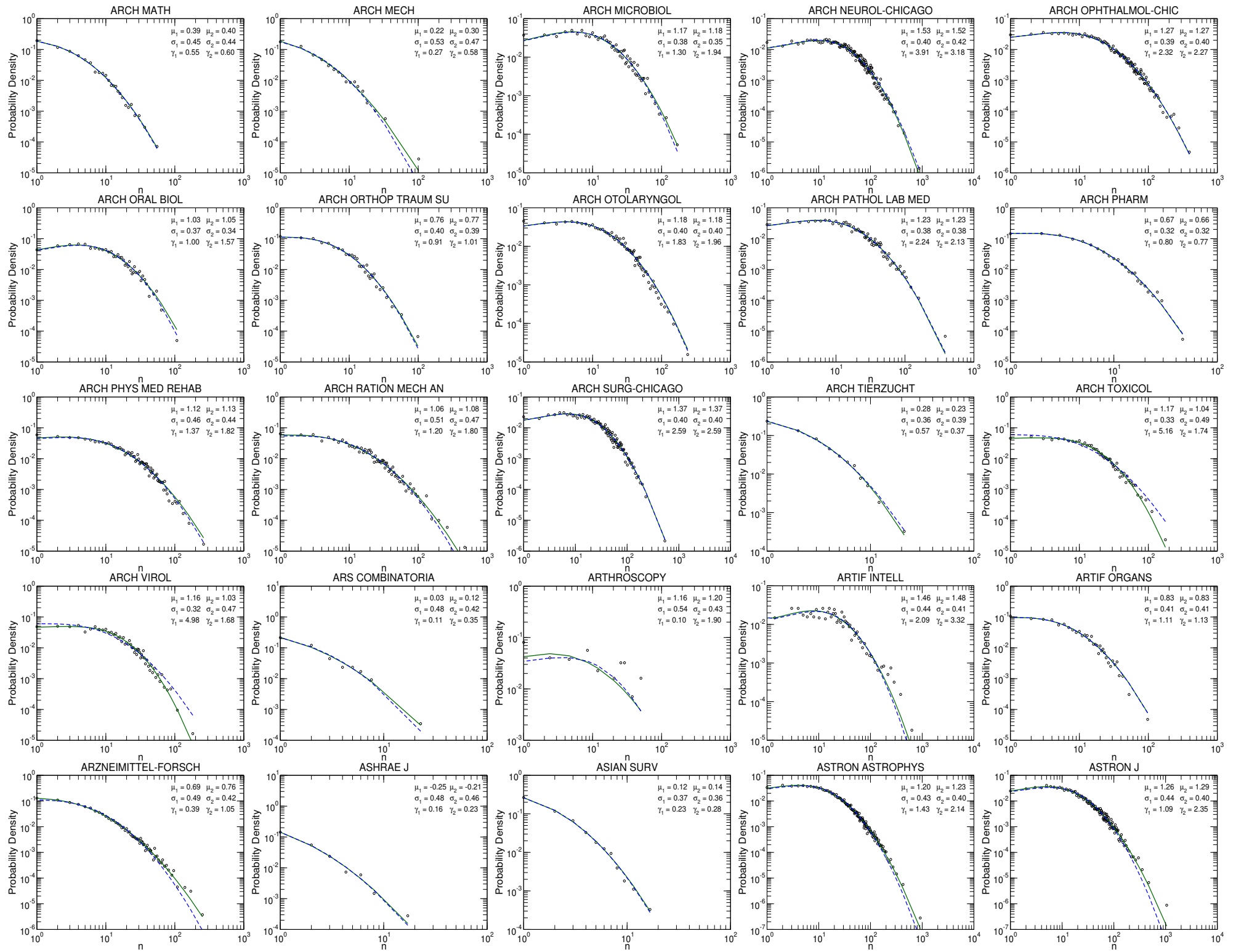

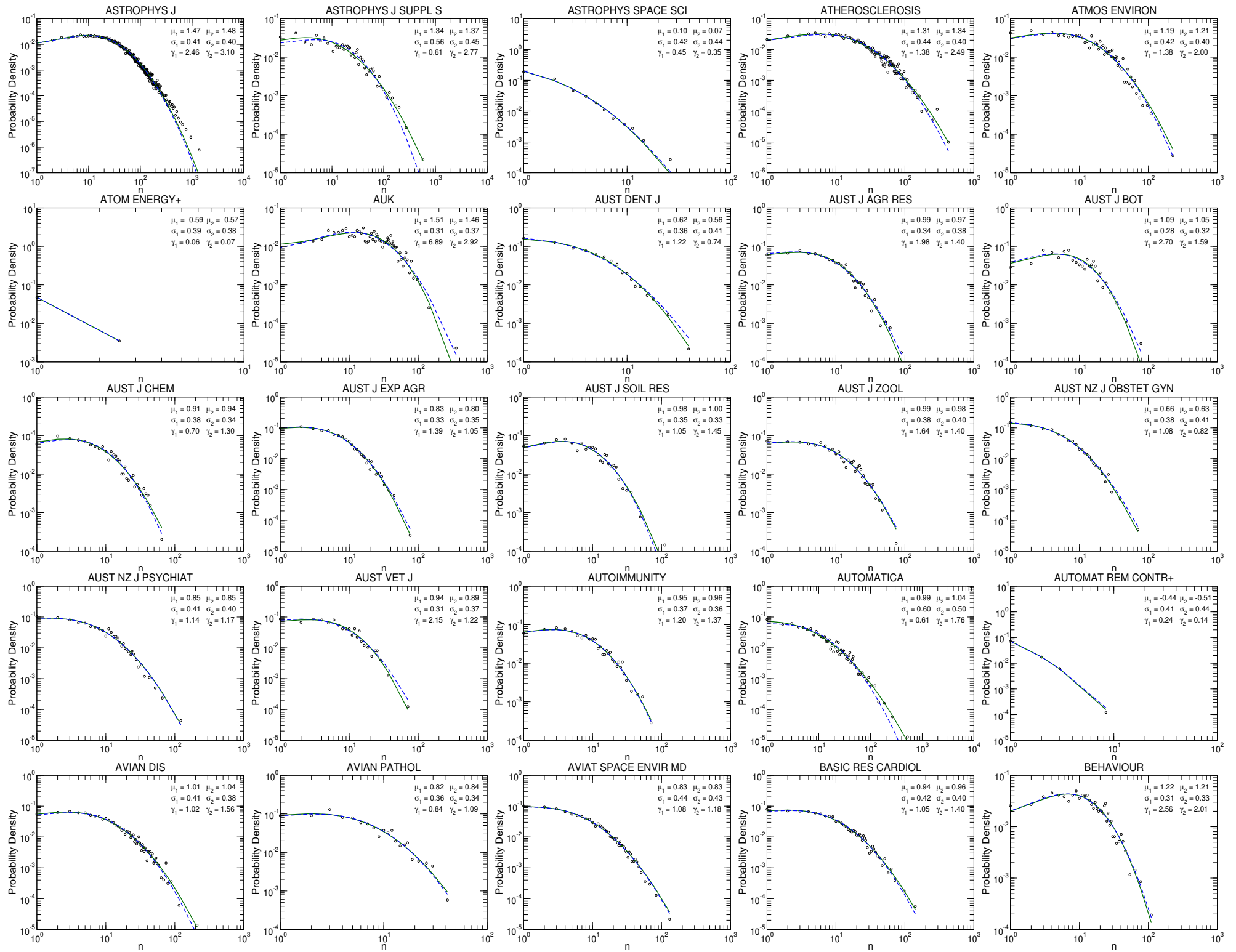

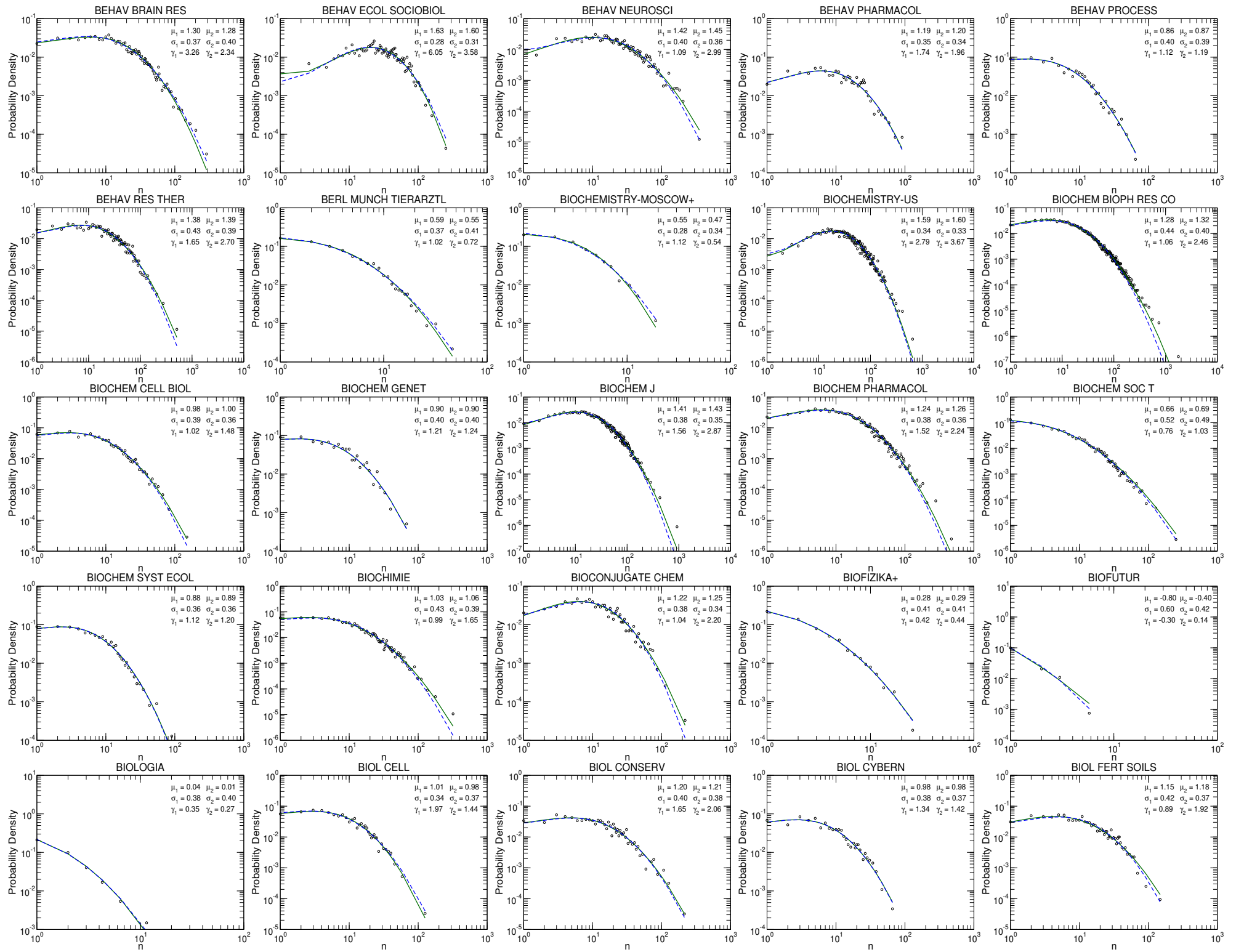

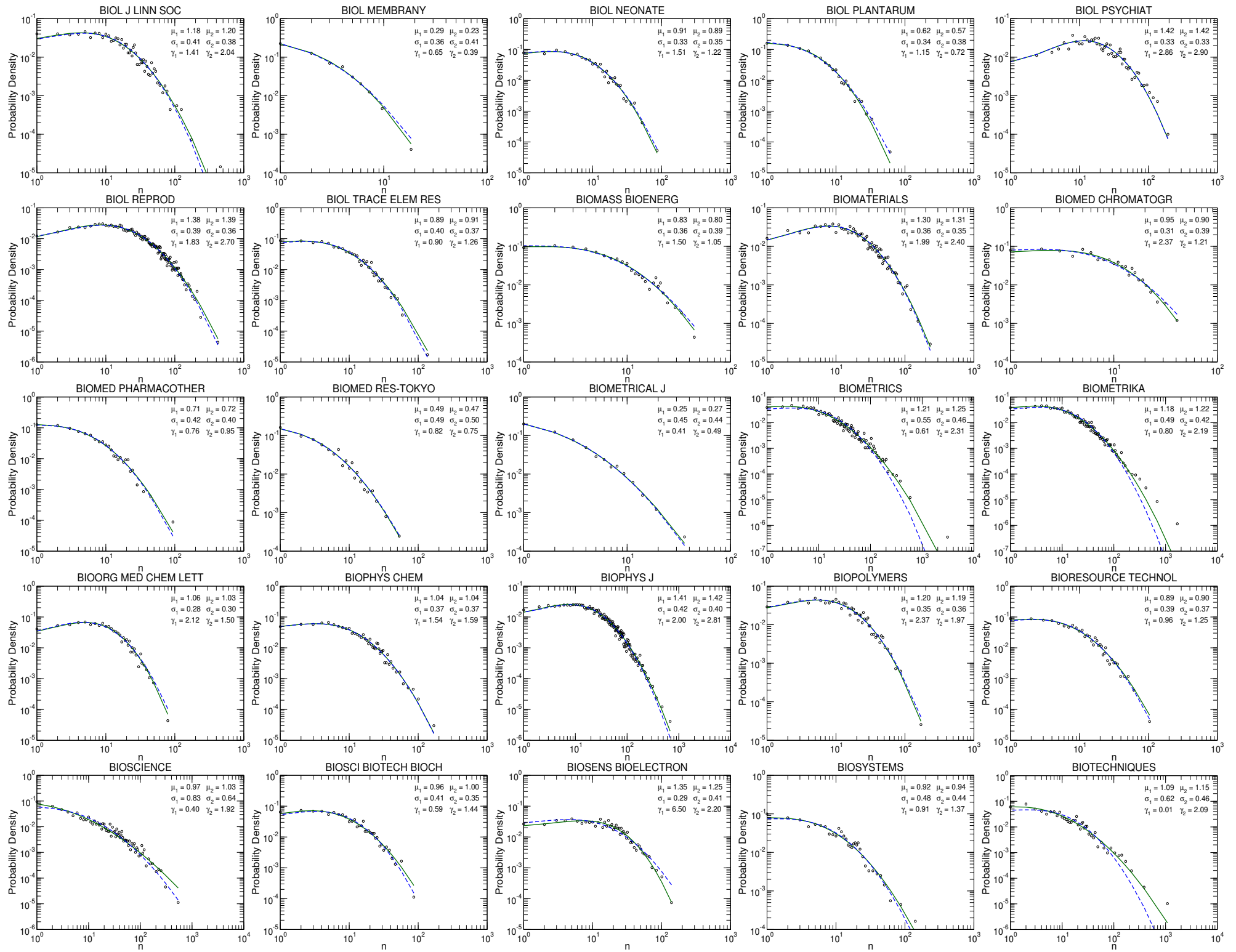

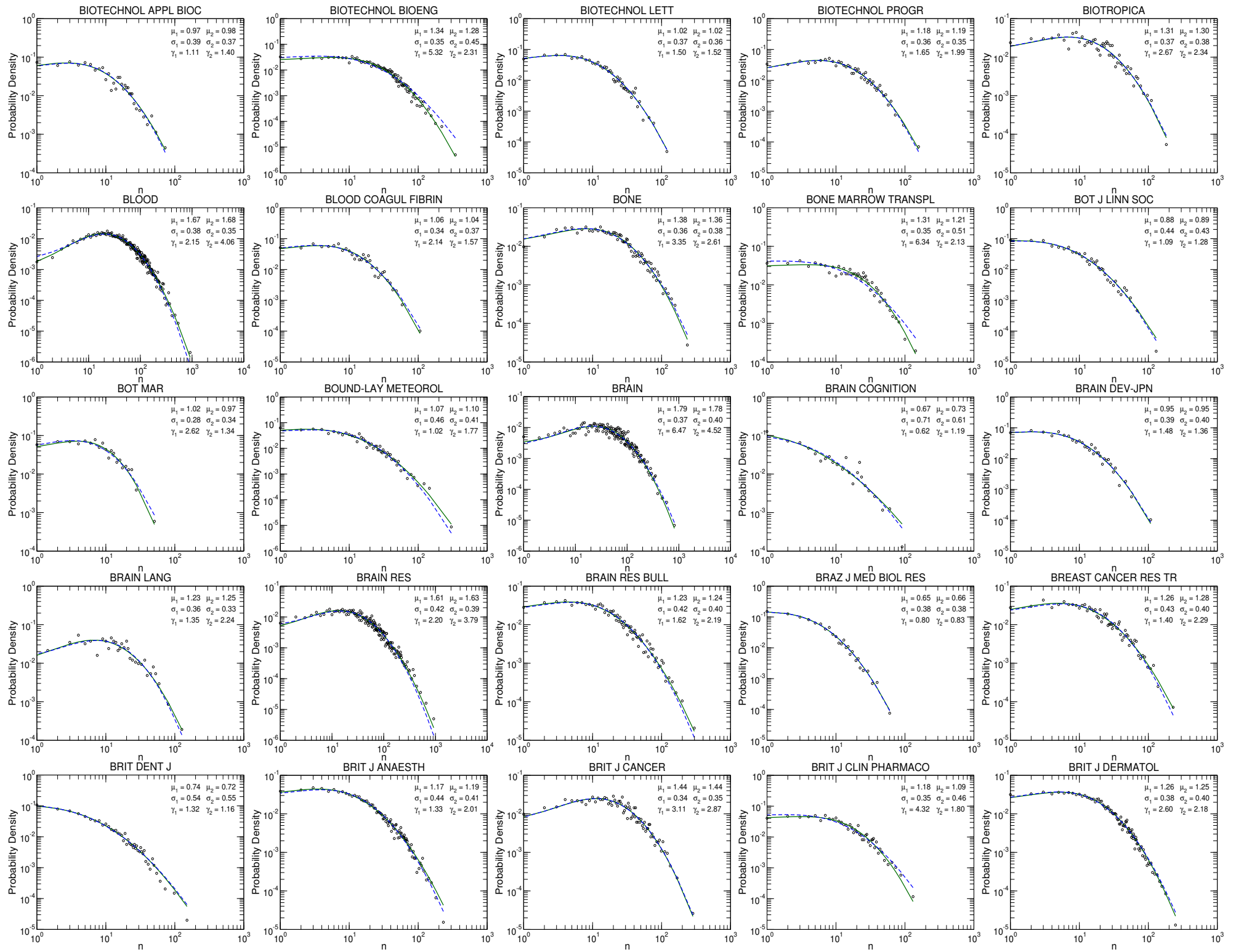

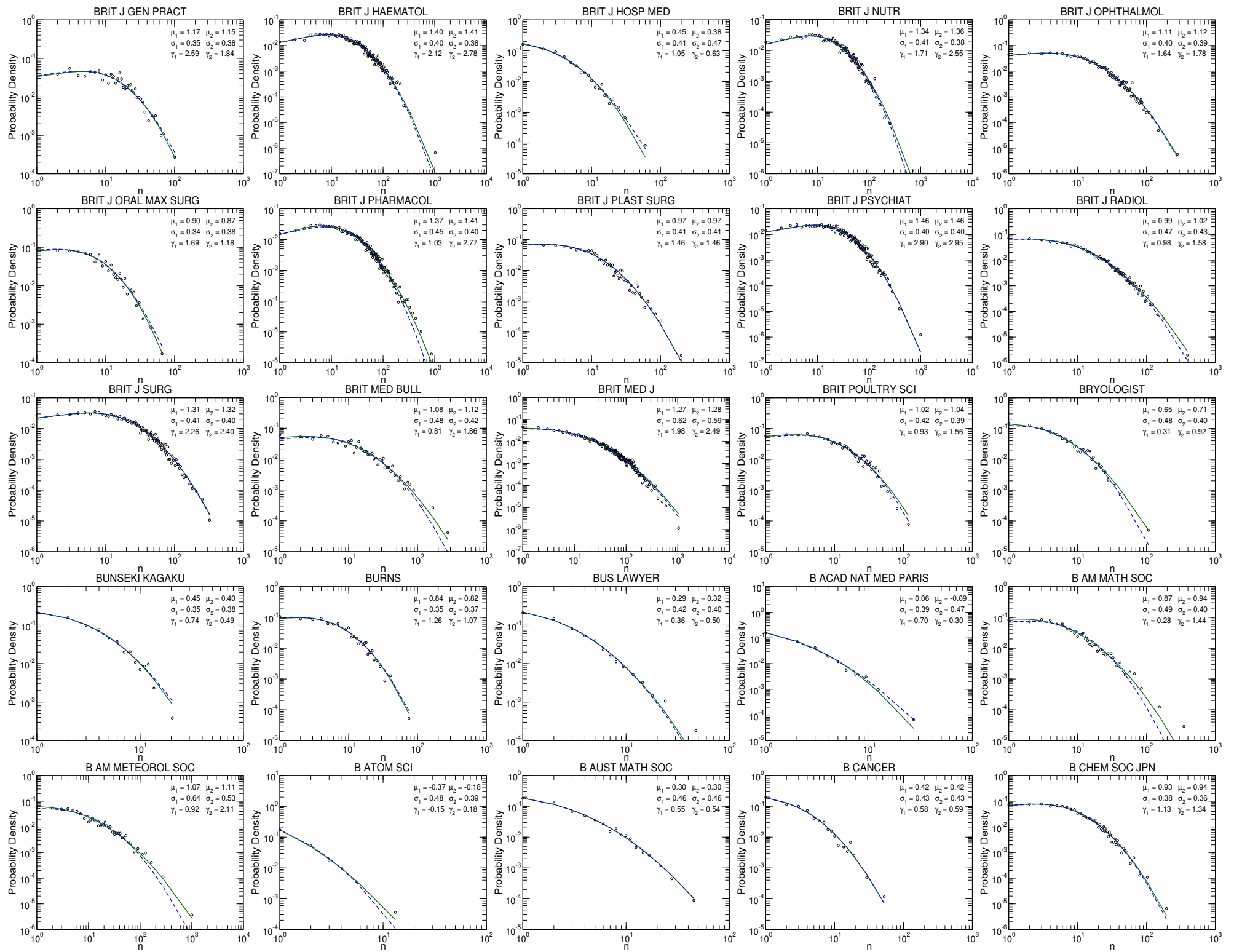

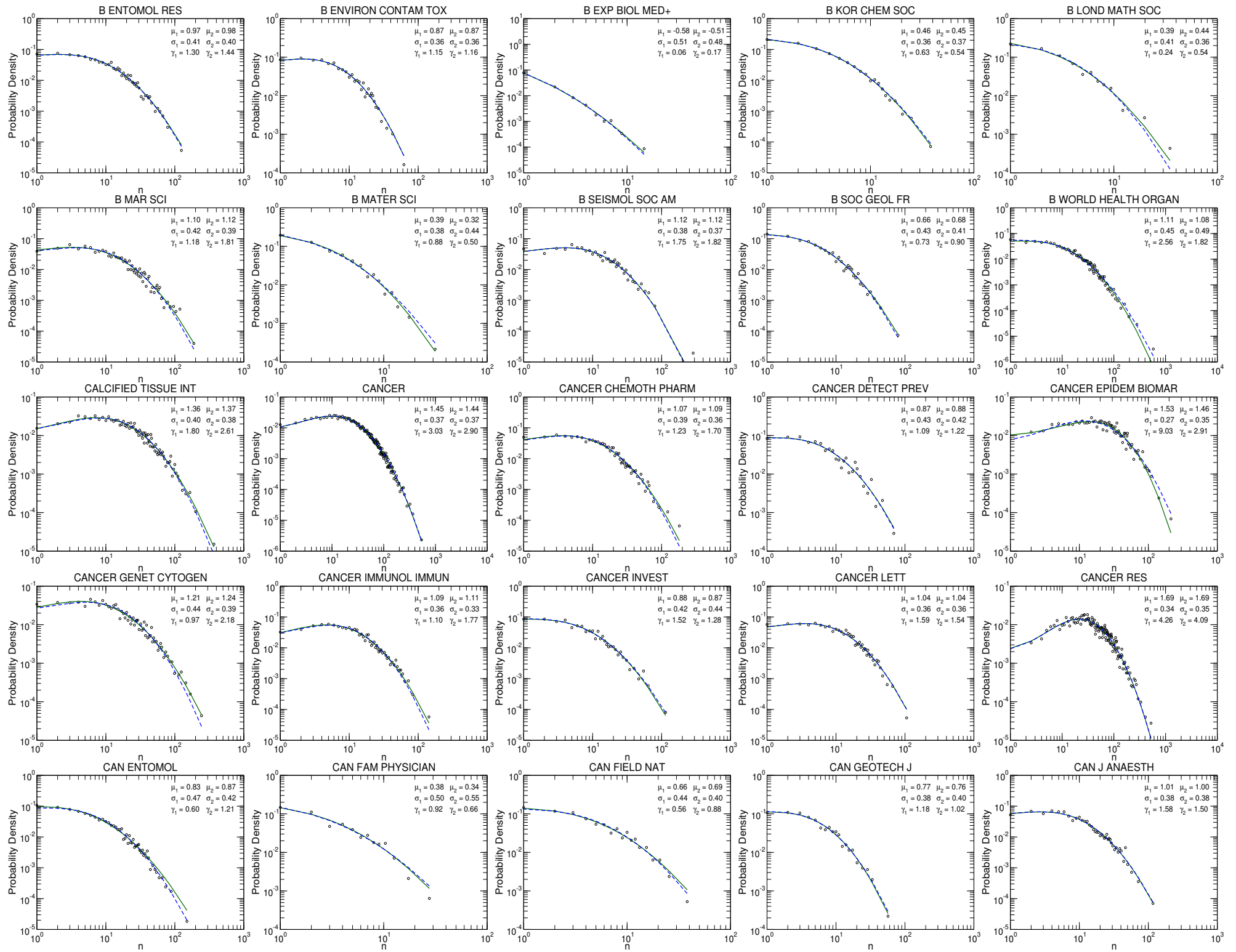

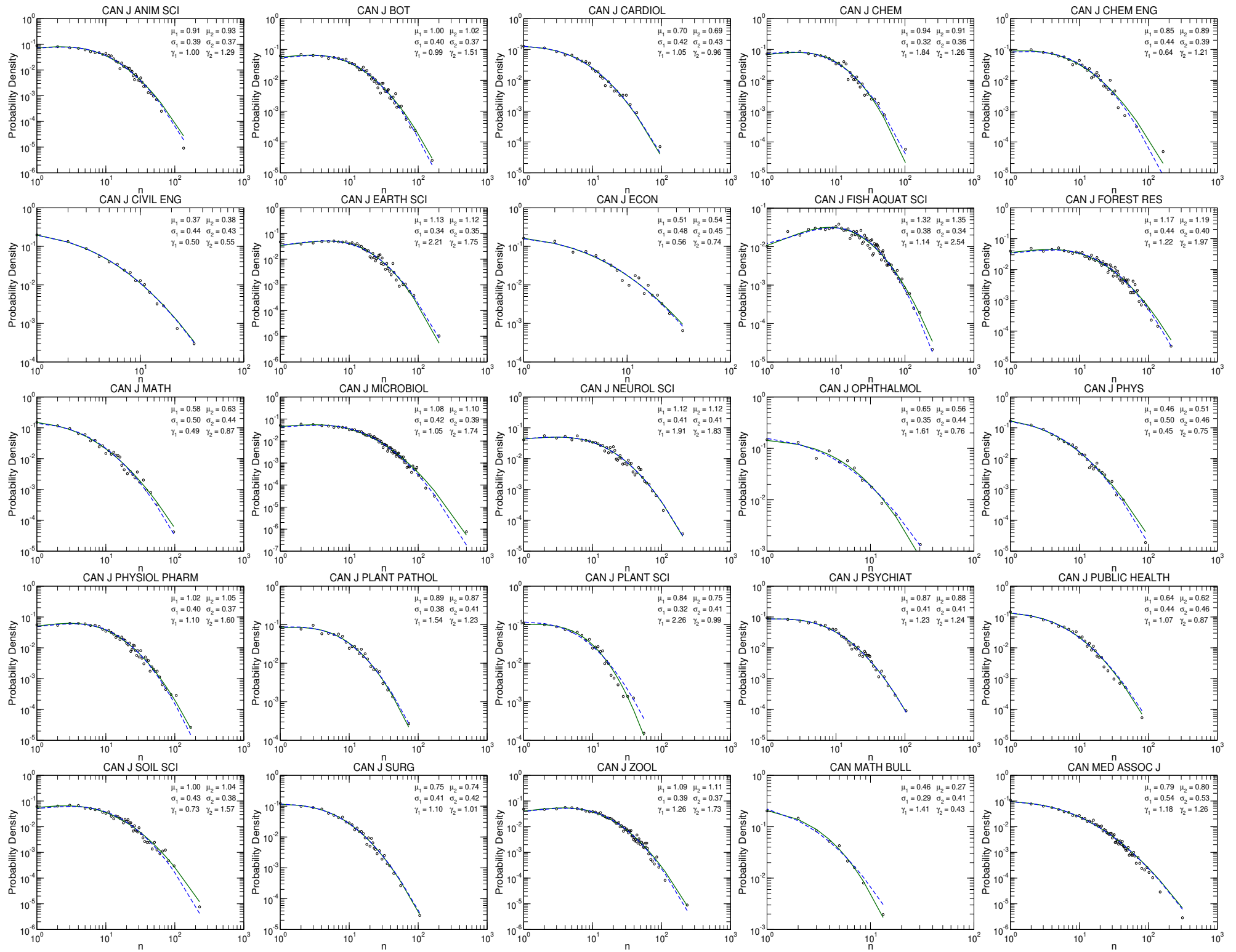

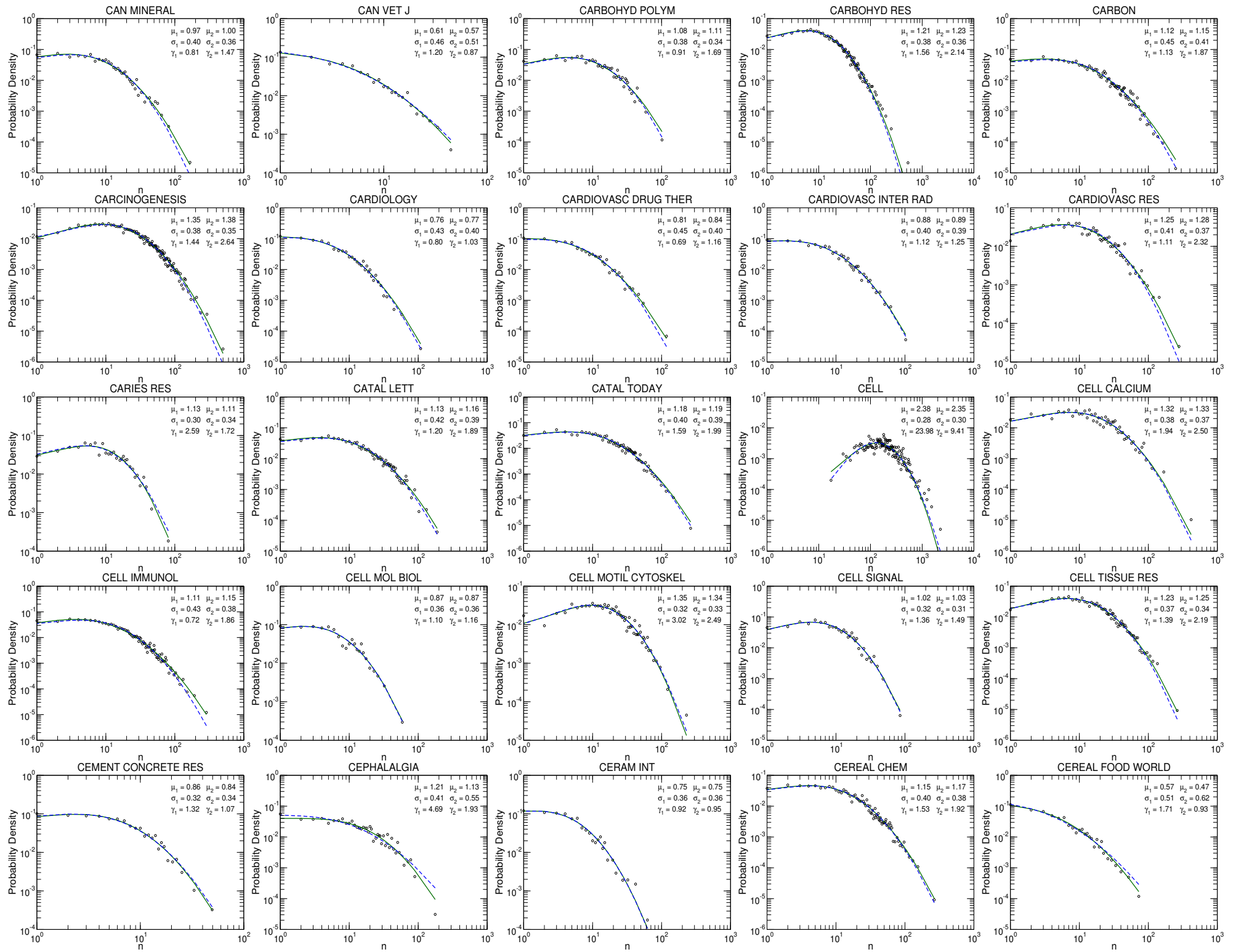

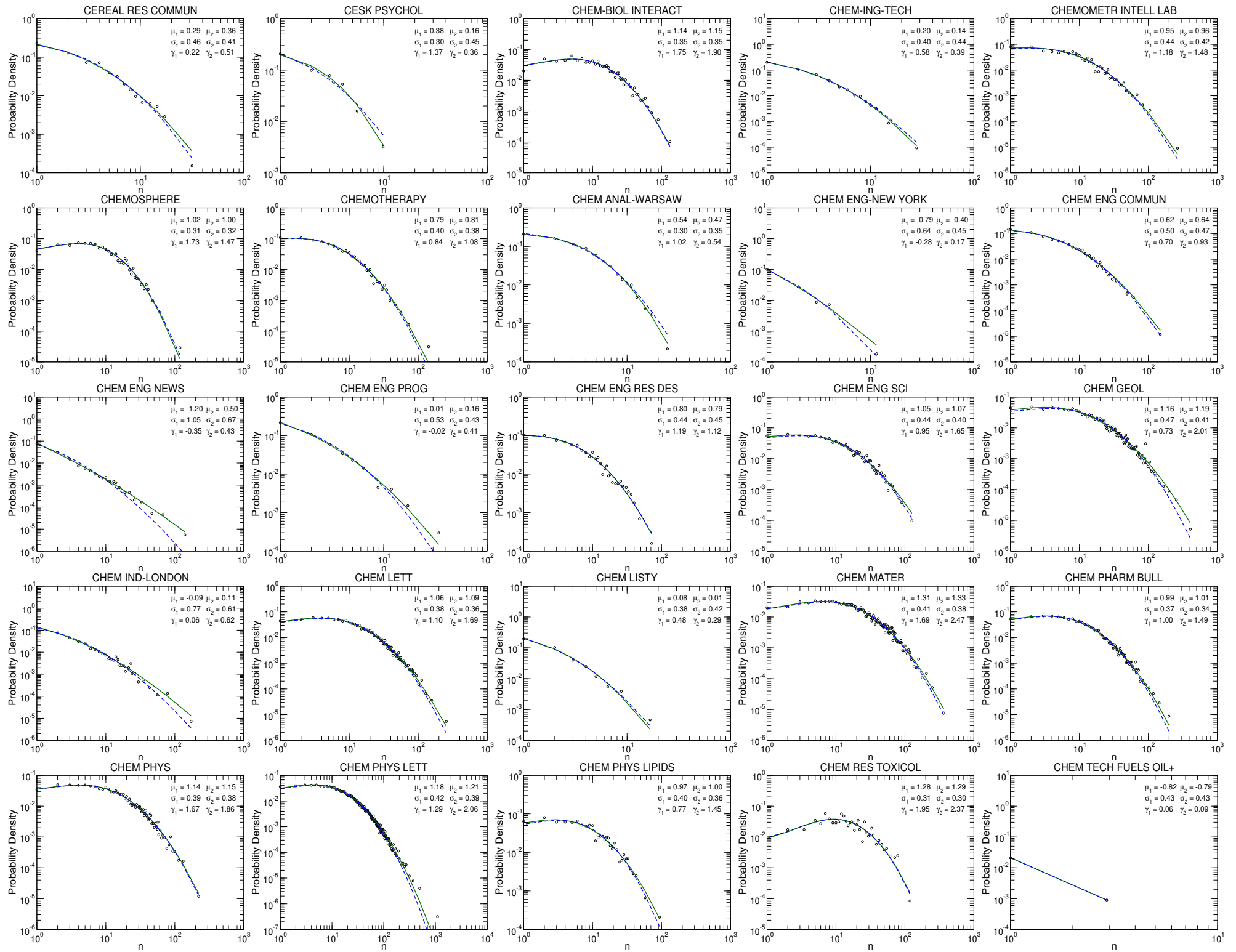

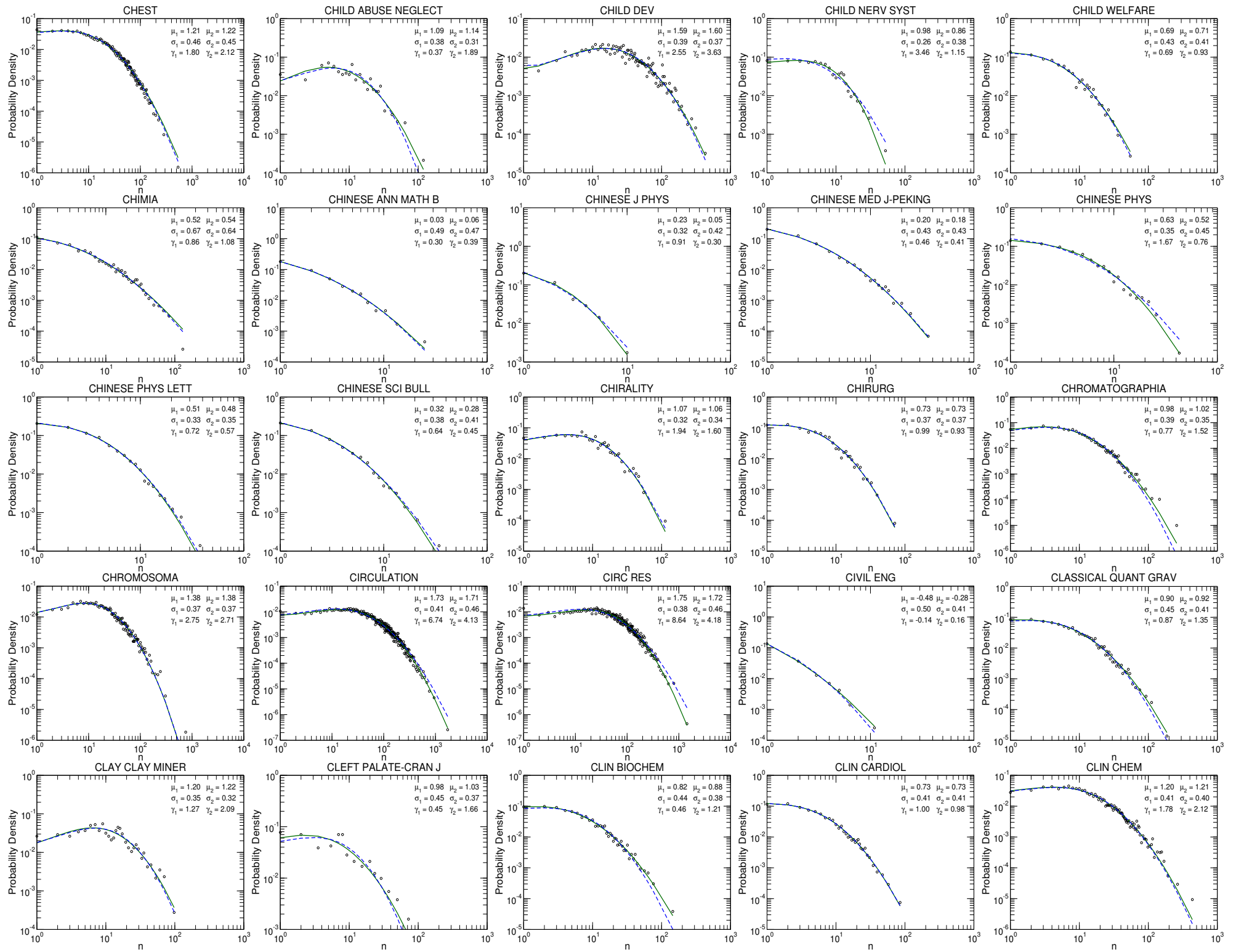

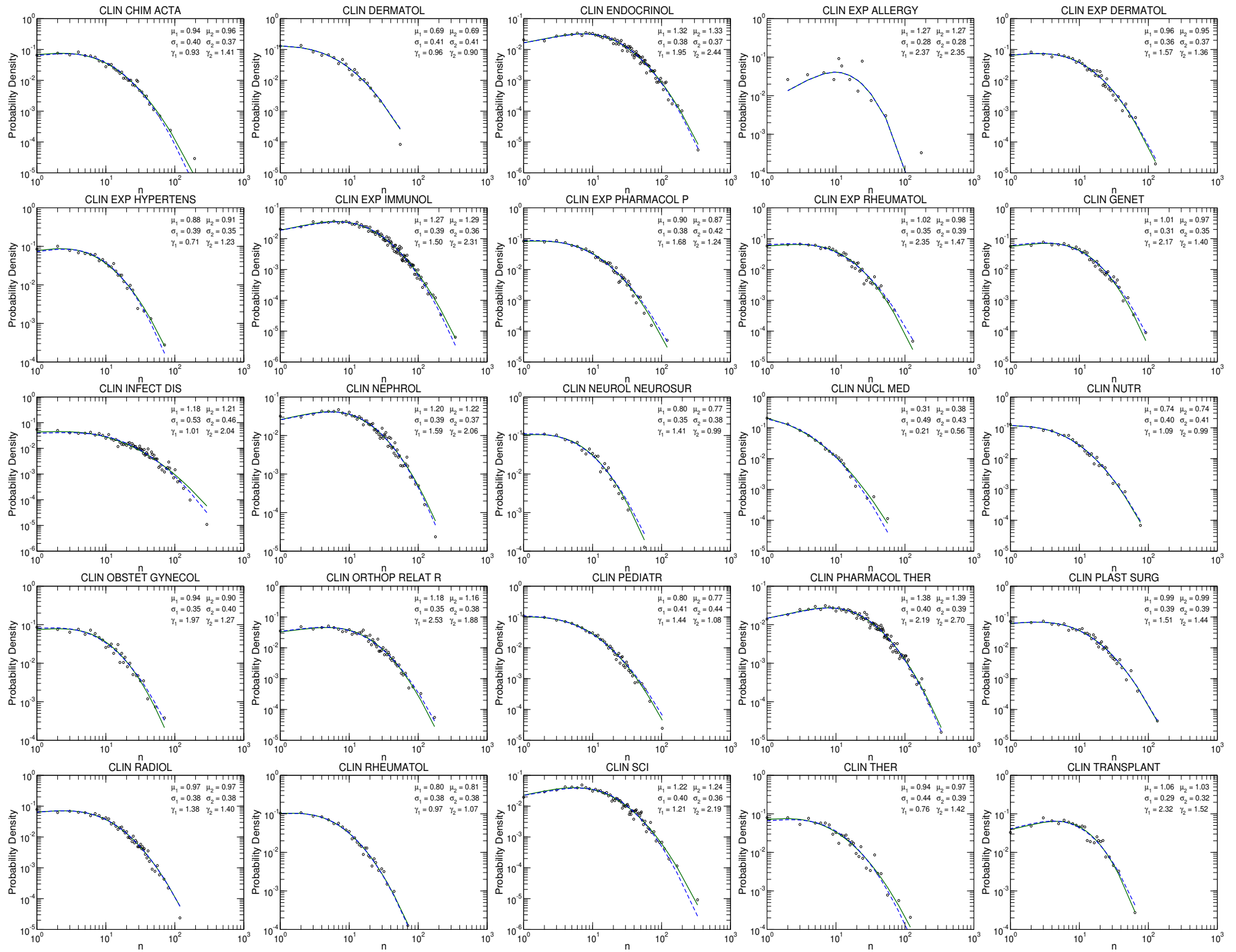

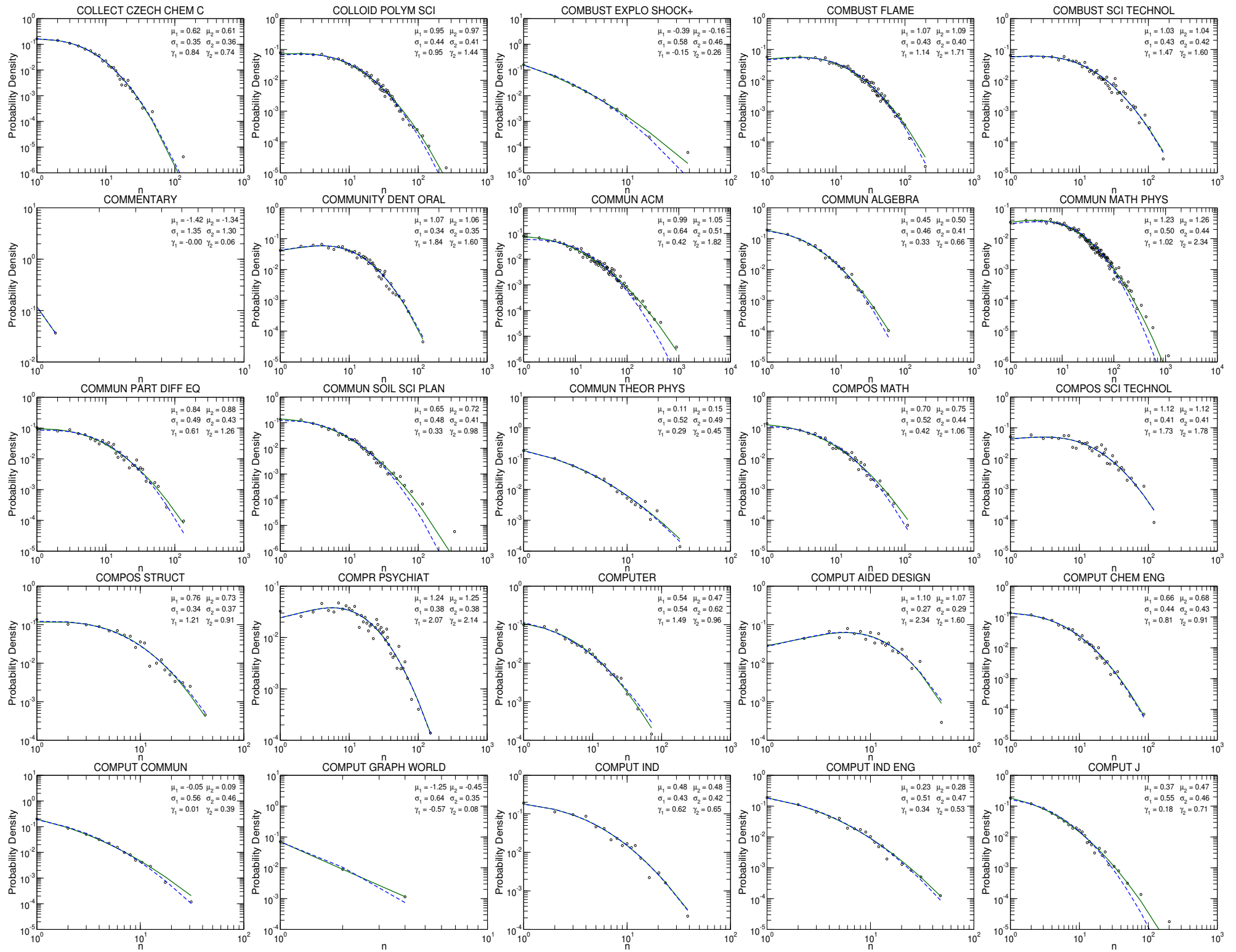

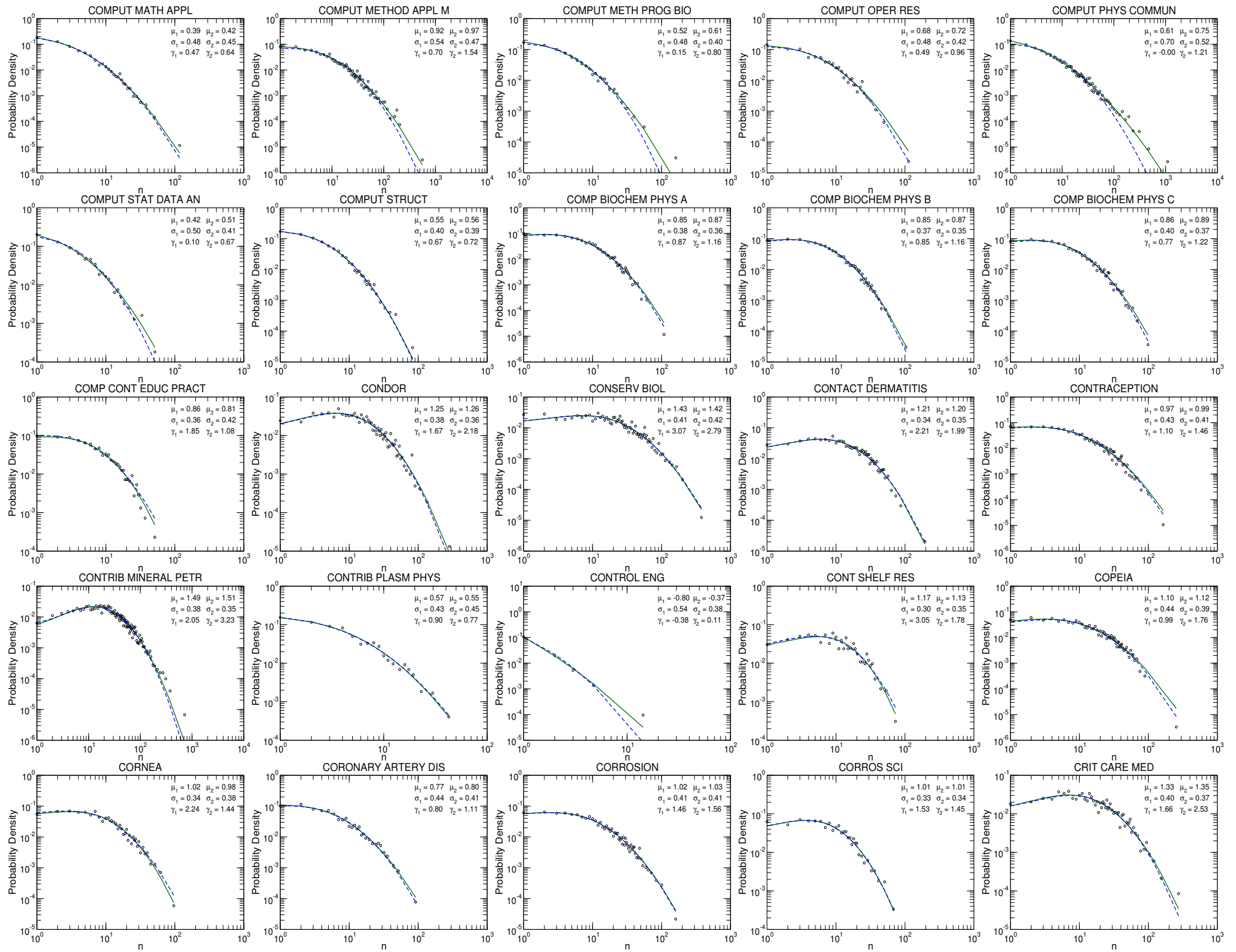

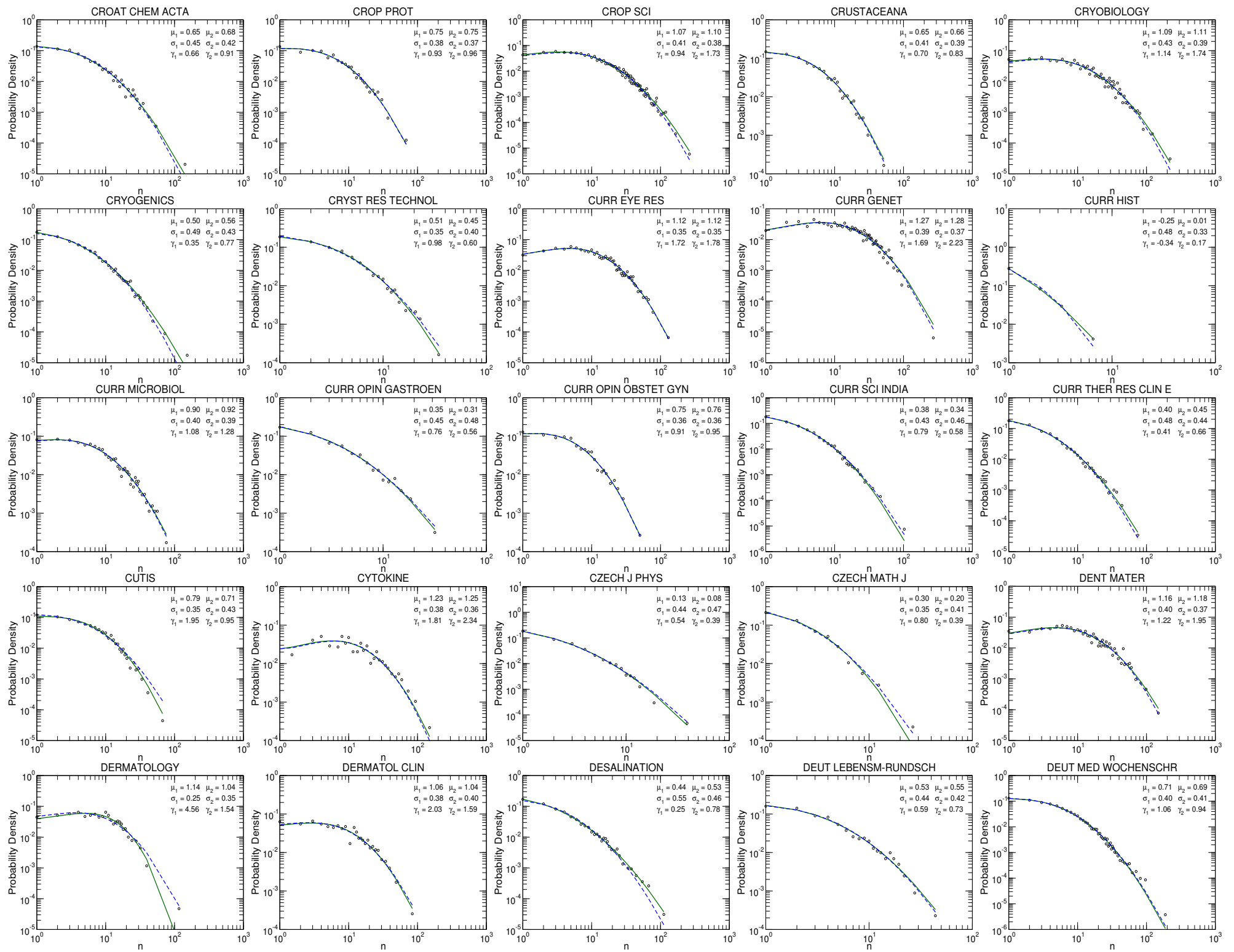

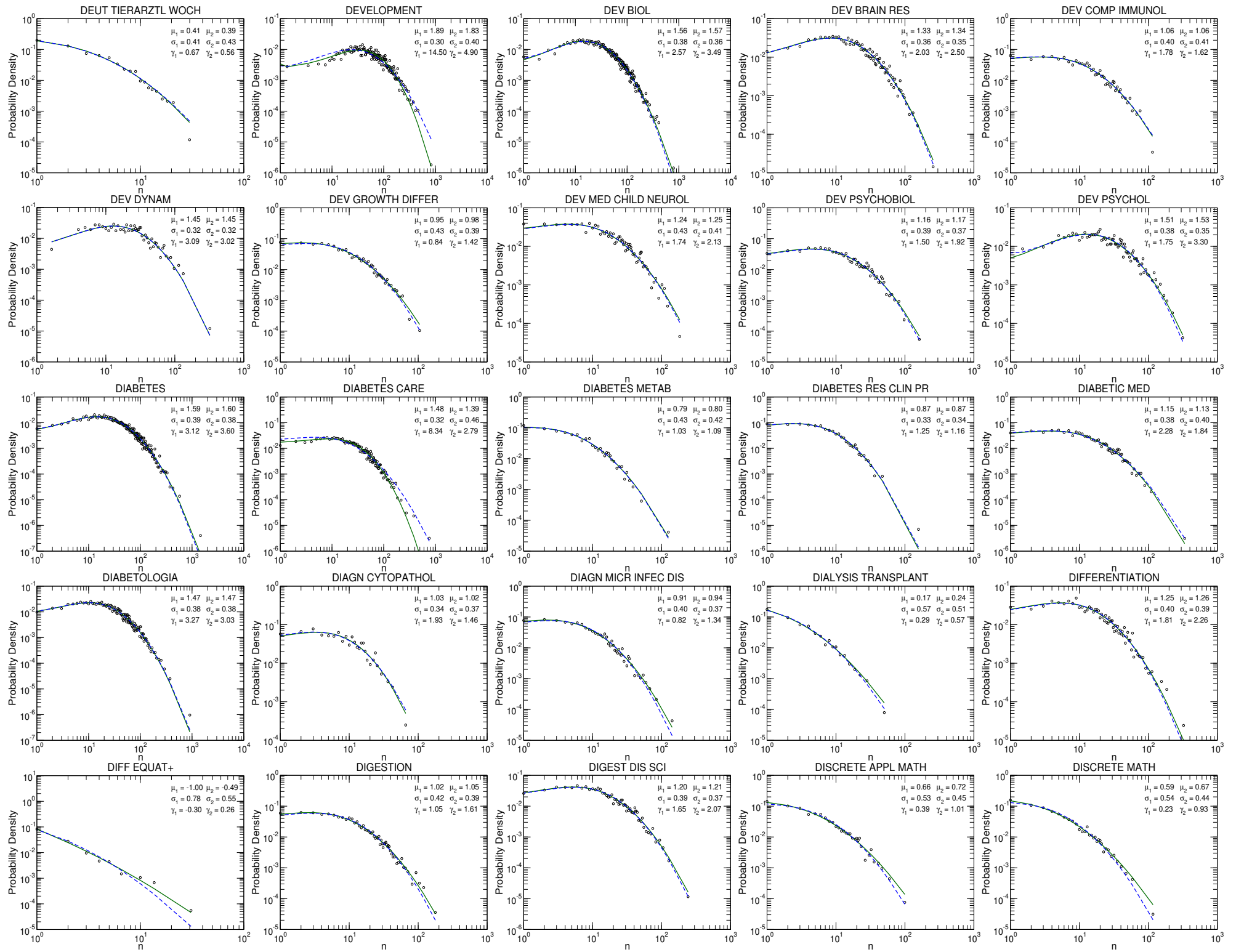

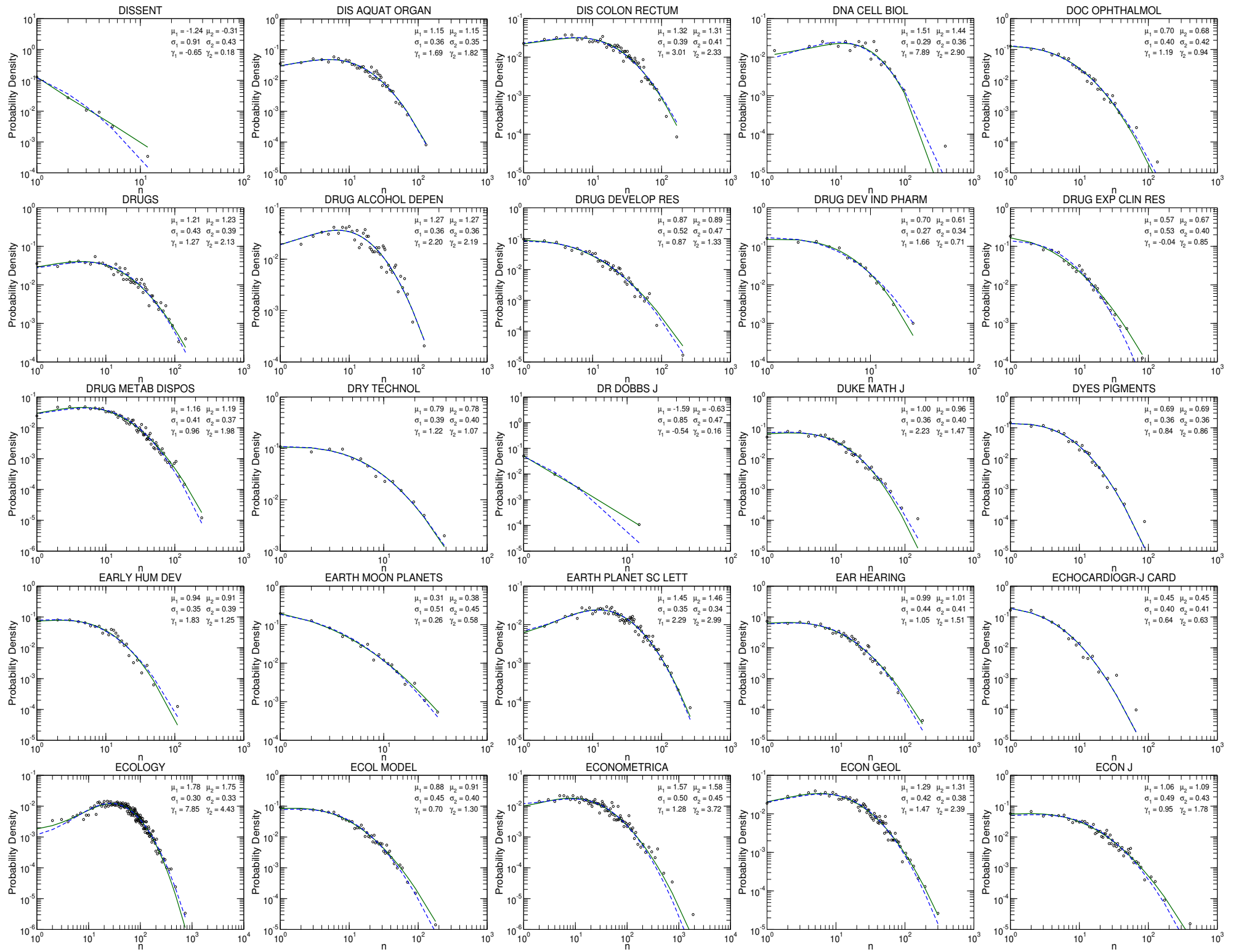

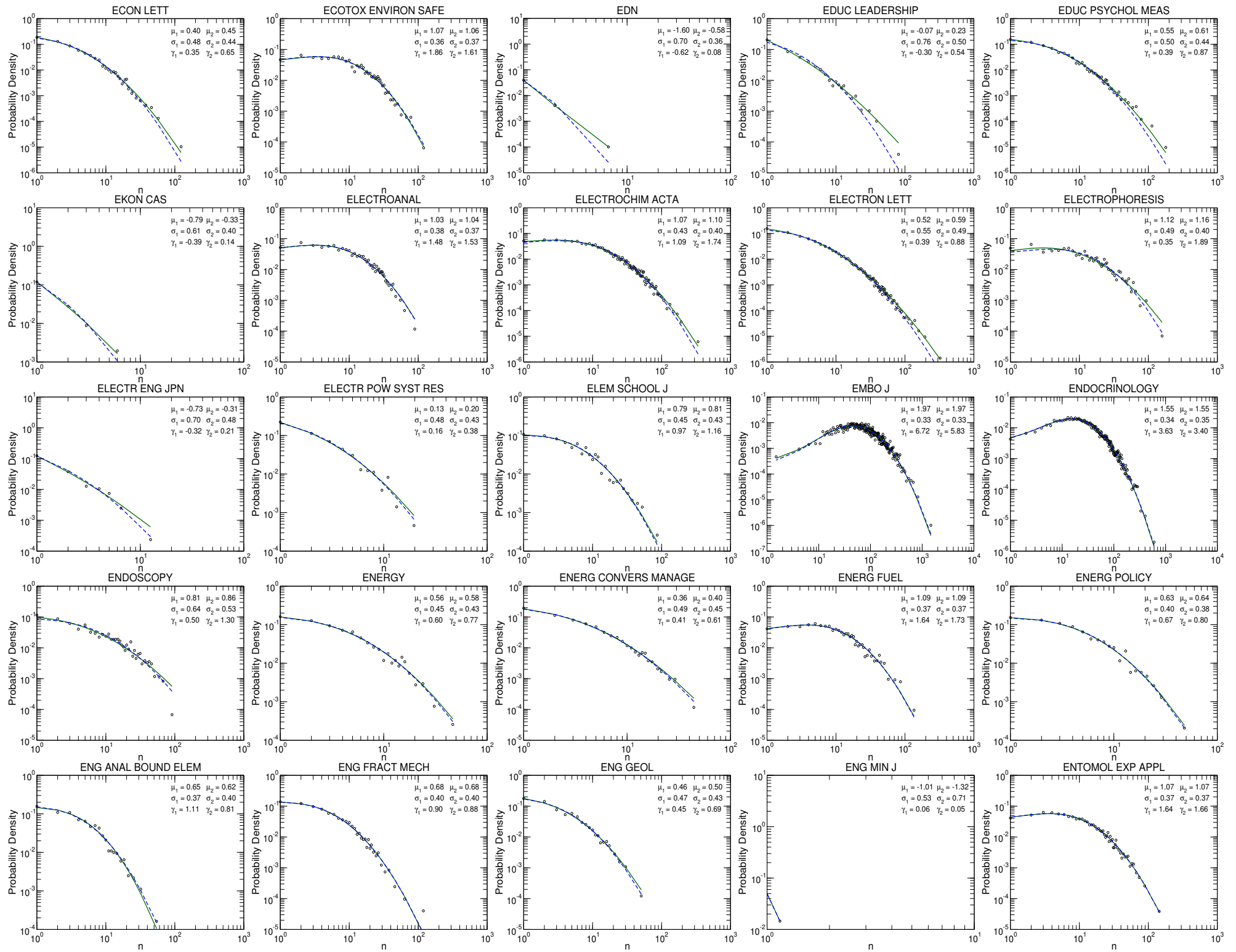

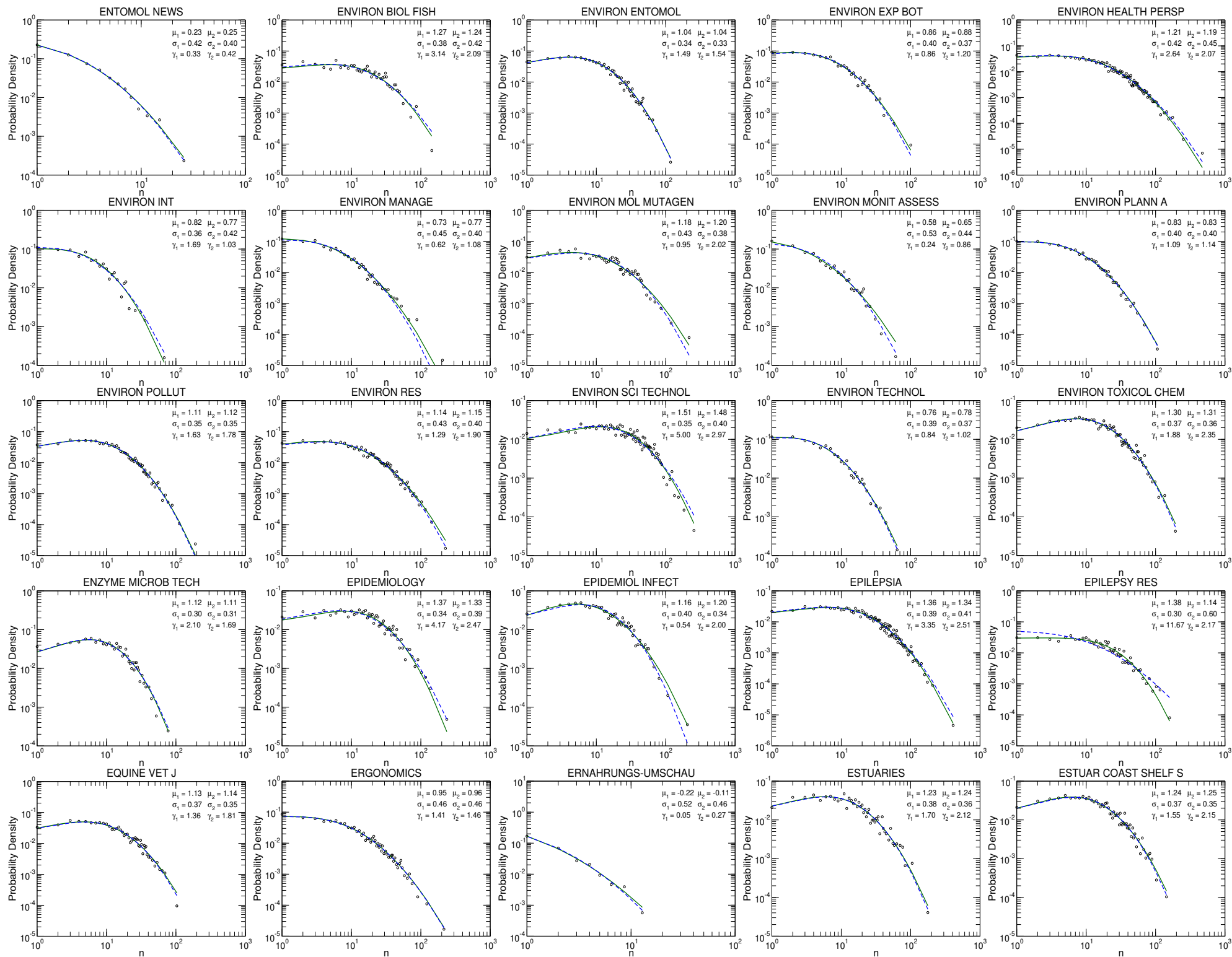

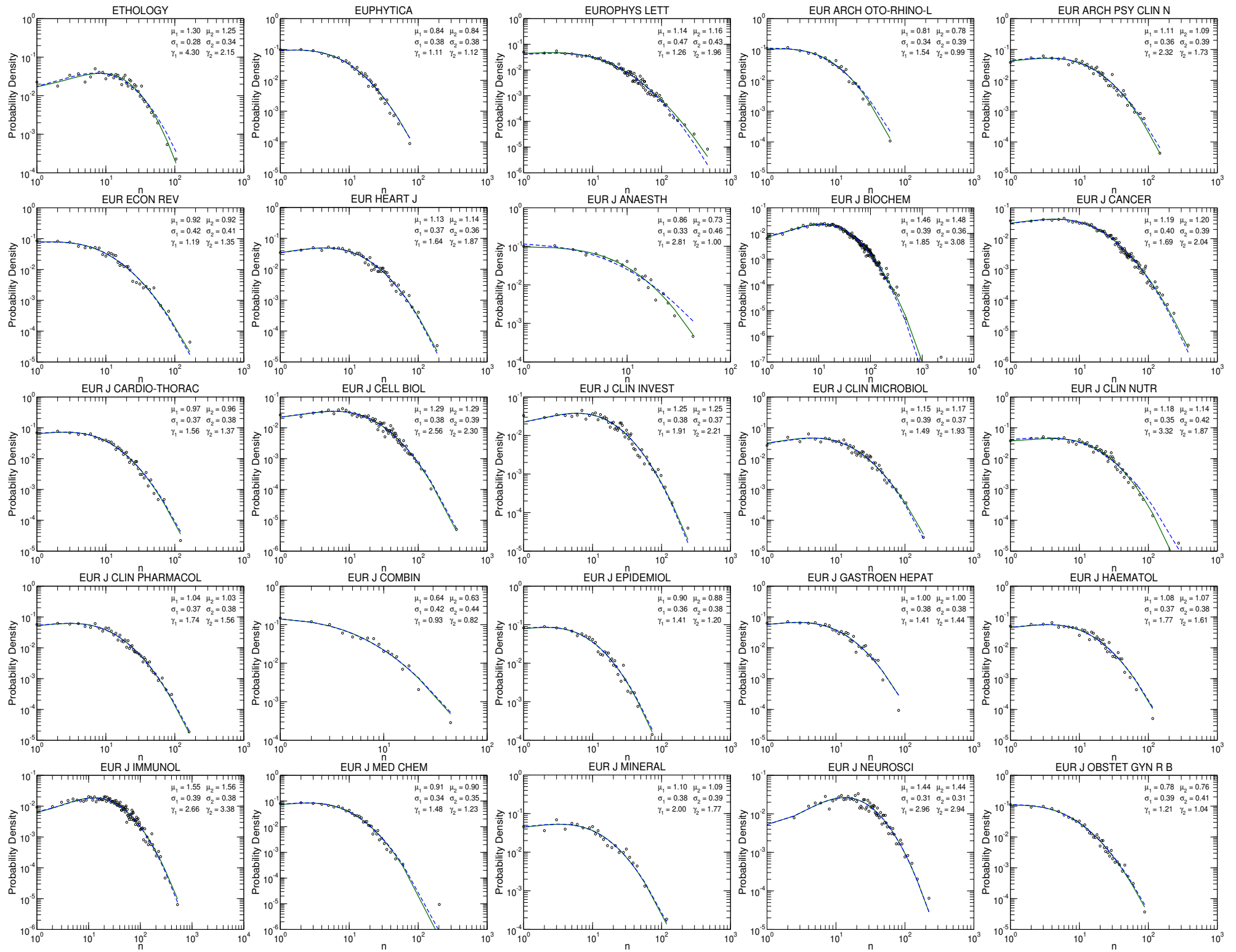

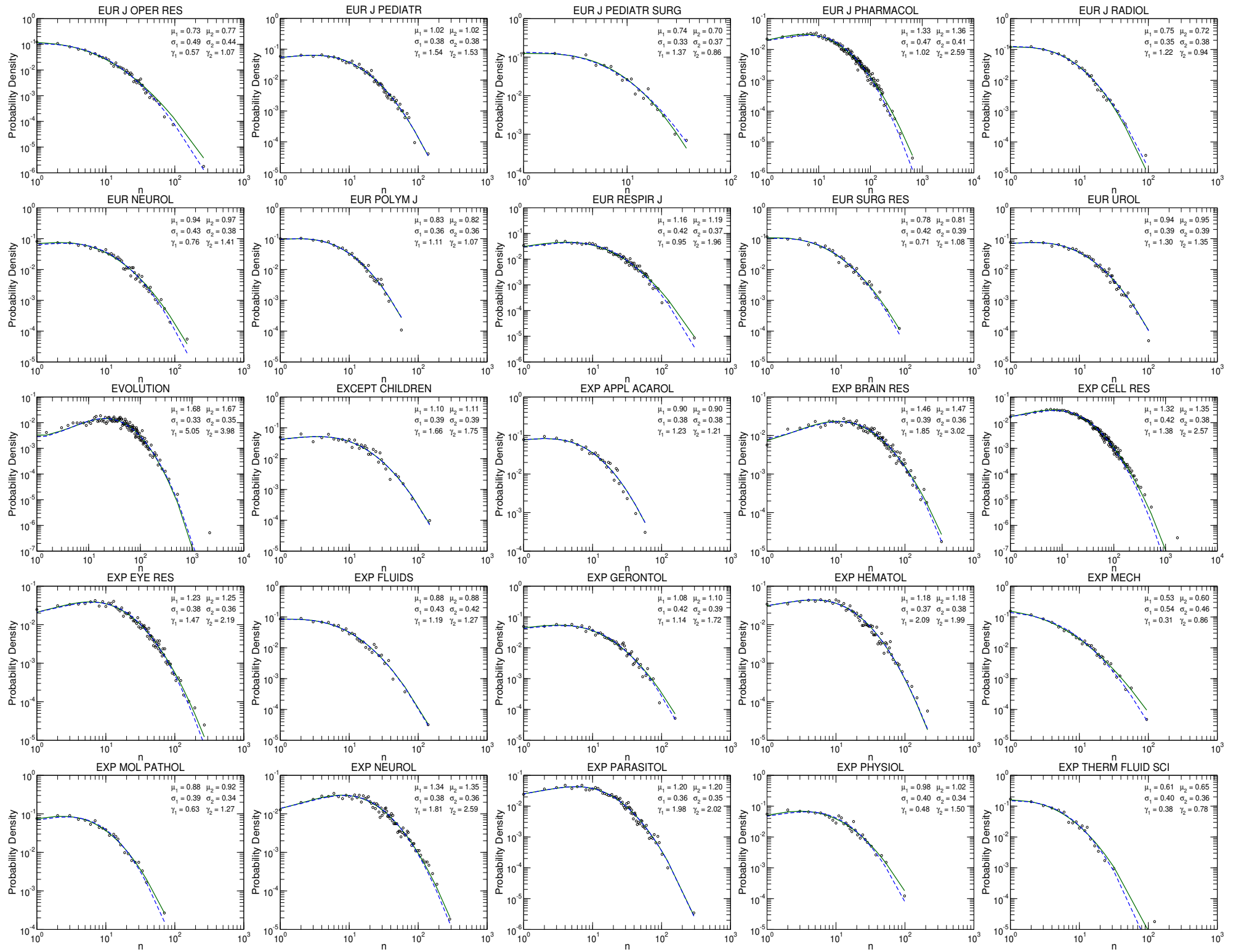

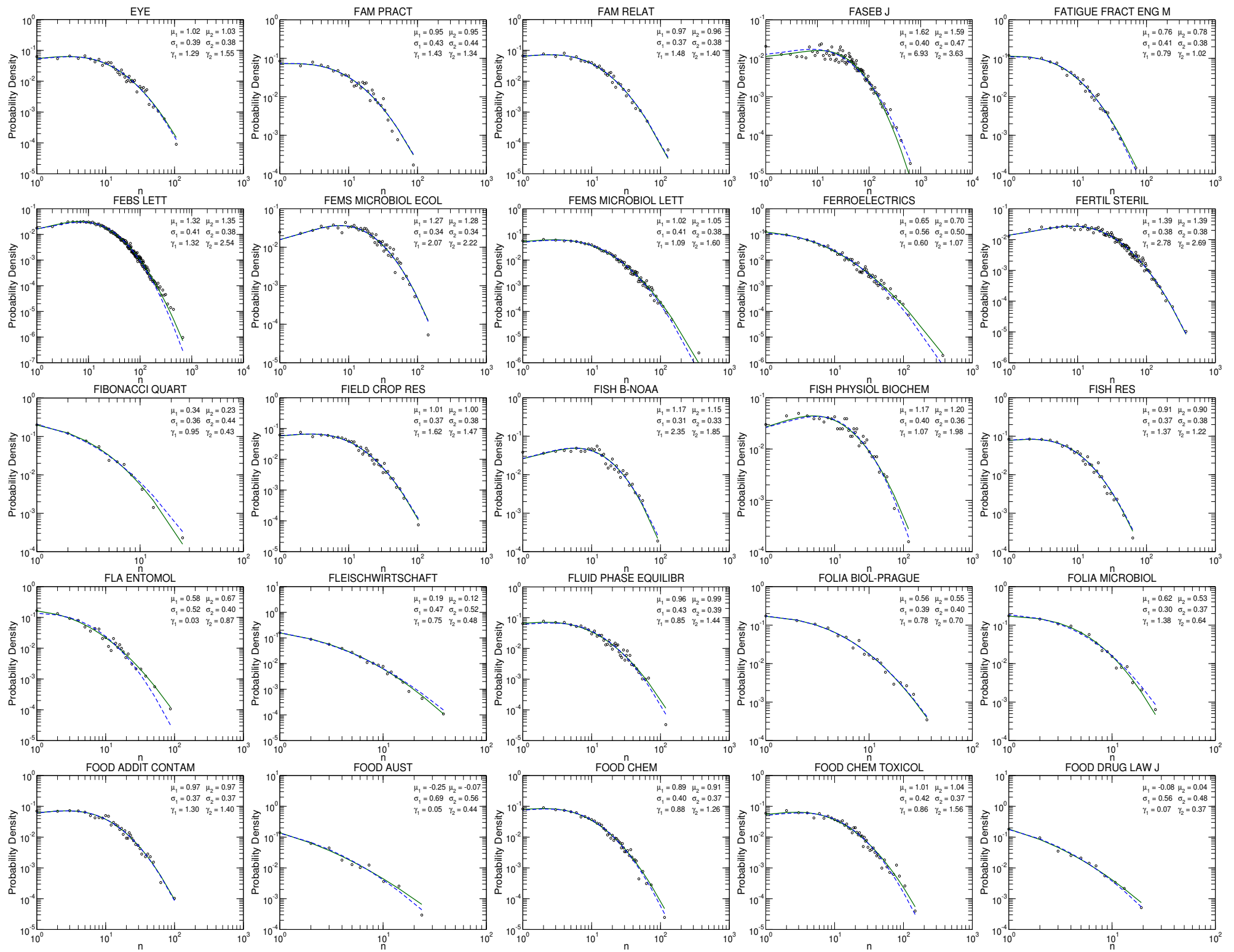

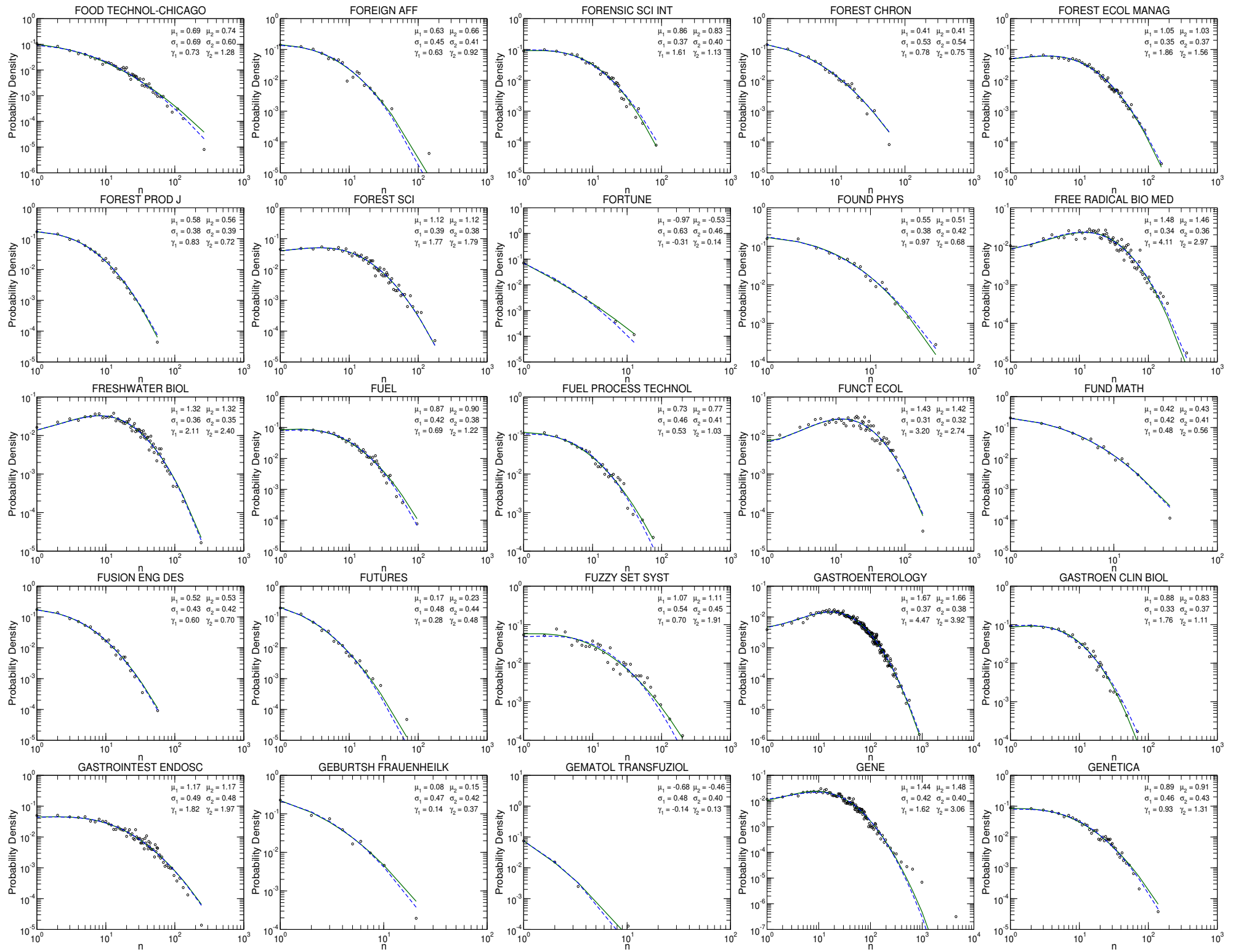

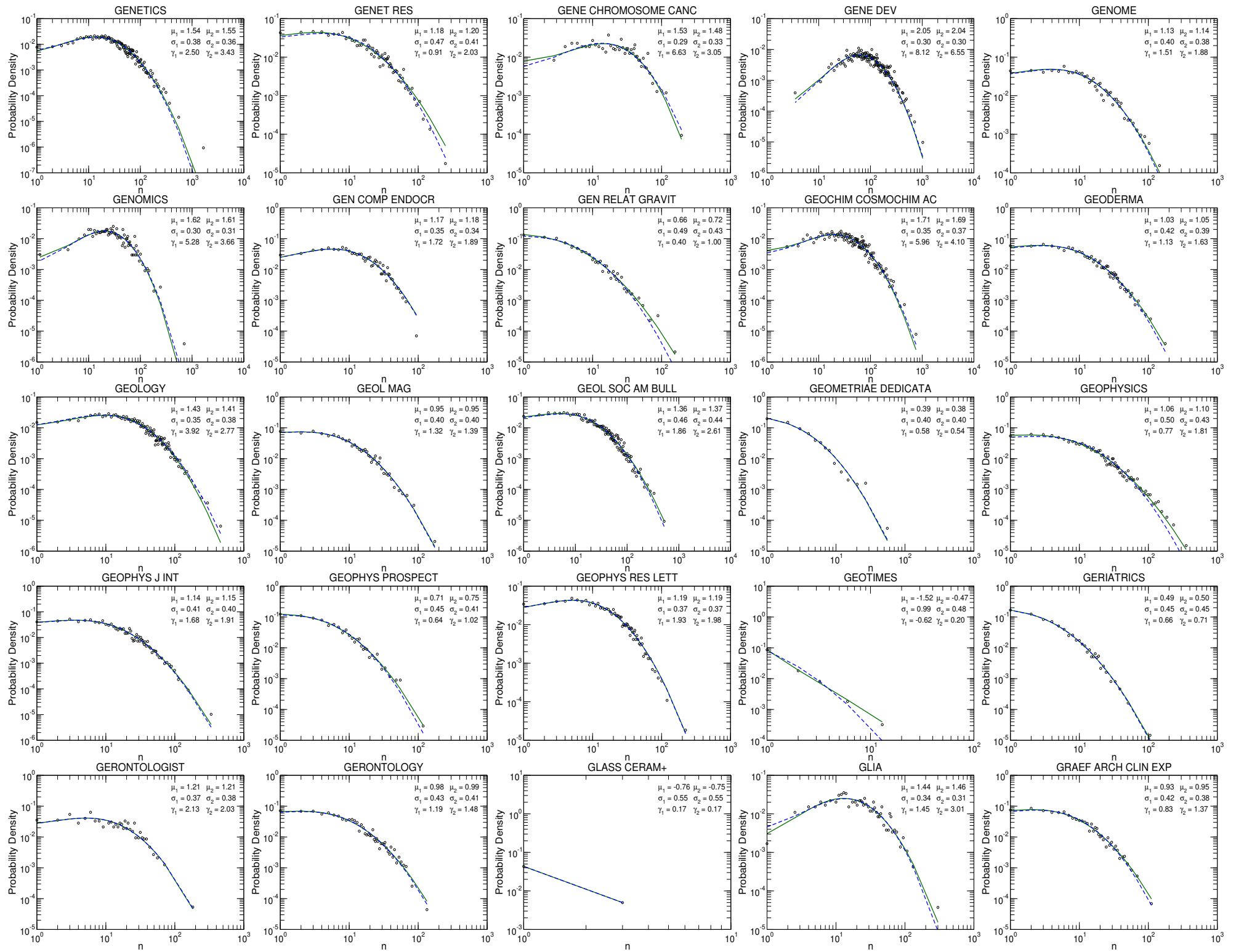

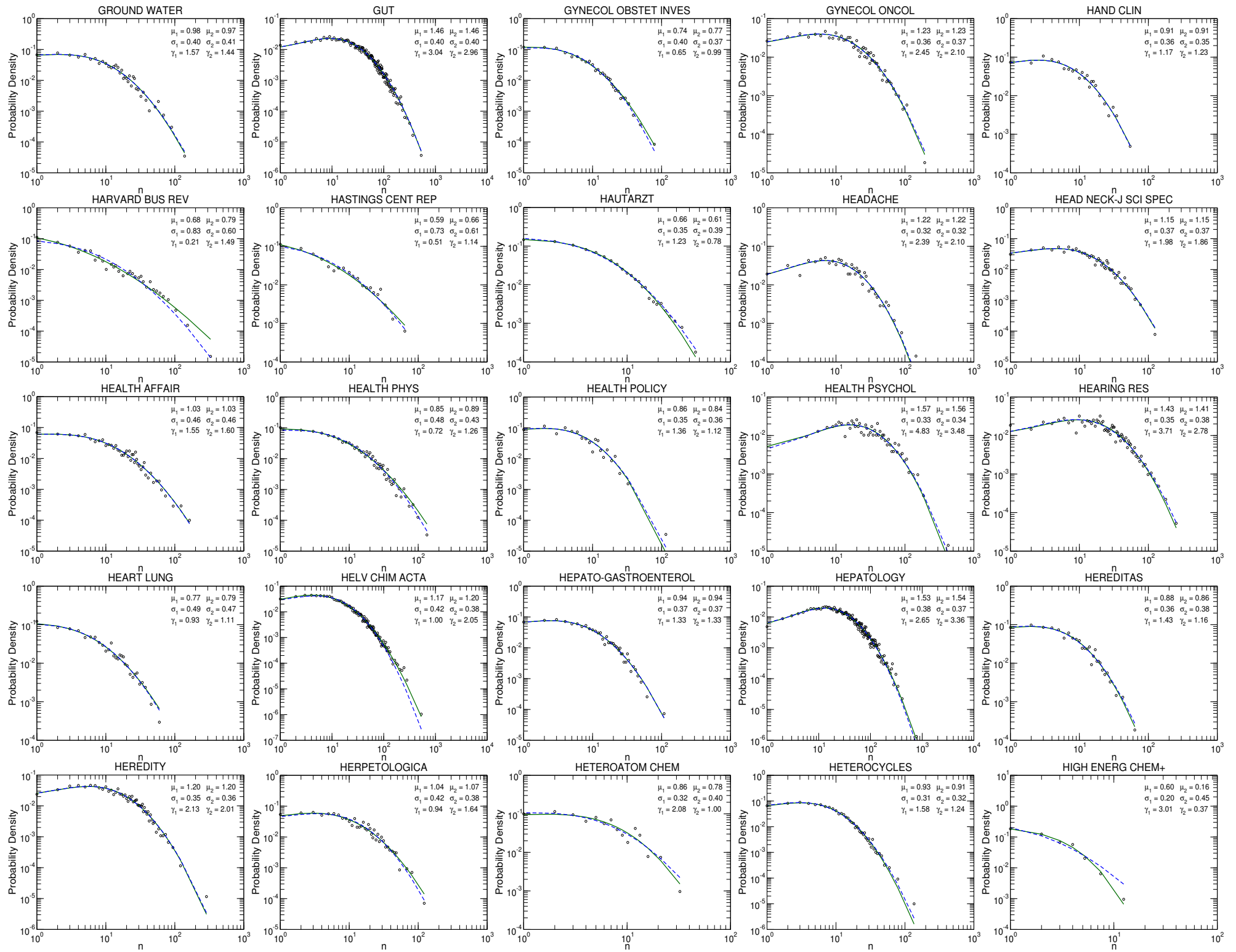

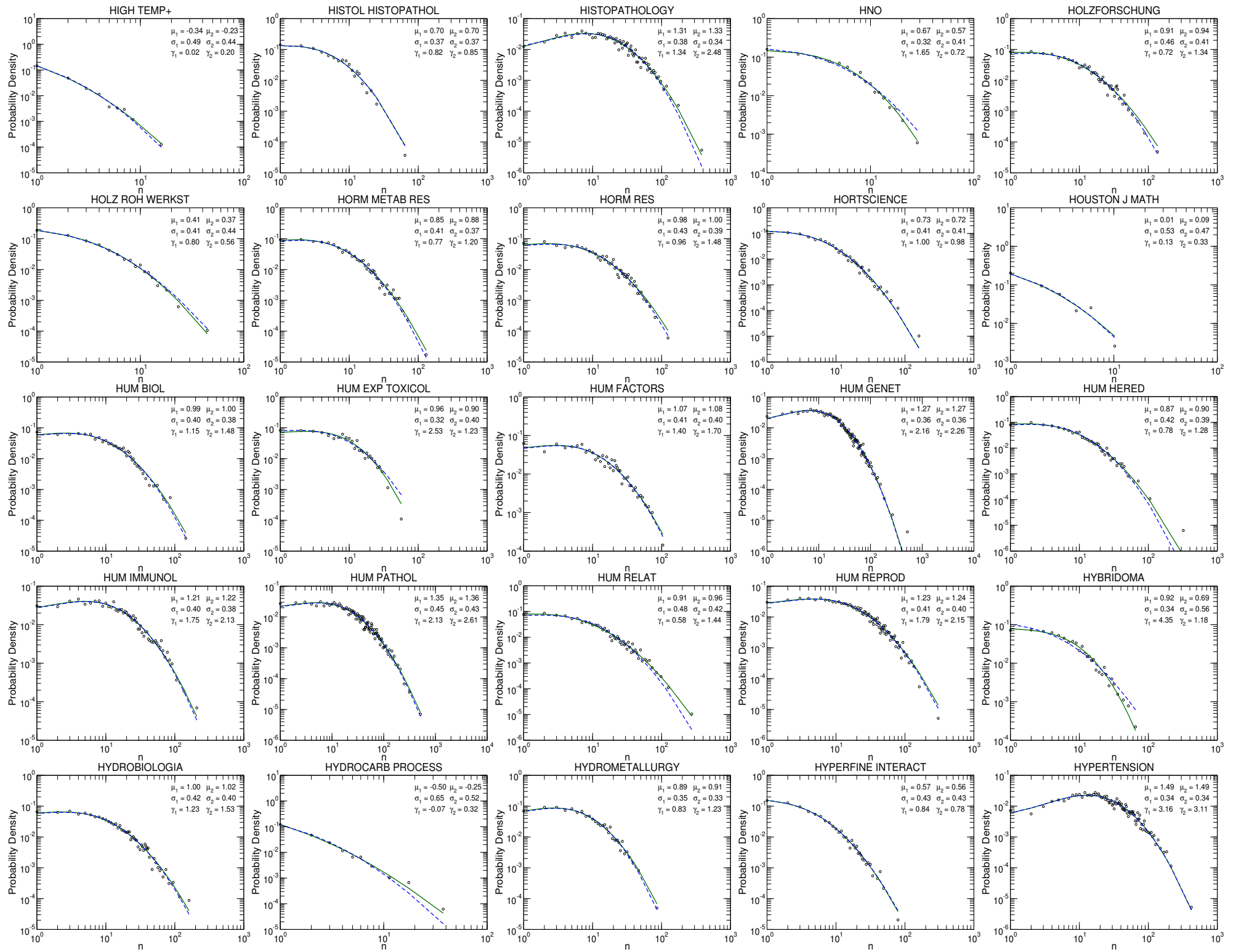

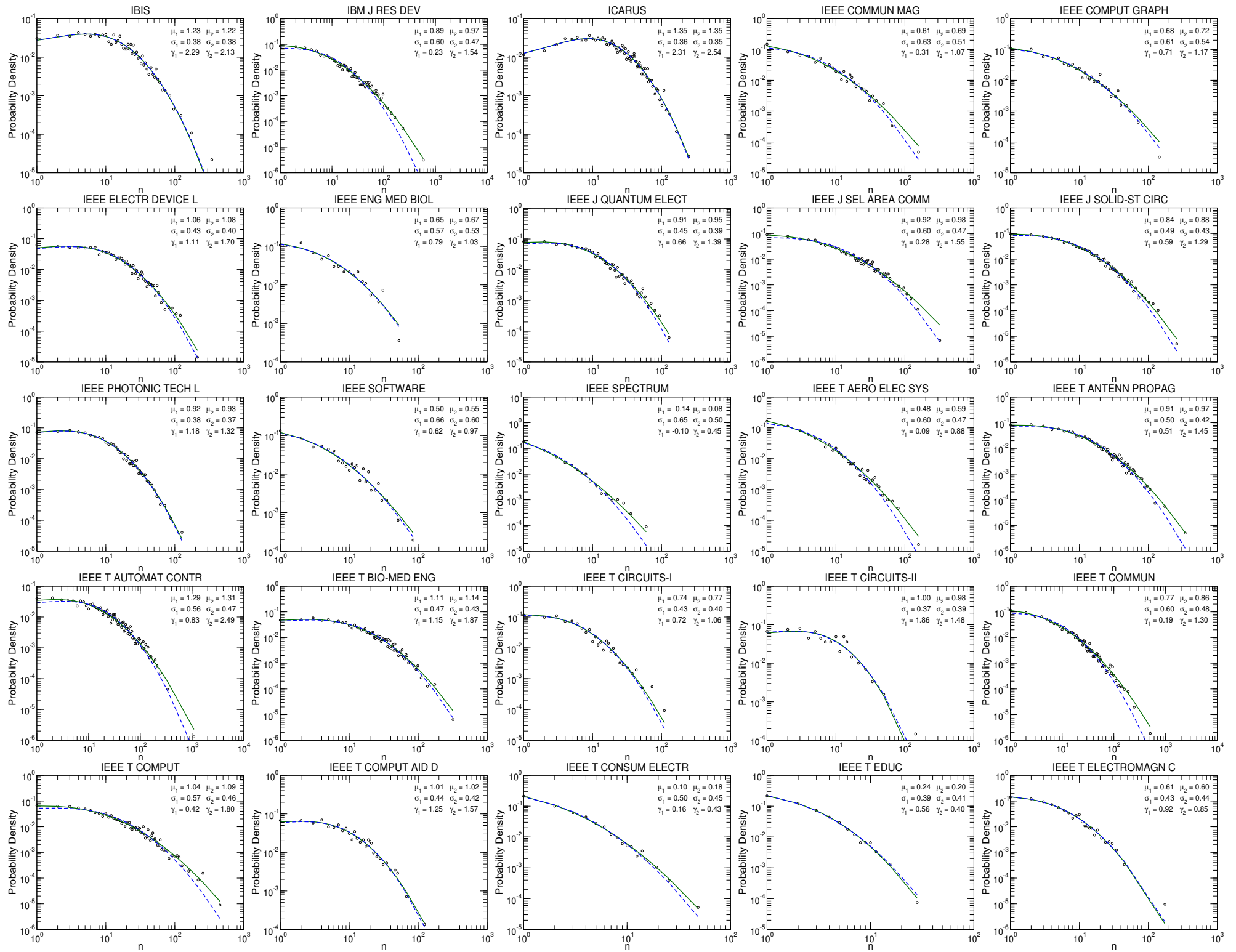

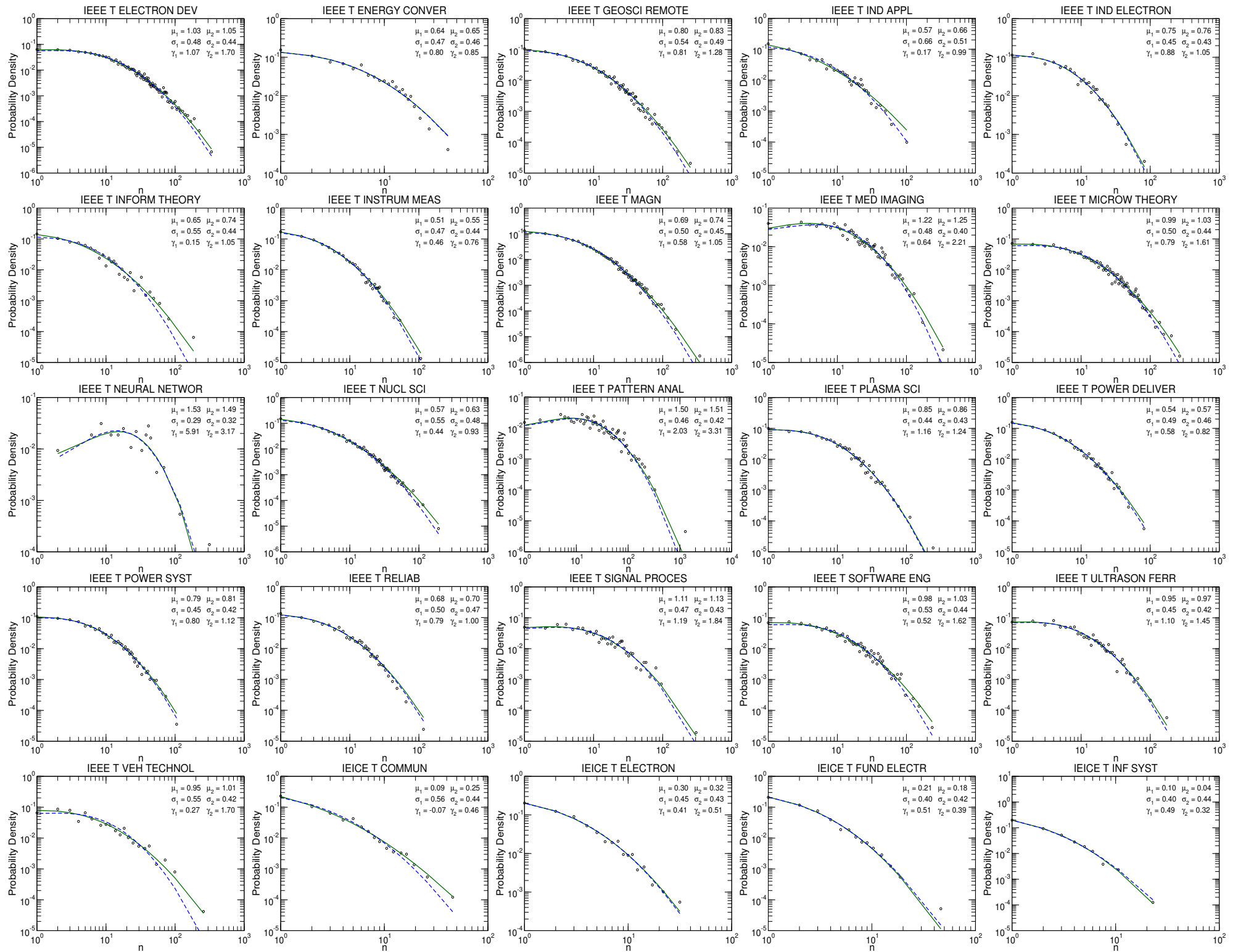

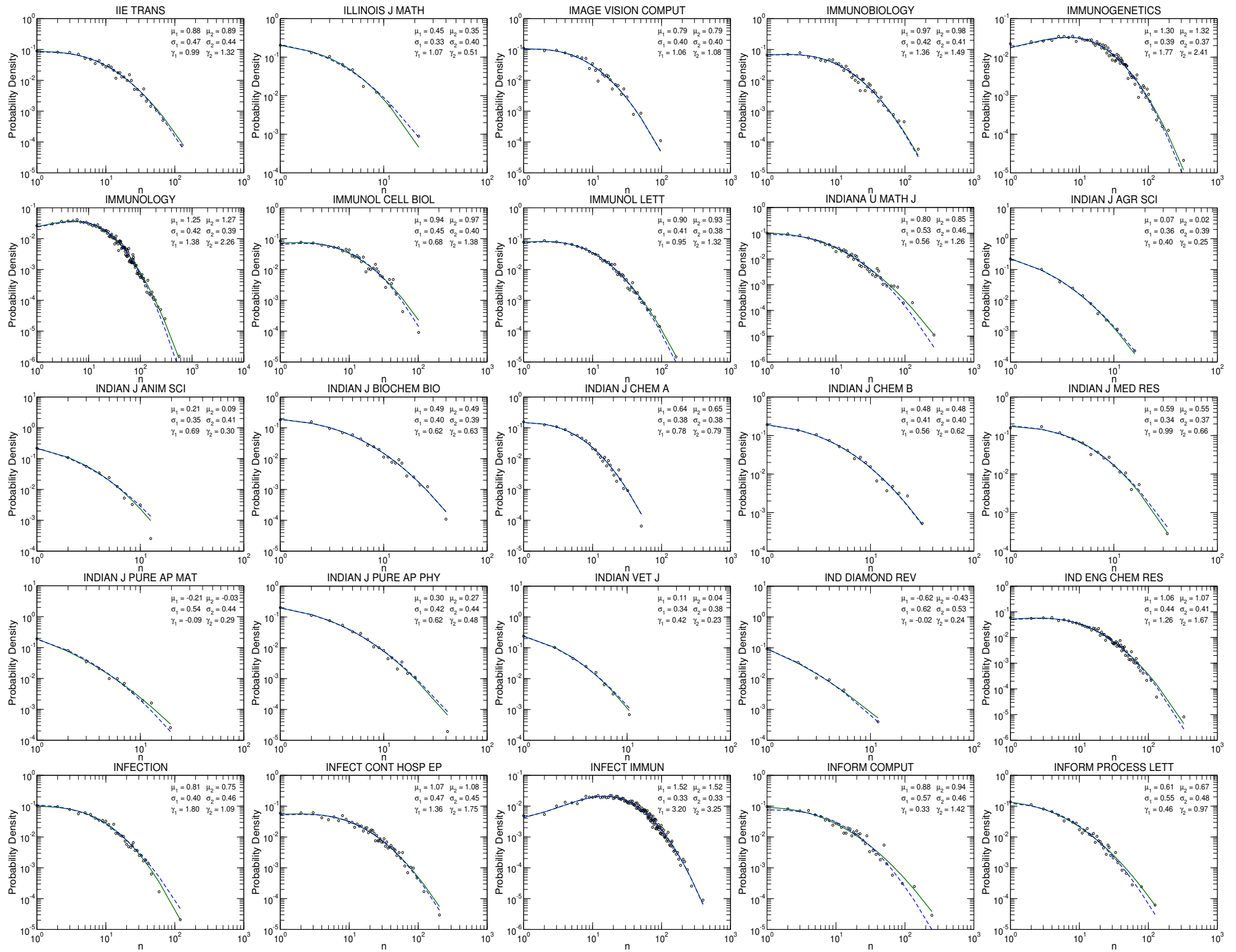

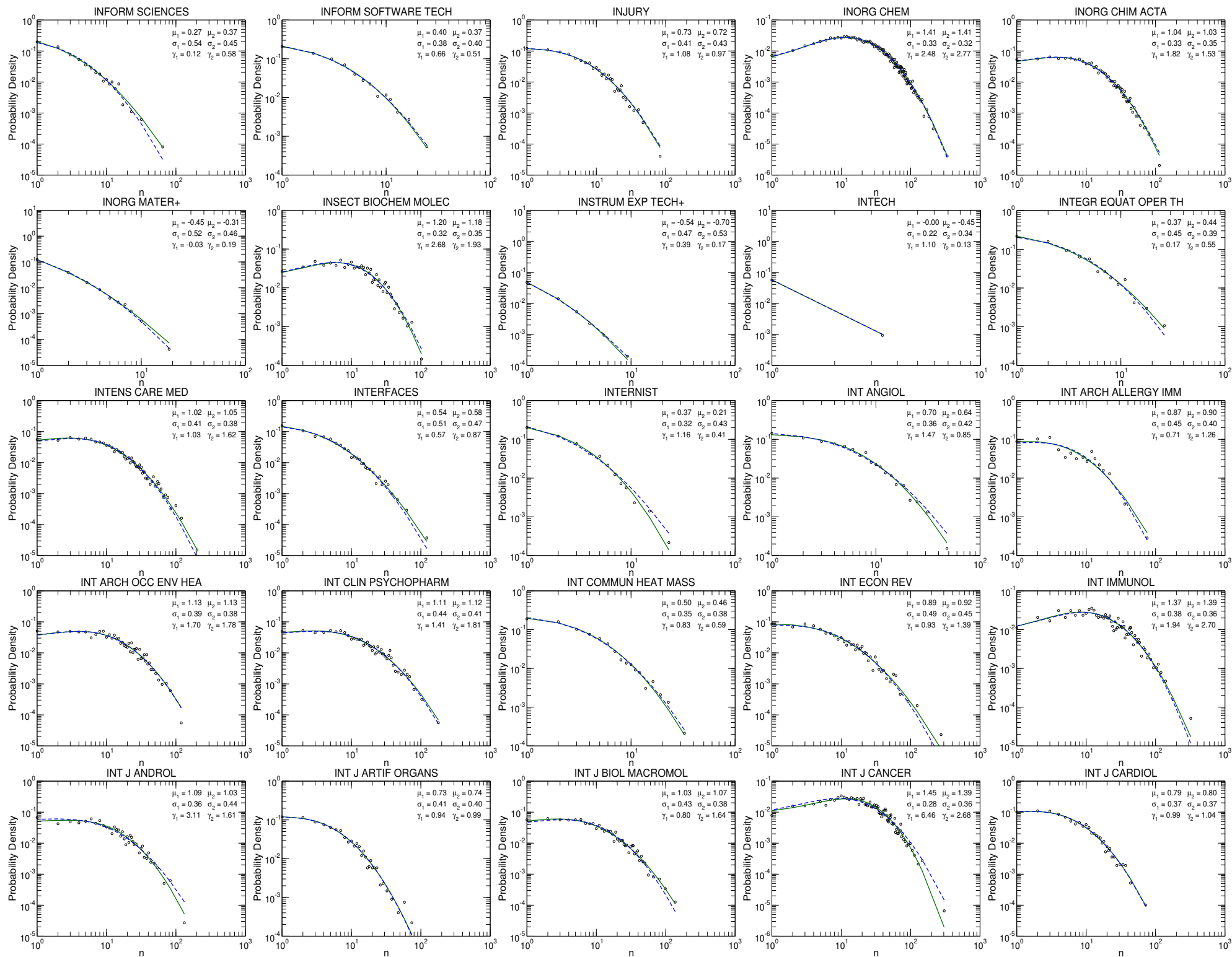

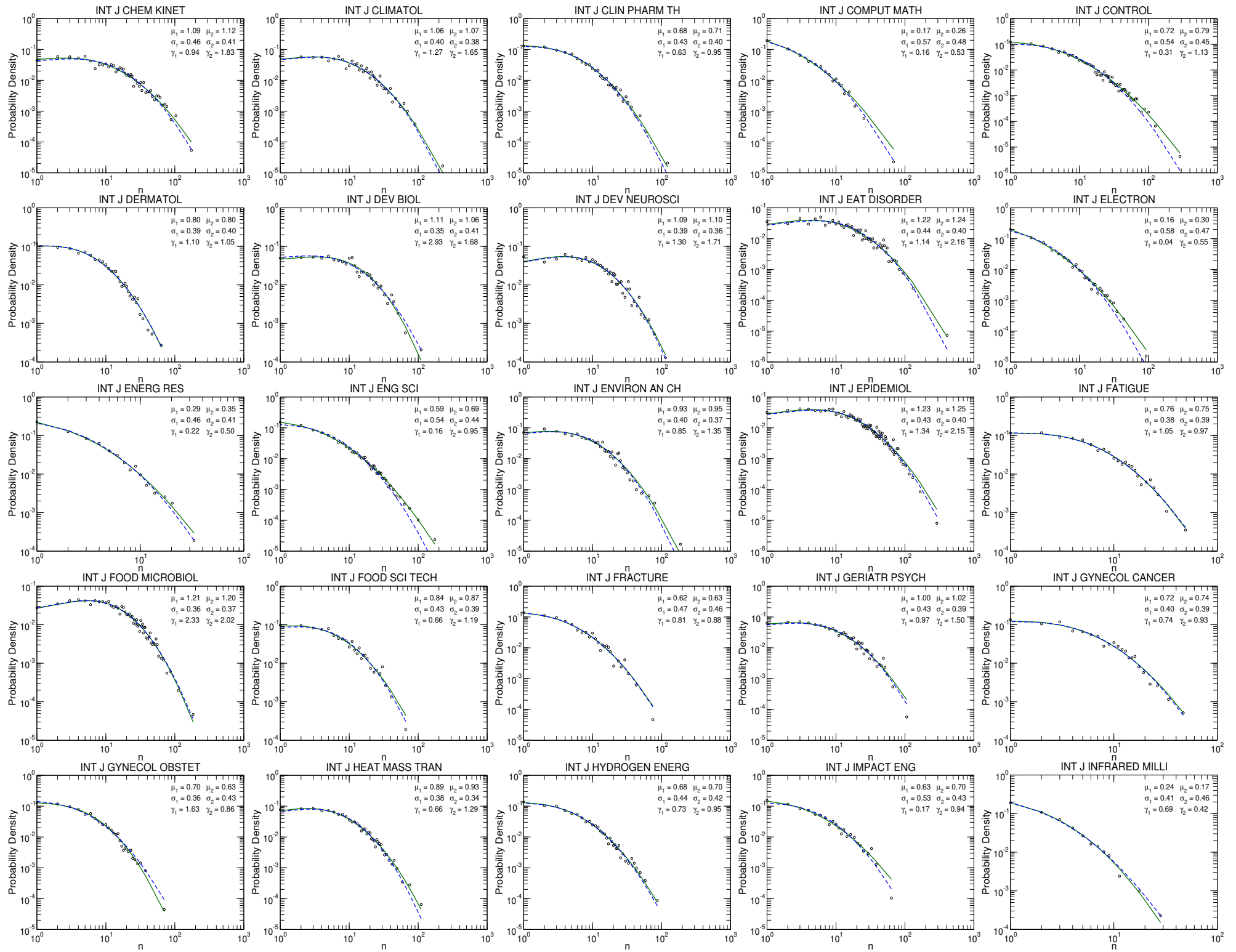

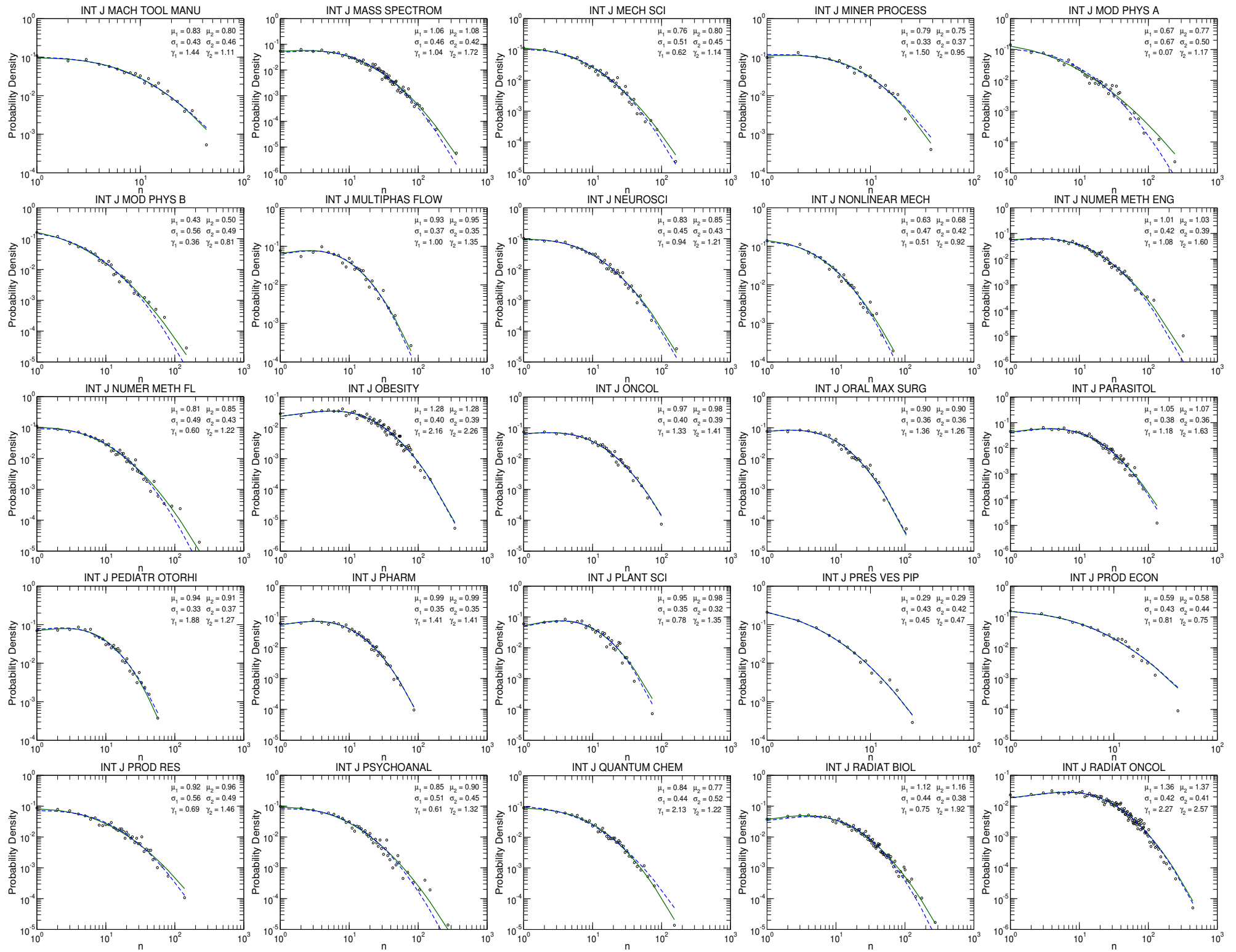

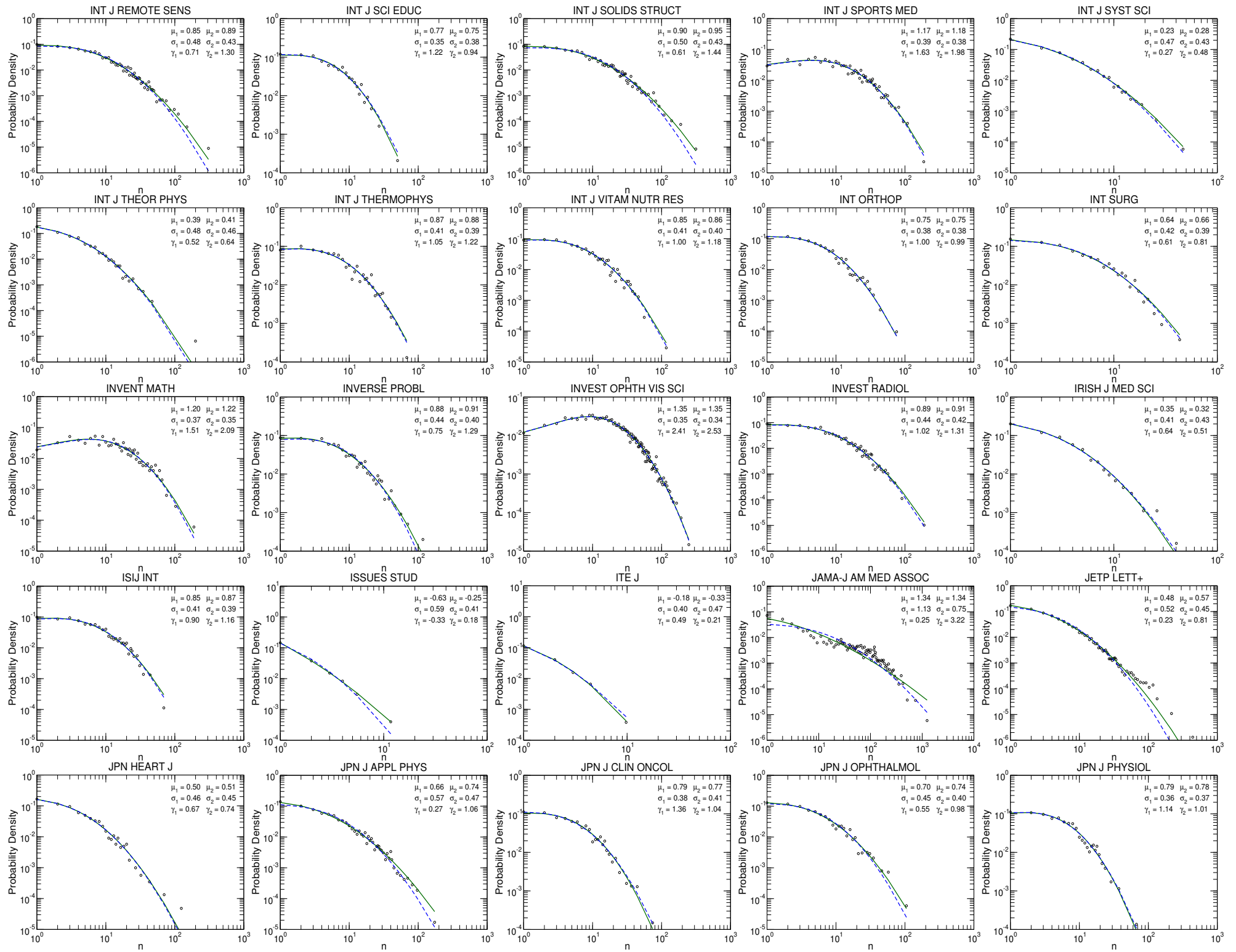

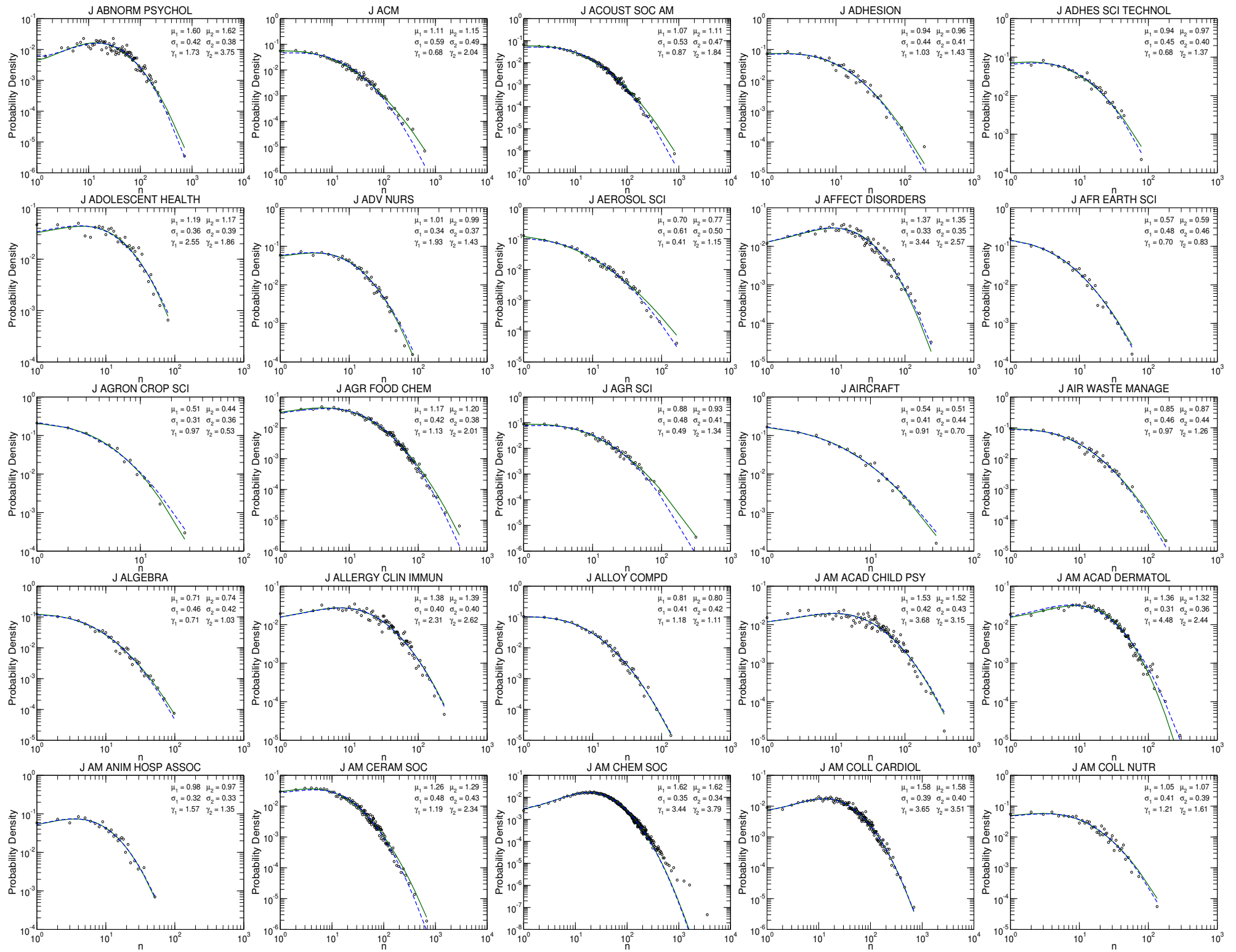

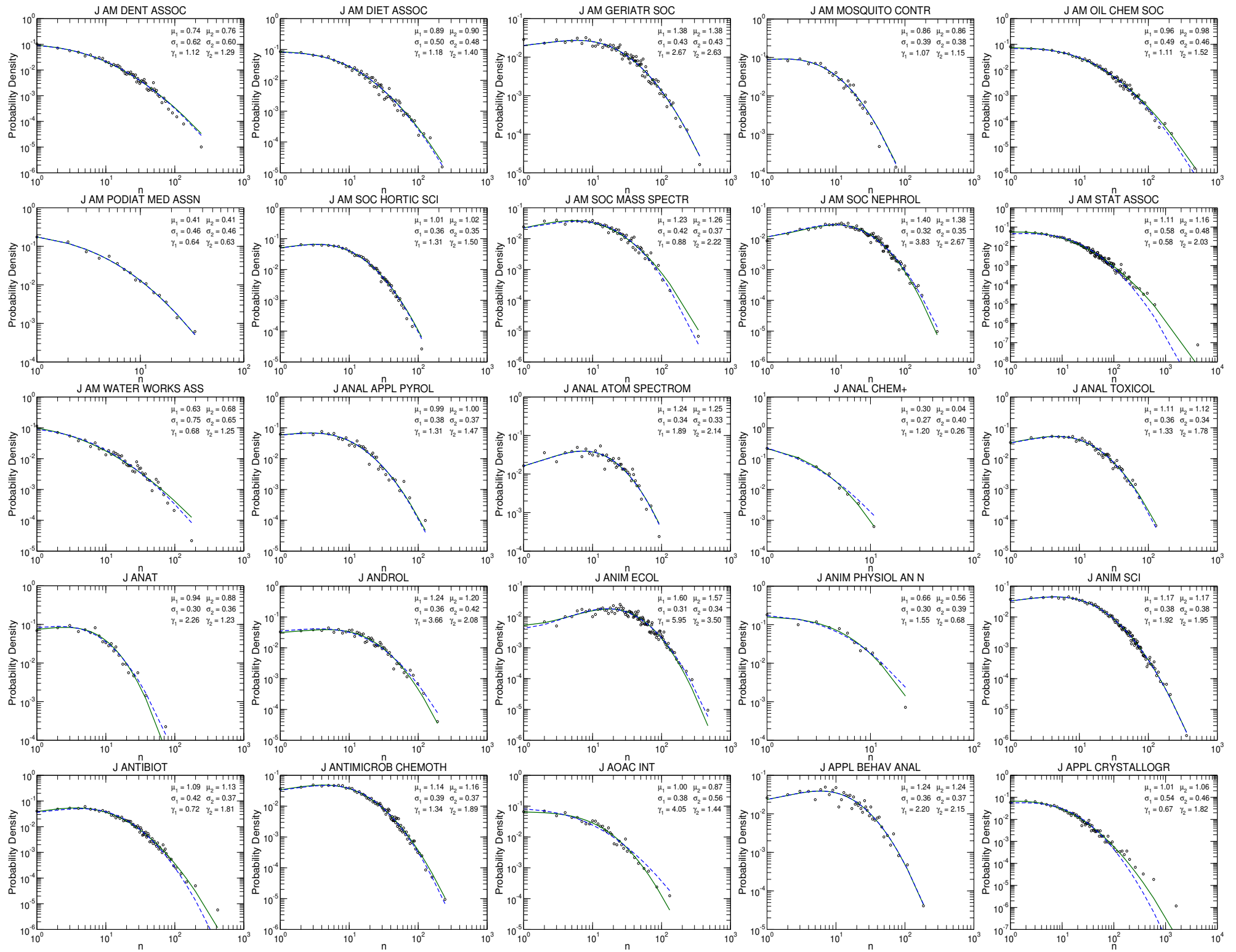

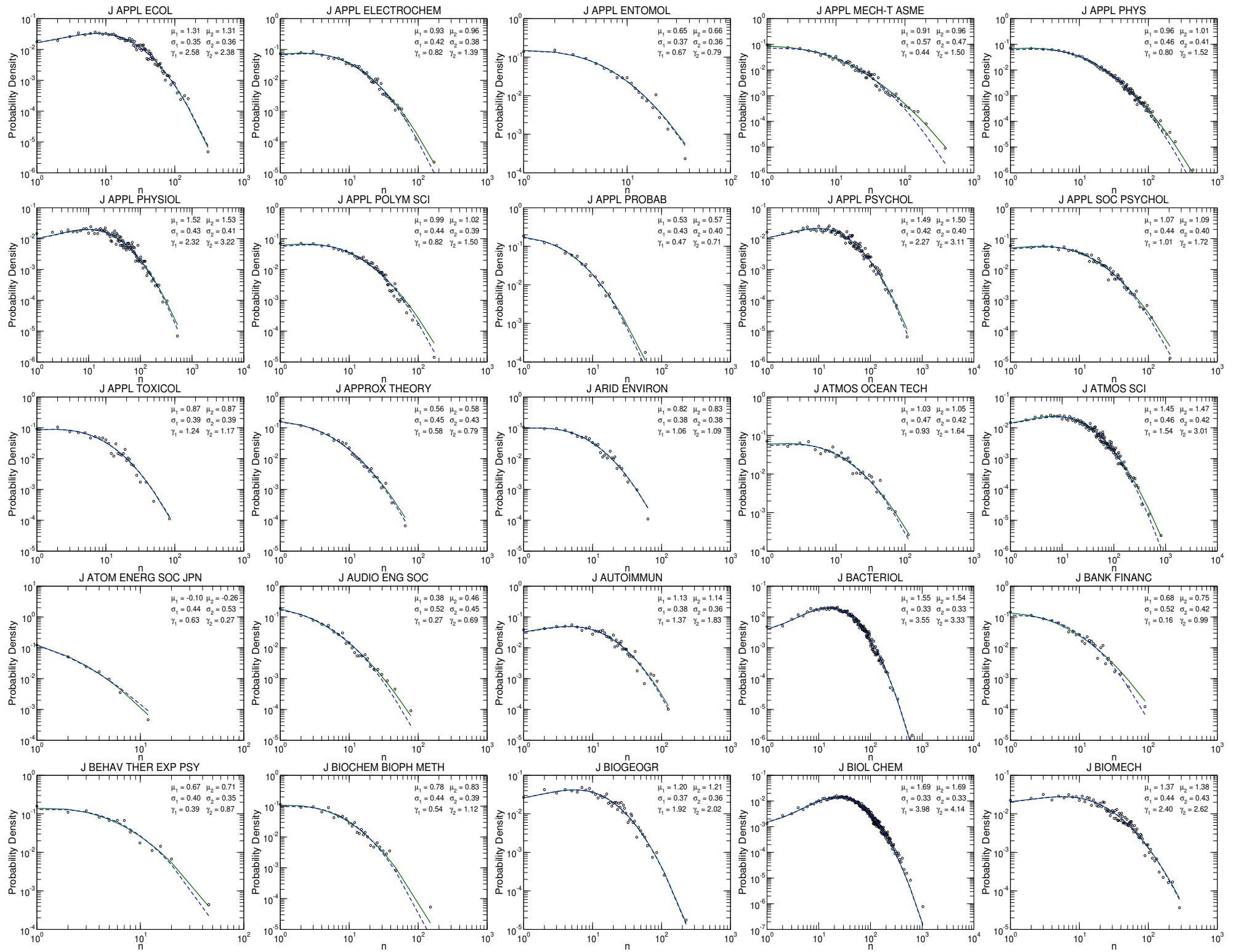

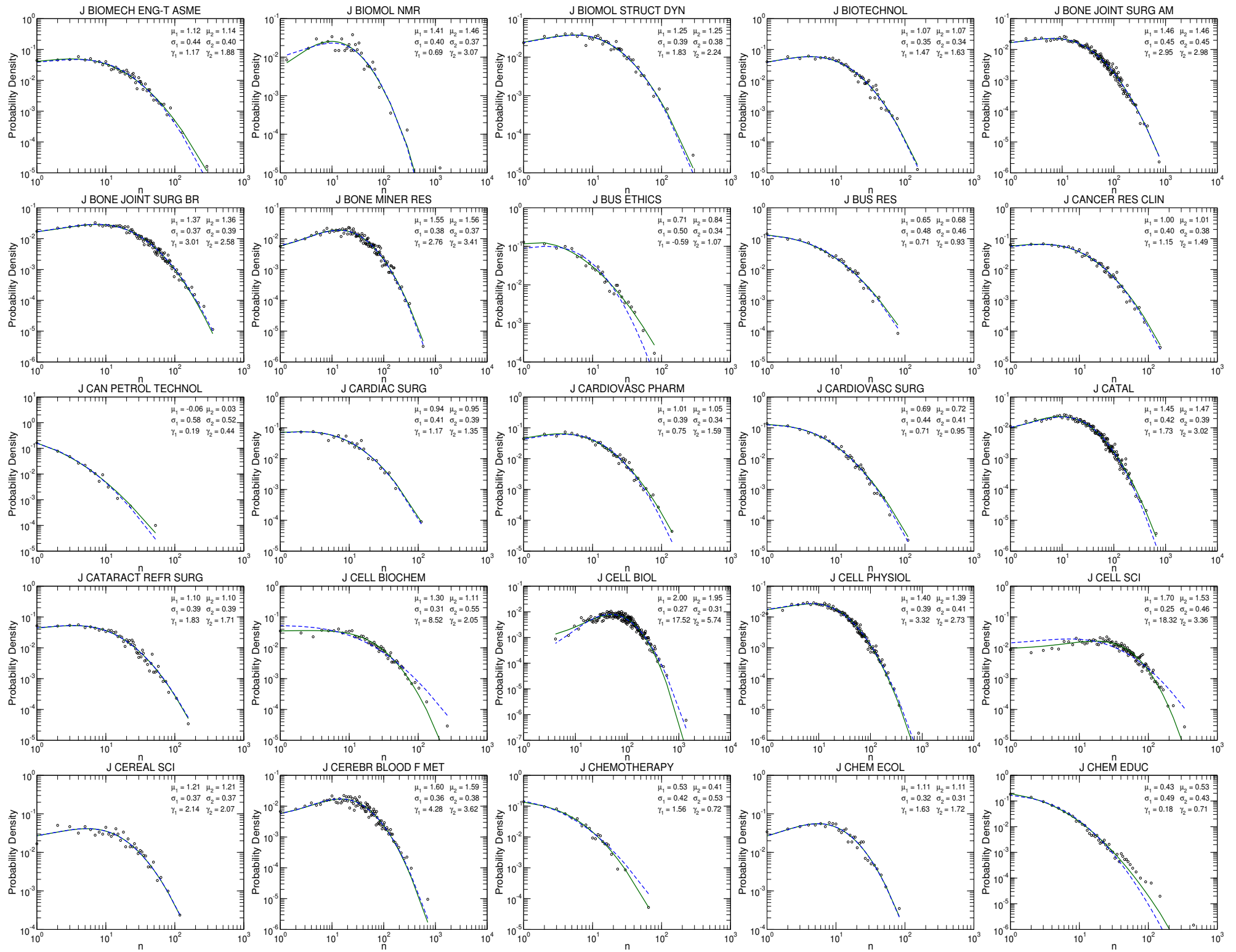

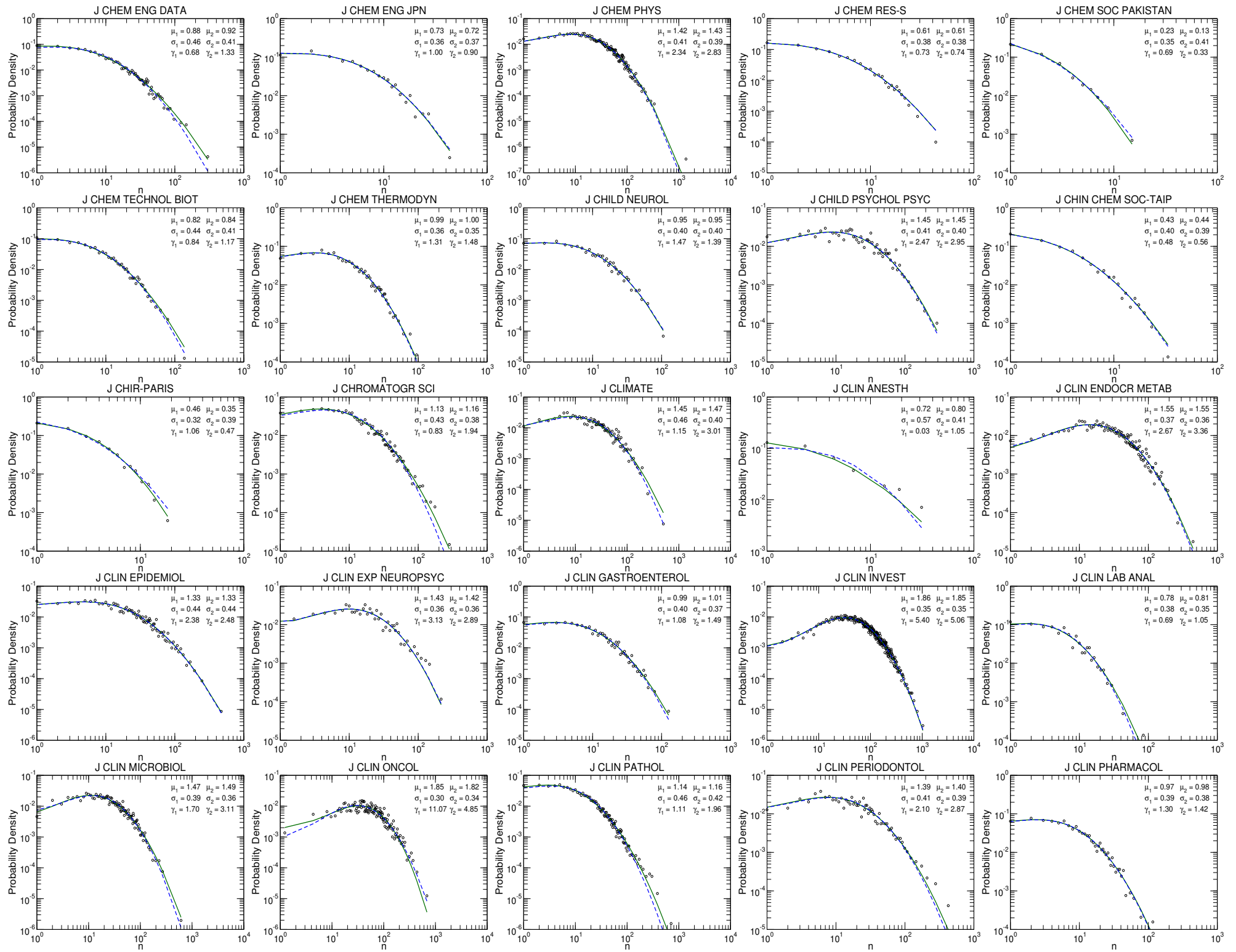

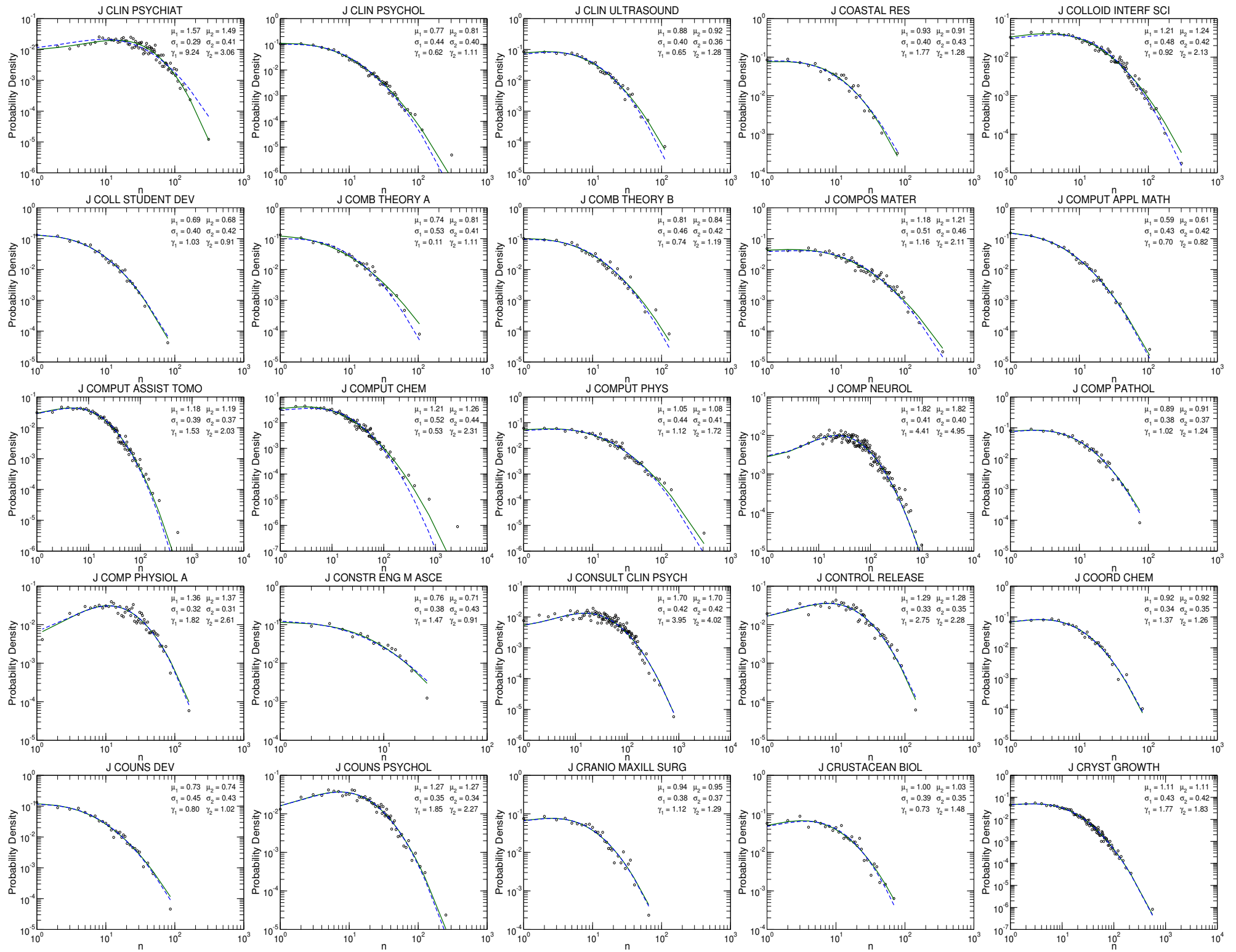

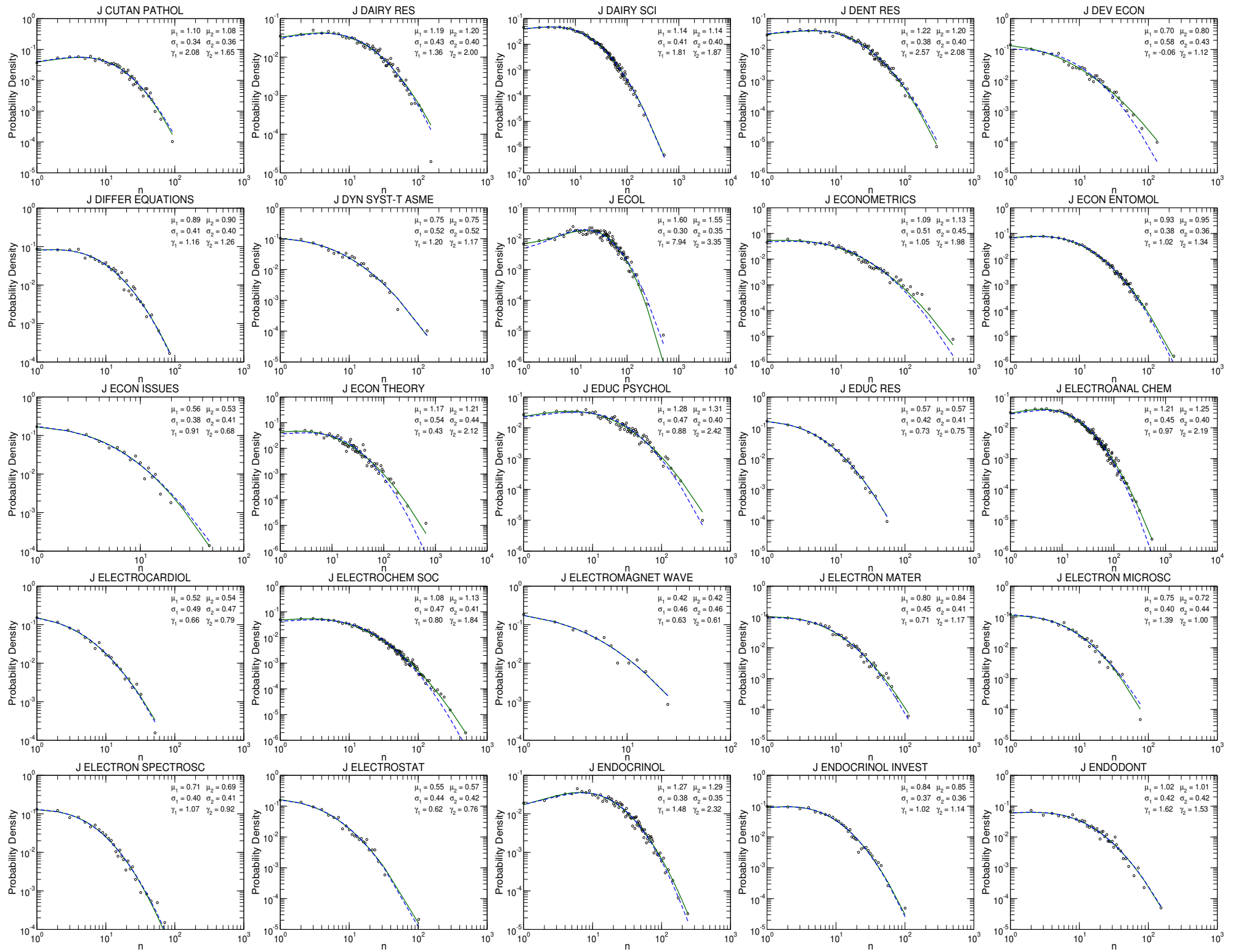

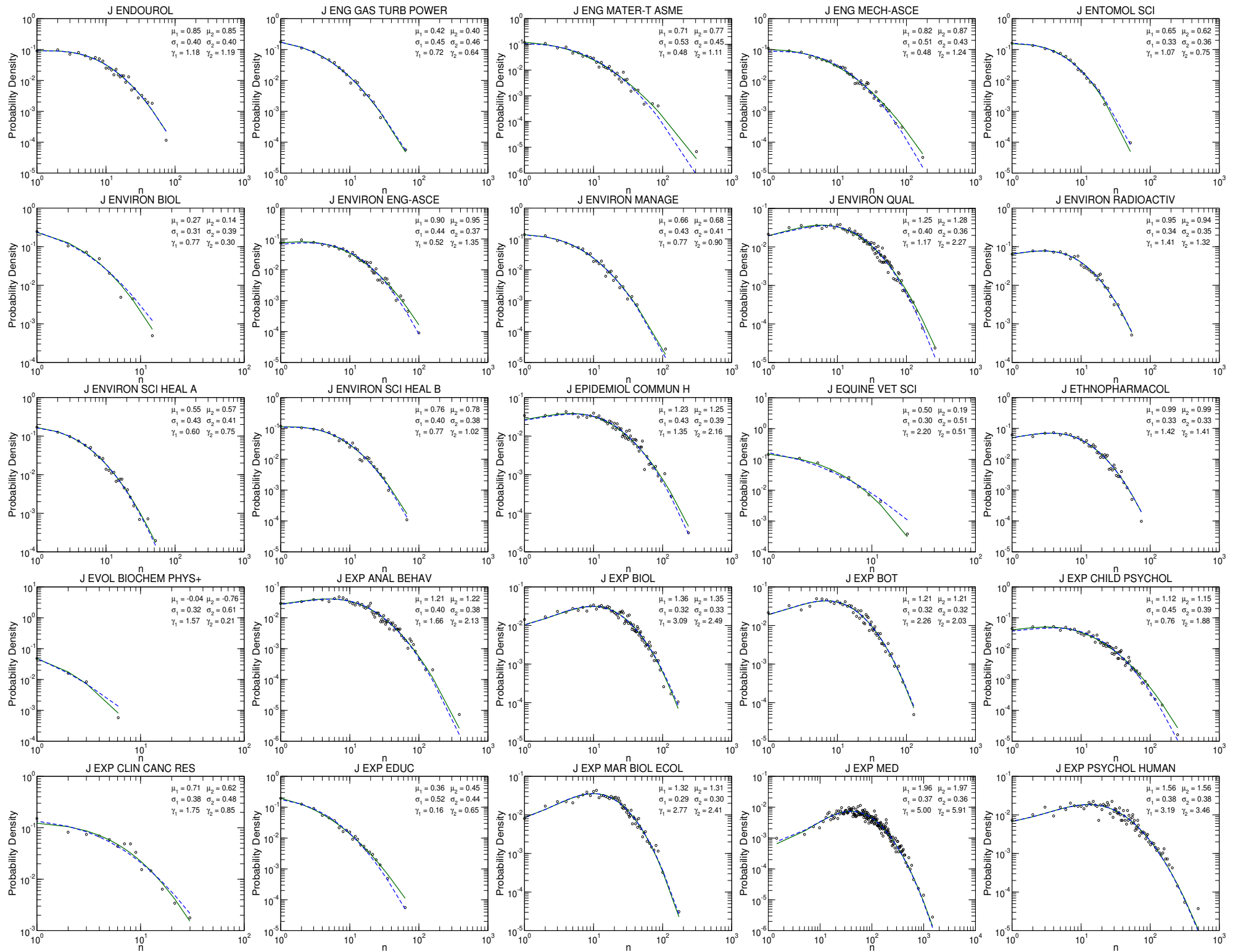

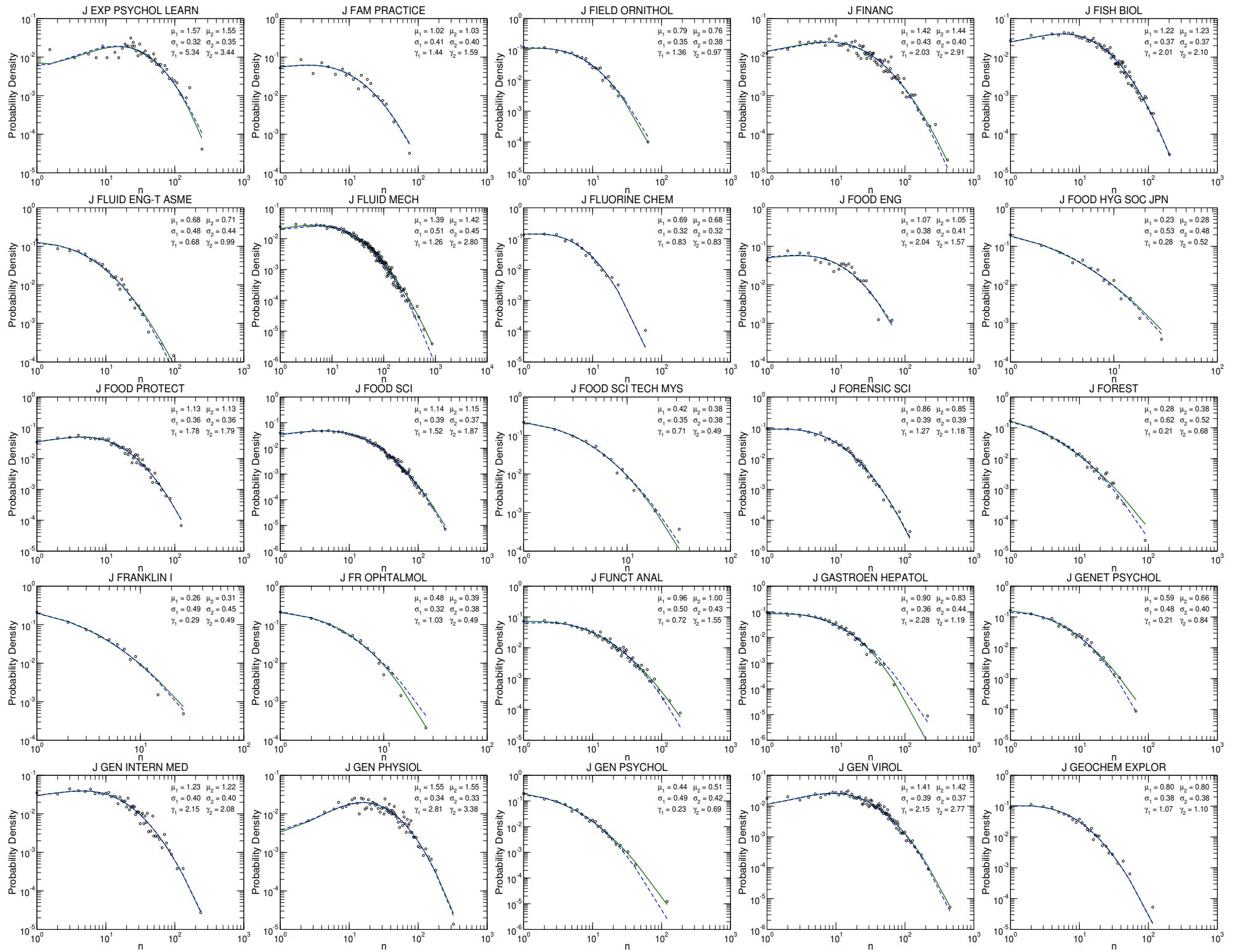

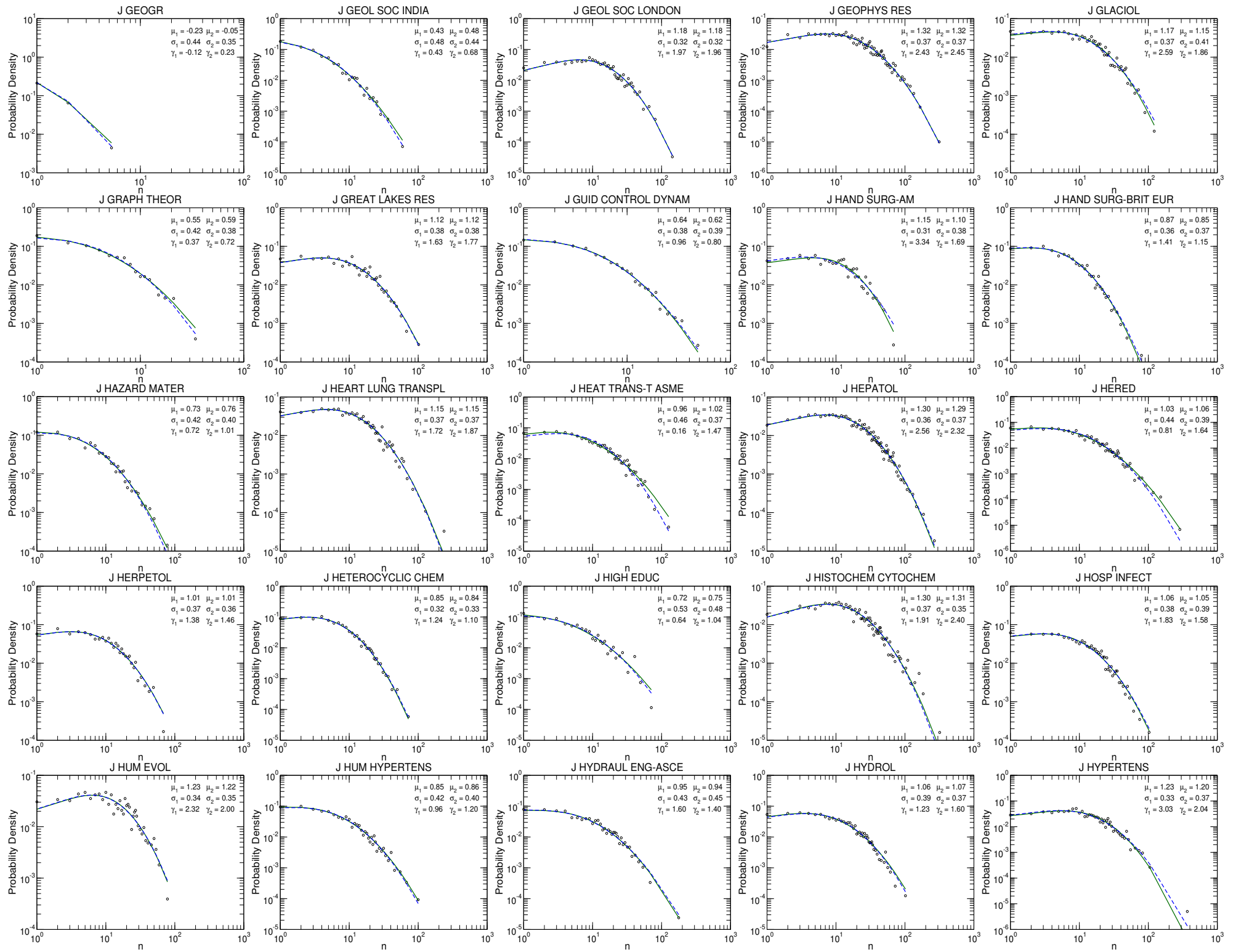

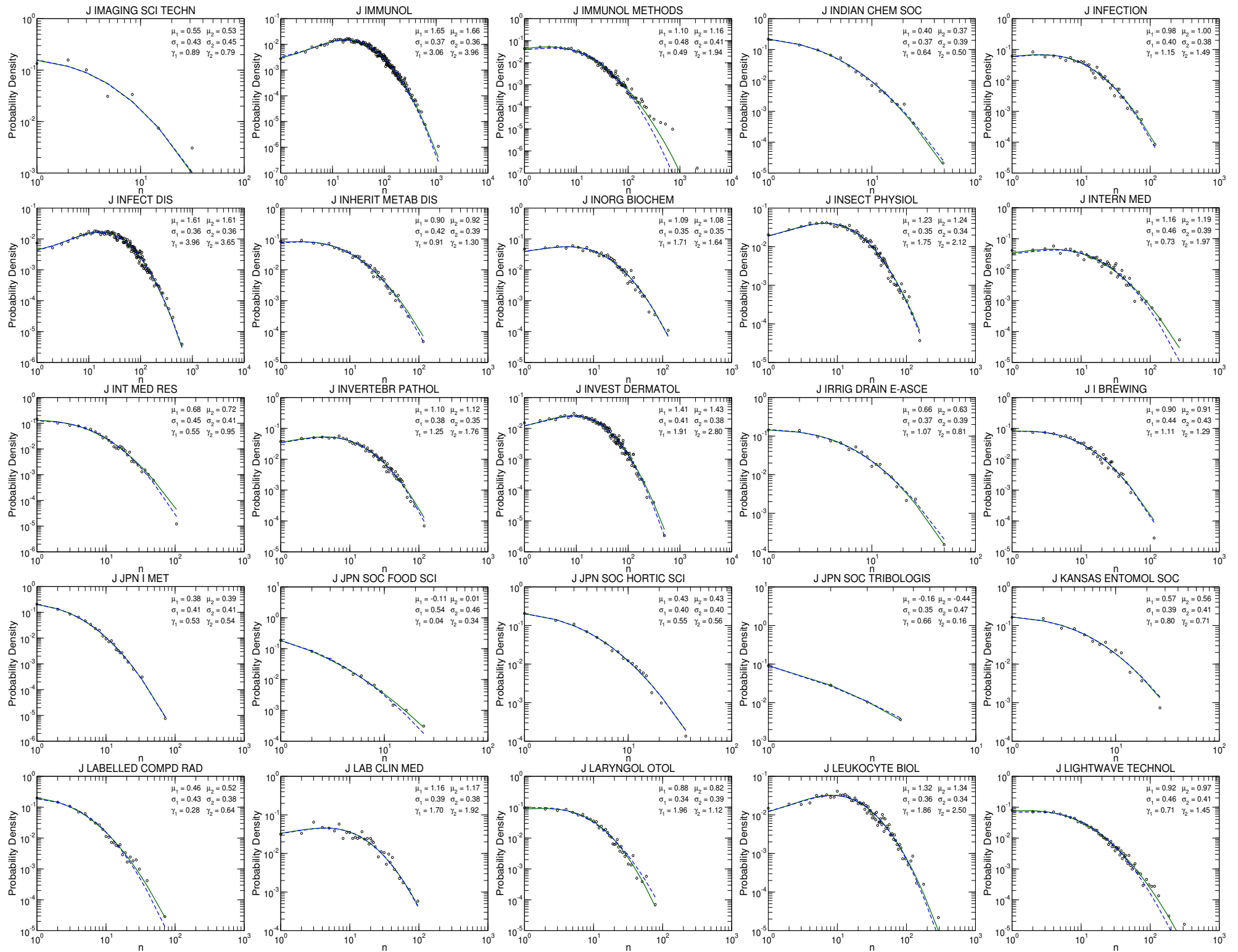

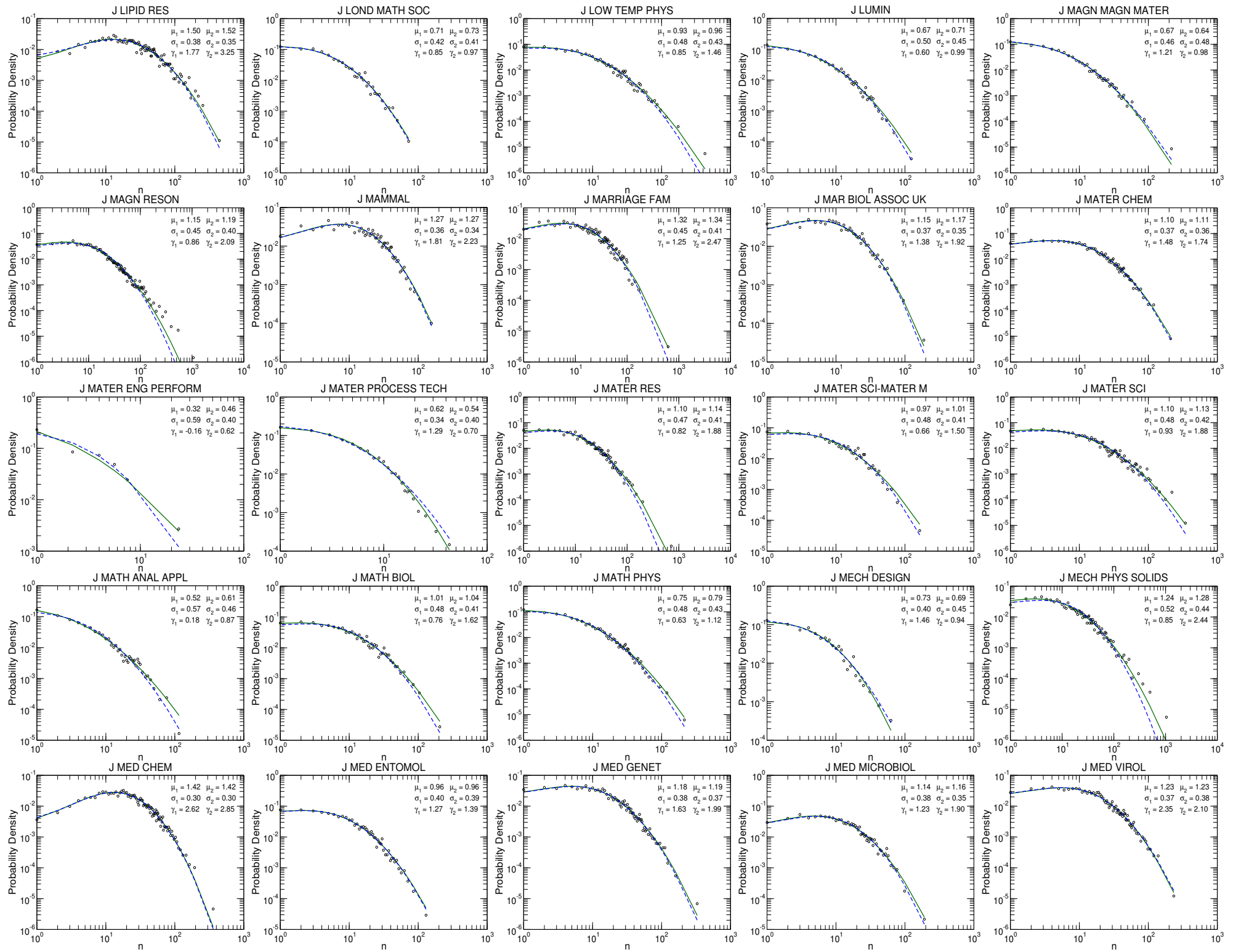

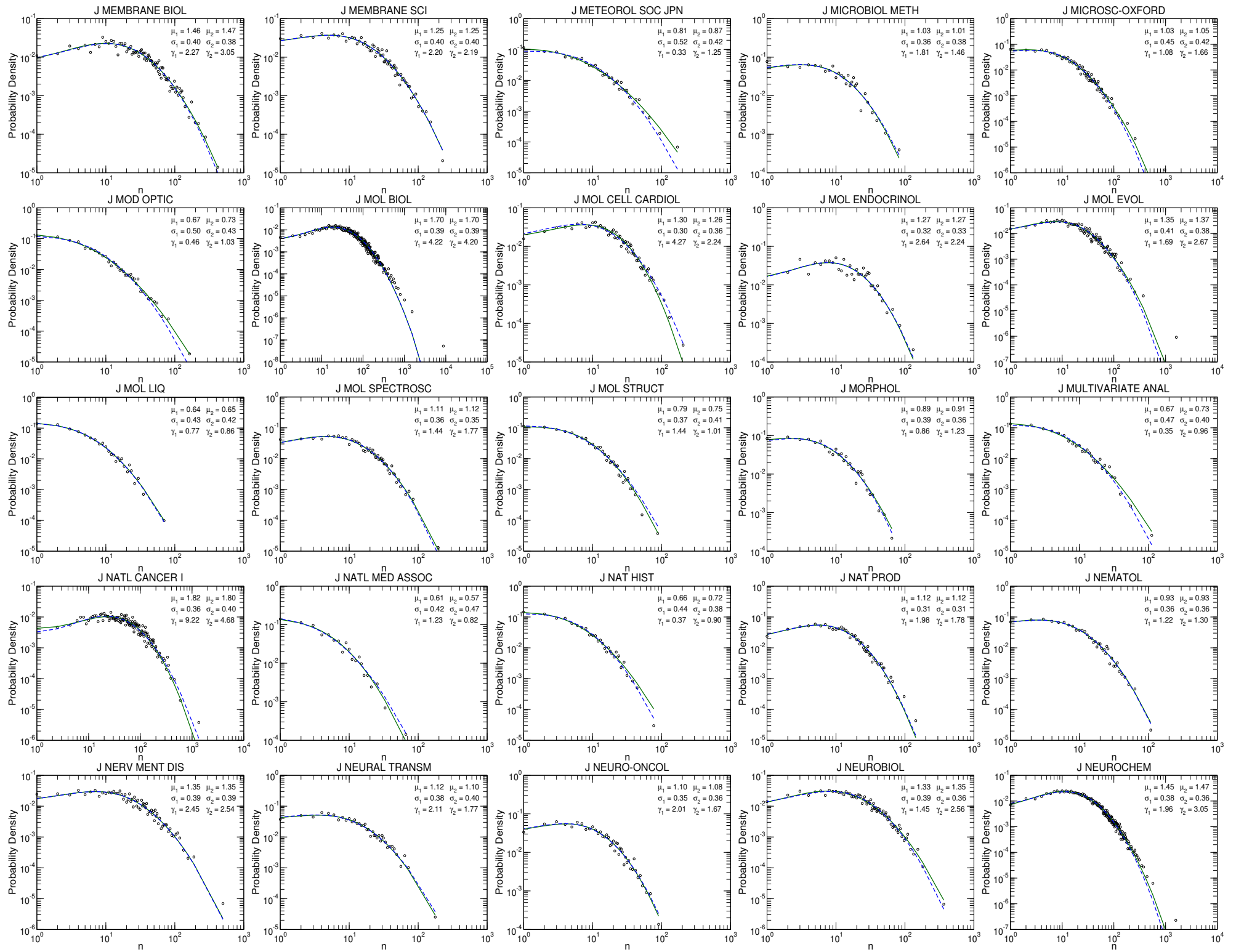

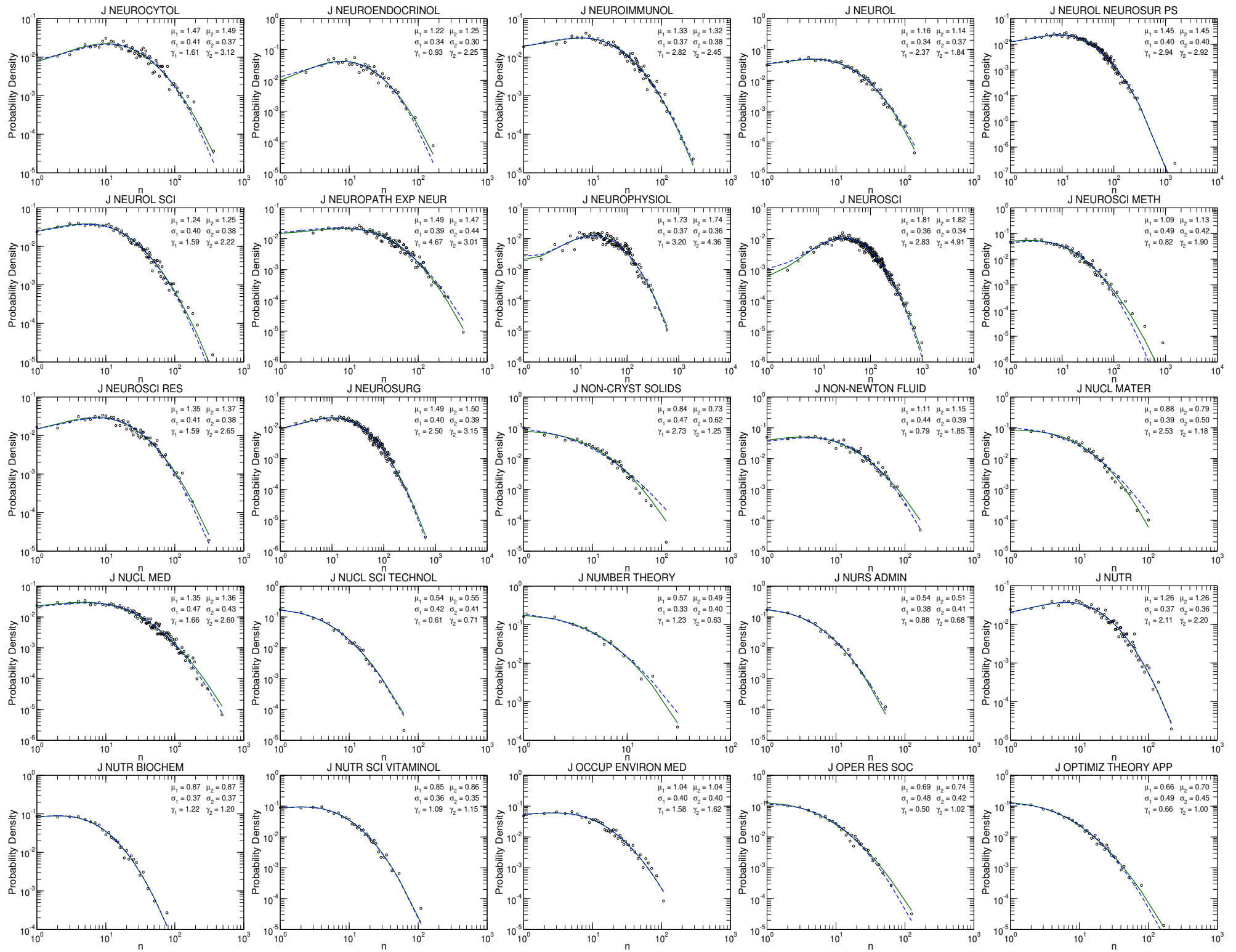

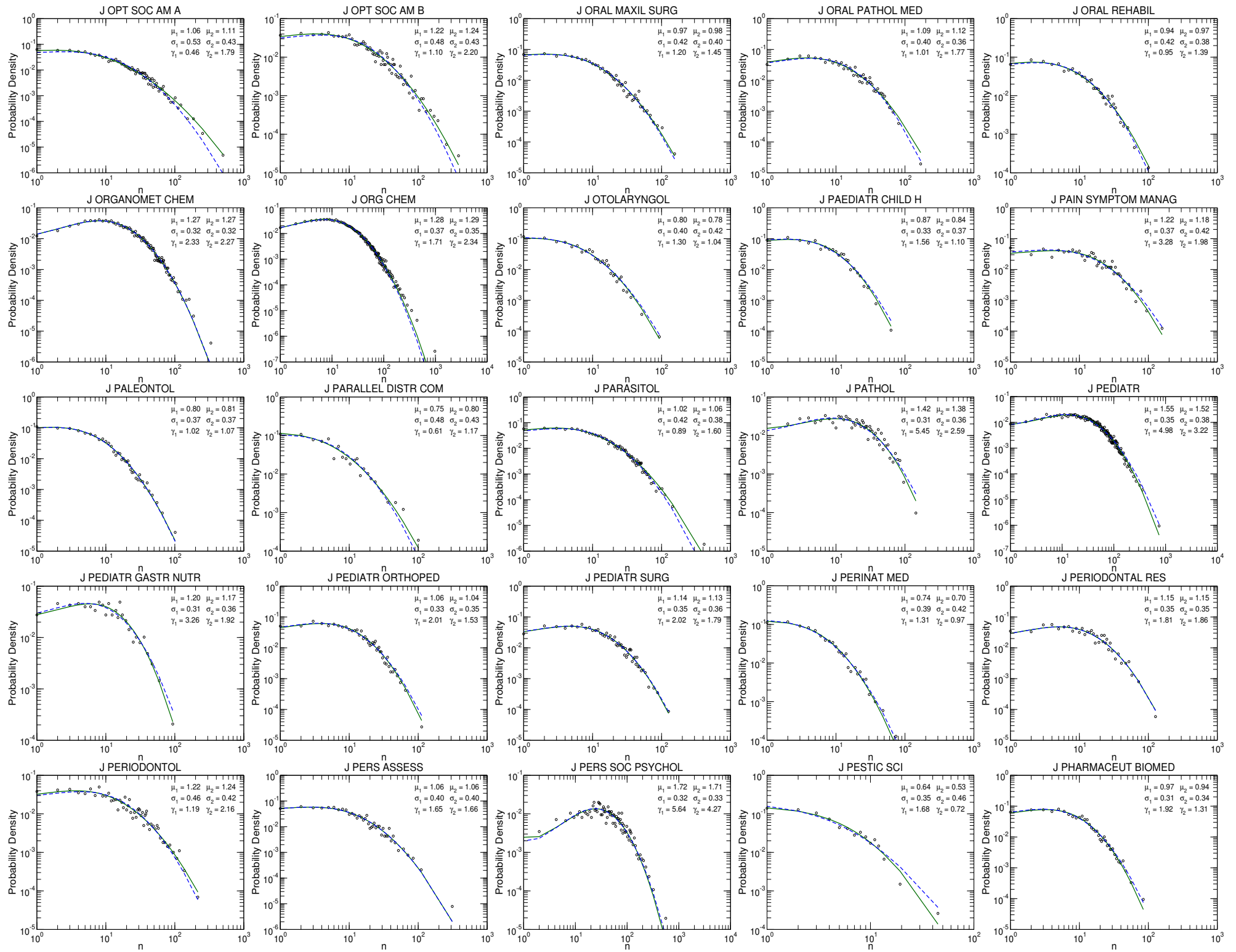

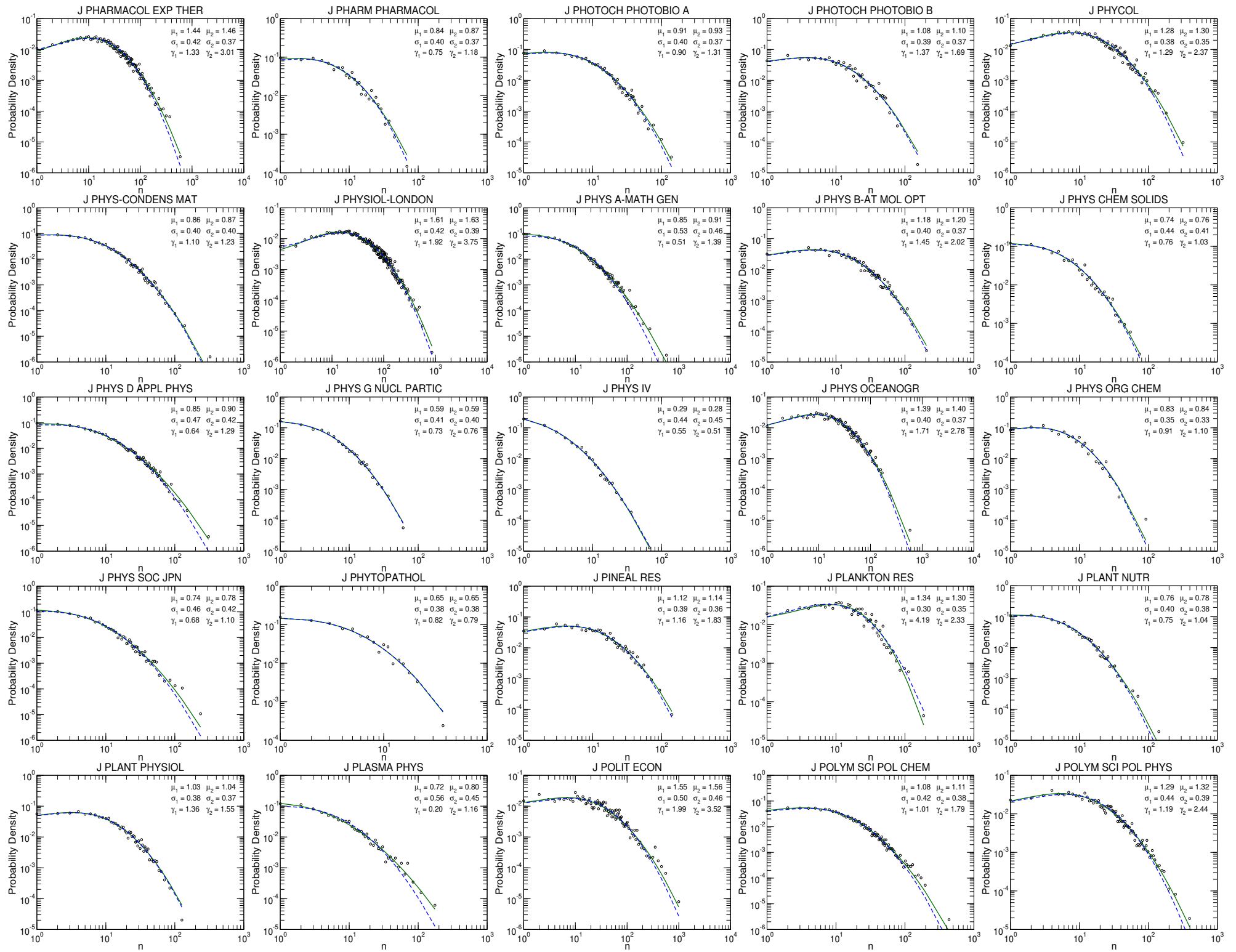

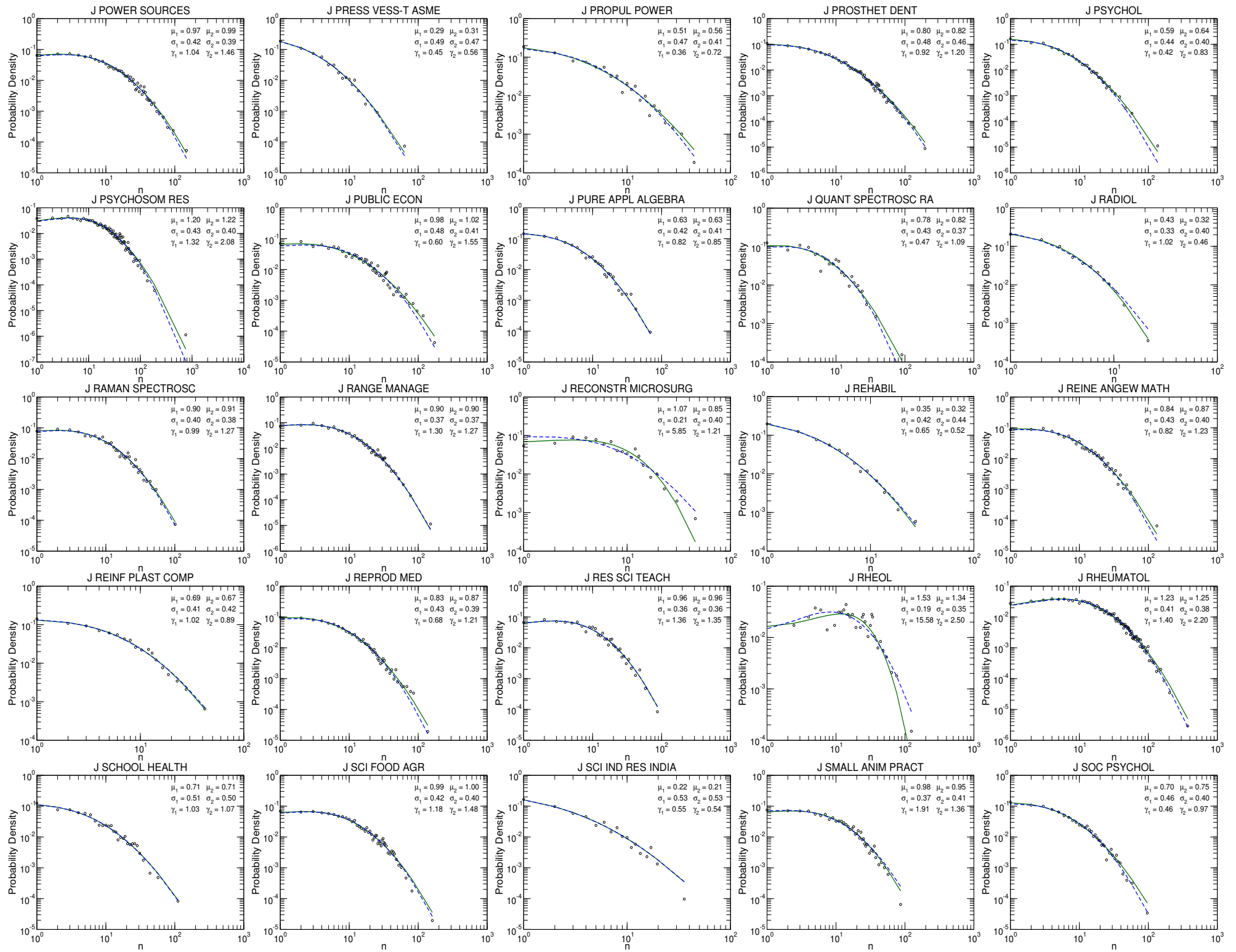

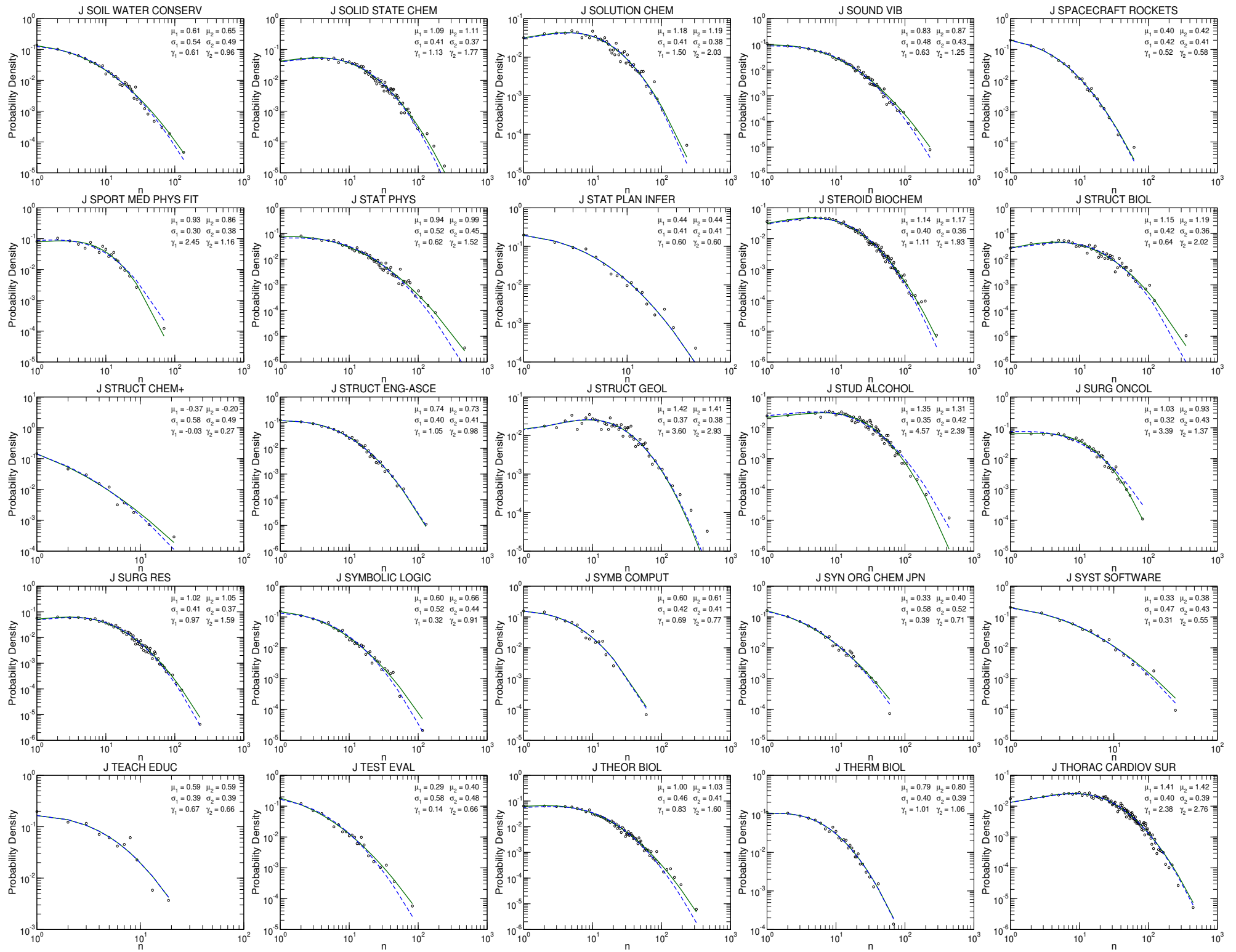

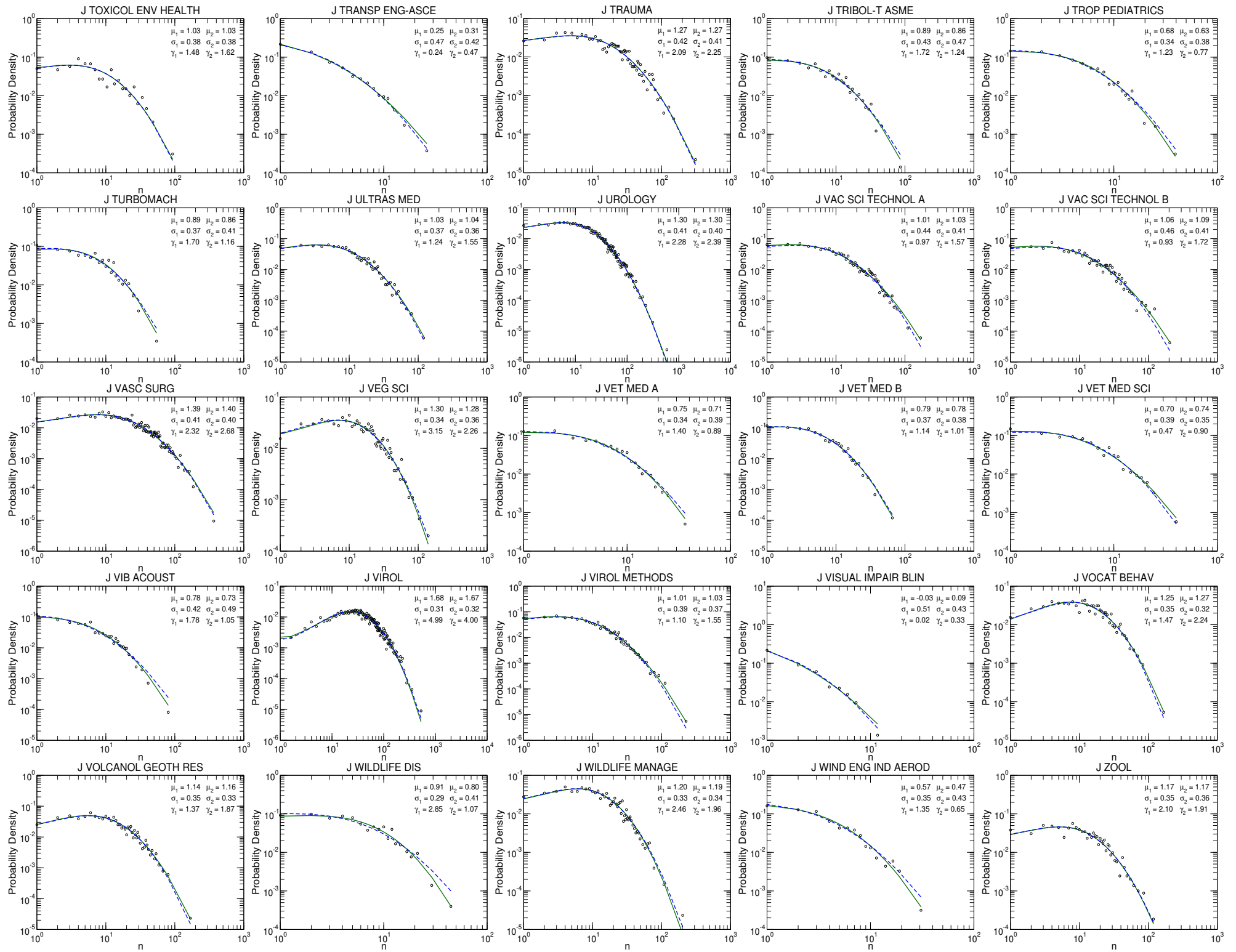

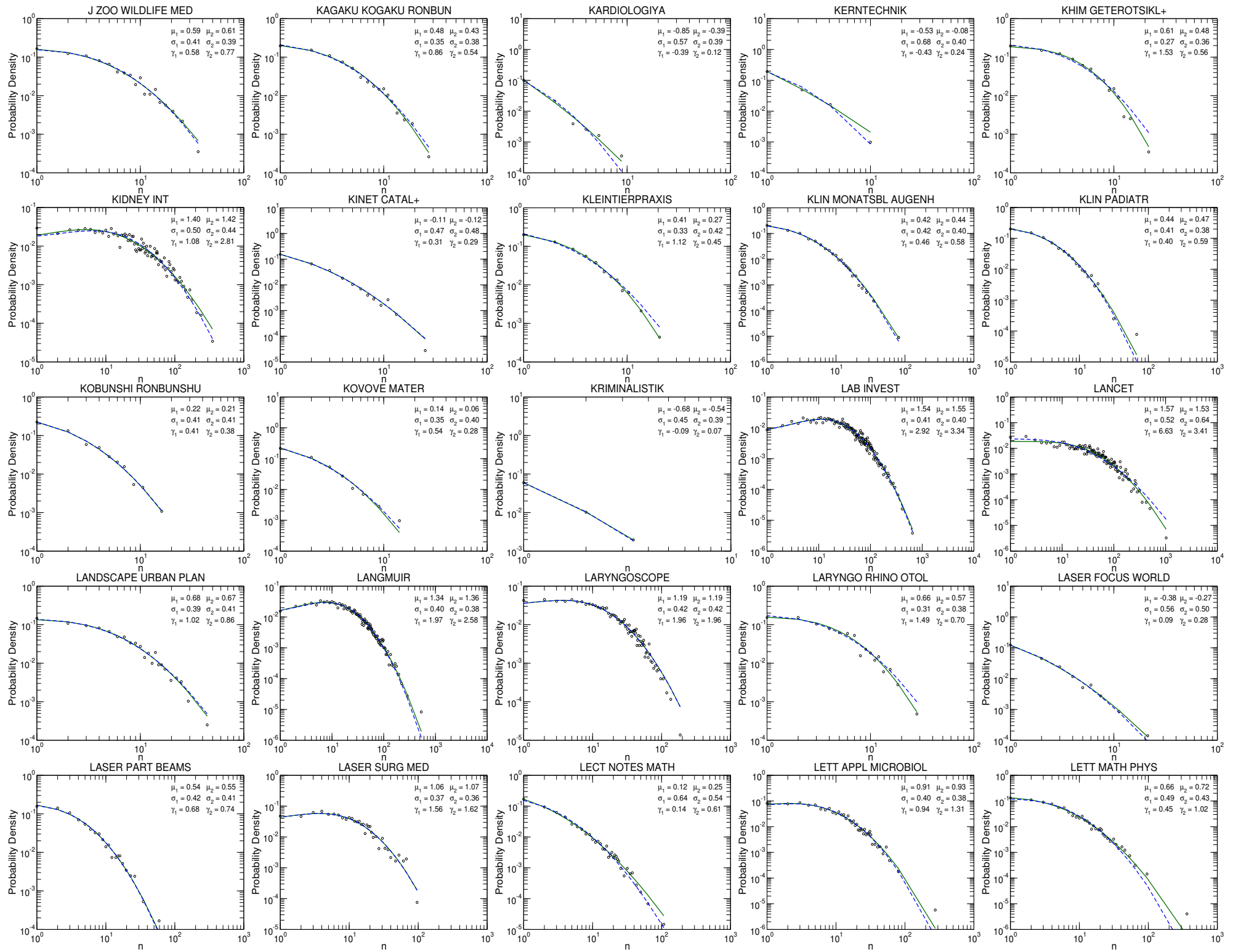

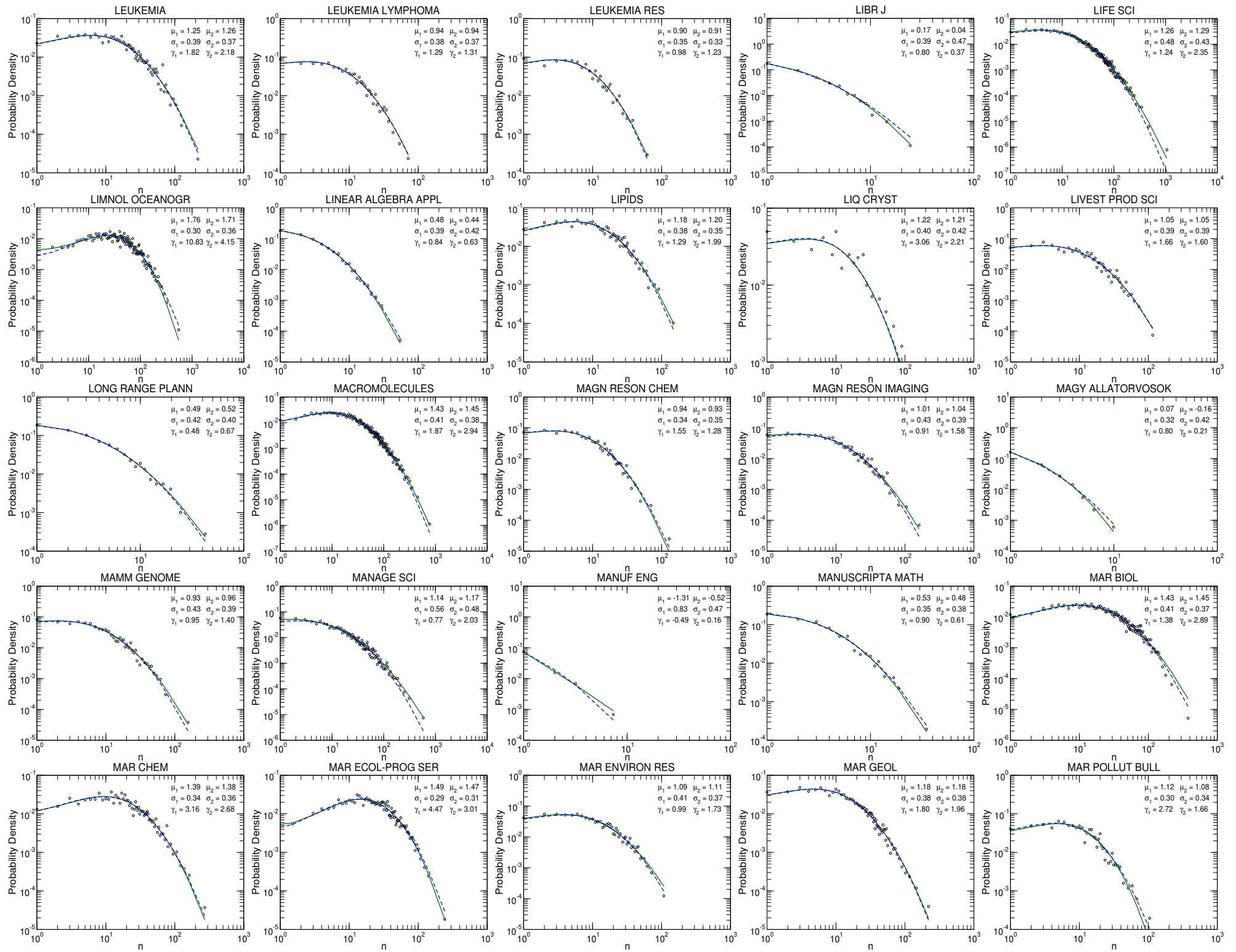

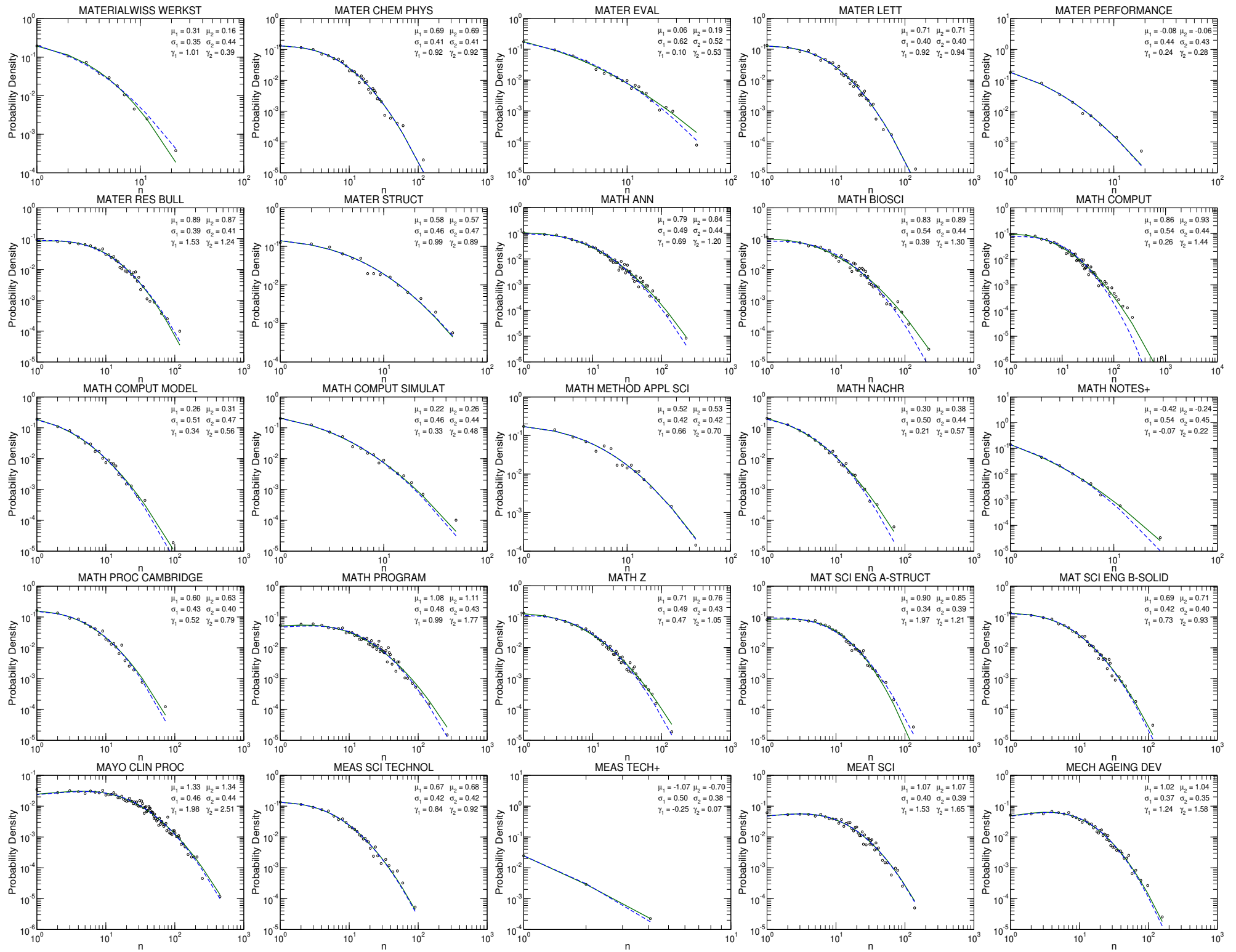

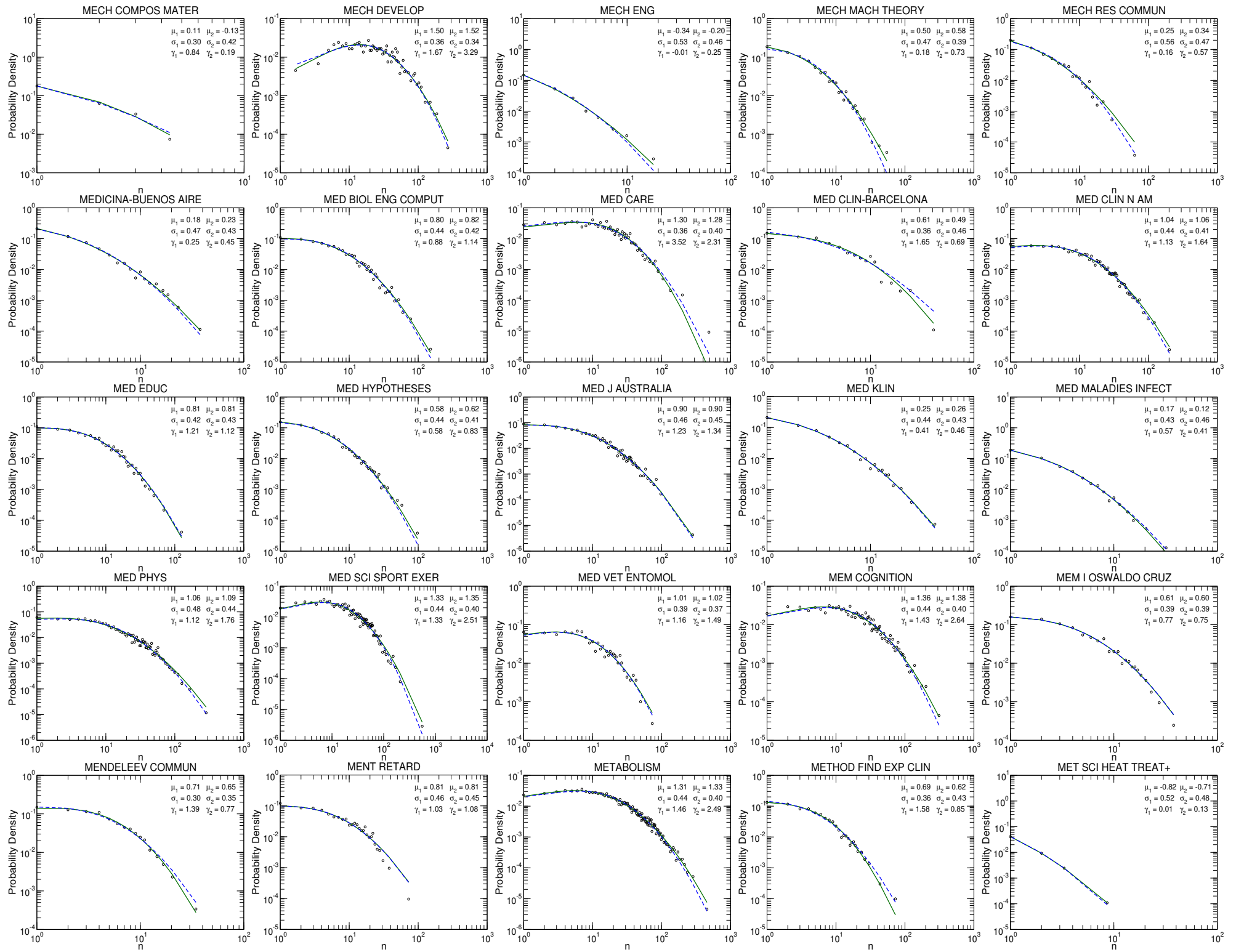

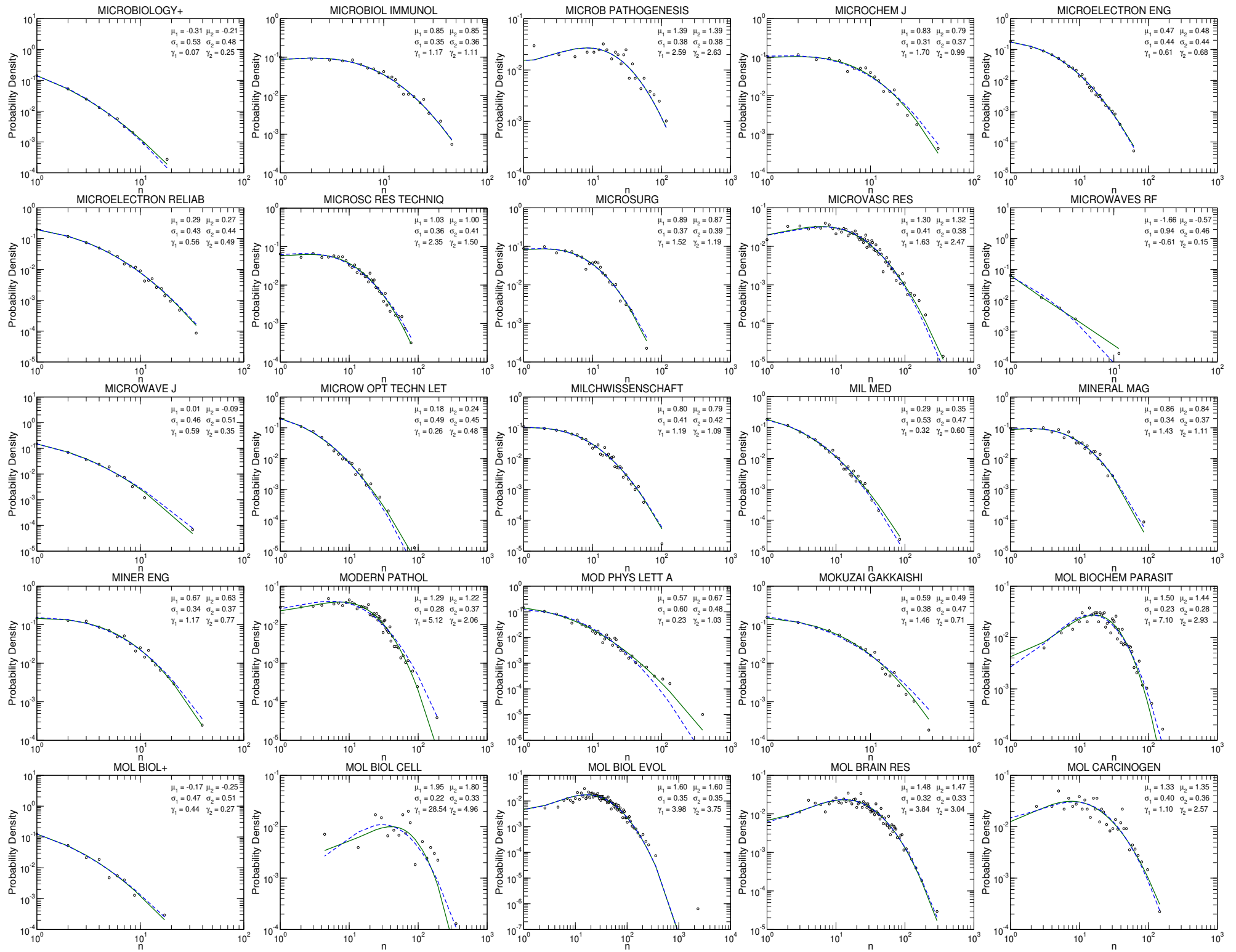

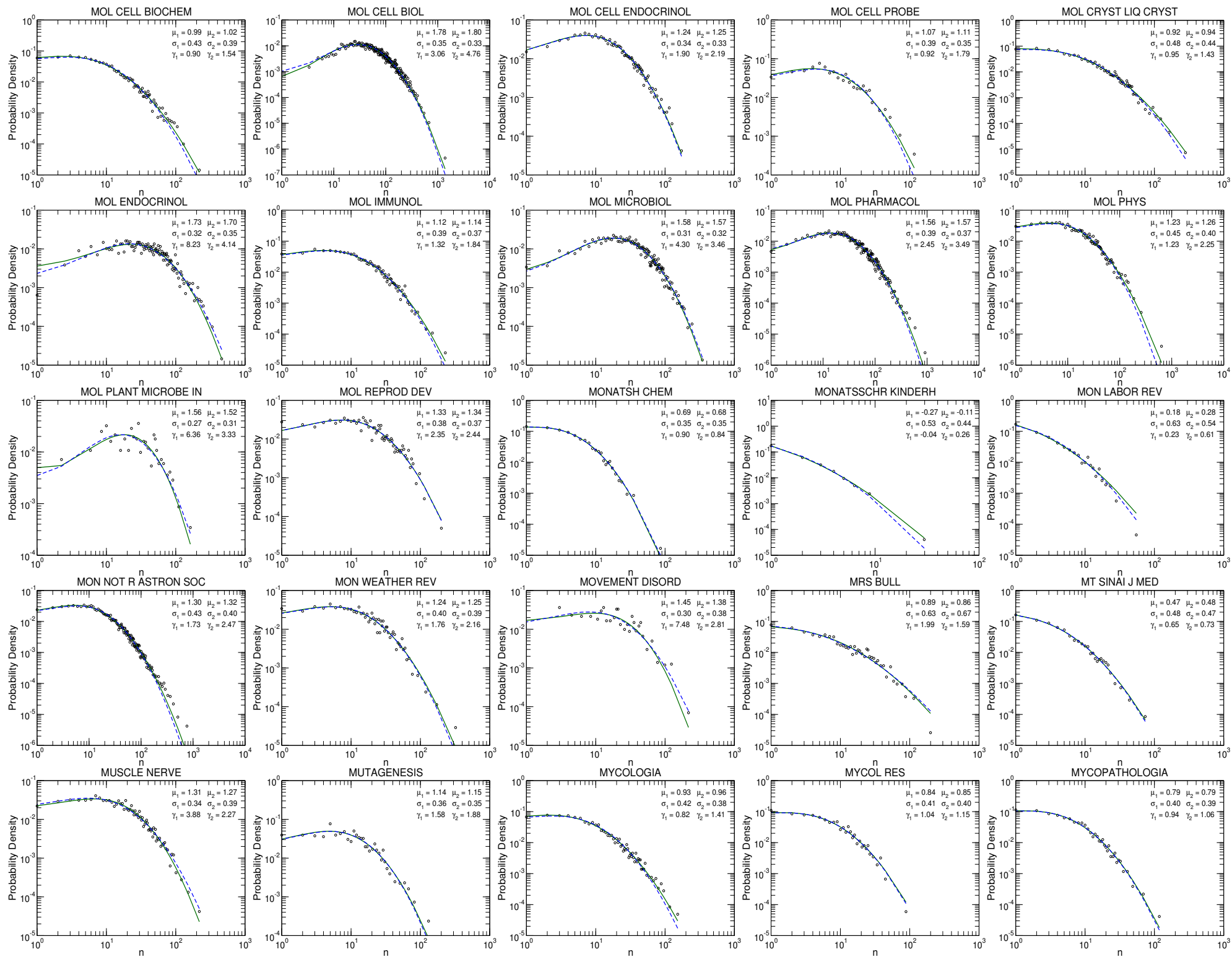

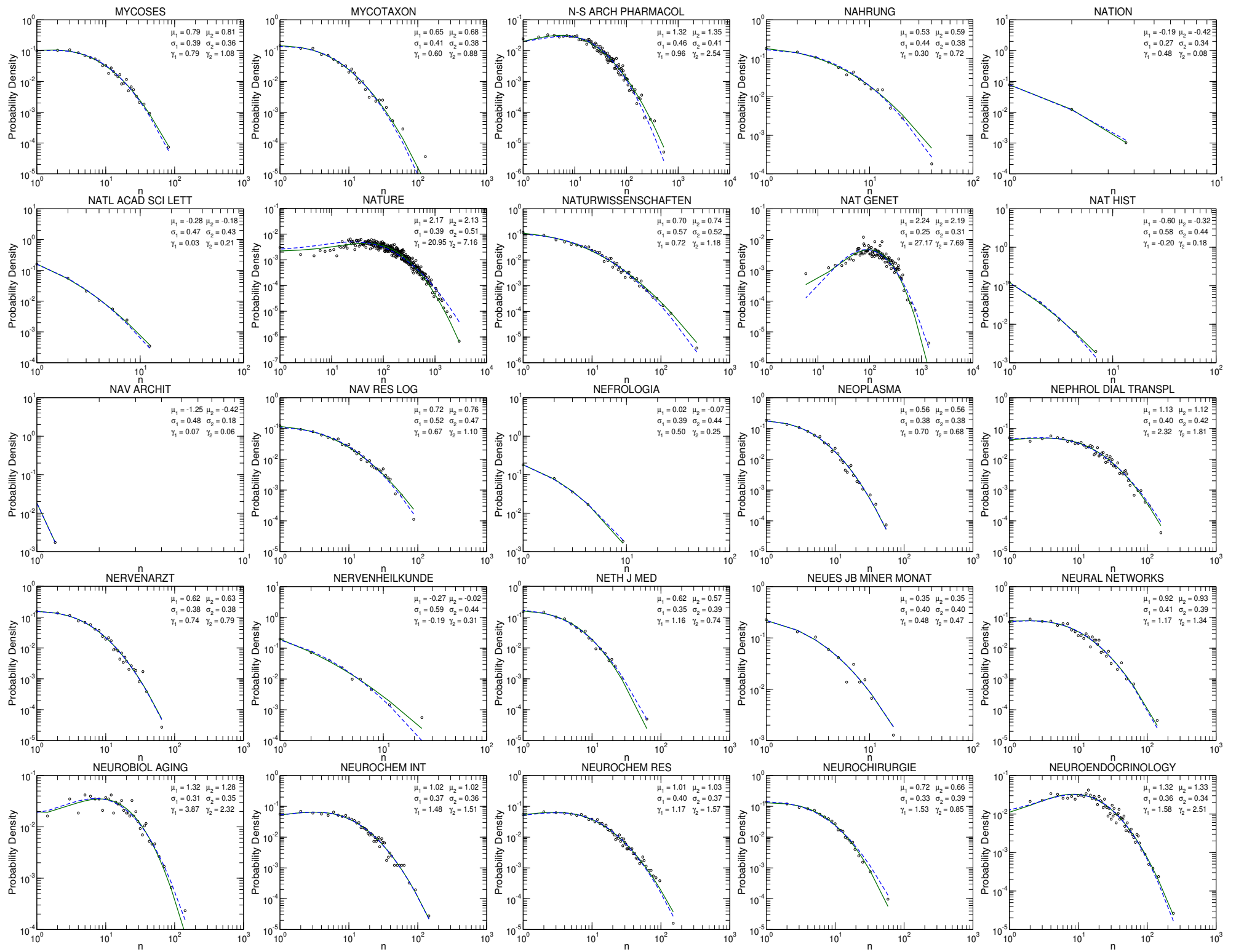

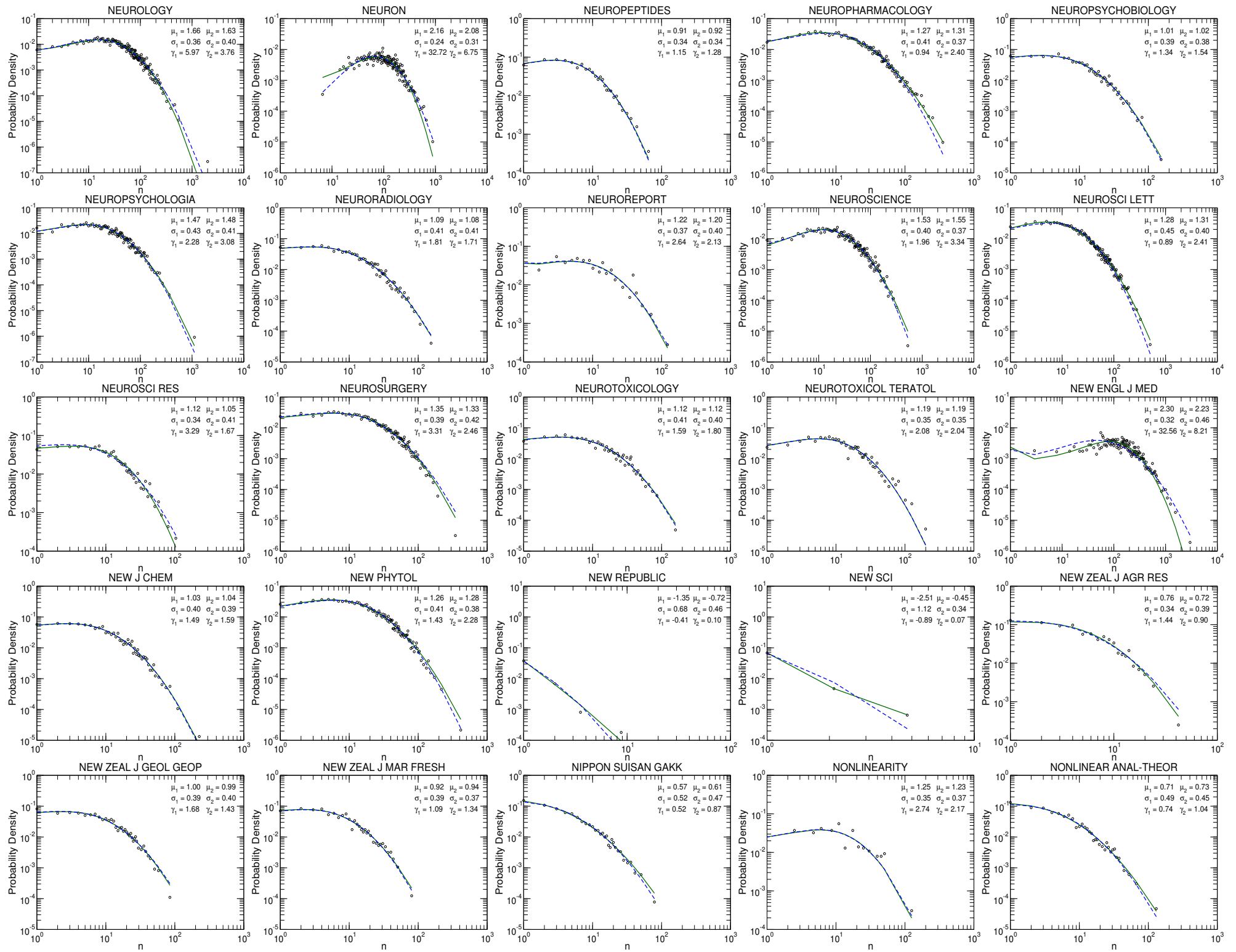

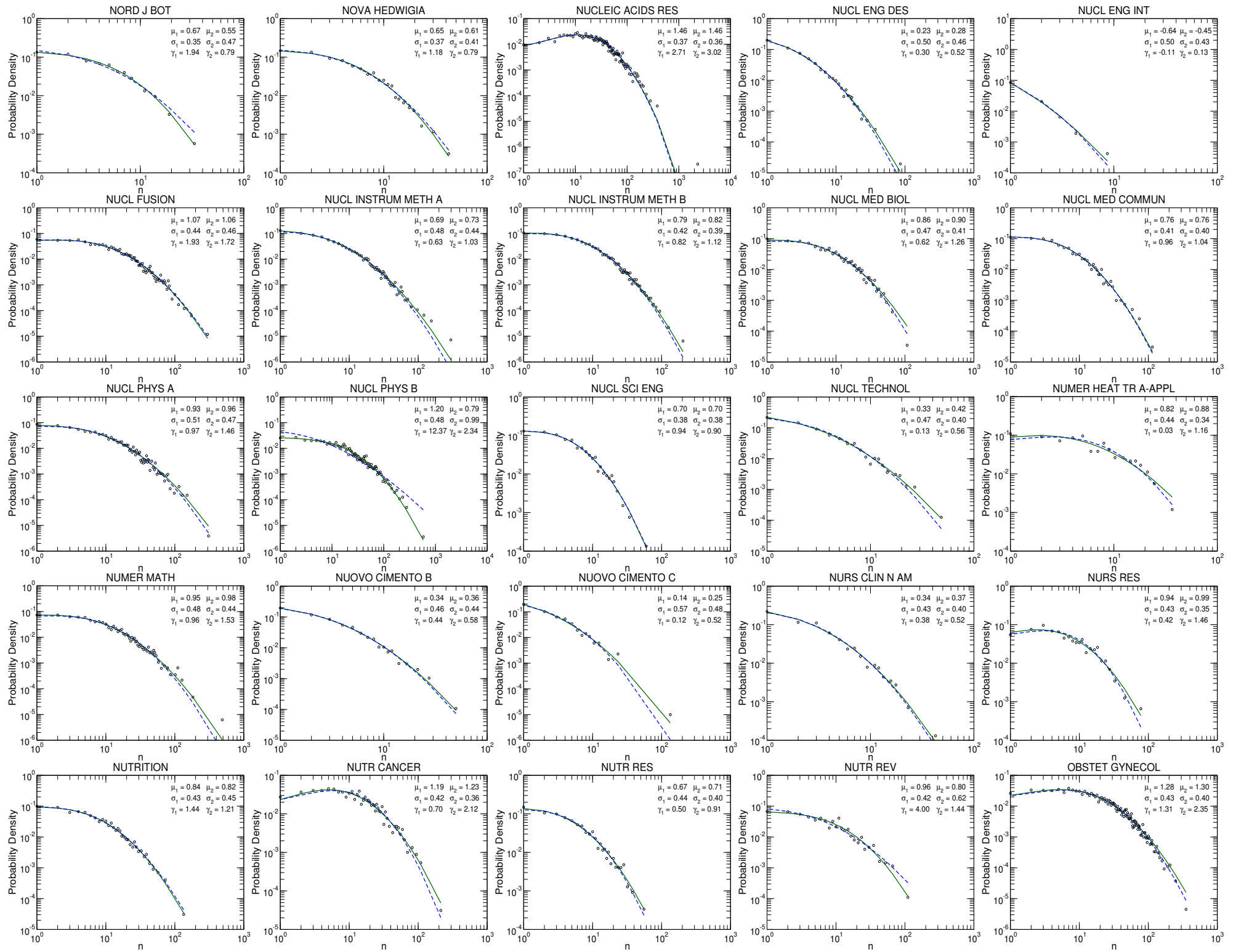

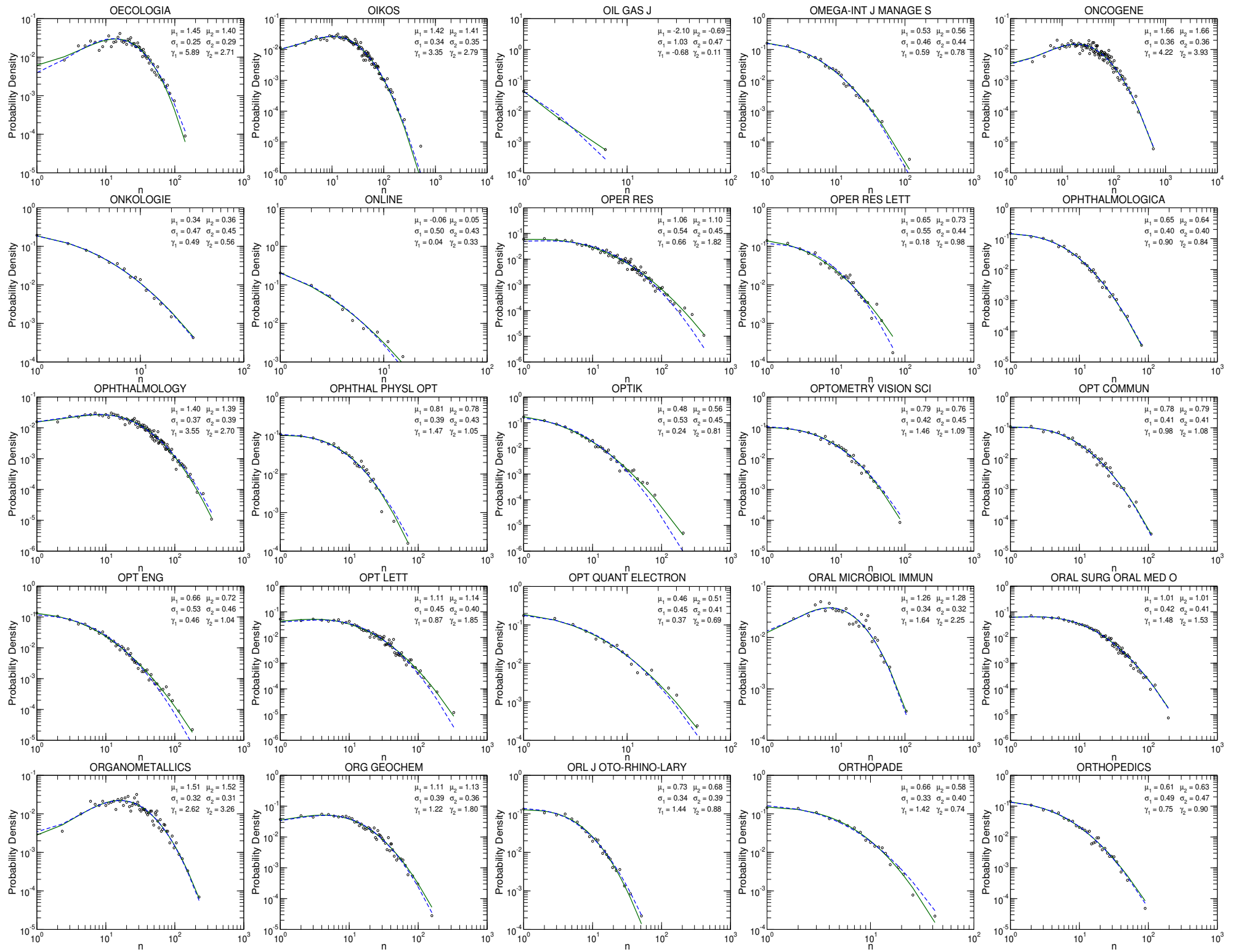

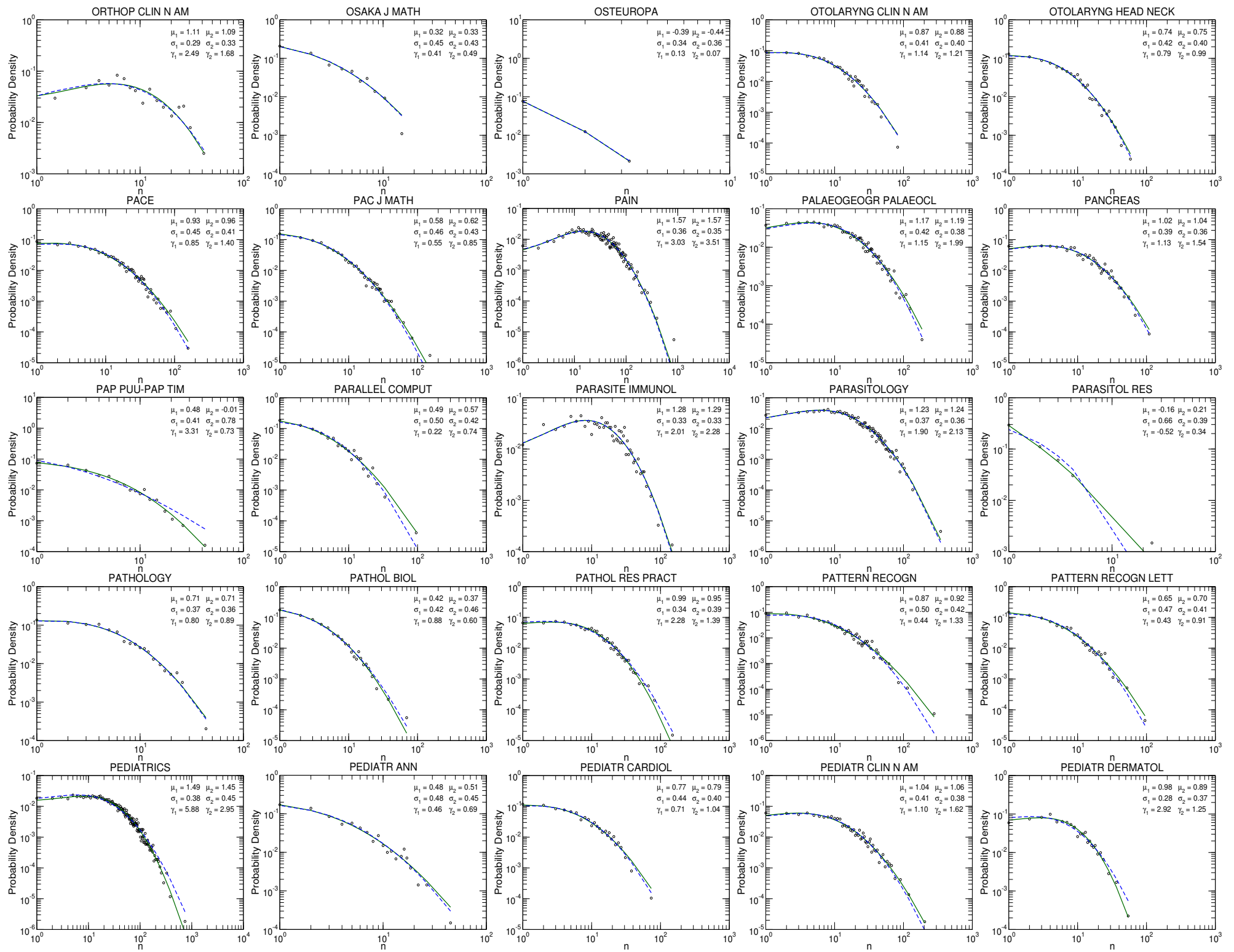

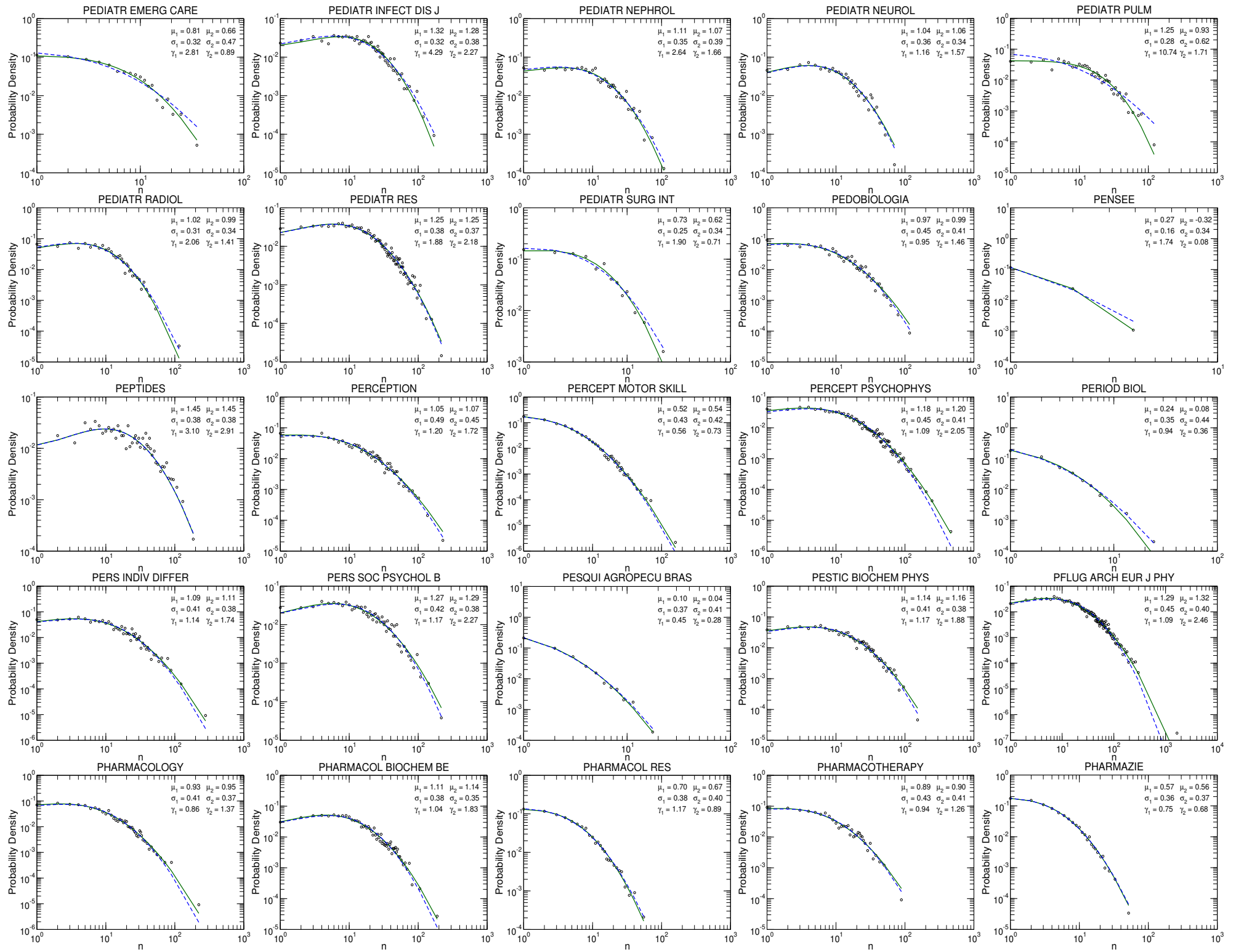

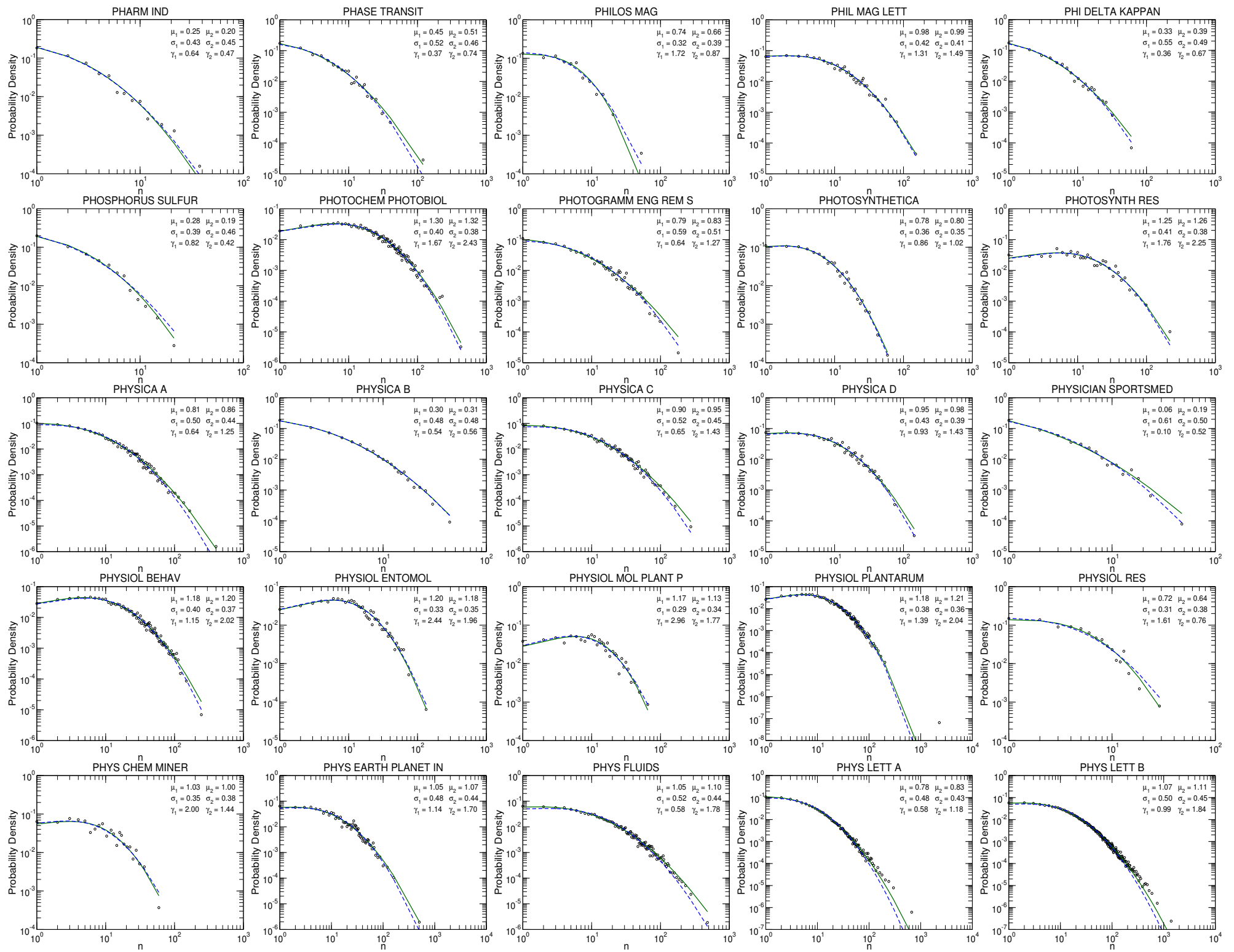

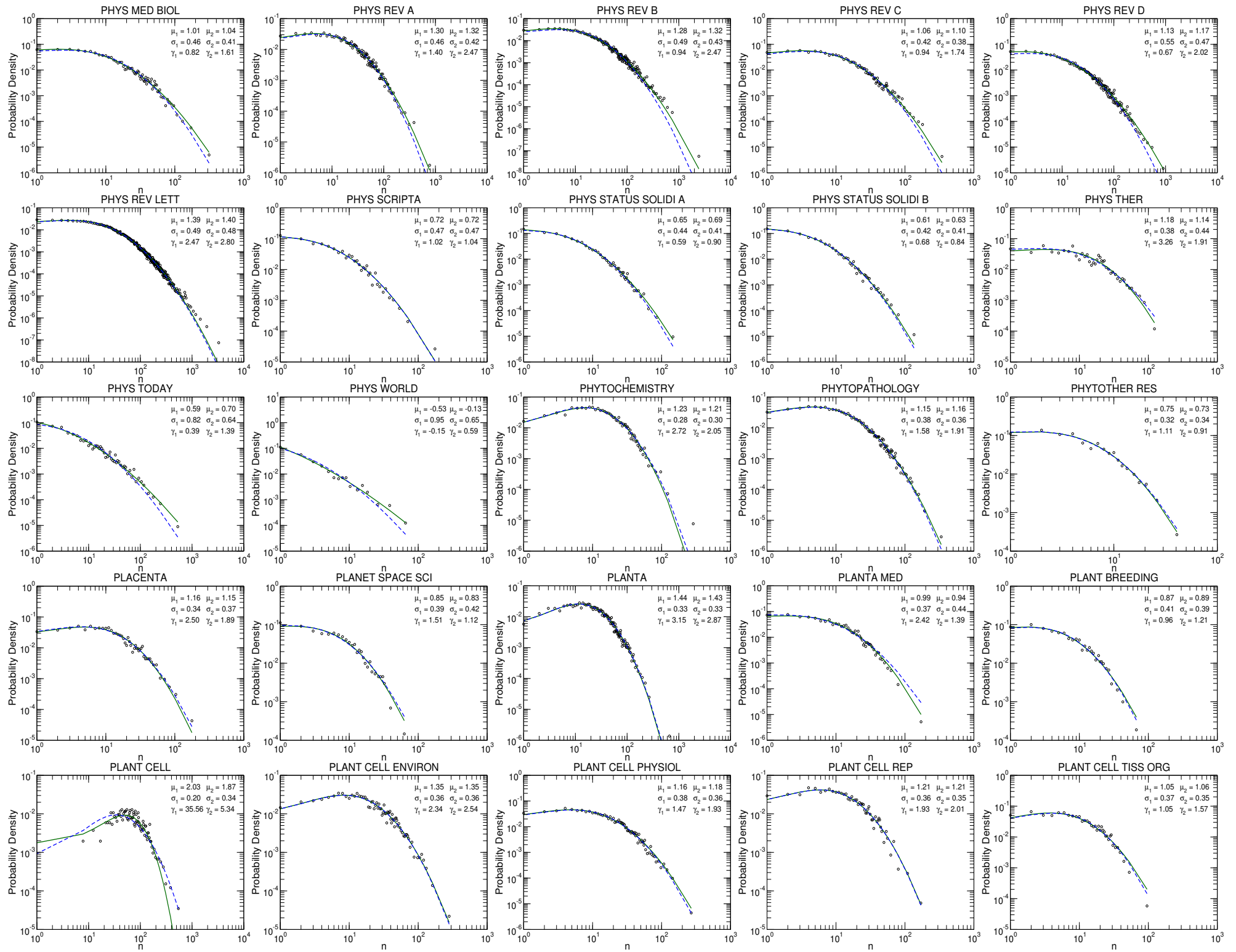

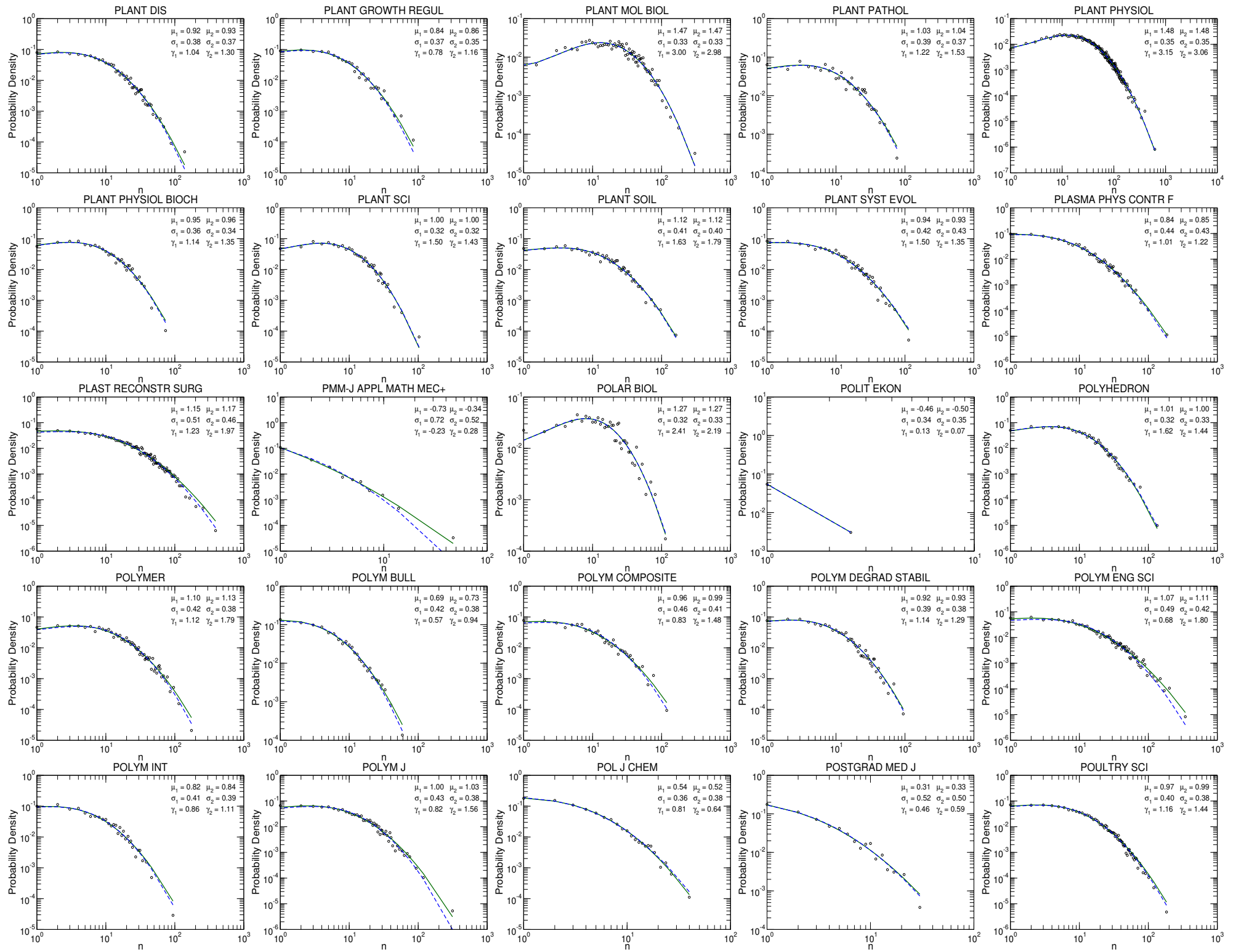

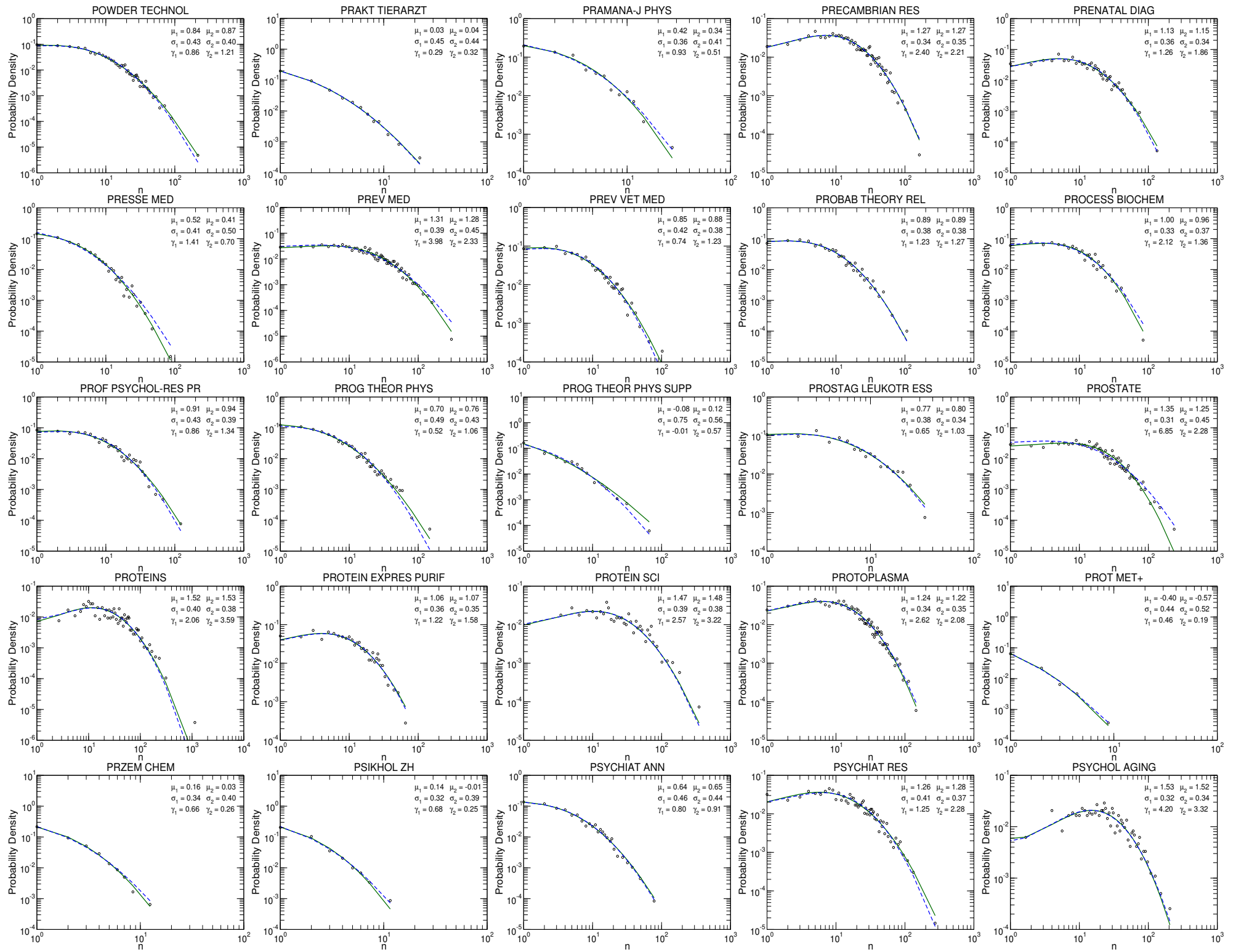

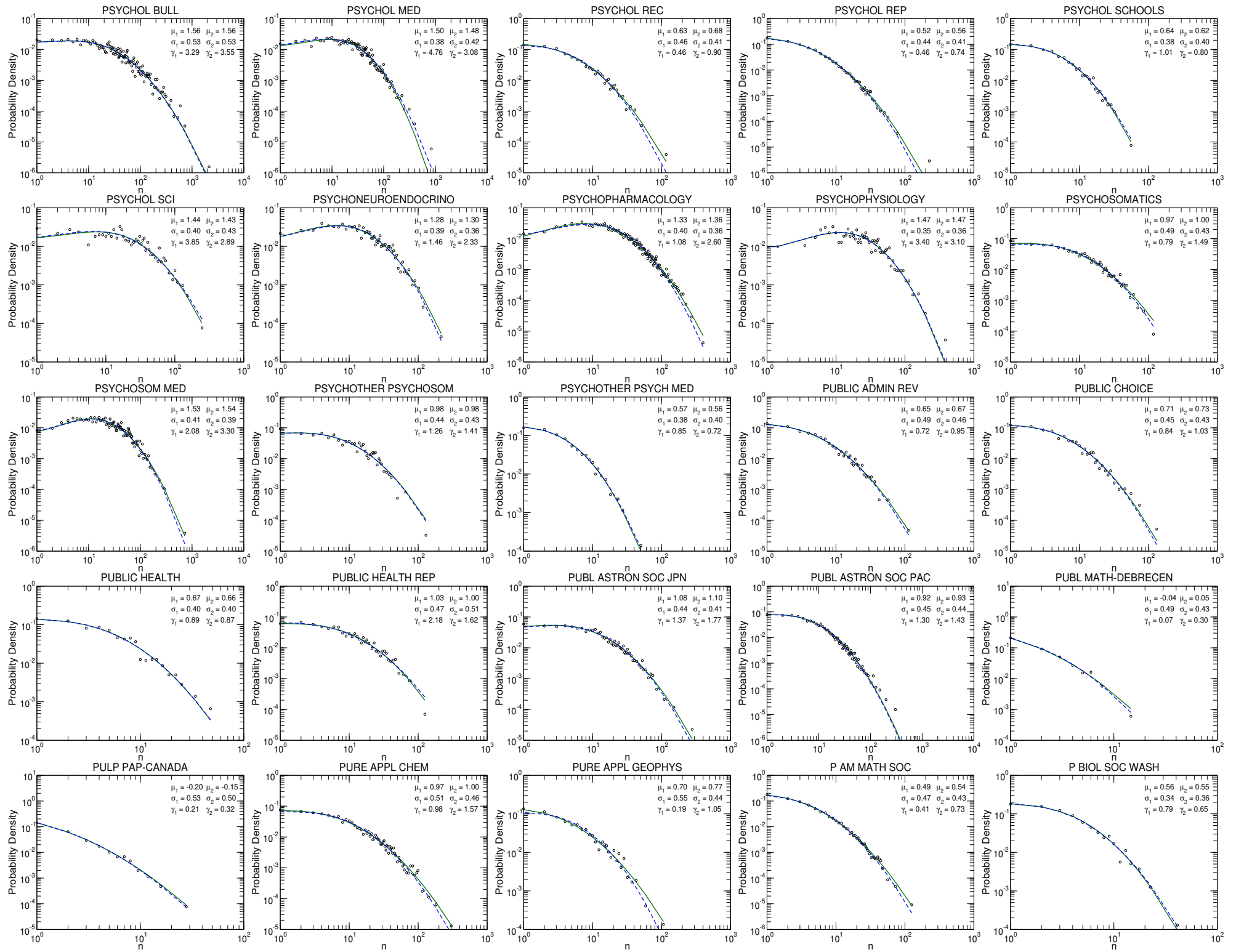

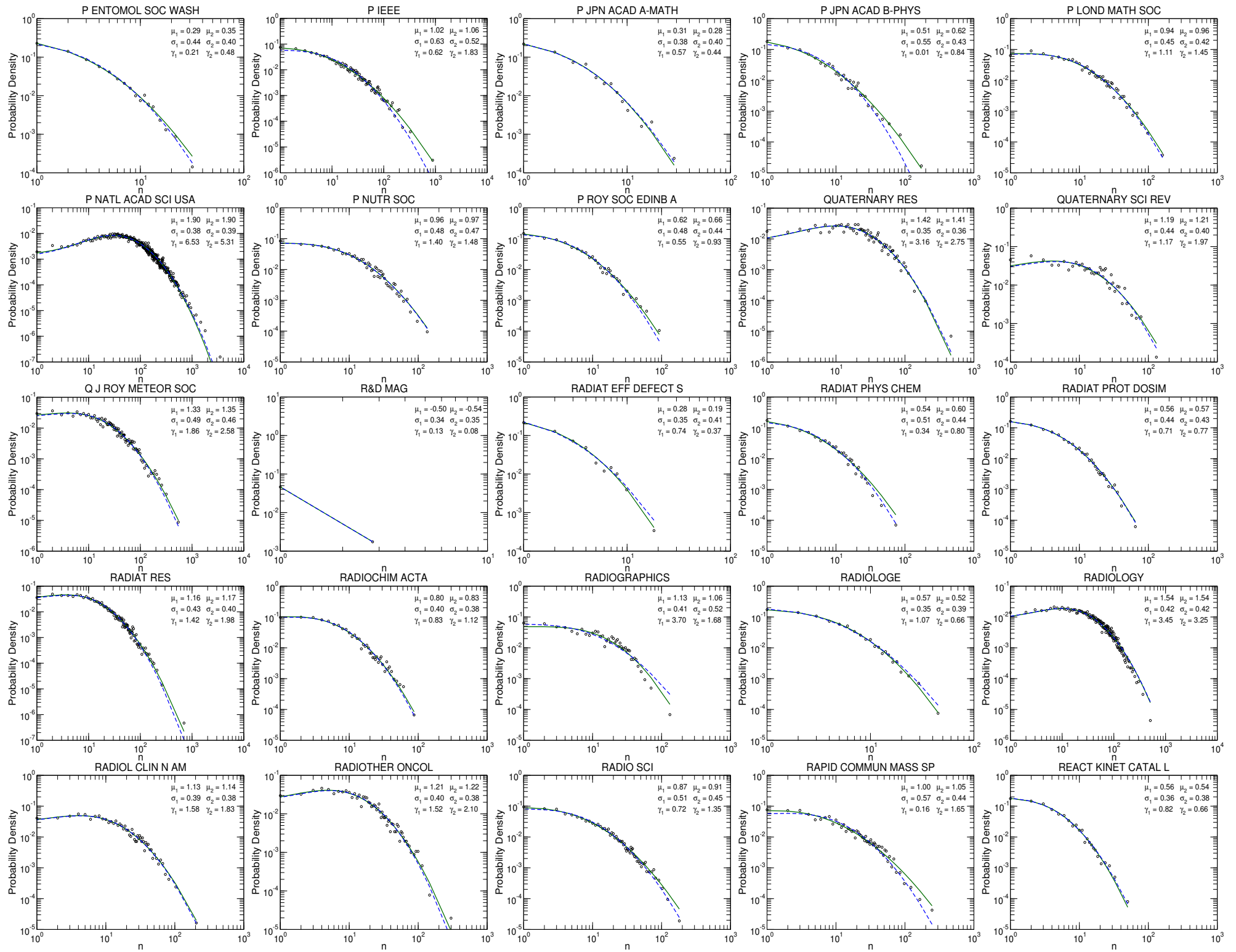

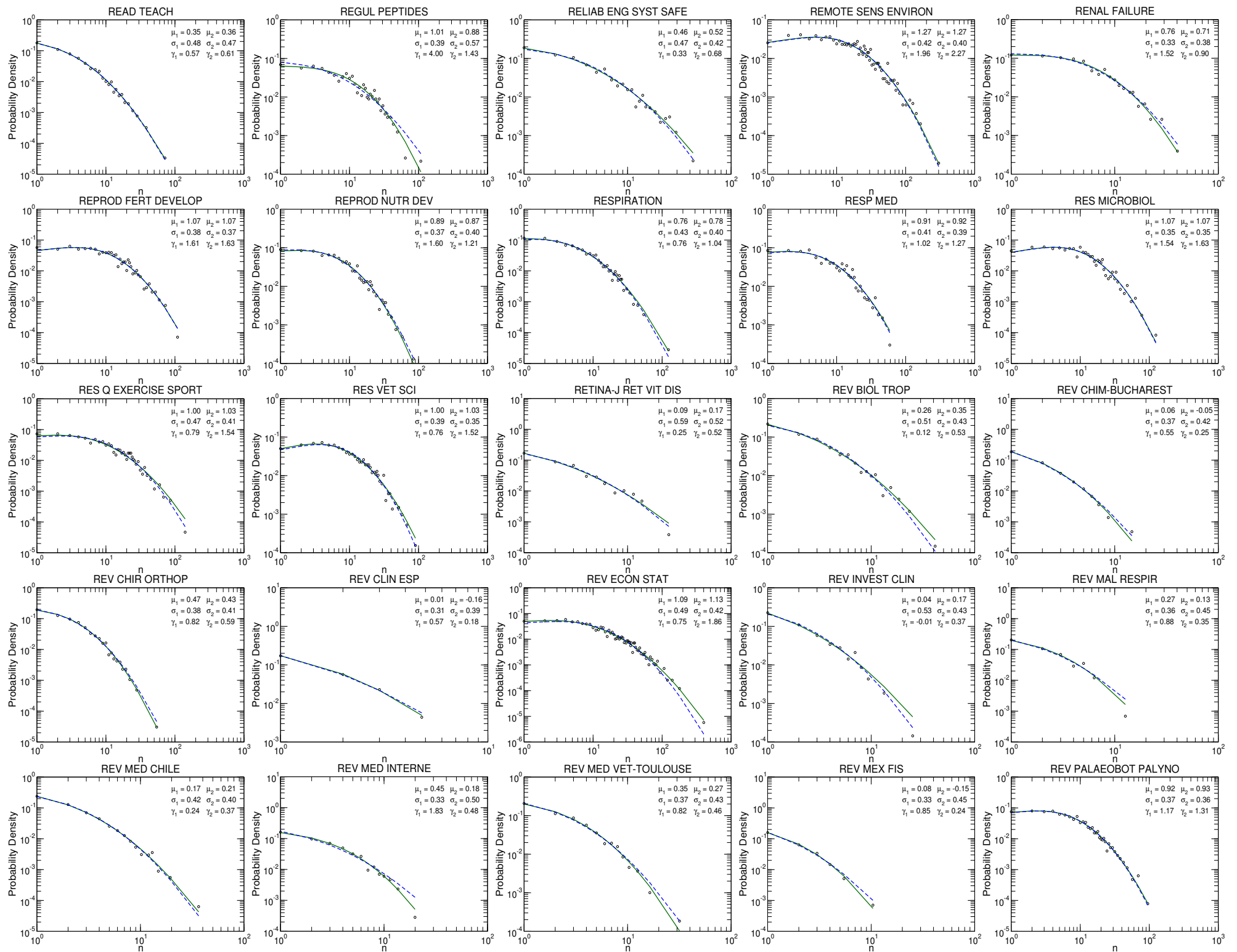

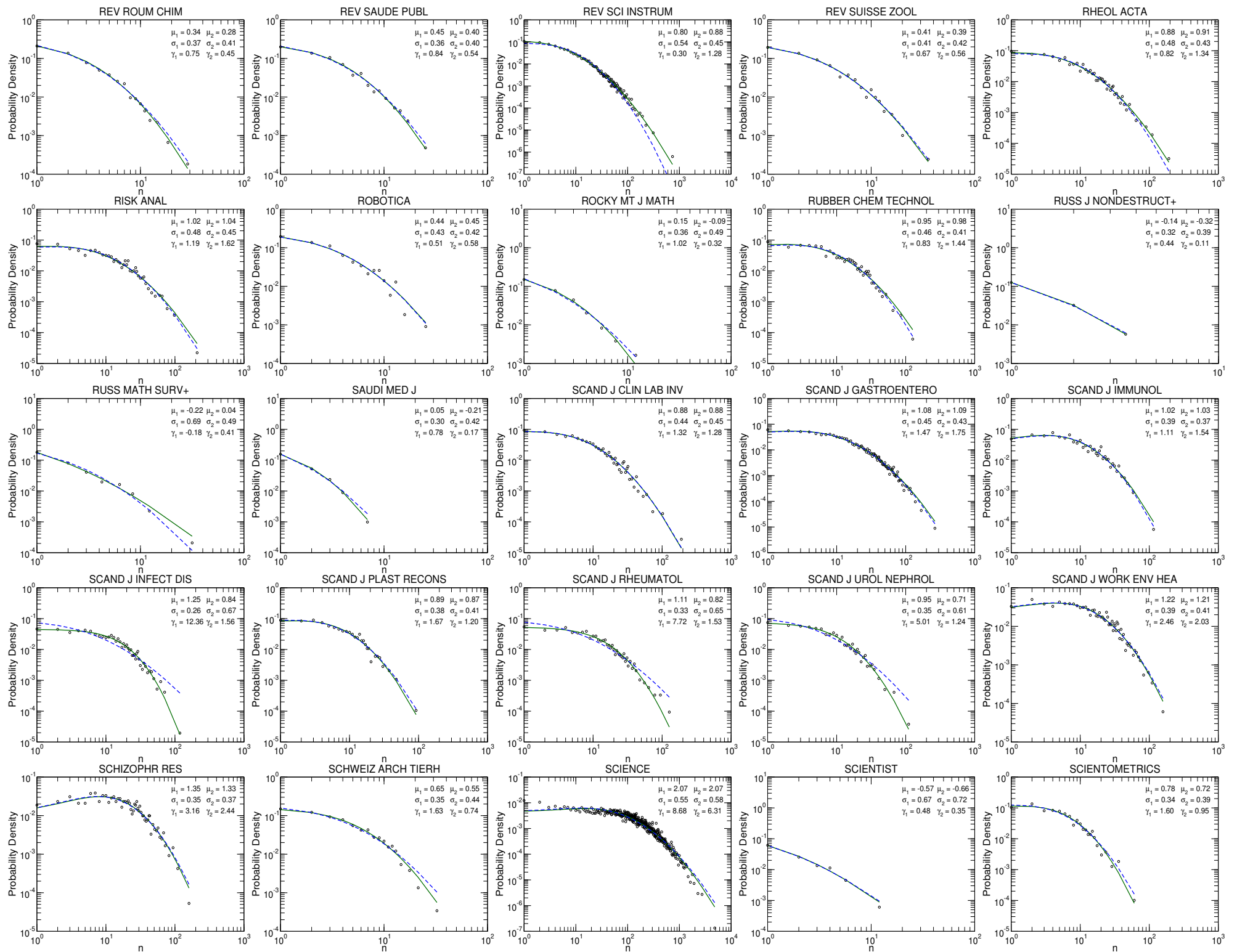

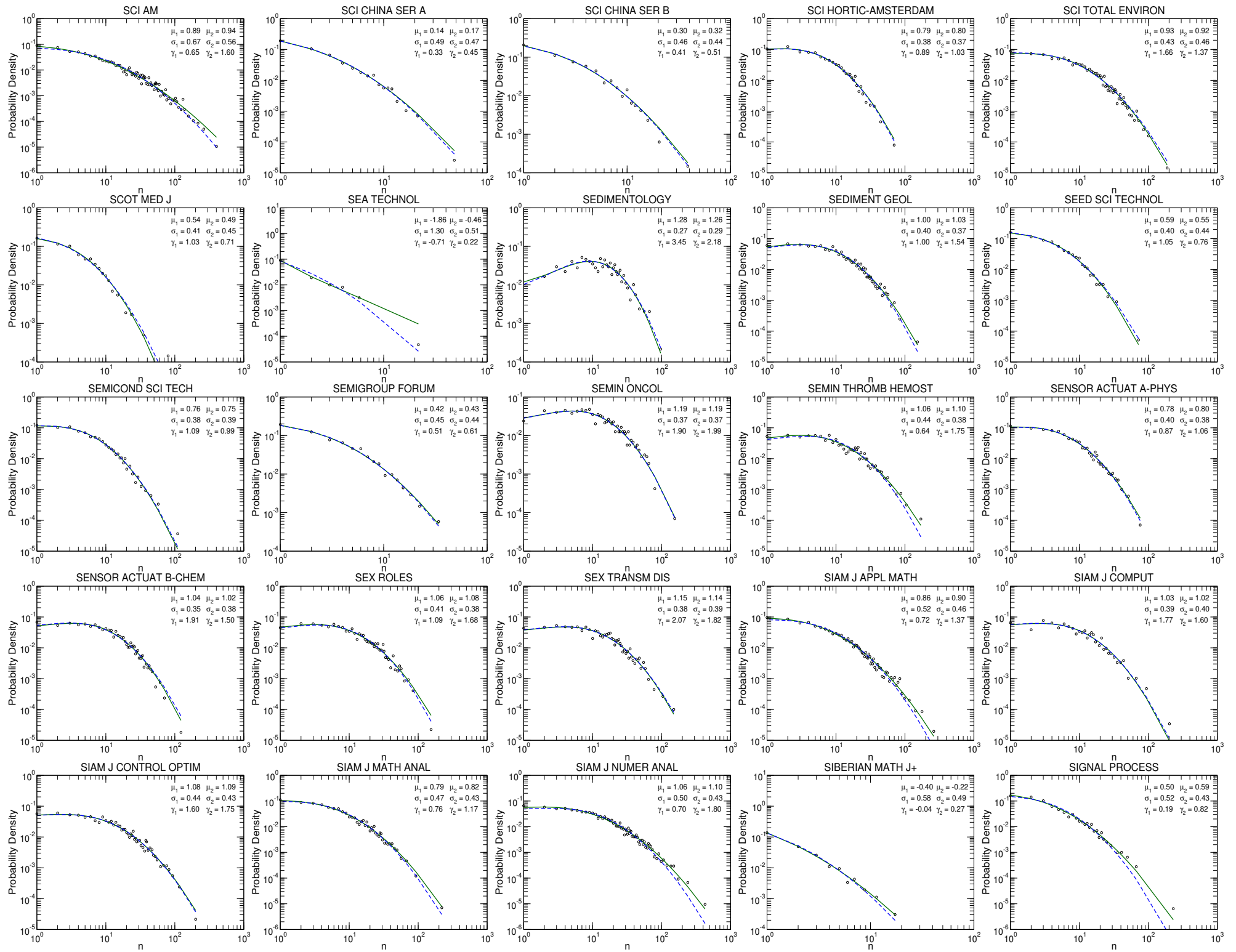

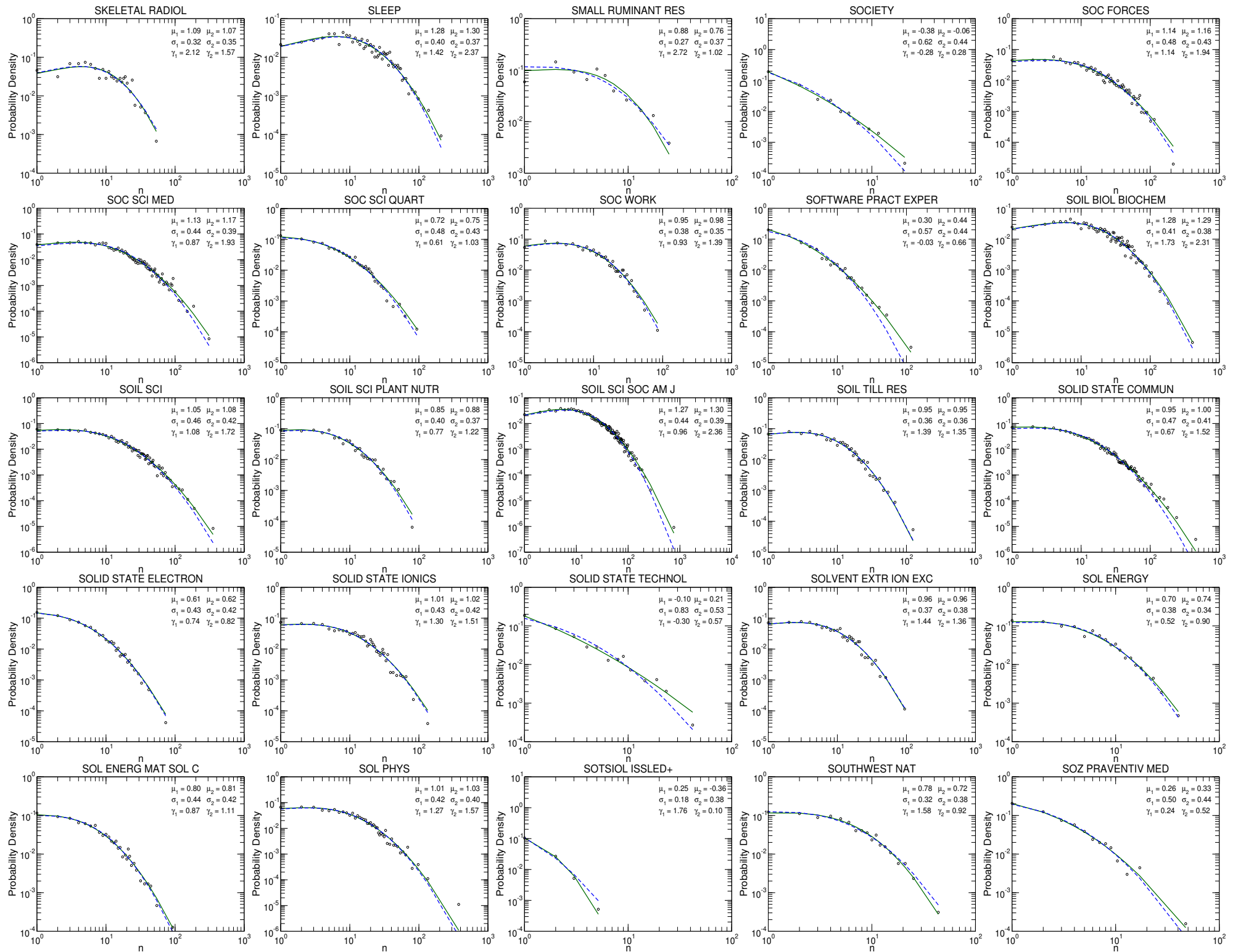

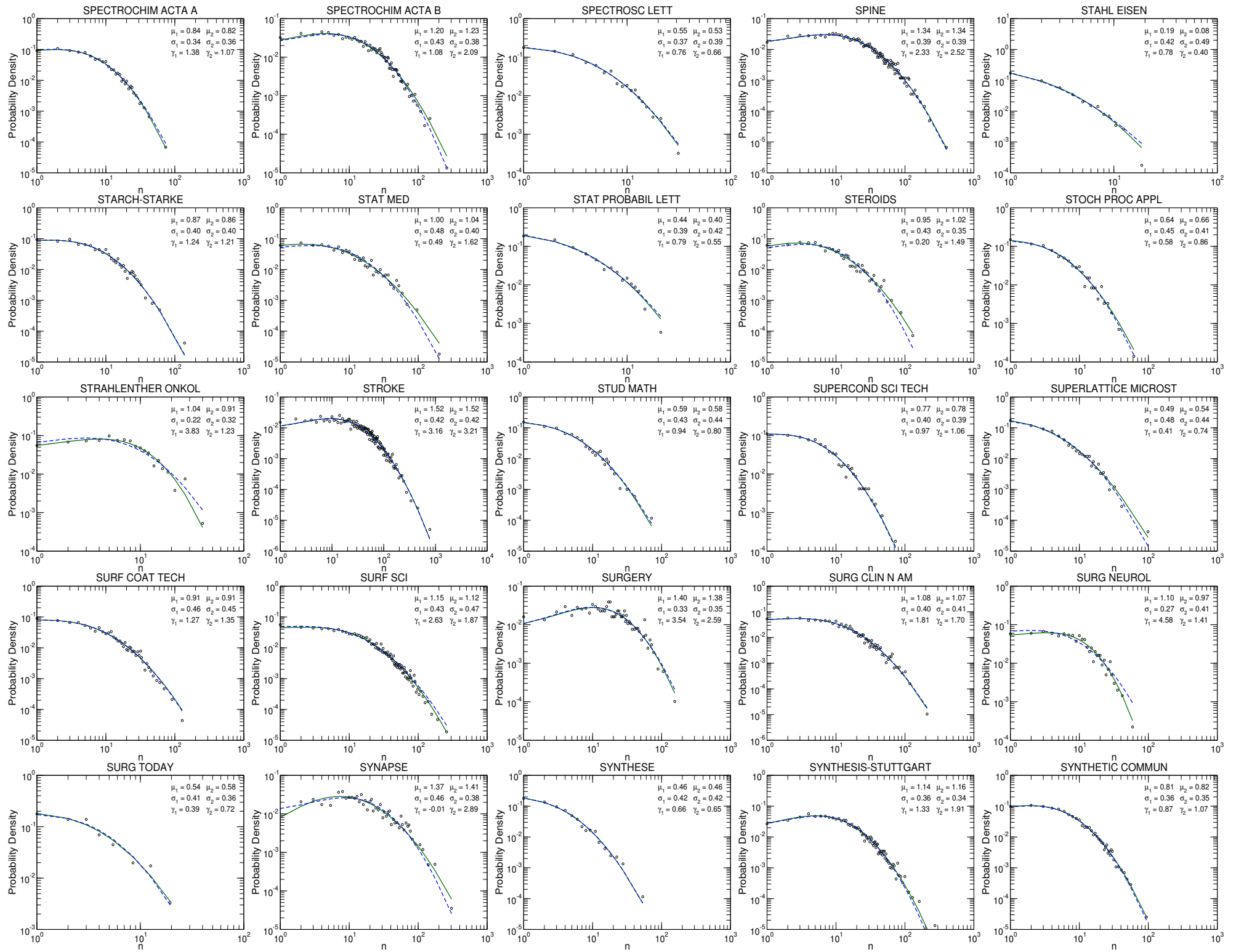

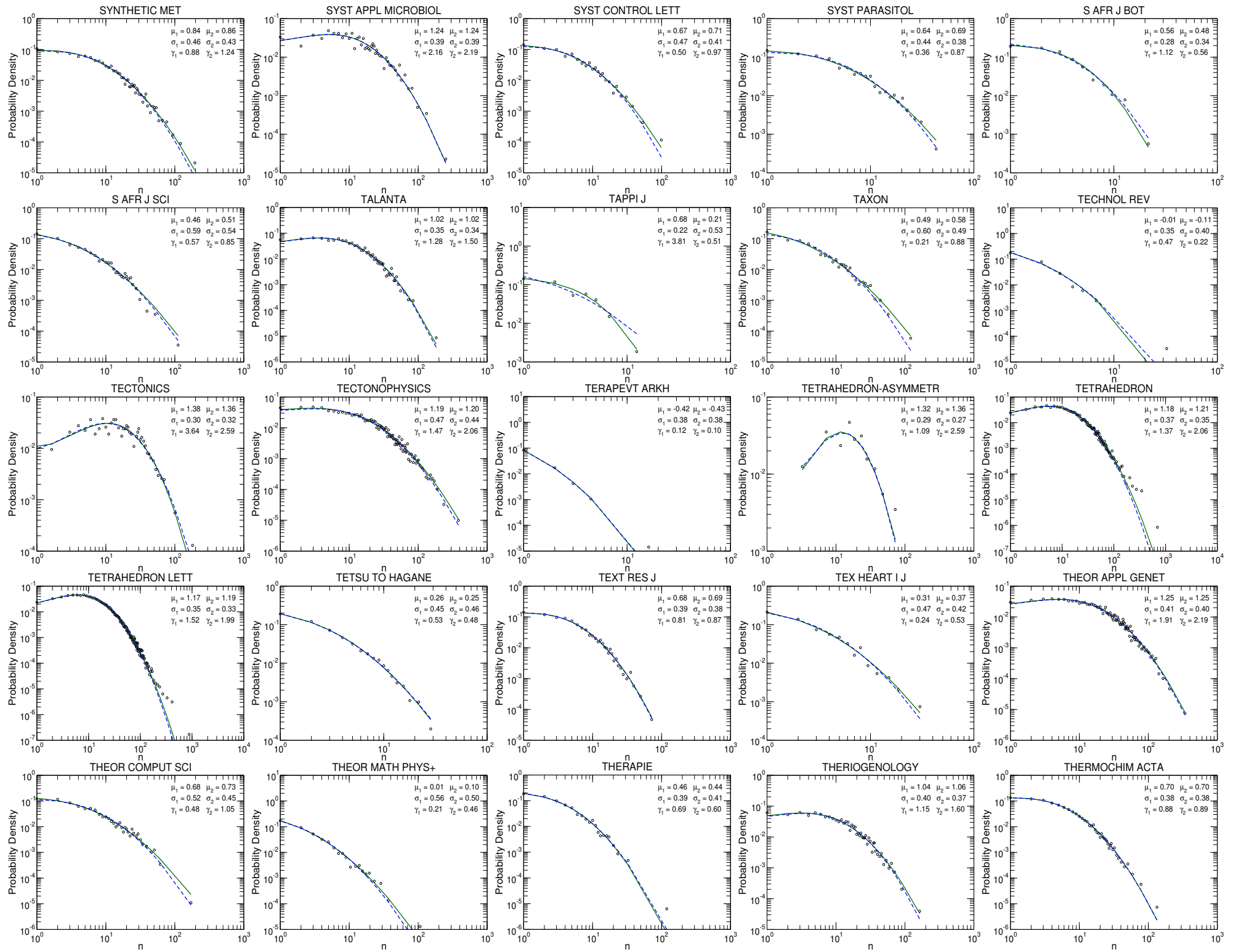

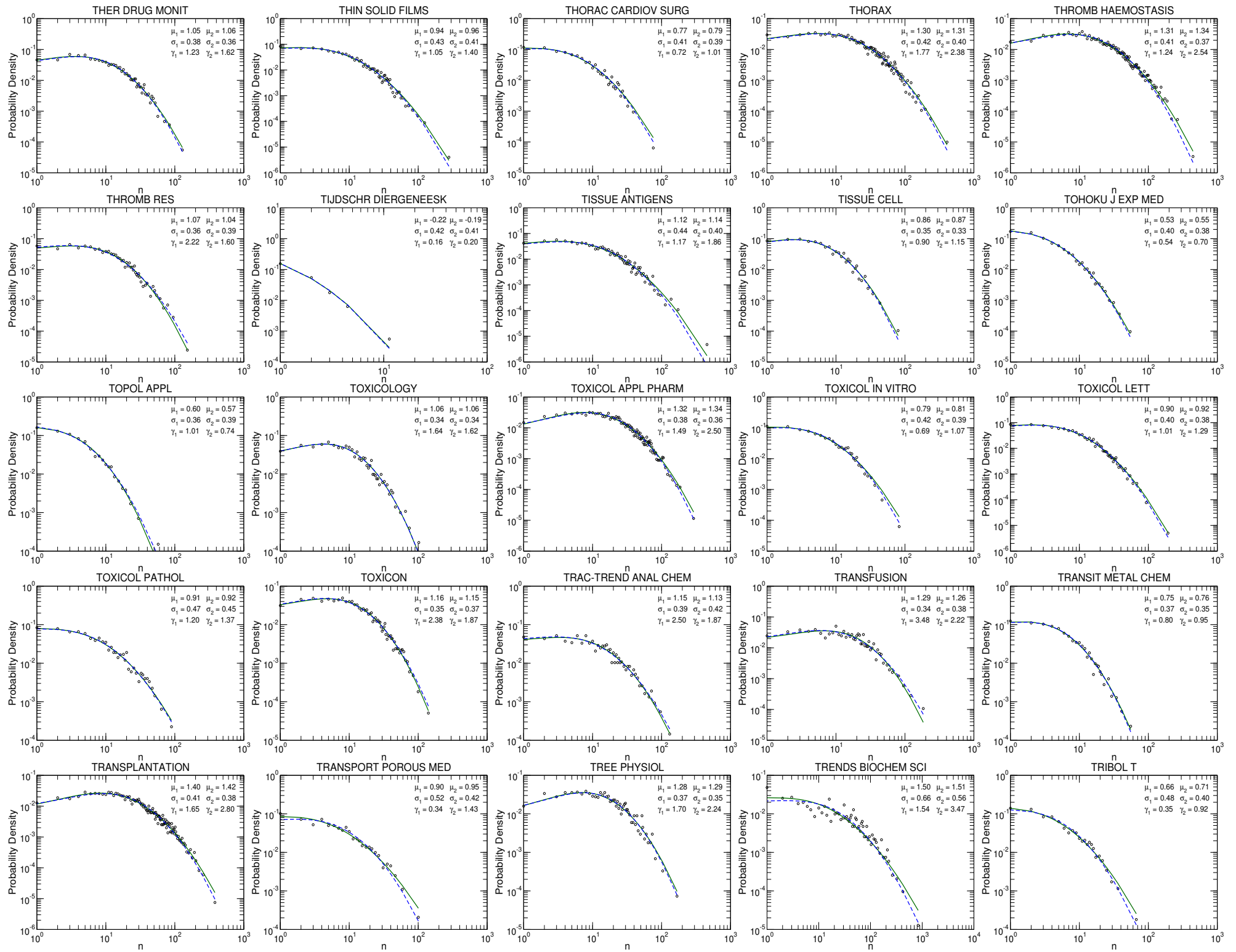

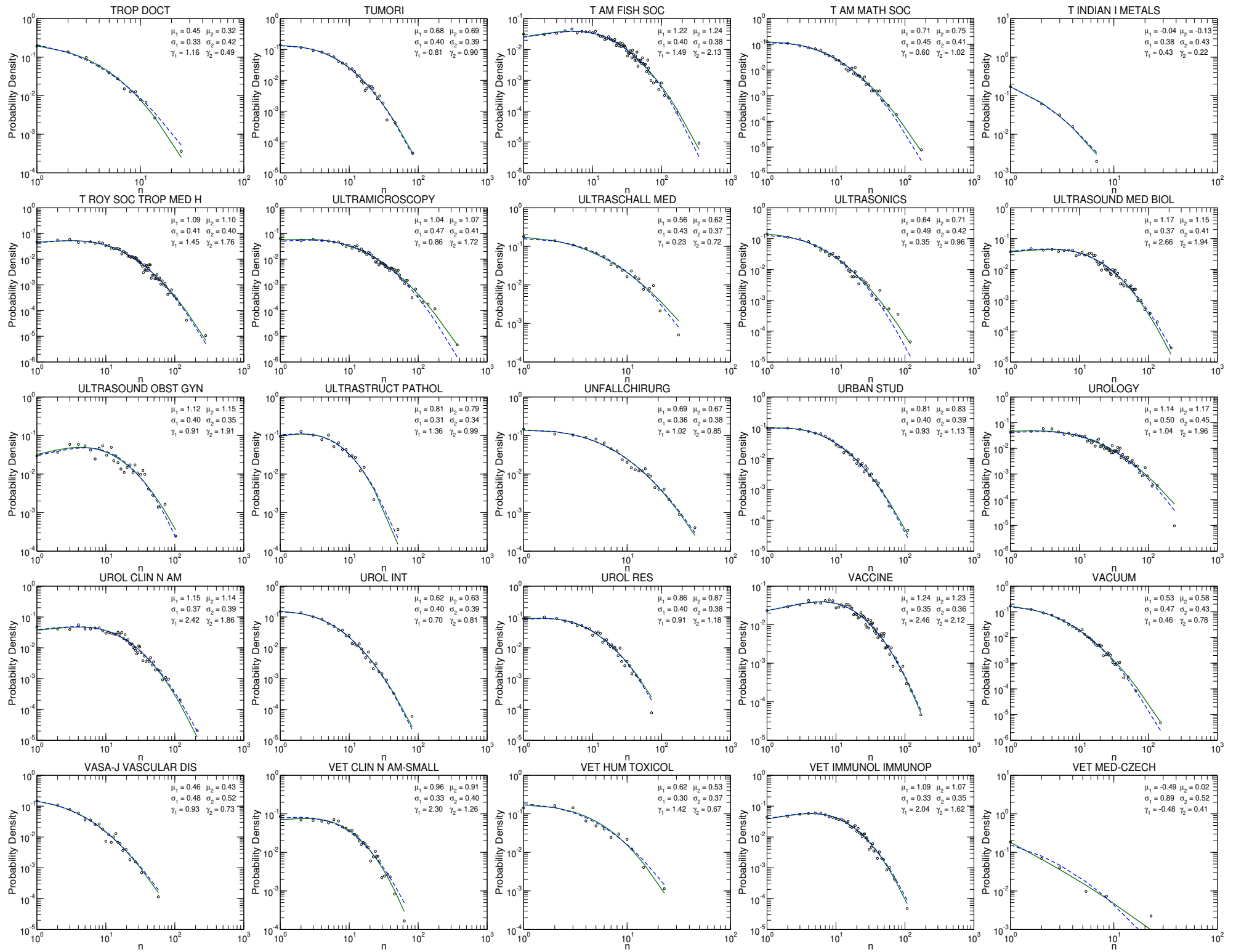

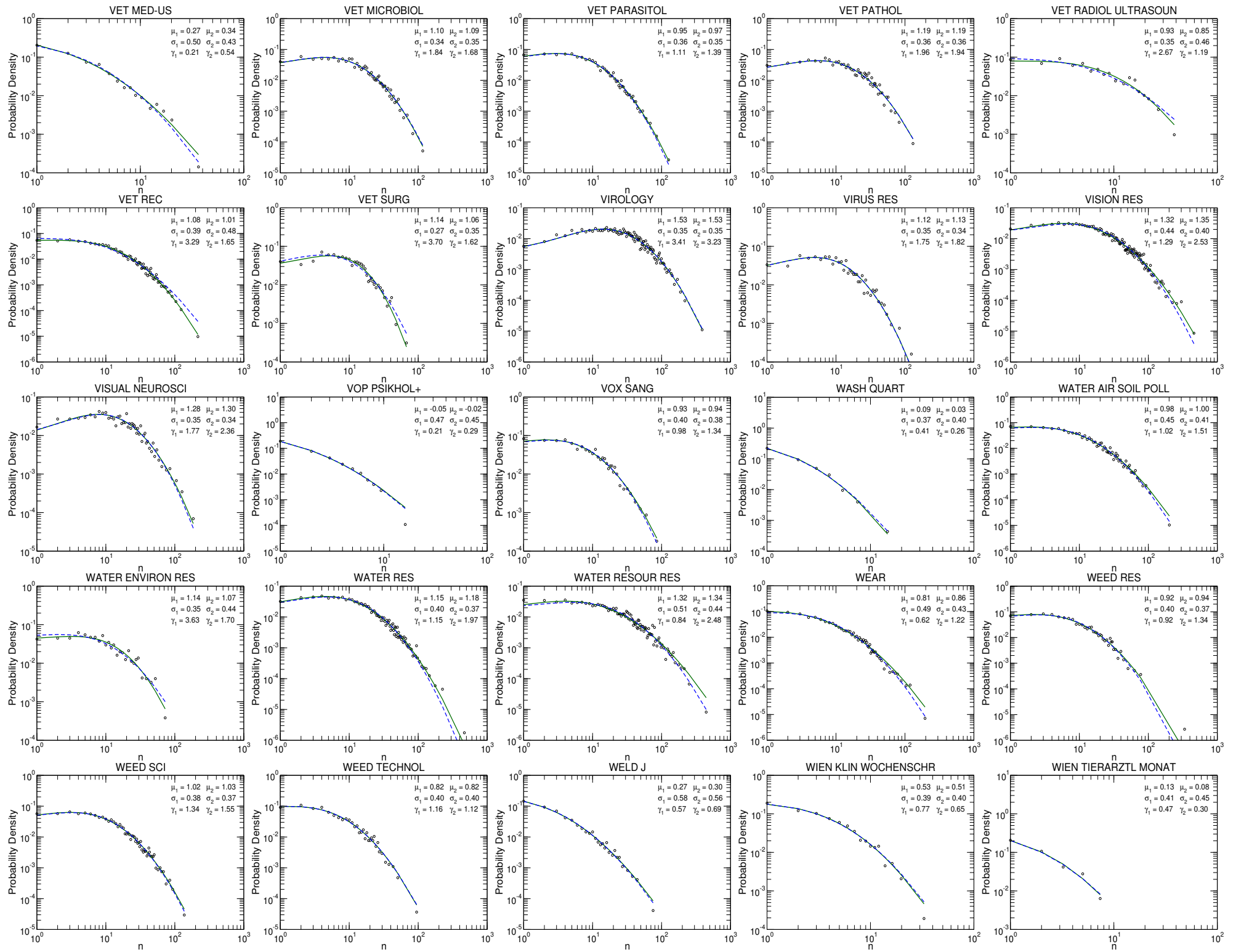

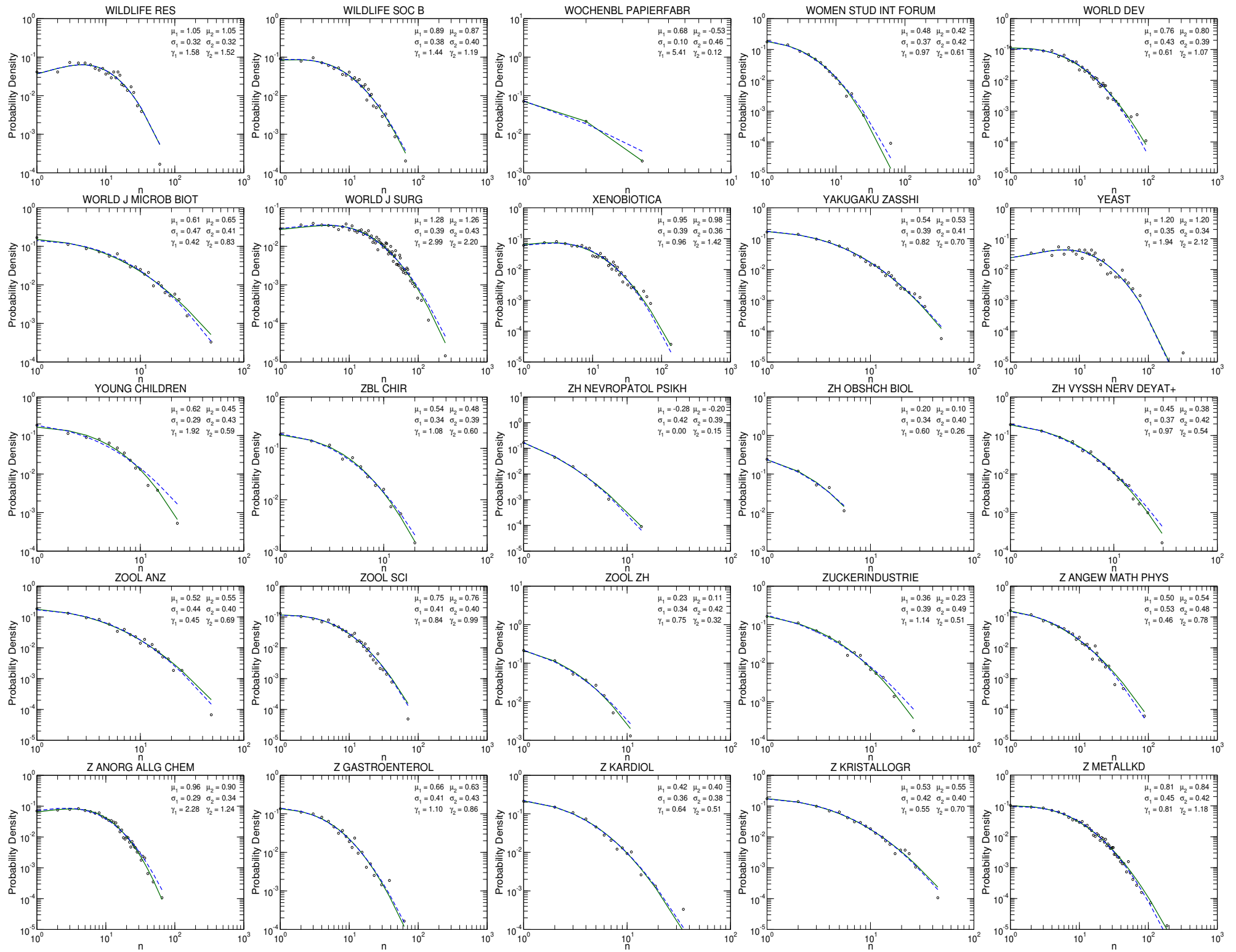

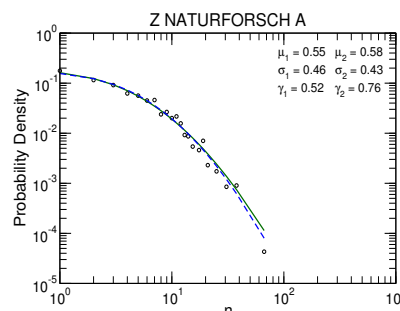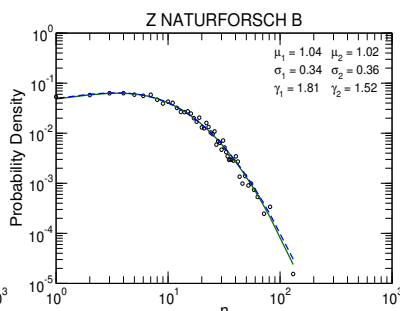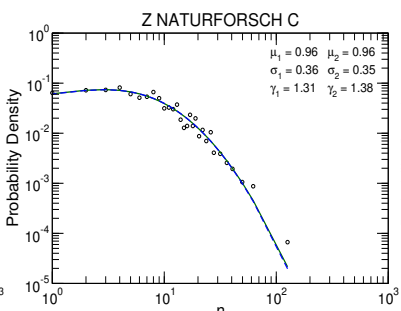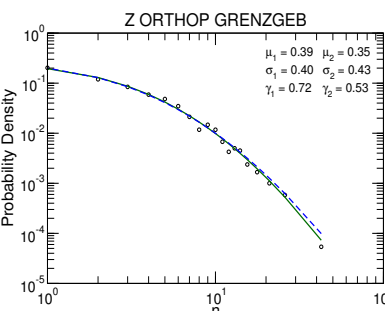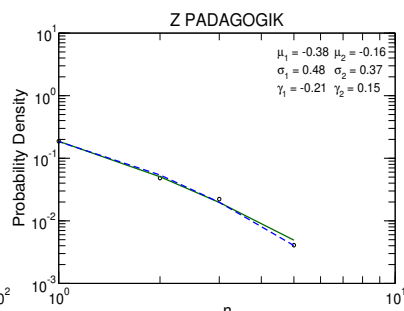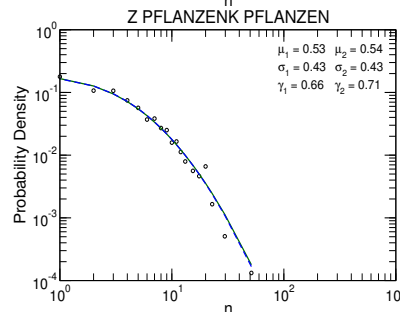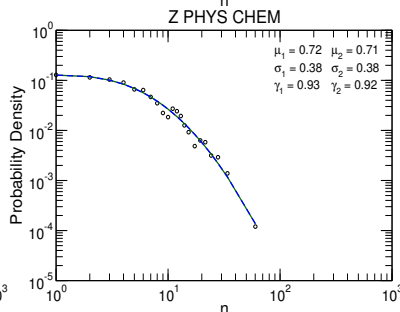

Supplement: Appendix S4 — Fit to the steady-state citation distribution for the 2,266 journals included in our analysis in alphabetical order. (21.06 MB PDF) [file pone.0001683.s004.pdf]
