# Supplementary material for: Effectiveness of Journal Ranking Schemes as a Tool for Locating Information
Source: PLoS One. 2008 Feb 27;3(2):e1683. doi: 10.1371/journal.pone.0001683 (PMC2244807; doi:10.1371/journal.pone.0001683)
Supplement: Appendix S3 — Comparison of ranking schemes for all the fields listed in the WoS. (12.95 MB PDF) [file pone.0001683.s003.pdf]

ACOUSTICS

ISI Category Description Acoustics covers resources on the study of the generation, control, transmission, reception, and effects of sounds. Relevant subjects include linear and nonlinear acoustics; atmospheric sound; underwater sound; the effects of mechanical vibrations; architectural acoustics; audio engineering; audiology; and ultrasound applications.

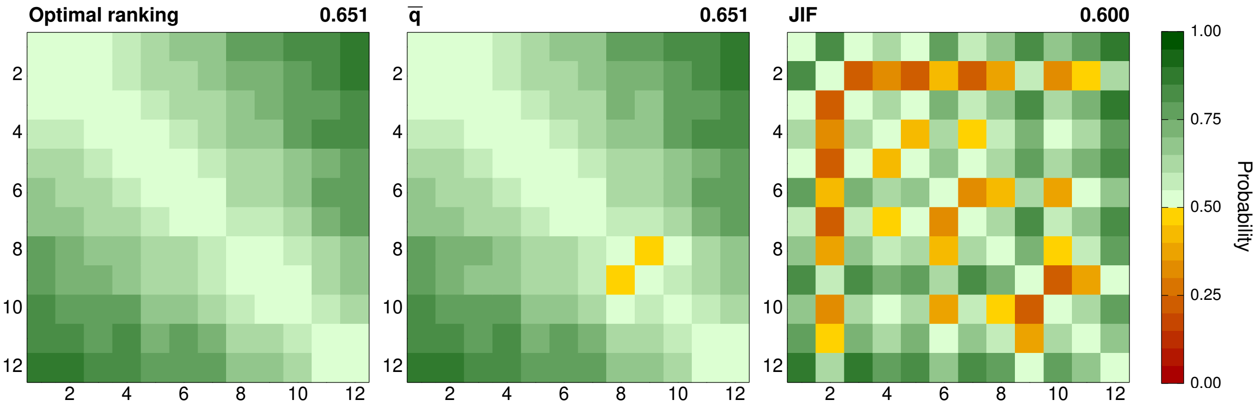

The color of each cell represents the probability of picking a paper with more citations from the higher-ranked journal. Green cells indicate adequate ranking, whereas red cells indicate that the ranking is inadequate (the probability is less than $\frac{1}{2}$). The value of M, the multi-class AUC statistic for each ranking scheme, is above each matrix.

Rank			$p_{ss}(q\|J)$		n			Steady-state
AUC	JIF	Journal abbreviation	$\bar{q}$	σ	$\bar{n}$	Q2	JIF	period
1	1	ULTRASOUND OBST GYN	1.15	0.35	18.5	12	2.288	1995–1997
2	3	ULTRASOUND MED BIOL	1.15	0.41	19.6	12	2.011	1979–1998
3	5	J ACOUST SOC AM	1.11	0.47	21.9	10	1.433	1954–1990
4	7	J ULTRAS MED	1.04	0.36	13.2	9	1.189	1981–1995
5	4	IEEE T ULTRASON FERR	0.97	0.42	13.4	7	1.729	1985–1998
6	10	J CLIN ULTRASOUND	0.92	0.36	10.5	6	0.573	1983–1996
7	8	J SOUND VIB	0.87	0.43	10.7	6	0.884	1966–1994
8	6	ULTRASONICS	0.71	0.42	7.6	4	1.322	1965–2001
9	11	J VIB ACOUST	0.73	0.49	7.0	4	0.565	1984–1997
10	2	ULTRASCHALL MED	0.62	0.37	4.5	3	2.103	1982–2003
11	9	J AUDIO ENG SOC	0.46	0.45	4.3	2	0.639	1964–2003
12	12	APPL ACOUST	0.37	0.45	2.9	1	0.484	1976–2002

AGRICULTURAL ENGINEERING

ISI Category Description Agricultural Engineering covers resources concerning many engineering applications in agriculture, including the design of machines, equipment, and buildings; soil and water engineering; irrigation and drainage engineering; crop harvesting, processing, and storage; animal production technology, housing, and equipment; precision agriculture; post-harvest processing and technology; rural development; agricultural mechanization; horticultural engineering; greenhouse structures and engineering, bioenergy and aquacultural engineering.

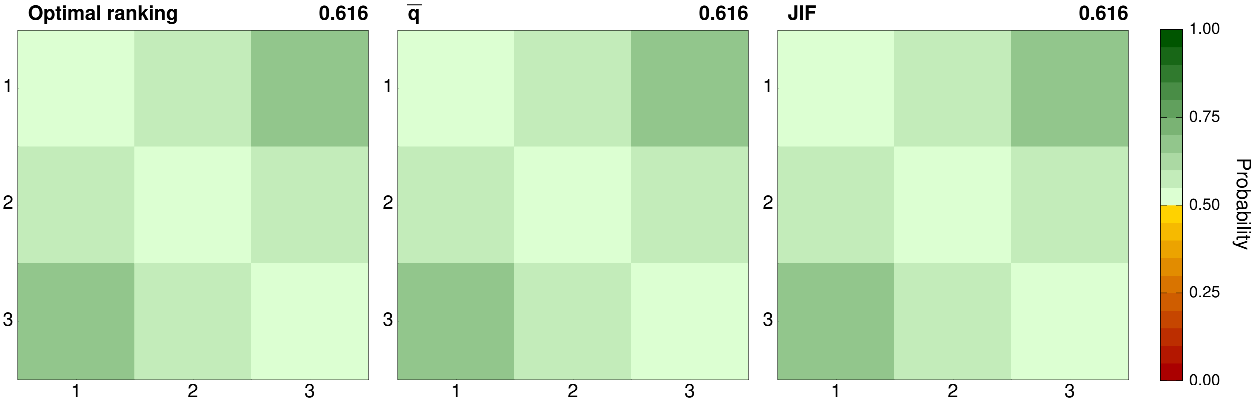

The color of each cell represents the probability of picking a paper with more citations from the higher-ranked journal. Green cells indicate adequate ranking, whereas red cells indicate that the ranking is inadequate (the probability is less than $\frac{1}{2}$). The value of M, the multi-class AUC statistic for each ranking scheme, is above each matrix.

Rank			$\mathbf{p_{ss}(q\|J)}$		n			Steady-state
**AUC**	**JIF**	**Journal abbreviation**	$\overline{\mathbf{q}}$	σ	$\overline{\mathbf{n}}$	**Q2**	**JIF**	**period**
1	1	BIORESOURCE TECHNOL	0.90	0.37	9.9	6	2.180	1991–2001
2	2	BIOMASS BIOENERG	0.80	0.39	7.0	5	1.483	1990–2002
3	3	J IRRIG DRAIN E-ASCE	0.63	0.39	5.0	3	1.250	1982–2002

AGRICULTURE, DAIRY & ANIMAL SCIENCE

ISI Category Description Agriculture, Dairy & Animal Science covers resources on the selection, breeding and management of livestock, including animal science, animal nutrition, poultry science, animal breeding and genetics, dairy science, and animal production science.

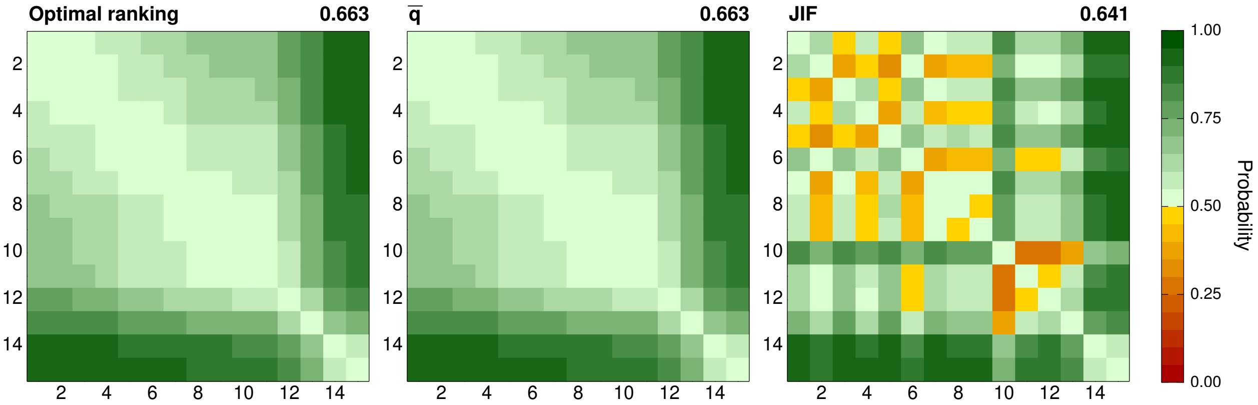

The color of each cell represents the probability of picking a paper with more citations from the higher-ranked journal. Green cells indicate adequate ranking, whereas red cells indicate that the ranking is inadequate (the probability is less than $\frac{1}{2}$). The value of M, the multi-class AUC statistic for each ranking scheme, is above each matrix.

Rank			$\mathbf{p_{ss}(q\|J)}$		n			Steady-state
**AUC**	**JIF**	**Journal abbreviation**	$\overline{\mathbf{q}}$	σ	$\overline{\mathbf{n}}$	**Q2**	**JIF**	**period**
1	5	J DAIRY RES	1.20	0.40	20.1	13	1.407	1954–1998
2	3	J ANIM SCI	1.17	0.38	19.5	12	1.983	1954–1996
3	1	J DAIRY SCI	1.14	0.40	18.8	11	2.284	1958–1998
4	7	APPL ANIM BEHAV SCI	1.11	0.38	15.3	11	1.177	1983–1994
5	9	LIVEST PROD SCI	1.05	0.39	13.9	9	1.131	1976–1998
6	8	BRIT POULTRY SCI	1.04	0.39	13.5	8	1.135	1968–1996
7	4	POULTRY SCI	0.99	0.38	12.0	8	1.656	1954–1999
8	2	ANIM REPROD SCI	0.95	0.37	11.2	7	2.186	1979–2001
9	12	CAN J ANIM SCI	0.93	0.37	10.3	7	0.767	1963–1994
10	11	J RANGE MANAGE	0.90	0.37	9.9	6	0.859	1966–1995
11	6	ANIM FEED SCI TECH	0.89	0.38	10.3	6	1.290	1976–1997
12	13	SMALL RUMINANT RES	0.76	0.37	6.2	5	0.637	1988–1991
13	10	J ANIM PHYSIOL AN N	0.56	0.39	3.7	3	1.075	1995–2001
14	14	ARCH TIERZUCHT	0.23	0.39	1.7	1	0.518	1975–2005
15	15	INDIAN J ANIM SCI	0.09	0.41	1.1	0	0.064	1973–1999

AGRICULTURE, MULTIDISCIPLINARY

ISI Category Description Agriculture, Multidisciplinary covers resources having a general or interdisciplinary approach to the agricultural sciences. Regional and multi-subject resources are also covered.

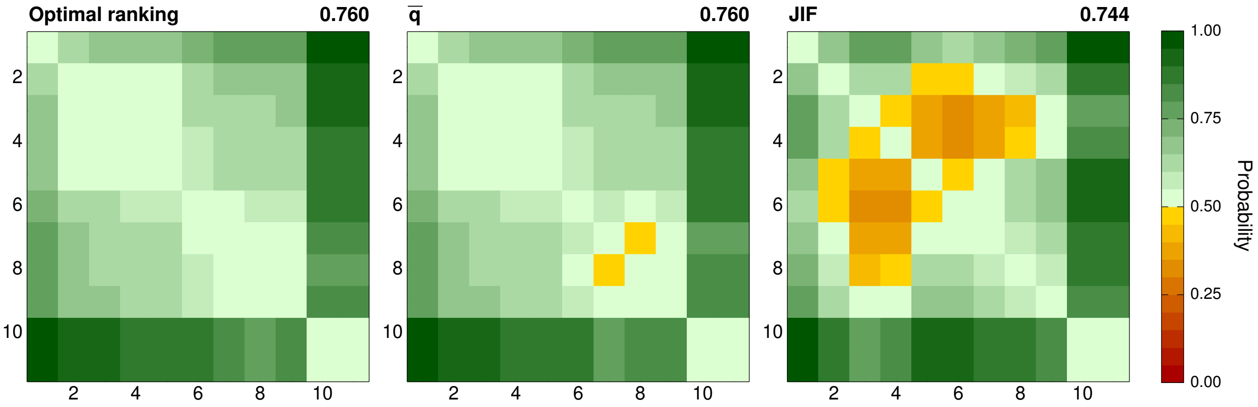

The color of each cell represents the probability of picking a paper with more citations from the higher-ranked journal. Green cells indicate adequate ranking, whereas red cells indicate that the ranking is inadequate (the probability is less than $\frac{1}{2}$). The value of M, the multi-class AUC statistic for each ranking scheme, is above each matrix.

**Rank**			$\mathbf{p_{ss}(q\|J)}$		**n**			**Steady-state**
**AUC**	**JIF**	**Journal abbreviation**	$\overline{\mathbf{q}}$	σ	$\overline{\mathbf{n}}$	**Q2**	**JIF**	**period**
1	1	J AGR FOOD CHEM	1.20	0.38	21.2	13	2.322	1966–1994
2	6	J SCI FOOD AGR	1.00	0.40	12.8	8	1.026	1986–1996
3	5	AUST J AGR RES	0.97	0.38	10.8	7	1.133	1987–1998
4	2	AGR ECOSYST ENVIRON	0.97	0.44	11.4	7	1.832	1982–2001
5	7	J AGR SCI	0.93	0.41	12.7	6	0.861	1979–1996
6	8	AUST J EXP AGR	0.80	0.35	7.2	5	0.861	1975–1996
7	4	AGR SYST	0.73	0.40	6.9	4	1.378	1978–2002
8	3	ANN APPL BIOL	0.74	0.49	7.7	4	1.379	1984–1998
9	9	NEW ZEAL J AGR RES	0.72	0.39	5.7	4	0.530	1990–2001
10	10	PESQUI AGROPECU BRAS	0.04	0.41	1.0	0	0.286	1980–2003
11	11	INDIAN J AGR SCI	0.02	0.39	0.9	0	0.106	1973–1999

AGRONOMY

ISI Category Description Agronomy covers resources on the selection, breeding, management, and post-harvest treatment of crops including crop protection and science, seed science, plant nutrition, plant and soil science, soil management and tillage, weed science, agroforestry, agroclimatology, and agricultural water management.

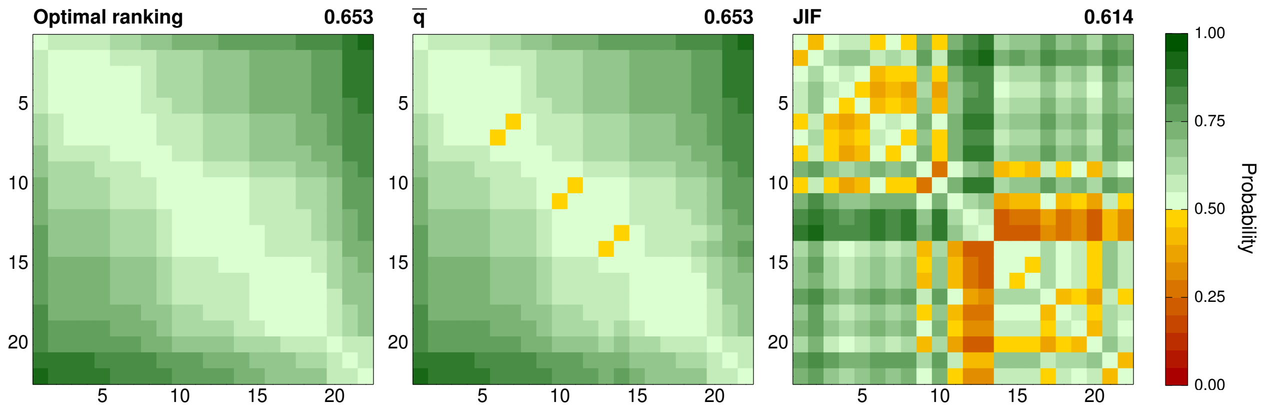

The color of each cell represents the probability of picking a paper with more citations from the higher-ranked journal. Green cells indicate adequate ranking, whereas red cells indicate that the ranking is inadequate (the probability is less than $\frac{1}{2}$). The value of M, the multi-class AUC statistic for each ranking scheme, is above each matrix.

Rank			$p_{ss}(q\|J)$		n			Steady-state
AUC	JIF	Journal abbreviation	$\overline{q}$	σ	$\overline{n}$	Q2	JIF	period
1	2	THEOR APPL GENET	1.25	0.40	23.9	15	2.715	1985–1999
2	6	PLANT SOIL	1.12	0.40	17.1	11	1.495	1988–1991
3	10	CROP SCI	1.10	0.38	16.6	10	1.153	1967–1995
4	8	AGRON J	1.10	0.38	16.5	10	1.272	1966–1994
5	1	AGR FOREST METEOROL	1.09	0.39	17.3	10	2.903	1983–2002
6	7	WEED SCI	1.03	0.37	13.3	9	1.476	1967–1999
7	3	PLANT PATHOL	1.04	0.37	12.8	9	2.198	1981–1993
8	5	FIELD CROP RES	1.00	0.38	12.1	8	1.634	1977–2000
9	4	WEED RES	0.94	0.37	12.4	7	1.705	1965–1998
10	20	SOIL SCI PLANT NUTR	0.88	0.37	9.7	6	0.443	1978–1994
11	14	PLANT BREEDING	0.89	0.39	9.2	6	0.954	1985–1996
12	16	EUPHYTICA	0.84	0.38	8.0	5	0.907	1984–1998
13	15	AGROFOREST SYST	0.81	0.30	7.1	5	0.921	1999–2001
14	18	WEED TECHNOL	0.82	0.40	8.2	5	0.626	1986–2000
15	9	CROP PROT	0.75	0.37	6.6	4	1.199	1982–2001
16	19	CAN J PLANT SCI	0.75	0.41	6.3	4	0.484	1988–1997
17	22	COMMUN SOIL SCI PLAN	0.72	0.41	8.9	4	0.302	1973–1995
18	11	AGR WATER MANAGE	0.71	0.40	6.0	4	1.122	1977–2002
19	17	AGRONOMIE	0.65	0.39	5.3	3	0.863	1980–2002
20	21	SEED SCI TECHNOL	0.55	0.44	4.6	2	0.410	1976–1998
21	12	J AGRON CROP SCI	0.44	0.36	2.8	2	1.046	1985–2003
22	13	CEREAL RES COMMUN	0.36	0.41	2.7	1	1.037	1984–2001

ALLERGY

ISI Category Description Allergy covers resources dealing with the full spectrum of immunologically-mediated hypersensitivity reactions including immediate or acute hypersensitivity, dermatitis, and asthma. This category also covers resources on the underlying cellular and molecular immunology specific to allergic reactivity, pathogenesis, tissue damage, clinical presentation, and modes of treatment.

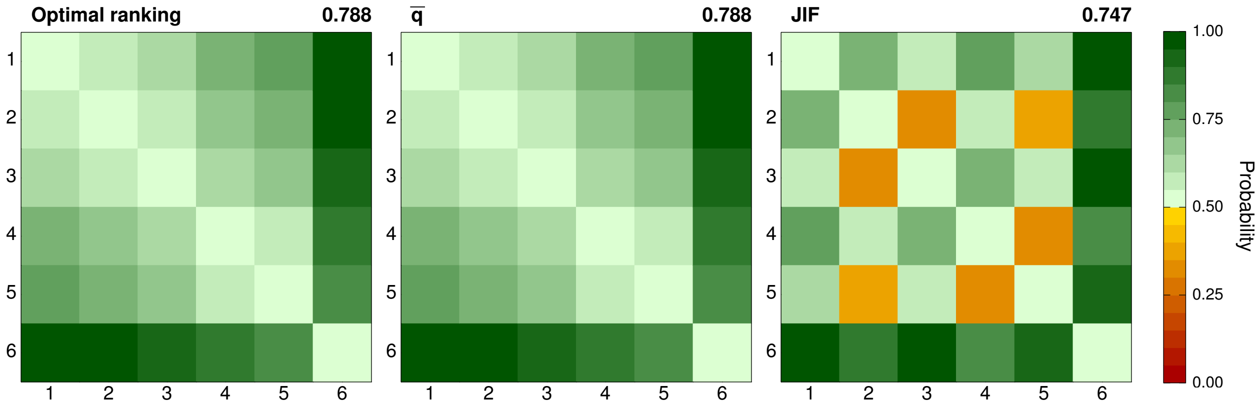

The color of each cell represents the probability of picking a paper with more citations from the higher-ranked journal. Green cells indicate adequate ranking, whereas red cells indicate that the ranking is inadequate (the probability is less than $\frac{1}{2}$). The value of M, the multi-class AUC statistic for each ranking scheme, is above each matrix.

Rank			$p_{ss}(q\|J)$		n			Steady-state
AUC	JIF	Journal abbreviation	$\overline{q}$	σ	$\overline{n}$	Q2	JIF	period
1	1	J ALLERGY CLIN IMMUN	1.39	0.40	30.8	21	8.829	1994–1999
2	3	CLIN EXP ALLERGY	1.27	0.28	30.1	15	3.668	1988–1989
3	5	CONTACT DERMATITIS	1.20	0.35	18.6	13	2.446	1975–1995
4	2	ALLERGY	1.02	0.38	12.7	8	5.334	1995–2001
5	4	INT ARCH ALLERGY IMM	0.90	0.40	10.9	6	2.524	1991–1992
6	6	ALLERGOLOGIE	0.13	0.46	1.5	0	0.237	1981–2003

ANATOMY & MORPHOLOGY

ISI Category Description Anatomy & Morphology includes resources describing the characteristics, generation, and organization of structure in vertebrates or invertebrates. Topics cover embryology, developmental morphology, and functional anatomy, as well as specific structures, systems, or organisms. Resources on plant structure and embryology are placed preferentially in the PLANT SCIENCE category.

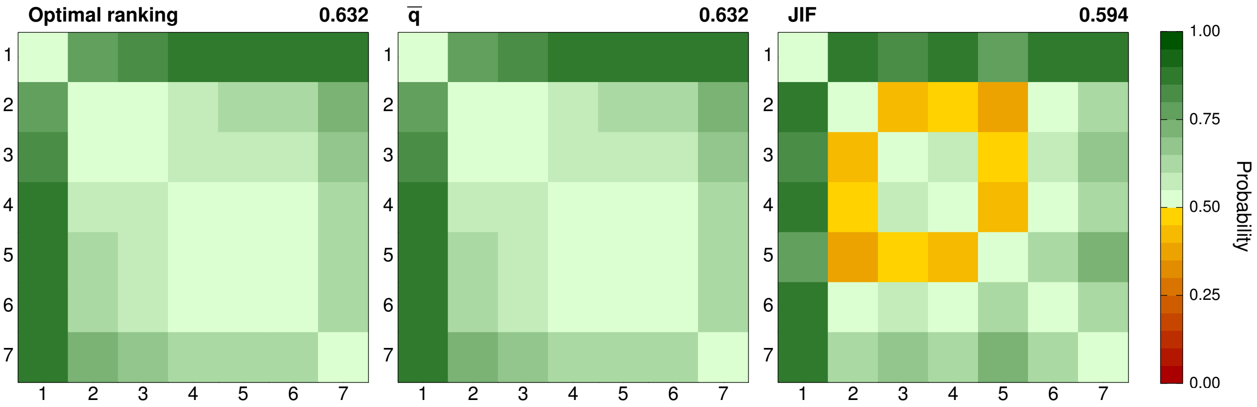

The color of each cell represents the probability of picking a paper with more citations from the higher-ranked journal. Green cells indicate adequate ranking, whereas red cells indicate that the ranking is inadequate (the probability is less than $\frac{1}{2}$). The value of M, the multi-class AUC statistic for each ranking scheme, is above each matrix.

Rank			$\mathbf{p_{ss}(q\|J)}$		n			Steady-state
**AUC**	**JIF**	**Journal abbreviation**	$\overline{\mathbf{q}}$	σ	$\overline{\mathbf{n}}$	**Q2**	**JIF**	**period**
1	1	DEV DYNAM	1.45	0.32	37.0	26	3.169	1991–1997
2	5	ANAT EMBRYOL	1.04	0.35	13.9	9	1.277	1993–1999
3	3	MICROSC RES TECHNIQ	1.00	0.41	12.1	8	1.680	1991–2001
4	4	J MORPHOL	0.91	0.36	9.4	6	1.553	1989–2001
5	2	J ANAT	0.88	0.36	9.1	6	2.458	1998–2002
6	6	TISSUE CELL	0.87	0.33	8.6	6	1.094	1988–1999
7	7	ANN ANAT	0.64	0.41	6.9	2	0.672	1991–1992

ANDROLOGY

ISI Category Description Andrology includes resources focused on the development, function, and disorders of male morphology and reproductive systems. Topics include gonad formation, gamete generation and function, male reproductive health and endocrinology, and sex determination in the male embryo.

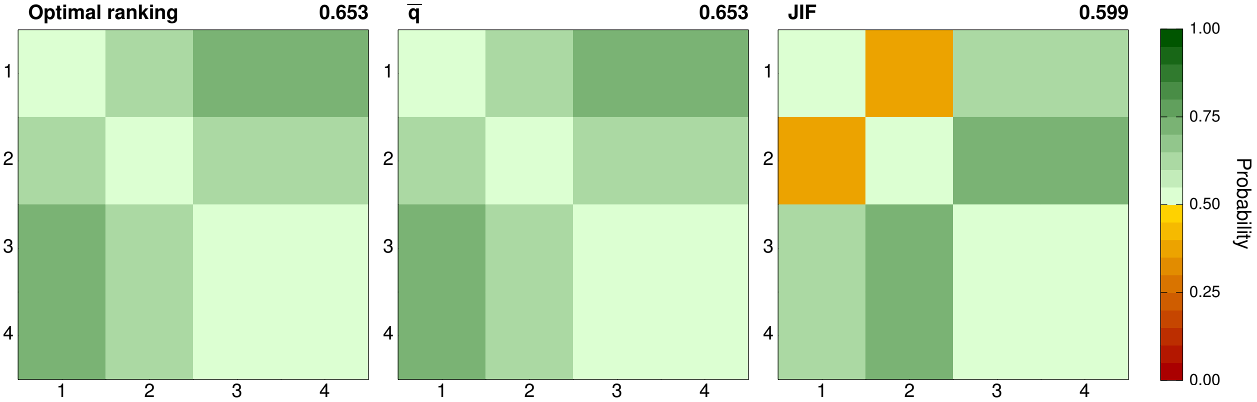

The color of each cell represents the probability of picking a paper with more citations from the higher-ranked journal. Green cells indicate adequate ranking, whereas red cells indicate that the ranking is inadequate (the probability is less than $\frac{1}{2}$). The value of M, the multi-class AUC statistic for each ranking scheme, is above each matrix.

**Rank**			$\mathbf{p_{ss}(q\|J)}$		**n**			**Steady-state**
**AUC**	**JIF**	**Journal abbreviation**	$\overline{\mathbf{q}}$	σ	$\overline{\mathbf{n}}$	**Q2**	**JIF**	**period**
1	2	J ANDROL	1.20	0.42	21.3	13	2.137	1981–1997
2	1	INT J ANDROL	1.03	0.44	13.8	8	2.183	1978–1997
3	3	ANDROLOGIA	0.84	0.40	7.0	5	1.025	1996–1998
4	4	ARCH ANDROLOGY	0.79	0.36	7.4	5	0.687	1978–1998

ANESTHESIOLOGY

ISI Category Description Anesthesiology covers resources that focus on the administration of anesthetics, the treatment of pain, and the use of life support systems. This category also includes specific resources on cardiovascular anesthesia, pediatric anesthesia, and neurosurgical anesthesia.

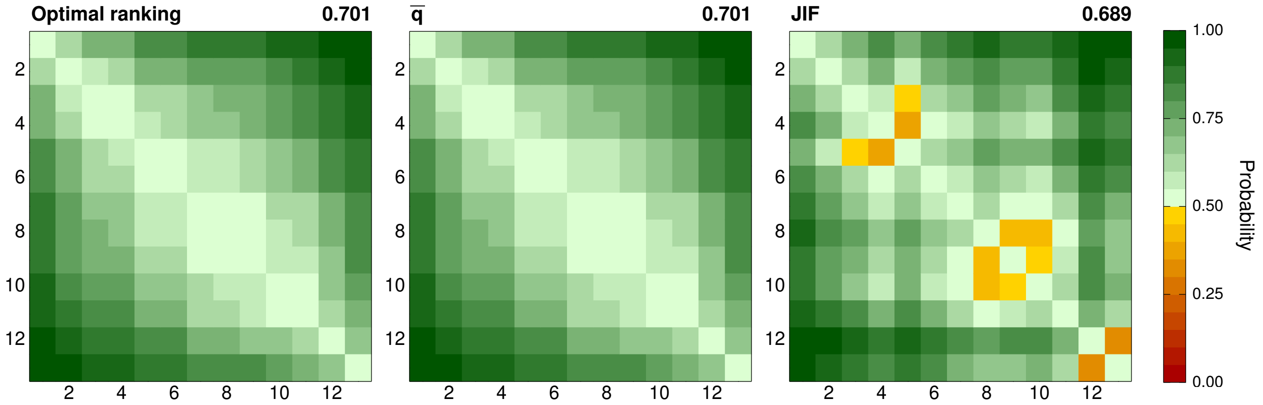

The color of each cell represents the probability of picking a paper with more citations from the higher-ranked journal. Green cells indicate adequate ranking, whereas red cells indicate that the ranking is inadequate (the probability is less than $\frac{1}{2}$). The value of M, the multi-class AUC statistic for each ranking scheme, is above each matrix.

Rank AUC	Rank JIF	Journal abbreviation	$p_{ss}(q\|J)$ $\overline{q}$	σ	n $\overline{n}$	n Q2	JIF	Steady-state period
1	1	PAIN	1.57	0.35	52.5	34	4.836	1979–1996
2	2	ANESTHESIOLOGY	1.40	0.45	35.0	21	4.207	1959–1993
3	5	ANESTH ANALG	1.23	0.39	22.6	14	2.131	1976–1996
4	3	BRIT J ANAESTH	1.19	0.41	21.2	13	2.679	1966–1995
5	4	ANAESTHESIA	1.03	0.42	14.7	8	2.427	1960–1997
6	6	CAN J ANAESTH	1.00	0.38	12.5	8	1.976	1985–1996
7	7	ACTA ANAESTH SCAND	0.91	0.49	11.4	6	1.863	1962–1997
8	10	ANAESTH INTENS CARE	0.87	0.43	9.9	5	0.945	1977–1996
9	9	J CLIN ANESTH	0.80	0.41	8.2	4	1.028	1991–1992
10	8	EUR J ANAESTH	0.73	0.46	5.9	4	1.169	1984–2000
11	11	ANAESTHESIST	0.61	0.43	4.9	3	0.863	1972–2001
12	13	ANN FR ANESTH	0.42	0.40	3.0	2	0.458	1988–2000
13	12	ANASTH INTENSIVMED	0.10	0.49	1.4	0	0.567	1996–2003

ANTHROPOLOGY

ISI Category Description Anthropology covers resources relating to the scientific study of human beings, especially their origin, distribution, behavior, as well as their physical, social and cultural characteristics and development. This category, by definition, borrows from related resources in history, archaeology, and several other social sciences.

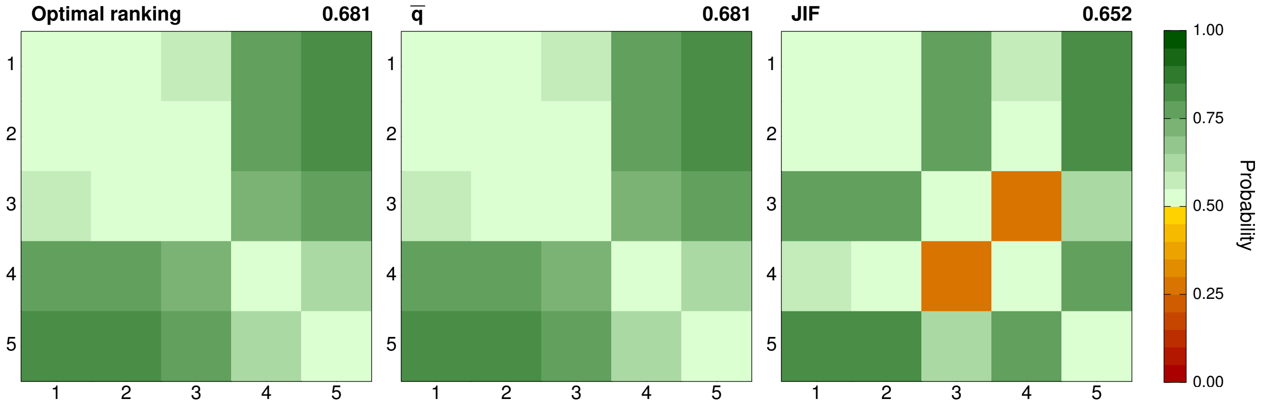

The color of each cell represents the probability of picking a paper with more citations from the higher-ranked journal. Green cells indicate adequate ranking, whereas red cells indicate that the ranking is inadequate (the probability is less than $\frac{1}{2}$). The value of M, the multi-class AUC statistic for each ranking scheme, is above each matrix.

Rank			$p_{ss}(q\|J)$		n			Steady-state
**AUC**	**JIF**	**Journal abbreviation**	$\overline{q}$	σ	$\overline{n}$	**Q2**	**JIF**	**period**
1	1	J HUM EVOL	1.22	0.35	18.1	14	3.267	1989–2000
2	2	AM J PHYS ANTHROPOL	1.19	0.40	19.2	13	2.136	1955–1992
3	4	AM ANTHROPOL	1.13	0.41	19.8	11	1.000	1955–1994
4	3	AM J HUM BIOL	0.76	0.37	6.4	4	1.669	1989–2003
5	5	ANTIQUITY	0.57	0.44	5.1	2	0.454	1955–1999

ASTRONOMY & ASTROPHYSICS

ISI Category Description Astronomy & Astrophysics covers resources that focus on the science of the celestial bodies and their magnitudes, motions, and constitution. Topics include the properties of celestial bodies such as luminosity, size, mass, density, temperature, and chemical composition, as well as their origin and evolution. This category includes some resources on planetary science that focus on astrophysical aspects of planets. General resources on planetary science are placed in the GEOCHEMISTRY & GEOPHYSICS category.

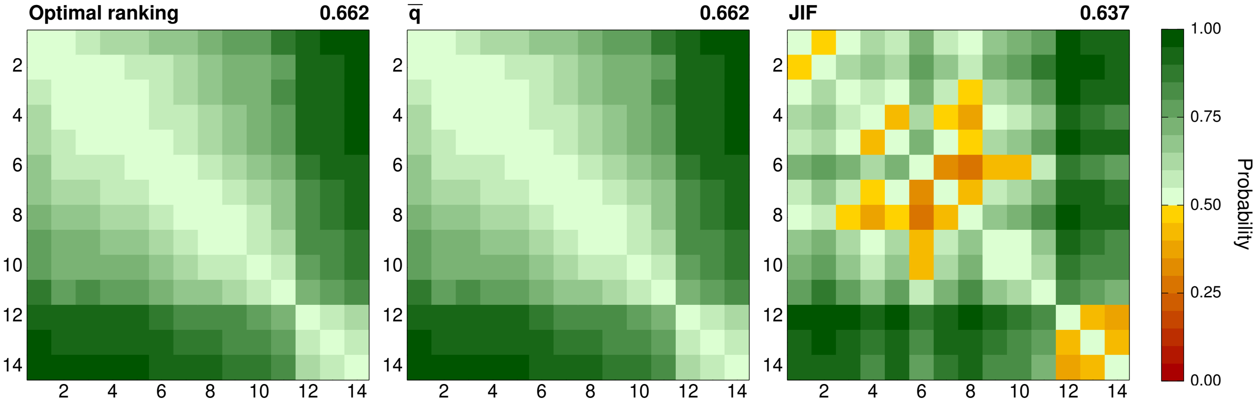

The color of each cell represents the probability of picking a paper with more citations from the higher-ranked journal. Green cells indicate adequate ranking, whereas red cells indicate that the ranking is inadequate (the probability is less than $\frac{1}{2}$). The value of M, the multi-class AUC statistic for each ranking scheme, is above each matrix.

Rank AUC	Rank JIF	Journal abbreviation	$p_{ss}(q\|J)$ $\overline{q}$	σ	n $\overline{n}$	Q2	JIF	Steady-state period
1	2	ASTROPHYS J	1.48	0.40	46.1	27	6.119	1957–1988
2	1	ASTROPHYS J SUPPL S	1.37	0.45	48.7	22	8.627	1992–1995
3	8	ICARUS	1.35	0.35	27.8	19	3.151	1980–1993
4	3	MON NOT R ASTRON SOC	1.32	0.40	32.4	17	5.057	1956–1994
5	5	ASTRON J	1.29	0.40	30.0	16	4.854	1967–1999
6	7	ASTRON ASTROPHYS	1.23	0.40	25.0	14	3.971	1969–1995
7	4	PHYS REV D	1.17	0.47	27.1	12	4.896	1982–1995
8	9	PUBL ASTRON SOC JPN	1.10	0.41	18.6	10	2.106	1970–1997
9	10	SOL PHYS	1.03	0.40	15.9	9	1.887	1985–1995
10	6	PUBL ASTRON SOC PAC	0.93	0.44	14.6	7	4.035	1954–2003
11	11	PLANET SPACE SCI	0.83	0.42	7.9	5	1.509	1995–2001
12	14	EARTH MOON PLANETS	0.38	0.45	3.0	1	0.252	1984–2003
13	13	NUOVO CIMENTO C	0.25	0.48	3.3	1	0.294	1986–1999
14	12	ASTROPHYS SPACE SCI	0.07	0.44	1.3	0	0.771	2000–2006

AUTOMATION & CONTROL SYSTEMS

ISI Category Description Automation & Control Systems covers resources on the design and development of processes and systems that minimize the necessity of human intervention. Resources in this category cover control theory, control engineering, and laboratory and manufacturing automation.

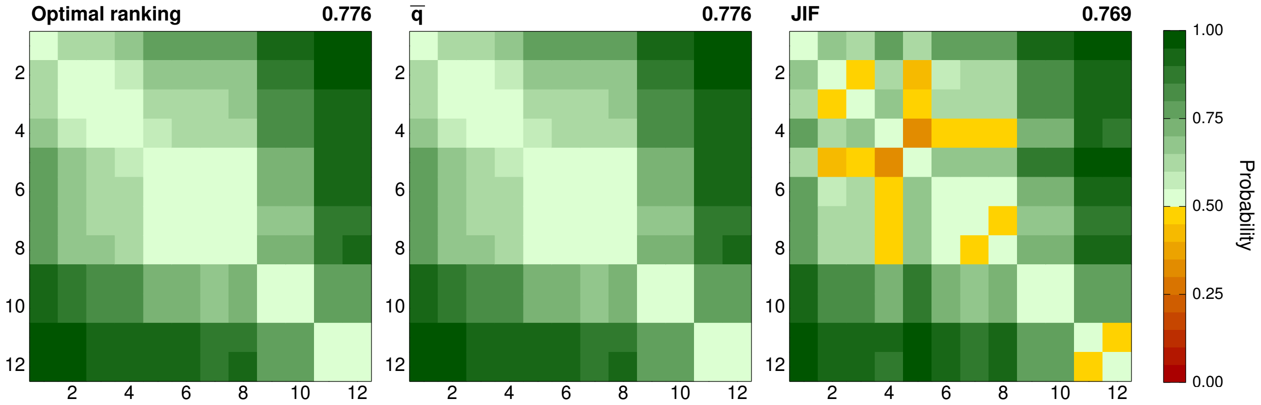

The color of each cell represents the probability of picking a paper with more citations from the higher-ranked journal. Green cells indicate adequate ranking, whereas red cells indicate that the ranking is inadequate (the probability is less than $\frac{1}{2}$). The value of M, the multi-class AUC statistic for each ranking scheme, is above each matrix.

Rank			$p_{ss}(q\|J)$		n			Steady-state
**AUC**	**JIF**	**Journal abbreviation**	$\overline{q}$	σ	$\overline{n}$	**Q2**	**JIF**	**period**
1	1	IEEE T AUTOMAT CONTR	1.31	0.47	40.9	18	2.772	1970–1995
2	5	SIAM J CONTROL OPTIM	1.09	0.43	17.5	10	1.263	1976–1996
3	3	AUTOMATICA	1.04	0.50	23.9	9	2.273	1970–1995
4	2	CHEMOMETR INTELL LAB	0.96	0.42	14.8	8	2.450	1986–2002
5	6	INT J CONTROL	0.79	0.45	10.3	4	0.866	1974–1995
6	8	IEEE T IND ELECTRON	0.76	0.43	7.7	4	0.590	1988–1998
7	7	J DYN SYST-T ASME	0.75	0.52	9.1	4	0.658	1982–1993
8	4	SYST CONTROL LETT	0.71	0.41	7.6	4	1.683	1994–2000
9	9	J FRANKLIN I	0.31	0.45	2.3	1	0.362	1985–2004
10	10	INT J SYST SCI	0.28	0.43	2.3	1	0.343	1986–2001
11	12	CONTROL ENG	-0.37	0.38	0.2	0	0.051	1966–2005
12	11	AUTOMAT REM CONTR+	-0.51	0.44	0.2	0	0.251	1990–2005

BEHAVIORAL SCIENCES

ISI Category Description Behavioral Sciences covers resources dealing with the biological correlates of observable action in humans or animals. These include sleep, aggression, sexual behavior, and learning as well as the various factors, natural or pharmacological, that alter such behaviors. Resources in this category cover neurobiology, experimental psychology, ethology, cognitive assessment, and behavioral consequences of neurological disorders.

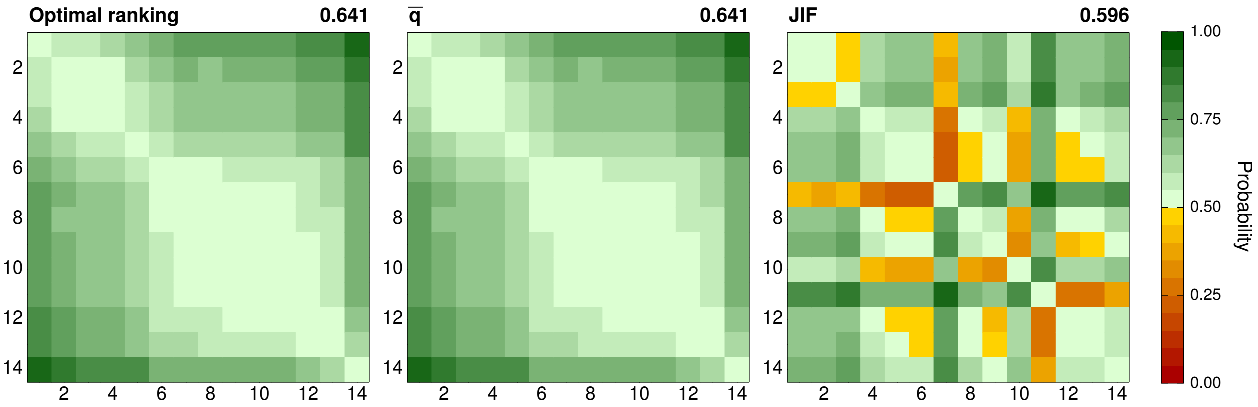

The color of each cell represents the probability of picking a paper with more citations from the higher-ranked journal. Green cells indicate adequate ranking, whereas red cells indicate that the ranking is inadequate (the probability is less than $\frac{1}{2}$). The value of M, the multi-class AUC statistic for each ranking scheme, is above each matrix.

Rank			$p_{ss}(q\|J)$		n			Steady-state
**AUC**	**JIF**	**Journal abbreviation**	$\overline{q}$	σ	$\overline{n}$	**Q2**	**JIF**	**period**
1	7	BEHAV ECOL SOCIOBIOL	1.60	0.31	44.4	36	2.316	1978–1990
2	3	ANIM BEHAV	1.51	0.33	38.6	28	2.711	1975–1993
3	1	NEUROPSYCHOLOGIA	1.48	0.41	48.6	27	3.924	1964–1995
4	2	BEHAV NEUROSCI	1.45	0.36	39.2	25	2.907	1984–1996
5	10	J COMP PHYSIOL A	1.37	0.31	26.4	21	1.751	1984–1989
6	4	BEHAV BRAIN RES	1.28	0.40	26.4	16	2.591	1979–1998
7	8	ETHOLOGY	1.25	0.34	19.7	15	2.245	1985–1993
8	12	J EXP ANAL BEHAV	1.22	0.38	23.8	14	1.221	1970–1991
9	5	PHYSIOL BEHAV	1.20	0.37	20.5	13	2.445	1975–1991
10	13	BEHAVIOUR	1.21	0.33	18.4	13	1.165	1988–1998
11	6	BEHAV PHARMACOL	1.20	0.34	17.9	13	2.388	1990–1998
12	9	PHARMACOL BIOCHEM BE	1.14	0.35	17.0	11	2.092	1991–1997
13	14	HUM FACTORS	1.08	0.40	15.6	10	0.861	1980–1996
14	11	BEHAV PROCESS	0.87	0.39	9.0	5	1.478	1975–2000

BIOCHEMICAL RESEARCH METHODS

ISI Category Description Biochemical Research Methods includes resources that describe specific techniques used in biological and biochemical research, including methods for the purification and analysis of biomolecules, the observation of the structure or function of living organisms and tissues (exclusive of microscopy), and the alteration of biomolecules for specific research applications. This category does not cover clinical applications or the development and design of diagnostic tools.

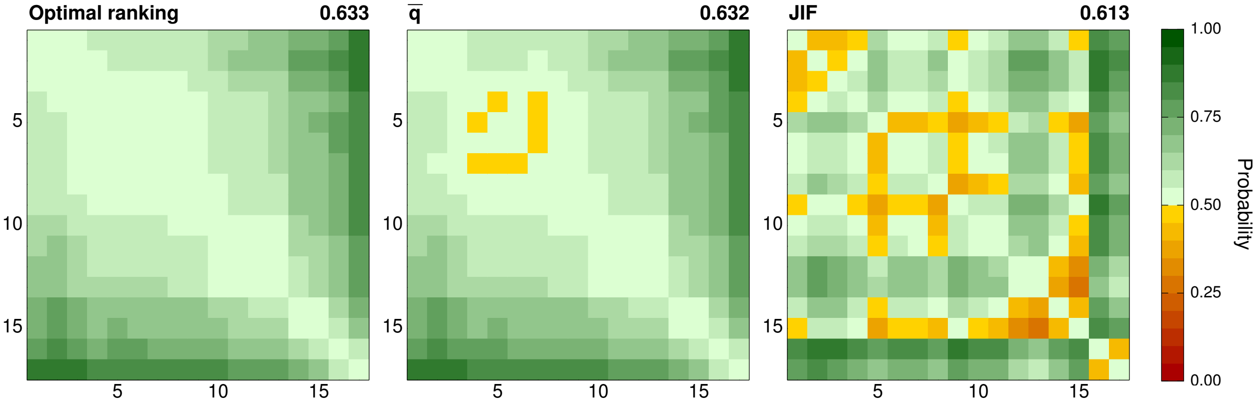

The color of each cell represents the probability of picking a paper with more citations from the higher-ranked journal. Green cells indicate adequate ranking, whereas red cells indicate that the ranking is inadequate (the probability is less than $\frac{1}{2}$). The value of M, the multi-class AUC statistic for each ranking scheme, is above each matrix.

**Rank**			$\mathbf{p_{ss}(q\|J)}$		**n**			**Steady-state**
**AUC**	**JIF**	**Journal abbreviation**	$\overline{\mathbf{q}}$	σ	$\overline{\mathbf{n}}$	**Q2**	**JIF**	**period**
1	3	ANAL BIOCHEM	1.28	0.47	67.8	15	2.948	1959–1989
2	2	BIOCONJUGATE CHEM	1.25	0.34	23.2	15	3.823	1990–2000
3	9	J MAGN RESON	1.19	0.40	30.0	13	2.076	1970–1992
4	4	BIOTECHNIQUES	1.15	0.46	45.6	12	2.462	1985–1994
5	15	J CHROMATOGR SCI	1.16	0.38	20.8	12	0.880	1968–1995
6	1	ELECTROPHORESIS	1.16	0.40	19.8	12	4.101	1994–1996
7	6	J IMMUNOL METHODS	1.16	0.41	28.4	11	2.402	1971–1997
8	7	J NEUROSCI METH	1.13	0.42	28.2	11	2.243	1980–1994
9	10	MOL CELL PROBE	1.11	0.35	17.5	10	2.016	1989–1994
10	11	PROTEIN EXPRES PURIF	1.07	0.35	12.8	9	1.867	1991–1998
11	8	J VIROL METHODS	1.03	0.37	14.1	9	2.097	1979–2000
12	14	CHROMATOGRAPHIA	1.02	0.35	13.8	8	1.171	1974–1998
13	5	J MICROBIOL METH	1.01	0.38	12.2	8	2.442	1998–2001
14	12	BIOMED CHROMATOGR	0.90	0.39	8.3	6	1.611	1988–1999
15	13	J BIOCHEM BIOPH METH	0.83	0.39	9.5	5	1.403	1986–2001
16	17	HYBRIDOMA	0.69	0.56	7.0	3	0.411	1986–2000
17	16	J LABELLED COMPD RAD	0.52	0.38	4.0	2	0.746	1978–2003

BIOCHEMISTRY & MOLECULAR BIOLOGY

ISI Category Description Biochemistry & Molecular Biology covers resources on general biochemistry and molecular biology topics such as carbohydrates, lipids, proteins, nucleic acids, genes, drugs, toxic substances, and other chemical or molecular constituents of cells, microbes, and higher plants and animals, including humans. Excluded are resources that are focus on biochemistry in cells, tissues or organs and those whose primary focus is the organism of study, e.g. plants, microbes, etc. Excluded, also, are resources that focus on methods in biochemistry or molecular biology.

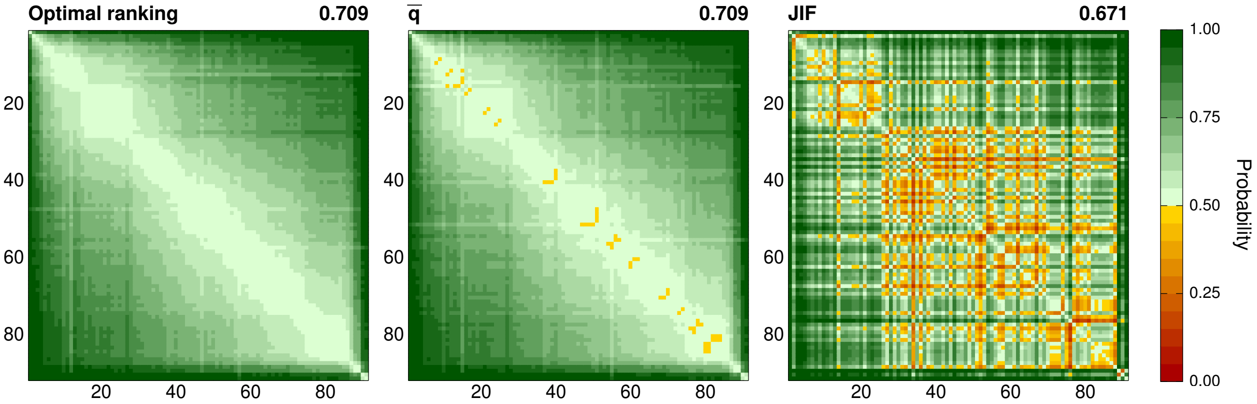

The color of each cell represents the probability of picking a paper with more citations from the higher-ranked journal. Green cells indicate adequate ranking, whereas red cells indicate that the ranking is inadequate (the probability is less than $\frac{1}{2}$). The value of M, the multi-class AUC statistic for each ranking scheme, is above each matrix.

Rank			$p_{ss}(q\|J)$		n			Steady-state
AUC	JIF	Journal abbreviation	$\overline{q}$	σ	$\overline{n}$	Q2	JIF	period
1	1	CELL	2.35	0.30	306.3	216	29.194	1994–1997
2	3	EMBO J	1.97	0.33	119.7	85	10.086	1986–1993
3	4	PLANT CELL	1.87	0.34	92.4	72	9.868	1988–1995
4	5	MOL CELL BIOL	1.80	0.33	82.7	57	6.773	1986–1995
5	13	J MOL BIOL	1.70	0.39	89.0	45	4.890	1972–1993
6	10	J BIOL CHEM	1.69	0.33	62.9	45	5.808	1990–1993
7	8	ONCOGENE	1.66	0.36	60.4	41	6.582	1992–1995
8	6	MOL BIOL EVOL	1.60	0.35	72.9	35	6.726	1983–1998
9	23	BIOCHEMISTRY-US	1.60	0.33	52.1	35	3.633	1989–1991
10	7	FASEB J	1.59	0.47	62.0	36	6.721	1986–1998
11	22	PROTEINS	1.53	0.38	69.4	34	3.730	1986–1994
12	2	TRENDS BIOCHEM SCI	1.51	0.56	72.3	36	13.863	1988–2000
13	11	MOL MICROBIOL	1.57	0.32	43.8	33	5.634	1988–1999
14	20	MOL PLANT MICROBE IN	1.52	0.31	41.0	34	3.936	1988–1993
15	16	J LIPID RES	1.52	0.35	44.4	29	4.357	1983–1992
16	28	PROTEIN SCI	1.48	0.38	46.4	29	3.462	1991–1993
17	15	AM J RESP CELL MOL	1.48	0.33	38.3	26	4.593	1988–1998
18	24	EUR J BIOCHEM	1.48	0.36	42.9	26	3.579	1972–1985
19	54	J MEMBRANE BIOL	1.47	0.38	41.2	27	2.112	1973–1988
20	17	J NEUROCHEM	1.47	0.36	41.2	26	4.260	1957–1997
21	9	NUCLEIC ACIDS RES	1.46	0.36	48.6	26	6.317	1992–1994

table continues on next page . . .

. . . table continued from previous page

Rank			$p_{ss}(q\|J)$		n			Steady-state
AUC	JIF	Journal abbreviation	$\overline{q}$	σ	$\overline{n}$	Q2	JIF	period
22	25	PLANT MOL BIOL	1.47	0.33	34.9	26	3.577	1989–1994
23	12	FREE RADICAL BIO MED	1.46	0.36	37.4	24	5.440	1986–1997
24	43	PEPTIDES	1.45	0.38	35.4	26	2.701	1979–1983
25	67	J BIOMOL NMR	1.46	0.37	73.5	25	1.791	1991–1995
26	62	DNA CELL BIOL	1.44	0.36	45.5	25	1.905	1989–1991
27	45	MOL BIOCHEM PARASIT	1.44	0.28	31.1	24	2.641	1991–1994
28	18	BIOCHEM J	1.43	0.35	36.7	23	4.100	1982–1990
29	19	CHROMOSOMA	1.38	0.37	34.4	21	4.065	1955–1996
30	40	J MOL EVOL	1.37	0.38	40.4	20	2.767	1971–1999
31	41	MOL CARCINOGEN	1.35	0.36	29.1	20	2.743	1988–1994
32	30	FEBS LETT	1.35	0.38	31.4	19	3.372	1983–1995
33	48	MOL REPROD DEV	1.34	0.37	26.9	18	2.379	1989–1995
34	37	BIOCHEM BIOPH RES CO	1.32	0.40	32.6	18	2.855	1977–1992
35	55	PHOTOCHEM PHOTOBIOL	1.32	0.38	28.3	18	2.061	1961–1991
36	33	ARCH BIOCHEM BIOPHYS	1.30	0.36	27.0	17	2.969	1981–1995
37	50	CYTOKINE	1.25	0.36	26.1	16	2.355	1990–1994
38	35	ANAL BIOCHEM	1.28	0.47	67.8	15	2.948	1959–1989
39	78	J BIOMOL STRUCT DYN	1.25	0.38	26.6	15	1.299	1982–1989
40	21	BIOCONJUGATE CHEM	1.25	0.34	23.2	15	3.823	1990–2000
41	69	CARBOHYD RES	1.23	0.36	23.6	14	1.703	1967–1989
42	59	YEAST	1.20	0.34	25.4	14	1.955	1992–1994
43	88	FISH PHYSIOL BIOCHEM	1.20	0.36	19.1	14	0.558	1986–1995
44	60	LIPIDS	1.20	0.35	19.3	13	1.935	1990–1995
45	26	J STRUCT BIOL	1.19	0.36	23.0	13	3.496	1989–2001
46	46	BIOPOLYMERS	1.19	0.36	18.8	13	2.480	1993–1999
47	47	BIOTECHNIQUES	1.15	0.46	45.6	12	2.462	1985–1994
48	42	INSECT BIOCHEM MOLEC	1.18	0.35	17.2	13	2.711	1991–1999
49	39	J STEROID BIOCHEM	1.17	0.36	19.5	12	2.825	1972–2001
50	74	ARCH INSECT BIOCHEM	1.18	0.35	16.9	13	1.474	1982–1994
51	81	PESTIC BIOCHEM PHYS	1.16	0.38	18.2	12	1.189	1975–1993
52	66	CHEM-BIOL INTERACT	1.15	0.35	17.8	12	1.800	1985–1995
53	14	MOL IMMUNOL	1.14	0.37	17.9	11	4.768	1978–1999
54	56	MOL CELL PROBE	1.11	0.35	17.5	10	2.016	1989–1994
55	64	J CHEM ECOL	1.11	0.31	14.4	10	1.896	1995–1998
56	79	CAN J MICROBIOL	1.10	0.39	17.3	11	1.275	1954–1995
57	29	J CELL BIOCHEM	1.11	0.55	19.0	11	3.409	1994–1999
58	61	J PHOTOCH PHOTOBIO B	1.10	0.37	15.2	10	1.909	1987–1998
59	44	J INORG BIOCHEM	1.08	0.35	13.8	10	2.654	1984–2000
60	77	INT J BIOL MACROMOL	1.07	0.38	15.5	9	1.323	1978–2000
61	31	BIOCHIMIE	1.06	0.39	16.3	9	3.237	1971–2002
62	65	PROTEIN EXPRES PURIF	1.07	0.35	12.8	9	1.867	1991–1998
63	68	BIOPHYS CHEM	1.04	0.37	14.1	9	1.784	1982–1999
64	53	NEUROCHEM RES	1.03	0.37	14.0	9	2.139	1978–1997

table continues on next page . . .

. . . table continued from previous page

Rank			$p_{ss}(q\|J)$		n			Steady-state
**AUC**	**JIF**	**Journal abbreviation**	$\overline{q}$	σ	$\overline{n}$	**Q2**	**JIF**	**period**
65	38	STEROIDS	1.02	0.35	13.5	8	2.849	1986–2001
66	32	NEUROCHEM INT	1.02	0.36	12.9	8	3.159	1981–2002
67	80	BIOSCI BIOTECH BIOCH	1.00	0.35	12.1	8	1.256	1991–1994
68	27	BIOCHEM CELL BIOL	1.00	0.36	13.1	8	3.483	1985–2000
69	49	CHEM PHYS LIPIDS	1.00	0.36	12.1	8	2.371	1994–2002
70	70	PLANT SCI	1.00	0.32	11.0	8	1.631	1993–2001
71	63	BIOTECHNOL APPL BIOC	0.98	0.37	11.4	7	1.903	1986–1999
72	87	Z NATURFORSCH C	0.96	0.35	11.6	7	0.720	1987–1991
73	51	MAMM GENOME	0.96	0.39	12.5	7	2.279	1997–2002
74	57	PROCESS BIOCHEM	0.96	0.37	10.3	7	2.008	1990–2000
75	83	BIOL TRACE ELEM RES	0.91	0.37	10.1	6	1.007	1979–1998
76	58	COMP BIOCHEM PHYS C	0.89	0.37	9.5	6	1.991	1985–2002
77	86	BIOCHEM GENET	0.90	0.40	9.4	6	0.876	1986–1997
78	85	BIOCHEM SYST ECOL	0.89	0.36	9.2	6	0.906	1980–1994
79	71	BIOMED CHROMATOGR	0.90	0.39	8.3	6	1.611	1988–1999
80	36	J NUTR BIOCHEM	0.87	0.37	9.1	6	2.945	1989–2002
81	72	COMP BIOCHEM PHYS A	0.87	0.36	8.6	6	1.553	1986–2002
82	73	COMP BIOCHEM PHYS B	0.87	0.35	8.6	6	1.532	1987–2001
83	84	CELL MOL BIOL	0.87	0.36	8.5	5	0.959	1990–1995
84	82	APPL BIOCHEM BIOTECH	0.87	0.40	9.3	6	1.102	1981–1996
85	75	J BIOCHEM BIOPH METH	0.83	0.39	9.5	5	1.403	1986–2001
86	52	PROSTAG LEUKOTR ESS	0.80	0.34	7.1	5	2.261	2000–2003
87	34	BIOCHEM SOC T	0.69	0.49	7.7	3	2.962	1974–2006
88	90	INDIAN J BIOCHEM BIO	0.49	0.39	3.6	2	0.277	1974–1999
89	76	BIOCHEMISTRY-MOSCOW+	0.47	0.34	2.9	2	1.368	1998–2002
90	89	MOL BIOL+	-0.25	0.51	0.6	0	0.330	1981–2000
91	91	J EVOL BIOCHEM PHYS+	-0.76	0.61	0.2	0	0.206	1983–2003

BIODIVERSITY CONSERVATION

ISI Category Description Biodiversity Conservation covers resources on the conservation management of species and ecosystems. Topics include conservation ecology, biological conservation, paleobiology, natural history and the natural sciences.

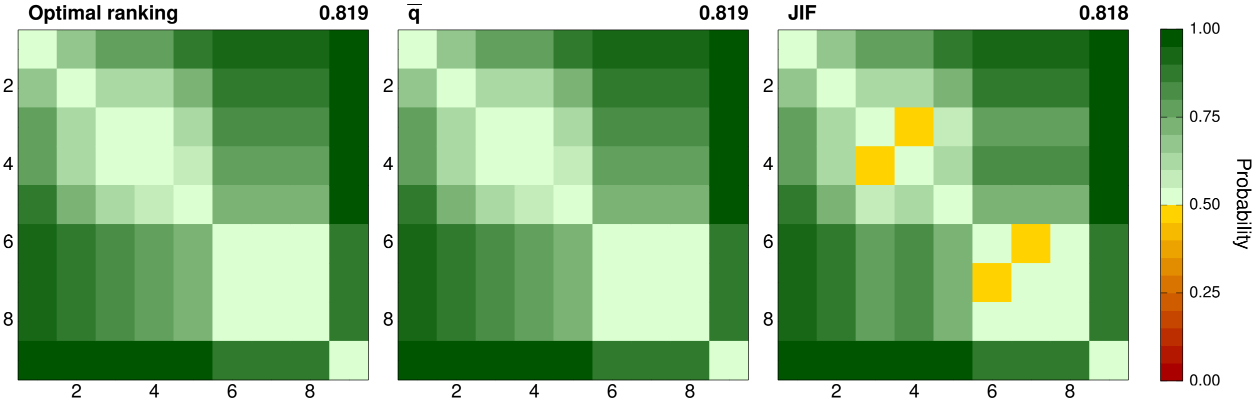

The color of each cell represents the probability of picking a paper with more citations from the higher-ranked journal. Green cells indicate adequate ranking, whereas red cells indicate that the ranking is inadequate (the probability is less than $\frac{1}{2}$). The value of M, the multi-class AUC statistic for each ranking scheme, is above each matrix.

Rank			$p_{ss}(q\|J)$		n			Steady-state
**AUC**	**JIF**	**Journal abbreviation**	$\overline{q}$	σ	$\overline{n}$	**Q2**	**JIF**	**period**
1	1	AM NAT	1.72	0.40	80.4	48	4.660	1967–1992
2	2	CONSERV BIOL	1.42	0.42	37.5	23	3.762	1988–1998
3	4	POLAR BIOL	1.27	0.33	20.8	16	1.502	1981–1994
4	3	BIOL CONSERV	1.21	0.38	22.1	14	2.854	1988–1996
5	5	AM MIDL NAT	1.07	0.36	14.5	9	0.667	1964–1995
6	7	SOUTHWEST NAT	0.72	0.38	5.9	4	0.309	1980–1994
7	6	J NAT HIST	0.72	0.38	6.4	4	0.631	1966–2000
8	8	CAN FIELD NAT	0.69	0.40	5.8	3	0.073	1983–1993
9	9	NAT HIST	-0.32	0.44	0.3	0	0.059	1989–2005

BIOLOGY

ISI Category Description The Biology category includes resources having a broad or interdisciplinary approach to biology. In addition, it includes materials that cover a specific area of biology not covered in other categories such as theoretical biology, mathematical biology, thermal biology, cryobiology, and biological rhythm research.

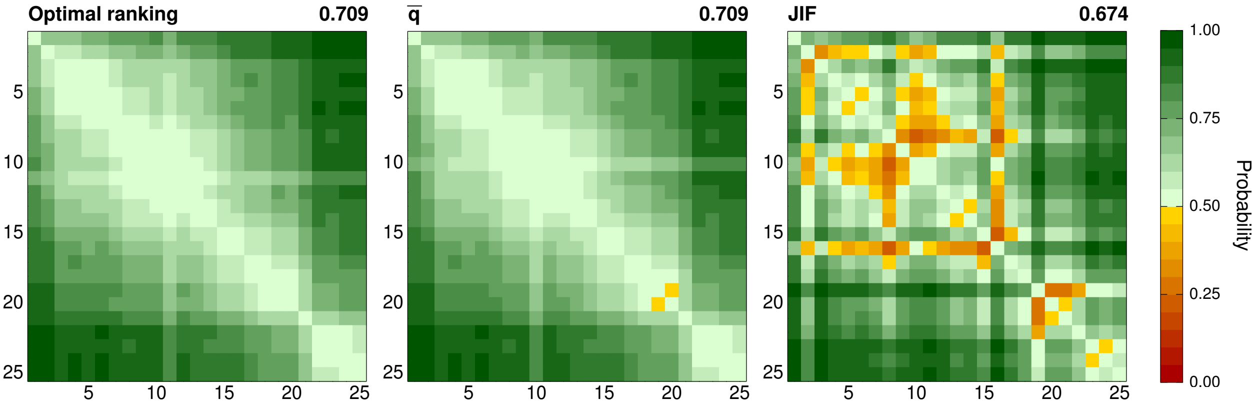

The color of each cell represents the probability of picking a paper with more citations from the higher-ranked journal. Green cells indicate adequate ranking, whereas red cells indicate that the ranking is inadequate (the probability is less than $\frac{1}{2}$). The value of M, the multi-class AUC statistic for each ranking scheme, is above each matrix.

Rank AUC	Rank JIF	Journal abbreviation	$p_{ss}(q\|J)$ $\overline{q}$	σ	n $\overline{n}$	n Q2	JIF	Steady-state period
1	1	FASEB J	1.59	0.47	62.0	36	6.721	1986–1998
2	3	J EXP BIOL	1.35	0.33	25.4	19	2.631	1985–1994
3	10	BIOMETRICS	1.25	0.46	48.1	14	1.489	1954–1994
4	16	BIOMETRIKA	1.22	0.42	36.8	13	1.014	1955–1996
5	4	RADIAT RES	1.17	0.40	21.8	12	2.602	1954–1998
6	11	INT J RADIAT BIOL	1.16	0.38	20.4	12	1.312	1972–1999
7	6	CRYOBIOLOGY	1.11	0.39	17.1	10	2.162	1963–1997
8	9	J MATH BIOL	1.04	0.41	16.5	9	1.664	1975–1998
9	5	J THEOR BIOL	1.03	0.41	16.3	9	2.264	1980–1998
10	12	HUM BIOL	1.00	0.38	13.2	8	1.132	1977–1999
11	2	BIOSCIENCE	1.03	0.64	27.9	8	5.424	1971–2002
12	7	MICROSC RES TECHNIQ	1.00	0.41	12.1	8	1.680	1991–2001
13	14	BIOSYSTEMS	0.94	0.44	12.3	7	1.080	1973–1996
14	13	MATH BIOSCI	0.89	0.44	12.4	6	1.126	1975–2001
15	17	J THERM BIOL	0.80	0.39	7.6	5	0.950	1976–2000
16	8	AM J HUM BIOL	0.76	0.37	6.4	4	1.669	1989–2003
17	15	BRAZ J MED BIOL RES	0.66	0.38	5.4	3	1.075	1981–2000
18	18	P JPN ACAD B-PHYS	0.62	0.43	6.9	3	0.808	1976–2002
19	21	P BIOL SOC WASH	0.55	0.36	3.8	2	0.347	1983–1998
20	20	FOLIA BIOL-PRAGUE	0.55	0.40	4.1	2	0.387	1973–2000
21	22	REV BIOL TROP	0.35	0.43	2.8	1	0.217	1975–1998
22	19	ZH OBSHCH BIOL	0.10	0.40	1.0	0	0.391	1998–2006
23	24	PERIOD BIOL	0.08	0.44	1.3	0	0.205	1974–2001
24	23	BIOLOGIA	0.01	0.40	0.9	0	0.213	2002–2006
25	25	AM BIOL TEACH	-0.04	0.43	1.0	0	0.171	1969–2005

BIOPHYSICS

ISI Category Description Biophysics covers resources that focus on the transfer and effects of physical forces and energy-light, sound, electricity, magnetism, heat, cold, pressure, mechanical forces, and radiation-within and on cells, tissues, and whole organisms.

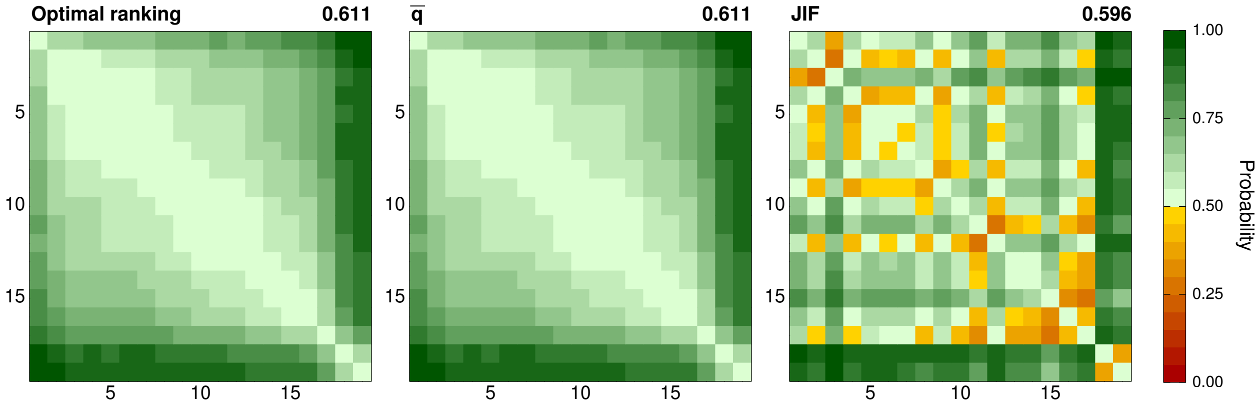

The color of each cell represents the probability of picking a paper with more citations from the higher-ranked journal. Green cells indicate adequate ranking, whereas red cells indicate that the ranking is inadequate (the probability is less than $\frac{1}{2}$). The value of M, the multi-class AUC statistic for each ranking scheme, is above each matrix.

Rank			$p_{ss}(q\|J)$		n			Steady-state
AUC	JIF	Journal abbreviation	$\overline{q}$	σ	$\overline{n}$	Q2	JIF	period
1	3	PROTEINS	1.53	0.38	69.4	34	3.730	1986–1994
2	1	BIOPHYS J	1.42	0.40	38.1	23	4.757	1962–1997
3	9	J BIOMECH	1.38	0.43	32.6	20	2.542	1968–1995
4	5	FEBS LETT	1.35	0.38	31.4	19	3.372	1983–1995
5	7	BIOCHEM BIOPH RES CO	1.32	0.40	32.6	18	2.855	1977–1992
6	12	PHOTOCHEM PHOTOBIOL	1.32	0.38	28.3	18	2.061	1961–1991
7	6	ARCH BIOCHEM BIOPHYS	1.30	0.36	27.0	17	2.969	1981–1995
8	17	J BIOMOL STRUCT DYN	1.25	0.38	26.6	15	1.299	1982–1989
9	2	BIOSENS BIOELECTRON	1.25	0.41	21.1	15	4.132	1990–2000
10	4	J STRUCT BIOL	1.19	0.36	23.0	13	3.496	1989–2001
11	10	BIOPOLYMERS	1.19	0.36	18.8	13	2.480	1993–1999
12	8	RADIAT RES	1.17	0.40	21.8	12	2.602	1954–1998
13	16	J BIOMECH ENG-T ASME	1.14	0.40	20.2	11	1.309	1978–1999
14	13	J PHOTOCH PHOTOBIO B	1.10	0.37	15.2	10	1.909	1987–1998
15	14	BIOPHYS CHEM	1.04	0.37	14.1	9	1.784	1982–1999
16	11	CHEM PHYS LIPIDS	1.00	0.36	12.1	8	2.371	1994–2002
17	15	J BIOCHEM BIOPH METH	0.83	0.39	9.5	5	1.403	1986–2001
18	19	INDIAN J BIOCHEM BIO	0.49	0.39	3.6	2	0.277	1974–1999
19	18	BIOFIZIKA+	0.29	0.41	2.1	1	0.435	1987–1998

BIOTECHNOLOGY & APPLIED MICROBIOLOGY

ISI Category Description Biotechnology & Applied Microbiology includes resources that cover a broad range of topics on the manipulation of living organisms to make products or solve problems to meet human needs. Topics include genetic engineering; molecular diagnostic and therapeutic techniques; genome data mining; bioprocessing of food and drugs; biological control of pests; environmental bioremediation; and bio-energy production. This category also covers resources that deal with the related social, business, and regulatory issues.

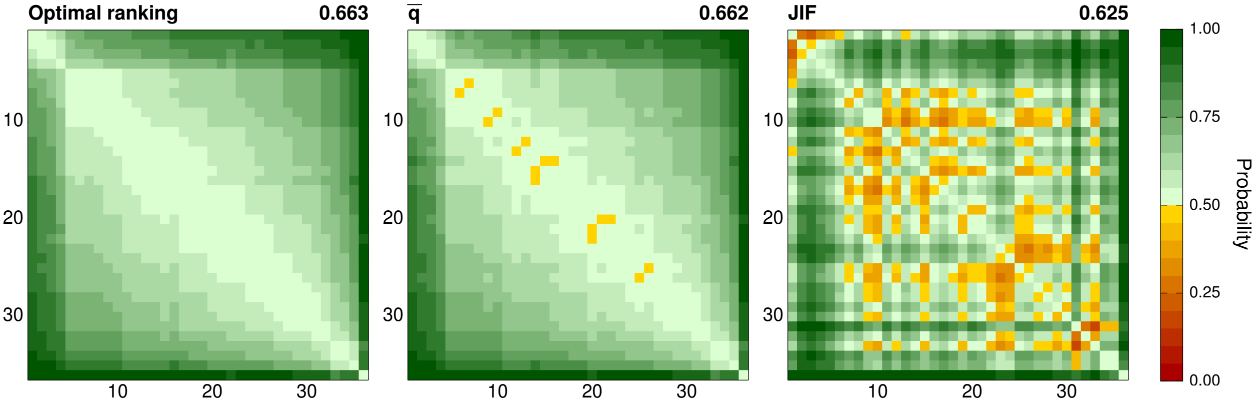

The color of each cell represents the probability of picking a paper with more citations from the higher-ranked journal. Green cells indicate adequate ranking, whereas red cells indicate that the ranking is inadequate (the probability is less than $\frac{1}{2}$). The value of M, the multi-class AUC statistic for each ranking scheme, is above each matrix.

Rank			$p_{ss}(q\|J)$		n			Steady-state
AUC	JIF	Journal abbreviation	$\overline{q}$	σ	$\overline{n}$	Q2	JIF	period
1	3	GENOMICS	1.61	0.31	54.6	35	3.558	1987–1991
2	2	MOL PLANT MICROBE IN	1.52	0.31	41.0	34	3.936	1988–1993
3	4	APPL ENVIRON MICROB	1.51	0.35	42.7	28	3.532	1990–1993
4	5	J GEN VIROL	1.42	0.37	34.8	22	3.110	1985–1991
5	6	BIOTECHNOL BIOENG	1.28	0.45	24.6	16	2.999	1965–1995
6	13	SYST APPL MICROBIOL	1.24	0.39	24.7	14	2.037	1985–1994
7	1	BIOSENS BIOELECTRON	1.25	0.41	21.1	15	4.132	1990–2000
8	17	YEAST	1.20	0.34	25.4	14	1.955	1992–1994
9	11	BIOTECHNOL PROGR	1.19	0.35	19.2	13	2.102	1984–1998
10	8	APPL MICROBIOL BIOT	1.19	0.36	18.7	13	2.441	1983–1993
11	16	GENOME	1.14	0.38	19.0	11	1.972	1992–1997
12	26	J ANTIBIOT	1.13	0.37	18.6	11	1.262	1963–1992
13	18	J FOOD PROTECT	1.13	0.36	16.0	11	1.921	1992–1999
14	14	MOL CELL PROBE	1.11	0.35	17.5	10	2.016	1989–1994
15	25	CAN J MICROBIOL	1.10	0.39	17.3	11	1.275	1954–1995
16	20	ENZYME MICROB TECH	1.11	0.31	13.5	11	1.897	1994–2000
17	7	J BIOTECHNOL	1.07	0.34	14.0	10	2.600	1984–2001
18	21	PROTEIN EXPRES PURIF	1.07	0.35	12.8	9	1.867	1991–1998
19	33	PLANT CELL TISS ORG	1.06	0.35	13.1	9	0.951	1985–1994
20	12	J VIROL METHODS	1.03	0.37	14.1	9	2.097	1979–2000
21	28	BIOTECHNOL LETT	1.02	0.36	12.7	8	1.134	1983–1992
22	30	AM J ENOL VITICULT	1.03	0.42	13.9	8	1.009	1967–2000

table continues on next page . . .

. . . table continued from previous page

Rank			$p_{ss}(q\|J)$		n			Steady-state
AUC	JIF	Journal abbreviation	$\overline{q}$	σ	$\overline{n}$	Q2	JIF	period
23	27	BIOSCI BIOTECH BIOCH	1.00	0.35	12.1	8	1.256	1991–1994
24	19	BIOTECHNOL APPL BIOC	0.98	0.37	11.4	7	1.903	1986–1999
25	9	MAMM GENOME	0.96	0.39	12.5	7	2.279	1997–2002
26	15	PROCESS BIOCHEM	0.96	0.37	10.3	7	2.008	1990–2000
27	22	LETT APPL MICROBIOL	0.93	0.38	11.5	7	1.593	1984–2000
28	10	BIORESOURCE TECHNOL	0.90	0.37	9.9	6	2.180	1991–2001
29	32	PLANT BREEDING	0.89	0.39	9.2	6	0.954	1985–1996
30	29	APPL BIOCHEM BIOTECH	0.87	0.40	9.3	6	1.102	1981–1996
31	24	J CHEM TECHNOL BIOT	0.84	0.41	9.4	5	1.276	1978–1999
32	23	BIOMASS BIOENERG	0.80	0.39	7.0	5	1.483	1990–2002
33	35	HYBRIDOMA	0.69	0.56	7.0	3	0.411	1986–2000
34	34	WORLD J MICROB BIOT	0.65	0.41	5.5	3	0.471	1989–1998
35	31	FOLIA MICROBIOL	0.53	0.37	3.4	2	0.963	1985–2002
36	36	BIOFUTUR	-0.40	0.42	0.2	0	0.059	1991–2004

BUSINESS, FINANCE

ISI Category Description Business, Finance covers resources primarily concerned with financial and economic correlations, accounting, financial management, investment strategies, the international monetary system, insurance, taxation, and banking.

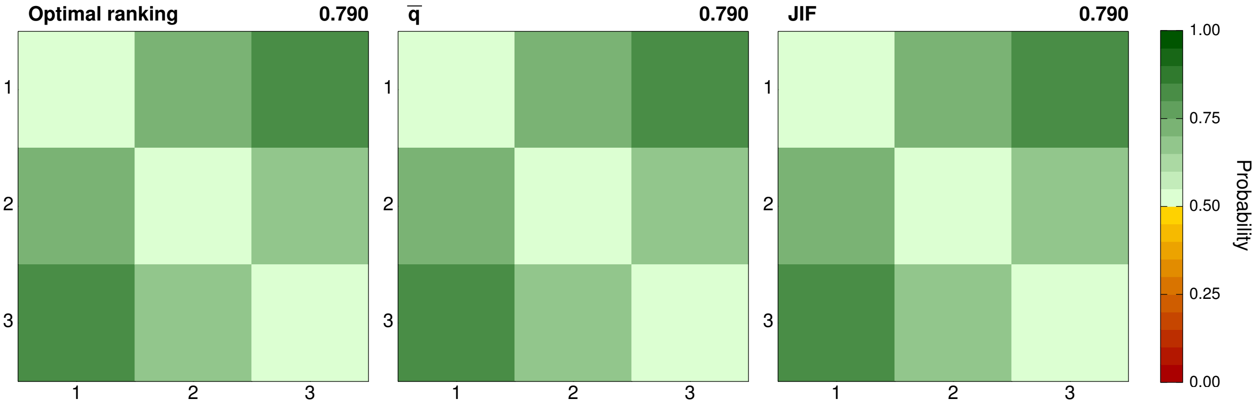

The color of each cell represents the probability of picking a paper with more citations from the higher-ranked journal. Green cells indicate adequate ranking, whereas red cells indicate that the ranking is inadequate (the probability is less than $\frac{1}{2}$). The value of M, the multi-class AUC statistic for each ranking scheme, is above each matrix.

Rank			$\mathbf{p_{ss}(q\|J)}$		n			Steady-state
**AUC**	**JIF**	**Journal abbreviation**	$\overline{\mathbf{q}}$	σ	$\overline{\mathbf{n}}$	**Q2**	**JIF**	**period**
1	1	J FINANC	1.44	0.40	41.4	24	3.257	1984–2000
2	2	ACCOUNT REV	1.04	0.37	13.5	8	2.185	1987–2000
3	3	J BANK FINANC	0.75	0.42	7.7	4	0.769	1979–2000

BUSINESS

ISI Category Description This category covers resources concerned with all aspects of business and the business world. These may include marketing and advertising, forecasting, planning, administration, organizational studies, compensation, strategy, retailing, consumer research, and management. Also covered are resources relating to business history and business ethics.

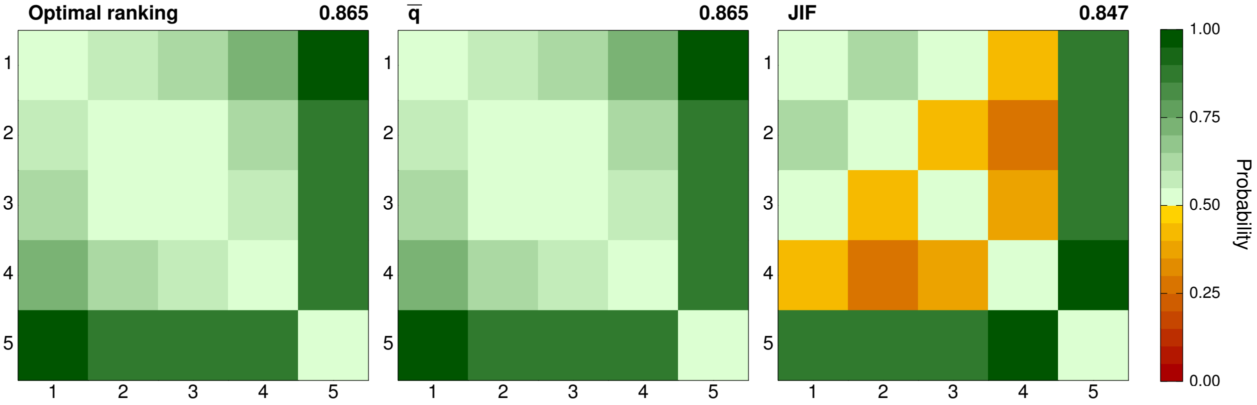

The color of each cell represents the probability of picking a paper with more citations from the higher-ranked journal. Green cells indicate adequate ranking, whereas red cells indicate that the ranking is inadequate (the probability is less than $\frac{1}{2}$). The value of M, the multi-class AUC statistic for each ranking scheme, is above each matrix.

**Rank**			$\mathbf{p_{ss}(q\|J)}$		**n**			**Steady-state**
**AUC**	**JIF**	**Journal abbreviation**	$\overline{\mathbf{q}}$	σ	$\overline{\mathbf{n}}$	**Q2**	**JIF**	**period**
1	4	J BUS ETHICS	0.84	0.34	9.1	5	0.597	1986–1996
2	1	HARVARD BUS REV	0.79	0.60	18.3	4	1.505	1987–1997
3	3	J BUS RES	0.68	0.46	6.3	3	0.815	1972–2001
4	2	LONG RANGE PLANN	0.52	0.40	4.0	2	0.982	1989–2001
5	5	FORTUNE	-0.53	0.46	0.2	0	0.201	1965–2003

CARDIAC & CARDIOVASCULAR SYSTEMS

ISI Category Description Cardiac & Cardiovascular Systems covers resources dealing with the diagnosis and treatment of heart disease. Coverage focuses on cardiac disease prevention, pharmacology, surgery, transplantation, and research. This category also includes cardiac testing, pacemakers, and medical devices. Resources focusing on circulation, hypertension, arterial disease, and stroke are placed in the PERIPHERAL VASCULAR DISEASE category.

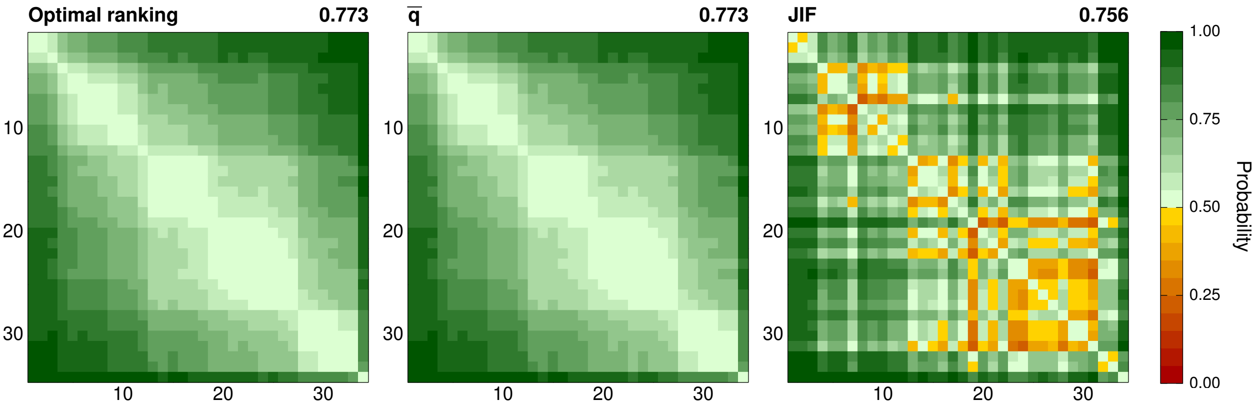

The color of each cell represents the probability of picking a paper with more citations from the higher-ranked journal. Green cells indicate adequate ranking, whereas red cells indicate that the ranking is inadequate (the probability is less than $\frac{1}{2}$). The value of M, the multi-class AUC statistic for each ranking scheme, is above each matrix.

Rank			$\mathbf{p_{ss}(q \mid J)}$		n			Steady-state
**AUC**	**JIF**	**Journal abbreviation**	$\overline{\mathbf{q}}$	σ	$\overline{\mathbf{n}}$	**Q2**	**JIF**	**period**
1	2	CIRC RES	1.72	0.46	75.8	47	9.854	1962–1994
2	1	CIRCULATION	1.71	0.46	75.4	45	10.940	1969–1997
3	3	J AM COLL CARDIOL	1.58	0.40	52.5	34	9.701	1986–1997
4	8	J THORAC CARDIOV SUR	1.42	0.39	34.0	23	3.560	1976–1995
5	10	AM J CARDIOL	1.33	0.41	30.0	18	3.015	1987–1995
6	5	CARDIOVASC RES	1.28	0.37	26.9	16	5.826	1992–1995
7	6	J MOL CELL CARDIOL	1.26	0.36	21.9	15	4.859	1991–2001
8	9	AM HEART J	1.24	0.36	22.9	15	3.514	1989–1995
9	12	ANN THORAC SURG	1.21	0.42	22.1	14	2.342	1973–1995
10	11	J HEART LUNG TRANSPL	1.15	0.37	18.6	12	2.830	1990–1998
11	4	EUR HEART J	1.14	0.36	18.2	11	7.286	1995–1998
12	17	J CARDIOVASC PHARM	1.05	0.34	14.0	9	1.625	1993–1997
13	7	BASIC RES CARDIOL	0.96	0.40	12.1	7	3.798	1973–2001
14	22	PACE	0.96	0.41	12.3	7	1.095	1978–1995
15	14	EUR J CARDIO-THORAC	0.96	0.38	11.0	7	2.106	1989–2000
16	31	J CARDIAC SURG	0.95	0.39	11.6	7	0.709	1990–1996
17	15	RESP MED	0.92	0.39	9.7	6	2.086	1995–2001
18	20	CARDIOVASC INTER RAD	0.89	0.39	10.0	6	1.149	1979–2000
19	18	CARDIOVASC DRUG THER	0.84	0.40	9.4	5	1.396	1988–1998
20	13	INT J CARDIOL	0.80	0.37	7.3	5	2.234	1991–2002
21	29	PEDIATR CARDIOL	0.79	0.40	7.5	5	0.826	1978–2000
22	30	THORAC CARDIOV SURG	0.79	0.39	7.1	4	0.814	1978–2000
23	16	CARDIOLOGY	0.77	0.40	7.5	4	1.795	1972–2001

table continues on next page . . .

. . . table continued from previous page

Rank			$p_{ss}(q\|J)$		n			Steady-state
**AUC**	**JIF**	**Journal abbreviation**	$\overline{q}$	σ	$\overline{n}$	**Q2**	**JIF**	**period**
24	27	HEART LUNG	0.79	0.47	7.9	4	0.955	1986–2001
25	26	CLIN CARDIOL	0.73	0.41	6.8	4	0.989	1980–2001
26	25	J CARDIOVASC SURG	0.72	0.41	6.7	4	1.020	1960–1997
27	21	CAN J CARDIOL	0.69	0.43	6.8	3	1.134	1986–1999
28	28	J ELECTROCARDIOL	0.54	0.47	4.7	2	0.912	1986–2000
29	23	JPN HEART J	0.51	0.45	4.7	2	1.076	1972–1997
30	24	ECHOCARDIOGR-J CARD	0.45	0.41	3.8	2	1.050	1989–2003
31	33	ARCH MAL COEUR VAISS	0.43	0.39	3.0	2	0.519	1979–1997
32	19	Z KARDIOL	0.40	0.38	2.8	2	1.157	1997–2004
33	32	TEX HEART I J	0.37	0.42	2.8	1	0.554	1992–2005
34	34	KARDIOLOGIYA	-0.39	0.39	0.2	0	0.145	1984–2005

CELL BIOLOGY

ISI Category Description Cell Biology includes resources on all aspects of the structure and function of eukaryotic cells. The principle characteristic of resources in this category is an emphasis on the integration at the cellular level of biochemical, molecular, genetic, physiological, and pathological information. This category considers material on specific tissues, differentiated as well as embryonic.

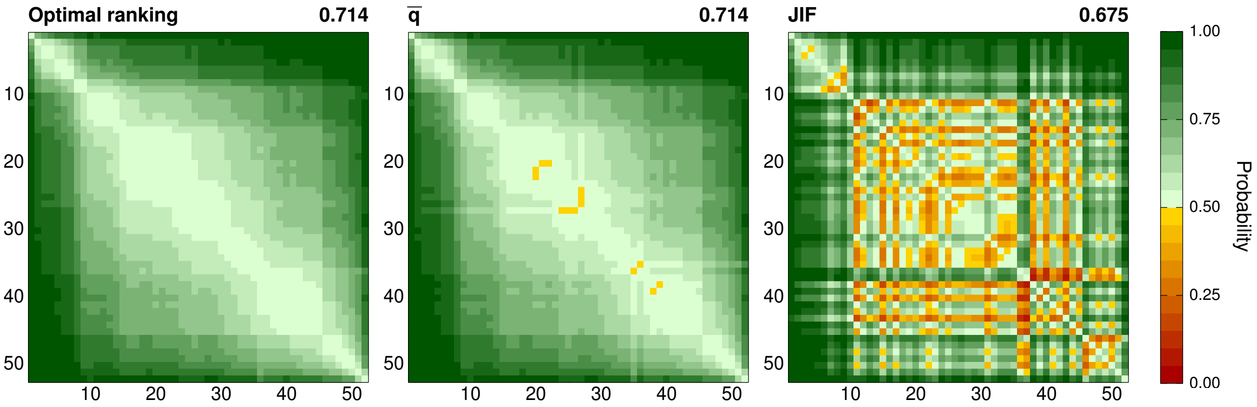

The color of each cell represents the probability of picking a paper with more citations from the higher-ranked journal. Green cells indicate adequate ranking, whereas red cells indicate that the ranking is inadequate (the probability is less than $\frac{1}{2}$). The value of M, the multi-class AUC statistic for each ranking scheme, is above each matrix.

Rank			$\mathbf{p_{ss}(q\|J)}$		n			Steady-state
**AUC**	**JIF**	**Journal abbreviation**	$\overline{\mathbf{q}}$	σ	$\overline{\mathbf{n}}$	**Q2**	**JIF**	**period**
1	1	CELL	2.35	0.30	306.3	216	29.194	1994–1997
2	2	GENE DEV	2.04	0.30	143.5	106	15.050	1988–1996
3	4	EMBO J	1.97	0.33	119.7	85	10.086	1986–1993
4	3	J CELL BIOL	1.95	0.31	111.7	84	10.152	1990–1995
5	5	PLANT CELL	1.87	0.34	92.4	72	9.868	1988–1995
6	9	MOL BIOL CELL	1.80	0.33	90.6	66	6.562	1991–1992
7	6	MOL CELL BIOL	1.80	0.33	82.7	57	6.773	1986–1995
8	8	ONCOGENE	1.66	0.36	60.4	41	6.582	1992–1995
9	7	FASEB J	1.59	0.47	62.0	36	6.721	1986–1998
10	10	J CELL SCI	1.53	0.46	41.3	30	6.427	1991–1997
11	43	J NEUROCYTOL	1.49	0.37	43.7	27	1.695	1971–1990
12	13	AM J RESP CELL MOL	1.48	0.33	38.3	26	4.593	1988–1998
13	38	J MEMBRANE BIOL	1.47	0.38	41.2	27	2.112	1973–1988
14	40	DNA CELL BIOL	1.44	0.36	45.5	25	1.905	1989–1991
15	20	J CELL PHYSIOL	1.39	0.41	35.4	21	3.638	1965–1993
16	18	EXP CELL RES	1.35	0.38	33.9	19	3.777	1955–1996
17	24	FEBS LETT	1.35	0.38	31.4	19	3.372	1983–1995
18	14	J LEUKOCYTE BIOL	1.34	0.34	27.8	18	4.572	1987–1999
19	27	CELL MOTIL CYTOSKEL	1.34	0.33	26.3	19	3.089	1979–1997
20	16	CELL CALCIUM	1.33	0.37	32.0	18	4.118	1979–1994
21	26	HISTOPATHOLOGY	1.33	0.34	28.8	18	3.216	1976–1994
22	34	MOL REPROD DEV	1.34	0.37	26.9	18	2.379	1989–1995
23	33	J HISTOCHEM CYTOCHEM	1.31	0.35	27.5	17	2.449	1987–1997

table continues on next page . . .

. . . table continued from previous page

Rank			$p_{ss}(q\|J)$		n			Steady-state
AUC	JIF	Journal abbreviation	$\overline{q}$	σ	$\overline{n}$	Q2	JIF	period
24	35	CYTOKINE	1.25	0.36	26.1	16	2.355	1990–1994
25	28	EUR J CELL BIOL	1.29	0.39	25.3	16	3.039	1979–1994
26	19	DIFFERENTIATION	1.26	0.39	26.0	15	3.745	1973–1999
27	12	J MOL CELL CARDIOL	1.26	0.36	21.9	15	4.859	1991–2001
28	29	MOL CELL ENDOCRINOL	1.25	0.33	21.4	15	2.918	1982–1997
29	30	CELL TISSUE RES	1.25	0.34	22.6	15	2.580	1986–1992
30	45	PROTOPLASMA	1.22	0.35	19.8	14	1.333	1971–1992
31	21	J STRUCT BIOL	1.19	0.36	23.0	13	3.496	1989–2001
32	25	PLANT CELL PHYSIOL	1.18	0.36	18.7	13	3.324	1958–1998
33	42	CELL IMMUNOL	1.15	0.38	18.9	12	1.709	1979–2000
34	32	TISSUE ANTIGENS	1.14	0.40	20.2	11	2.462	1971–1999
35	39	MOL CELL PROBE	1.11	0.35	17.5	10	2.016	1989–1994
36	23	J CELL BIOCHEM	1.11	0.55	19.0	11	3.409	1994–1999
37	50	ACTA CYTOL	1.06	0.38	14.6	9	0.793	1961–1995
38	17	MECH AGEING DEV	1.04	0.35	14.0	9	3.846	1989–2002
39	48	ANAL QUANT CYTOL	1.04	0.37	13.5	9	0.989	1978–1993
40	11	CELL SIGNAL	1.03	0.31	11.6	8	4.887	1989–2002
41	41	MOL CELL BIOCHEM	1.02	0.39	14.4	8	1.862	1973–1999
42	22	BIOCHEM CELL BIOL	1.00	0.36	13.1	8	3.483	1985–2000
43	15	BIOL CELL	0.98	0.37	11.7	8	4.303	1985–2000
44	44	DEV GROWTH DIFFER	0.98	0.39	12.0	7	1.545	1968–2000
45	31	IMMUNOL CELL BIOL	0.97	0.40	11.8	7	2.482	1986–2002
46	47	TISSUE CELL	0.87	0.33	8.6	6	1.094	1988–1999
47	49	CELL MOL BIOL	0.87	0.36	8.5	5	0.959	1990–1995
48	36	PROSTAG LEUKOTR ESS	0.80	0.34	7.1	5	2.261	2000–2003
49	51	ACTA HISTOCHEM CYTOC	0.74	0.41	7.6	4	0.429	1973–1990
50	37	HISTOL HISTOPATHOL	0.70	0.37	5.6	3	2.182	1985–2001
51	46	ACTA HISTOCHEM	0.53	0.50	4.6	2	1.167	1960–2003
52	52	BIOL MEMBRANY	0.23	0.41	1.7	1	0.318	1990–2001

CHEMISTRY, ANALYTICAL

ISI Category Description Chemistry, Analytical covers resources on the techniques that yield any type of information about chemical systems. Topics include chromatography, thermal analysis, chemometrics, separation techniques, pyrolysis, and electroanalytical and radioanalytical chemistry. Some spectroscopy resources may be included in this category when focusing on analytical techniques and applications in chemistry.

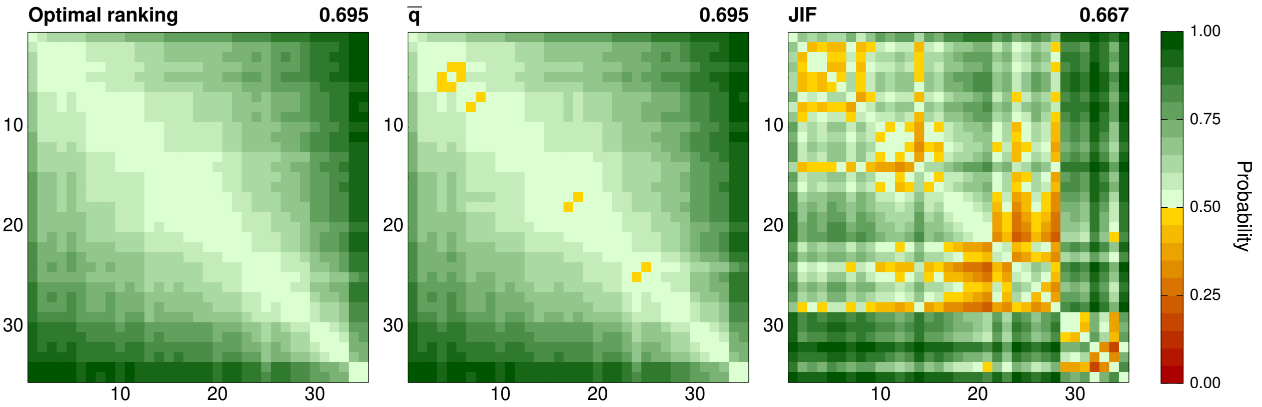

The color of each cell represents the probability of picking a paper with more citations from the higher-ranked journal. Green cells indicate adequate ranking, whereas red cells indicate that the ranking is inadequate (the probability is less than $\frac{1}{2}$). The value of M, the multi-class AUC statistic for each ranking scheme, is above each matrix.

Rank			$p_{ss}(q\|J)$		n			Steady-state
AUC	JIF	Journal abbreviation	$\overline{q}$	σ	$\overline{n}$	Q2	JIF	period
1	1	ANAL CHEM	1.44	0.40	39.0	24	5.646	1970–1998
2	8	ANAL BIOCHEM	1.28	0.47	67.8	15	2.948	1959–1989
3	6	J AM SOC MASS SPECTR	1.26	0.37	25.5	15	3.307	1989–2000
4	14	J ELECTROANAL CHEM	1.25	0.40	25.8	15	2.339	1963–1991
5	5	J ANAL ATOM SPECTROM	1.25	0.33	19.6	14	3.630	1998–2000
6	3	BIOSENS BIOELECTRON	1.25	0.41	21.1	15	4.132	1990–2000
7	28	J CHROMATOGR SCI	1.16	0.38	20.8	12	0.880	1968–1995
8	4	ELECTROPHORESIS	1.16	0.40	19.8	12	4.101	1994–1996
9	9	ANAL CHIM ACTA	1.16	0.36	18.4	12	2.894	1964–1999
10	2	TRAC-TREND ANAL CHEM	1.13	0.42	18.0	11	5.068	1990–2003
11	24	J ANAL TOXICOL	1.12	0.34	15.9	11	1.242	1977–1996
12	7	ANALYST	1.10	0.36	15.4	10	3.198	1985–1996
13	11	RAPID COMMUN MASS SP	1.05	0.44	18.4	9	2.680	1989–1997
14	16	CHIRALITY	1.06	0.34	13.7	9	2.165	1990–1997
15	13	ELECTROANAL	1.04	0.37	12.4	9	2.444	1989–1999
16	10	TALANTA	1.02	0.34	12.4	9	2.810	1985–2001
17	25	CHROMATOGRAPHIA	1.02	0.35	13.8	8	1.171	1974–1998
18	15	SENSOR ACTUAT B-CHEM	1.02	0.38	12.2	8	2.331	1995–2001
19	22	J ANAL APPL PYROL	1.00	0.37	12.8	8	1.412	1979–1998
20	12	CHEMOMETR INTELL LAB	0.96	0.42	14.8	8	2.450	1986–2002
21	27	INT J ENVIRON AN CH	0.95	0.37	11.7	7	0.917	1976–1996
22	17	J PHARMACEUT BIOMED	0.94	0.34	9.6	7	2.032	1984–2000
23	18	BIOMED CHROMATOGR	0.90	0.39	8.3	6	1.611	1988–1999

table continues on next page . . .

. . . table continued from previous page

Rank			$\mathbf{p_{ss}(q\|J)}$		n			Steady-state
**AUC**	**JIF**	**Journal abbreviation**	$\overline{\mathbf{q}}$	σ	$\overline{\mathbf{n}}$	**Q2**	**JIF**	**period**
24	26	ANAL LETT	0.85	0.36	8.6	5	0.986	1977–1998
25	23	J AOAC INT	0.87	0.56	11.0	5	1.352	1991–1996
26	19	ANAL SCI	0.81	0.36	7.2	5	1.589	1987–1998
27	20	MICROCHEM J	0.79	0.37	6.5	4	1.558	1995–2001
28	34	COMMUN SOIL SCI PLAN	0.72	0.41	8.9	4	0.302	1973–1995
29	21	THERMOCHIM ACTA	0.70	0.38	6.0	4	1.417	1985–2001
30	31	ANN CHIM-ROME	0.57	0.38	4.4	3	0.516	1968–1998
31	29	J LABELLED COMPD RAD	0.52	0.38	4.0	2	0.746	1978–2003
32	30	CHEM ANAL-WARSAW	0.47	0.35	2.9	2	0.560	1976–2000
33	33	BUNSEKI KAGAKU	0.40	0.38	2.5	2	0.307	1988–2000
34	32	J ANAL CHEM+	0.04	0.40	0.9	0	0.444	1994–2003
35	35	AM LAB	-0.02	0.44	1.0	0	0.220	2000–2003

CHEMISTRY, APPLIED

ISI Category Description Chemistry, Applied covers resources that report on the application of basic chemical sciences to other sciences, engineering, and industry. Topics include chemical engineering (catalysis, fuel processing, microencapsulation, and functional polymers); food science and technology (cereals, hydrocolloids, and food additives); medicinal chemistry (pharmacology); dyes and pigments; coatings technology; and cosmetics.

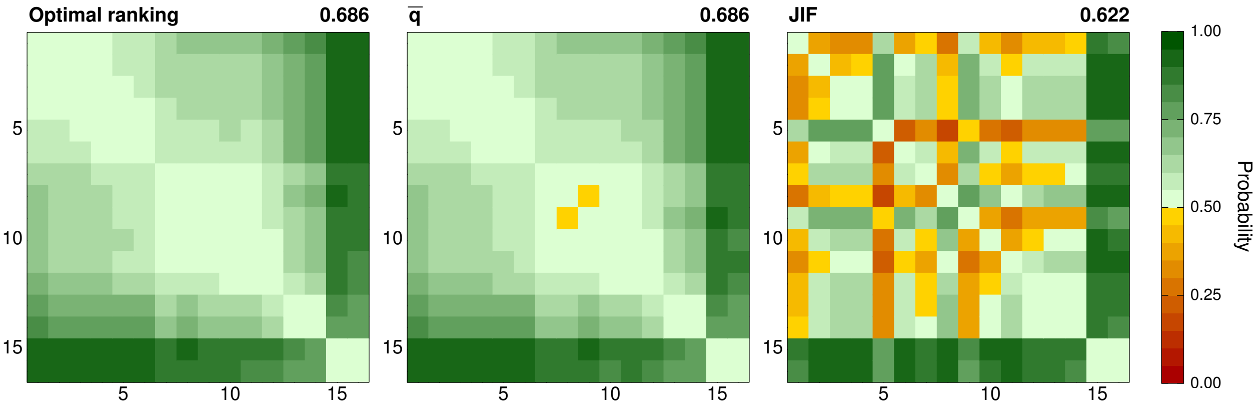

The color of each cell represents the probability of picking a paper with more citations from the higher-ranked journal. Green cells indicate adequate ranking, whereas red cells indicate that the ranking is inadequate (the probability is less than $\frac{1}{2}$). The value of M, the multi-class AUC statistic for each ranking scheme, is above each matrix.

Rank			$p_{ss}(q\|J)$		n			Steady-state
AUC	JIF	Journal abbreviation	$\overline{q}$	σ	$\overline{n}$	Q2	JIF	period
1	8	CARBOHYD RES	1.23	0.36	23.6	14	1.703	1967–1989
2	3	J AGR FOOD CHEM	1.20	0.38	21.2	13	2.322	1966–1994
3	4	CATAL TODAY	1.19	0.39	20.5	13	2.148	1992–2000
4	11	CEREAL CHEM	1.17	0.38	19.3	12	1.254	1954–1995
5	2	J NAT PROD	1.12	0.31	15.2	11	2.418	1978–1996
6	6	CARBOHYD POLYM	1.11	0.34	14.6	10	1.784	1993–2000
7	12	APPL ORGANOMET CHEM	1.02	0.39	12.4	8	1.233	1989–1998
8	10	BIOSCI BIOTECH BIOCH	1.00	0.35	12.1	8	1.256	1991–1994
9	13	J SCI FOOD AGR	1.00	0.40	12.8	8	1.026	1986–1996
10	7	FOOD ADDIT CONTAM	0.97	0.37	11.4	8	1.780	1985–2001
11	14	J AM OIL CHEM SOC	0.98	0.46	15.0	7	0.910	1955–1991
12	1	FOOD CHEM	0.91	0.37	9.9	6	2.433	1976–2002
13	9	FUEL PROCESS TECHNOL	0.77	0.41	7.5	4	1.323	1976–2001
14	5	DYES PIGMENTS	0.69	0.36	5.9	3	1.909	1980–2003
15	15	AGROCHIMICA	0.13	0.43	1.5	0	0.231	1965–2003
16	16	CHEM IND-LONDON	0.11	0.61	2.8	0	0.225	1954–2002

CHEMISTRY, INORGANIC & NUCLEAR

ISI Category Description Chemistry, Inorganic & Nuclear includes resources on both inorganic and nuclear chemistry. Chemistry, Inorganic covers resources that are concerned with non-carbon elements and the preparation, properties, and reactions of their compounds. It also includes resources on the study of certain simple carbon compounds, including the oxides, carbon disulfide, the halides, hydrogen cyanide, and salts, such as the cyanides, cyanates, carbonates, and hydrogencarbonates. Resources on coordination chemistry and organo-metallic compounds (those containing a carbon-metal bond) are also covered in this category. Chemistry, Nuclear includes resources on the study of the atomic nucleus, including fission and fusion reactions and their products. This category also covers radiochemistry resources focusing on such topics as the preparation of radioactive compounds, the separation of isotopes by chemical reactions, the use of radioactive labels in studies of mechanisms, and experiments on the chemical reactions and compounds of transuranic elements.

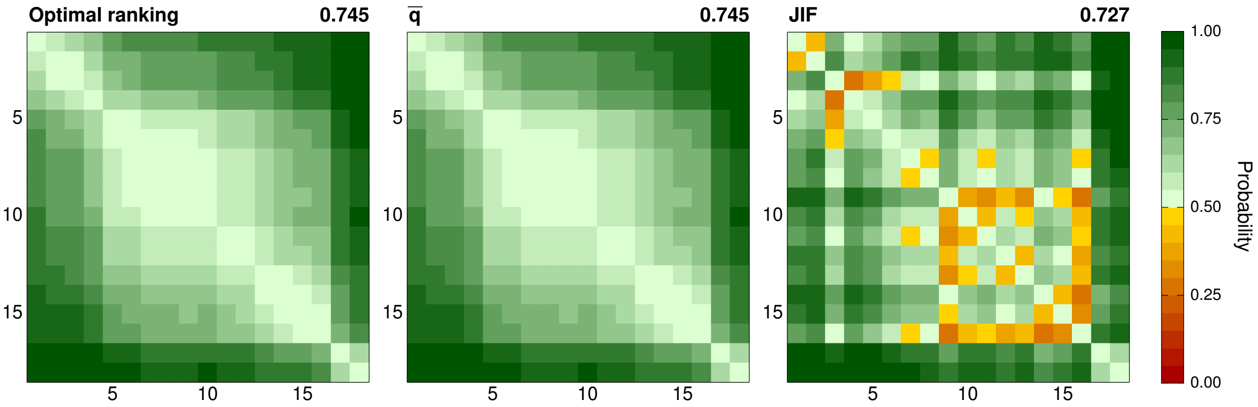

The color of each cell represents the probability of picking a paper with more citations from the higher-ranked journal. Green cells indicate adequate ranking, whereas red cells indicate that the ranking is inadequate (the probability is less than $\frac{1}{2}$). The value of M, the multi-class AUC statistic for each ranking scheme, is above each matrix.

Rank			$p_{ss}(q\|J)$		n			Steady-state
**AUC**	**JIF**	**Journal abbreviation**	$\bar{q}$	σ	$\bar{n}$	**Q2**	**JIF**	**period**
1	2	ORGANOMETALLICS	1.52	0.31	39.2	29	3.632	1981–1984
2	1	INORG CHEM	1.41	0.32	30.2	23	3.911	1982–1994
3	4	TETRAHEDRON-ASYMMETR	1.36	0.27	25.9	21	2.468	1989–1990
4	5	J ORGANOMET CHEM	1.27	0.32	22.3	16	2.332	1970–1981
5	6	J SOLID STATE CHEM	1.11	0.37	17.7	11	2.107	1980–1992
6	3	J INORG BIOCHEM	1.08	0.35	13.8	10	2.654	1984–2000
7	8	INORG CHIM ACTA	1.03	0.35	12.2	9	1.674	1979–1996
8	16	Z NATURFORSCH B	1.02	0.36	12.3	9	0.825	1971–1988
9	11	APPL ORGANOMET CHEM	1.02	0.39	12.4	8	1.233	1989–1998
10	7	POLYHEDRON	1.00	0.33	11.2	8	1.843	1990–1997
11	13	J COORD CHEM	0.92	0.35	9.4	6	0.978	1975–1994
12	10	Z ANORG ALLG CHEM	0.90	0.34	8.7	6	1.241	1986–1996
13	12	RADIOCHIM ACTA	0.83	0.38	8.4	5	1.068	1965–2000
14	15	TRANSIT METAL CHEM	0.76	0.35	6.5	4	0.918	1986–2001
15	9	J FLUORINE CHEM	0.68	0.32	5.7	3	1.515	2000–2002
16	14	APPL RADIAT ISOTOPES	0.65	0.40	5.0	3	0.924	1993–2000
17	17	PHOSPHORUS SULFUR	0.19	0.46	1.6	1	0.520	1998–2002
18	18	J STRUCT CHEM+	-0.20	0.49	0.7	0	0.345	1974–2004

CHEMISTRY, MEDICINAL

ISI Category Description Chemistry, Medicinal includes resources emphasizing the isolation and study of substances with therapeutic potential. Topics of interest are quantitative structure-function relationships, structural characterization and organic syntheses of naturally occurring compounds, and chemical and analytical techniques used in rational drug design. See also the PHARMACOLOGY & PHARMACY category.

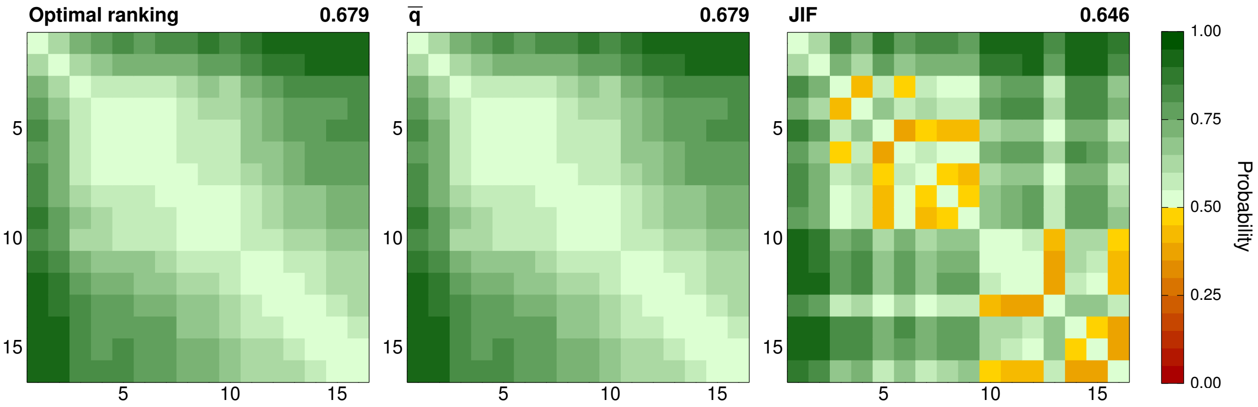

The color of each cell represents the probability of picking a paper with more citations from the higher-ranked journal. Green cells indicate adequate ranking, whereas red cells indicate that the ranking is inadequate (the probability is less than $\frac{1}{2}$). The value of M, the multi-class AUC statistic for each ranking scheme, is above each matrix.

**Rank**			$\mathbf{p_{ss}(q\|J)}$		**n**			**Steady-state**
**AUC**	**JIF**	**Journal abbreviation**	$\bar{\mathbf{q}}$	σ	$\bar{\mathbf{n}}$	**Q2**	**JIF**	**period**
1	1	J MED CHEM	1.42	0.30	31.2	23	5.115	1992–1998
2	2	CHEM RES TOXICOL	1.29	0.30	23.4	16	3.162	1997–1999
3	4	J NAT PROD	1.12	0.31	15.2	11	2.418	1978–1996
4	6	CHIRALITY	1.06	0.34	13.7	9	2.165	1990–1997
5	3	BIOORG MED CHEM LETT	1.03	0.30	11.2	9	2.538	1999–2001
6	9	CHEM PHARM BULL	1.01	0.34	12.5	8	1.262	1961–1990
7	8	J ETHNOPHARMACOL	0.99	0.33	10.8	8	1.625	1978–2001
8	7	PLANTA MED	0.94	0.44	11.0	7	1.746	1977–2000
9	5	EUR J MED CHEM	0.90	0.35	9.7	6	2.187	1978–2001
10	13	DRUG DEVELOP RES	0.89	0.47	12.0	6	0.752	1980–1999
11	16	ARZNEIMITTEL-FORSCH	0.76	0.42	8.7	4	0.596	1955–1996
12	10	PHYTOTHER RES	0.73	0.34	6.0	4	1.144	1998–2001
13	11	ARCH PHARM	0.66	0.32	4.8	3	1.076	1968–2001
14	12	DRUG DEV IND PHARM	0.61	0.34	4.1	3	0.821	1999–2002
15	15	PHARMAZIE	0.56	0.37	4.0	2	0.606	1974–2001
16	14	J LABELLED COMPD RAD	0.52	0.38	4.0	2	0.746	1978–2003

CHEMISTRY, MULTIDISCIPLINARY

ISI Category Description Chemistry, Multidisciplinary includes resources having a general or interdisciplinary approach to the chemical sciences. Special topic chemistry resources that have relevance to many areas of chemistry are also included in this category. Resources having a primary focus on analytical, inorganic and nuclear, organic, physical, or polymer chemistry are placed in their own categories.

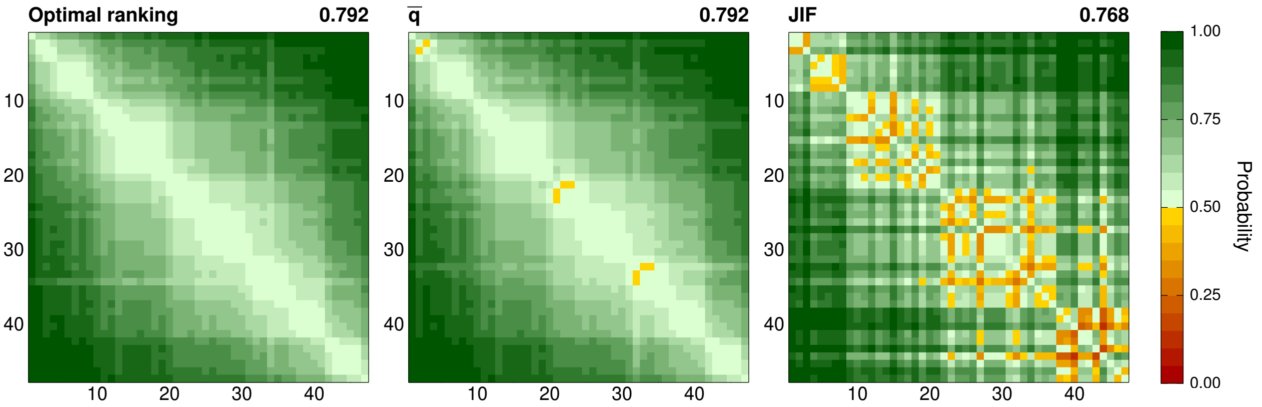

The color of each cell represents the probability of picking a paper with more citations from the higher-ranked journal. Green cells indicate adequate ranking, whereas red cells indicate that the ranking is inadequate (the probability is less than $\frac{1}{2}$). The value of M, the multi-class AUC statistic for each ranking scheme, is above each matrix.

Rank			$p_{ss}(q\|J)$		n			Steady-state
AUC	JIF	Journal abbreviation	$\overline{q}$	σ	$\overline{n}$	Q2	JIF	period
1	3	J AM CHEM SOC	1.62	0.34	58.4	37	7.696	1967–1994
2	1	ANGEW CHEM INT EDIT	1.47	0.35	37.6	25	10.232	1992–1999
3	2	ADV MATER	1.47	0.43	39.9	25	7.896	1995–2001
4	8	MAR CHEM	1.38	0.36	31.1	20	2.663	1976–1996
5	7	CHEM RES TOXICOL	1.29	0.30	23.4	16	3.162	1997–1999
6	5	J CONTROL RELEASE	1.28	0.35	22.8	16	4.012	1997–2000
7	4	J COMPUT CHEM	1.26	0.44	47.1	15	4.893	1979–1999
8	6	BIOCONJUGATE CHEM	1.25	0.34	23.2	15	3.823	1990–2000
9	15	HELV CHIM ACTA	1.20	0.38	22.5	13	1.550	1955–1996
10	12	CHEM LETT	1.09	0.36	15.5	10	1.734	1978–1991
11	9	NEW J CHEM	1.04	0.39	14.6	9	2.647	1984–2000
12	18	CHEM PHARM BULL	1.01	0.34	12.5	8	1.262	1961–1990
13	10	PURE APPL CHEM	1.00	0.46	16.0	8	1.920	1988–2000
14	20	SOLVENT EXTR ION EXC	0.96	0.38	11.0	7	1.162	1987–1998
15	16	B CHEM SOC JPN	0.94	0.36	11.0	7	1.505	1990–1998
16	11	AUST J CHEM	0.94	0.34	10.0	7	1.895	1987–1995
17	14	MAGN RESON CHEM	0.93	0.35	9.9	6	1.610	1984–1994
18	13	J CHEM ENG DATA	0.92	0.41	12.0	6	1.642	1964–1998
19	21	CAN J CHEM	0.91	0.36	9.6	6	1.153	1994–1998
20	17	J CHEM TECHNOL BIOT	0.84	0.41	9.4	5	1.276	1978–1999
21	34	ARZNEIMITTEL-FORSCH	0.76	0.42	8.7	4	0.596	1955–1996
22	19	J PHYS CHEM SOLIDS	0.76	0.41	7.5	4	1.164	1996–2000
23	26	HETEROATOM CHEM	0.78	0.40	6.3	4	0.838	1990–1995

table continues on next page . . .

. . . table continued from previous page

Rank			$p_{ss}(q\|J)$		n			Steady-state
**AUC**	**JIF**	**Journal abbreviation**	$\overline{q}$	σ	$\overline{n}$	**Q2**	**JIF**	**period**
24	24	MONATSH CHEM	0.68	0.35	5.5	3	0.920	1985–2000
25	28	CROAT CHEM ACTA	0.68	0.42	6.7	3	0.778	1963–1998
26	22	ARCH PHARM	0.66	0.32	4.8	3	1.076	1968–2001
27	29	MENDELEEV COMMUN	0.65	0.35	4.6	3	0.712	1991–2000
28	30	INDIAN J CHEM A	0.65	0.38	4.9	3	0.631	1976–1995
29	25	COLLECT CZECH CHEM C	0.61	0.36	4.7	3	0.881	1984–2001
30	44	J CHEM RES-S	0.61	0.38	4.5	3	0.210	1985–2000
31	33	PHARMAZIE	0.56	0.37	4.0	2	0.606	1974–2001
32	37	J CHEM EDUC	0.53	0.43	5.4	2	0.439	1968–1999
33	36	POL J CHEM	0.52	0.38	3.6	2	0.491	1977–2002
34	31	CHIMIA	0.54	0.64	7.1	2	0.626	1960–1998
35	23	B KOR CHEM SOC	0.45	0.37	3.0	2	0.950	1980–2003
36	35	J CHIN CHEM SOC-TAIP	0.44	0.39	3.1	2	0.577	1960–2002
37	41	J INDIAN CHEM SOC	0.37	0.39	2.6	1	0.340	1982–2001
38	42	ANN CHIM-SCI MAT	0.35	0.43	2.7	1	0.288	1977–2002
39	27	ACTA CHIM SINICA	0.34	0.42	2.4	1	0.783	1979–2003
40	32	SCI CHINA SER B	0.32	0.44	2.4	1	0.617	1984–2002
41	45	REV ROUM CHIM	0.28	0.41	2.0	1	0.208	1979–2000
42	47	J CHEM SOC PAKISTAN	0.13	0.41	1.3	1	0.152	1984–2002
43	39	PRZEM CHEM	0.03	0.40	0.9	0	0.429	1969–2002
44	38	CHEM LISTY	0.01	0.42	1.0	0	0.431	1987–2004
45	46	AFINIDAD	-0.05	0.46	0.9	0	0.188	1978–2000
46	43	REV CHIM-BUCHAREST	-0.05	0.42	0.8	0	0.287	1976–2002
47	40	CHEM ENG NEWS	-0.50	0.67	0.9	0	0.379	1967–2004

CHEMISTRY, ORGANIC

ISI Category Description Chemistry, Organic includes resources that focus on synthetic and natural organic compounds their synthesis, structure, properties, and reactivity. Research on hydrocarbons, a major area of organic chemistry, is included in this category.

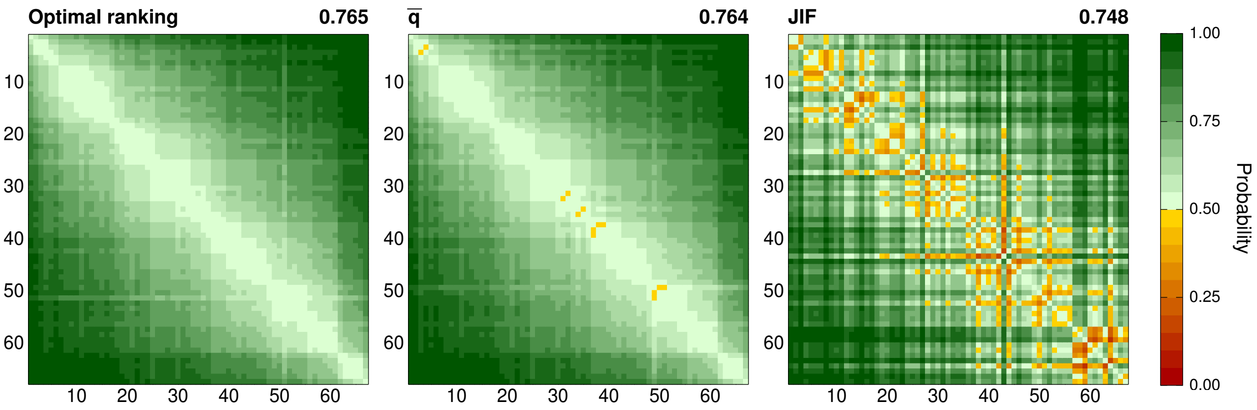

The color of each cell represents the probability of picking a paper with more citations from the higher-ranked journal. Green cells indicate adequate ranking, whereas red cells indicate that the ranking is inadequate (the probability is less than $\frac{1}{2}$). The value of M, the multi-class AUC statistic for each ranking scheme, is above each matrix.

Rank			$p_{ss}(q\|J)$		n			Steady-state
**AUC**	**JIF**	**Journal abbreviation**	$\overline{q}$	σ	$\overline{n}$	**Q2**	**JIF**	**period**
1	3	J AM CHEM SOC	1.62	0.34	58.4	37	7.696	1967–1994
2	8	ORGANOMETALLICS	1.52	0.31	39.2	29	3.632	1981–1984
3	1	ANGEW CHEM INT EDIT	1.47	0.35	37.6	25	10.232	1992–1999
4	2	ADV MATER	1.47	0.43	39.9	25	7.896	1995–2001
5	11	MAR CHEM	1.38	0.36	31.1	20	2.663	1976–1996
6	15	TETRAHEDRON-ASYMMETR	1.36	0.27	25.9	21	2.468	1989–1990
7	9	CHEM RES TOXICOL	1.29	0.30	23.4	16	3.162	1997–1999
8	7	J ORG CHEM	1.29	0.35	25.9	17	3.790	1968–1989
9	5	J CONTROL RELEASE	1.28	0.35	22.8	16	4.012	1997–2000
10	17	J ORGANOMET CHEM	1.27	0.32	22.3	16	2.332	1970–1981
11	4	J COMPUT CHEM	1.26	0.44	47.1	15	4.893	1979–1999
12	6	BIOCONJUGATE CHEM	1.25	0.34	23.2	15	3.823	1990–2000
13	23	CARBOHYD RES	1.23	0.36	23.6	14	1.703	1967–1989
14	10	TETRAHEDRON	1.21	0.35	21.7	13	2.817	1976–1993
15	27	HELV CHIM ACTA	1.20	0.38	22.5	13	1.550	1955–1996
16	14	TETRAHEDRON LETT	1.19	0.33	19.1	13	2.509	1959–1998
17	16	SYNTHESIS-STUTTGART	1.16	0.34	18.3	12	2.333	1971–1995
18	21	CARBOHYD POLYM	1.11	0.34	14.6	10	1.784	1993–2000
19	22	CHEM LETT	1.09	0.36	15.5	10	1.734	1978–1991
20	18	CHIRALITY	1.06	0.34	13.7	9	2.165	1990–1997
21	12	NEW J CHEM	1.04	0.39	14.6	9	2.647	1984–2000
22	13	BIOORG MED CHEM LETT	1.03	0.30	11.2	9	2.538	1999–2001
23	43	Z NATURFORSCH B	1.02	0.36	12.3	9	0.825	1971–1988
24	31	CHEM PHARM BULL	1.01	0.34	12.5	8	1.262	1961–1990

table continues on next page . . .

. . . table continued from previous page

Rank			$p_{ss}(q\|J)$		n			Steady-state
AUC	JIF	Journal abbreviation	$\overline{q}$	σ	$\overline{n}$	Q2	JIF	period
25	19	PURE APPL CHEM	1.00	0.46	16.0	8	1.920	1988–2000
26	33	SOLVENT EXTR ION EXC	0.96	0.38	11.0	7	1.162	1987–1998
27	29	B CHEM SOC JPN	0.94	0.36	11.0	7	1.505	1990–1998
28	20	AUST J CHEM	0.94	0.34	10.0	7	1.895	1987–1995
29	25	MAGN RESON CHEM	0.93	0.35	9.9	6	1.610	1984–1994
30	24	J CHEM ENG DATA	0.92	0.41	12.0	6	1.642	1964–1998
31	35	HETEROCYCLES	0.91	0.32	9.0	6	1.077	1974–1993
32	34	CAN J CHEM	0.91	0.36	9.6	6	1.153	1994–1998
33	46	J HETEROCYCLIC CHEM	0.84	0.33	7.7	5	0.776	1984–1992
34	26	J PHYS ORG CHEM	0.84	0.33	8.4	5	1.593	1995–2000
35	30	J CHEM TECHNOL BIOT	0.84	0.41	9.4	5	1.276	1978–1999
36	37	SYNTHETIC COMMUN	0.82	0.35	7.6	5	1.001	1986–1997
37	52	ARZNEIMITTEL-FORSCH	0.76	0.42	8.7	4	0.596	1955–1996
38	32	J PHYS CHEM SOLIDS	0.76	0.41	7.5	4	1.164	1996–2000
39	41	HETEROATOM CHEM	0.78	0.40	6.3	4	0.838	1990–1995
40	28	J FLUORINE CHEM	0.68	0.32	5.7	3	1.515	2000–2002
41	39	MONATSH CHEM	0.68	0.35	5.5	3	0.920	1985–2000
42	45	CROAT CHEM ACTA	0.68	0.42	6.7	3	0.778	1963–1998
43	36	ARCH PHARM	0.66	0.32	4.8	3	1.076	1968–2001
44	47	MENDELEEV COMMUN	0.65	0.35	4.6	3	0.712	1991–2000
45	48	INDIAN J CHEM A	0.65	0.38	4.9	3	0.631	1976–1995
46	40	COLLECT CZECH CHEM C	0.61	0.36	4.7	3	0.881	1984–2001
47	64	J CHEM RES-S	0.61	0.38	4.5	3	0.210	1985–2000
48	51	PHARMAZIE	0.56	0.37	4.0	2	0.606	1974–2001
49	56	J CHEM EDUC	0.53	0.43	5.4	2	0.439	1968–1999
50	54	POL J CHEM	0.52	0.38	3.6	2	0.491	1977–2002
51	49	CHIMIA	0.54	0.64	7.1	2	0.626	1960–1998
52	61	KHIM GETEROTSIKL+	0.48	0.36	2.9	2	0.313	1988–1996
53	55	INDIAN J CHEM B	0.48	0.40	3.4	2	0.491	1995–1999
54	38	B KOR CHEM SOC	0.45	0.37	3.0	2	0.950	1980–2003
55	53	J CHIN CHEM SOC-TAIP	0.44	0.39	3.1	2	0.577	1960–2002
56	42	J SYN ORG CHEM JPN	0.40	0.52	3.8	1	0.832	1978–2001
57	60	J INDIAN CHEM SOC	0.37	0.39	2.6	1	0.340	1982–2001
58	62	ANN CHIM-SCI MAT	0.35	0.43	2.7	1	0.288	1977–2002
59	44	ACTA CHIM SINICA	0.34	0.42	2.4	1	0.783	1979–2003
60	50	SCI CHINA SER B	0.32	0.44	2.4	1	0.617	1984–2002
61	65	REV ROUM CHIM	0.28	0.41	2.0	1	0.208	1979–2000
62	67	J CHEM SOC PAKISTAN	0.13	0.41	1.3	1	0.152	1984–2002
63	58	PRZEM CHEM	0.03	0.40	0.9	0	0.429	1969–2002
64	57	CHEM LISTY	0.01	0.42	1.0	0	0.431	1987–2004
65	66	AFINIDAD	-0.05	0.46	0.9	0	0.188	1978–2000
66	63	REV CHIM-BUCHAREST	-0.05	0.42	0.8	0	0.287	1976–2002
67	59	CHEM ENG NEWS	-0.50	0.67	0.9	0	0.379	1967–2004

CHEMISTRY, PHYSICAL

ISI Category Description Chemistry, Physical includes resources on photochemistry, solid state chemistry, kinetics, catalysis, quantum chemistry, surface chemistry, electrochemistry, chemical thermodynamics, thermophysics, colloids, fullerenes, and zeolites.

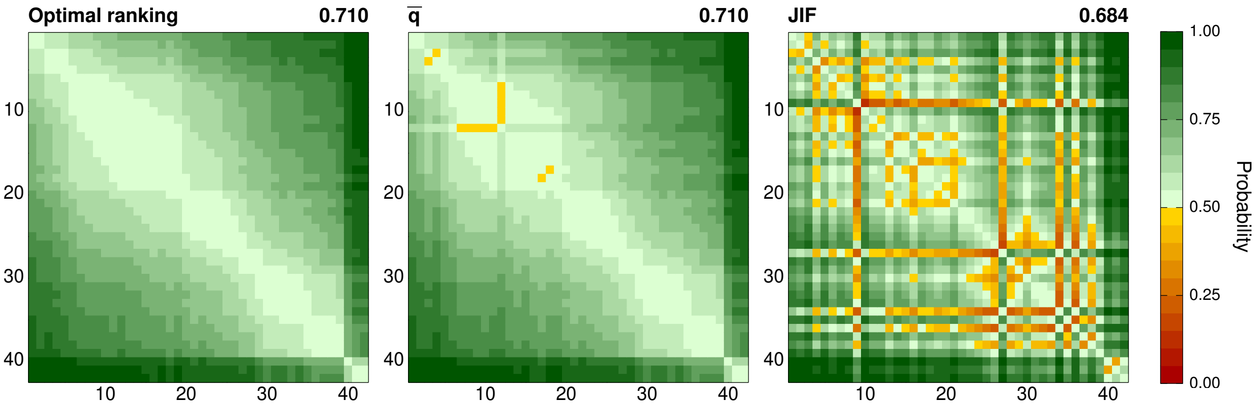

The color of each cell represents the probability of picking a paper with more citations from the higher-ranked journal. Green cells indicate adequate ranking, whereas red cells indicate that the ranking is inadequate (the probability is less than $\frac{1}{2}$). The value of M, the multi-class AUC statistic for each ranking scheme, is above each matrix.

Rank			$p_{ss}(q\|J)$		n			Steady-state
**AUC**	**JIF**	**Journal abbreviation**	$\overline{q}$	σ	$\overline{n}$	**Q2**	**JIF**	**period**
1	3	J CATAL	1.47	0.39	41.0	26	4.533	1961–1995
2	1	ADV MATER	1.47	0.43	39.9	25	7.896	1995–2001
3	10	TETRAHEDRON-ASYMMETR	1.36	0.27	25.9	21	2.468	1989–1990
4	5	LANGMUIR	1.36	0.38	31.6	20	3.902	1984–1995
5	2	CHEM MATER	1.33	0.38	29.0	18	5.104	1989–2000
6	7	J AM SOC MASS SPECTR	1.26	0.37	25.5	15	3.307	1989–2000
7	27	SURF INTERFACE ANAL	1.17	0.38	30.9	14	1.427	1980–1984
8	12	J COLLOID INTERF SCI	1.24	0.42	24.4	14	2.233	1986–1992
9	11	CHEM PHYS LETT	1.21	0.39	23.1	14	2.462	1972–1993
10	34	J SOLUTION CHEM	1.19	0.38	21.9	13	1.026	1974–1985
11	14	CATAL TODAY	1.19	0.39	20.5	13	2.148	1992–2000
12	8	APPL CATAL A-GEN	1.18	0.34	16.8	13	2.630	1997–1999
13	21	CATAL LETT	1.16	0.39	19.0	12	1.772	1988–1997
14	17	CHEM PHYS	1.15	0.38	17.7	12	1.984	1986–1995
15	6	CARBON	1.15	0.41	19.3	11	3.884	1963–1998
16	36	INT J CHEM KINET	1.12	0.41	18.9	11	0.870	1980–1992
17	15	J SOLID STATE CHEM	1.11	0.37	17.7	11	2.107	1980–1992
18	18	SURF SCI	1.12	0.47	18.5	11	1.880	1989–1995
19	4	J MATER CHEM	1.11	0.36	15.9	10	4.287	1990–2001
20	20	BIOPHYS CHEM	1.04	0.37	14.1	9	1.784	1982–1999
21	13	SOLID STATE IONICS	1.02	0.42	13.3	8	2.190	1998–2000
22	19	J CHEM THERMODYN	1.00	0.35	12.1	8	1.842	1983–1994
23	22	FLUID PHASE EQUILIBR	0.99	0.39	12.3	7	1.680	1987–1995
24	30	COLLOID POLYM SCI	0.97	0.41	13.3	7	1.249	1973–1999

table continues on next page . . .

...table continued from previous page

**Rank**			$\mathbf{p_{ss}(q\|J)}$		$\mathbf{n}$			**Steady-state**
**AUC**	**JIF**	**Journal abbreviation**	$\overline{\mathbf{q}}$	σ	$\overline{\mathbf{n}}$	**Q2**	**JIF**	**period**
25	16	J PHOTOCH PHOTOBIO A	0.93	0.37	10.7	7	2.098	1986–2002
26	23	MAGN RESON CHEM	0.93	0.35	9.9	6	1.610	1984–1994
27	38	INT J THERMOPHYS	0.88	0.39	9.2	6	0.793	1985–1997
28	24	J PHYS ORG CHEM	0.84	0.33	8.4	5	1.593	1995–2000
29	29	J ALLOY COMPD	0.80	0.42	8.2	5	1.250	1990–1997
30	31	INT J QUANTUM CHEM	0.77	0.52	8.9	4	1.182	1992–1998
31	25	J MOL STRUCT	0.75	0.41	6.7	4	1.495	1991–2000
32	32	Z PHYS CHEM	0.71	0.38	6.3	4	1.132	1990–2000
33	9	INT J HYDROGEN ENERG	0.70	0.42	6.7	4	2.612	1975–2004
34	28	THERMOCHIM ACTA	0.70	0.38	6.0	4	1.417	1985–2001
35	33	J MOL LIQ	0.65	0.42	5.6	3	1.106	1982–2002
36	26	APPL SURF SCI	0.62	0.49	5.7	3	1.436	1996–1999
37	37	RADIAT PHYS CHEM	0.60	0.44	5.1	2	0.868	1992–2000
38	35	Z NATURFORSCH A	0.58	0.43	4.7	2	0.904	1991–2001
39	39	REACT KINET CATAL L	0.54	0.38	3.8	2	0.514	1982–2000
40	41	HIGH ENERG CHEM+	0.16	0.45	1.3	0	0.418	1993–2002
41	40	KINET CATAL+	-0.12	0.48	0.8	0	0.482	1975–2004
42	42	J STRUCT CHEM+	-0.20	0.49	0.7	0	0.345	1974–2004

CLINICAL NEUROLOGY

ISI Category Description Clinical Neurology covers resources on all areas of clinical research and medical practice in neurology. The focus is on traditional neurological illnesses and diseases such as dementia, stroke, epilepsy, headache, multiple sclerosis, and movement disorders that have clinical and socio-economic importance. This category also includes resources on medical specialties such as pediatric neurology, neurosurgery, neuroradiology, pain management, and neuropsychiatry that affect neurological diagnosis and treatment.

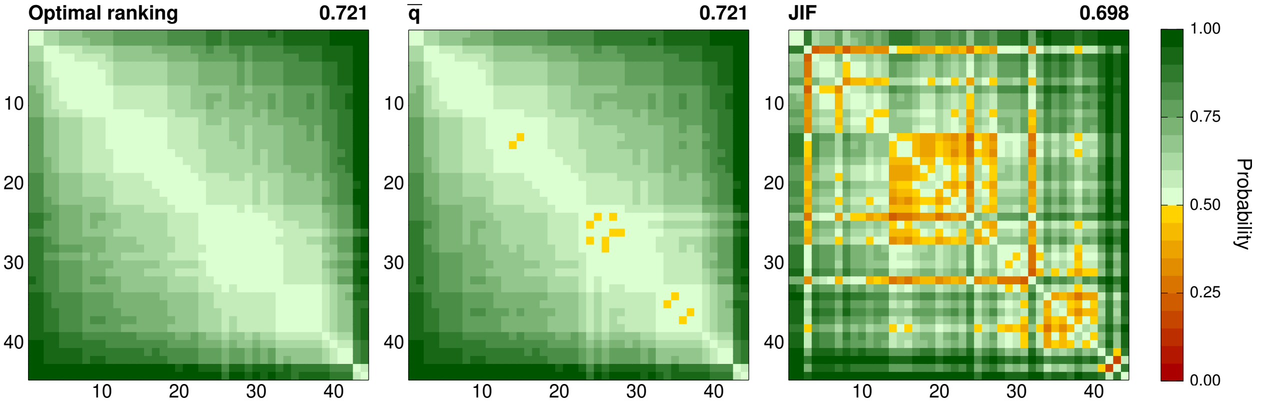

The color of each cell represents the probability of picking a paper with more citations from the higher-ranked journal. Green cells indicate adequate ranking, whereas red cells indicate that the ranking is inadequate (the probability is less than $\frac{1}{2}$). The value of M, the multi-class AUC statistic for each ranking scheme, is above each matrix.

Rank			$p_{ss}(q\|J)$		n			Steady-state
**AUC**	**JIF**	**Journal abbreviation**	$\overline{q}$	σ	$\overline{n}$	**Q2**	**JIF**	**period**
1	1	ANN NEUROL	1.77	0.38	83.5	55	8.051	1984–1993
2	2	BRAIN	1.78	0.40	82.8	55	7.617	1954–1996
3	4	NEUROLOGY	1.63	0.40	61.0	38	5.690	1974–1994
4	8	PAIN	1.57	0.35	52.5	34	4.836	1979–1996
5	5	STROKE	1.52	0.42	48.5	30	5.391	1976–1993
6	6	ARCH NEUROL-CHICAGO	1.52	0.42	45.7	29	5.204	1960–1996
7	24	J NEUROSURG	1.50	0.39	44.0	28	2.242	1974–1992
8	9	J NEUROPATH EXP NEUR	1.47	0.44	41.1	25	4.371	1956–1999
9	10	J NEUROL NEUROSUR PS	1.45	0.40	40.5	25	3.630	1954–1995
10	32	J CLIN EXP NEUROPSYC	1.42	0.36	37.2	23	1.590	1984–1993
11	12	MOVEMENT DISORD	1.38	0.38	34.6	22	3.323	1989–1994
12	13	J AFFECT DISORDERS	1.35	0.35	28.7	19	3.138	1982–1998
13	27	J NERV MENT DIS	1.35	0.39	32.0	19	1.957	1980–1995
14	22	SPINE	1.34	0.39	29.8	19	2.351	1978–1995
15	11	EPILEPSIA	1.34	0.41	29.3	19	3.526	1962–1998
16	19	NEUROSURGERY	1.33	0.42	27.8	18	2.692	1977–1997
17	7	SLEEP	1.30	0.37	26.8	17	5.126	1992–1999
18	18	ACTA NEUROPATHOL	1.29	0.39	27.6	17	2.694	1965–1996
19	21	J NEUROL SCI	1.25	0.38	25.2	15	2.412	1977–1994
20	26	DEV MED CHILD NEUROL	1.25	0.41	22.6	15	2.008	1976–1995
21	23	AM J NEURORADIOL	1.23	0.39	22.3	14	2.279	1979–2000
22	17	HEADACHE	1.22	0.32	20.3	14	2.740	1989–1995

table continues on next page . . .

. . . table continued from previous page

Rank			$\mathbf{p_{ss}(q\|J)}$		n			Steady-state
**AUC**	**JIF**	**Journal abbreviation**	$\overline{\mathbf{q}}$	σ	$\overline{\mathbf{n}}$	**Q2**	**JIF**	**period**
23	20	J PAIN SYMPTOM MANAG	1.18	0.42	19.5	13	2.437	1990–2000
24	15	J NEUROL	1.14	0.37	16.6	11	2.984	1974–2001
25	38	CAN J NEUROL SCI	1.12	0.41	18.0	11	1.180	1976–1997
26	25	EPILEPSY RES	1.14	0.60	19.5	11	2.088	1986–1999
27	16	J NEURAL TRANSM	1.10	0.40	16.7	10	2.938	1985–1998
28	14	EUR ARCH PSY CLIN N	1.09	0.39	15.9	10	3.042	1983–2001
29	3	CEPHALALGIA	1.13	0.55	18.3	11	6.049	1988–1997
30	28	J NEURO-ONCOL	1.08	0.36	14.0	10	1.848	1983–2000
31	30	NEURORADIOLOGY	1.08	0.41	15.8	10	1.625	1971–1994
32	33	PEDIATR NEUROL	1.06	0.34	12.7	9	1.542	1986–1997
33	29	ACTA NEUROL SCAND	1.02	0.50	14.7	8	1.833	1963–1995
34	40	EUR NEUROL	0.97	0.38	12.3	8	1.031	1967–1997
35	37	ACTA NEUROCHIR	0.98	0.48	11.8	8	1.212	1970–1995
36	35	J CHILD NEUROL	0.95	0.40	11.4	7	1.350	1985–2000
37	39	SURG NEUROL	0.97	0.41	10.0	7	1.057	1993–1998
38	31	BRAIN DEV-JPN	0.95	0.40	11.1	7	1.598	1980–1995
39	36	CHILD NERV SYST	0.86	0.38	7.6	6	1.257	1995–2000
40	34	CLIN NEUROL NEUROSUR	0.77	0.38	6.6	4	1.506	1977–1998
41	43	NEUROCHIRURGIE	0.66	0.39	5.2	3	0.386	1964–1999
42	41	NERVENARZT	0.63	0.38	5.0	3	0.711	1971–2001
43	42	NERVENHEILKUNDE	-0.02	0.44	1.1	0	0.396	1982–2003
44	44	ZH NEVROPATOL PSIKH	-0.20	0.39	0.4	0	0.129	1956–1995

COMPUTER SCIENCE, ARTIFICIAL INTELLIGENCE

ISI Category Description Computer Science, Artificial Intelligence covers resources that focus on research and techniques to create machines that attempt to efficiently reason, problem-solve, use knowledge representation, and perform analysis of contradictory or ambiguous information. This category includes resources on artificial intelligence technologies such as expert systems, fuzzy systems, natural language processing, speech recognition, pattern recognition, computer vision, decision-support systems, knowledge bases, and neural networks.

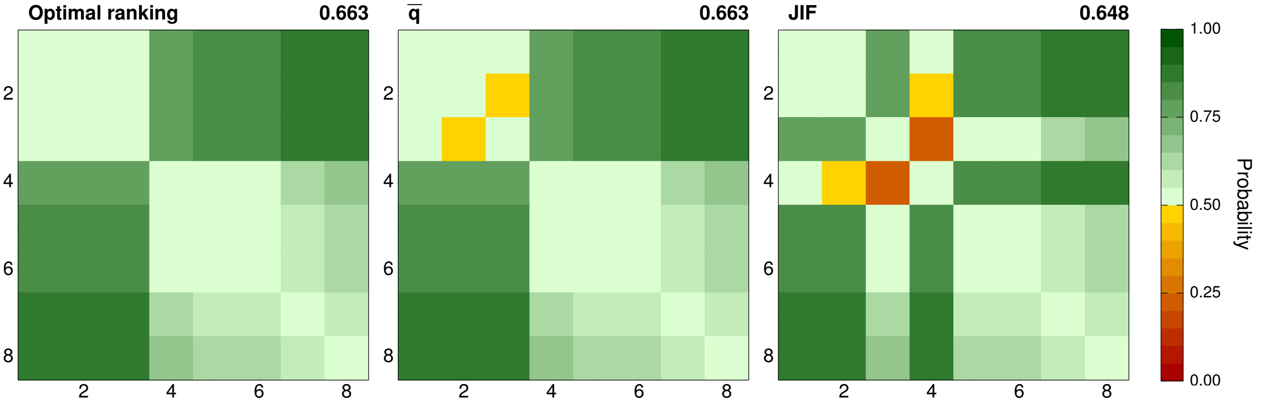

The color of each cell represents the probability of picking a paper with more citations from the higher-ranked journal. Green cells indicate adequate ranking, whereas red cells indicate that the ranking is inadequate (the probability is less than $\frac{1}{2}$). The value of M, the multi-class AUC statistic for each ranking scheme, is above each matrix.

Rank			$p_{ss}(q\|J)$		n			Steady-state
AUC	JIF	Journal abbreviation	$\overline{q}$	σ	$\overline{n}$	Q2	JIF	period
1	1	IEEE T PATTERN ANAL	1.51	0.42	67.3	29	4.306	1982–1993
2	4	ARTIF INTELL	1.48	0.41	63.6	28	2.271	1969–1992
3	2	IEEE T NEURAL NETWOR	1.49	0.32	48.8	27	2.620	1990–1992
4	3	CHEMOMETR INTELL LAB	0.96	0.42	14.8	8	2.450	1986–2002
5	5	NEURAL NETWORKS	0.93	0.39	11.5	6	2.000	1995–2002
6	6	PATTERN RECOGN	0.92	0.42	12.6	6	1.822	1967–1997
7	7	IMAGE VISION COMPUT	0.79	0.40	8.2	5	1.171	1984–2001
8	8	PATTERN RECOGN LETT	0.70	0.41	6.5	3	0.952	1983–1999

COMPUTER SCIENCE, HARDWARE & ARCHITECTURE

ISI Category Description Computer Science, Hardware & Architecture covers resources on the physical components of a computer system: main and logic boards, internal buses and interfaces, static and dynamic memory, storage devices and storage media, power supplies, input and output devices, networking interfaces, and networking hardware such as routers and bridges. Resources in this category also cover the architecture of computing devices, such as SPARC, RISC, and CISC designs, as well as scalable, parallel, and multi-processor computing architectures.

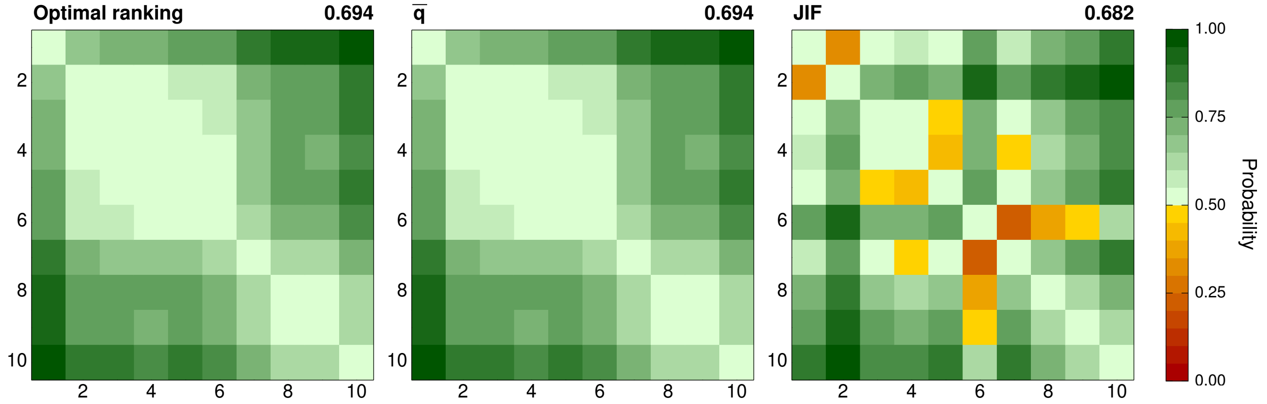

The color of each cell represents the probability of picking a paper with more citations from the higher-ranked journal. Green cells indicate adequate ranking, whereas red cells indicate that the ranking is inadequate (the probability is less than $\frac{1}{2}$). The value of M, the multi-class AUC statistic for each ranking scheme, is above each matrix.

Rank			$p_{ss}(q\|J)$		n			Steady-state
AUC	JIF	Journal abbreviation	$\overline{q}$	σ	$\overline{n}$	Q2	JIF	period
1	2	IEEE T NEURAL NETWOR	1.49	0.32	48.8	27	2.620	1990–1992
2	1	J ACM	1.15	0.49	30.0	12	2.917	1957–2002
3	5	IEEE T COMPUT	1.09	0.46	23.1	10	1.426	1971–1991
4	3	COMMUN ACM	1.05	0.51	28.2	9	1.509	1968–1997
5	7	IEEE T COMPUT AID D	1.02	0.42	14.7	8	0.838	1983–1989
6	4	IBM J RES DEV	0.97	0.47	20.0	7	1.483	1956–2001
7	8	IEEE T RELIAB	0.70	0.47	7.1	3	0.800	1963–1995
8	9	COMPUT J	0.47	0.46	5.3	2	0.593	1972–2003
9	6	COMPUTER	0.47	0.62	5.2	2	1.289	1995–2001
10	10	IEICE T FUND ELECTR	0.18	0.42	1.7	1	0.312	1991–2002

COMPUTER SCIENCE, INFORMATION SYSTEMS

ISI Category Description Computer Science, Information Systems covers resources that focus on the acquisition, processing, storage, management, and dissemination of electronic information that can be read by humans, machines, or both. This category also includes resources for telecommunications systems and discipline-specific subjects such as medical informatics, chemical information processing systems, geographical information systems, and some library science.

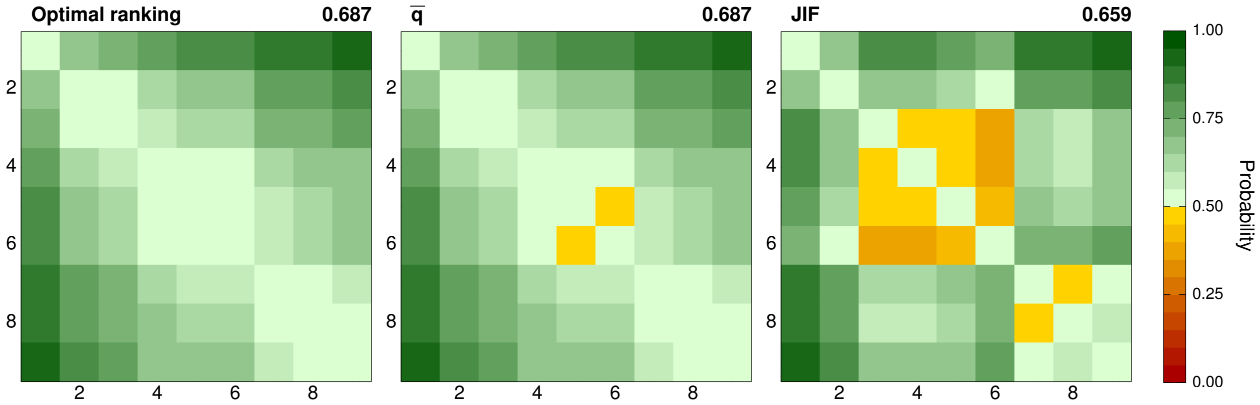

The color of each cell represents the probability of picking a paper with more citations from the higher-ranked journal. Green cells indicate adequate ranking, whereas red cells indicate that the ranking is inadequate (the probability is less than $\frac{1}{2}$). The value of M, the multi-class AUC statistic for each ranking scheme, is above each matrix.

**Rank**			$\mathbf{p_{ss}(q\|J)}$		**n**			**Steady-state**
**AUC**	**JIF**	**Journal abbreviation**	$\overline{\mathbf{q}}$	σ	$\overline{\mathbf{n}}$	**Q2**	**JIF**	**period**
1	1	J ACM	1.15	0.49	30.0	12	2.917	1957–2002
2	2	IEEE T INFORM THEORY	0.74	0.44	10.0	4	1.938	2000–2003
3	6	INFORM PROCESS LETT	0.67	0.48	7.2	3	0.532	1976–1993
4	5	COMPUT J	0.47	0.46	5.3	2	0.593	1972–2003
5	4	INFORM SOFTWARE TECH	0.37	0.40	2.6	1	0.726	1986–2002
6	3	INFORM SCIENCES	0.37	0.45	3.3	1	1.003	1988–2002
7	8	IEICE T FUND ELECTR	0.18	0.42	1.7	1	0.312	1991–2002
8	7	COMPUT COMMUN	0.09	0.46	1.5	0	0.444	1990–2006
9	9	IEICE T INF SYST	0.04	0.44	1.1	0	0.280	1991–2001

COMPUTER SCIENCE, INTERDISCIPLINARY APPLICATIONS

ISI Category Description Computer Science, Interdisciplinary Applications includes resources concerned with the application of computer technology and methodology to other disciplines, such as information management, engineering, biology, medicine, environmental studies, geosciences, arts and humanities, agriculture, chemistry, and physics.

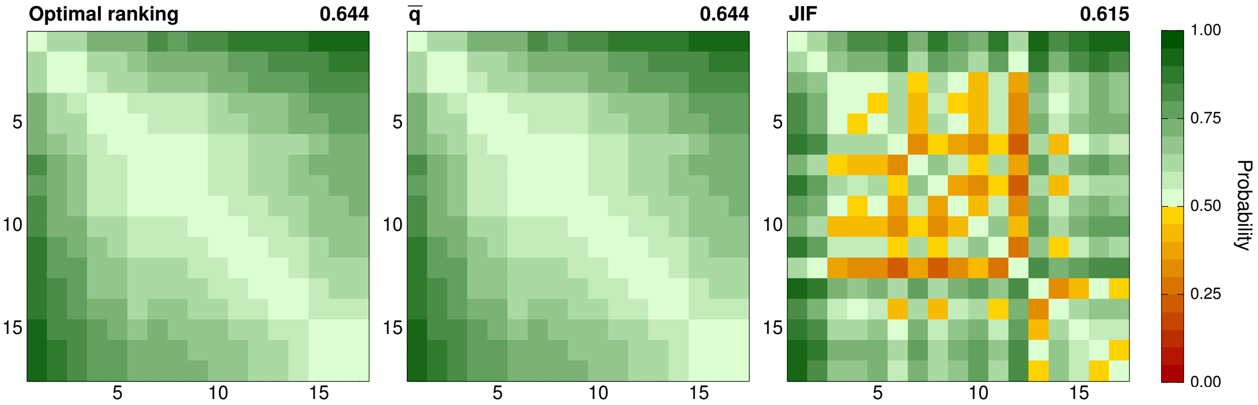

The color of each cell represents the probability of picking a paper with more citations from the higher-ranked journal. Green cells indicate adequate ranking, whereas red cells indicate that the ranking is inadequate (the probability is less than $\frac{1}{2}$). The value of M, the multi-class AUC statistic for each ranking scheme, is above each matrix.

**Rank**			$\mathbf{p_{ss}(q\|J)}$		**n**			**Steady-state**
**AUC**	**JIF**	**Journal abbreviation**	$\overline{\mathbf{q}}$	σ	$\overline{\mathbf{n}}$	**Q2**	**JIF**	**period**
1	1	IEEE T MED IMAGING	1.25	0.40	27.5	15	3.757	1985–1999
2	2	J COMPUT PHYS	1.08	0.41	19.6	10	2.328	1994–1999
3	12	IEEE T COMPUT AID D	1.02	0.42	14.7	8	0.838	1983–1989
4	10	INT J NUMER METH FL	0.85	0.43	11.5	5	0.870	1980–1998
5	7	MED BIOL ENG COMPUT	0.82	0.42	9.2	5	1.018	1976–1999
6	3	COMPUT PHYS COMMUN	0.75	0.52	17.4	4	1.595	1970–2000
7	5	SCIENTOMETRICS	0.72	0.39	6.2	4	1.363	1989–2002
8	9	COMPUT OPER RES	0.72	0.42	7.1	3	0.893	1975–2000
9	4	COMPUT CHEM ENG	0.68	0.43	6.2	3	1.404	1994–2002
10	14	COMPUT METH PROG BIO	0.61	0.40	6.4	3	0.624	1984–2002
11	11	COMPUT STRUCT	0.56	0.39	4.5	2	0.846	1990–2002
12	8	COMPUT STAT DATA AN	0.51	0.41	4.3	2	0.928	1982–2003
13	6	COMPUT IND	0.48	0.42	3.7	2	1.141	1982–2003
14	15	COMPUT MATH APPL	0.42	0.45	3.7	1	0.611	1979–2002
15	17	MATH COMPUT MODEL	0.31	0.47	2.9	1	0.432	1987–2002
16	13	COMPUT IND ENG	0.28	0.47	2.5	1	0.650	1975–2002
17	16	MATH COMPUT SIMULAT	0.26	0.44	2.3	1	0.534	1981–2003

COMPUTER SCIENCE, SOFTWARE ENGINEERING

ISI Category Description Computer Science, Software Engineering includes resources that are concerned with the programs, routines, and symbolic languages that control the functioning of the hardware and direct its operation. Also covered in this category are computer graphics, digital signal processing, and programming languages.

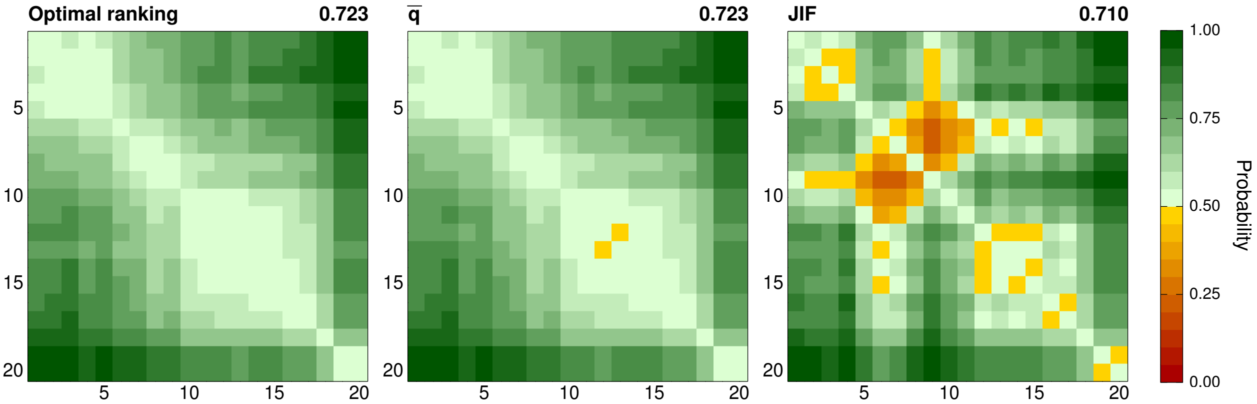

The color of each cell represents the probability of picking a paper with more citations from the higher-ranked journal. Green cells indicate adequate ranking, whereas red cells indicate that the ranking is inadequate (the probability is less than $\frac{1}{2}$). The value of M, the multi-class AUC statistic for each ranking scheme, is above each matrix.

Rank			$p_{ss}(q\|J)$		n			Steady-state
AUC	JIF	Journal abbreviation	$\overline{q}$	σ	$\overline{n}$	Q2	JIF	period
1	1	J ACM	1.15	0.49	30.0	12	2.917	1957–2002
2	9	MATH PROGRAM	1.11	0.43	18.6	10	1.117	1976–1998
3	4	COMPUT AIDED DESIGN	1.07	0.29	12.1	10	1.446	1997–1999
4	3	COMMUN ACM	1.05	0.51	28.2	9	1.509	1968–1997
5	2	IEEE T SOFTWARE ENG	1.03	0.44	17.2	8	2.132	1976–1999
6	10	ALGORITHMICA	0.90	0.46	12.1	6	0.850	1988–1997
7	8	IMAGE VISION COMPUT	0.79	0.40	8.2	5	1.171	1984–2001
8	5	IEEE COMPUT GRAPH	0.72	0.54	9.5	3	1.429	1981–1999
9	11	IEEE T RELIAB	0.70	0.47	7.1	3	0.800	1963–1995
10	7	IEEE SOFTWARE	0.55	0.60	6.2	2	1.252	1985–1997
11	13	COMPUT J	0.47	0.46	5.3	2	0.593	1972–2003
12	15	SOFTWARE PRACT EXPER	0.44	0.44	4.5	2	0.543	1976–2002
13	6	COMPUTER	0.47	0.62	5.2	2	1.289	1995–2001
14	14	J SYST SOFTWARE	0.38	0.43	2.9	1	0.592	1983–2003
15	12	INFORM SOFTWARE TECH	0.37	0.40	2.6	1	0.726	1986–2002
16	17	MATH COMPUT MODEL	0.31	0.47	2.9	1	0.432	1987–2002
17	16	MATH COMPUT SIMULAT	0.26	0.44	2.3	1	0.534	1981–2003
18	18	IEICE T INF SYST	0.04	0.44	1.1	0	0.280	1991–2001
19	20	COMPUT GRAPH WORLD	-0.45	0.35	0.1	0	0.008	1981–2002
20	19	DR DOBBS J	-0.63	0.47	0.2	0	0.039	1981–2004

COMPUTER SCIENCE, THEORY & METHODS

ISI Category Description Computer Science, Theory & Methods includes resources that emphasize experimental computer processing methods or programming techniques such as parallel computing, distributed computing, logic programming, object-oriented programming, high-speed computing, and supercomputing.

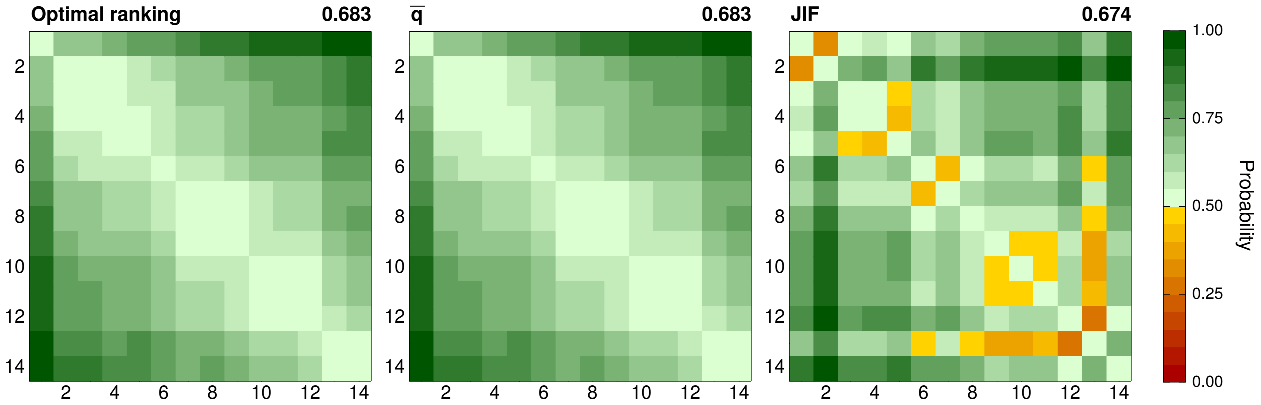

The color of each cell represents the probability of picking a paper with more citations from the higher-ranked journal. Green cells indicate adequate ranking, whereas red cells indicate that the ranking is inadequate (the probability is less than $\frac{1}{2}$). The value of M, the multi-class AUC statistic for each ranking scheme, is above each matrix.

Rank			$p_{ss}(q\|J)$		n			Steady-state
AUC	JIF	Journal abbreviation	$\overline{q}$	σ	$\overline{n}$	Q2	JIF	period
1	2	IEEE T NEURAL NETWOR	1.49	0.32	48.8	27	2.620	1990–1992
2	1	J ACM	1.15	0.49	30.0	12	2.917	1957–2002
3	5	FUZZY SET SYST	1.11	0.45	23.4	11	1.181	1980–1987
4	3	COMMUN ACM	1.05	0.51	28.2	9	1.509	1968–1997
5	4	SIAM J COMPUT	1.02	0.40	16.1	8	1.361	1989–1997
6	7	INFORM COMPUT	0.94	0.46	15.7	6	1.107	1983–1996
7	13	J PARALLEL DISTR COM	0.80	0.43	10.4	5	0.430	1984–1992
8	6	IMAGE VISION COMPUT	0.79	0.40	8.2	5	1.171	1984–2001
9	8	THEOR COMPUT SCI	0.73	0.45	8.4	4	0.843	1988–1996
10	11	COMPUT METH PROG BIO	0.61	0.40	6.4	3	0.624	1984–2002
11	10	J SYMB COMPUT	0.61	0.41	5.0	3	0.641	1994–2002
12	9	PARALLEL COMPUT	0.57	0.42	5.0	2	0.685	1989–2001
13	12	J SYST SOFTWARE	0.38	0.43	2.9	1	0.592	1983–2003
14	14	INT J SYST SCI	0.28	0.43	2.3	1	0.343	1986–2001

CONSTRUCTION & BUILDING TECHNOLOGY

ISI Category Description Construction & Building Technology includes resources that provide information on the physical features and design of structures (e.g., buildings, dams, bridges, tunnels) and the materials used to construct them (concrete, cement, steel). Other topics covered in this category include heating and air conditioning, energy systems, and indoor air quality.

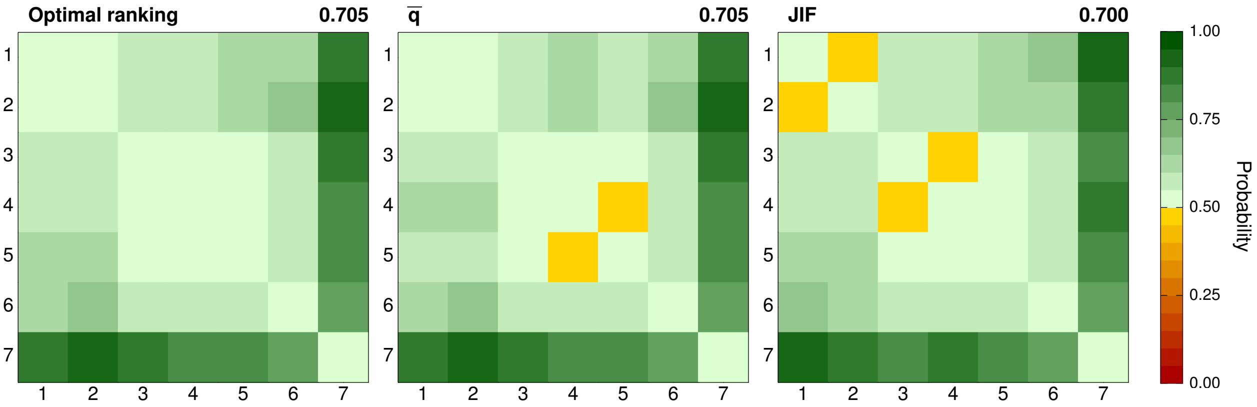

The color of each cell represents the probability of picking a paper with more citations from the higher-ranked journal. Green cells indicate adequate ranking, whereas red cells indicate that the ranking is inadequate (the probability is less than $\frac{1}{2}$). The value of M, the multi-class AUC statistic for each ranking scheme, is above each matrix.

**Rank**			$\mathbf{p_{ss}(q\|J)}$		**n**			**Steady-state**
**AUC**	**JIF**	**Journal abbreviation**	$\overline{\mathbf{q}}$	σ	$\overline{\mathbf{n}}$	**Q2**	**JIF**	**period**
1	2	ACI MATER J	0.87	0.44	8.8	6	0.764	1986–1998
2	1	CEMENT CONCRETE RES	0.84	0.34	7.3	5	1.185	1996–2000
3	4	J STRUCT ENG-ASCE	0.73	0.41	6.9	4	0.635	1982–2000
4	3	ACI STRUCT J	0.69	0.43	6.7	4	0.657	1986–1999
5	5	J CONSTR ENG M ASCE	0.71	0.43	5.3	3	0.471	1989–1999
6	6	MATER STRUCT	0.57	0.47	5.7	2	0.459	1990–1996
7	7	ASHRAE J	-0.21	0.46	0.6	0	0.297	1971–2004

CRITICAL CARE MEDICINE

ISI Category Description Critical Care Medicine covers resources on healthcare specialties that focus on the care of patients with acute, life-threatening illness or injury. This category covers resources such as heart attack; poisoning; burns, pneumonia; surgical complications; premature birth; trauma including head trauma; stroke, and other neural injuries; intensive care anesthesia; and resuscitation.

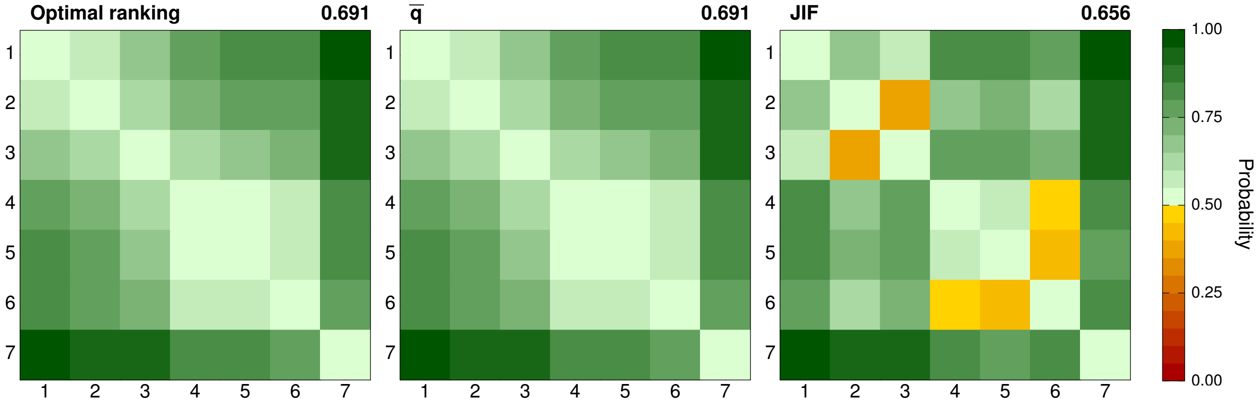

The color of each cell represents the probability of picking a paper with more citations from the higher-ranked journal. Green cells indicate adequate ranking, whereas red cells indicate that the ranking is inadequate (the probability is less than $\frac{1}{2}$). The value of M, the multi-class AUC statistic for each ranking scheme, is above each matrix.

Rank AUC	Rank JIF	Journal abbreviation	$p_{ss}(q\|J)$ $\overline{q}$	σ	n $\overline{n}$	Q2	JIF	Steady-state period
1	1	CRIT CARE MED	1.35	0.37	30.4	19	6.599	1996–1998
2	3	J TRAUMA	1.27	0.41	25.6	16	2.035	1989–1993
3	2	INTENS CARE MED	1.05	0.38	15.2	9	4.406	1976–2002
4	6	ANAESTH INTENS CARE	0.87	0.43	9.9	5	0.945	1977–1996
5	4	BURNS	0.82	0.37	7.5	5	1.139	1988–2001
6	5	INJURY	0.72	0.43	6.5	4	1.067	1973–1996
7	7	ANASTH INTENSIVMED	0.10	0.49	1.4	0	0.567	1996–2003

CRYSTALLOGRAPHY

ISI Category Description Crystallography covers resources that report on the study of the formation, structure, and properties of crystals. This category also includes resources on X-ray crystallography, the study of the internal structure of crystals through the use of X-ray diffraction.

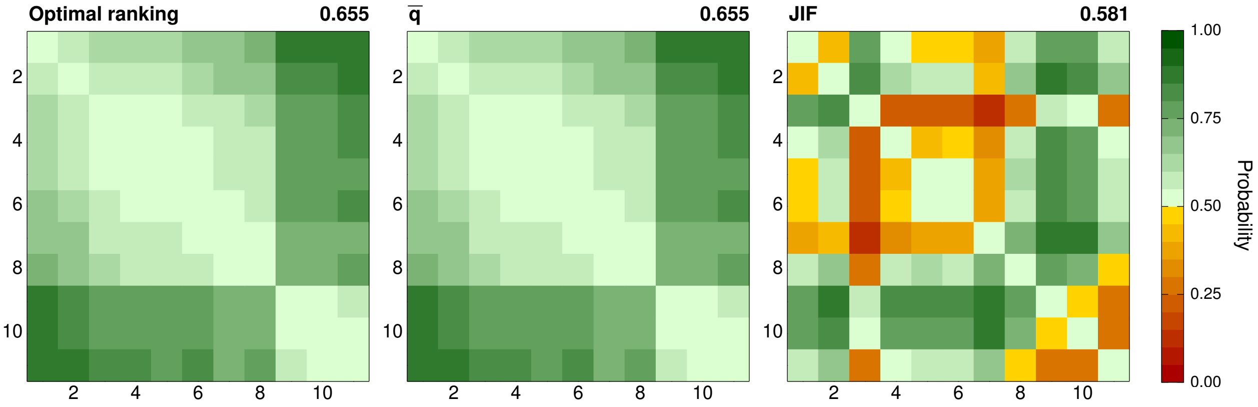

The color of each cell represents the probability of picking a paper with more citations from the higher-ranked journal. Green cells indicate adequate ranking, whereas red cells indicate that the ranking is inadequate (the probability is less than $\frac{1}{2}$). The value of M, the multi-class AUC statistic for each ranking scheme, is above each matrix.

Rank			$p_{ss}(q\|J)$		n			Steady-state
**AUC**	**JIF**	**Journal abbreviation**	$\overline{q}$	σ	$\overline{n}$	**Q2**	**JIF**	**period**
1	7	LIQ CRYST	1.21	0.42	24.8	18	1.417	1985–1987
2	2	ACTA CRYSTALLOGR B	1.19	0.36	22.6	13	2.172	1975–1993
3	5	J CRYST GROWTH	1.11	0.42	19.0	11	1.809	1970–1989
4	6	ACTA CRYSTALLOGR A	1.10	0.46	41.7	9	1.676	1978–1996
5	1	J APPL CRYSTALLOGR	1.06	0.46	32.4	9	2.495	1967–1993
6	4	POLYHEDRON	1.00	0.33	11.2	8	1.843	1990–1997
7	11	MOL CRYST LIQ CRYST	0.94	0.44	13.3	7	0.478	1974–1989
8	8	ACTA CRYSTALLOGR C	0.92	0.34	9.9	6	0.896	1982–1984
9	3	Z KRISTALLOGR	0.55	0.40	4.2	2	1.897	1993–2004
10	10	PHASE TRANSIT	0.51	0.46	4.8	2	0.830	1986–2005
11	9	CRYST RES TECHNOL	0.45	0.40	3.1	2	0.863	1985–2003

DENTISTRY, ORAL SURGERY & MEDICINE

ISI Category Description Dentistry, Oral Surgery & Medicine covers resources on the anatomy, physiology, biochemistry, and pathology of the teeth and oral cavity. This category includes specific resources on periodontal disease, dental implants, oral and maxillofacial surgery, oral pathology, and oral surgery. Coverage also includes resources on community dentistry, public health dentistry, and pediatric dentistry.

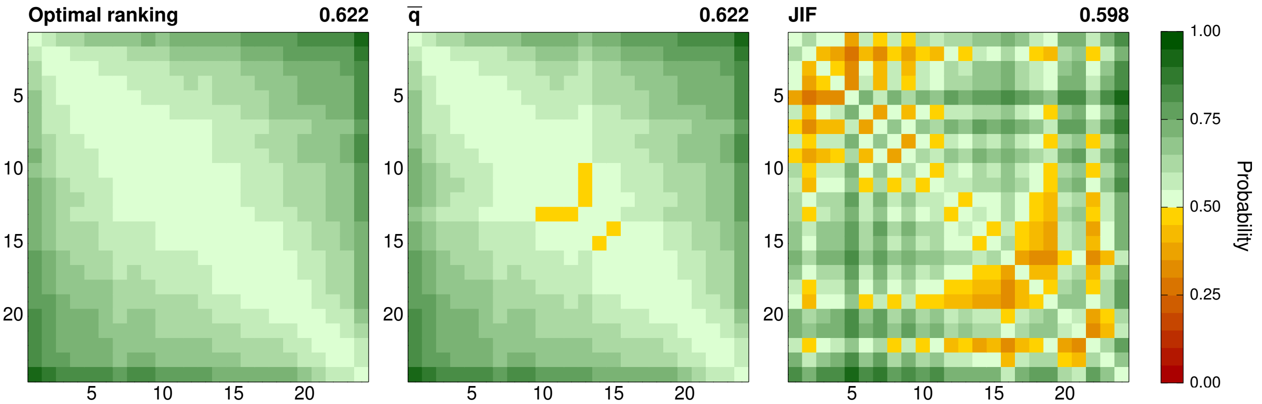

The color of each cell represents the probability of picking a paper with more citations from the higher-ranked journal. Green cells indicate adequate ranking, whereas red cells indicate that the ranking is inadequate (the probability is less than $\frac{1}{2}$). The value of M, the multi-class AUC statistic for each ranking scheme, is above each matrix.

Rank			$p_{ss}(q\|J)$		n			Steady-state
**AUC**	**JIF**	**Journal abbreviation**	$\overline{q}$	σ	$\overline{n}$	**Q2**	**JIF**	**period**
1	5	J CLIN PERIODONTOL	1.40	0.39	42.1	22	2.380	1973–1988
2	7	ORAL MICROBIOL IMMUN	1.28	0.32	21.6	16	2.089	1987–1993
3	9	J PERIODONTOL	1.24	0.42	25.2	14	1.703	1980–1990
4	1	J DENT RES	1.20	0.40	21.6	13	3.475	1980–2000
5	4	DENT MATER	1.18	0.37	19.1	12	2.381	1987–2001
6	3	J PERIODONTAL RES	1.15	0.35	17.3	12	2.472	1991–1999
7	19	AM J ORTHOD DENTOFAC	1.15	0.48	18.5	11	0.968	1963–1990
8	11	J ORAL PATHOL MED	1.12	0.36	16.6	11	1.530	1972–1995
9	6	CARIES RES	1.11	0.34	14.0	10	2.304	1993–1999
10	22	CLEFT PALATE-CRAN J	1.03	0.37	16.3	9	0.724	1990–1992
11	8	COMMUNITY DENT ORAL	1.06	0.35	13.3	9	1.870	1975–1999
12	10	ARCH ORAL BIOL	1.05	0.34	13.2	9	1.655	1987–1996
13	18	ACTA ODONTOL SCAND	1.04	0.39	15.2	9	1.017	1967–1994
14	13	ORAL SURG ORAL MED O	1.01	0.41	13.5	8	1.221	1970–1995
15	2	J ENDODONT	1.01	0.42	13.5	8	3.077	1981–1995
16	12	J ORAL MAXIL SURG	0.98	0.40	12.7	7	1.252	1981–1996
17	17	J ORAL REHABIL	0.97	0.38	11.4	7	1.044	1973–1995
18	15	J CRANIO MAXILL SURG	0.95	0.37	9.8	7	1.171	1986–2000
19	14	INT J ORAL MAX SURG	0.90	0.36	9.7	6	1.212	1985–1999
20	23	BRIT J ORAL MAX SURG	0.87	0.38	8.6	6	0.654	1983–1998
21	20	J PROSTHET DENT	0.82	0.46	9.7	5	0.879	1960–1999
22	16	J AM DENT ASSOC	0.76	0.60	10.5	4	1.162	1963–1997
23	21	BRIT DENT J	0.72	0.55	8.4	3	0.848	1970–1997
24	24	AUST DENT J	0.56	0.41	4.2	2	0.568	1978–2000

DERMATOLOGY

ISI Category Description Dermatology covers resources on the anatomy, physiology, and pathology of the skin. It contains resources on investigative and experimental dermatology, contact dermatitis, dermatologic surgery, dermatologic pathology, and dermatologic oncology. This category also includes specific resources on burns, wounds and leprosy.

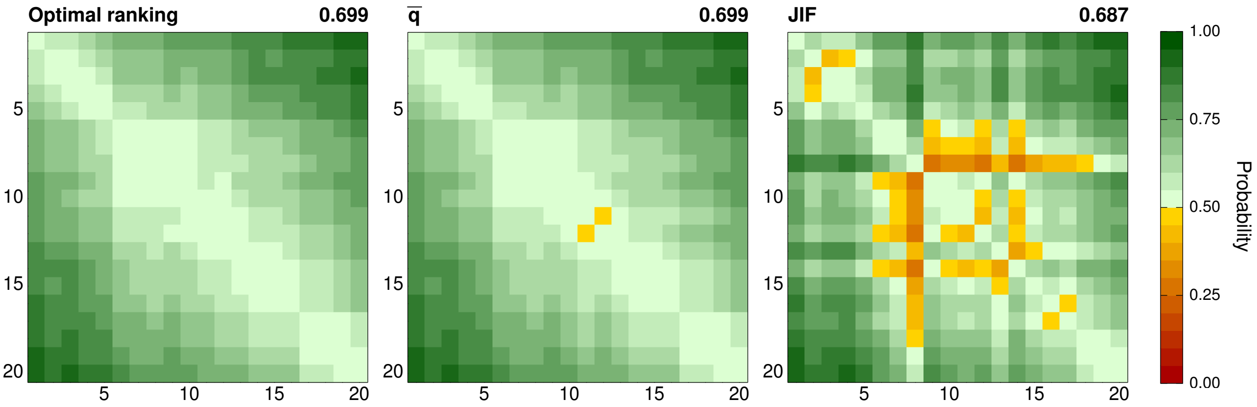

The color of each cell represents the probability of picking a paper with more citations from the higher-ranked journal. Green cells indicate adequate ranking, whereas red cells indicate that the ranking is inadequate (the probability is less than $\frac{1}{2}$). The value of M, the multi-class AUC statistic for each ranking scheme, is above each matrix.

Rank			$p_{ss}(q\|J)$		n			Steady-state
**AUC**	**JIF**	**Journal abbreviation**	$\overline{q}$	σ	$\overline{n}$	**Q2**	**JIF**	**period**
1	1	J INVEST DERMATOL	1.43	0.38	35.7	23	4.535	1974–1992
2	3	ARCH DERMATOL	1.35	0.41	27.7	20	2.851	1967–1995
3	4	J AM ACAD DERMATOL	1.32	0.36	25.5	18	2.553	1978–1995
4	2	BRIT J DERMATOL	1.25	0.40	22.8	15	3.334	1967–1995
5	5	CONTACT DERMATITIS	1.20	0.35	18.6	13	2.446	1975–1995
6	9	J CUTAN PATHOL	1.08	0.36	13.6	10	1.582	1973–1998
7	14	AM J DERMATOPATH	1.08	0.44	13.5	10	1.100	1986–1995
8	12	DERMATOL CLIN	1.04	0.40	13.7	9	1.179	1992–1997
9	6	DERMATOLOGY	1.04	0.35	12.5	9	1.854	1991–1995
10	10	ARCH DERMATOL RES	1.02	0.38	13.1	8	1.333	1976–2000
11	11	CLIN EXP DERMATOL	0.95	0.37	10.8	7	1.295	1977–1995
12	7	ACTA DERM-VENEREOL	0.97	0.52	12.4	7	1.837	1960–1995
13	15	PEDIATR DERMATOL	0.89	0.37	8.7	6	1.014	1987–1998
14	13	BURNS	0.82	0.37	7.5	5	1.139	1988–2001
15	17	MYCOSES	0.81	0.36	8.0	5	0.959	1983–1999
16	16	INT J DERMATOL	0.80	0.40	7.3	5	0.998	1973–2000
17	18	CUTIS	0.71	0.43	5.9	4	0.920	1974–1996
18	8	CLIN DERMATOL	0.69	0.41	5.9	3	1.600	1986–1999
19	19	HAUTARZT	0.61	0.39	4.6	3	0.498	1968–1999
20	20	ANN DERMATOL VENER	0.53	0.41	3.7	2	0.495	1983–1999

DEVELOPMENTAL BIOLOGY

ISI Category Description Developmental Biology includes resources focused on the specific mechanisms of cell, tissue, and organism development, as well as gametogenesis, fertilization, biochemistry and molecular genetic control of development, cell biology of gametes and zygotes, and embryology.

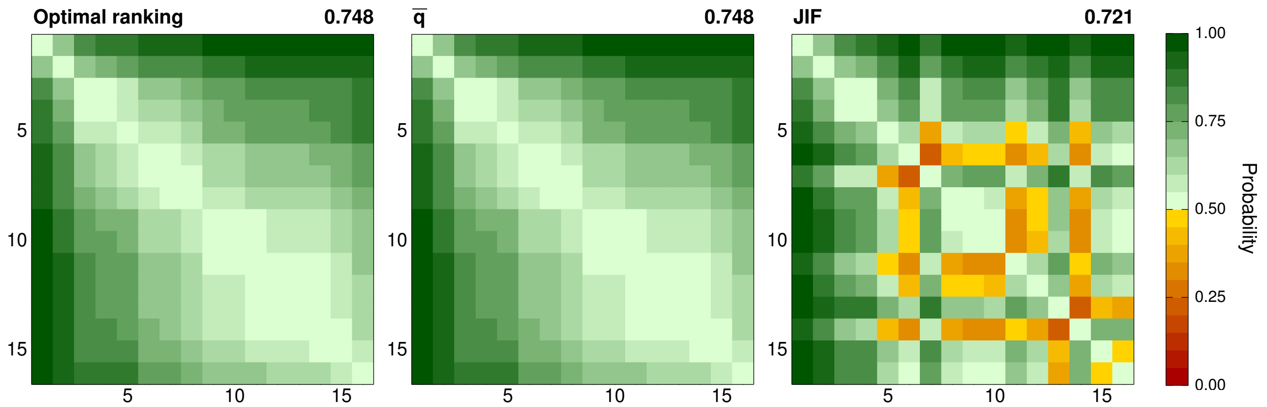

The color of each cell represents the probability of picking a paper with more citations from the higher-ranked journal. Green cells indicate adequate ranking, whereas red cells indicate that the ranking is inadequate (the probability is less than $\frac{1}{2}$). The value of M, the multi-class AUC statistic for each ranking scheme, is above each matrix.

Rank			$p_{ss}(q\|J)$		n			Steady-state
**AUC**	**JIF**	**Journal abbreviation**	$\overline{q}$	σ	$\overline{n}$	**Q2**	**JIF**	**period**
1	1	GENE DEV	2.04	0.30	143.5	106	15.050	1988–1996
2	2	DEVELOPMENT	1.83	0.40	87.2	61	7.764	1990–1996
3	3	DEV BIOL	1.57	0.36	49.9	33	4.893	1958–1997
4	4	MECH DEVELOP	1.52	0.34	43.1	30	3.836	1989–1997
5	7	DEV DYNAM	1.45	0.32	37.0	26	3.169	1991–1997
6	14	DEV BRAIN RES	1.34	0.35	27.2	19	1.598	1982–1993
7	11	MOL REPROD DEV	1.34	0.37	26.9	18	2.379	1989–1995
8	5	DIFFERENTIATION	1.26	0.39	26.0	15	3.745	1973–1999
9	12	DEV PSYCHOBIOL	1.17	0.37	18.6	12	1.946	1971–1997
10	8	PLACENTA	1.15	0.37	17.9	11	2.969	1980–2000
11	9	INT J DEV NEUROSCI	1.10	0.36	15.4	10	2.924	1983–2000
12	10	REPROD FERT DEVELOP	1.07	0.37	14.5	10	2.541	1988–1998
13	6	INT J DEV BIOL	1.06	0.41	14.9	10	3.577	1991–1998
14	16	ANAT EMBRYOL	1.04	0.35	13.9	9	1.277	1993–1999
15	15	DEV GROWTH DIFFER	0.98	0.39	12.0	7	1.545	1968–2000
16	13	REPROD NUTR DEV	0.87	0.40	9.1	6	1.817	1979–2000

ECOLOGY

ISI Category Description Ecology covers resources concerning many areas relating to the study of the interrelationship of organisms and their environments, including ecological economics, ecological engineering, ecotoxicology, ecological modeling, evolutionary ecology, biogeography, chemical ecology, marine ecology, wildlife research, microbial ecology, molecular ecology, and population ecology. This category also includes general ecology resources and ones devoted to particular ecological systems.

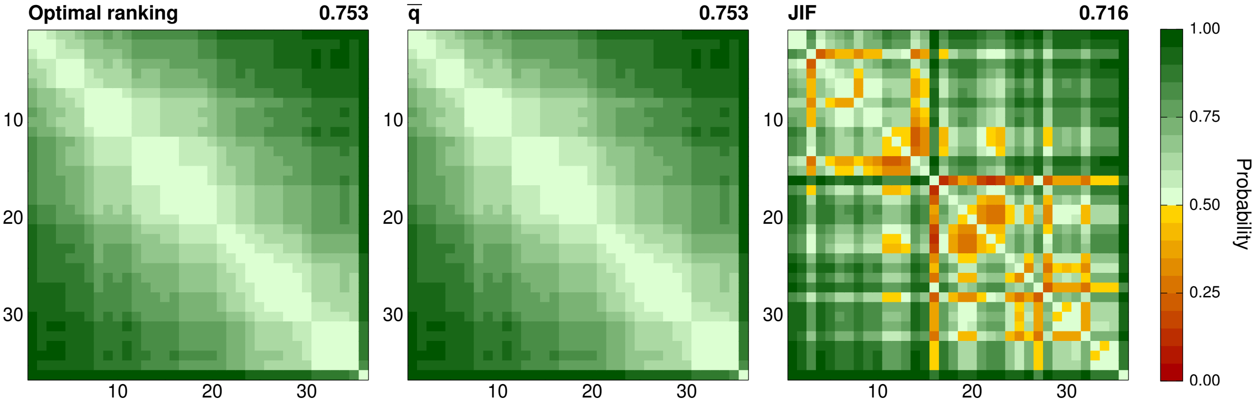

The color of each cell represents the probability of picking a paper with more citations from the higher-ranked journal. Green cells indicate adequate ranking, whereas red cells indicate that the ranking is inadequate (the probability is less than $\frac{1}{2}$). The value of M, the multi-class AUC statistic for each ranking scheme, is above each matrix.

Rank			$p_{ss}(q\|J)$		n			Steady-state
AUC	JIF	Journal abbreviation	$\overline{q}$	σ	$\overline{n}$	Q2	JIF	period
1	1	ECOLOGY	1.75	0.33	71.1	52	4.782	1974–1994
2	2	AM NAT	1.72	0.40	80.4	48	4.660	1967–1992
3	4	EVOLUTION	1.67	0.35	69.8	43	4.292	1973–1993
4	14	BEHAV ECOL SOCIOBIOL	1.60	0.31	44.4	36	2.316	1978–1990
5	8	J ANIM ECOL	1.57	0.34	47.5	33	3.390	1954–1996
6	5	J ECOL	1.55	0.35	45.1	32	4.239	1973–1996
7	15	MAR ECOL-PROG SER	1.47	0.31	33.6	26	2.286	1991–1995
8	6	CONSERV BIOL	1.42	0.42	37.5	23	3.762	1988–1998
9	7	FUNCT ECOL	1.42	0.32	29.6	23	3.417	1989–1996
10	9	OIKOS	1.41	0.35	34.2	22	3.381	1974–1995
11	10	OECOLOGIA	1.40	0.29	27.5	22	3.333	1994–1997
12	17	J EXP MAR BIOL ECOL	1.31	0.30	23.5	18	1.919	1988–1995
13	3	J APPL ECOL	1.31	0.36	25.6	17	4.527	1965–2000
14	23	BIOTROPICA	1.30	0.38	25.5	17	1.391	1975–1994
15	13	J VEG SCI	1.28	0.36	22.4	16	2.382	1989–1999
16	22	POLAR BIOL	1.27	0.33	20.8	16	1.502	1981–1994
17	28	ENVIRON BIOL FISH	1.24	0.42	21.0	15	0.934	1981–1990
18	12	BIOL CONSERV	1.21	0.38	22.1	14	2.854	1988–1996
19	11	J BIOGEOGR	1.21	0.36	20.4	13	2.878	1976–1998
20	21	J WILDLIFE MANAGE	1.19	0.34	18.3	13	1.538	1984–1995
21	18	J CHEM ECOL	1.11	0.31	14.4	10	1.896	1995–1998
22	32	AM MIDL NAT	1.07	0.36	14.5	9	0.667	1964–1995

table continues on next page . . .

...table continued from previous page

Rank			$p_{ss}(q\|J)$		n			Steady-state
**AUC**	**JIF**	**Journal abbreviation**	$\overline{q}$	σ	$\overline{n}$	**Q2**	**JIF**	**period**
23	26	WILDLIFE RES	1.05	0.32	11.6	9	1.032	1990–1997
24	24	PEDOBIOLOGIA	0.99	0.41	12.8	8	1.347	1965–1997
25	20	AGR ECOSYST ENVIRON	0.97	0.44	11.4	7	1.832	1982–2001
26	19	ECOL MODEL	0.91	0.40	11.3	6	1.888	1977–1998
27	30	J RANGE MANAGE	0.90	0.37	9.9	6	0.859	1966–1995
28	29	BIOCHEM SYST ECOL	0.89	0.36	9.2	6	0.906	1980–1994
29	31	WILDLIFE SOC B	0.87	0.40	8.7	6	0.843	1983–1998
30	25	J ARID ENVIRON	0.83	0.38	7.8	5	1.238	1989–2000
31	34	SOUTHWEST NAT	0.72	0.38	5.9	4	0.309	1980–1994
32	33	J NAT HIST	0.72	0.38	6.4	4	0.631	1966–2000
33	35	CAN FIELD NAT	0.69	0.40	5.8	3	0.073	1983–1993
34	16	LANDSCAPE URBAN PLAN	0.67	0.41	5.3	3	2.029	1985–2004
35	27	J SOIL WATER CONSERV	0.65	0.49	7.0	3	0.949	1966–2002
36	36	NAT HIST	-0.32	0.44	0.3	0	0.059	1989–2005

ECONOMICS

ISI Category Description Economics covers resources on all aspects, both theoretical and applied, of the production, distribution, and consumption of goods and services. These include generalist as well as specialist resources, such as political economy, agricultural economics, macroeconomics, microeconomics, econometrics, trade, and planning.

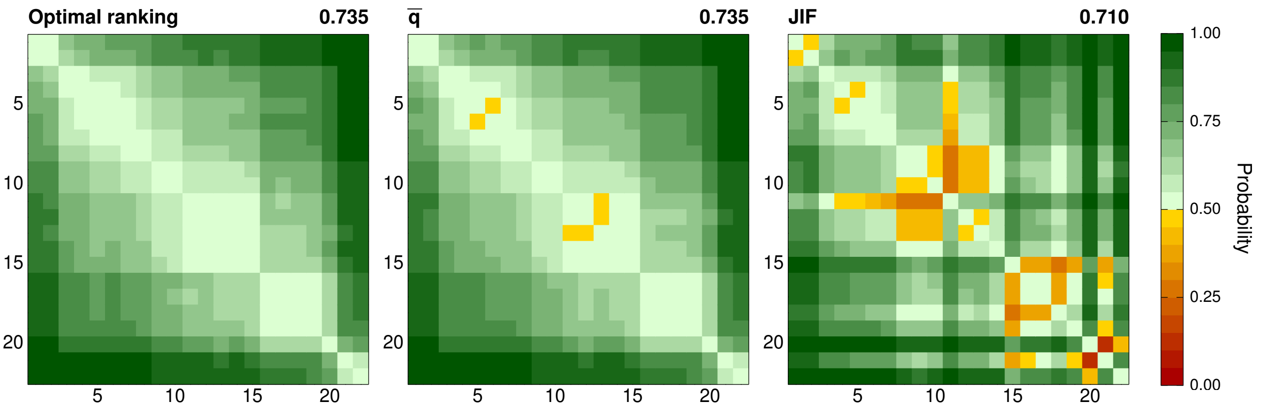

The color of each cell represents the probability of picking a paper with more citations from the higher-ranked journal. Green cells indicate adequate ranking, whereas red cells indicate that the ranking is inadequate (the probability is less than $\frac{1}{2}$). The value of M, the multi-class AUC statistic for each ranking scheme, is above each matrix.

Rank			$p_{ss}(q\|J)$		n			Steady-state
AUC	JIF	Journal abbreviation	$\overline{q}$	σ	$\overline{n}$	Q2	JIF	period
1	2	ECONOMETRICA	1.58	0.45	88.6	35	2.402	1980–1994
2	1	J POLIT ECON	1.56	0.46	69.7	33	3.194	1974–1996
3	3	AM ECON REV	1.29	0.51	37.9	17	1.876	1983–1994
4	11	J ECON THEORY	1.21	0.44	31.7	13	1.046	1968–1994
5	5	J ECONOMETRICS	1.13	0.45	28.1	11	1.669	1979–1996
6	4	REV ECON STAT	1.13	0.42	21.4	11	1.766	1964–1999
7	6	ECON J	1.09	0.43	20.4	10	1.629	1970–1998
8	7	J PUBLIC ECON	1.02	0.41	15.3	8	1.405	1975–2000
9	13	EUR ECON REV	0.92	0.41	12.3	7	1.019	1991–1999
10	12	INT ECON REV	0.92	0.45	13.9	6	1.031	1959–1999
11	10	J DEV ECON	0.80	0.43	10.1	5	1.075	1975–2000
12	8	WORLD DEV	0.80	0.39	8.2	5	1.298	1981–2001
13	9	AM J AGR ECON	0.80	0.49	9.6	4	1.196	1971–1996
14	14	J BANK FINANC	0.75	0.42	7.7	4	0.769	1979–2000
15	18	PUBLIC CHOICE	0.73	0.43	8.1	4	0.446	1968–1995
16	21	J ECON ISSUES	0.53	0.41	3.9	2	0.338	1972–1996
17	17	CAN J ECON	0.54	0.45	4.3	2	0.475	1988–2001
18	16	APPL ECON	0.52	0.41	3.9	2	0.522	1968–1997
19	19	ECON LETT	0.45	0.44	4.0	2	0.366	1977–1999
20	15	FUTURES	0.23	0.44	2.4	1	0.738	1967–2004
21	22	EKON CAS	-0.33	0.40	0.3	0	0.268	1979–2000
22	20	POLIT EKON	-0.50	0.35	0.1	0	0.363	1969–2005

EDUCATION, SCIENTIFIC DISCIPLINES

ISI Category Description Education, Scientific Disciplines covers all education resources in the scientific disciplines, including biology, pharmacy, biochemistry, engineering, chemistry, nutrition, and medicine.

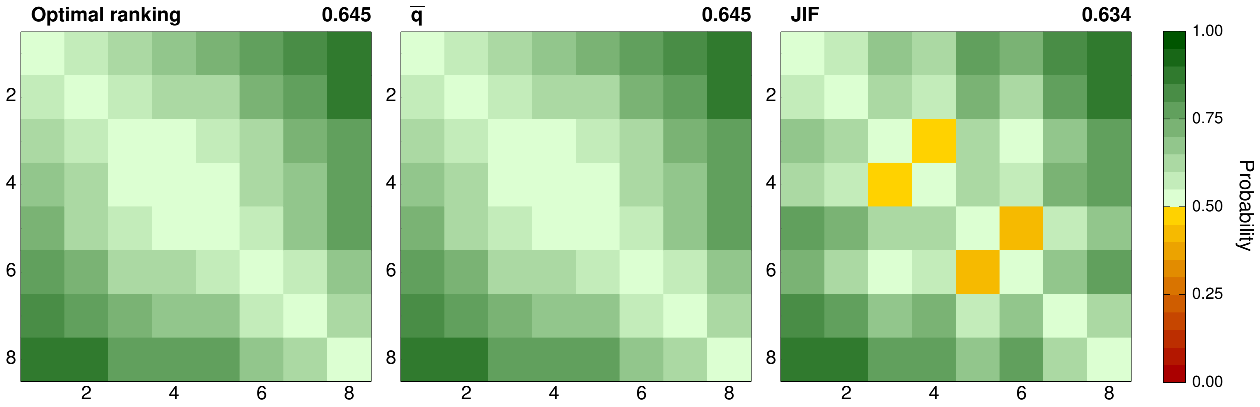

The color of each cell represents the probability of picking a paper with more citations from the higher-ranked journal. Green cells indicate adequate ranking, whereas red cells indicate that the ranking is inadequate (the probability is less than $\frac{1}{2}$). The value of M, the multi-class AUC statistic for each ranking scheme, is above each matrix.

**Rank**			$\mathbf{p_{ss}(q\|J)}$		**n**			**Steady-state**
**AUC**	**JIF**	**Journal abbreviation**	$\overline{\mathbf{q}}$	σ	$\overline{\mathbf{n}}$	**Q2**	**JIF**	**period**
1	1	ACAD MED	0.92	0.43	11.3	7	2.607	1988–1998
2	2	MED EDUC	0.81	0.43	8.4	5	2.467	1981–2002
3	4	J SCHOOL HEALTH	0.71	0.50	7.8	4	0.856	1983–1999
4	3	AM J PHYS	0.58	0.42	6.3	2	0.919	1960–1999
5	6	J CHEM EDUC	0.53	0.43	5.4	2	0.439	1968–1999
6	5	AM J PHARM EDUC	0.40	0.42	2.7	1	0.743	1966–2001
7	7	IEEE T EDUC	0.20	0.41	1.7	1	0.362	1964–2003
8	8	AM BIOL TEACH	-0.04	0.43	1.0	0	0.171	1969–2005

EDUCATION, SPECIAL

ISI Category Description Education, Special covers resources that are concerned with the education and development of persons with special needs, including the gifted as well as those with learning disabilities.

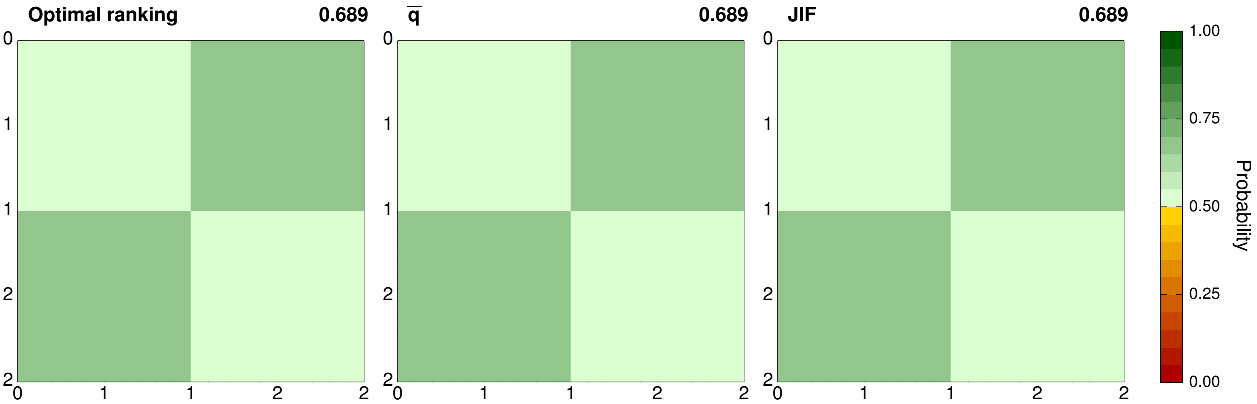

The color of each cell represents the probability of picking a paper with more citations from the higher-ranked journal. Green cells indicate adequate ranking, whereas red cells indicate that the ranking is inadequate (the probability is less than $\frac{1}{2}$). The value of M, the multi-class AUC statistic for each ranking scheme, is above each matrix.

**Rank**			$\mathbf{p_{ss}(q \mid J)}$		**n**			**Steady-state**
**AUC**	**JIF**	**Journal abbreviation**	$\overline{\mathbf{q}}$	σ	$\overline{\mathbf{n}}$	**Q2**	**JIF**	**period**
1	1	EXCEPT CHILDREN	1.11	0.39	16.6	10	3.226	1985–2000
2	2	MENT RETARD	0.81	0.45	7.5	4	1.373	1971–1998

EDUCATION & EDUCATIONAL RESEARCH

ISI Category Description Education & Educational Research covers resources on the full spectrum of education, from theoretical to applied, from nursery school to Ph.D. Included in this category are resources on pedagogy and methodology as well as on the history of education, reading, curriculum studies, education policy, and the sociology and economics of education, as well as the use of computers in the classroom.

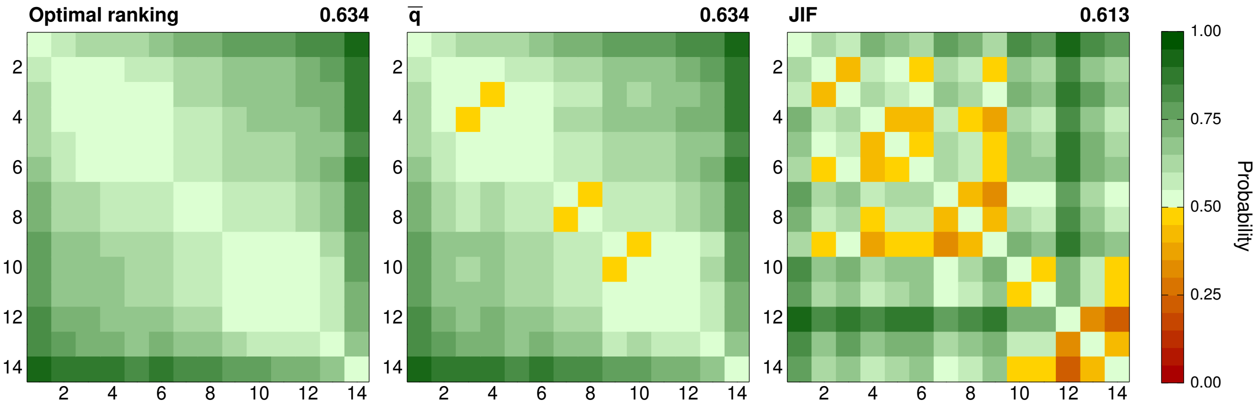

The color of each cell represents the probability of picking a paper with more citations from the higher-ranked journal. Green cells indicate adequate ranking, whereas red cells indicate that the ranking is inadequate (the probability is less than $\frac{1}{2}$). The value of M, the multi-class AUC statistic for each ranking scheme, is above each matrix.

Rank			$\mathbf{p_{ss}(q\|J)}$		n			Steady-state
**AUC**	**JIF**	**Journal abbreviation**	$\bar{q}$	σ	$\bar{n}$	**Q2**	**JIF**	**period**
1	1	J RES SCI TEACH	0.96	0.36	10.6	7	1.022	1965–2001
2	3	ELEM SCHOOL J	0.81	0.43	9.3	5	0.591	1981–2001
3	9	INT J SCI EDUC	0.75	0.38	6.2	4	0.415	1986–2000
4	6	J HIGH EDUC	0.75	0.48	7.5	4	0.500	1974–2000
5	2	J SCHOOL HEALTH	0.71	0.50	7.8	4	0.856	1983–1999
6	5	J COLL STUDENT DEV	0.68	0.42	6.0	3	0.537	1978–2000
7	8	J EDUC RES	0.57	0.41	4.5	2	0.444	1955–2001
8	4	J TEACH EDUC	0.59	0.39	3.7	2	0.563	1994–2002
9	7	J EXP EDUC	0.45	0.44	3.8	2	0.484	1956–2002
10	14	YOUNG CHILDREN	0.45	0.43	2.9	2	0.091	1976–1997
11	11	PHI DELTA KAPPAN	0.39	0.49	3.6	1	0.241	1983–1995
12	10	READ TEACH	0.36	0.47	3.1	1	0.368	1958–2000
13	13	EDUC LEADERSHIP	0.23	0.50	2.9	1	0.210	1980–1994
14	12	Z PADAGOGIK	-0.16	0.37	0.5	0	0.213	1997–2005

ELECTROCHEMISTRY

ISI Category Description Electrochemistry covers resources that deal with the chemical changes produced by electricity and the generation of electricity by chemical reactions. Applications include dry cells, lead plate, storage batteries, electroplating, electrodeposition (electrolysis), purification of copper, production of aluminum, fuel cells, and corrosion of metals.

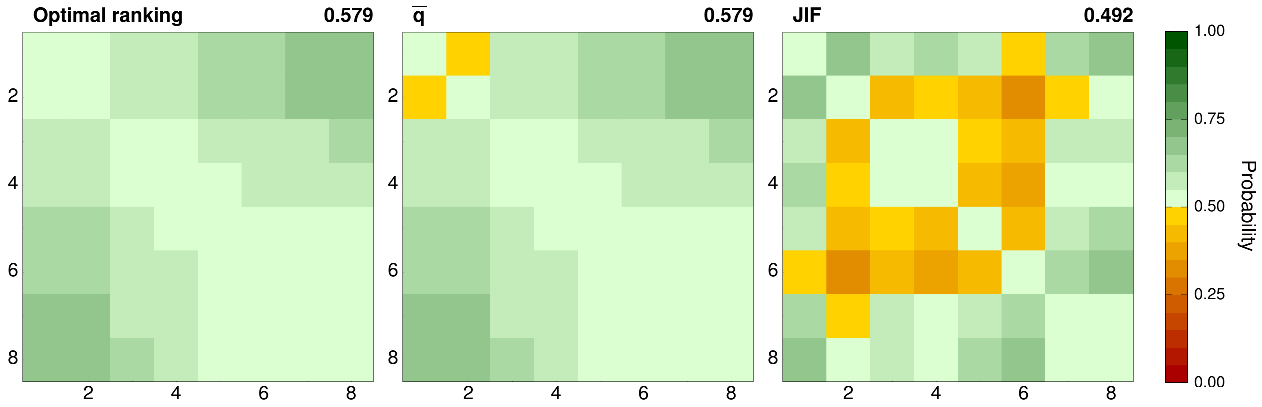

The color of each cell represents the probability of picking a paper with more citations from the higher-ranked journal. Green cells indicate adequate ranking, whereas red cells indicate that the ranking is inadequate (the probability is less than $\frac{1}{2}$). The value of M, the multi-class AUC statistic for each ranking scheme, is above each matrix.

Rank			$\mathbf{p_{ss}(q\|J)}$		n			Steady-state
**AUC**	**JIF**	**Journal abbreviation**	$\overline{\mathbf{q}}$	σ	$\overline{\mathbf{n}}$	**Q2**	**JIF**	**period**
1	6	J ELECTROANAL CHEM	1.25	0.40	25.8	15	2.339	1963–1991
2	1	BIOSENS BIOELECTRON	1.25	0.41	21.1	15	4.132	1990–2000
3	5	J ELECTROCHEM SOC	1.13	0.41	20.5	11	2.387	1985–1998
4	3	ELECTROCHIM ACTA	1.10	0.40	17.4	10	2.955	1966–1996
5	4	ELECTROANAL	1.04	0.37	12.4	9	2.444	1989–1999
6	7	SENSOR ACTUAT B-CHEM	1.02	0.38	12.2	8	2.331	1995–2001
7	2	J POWER SOURCES	0.99	0.39	12.8	8	3.521	1996–2002
8	8	J APPL ELECTROCHEM	0.96	0.38	12.0	7	1.409	1977–1997

EMERGENCY MEDICINE

ISI Category Description Emergency Medicine covers resources on the science, education, and clinical practice of emergency medicine. Coverage spans the breadth of the specialty on trauma, pediatrics, toxicology, injury prevention and control, resuscitation, and emergency medical services.

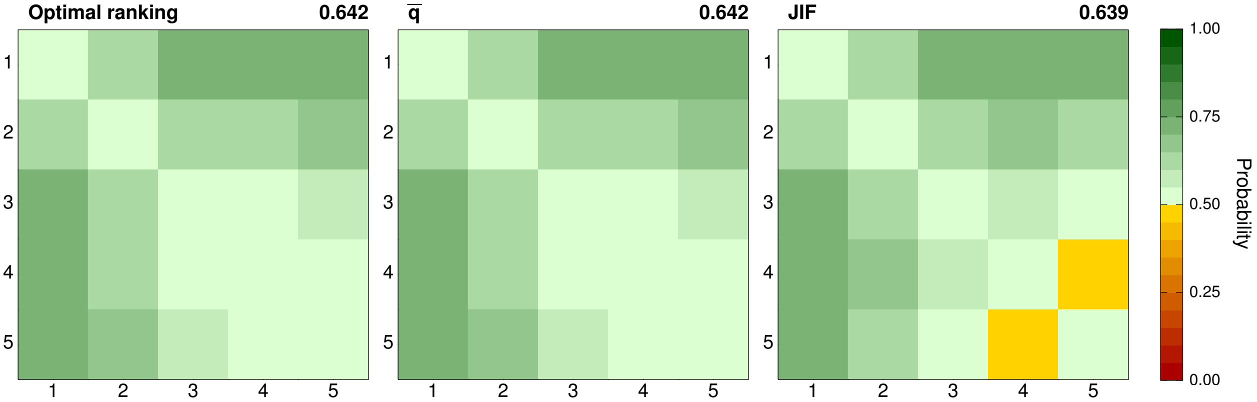

The color of each cell represents the probability of picking a paper with more citations from the higher-ranked journal. Green cells indicate adequate ranking, whereas red cells indicate that the ranking is inadequate (the probability is less than $\frac{1}{2}$). The value of M, the multi-class AUC statistic for each ranking scheme, is above each matrix.

**Rank**			$\mathbf{p_{ss}(q\|J)}$		**n**			**Steady-state**
**AUC**	**JIF**	**Journal abbreviation**	$\overline{\mathbf{q}}$	σ	$\overline{\mathbf{n}}$	**Q2**	**JIF**	**period**
1	1	ANN EMERG MED	1.08	0.42	15.5	10	3.120	1988–1999
2	2	AM J EMERG MED	0.90	0.41	10.3	6	1.518	1984–1996
3	3	INJURY	0.72	0.43	6.5	4	1.067	1973–1996
4	5	UNFALLCHIRURG	0.67	0.38	5.5	3	0.645	1988–1998
5	4	PEDIATR EMERG CARE	0.66	0.47	4.9	3	0.700	1991–2001

ENDOCRINOLOGY & METABOLISM

ISI Category Description Endocrinology & Metabolism includes resources focused on endocrine glands; the regulation of cell, organ, and system function by the action of secreted hormones; the generation and chemical/biological properties of these substances; and the pathogenesis and treatment of disorders associated with either source or target organs. Specific areas covered include neuroendocrinology, reproductive endocrinology, pancreatic hormones and diabetes, regulation of bone formation and loss, and control of growth.

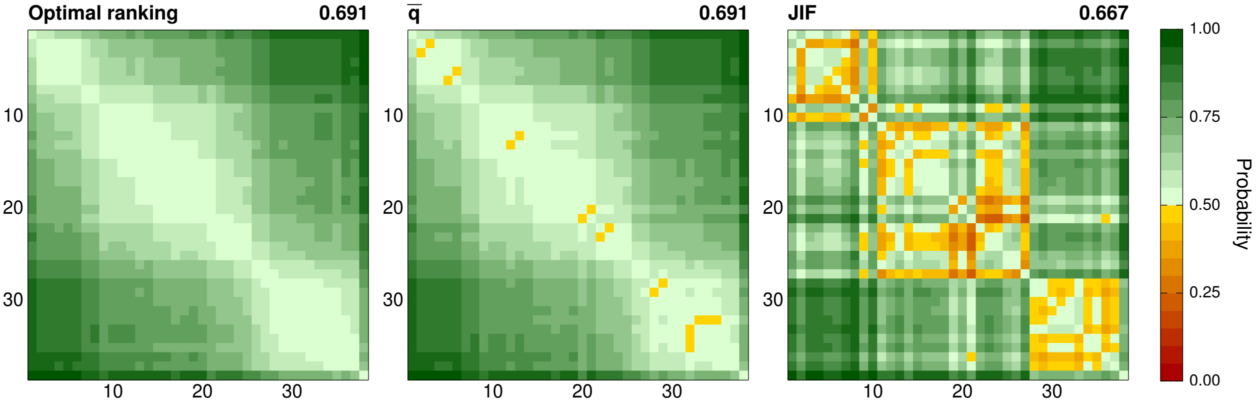

The color of each cell represents the probability of picking a paper with more citations from the higher-ranked journal. Green cells indicate adequate ranking, whereas red cells indicate that the ranking is inadequate (the probability is less than $\frac{1}{2}$). The value of M, the multi-class AUC statistic for each ranking scheme, is above each matrix.

Rank			$p_{ss}(q\|J)$		n			Steady-state
**AUC**	**JIF**	**Journal abbreviation**	$\overline{q}$	σ	$\overline{n}$	**Q2**	**JIF**	**period**
1	8	MOL ENDOCRINOL	1.70	0.35	64.5	47	4.967	1986–1994
2	10	J CEREBR BLOOD F MET	1.59	0.38	56.6	35	4.843	1982–1996
3	1	DIABETES	1.60	0.38	55.5	35	7.955	1967–1999
4	3	J BONE MINER RES	1.56	0.37	49.3	31	6.635	1985–1998
5	7	ENDOCRINOLOGY	1.55	0.35	45.3	31	5.236	1980–1993
6	4	J CLIN ENDOCR METAB	1.55	0.36	45.4	31	5.799	1994–1997
7	6	DIABETOLOGIA	1.47	0.38	41.6	26	5.247	1969–1999
8	5	FREE RADICAL BIO MED	1.46	0.36	37.4	24	5.440	1986–1997
9	2	DIABETES CARE	1.39	0.46	34.3	21	7.912	1978–2001
10	27	CALCIFIED TISSUE INT	1.37	0.38	31.7	21	2.483	1978–1994
11	13	BONE	1.36	0.38	29.9	20	3.829	1984–1998
12	23	NEUROENDOCRINOLOGY	1.33	0.34	27.6	18	2.680	1989–1995
13	24	METABOLISM	1.33	0.40	31.0	18	2.497	1955–1992
14	15	CLIN ENDOCRINOL	1.33	0.37	27.5	18	3.358	1973–1996
15	9	PSYCHONEUROENDOCRINO	1.30	0.36	25.2	17	4.850	1974–2001
16	16	J ENDOCRINOL	1.29	0.35	24.8	17	3.072	1980–1992
17	12	INT J OBESITY	1.28	0.39	25.0	16	4.055	1985–1999
18	17	J MOL ENDOCRINOL	1.27	0.33	22.1	16	2.988	1987–1995
19	22	J NEUROENDOCRINOL	1.25	0.30	22.2	16	2.774	1994–1999
20	18	MOL CELL ENDOCRINOL	1.25	0.33	21.4	15	2.918	1982–1997
21	14	PROSTATE	1.25	0.45	23.7	15	3.724	1986–2000
22	20	J STEROID BIOCHEM	1.17	0.36	19.5	12	2.825	1972–2001

table continues on next page . . .

. . . table continued from previous page

Rank			$p_{ss}(q\|J)$		n			Steady-state
**AUC**	**JIF**	**Journal abbreviation**	$\overline{q}$	σ	$\overline{n}$	**Q2**	**JIF**	**period**
23	25	GEN COMP ENDOCR	1.18	0.34	16.6	12	2.487	1992–1999
24	11	J PINEAL RES	1.14	0.36	17.3	12	4.228	1983–2002
25	26	DIABETIC MED	1.13	0.40	18.1	11	2.484	1984–2000
26	19	STEROIDS	1.02	0.35	13.5	8	2.849	1986–2001
27	36	HORM RES	1.00	0.39	12.8	8	1.385	1973–1998
28	21	NEUROPEPTIDES	0.92	0.34	9.7	7	2.789	1993–2002
29	34	J INHERIT METAB DIS	0.92	0.39	10.5	6	1.574	1980–1998
30	37	BIOL TRACE ELEM RES	0.91	0.37	10.1	6	1.007	1979–1998
31	31	COMP BIOCHEM PHYS C	0.89	0.37	9.5	6	1.991	1985–2002
32	30	HORM METAB RES	0.88	0.37	9.5	6	1.997	1980–2002
33	32	DIABETES RES CLIN PR	0.87	0.34	8.9	6	1.837	1990–2002
34	35	J ENDOCRINOL INVEST	0.85	0.36	8.6	5	1.469	1977–2001
35	28	REGUL PEPTIDES	0.88	0.57	10.4	5	2.442	1994–1998
36	29	PROSTAG LEUKOTR ESS	0.80	0.34	7.1	5	2.261	2000–2003
37	33	DIABETES METAB	0.80	0.42	8.2	5	1.742	1975–2000
38	38	ANN ENDOCRINOL-PARIS	0.46	0.43	3.6	2	0.388	1971–2000

ENERGY & FUELS

ISI Category Description Energy & Fuels covers resources on the development, production, use, application, conversion, and management of nonrenewable (combustible) fuels (such as wood, coal, petroleum, and gas) and renewable energy sources (solar, wind, biomass, geothermal, hydroelectric). Note: Resources dealing with nuclear energy and nuclear technology appear in the NUCLEAR SCIENCE & TECHNOLOGY category.

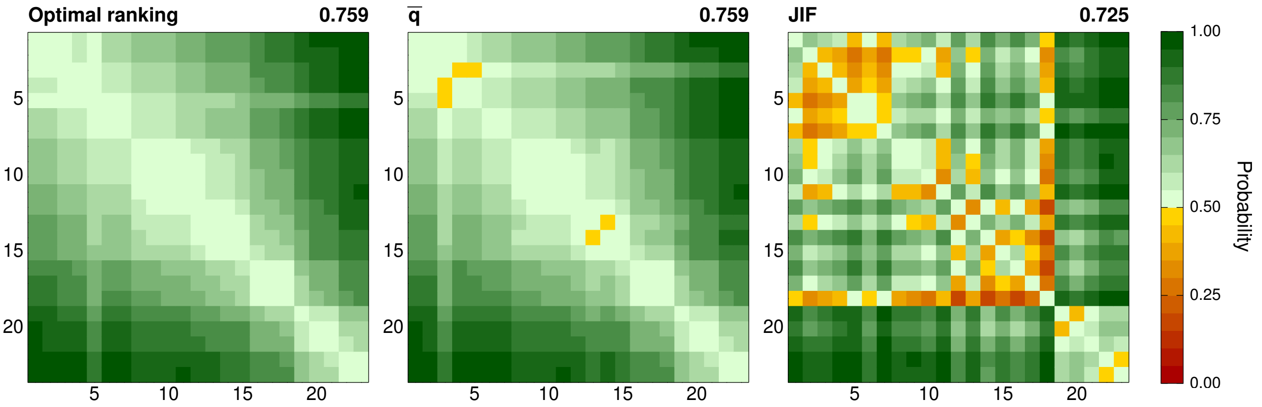

The color of each cell represents the probability of picking a paper with more citations from the higher-ranked journal. Green cells indicate adequate ranking, whereas red cells indicate that the ranking is inadequate (the probability is less than $\frac{1}{2}$). The value of M, the multi-class AUC statistic for each ranking scheme, is above each matrix.

Rank			$p_{ss}(q\|J)$		n			Steady-state
**AUC**	**JIF**	**Journal abbreviation**	$\overline{q}$	σ	$\overline{n}$	**Q2**	**JIF**	**period**
1	7	ENERG FUEL	1.09	0.37	16.0	10	1.519	1986–1993
2	5	COMBUST FLAME	1.09	0.40	16.2	10	1.828	1957–1999
3	18	COMBUST SCI TECHNOL	1.04	0.42	15.0	9	0.651	1969–1992
4	1	J POWER SOURCES	0.99	0.39	12.8	8	3.521	1996–2002
5	6	AAPG BULL	1.04	0.68	21.0	8	1.553	1973–1994
6	4	BIORESOURCE TECHNOL	0.90	0.37	9.9	6	2.180	1991–2001
7	11	FUEL	0.90	0.38	9.5	6	1.358	1989–1998
8	3	SOL ENERG MAT SOL C	0.81	0.42	8.4	5	2.321	1991–2000
9	8	BIOMASS BIOENERG	0.80	0.39	7.0	5	1.483	1990–2002
10	13	FUEL PROCESS TECHNOL	0.77	0.41	7.5	4	1.323	1976–2001
11	9	SOL ENERGY	0.74	0.34	6.1	4	1.431	1995–2000
12	2	INT J HYDROGEN ENERG	0.70	0.42	6.7	4	2.612	1975–2004
13	10	ENERG POLICY	0.64	0.38	5.2	3	1.362	1993–2001
14	17	IEEE T ENERGY CONVER	0.65	0.46	5.1	3	0.716	1985–1998
15	15	ENERGY	0.58	0.43	4.7	2	0.935	1991–2001
16	12	ENERG CONVERS MANAGE	0.40	0.45	3.1	1	1.325	1987–2006
17	16	INT J ENERG RES	0.35	0.41	2.6	1	0.718	1976–2002
18	14	APPL ENERG	0.32	0.42	2.2	1	1.006	1976–2004
19	20	J CAN PETROL TECHNOL	0.03	0.52	1.6	0	0.273	1968–2003
20	19	COMBUST EXPLO SHOCK+	-0.16	0.46	0.7	0	0.358	1972–2005
21	21	HYDROCARB PROCESS	-0.25	0.52	0.8	0	0.145	1983–2005
22	23	OIL GAS J	-0.69	0.47	0.1	0	0.034	1999–2006
23	22	CHEM TECH FUELS OIL+	-0.79	0.43	0.0	0	0.131	1976–2005

ENGINEERING, AEROSPACE

ISI Category Description Engineering, Aerospace includes resources concerned with astronautics, aeronautics, aerospace, and aviation. Topics covered include the design and construction of aircraft, space vehicles, missiles, satellites, instrumentation, and power units, as well as the launch, flight, and guidance of crafts in the earth's atmosphere or in space. Resources in this category draw from many fields, including mechanics and mechanical engineering, automation, instrumentation, and materials science.

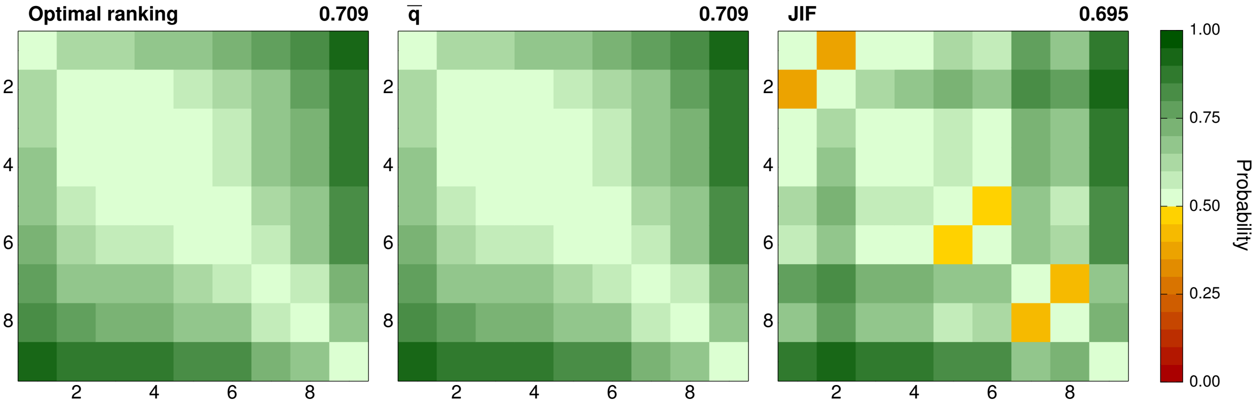

The color of each cell represents the probability of picking a paper with more citations from the higher-ranked journal. Green cells indicate adequate ranking, whereas red cells indicate that the ranking is inadequate (the probability is less than $\frac{1}{2}$). The value of M, the multi-class AUC statistic for each ranking scheme, is above each matrix.

**Rank**			$\mathbf{p_{ss}(q\|J)}$		**n**			**Steady-state**
**AUC**	**JIF**	**Journal abbreviation**	$\overline{\mathbf{q}}$	σ	$\overline{\mathbf{n}}$	**Q2**	**JIF**	**period**
1	2	AIAA J	0.88	0.43	11.6	6	0.970	1968–1993
2	1	J GUID CONTROL DYNAM	0.62	0.39	5.0	3	0.986	1992–2000
3	3	IEEE T AERO ELEC SYS	0.59	0.47	6.8	2	0.836	1971–1999
4	4	J PROPUL POWER	0.56	0.41	4.5	2	0.674	1984–1998
5	6	J AIRCRAFT	0.51	0.44	3.8	2	0.456	1988–1997
6	5	J SPACECRAFT ROCKETS	0.42	0.41	3.2	2	0.546	1964–2001
7	8	AERONAUT J	0.24	0.50	2.1	1	0.267	1983–1998
8	7	ACTA ASTRONAUT	0.08	0.49	1.5	0	0.314	1982–2001
9	9	AEROSPACE AM	-0.63	0.51	0.2	0	0.064	1983–2005

ENGINEERING, BIOMEDICAL

ISI Category Description Engineering, Biomedical covers resources that apply engineering technology to solving medical problems. Resources in this category span a wide range of applications including applied biomechanics, biorheology, medical imaging, medical monitoring equipment, artificial organs, and implanted materials and devices.

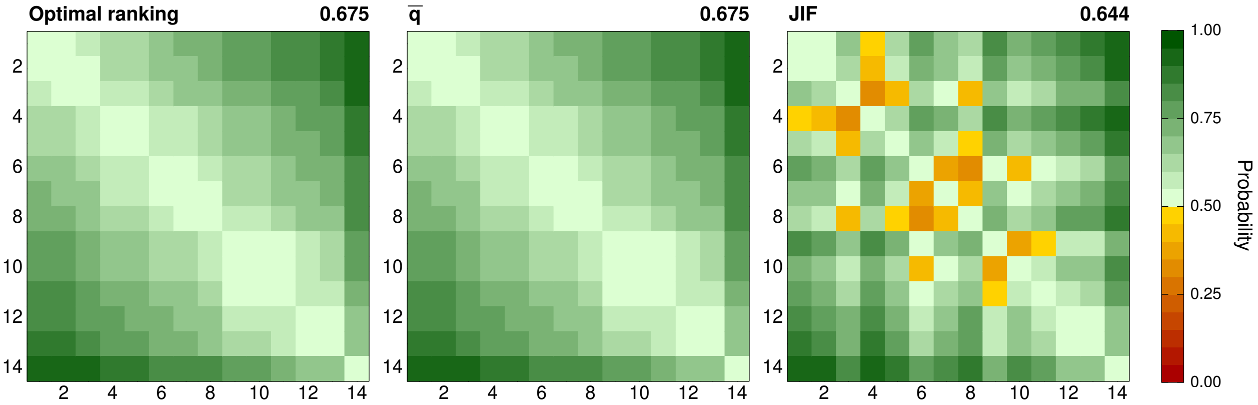

The color of each cell represents the probability of picking a paper with more citations from the higher-ranked journal. Green cells indicate adequate ranking, whereas red cells indicate that the ranking is inadequate (the probability is less than $\frac{1}{2}$). The value of M, the multi-class AUC statistic for each ranking scheme, is above each matrix.

Rank			$\mathbf{p_{ss}(q\|J)}$		n			Steady-state
**AUC**	**JIF**	**Journal abbreviation**	$\overline{\mathbf{q}}$	σ	$\overline{\mathbf{n}}$	**Q2**	**JIF**	**period**
1	4	J BIOMECH	1.38	0.43	32.6	20	2.542	1968–1995
2	1	BIOMATERIALS	1.31	0.35	25.7	18	5.196	1991–2000
3	2	IEEE T MED IMAGING	1.25	0.40	27.5	15	3.757	1985–1999
4	8	J BIOMECH ENG-T ASME	1.14	0.40	20.2	11	1.309	1978–1999
5	5	IEEE T BIO-MED ENG	1.14	0.43	20.0	11	2.302	1965–1997
6	3	PHYS MED BIOL	1.04	0.41	16.1	9	2.873	1955–2000
7	7	J MATER SCI-MATER M	1.01	0.41	14.1	8	1.562	1990–1999
8	10	PACE	0.96	0.41	12.3	7	1.095	1978–1995
9	6	ARTIF ORGANS	0.83	0.41	8.3	5	1.903	1978–1999
10	11	MED BIOL ENG COMPUT	0.82	0.42	9.2	5	1.018	1976–1999
11	9	INT J ARTIF ORGANS	0.74	0.40	7.0	4	1.253	1977–2001
12	12	IEEE ENG MED BIOL	0.67	0.53	6.7	3	0.940	1995–1999
13	13	COMPUT METH PROG BIO	0.61	0.40	6.4	3	0.624	1984–2002
14	14	DIALYSIS TRANSPLANT	0.24	0.51	2.6	1	0.248	1977–2002

ENGINEERING, CHEMICAL

ISI Category Description Engineering, Chemical covers resources that discuss the chemical conversion of raw materials into a variety of products. This category includes resources that deal with the design and operation of efficient and cost-effective plants and equipment for the production of the various end products.

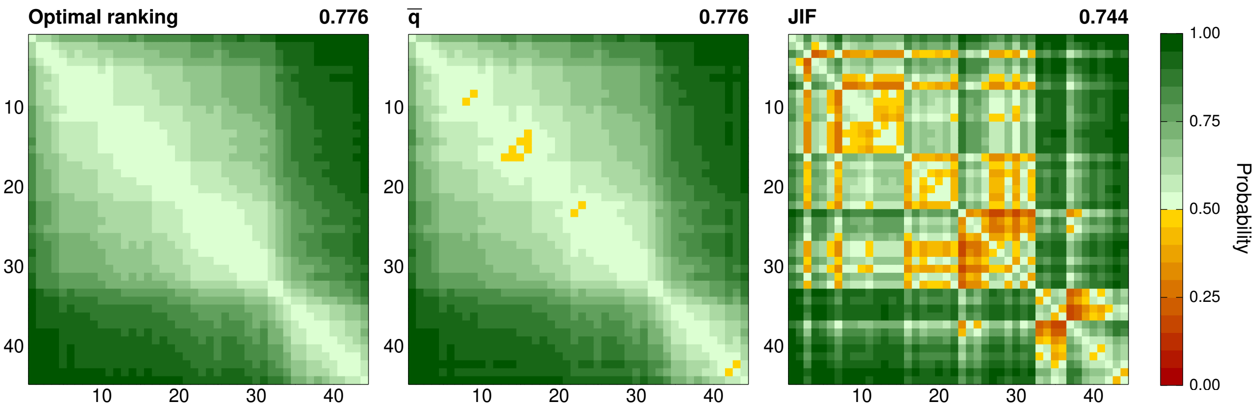

The color of each cell represents the probability of picking a paper with more citations from the higher-ranked journal. Green cells indicate adequate ranking, whereas red cells indicate that the ranking is inadequate (the probability is less than $\frac{1}{2}$). The value of M, the multi-class AUC statistic for each ranking scheme, is above each matrix.

Rank			$p_{ss}(q\|J)$		n			Steady-state
AUC	JIF	Journal abbreviation	$\overline{q}$	σ	$\overline{n}$	Q2	JIF	period
1	1	J CATAL	1.47	0.39	41.0	26	4.533	1961–1995
2	4	AICHE J	1.28	0.41	30.1	16	2.153	1954–1994
3	2	J MEMBRANE SCI	1.25	0.40	23.6	15	3.442	1975–1994
4	5	CATAL TODAY	1.19	0.39	20.5	13	2.148	1992–2000
5	15	POLYM ENG SCI	1.11	0.42	19.9	10	1.414	1968–1992
6	13	ENERG FUEL	1.09	0.37	16.0	10	1.519	1986–1993
7	8	COMBUST FLAME	1.09	0.40	16.2	10	1.828	1957–1999
8	12	CHEM ENG SCI	1.07	0.40	15.2	9	1.629	1990–1995
9	14	IND ENG CHEM RES	1.07	0.41	15.9	9	1.518	1986–1996
10	9	J FOOD ENG	1.05	0.41	12.7	9	1.696	1991–1995
11	30	COMBUST SCI TECHNOL	1.04	0.42	15.0	9	0.651	1969–1992
12	10	FLUID PHASE EQUILIBR	0.99	0.39	12.3	7	1.680	1987–1995
13	28	TRANSPORT POROUS MED	0.95	0.42	13.4	7	0.774	1986–1995
14	27	J ADHES SCI TECHNOL	0.97	0.40	11.2	7	0.868	1987–1997
15	22	J ADHESION	0.96	0.41	13.8	7	1.046	1968–1992
16	6	PROCESS BIOCHEM	0.96	0.37	10.3	7	2.008	1990–2000
17	11	J CHEM ENG DATA	0.92	0.41	12.0	6	1.642	1964–1998
18	17	FUEL	0.90	0.38	9.5	6	1.358	1989–1998
19	32	CAN J CHEM ENG	0.89	0.39	10.4	6	0.480	1986–1993
20	20	POWDER TECHNOL	0.87	0.40	10.0	5	1.232	1971–2000
21	19	J CHEM TECHNOL BIOT	0.84	0.41	9.4	5	1.276	1978–1999
22	21	DRY TECHNOL	0.78	0.40	7.5	4	1.100	1989–1992
23	29	CHEM ENG RES DES	0.79	0.45	8.1	4	0.747	1983–1999
24	18	FUEL PROCESS TECHNOL	0.77	0.41	7.5	4	1.323	1976–2001

table continues on next page . . .

. . . table continued from previous page

Rank			$\mathbf{p_{ss}(q\|J)}$		**n**			**Steady-state**
**AUC**	**JIF**	**Journal abbreviation**	$\overline{\mathbf{q}}$	σ	$\overline{\mathbf{n}}$	**Q2**	**JIF**	**period**
25	3	J AEROSOL SCI	0.77	0.50	9.6	4	2.952	1979–1999
26	26	INT J MINER PROCESS	0.75	0.37	5.9	4	0.884	1991–2002
27	31	J CHEM ENG JPN	0.72	0.37	5.7	4	0.594	1994–1999
28	7	DYES PIGMENTS	0.69	0.36	5.9	3	1.909	1980–2003
29	16	COMPUT CHEM ENG	0.68	0.43	6.2	3	1.404	1994–2002
30	37	CHEM ENG COMMUN	0.64	0.47	6.6	3	0.350	1977–1994
31	24	MINER ENG	0.63	0.37	4.6	3	0.942	1987–2000
32	25	DESALINATION	0.53	0.46	5.3	2	0.917	1975–1997
33	38	KAGAKU KOGAKU RONBUN	0.43	0.38	2.8	2	0.294	1979–1996
34	23	APPL ENERG	0.32	0.42	2.2	1	1.006	1976–2004
35	39	CHEM ENG PROG	0.16	0.43	1.9	1	0.293	1995–2002
36	34	CHEM-ING-TECH	0.14	0.44	1.5	0	0.407	1985–2002
37	41	J CAN PETROL TECHNOL	0.03	0.52	1.6	0	0.273	1968–2003
38	33	PRZEM CHEM	0.03	0.40	0.9	0	0.429	1969–2002
39	40	REV CHIM-BUCHAREST	-0.05	0.42	0.8	0	0.287	1976–2002
40	36	COMBUST EXPLO SHOCK+	-0.16	0.46	0.7	0	0.358	1972–2005
41	42	HYDROCARB PROCESS	-0.25	0.52	0.8	0	0.145	1983–2005
42	35	CHEM ENG NEWS	-0.50	0.67	0.9	0	0.379	1967–2004
43	44	CHEM ENG-NEW YORK	-0.40	0.45	0.3	0	0.102	1996–2006
44	43	CHEM TECH FUELS OIL+	-0.79	0.43	0.0	0	0.131	1976–2005

ENGINEERING, CIVIL

ISI Category Description Engineering, Civil includes resources on the planning, design, construction, and maintenance of fixed structures and ground facilities for industry, occupancy, transportation, use and control of water, and harbor facilities. Resources also may cover the sub-fields of structural engineering, geotechnics, earthquake engineering, ocean engineering, water resources and supply, marine engineering, transportation engineering, and municipal engineering.

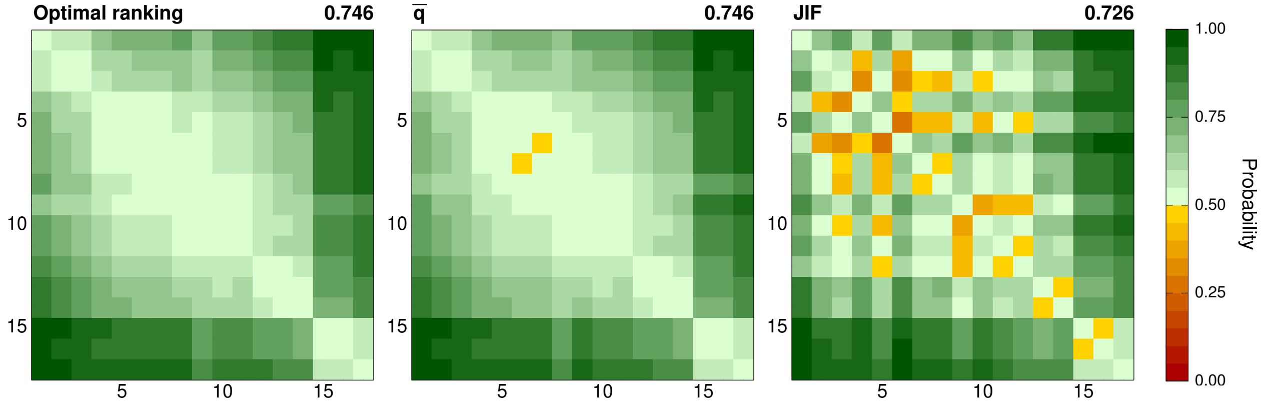

The color of each cell represents the probability of picking a paper with more citations from the higher-ranked journal. Green cells indicate adequate ranking, whereas red cells indicate that the ranking is inadequate (the probability is less than $\frac{1}{2}$). The value of M, the multi-class AUC statistic for each ranking scheme, is above each matrix.

Rank			$p_{ss}(q\|J)$		n			Steady-state
AUC	JIF	Journal abbreviation	$\overline{q}$	σ	$\overline{n}$	Q2	JIF	period
1	1	J HYDROL	1.07	0.37	13.5	10	2.117	1991–2000
2	6	J ENVIRON ENG-ASCE	0.95	0.37	11.5	7	0.725	1982–1998
3	4	J HYDRAUL ENG-ASCE	0.94	0.45	12.2	7	1.004	1982–1995
4	2	J HAZARD MATER	0.76	0.40	7.4	4	1.855	1990–2003
5	8	J STRUCT ENG-ASCE	0.73	0.41	6.9	4	0.635	1982–2000
6	7	ACI STRUCT J	0.69	0.43	6.7	4	0.657	1986–1999
7	10	J CONSTR ENG M ASCE	0.71	0.43	5.3	3	0.471	1989–1999
8	3	J IRRIG DRAIN E-ASCE	0.63	0.39	5.0	3	1.250	1982–2002
9	12	J AM WATER WORKS ASS	0.68	0.65	9.8	3	0.431	1977–1994
10	11	MATER STRUCT	0.57	0.47	5.7	2	0.459	1990–1996
11	5	COMPUT STRUCT	0.56	0.39	4.5	2	0.846	1990–2002
12	9	J WIND ENG IND AEROD	0.47	0.43	3.4	2	0.586	1994–1999
13	14	CAN J CIVIL ENG	0.38	0.43	2.7	1	0.314	1978–2000
14	13	J TRANSP ENG-ASCE	0.31	0.42	2.3	1	0.410	1983–2002
15	16	CIVIL ENG	-0.28	0.41	0.3	0	0.072	1966–2004
16	15	ITE J	-0.33	0.47	0.4	0	0.132	1977–2004
17	17	NAV ARCHIT	-0.42	0.18	0.0	0	0.001	1979–2006

ENGINEERING, ELECTRICAL & ELECTRONIC

ISI Category Description Engineering, Electrical & Electronic covers resources that deal with the applications of electricity, generally those involving current flows through conductors, as in motors and generators. This category also includes resources that cover the conduction of electricity through gases or a vacuum as well as through semi-conducting and superconducting materials. Other relevant topics in this category include image and signal processing, electromagnetics, electronic components and materials, microwave technology, and microelectronics.

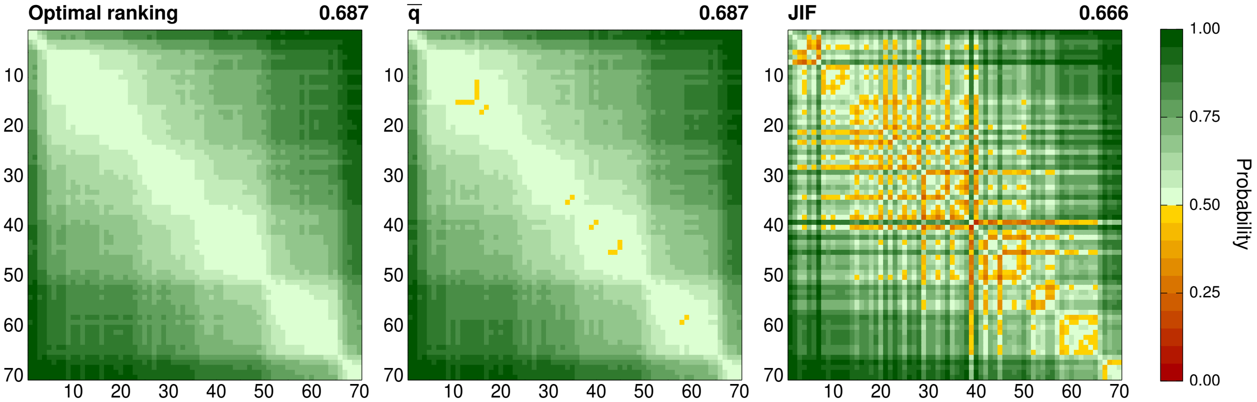

The color of each cell represents the probability of picking a paper with more citations from the higher-ranked journal. Green cells indicate adequate ranking, whereas red cells indicate that the ranking is inadequate (the probability is less than $\frac{1}{2}$). The value of M, the multi-class AUC statistic for each ranking scheme, is above each matrix.

**Rank**			$\mathbf{p_{ss}(q\|J)}$		**n**			**Steady-state**
**AUC**	**JIF**	**Journal abbreviation**	$\overline{\mathbf{q}}$	σ	$\overline{\mathbf{n}}$	**Q2**	**JIF**	**period**
1	1	IEEE T PATTERN ANAL	1.51	0.42	67.3	29	4.306	1982–1993
2	7	IEEE T NEURAL NETWOR	1.49	0.32	48.8	27	2.620	1990–1992
3	5	IEEE T AUTOMAT CONTR	1.31	0.47	40.9	18	2.772	1970–1995
4	2	IEEE T MED IMAGING	1.25	0.40	27.5	15	3.757	1985–1999
5	23	IEEE T SIGNAL PROCES	1.13	0.43	21.3	10	1.570	1990–1993
6	28	IEEE T COMPUT	1.09	0.46	23.1	10	1.426	1971–1991
7	21	J VAC SCI TECHNOL B	1.09	0.41	17.5	10	1.597	1987–1990
8	6	IEEE ELECTR DEVICE L	1.08	0.40	16.8	10	2.716	1982–1994
9	3	P IEEE	1.06	0.52	25.5	9	3.686	1962–1998
10	12	IEEE T ELECTRON DEV	1.05	0.44	18.1	9	2.052	1973–1994
11	34	IEEE T VEH TECHNOL	1.01	0.42	23.4	8	1.071	1989–1993
12	9	AUTOMATICA	1.04	0.50	23.9	9	2.273	1970–1995
13	11	IEEE T SOFTWARE ENG	1.03	0.44	17.2	8	2.132	1976–1999
14	13	IEEE T MICROW THEORY	1.03	0.44	16.6	8	2.027	1968–1992
15	40	IEEE T COMPUT AID D	1.02	0.42	14.7	8	0.838	1983–1989
16	17	IEEE J SEL AREA COMM	0.98	0.47	17.5	7	1.816	1988–2000
17	38	IEEE T CIRCUITS-II	0.98	0.39	14.8	8	0.922	1991–1995
18	4	J LIGHTWAVE TECHNOL	0.97	0.41	14.2	7	2.824	1983–2000
19	26	IEEE T ANTENN PROPAG	0.97	0.42	14.5	7	1.480	1969–1998
20	19	IEEE T ULTRASON FERR	0.97	0.42	13.4	7	1.729	1985–1998
21	10	IEEE J QUANTUM ELECT	0.95	0.39	12.3	7	2.262	1994–2002
22	8	IEEE PHOTONIC TECH L	0.93	0.37	10.5	7	2.353	1994–2000

table continues on next page . . .

. . . table continued from previous page

Rank			$p_{ss}(q\|J)$		n			Steady-state
AUC	JIF	Journal abbreviation	$\overline{q}$	σ	$\overline{n}$	Q2	JIF	period
23	16	PATTERN RECOGN	0.92	0.42	12.6	6	1.822	1967–1997
24	14	IEEE J SOLID-ST CIRC	0.88	0.43	11.9	6	2.002	1970–1999
25	30	IEEE T COMMUN	0.86	0.48	14.0	5	1.208	1972–2001
26	24	J ELECTRON MATER	0.84	0.41	9.4	5	1.504	1988–1999
27	18	IEEE T GEOSCI REMOTE	0.83	0.49	11.6	5	1.752	1964–2006
28	37	IEEE T POWER SYST	0.81	0.42	8.6	5	0.922	1985–1999
29	27	SENSOR ACTUAT A-PHYS	0.80	0.38	7.7	5	1.434	1997–2001
30	31	IMAGE VISION COMPUT	0.79	0.40	8.2	5	1.171	1984–2001
31	33	IEEE T CIRCUITS-I	0.77	0.40	8.6	4	1.139	1995–2001
32	50	IEEE T IND ELECTRON	0.76	0.43	7.7	4	0.590	1988–1998
33	22	SEMICOND SCI TECH	0.75	0.39	7.1	4	1.586	1991–1997
34	15	IEEE T INFORM THEORY	0.74	0.44	10.0	4	1.938	2000–2003
35	36	IEEE T MAGN	0.74	0.45	8.4	4	0.938	1965–1991
36	43	IEEE T RELIAB	0.70	0.47	7.1	3	0.800	1963–1995
37	20	IEEE COMMUN MAG	0.69	0.51	8.8	3	1.678	1996–2002
38	49	IEEE T IND APPL	0.66	0.51	7.4	3	0.611	1990–1997
39	25	IEEE T NUCL SCI	0.63	0.48	6.8	3	1.497	1981–1998
40	46	IEEE T ENERGY CONVER	0.65	0.46	5.1	3	0.716	1985–1998
41	32	SOLID STATE ELECTRON	0.62	0.42	5.2	3	1.159	1993–2000
42	44	IEEE T ELECTROMAGN C	0.60	0.44	6.1	3	0.770	1966–2002
43	47	SIGNAL PROCESS	0.59	0.43	6.8	2	0.669	1978–1998
44	41	IEEE T AERO ELEC SYS	0.59	0.47	6.8	2	0.836	1971–1999
45	35	ELECTRON LETT	0.59	0.49	6.3	2	1.063	1969–2006
46	55	IEEE T POWER DELIVER	0.57	0.46	5.1	2	0.496	1985–1999
47	56	J ELECTROSTAT	0.57	0.42	5.0	2	0.467	1976–2003
48	51	IEEE T INSTRUM MEAS	0.55	0.44	4.8	2	0.572	1963–2001
49	48	OPT QUANT ELECTRON	0.51	0.41	4.3	2	0.644	1992–2002
50	29	MICROELECTRON ENG	0.48	0.44	3.9	2	1.398	1992–2003
51	53	J ELECTROMAGNET WAVE	0.42	0.46	3.1	1	0.524	1995–1999
52	54	IEICE T ELECTRON	0.32	0.43	2.5	1	0.508	1991–2000
53	60	J FRANKLIN I	0.31	0.45	2.3	1	0.362	1985–2004
54	57	INT J ELECTRON	0.30	0.47	2.9	1	0.459	1964–2001
55	42	MICROELECTRON RELIAB	0.27	0.44	2.2	1	0.815	1965–2004
56	65	IEICE T COMMUN	0.25	0.44	2.3	1	0.290	1991–2000
57	52	MICROW OPT TECHN LET	0.24	0.45	2.4	1	0.568	1987–2002
58	61	IEEE T EDUC	0.20	0.41	1.7	1	0.362	1964–2003
59	64	SOLID STATE TECHNOL	0.21	0.53	2.7	1	0.293	1992–1999
60	59	ELECTR POW SYST RES	0.20	0.43	1.6	1	0.393	1976–2002
61	45	IEEE T CONSUM ELECTR	0.18	0.45	1.9	1	0.727	1974–2002
62	63	IEICE T FUND ELECTR	0.18	0.42	1.7	1	0.312	1991–2002
63	62	INT J INFRARED MILLI	0.17	0.46	1.7	1	0.326	1994–2002
64	58	COMPUT COMMUN	0.09	0.46	1.5	0	0.444	1990–2006
65	39	IEEE SPECTRUM	0.08	0.50	1.9	0	0.840	1971–2002

table continues on next page . . .

. . . table continued from previous page

Rank			$\mathbf{p_{ss}(q\|J)}$		n			Steady-state
**AUC**	**JIF**	**Journal abbreviation**	$\overline{\mathbf{q}}$	σ	$\overline{\mathbf{n}}$	**Q2**	**JIF**	**period**
66	66	MICROWAVE J	-0.09	0.51	1.0	0	0.223	1976–1998
67	68	ELECTR ENG JPN	-0.31	0.48	0.4	0	0.100	1964–2005
68	69	CONTROL ENG	-0.37	0.38	0.2	0	0.051	1966–2005
69	70	MICROWAVES RF	-0.57	0.46	0.2	0	0.045	1981–2005
70	67	EDN	-0.58	0.36	0.1	0	0.132	1976–2002

ENGINEERING, ENVIRONMENTAL

ISI Category Description Engineering, Environmental includes resources that discuss the effects of human beings on the environment and the development of controls to minimize environmental degradation. Relevant topics in this category include water and air pollution control, hazardous waste management, land reclamation, pollution prevention, bioremediation, incineration, management of sludge problems, landfill and waste repository design and construction, facility decommissioning, and environmental policy and compliance.

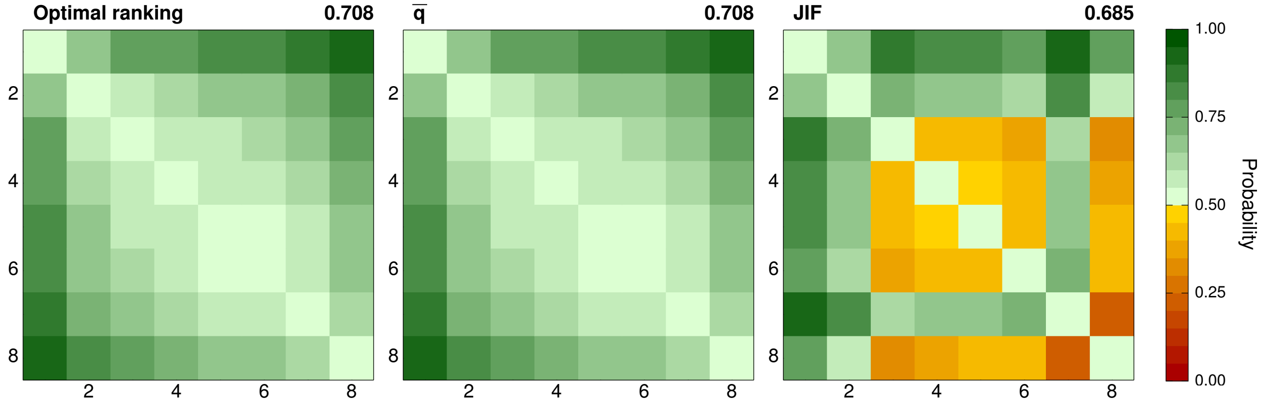

The color of each cell represents the probability of picking a paper with more citations from the higher-ranked journal. Green cells indicate adequate ranking, whereas red cells indicate that the ranking is inadequate (the probability is less than $\frac{1}{2}$). The value of M, the multi-class AUC statistic for each ranking scheme, is above each matrix.

Rank			$\mathbf{p_{ss}(q\|J)}$		n			Steady-state
**AUC**	**JIF**	**Journal abbreviation**	$\overline{\mathbf{q}}$	σ	$\overline{\mathbf{n}}$	**Q2**	**JIF**	**period**
1	1	ENVIRON SCI TECHNOL	1.48	0.40	36.2	26	4.040	1995–1997
2	2	WATER RES	1.18	0.37	20.5	13	2.459	1966–1998
3	8	WATER ENVIRON RES	1.07	0.44	14.0	9	0.665	1991–1996
4	6	J ENVIRON ENG-ASCE	0.95	0.37	11.5	7	0.725	1982–1998
5	5	AMBIO	0.88	0.48	11.2	6	1.433	1994–2002
6	4	J AIR WASTE MANAGE	0.87	0.44	11.0	5	1.441	1989–2000
7	3	J HAZARD MATER	0.76	0.40	7.4	4	1.855	1990–2003
8	7	J ENVIRON SCI HEAL A	0.57	0.41	4.6	2	0.669	1975–2000

ENGINEERING, GEOLOGICAL

ISI Category Description Engineering, Geological includes multidisciplinary resources that encompass the knowledge and experience drawn from both the geosciences and various engineering disciplines (primarily civil engineering). Resources in this category cover geotechnical engineering, geotechnics, geotechnology, soil dynamics, earthquake engineering, geotextiles and geomembranes, engineering geology, and rock mechanics.

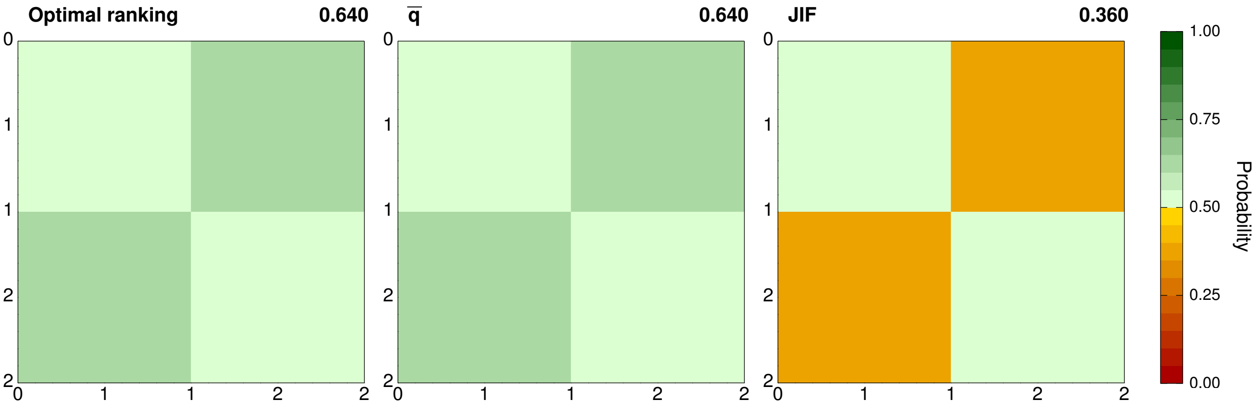

The color of each cell represents the probability of picking a paper with more citations from the higher-ranked journal. Green cells indicate adequate ranking, whereas red cells indicate that the ranking is inadequate (the probability is less than $\frac{1}{2}$). The value of M, the multi-class AUC statistic for each ranking scheme, is above each matrix.

**Rank**			$\mathbf{p_{ss}(q\|J)}$		**n**			**Steady-state**
**AUC**	**JIF**	**Journal abbreviation**	$\overline{\mathbf{q}}$	σ	$\overline{\mathbf{n}}$	**Q2**	**JIF**	**period**
1	2	CAN GEOTECH J	0.76	0.40	7.1	4	0.358	1990–1998
2	1	ENG GEOL	0.50	0.43	4.0	2	0.900	1967–2004

ENGINEERING, INDUSTRIAL

ISI Category Description Engineering, Industrial includes resources that focus on engineering systems that integrate people, materials, capital, and equipment to provide products and services. Relevant topics covered in the category include operations research, process engineering, productivity engineering, manufacturing, computer-integrated manufacturing (CIM), industrial economics, and design engineering.

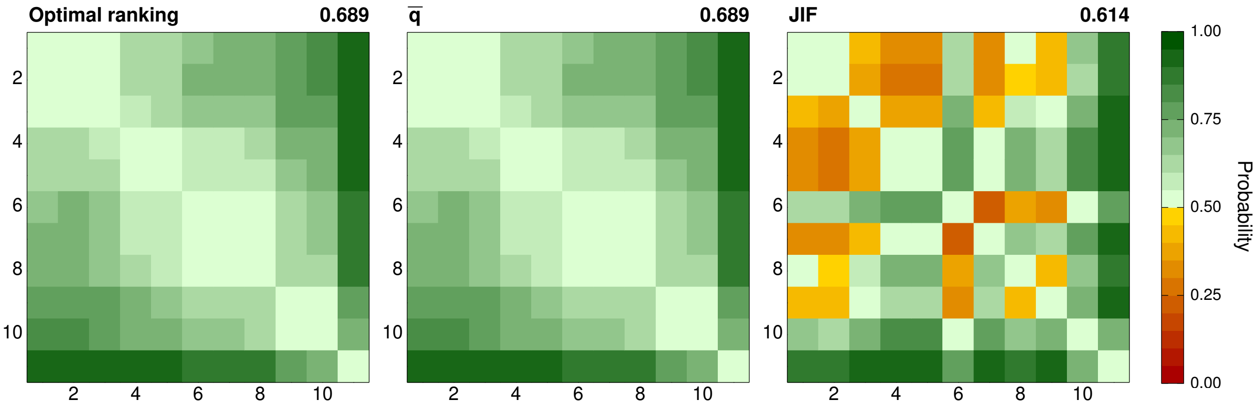

The color of each cell represents the probability of picking a paper with more citations from the higher-ranked journal. Green cells indicate adequate ranking, whereas red cells indicate that the ranking is inadequate (the probability is less than $\frac{1}{2}$). The value of M, the multi-class AUC statistic for each ranking scheme, is above each matrix.

Rank			$p_{ss}(q\|J)$		n			Steady-state
**AUC**	**JIF**	**Journal abbreviation**	$\overline{q}$	σ	$\overline{n}$	**Q2**	**JIF**	**period**
1	5	INT J PROD RES	0.96	0.49	13.4	7	0.799	1986–1992
2	4	ERGONOMICS	0.96	0.46	13.1	7	0.826	1957–1994
3	7	IIE TRANS	0.89	0.44	11.3	6	0.637	1981–1997
4	3	COMPUT OPER RES	0.72	0.42	7.1	3	0.893	1975–2000
5	9	J CONSTR ENG M ASCE	0.71	0.43	5.3	3	0.471	1989–1999
6	1	INT J PROD ECON	0.58	0.44	4.2	2	1.183	1990–2002
7	8	J MATER PROCESS TECH	0.54	0.40	3.8	2	0.615	1989–2001
8	2	RELIAB ENG SYST SAFE	0.52	0.42	4.0	2	0.920	1981–2002
9	6	COMPUT IND ENG	0.28	0.47	2.5	1	0.650	1975–2002
10	10	J SCI IND RES INDIA	0.21	0.53	2.2	1	0.178	1960–1998
11	11	R&D MAG	-0.54	0.35	0.1	0	0.041	1990–2005

ENGINEERING, MANUFACTURING

ISI Category Description Engineering, Manufacturing covers resources on the conversion of raw materials into end-use products or processed materials. Topics in this category include computer-integrated manufacturing (CIM), computer-aided design (CAD), and computer-aided manufacturing (CAM); design of products, tools, and machines; quality control; scheduling; production; and inventory control.

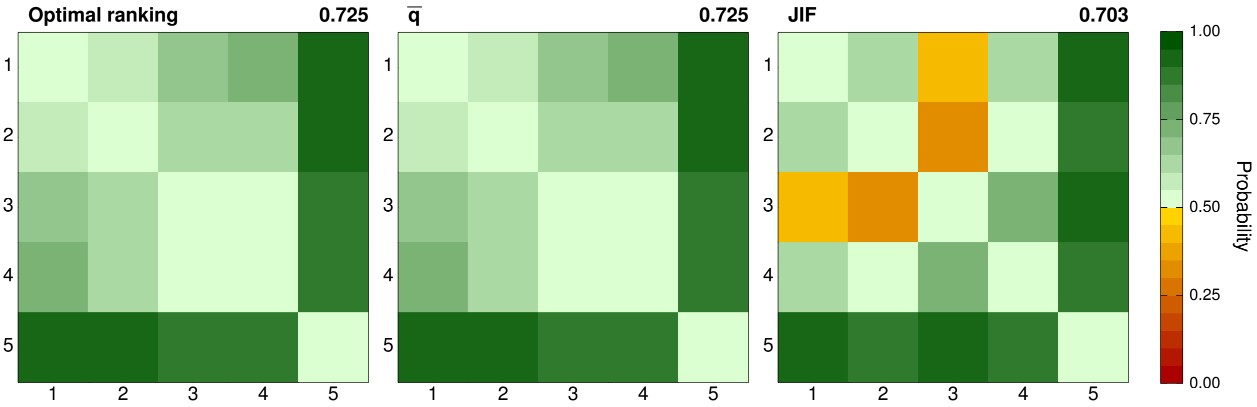

The color of each cell represents the probability of picking a paper with more citations from the higher-ranked journal. Green cells indicate adequate ranking, whereas red cells indicate that the ranking is inadequate (the probability is less than $\frac{1}{2}$). The value of M, the multi-class AUC statistic for each ranking scheme, is above each matrix.

Rank			$\mathbf{p_{ss}(q\|J)}$		n			Steady-state
**AUC**	**JIF**	**Journal abbreviation**	$\overline{\mathbf{q}}$	σ	$\overline{\mathbf{n}}$	**Q2**	**JIF**	**period**
1	3	INT J PROD RES	0.96	0.49	13.4	7	0.799	1986–1992
2	1	INT J MACH TOOL MANU	0.80	0.46	7.5	5	1.184	1989–1996
3	2	INT J PROD ECON	0.58	0.44	4.2	2	1.183	1990–2002
4	4	J MATER PROCESS TECH	0.54	0.40	3.8	2	0.615	1989–2001
5	5	MANUF ENG	-0.52	0.47	0.2	0	0.067	1981–2003

ENGINEERING, MECHANICAL

ISI Category Description Engineering, Mechanical includes resources on the generation, transmission, and use of heat and mechanical power, as well as with the production and operation of tools, machinery, and their products. Topics in this category include heat transfer and thermodynamics, fatigue and fracture, wear, tribology, energy conversion, hydraulics, pneumatics, microelectronics, plasticity, strain analysis, and aerosol technology.

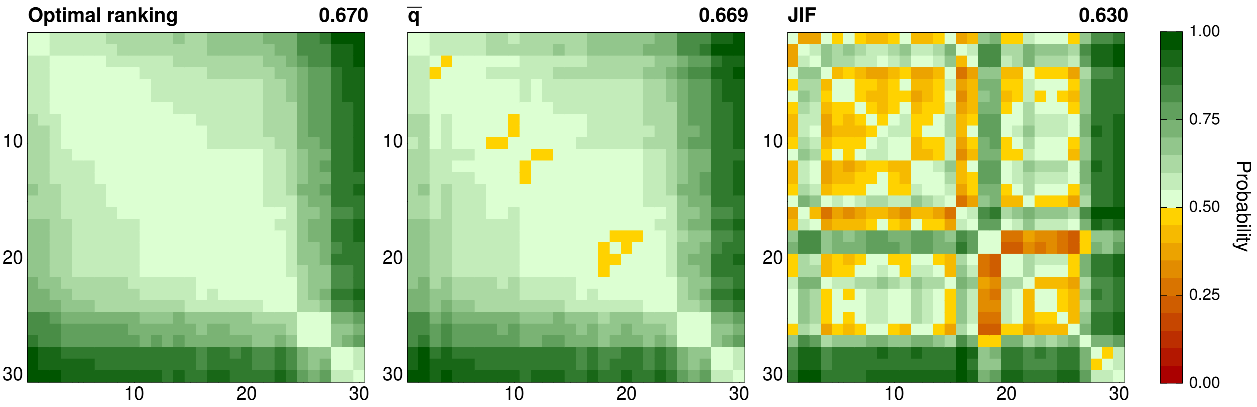

The color of each cell represents the probability of picking a paper with more citations from the higher-ranked journal. Green cells indicate adequate ranking, whereas red cells indicate that the ranking is inadequate (the probability is less than $\frac{1}{2}$). The value of M, the multi-class AUC statistic for each ranking scheme, is above each matrix.

Rank			$p_{ss}(q\|J)$		n			Steady-state
AUC	JIF	Journal abbreviation	$\overline{q}$	σ	$\overline{n}$	Q2	JIF	period
1	16	J HEAT TRANS-T ASME	1.02	0.37	13.1	8	0.886	1978–1994
2	2	AEROSOL SCI TECH	1.03	0.41	15.1	8	2.905	1981–2000
3	3	INT J HEAT MASS TRAN	0.93	0.34	10.4	7	1.482	1991–1997
4	12	J HYDRAUL ENG-ASCE	0.94	0.45	12.2	7	1.004	1982–1995
5	9	EXP FLUIDS	0.88	0.42	10.8	6	1.112	1983–1994
6	17	J SOUND VIB	0.87	0.43	10.7	6	0.884	1966–1994
7	13	J ENG MECH-ASCE	0.87	0.43	11.0	6	0.965	1982–1995
8	8	WEAR	0.86	0.43	10.3	5	1.180	1965–1996
9	26	J TURBOMACH	0.86	0.41	8.2	6	0.478	1989–1995
10	20	J TRIBOL-T ASME	0.86	0.47	9.4	5	0.810	1988–1994
11	14	INT J MECH SCI	0.80	0.45	9.4	4	0.950	1968–1997
12	10	DRY TECHNOL	0.78	0.40	7.5	4	1.100	1989–1992
13	7	INT J MACH TOOL MANU	0.80	0.46	7.5	5	1.184	1989–1996
14	21	FATIGUE FRACT ENG M	0.78	0.38	7.3	4	0.756	1985–1999
15	5	J ENG MATER-T ASME	0.77	0.45	10.5	4	1.239	1976–1999
16	1	J AEROSOL SCI	0.77	0.50	9.6	4	2.952	1979–1999
17	11	INT J FATIGUE	0.75	0.39	6.4	4	1.020	1982–2001
18	25	TRIBOL T	0.71	0.40	6.6	4	0.507	1989–1997
19	23	J FLUID ENG-T ASME	0.71	0.44	7.3	4	0.678	1982–1997
20	6	INT J IMPACT ENG	0.70	0.43	6.9	3	1.195	1987–2000
21	24	J VIB ACOUST	0.73	0.49	7.0	4	0.565	1984–1997
22	4	J MECH DESIGN	0.69	0.45	6.1	3	1.252	1989–1998
23	15	EXP THERM FLUID SCI	0.65	0.36	5.9	3	0.894	1987–2000

table continues on next page . . .

. . . table continued from previous page

Rank			$p_{ss}(q\|J)$		n			Steady-state
**AUC**	**JIF**	**Journal abbreviation**	$\overline{q}$	σ	$\overline{n}$	**Q2**	**JIF**	**period**
24	22	MECH MACH THEORY	0.58	0.39	4.9	3	0.750	1971–2001
25	27	J ENG GAS TURB POWER	0.40	0.46	3.4	1	0.304	1985–2001
26	18	J PRESS VESS-T ASME	0.31	0.47	2.8	1	0.843	1976–2000
27	19	INT J PRES VES PIP	0.29	0.42	2.2	1	0.830	1980–2003
28	29	MECH ENG	-0.20	0.46	0.6	0	0.107	1965–1996
29	28	ASHRAE J	-0.21	0.46	0.6	0	0.297	1971–2004
30	30	J JPN SOC TRIBOLOGIS	-0.44	0.47	0.2	0	0.012	1988–2004

ENGINEERING, MULTIDISCIPLINARY

ISI Category Description Engineering, Multidisciplinary covers resources having a general or interdisciplinary approach to engineering. Relevant topics include computer science and mathematics in engineering, engineering education, reliability studies, and audio engineering.

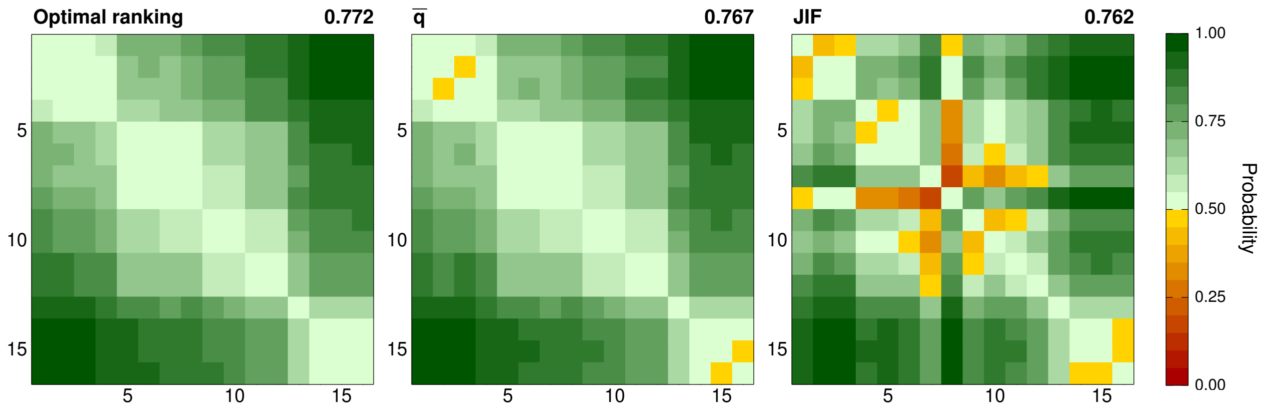

The color of each cell represents the probability of picking a paper with more citations from the higher-ranked journal. Green cells indicate adequate ranking, whereas red cells indicate that the ranking is inadequate (the probability is less than $\frac{1}{2}$). The value of M, the multi-class AUC statistic for each ranking scheme, is above each matrix.

Rank			$p_{ss}(q\|J)$		n			Steady-state
AUC	JIF	Journal abbreviation	$\bar{q}$	σ	$\bar{n}$	Q2	JIF	period
1	2	COMBUST FLAME	1.09	0.40	16.2	10	1.828	1957–1999
2	3	INT J NUMER METH ENG	1.03	0.39	16.3	9	1.497	1990–1996
3	8	COMBUST SCI TECHNOL	1.04	0.42	15.0	9	0.651	1969–1992
4	1	COMPUT METHOD APPL M	0.97	0.47	17.8	7	2.015	1976–1998
5	5	INT J ENG SCI	0.69	0.44	7.7	3	1.060	1972–2000
6	4	MEAS SCI TECHNOL	0.68	0.42	6.5	3	1.228	1989–2000
7	10	IEEE T IND APPL	0.66	0.51	7.4	3	0.611	1990–1997
8	6	ENG ANAL BOUND ELEM	0.62	0.40	5.1	3	0.883	1984–2002
9	11	APPL MATH MODEL	0.47	0.43	4.0	2	0.583	1977–2002
10	9	J AUDIO ENG SOC	0.46	0.45	4.3	2	0.639	1964–2003
11	12	J FRANKLIN I	0.31	0.45	2.3	1	0.362	1985–2004
12	7	INT J PRES VES PIP	0.29	0.42	2.2	1	0.830	1980–2003
13	13	COMBUST EXPLO SHOCK+	-0.16	0.46	0.7	0	0.358	1972–2005
14	16	INTECH	-0.45	0.34	0.1	0	0.031	1985–2001
15	14	INSTRUM EXP TECH+	-0.70	0.53	0.1	0	0.329	1963–2005
16	15	MEAS TECH+	-0.70	0.38	0.0	0	0.129	1971–2003

ENGINEERING, OCEAN

ISI Category Description Engineering, Ocean includes resources concerned with the development of equipment and techniques that allow humans to operate successfully beneath and on the surface of the ocean in order to develop and utilize marine resources.

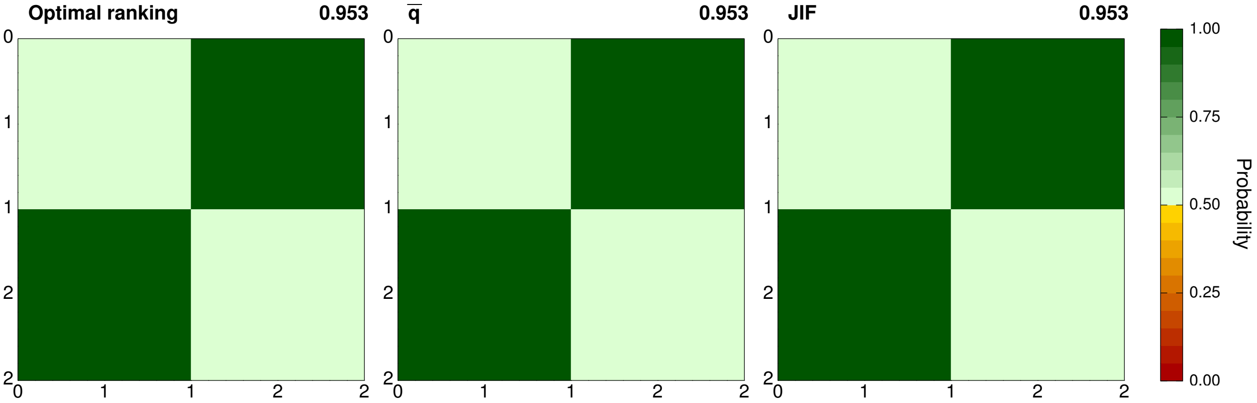

The color of each cell represents the probability of picking a paper with more citations from the higher-ranked journal. Green cells indicate adequate ranking, whereas red cells indicate that the ranking is inadequate (the probability is less than $\frac{1}{2}$). The value of M, the multi-class AUC statistic for each ranking scheme, is above each matrix.

**Rank**			$\mathbf{p_{ss}(q\|J)}$		**n**			**Steady-state**
**AUC**	**JIF**	**Journal abbreviation**	$\overline{\mathbf{q}}$	σ	$\overline{\mathbf{n}}$	**Q2**	**JIF**	**period**
1	1	J ATMOS OCEAN TECH	1.05	0.42	15.8	9	1.298	1988–1996
2	2	SEA TECHNOL	-0.46	0.51	0.4	0	0.096	1976–2006

ENGINEERING, PETROLEUM

ISI Category Description Engineering, Petroleum covers resources that report on a combination of engineering concepts, methods, and techniques on drilling and extracting hydrocarbons and other fluids from the earth (e.g., chemical flooding, thermal flooding, miscible displacement techniques, and horizontal drilling) and on the refining process. Relevant topics in this category include drilling engineering, production engineering, reservoir engineering, and formation evaluation, which infers reservoir properties through indirect measurements.

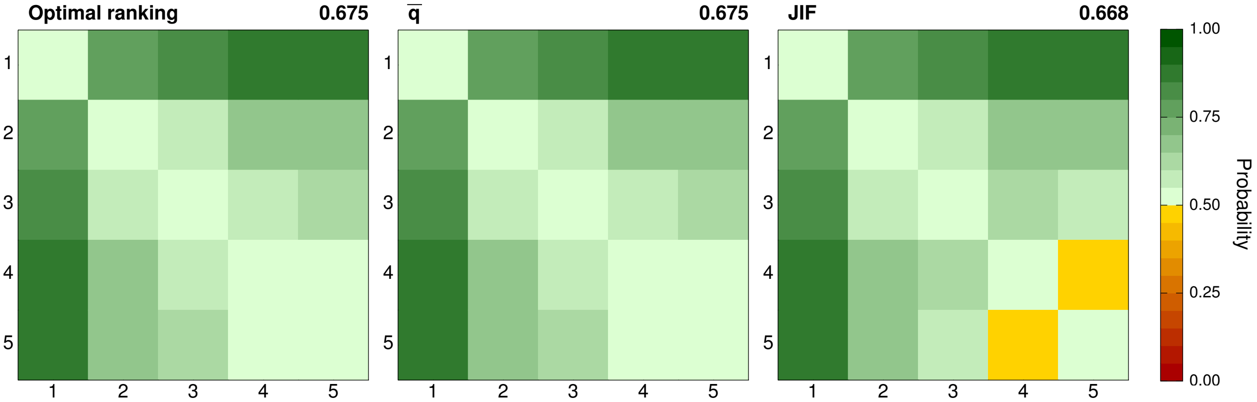

The color of each cell represents the probability of picking a paper with more citations from the higher-ranked journal. Green cells indicate adequate ranking, whereas red cells indicate that the ranking is inadequate (the probability is less than $\frac{1}{2}$). The value of M, the multi-class AUC statistic for each ranking scheme, is above each matrix.

Rank			$p_{ss}(q\|J)$		n			Steady-state
AUC	JIF	Journal abbreviation	$\overline{q}$	σ	$\overline{n}$	Q2	JIF	period
1	1	AAPG BULL	1.04	0.68	21.0	8	1.553	1973–1994
2	2	J CAN PETROL TECHNOL	0.03	0.52	1.6	0	0.273	1968–2003
3	3	HYDROCARB PROCESS	-0.25	0.52	0.8	0	0.145	1983–2005
4	5	OIL GAS J	-0.69	0.47	0.1	0	0.034	1999–2006
5	4	CHEM TECH FUELS OIL+	-0.79	0.43	0.0	0	0.131	1976–2005

ENTOMOLOGY

ISI Category Description Entomology covers resources concerning many aspects of the study of insects, including general entomology, applied entomology, regional entomology, apidology, aquatic insects, insect biochemistry and physiology, economic entomology, integrated pest management, environmental entomology, and pesticide science.

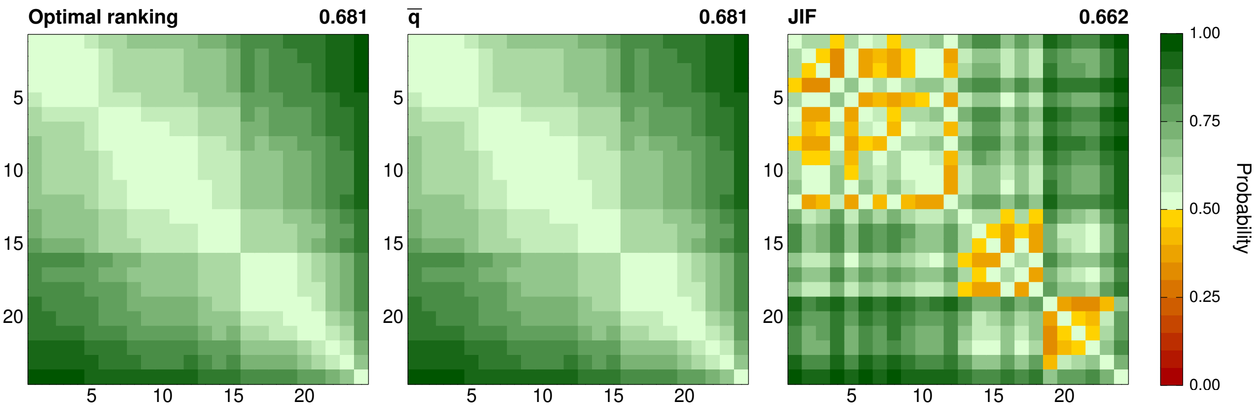

The color of each cell represents the probability of picking a paper with more citations from the higher-ranked journal. Green cells indicate adequate ranking, whereas red cells indicate that the ranking is inadequate (the probability is less than $\frac{1}{2}$). The value of M, the multi-class AUC statistic for each ranking scheme, is above each matrix.

Rank			$p_{ss}(q\|J)$		n			Steady-state
AUC	JIF	Journal abbreviation	$\overline{q}$	σ	$\overline{n}$	Q2	JIF	period
1	4	J INSECT PHYSIOL	1.24	0.34	20.2	15	2.019	1970–1995
2	8	PHYSIOL ENTOMOL	1.18	0.35	17.8	12	1.355	1975–1995
3	1	INSECT BIOCHEM MOLEC	1.18	0.35	17.2	13	2.711	1991–1999
4	6	ARCH INSECT BIOCHEM	1.18	0.35	16.9	13	1.474	1982–1994
5	12	PESTIC BIOCHEM PHYS	1.16	0.38	18.2	12	1.189	1975–1993
6	7	ENTOMOL EXP APPL	1.07	0.37	14.3	10	1.391	1965–1995
7	9	ENVIRON ENTOMOL	1.04	0.33	12.4	9	1.344	1980–1995
8	3	MED VET ENTOMOL	1.02	0.37	12.3	8	2.033	1987–1998
9	2	B ENTOMOL RES	0.98	0.40	12.2	8	2.046	1964–1997
10	10	ANN ENTOMOL SOC AM	0.97	0.39	11.9	7	1.289	1965–1993
11	5	J MED ENTOMOL	0.96	0.39	11.5	7	1.950	1966–1998
12	11	J ECON ENTOMOL	0.95	0.36	10.9	7	1.275	1958–1997
13	16	EXP APPL ACAROL	0.90	0.38	8.8	6	0.716	1985–1999
14	18	CAN ENTOMOL	0.87	0.42	10.0	6	0.568	1963–1995
15	13	J AM MOSQUITO CONTR	0.86	0.38	8.5	6	0.895	1984–1996
16	15	APPL ENTOMOL ZOOL	0.69	0.36	4.9	4	0.770	1993–1999
17	17	FLA ENTOMOL	0.67	0.40	6.6	3	0.667	1976–1999
18	14	J APPL ENTOMOL	0.66	0.36	4.9	3	0.841	1990–2000
19	22	J ENTOMOL SCI	0.62	0.36	4.7	3	0.420	1984–1999
20	21	J KANSAS ENTOMOL SOC	0.56	0.41	4.0	2	0.472	1991–1998
21	20	J PESTIC SCI	0.53	0.46	3.8	2	0.548	1987–1999
22	23	P ENTOMOL SOC WASH	0.35	0.40	2.5	1	0.394	1965–2002
23	19	ENTOMOL NEWS	0.25	0.40	2.0	1	0.556	1976–2003
24	24	AM BEE J	-0.26	0.67	1.1	0	0.087	1976–2004

ENVIRONMENTAL SCIENCES

ISI Category Description Environmental Sciences covers resources concerning many aspects of the study of the environment, among them environmental contamination and toxicology, environmental health, environmental monitoring, environmental geology, and environmental management. This category also includes soil science and conservation, water resources research and engineering and climate change.

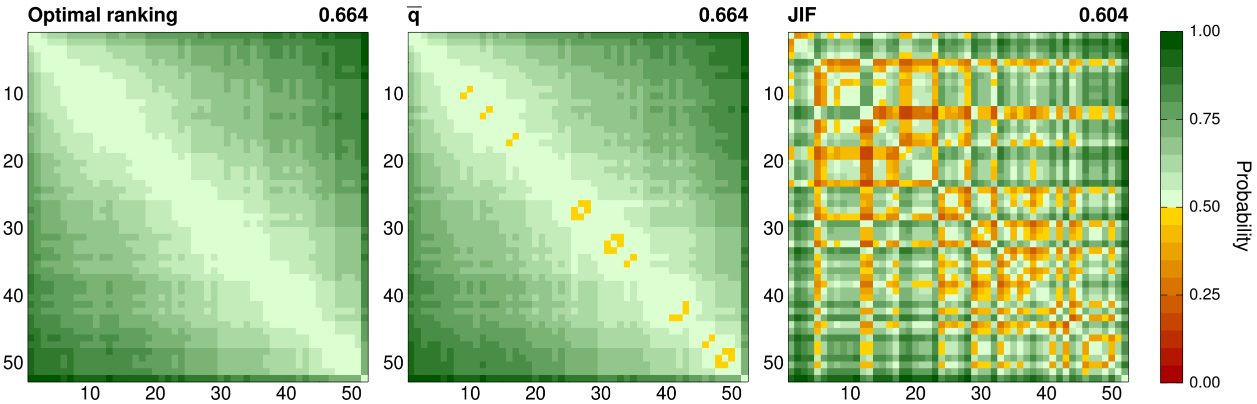

The color of each cell represents the probability of picking a paper with more citations from the higher-ranked journal. Green cells indicate adequate ranking, whereas red cells indicate that the ranking is inadequate (the probability is less than $\frac{1}{2}$). The value of M, the multi-class AUC statistic for each ranking scheme, is above each matrix.

Rank			$p_{ss}(q\|J)$		n			Steady-state
AUC	JIF	Journal abbreviation	$\overline{q}$	σ	$\overline{n}$	Q2	JIF	period
1	2	ENVIRON SCI TECHNOL	1.48	0.40	36.2	26	4.040	1995–1997
2	3	CONSERV BIOL	1.42	0.42	37.5	23	3.762	1988–1998
3	23	WATER RESOUR RES	1.34	0.44	33.2	19	1.894	1981–1992
4	19	ENVIRON TOXICOL CHEM	1.31	0.36	25.0	18	2.202	1983–1995
5	18	J ENVIRON QUAL	1.28	0.36	24.5	16	2.272	1973–1998
6	4	REMOTE SENS ENVIRON	1.27	0.40	26.7	16	3.064	1980–1998
7	28	ESTUARIES	1.24	0.36	21.7	14	1.563	1978–1997
8	7	BIOL CONSERV	1.21	0.38	22.1	14	2.854	1988–1996
9	9	ENVIRON MOL MUTAGEN	1.20	0.38	21.3	14	2.653	1985–1996
10	10	ATMOS ENVIRON	1.21	0.40	21.1	14	2.630	1989–1997
11	1	ENVIRON HEALTH PERSP	1.19	0.45	23.1	13	5.861	1985–2001
12	15	WATER RES	1.18	0.37	20.5	13	2.459	1966–1998
13	11	APPL CATAL A-GEN	1.18	0.34	16.8	13	2.630	1997–1999
14	32	ARCH ENVIRON CON TOX	1.17	0.36	17.5	12	1.419	1975–1995
15	14	ENVIRON RES	1.15	0.40	19.4	12	2.556	1966–1998
16	8	ENVIRON POLLUT	1.12	0.35	16.3	11	2.769	1978–2001
17	38	J GREAT LAKES RES	1.12	0.38	16.1	11	1.000	1982–1995
18	20	MAR ENVIRON RES	1.11	0.37	15.7	10	2.106	1977–1997
19	21	MAR POLLUT BULL	1.08	0.34	14.0	10	2.007	1995–2000
20	22	ECOTOX ENVIRON SAFE	1.06	0.37	13.7	9	2.000	1976–1999
21	44	WATER ENVIRON RES	1.07	0.44	14.0	9	0.665	1991–1996
22	27	J TOXICOL ENV HEALTH	1.03	0.38	14.6	8	1.811	1994–1996
23	6	AEROSOL SCI TECH	1.03	0.41	15.1	8	2.905	1981–2000

table continues on next page . . .

. . . table continued from previous page

Rank			$p_{ss}(q\|J)$		n			Steady-state
**AUC**	**JIF**	**Journal abbreviation**	$\overline{q}$	σ	$\overline{n}$	**Q2**	**JIF**	**period**
24	16	CHEMOSPHERE	1.00	0.32	11.5	8	2.442	2000–2002
25	35	WATER AIR SOIL POLL	1.00	0.41	13.9	8	1.205	1974–1995
26	42	J ENVIRON ENG-ASCE	0.95	0.37	11.5	7	0.725	1982–1998
27	39	INT J ENVIRON AN CH	0.95	0.37	11.7	7	0.917	1976–1996
28	25	AGR ECOSYST ENVIRON	0.97	0.44	11.4	7	1.832	1982–2001
29	37	J ENVIRON RADIOACTIV	0.94	0.35	9.8	7	1.073	1983–1998
30	17	SCI TOTAL ENVIRON	0.92	0.46	11.3	6	2.359	1980–1995
31	26	ENVIRON EXP BOT	0.88	0.37	9.4	6	1.820	1975–2003
32	40	HEALTH PHYS	0.89	0.43	10.5	6	0.902	1964–1994
33	45	J COASTAL RES	0.91	0.43	9.9	6	0.665	1989–1995
34	50	B ENVIRON CONTAM TOX	0.87	0.36	8.4	6	0.505	1987–1991
35	31	AMBIO	0.88	0.48	11.2	6	1.433	1994–2002
36	30	J AIR WASTE MANAGE	0.87	0.44	11.0	5	1.441	1989–2000
37	34	J ARID ENVIRON	0.83	0.38	7.8	5	1.238	1989–2000
38	48	ENVIRON TECHNOL	0.78	0.37	7.3	4	0.528	1989–1999
39	47	J ENVIRON SCI HEAL B	0.78	0.38	7.2	4	0.604	1975–1999
40	36	ENVIRON MANAGE	0.77	0.40	9.4	4	1.097	1976–2001
41	24	J HAZARD MATER	0.76	0.40	7.4	4	1.855	1990–2003
42	5	J AEROSOL SCI	0.77	0.50	9.6	4	2.952	1979–1999
43	12	ENVIRON INT	0.77	0.42	7.1	4	2.626	1996–2003
44	13	INT J HYDROGEN ENERG	0.70	0.42	6.7	4	2.612	1975–2004
45	29	J ENVIRON MANAGE	0.68	0.41	6.2	3	1.477	1973–2003
46	33	ENERG POLICY	0.64	0.38	5.2	3	1.362	1993–2001
47	41	ENVIRON MONIT ASSESS	0.65	0.44	5.9	3	0.793	1983–1999
48	49	ANN CHIM-ROME	0.57	0.38	4.4	3	0.516	1968–1998
49	43	J ENVIRON SCI HEAL A	0.57	0.41	4.6	2	0.669	1975–2000
50	51	RADIAT PROT DOSIM	0.57	0.43	4.6	2	0.446	1983–1999
51	46	ACTA HYDROCH HYDROB	0.55	0.43	4.5	2	0.632	1991–2001
52	52	J ENVIRON BIOL	0.14	0.39	1.2	1	0.197	1980–2003

ENVIRONMENTAL STUDIES

ISI Category Description Environmental Studies covers resources that are multidisciplinary in nature. These include environmental policy, regional science, planning and law, management of natural resources, energy policy, and environmental psychology.

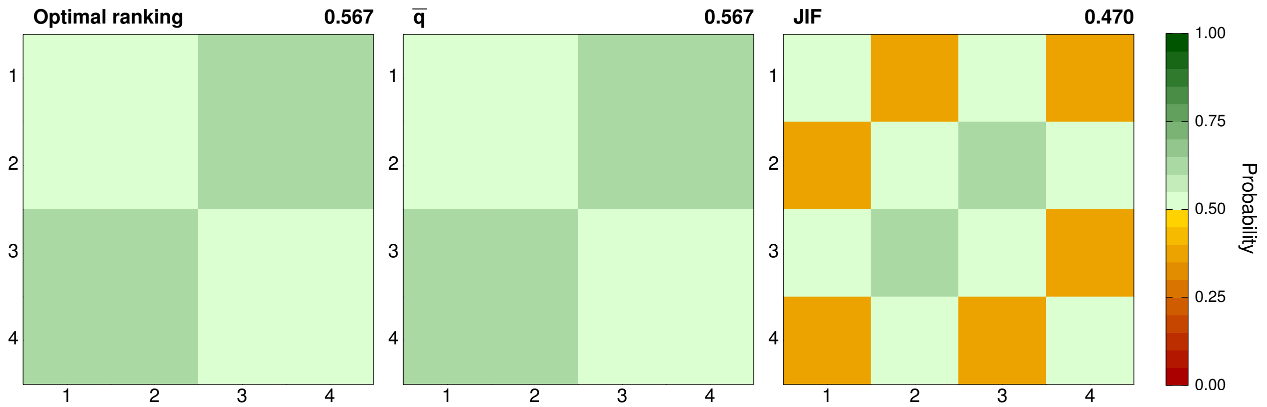

The color of each cell represents the probability of picking a paper with more citations from the higher-ranked journal. Green cells indicate adequate ranking, whereas red cells indicate that the ranking is inadequate (the probability is less than $\frac{1}{2}$). The value of M, the multi-class AUC statistic for each ranking scheme, is above each matrix.

Rank			$p_{ss}(q\|J)$		n			Steady-state
AUC	JIF	Journal abbreviation	$\overline{q}$	σ	$\overline{n}$	Q2	JIF	period
1	4	URBAN STUD	0.83	0.39	8.7	5	0.992	1963–2002
2	2	ENVIRON PLANN A	0.83	0.40	8.6	5	1.610	1972–2002
3	1	LANDSCAPE URBAN PLAN	0.67	0.41	5.3	3	2.029	1985–2004
4	3	ENERG POLICY	0.64	0.38	5.2	3	1.362	1993–2001

ERGONOMICS

ISI Category Description Ergonomics includes resources concerned with the study of the relationship between humans and machines, particularly in a work environment. This category also covers resources on cybernetics, general systems, artificial intelligence, and systems research.

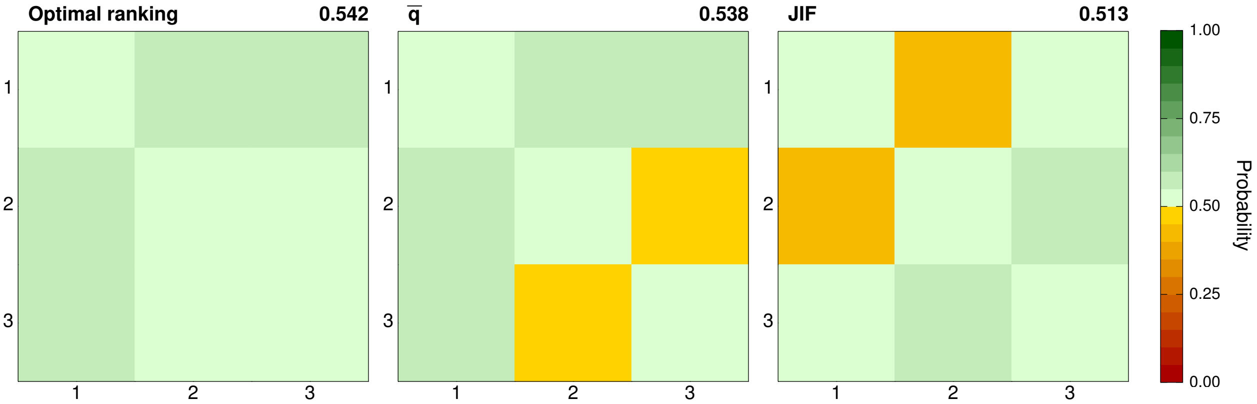

The color of each cell represents the probability of picking a paper with more citations from the higher-ranked journal. Green cells indicate adequate ranking, whereas red cells indicate that the ranking is inadequate (the probability is less than $\frac{1}{2}$). The value of M, the multi-class AUC statistic for each ranking scheme, is above each matrix.

Rank			$p_{ss}(q\|J)$		n			Steady-state
AUC	JIF	Journal abbreviation	$\overline{q}$	σ	$\overline{n}$	Q2	JIF	period
1	2	HUM FACTORS	1.08	0.40	15.6	10	0.861	1980–1996
2	1	ACCIDENT ANAL PREV	0.95	0.38	11.2	7	1.587	1974–2001
3	3	ERGONOMICS	0.96	0.46	13.1	7	0.826	1957–1994

ETHICS

ISI Category Description Ethics covers resources on normative ethics, including all aspects of the evaluation of human conduct and social relations, such as business ethics, medical ethics, environmental ethics, etc. Descriptive ethics is covered extensively in A&HCI, Philosophy.

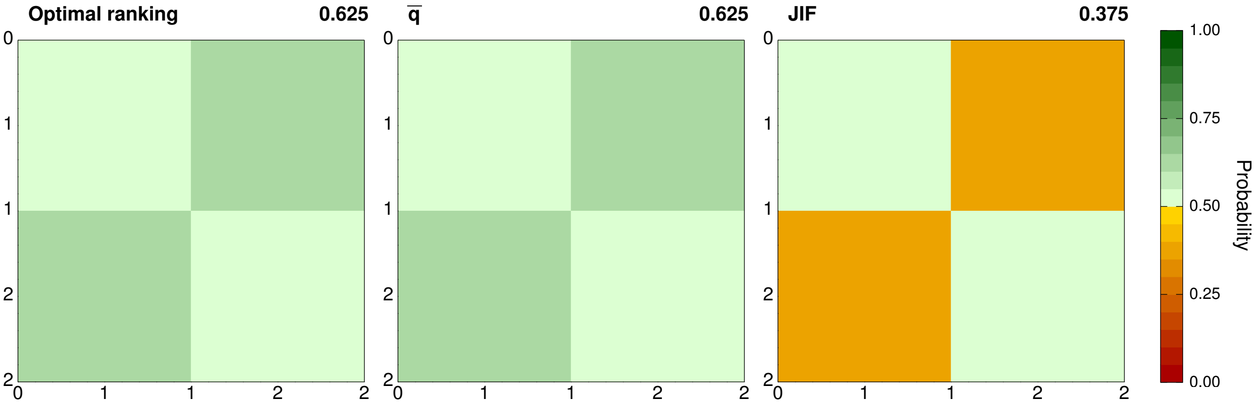

The color of each cell represents the probability of picking a paper with more citations from the higher-ranked journal. Green cells indicate adequate ranking, whereas red cells indicate that the ranking is inadequate (the probability is less than $\frac{1}{2}$). The value of M, the multi-class AUC statistic for each ranking scheme, is above each matrix.

Rank			$\mathbf{p_{ss}(q\|J)}$		n			Steady-state
**AUC**	**JIF**	**Journal abbreviation**	$\overline{\mathbf{q}}$	σ	$\overline{\mathbf{n}}$	**Q2**	**JIF**	**period**
1	2	J BUS ETHICS	0.84	0.34	9.1	5	0.597	1986–1996
2	1	HASTINGS CENT REP	0.66	0.61	7.8	3	1.848	1992–1999

EVOLUTIONARY BIOLOGY

ISI Category Description Evolutionary Biology covers resources concerning the molecular, natural selection, and population mechanisms of evolution; the evolution of species and related groups; the classification of organisms based on evolutionary relationships; and the biology and ecology of extinct organisms.

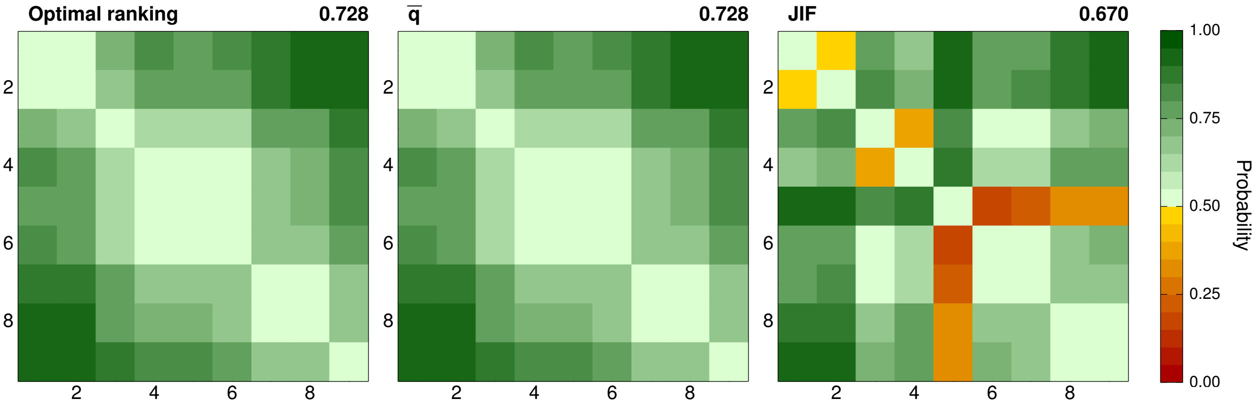

The color of each cell represents the probability of picking a paper with more citations from the higher-ranked journal. Green cells indicate adequate ranking, whereas red cells indicate that the ranking is inadequate (the probability is less than $\frac{1}{2}$). The value of M, the multi-class AUC statistic for each ranking scheme, is above each matrix.

Rank			$p_{ss}(q\|J)$		n			Steady-state
**AUC**	**JIF**	**Journal abbreviation**	$\overline{q}$	σ	$\overline{n}$	**Q2**	**JIF**	**period**
1	2	EVOLUTION	1.67	0.35	69.8	43	4.292	1973–1993
2	1	MOL BIOL EVOL	1.60	0.35	72.9	35	6.726	1983–1998
3	4	J MOL EVOL	1.37	0.38	40.4	20	2.767	1971–1999
4	3	J HUM EVOL	1.22	0.35	18.1	14	3.267	1989–2000
5	6	BIOL J LINN SOC	1.20	0.38	23.9	13	2.445	1973–1999
6	7	AM J PHYS ANTHROPOL	1.19	0.40	19.2	13	2.136	1955–1992
7	8	PLANT SYST EVOL	0.93	0.43	10.8	7	1.239	1973–2000
8	9	BIOCHEM SYST ECOL	0.89	0.36	9.2	6	0.906	1980–1994
9	5	TAXON	0.58	0.49	6.4	2	2.519	1975–2003

FAMILY STUDIES

ISI Category Description Family Studies includes resources on such issues and areas as family therapy, family law, marriage, divorce, family planning, and family history. Cross-disciplinary in nature, many resources in this category also appear in other categories.

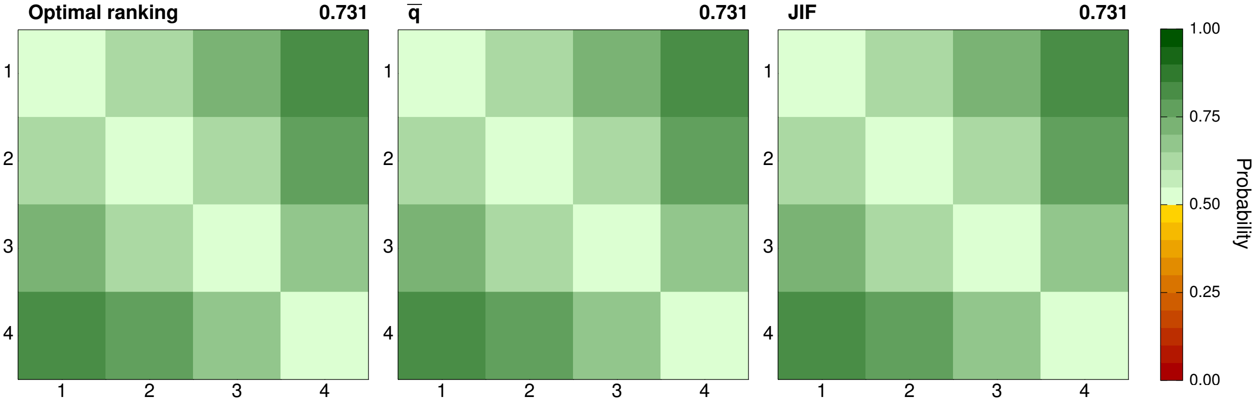

The color of each cell represents the probability of picking a paper with more citations from the higher-ranked journal. Green cells indicate adequate ranking, whereas red cells indicate that the ranking is inadequate (the probability is less than $\frac{1}{2}$). The value of M, the multi-class AUC statistic for each ranking scheme, is above each matrix.

**Rank**			$\mathbf{p_{ss}(q\|J)}$		**n**			**Steady-state**
**AUC**	**JIF**	**Journal abbreviation**	$\overline{\mathbf{q}}$	σ	$\overline{\mathbf{n}}$	**Q2**	**JIF**	**period**
1	1	J MARRIAGE FAM	1.34	0.41	31.6	19	1.763	1975–1994
2	2	CHILD ABUSE NEGLECT	1.14	0.31	18.3	12	1.623	1994–1996
3	3	FAM RELAT	0.96	0.38	11.6	7	0.731	1981–1998
4	4	CHILD WELFARE	0.71	0.41	6.3	4	0.500	1977–2001

FISHERIES

ISI Category Description Fisheries covers resources concerning numerous aspects of fisheries science, technology and industry, including fish pathology, fish physiology and biochemistry, fish diseases and aquaculture.

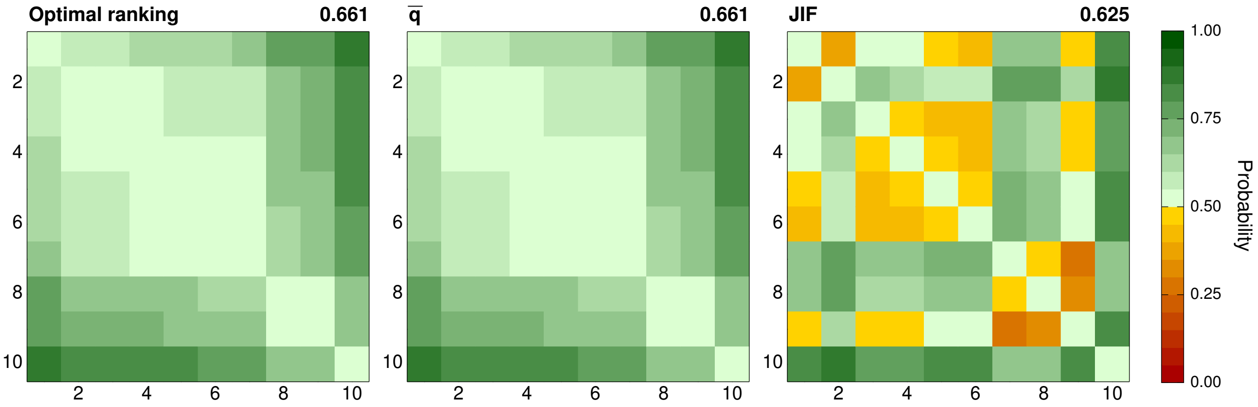

The color of each cell represents the probability of picking a paper with more citations from the higher-ranked journal. Green cells indicate adequate ranking, whereas red cells indicate that the ranking is inadequate (the probability is less than $\frac{1}{2}$). The value of M, the multi-class AUC statistic for each ranking scheme, is above each matrix.

Rank			$\mathbf{p_{ss}(q\|J)}$		n			Steady-state
**AUC**	**JIF**	**Journal abbreviation**	$\overline{\mathbf{q}}$	σ	$\overline{\mathbf{n}}$	**Q2**	**JIF**	**period**
1	2	CAN J FISH AQUAT SCI	1.35	0.34	28.4	20	1.882	1988–1993
2	6	T AM FISH SOC	1.24	0.38	22.8	14	1.386	1964–1995
3	5	J FISH BIOL	1.23	0.37	21.1	14	1.393	1968–1993
4	9	FISH PHYSIOL BIOCHEM	1.20	0.36	19.1	14	0.558	1986–1995
5	1	AQUACULTURE	1.16	0.33	16.0	12	2.081	1993–1999
6	4	FISH B-NOAA	1.15	0.33	16.5	12	1.403	1988–1995
7	3	DIS AQUAT ORGAN	1.15	0.35	16.3	11	1.509	1984–1999
8	8	NEW ZEAL J MAR FRESH	0.94	0.37	10.7	7	0.931	1976–1998
9	7	FISH RES	0.90	0.38	9.0	6	1.216	1980–2000
10	10	NIPPON SUISAN GAKK	0.61	0.47	5.8	3	0.176	1986–1993

FOOD SCIENCE & TECHNOLOGY

ISI Category Description Food Science & Technology covers resources concerning various aspects of food research and production, including food additives and contaminants, food chemistry and biochemistry, meat science, food microbiology and technology, dairy science, food engineering and processing, cereal science, brewing, and food quality and safety.

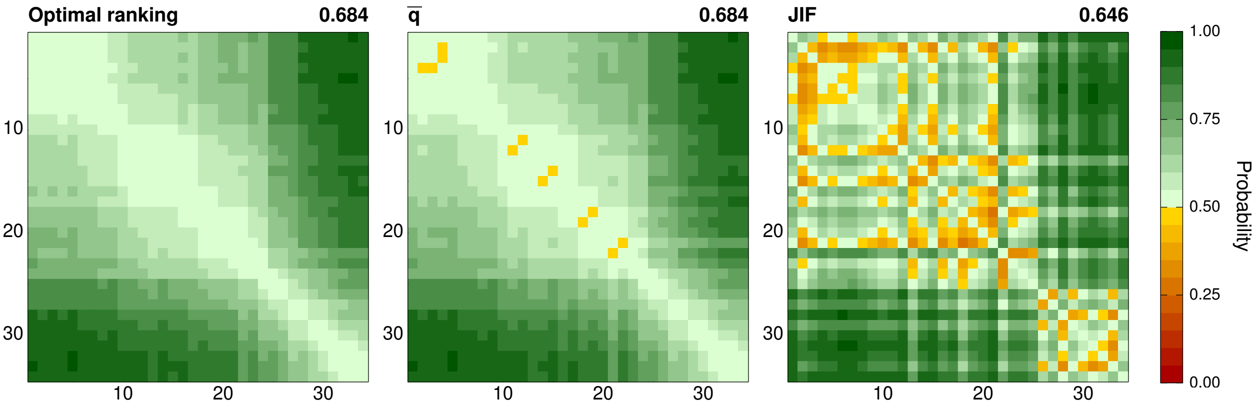

The color of each cell represents the probability of picking a paper with more citations from the higher-ranked journal. Green cells indicate adequate ranking, whereas red cells indicate that the ranking is inadequate (the probability is less than $\frac{1}{2}$). The value of M, the multi-class AUC statistic for each ranking scheme, is above each matrix.

Rank			$\mathbf{p_{ss}(q\|J)}$		n			Steady-state
**AUC**	**JIF**	**Journal abbreviation**	$\overline{\mathbf{q}}$	σ	$\overline{\mathbf{n}}$	**Q2**	**JIF**	**period**
1	7	J CEREAL SCI	1.21	0.37	20.2	14	2.046	1987–1996
2	4	J AGR FOOD CHEM	1.20	0.38	21.2	13	2.322	1966–1994
3	1	INT J FOOD MICROBIOL	1.20	0.37	19.5	13	2.608	1983–2000
4	12	J DAIRY RES	1.20	0.40	20.1	13	1.407	1954–1998
5	6	BIOTECHNOL PROGR	1.19	0.35	19.2	13	2.102	1984–1998
6	15	CEREAL CHEM	1.17	0.38	19.3	12	1.254	1954–1995
7	21	J FOOD SCI	1.15	0.37	17.9	12	1.004	1960–1995
8	5	J DAIRY SCI	1.14	0.40	18.8	11	2.284	1958–1998
9	8	J FOOD PROTECT	1.13	0.36	16.0	11	1.921	1992–1999
10	9	MEAT SCI	1.07	0.39	15.0	10	1.840	1979–2000
11	3	FOOD CHEM TOXICOL	1.04	0.37	14.3	9	2.393	1981–1999
12	11	J FOOD ENG	1.05	0.41	12.7	9	1.696	1991–1995
13	20	AM J ENOL VITICULT	1.03	0.42	13.9	8	1.009	1967–2000
14	14	BIOSCI BIOTECH BIOCH	1.00	0.35	12.1	8	1.256	1991–1994
15	17	J SCI FOOD AGR	1.00	0.40	12.8	8	1.026	1986–1996
16	10	FOOD ADDIT CONTAM	0.97	0.37	11.4	8	1.780	1985–2001
17	23	J AM OIL CHEM SOC	0.98	0.46	15.0	7	0.910	1955–1991
18	2	FOOD CHEM	0.91	0.37	9.9	6	2.433	1976–2002
19	19	J I BREWING	0.91	0.43	10.3	6	1.012	1968–1994
20	24	INT J FOOD SCI TECH	0.87	0.39	9.1	5	0.832	1990–2000
21	16	STARCH-STARKE	0.86	0.40	9.6	6	1.136	1984–2001
22	13	J AOAC INT	0.87	0.56	11.0	5	1.352	1991–1996
23	25	MILCHWISSENSCHAFT	0.79	0.42	7.9	5	0.528	1975–1997

table continues on next page . . .

. . . table continued from previous page

Rank			$p_{ss}(q\|J)$		n			Steady-state
**AUC**	**JIF**	**Journal abbreviation**	$\overline{q}$	σ	$\overline{n}$	**Q2**	**JIF**	**period**
24	18	FOOD TECHNOL-CHICAGO	0.74	0.60	11.1	3	1.024	1972–1998
25	22	NAHRUNG	0.59	0.38	4.6	3	0.978	1994–2001
26	27	DEUT LEBENSM-RUNDSCH	0.55	0.42	4.4	2	0.414	1976–1997
27	29	CEREAL FOOD WORLD	0.47	0.62	4.9	1	0.200	1974–2000
28	33	J FOOD SCI TECH MYS	0.38	0.38	2.6	1	0.107	1975–1998
29	26	J FOOD HYG SOC JPN	0.28	0.48	2.3	1	0.451	1983–2001
30	30	ZUCKERINDUSTRIE	0.23	0.49	2.0	1	0.168	1977–2000
31	32	FLEISCHWIRTSCHAFT	0.12	0.52	1.7	0	0.115	1977–1998
32	28	FOOD DRUG LAW J	0.04	0.48	1.2	0	0.397	1972–2005
33	31	J JPN SOC FOOD SCI	0.01	0.46	1.2	0	0.135	1977–2000
34	34	FOOD AUST	-0.07	0.56	1.3	0	0.088	1987–2003

FORESTRY

ISI Category Description Forestry covers resources concerning the science and technology involved in establishing, maintaining and managing forests for various uses, including wood production, water resource management, wildlife conservation and recreation.

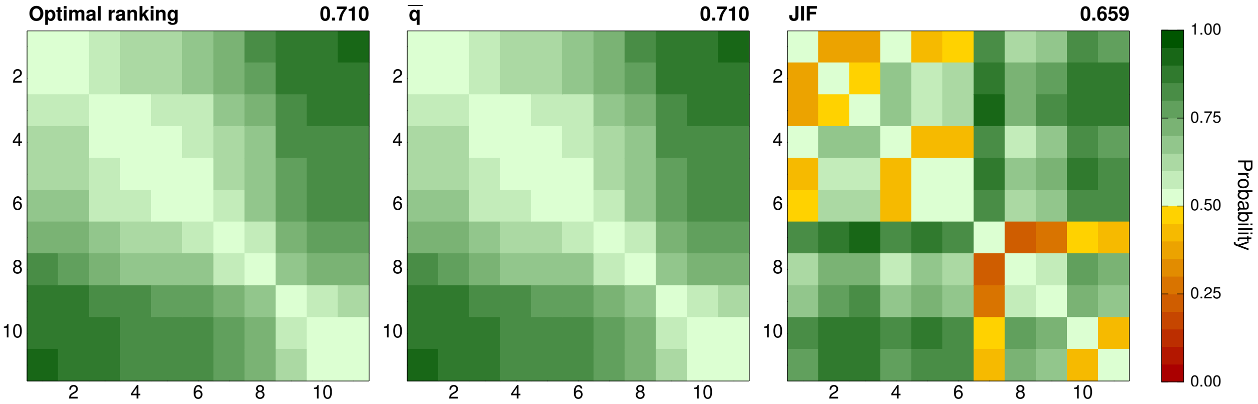

The color of each cell represents the probability of picking a paper with more citations from the higher-ranked journal. Green cells indicate adequate ranking, whereas red cells indicate that the ranking is inadequate (the probability is less than $\frac{1}{2}$). The value of M, the multi-class AUC statistic for each ranking scheme, is above each matrix.

Rank			$p_{ss}(q\|J)$		n			Steady-state
**AUC**	**JIF**	**Journal abbreviation**	$\overline{q}$	σ	$\overline{n}$	**Q2**	**JIF**	**period**
1	3	TREE PHYSIOL	1.29	0.35	22.5	16	2.297	1986–1999
2	2	J VEG SCI	1.28	0.36	22.4	16	2.382	1989–1999
3	5	CAN J FOREST RES	1.19	0.40	20.2	13	1.549	1976–1993
4	6	FOREST SCI	1.12	0.38	17.1	11	1.457	1965–1994
5	1	AGR FOREST METEOROL	1.09	0.39	17.3	10	2.903	1983–2002
6	4	FOREST ECOL MANAG	1.03	0.37	13.2	9	1.839	1975–2000
7	8	HOLZFORSCHUNG	0.94	0.41	11.5	7	1.014	1965–1999
8	9	AGROFOREST SYST	0.81	0.30	7.1	5	0.921	1999–2001
9	11	FOREST PROD J	0.56	0.39	4.3	2	0.387	1975–2000
10	10	FOREST CHRON	0.41	0.54	3.8	1	0.831	1965–2001
11	7	J FOREST	0.38	0.52	3.7	1	1.188	1965–2003

GASTROENTEROLOGY & HEPATOLOGY

ISI Category Description Gastroenterology & Hepatology covers resources on the anatomy, physiology, biochemistry, and pathology of the digestive system. This category includes specific resources on the prognosis and treatment of digestive diseases; stomach ulcers; metabolic, genetic, infectious and chemically induced diseases of the liver; colitis; diseases of the pancreas and diseases of the rectum.

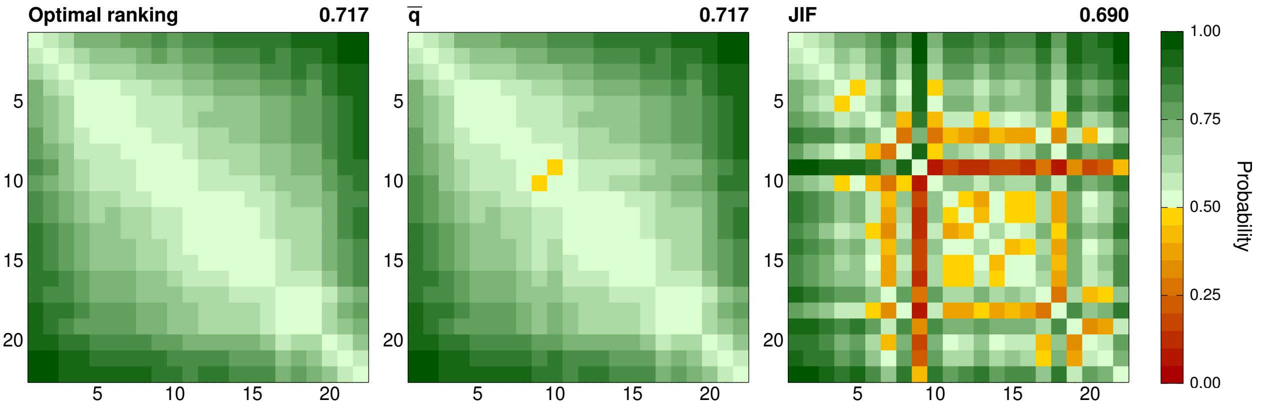

The color of each cell represents the probability of picking a paper with more citations from the higher-ranked journal. Green cells indicate adequate ranking, whereas red cells indicate that the ranking is inadequate (the probability is less than $\frac{1}{2}$). The value of M, the multi-class AUC statistic for each ranking scheme, is above each matrix.

Rank			$p_{ss}(q\|J)$		n			Steady-state
**AUC**	**JIF**	**Journal abbreviation**	$\bar{q}$	σ	$\bar{n}$	**Q2**	**JIF**	**period**
1	1	GASTROENTEROLOGY	1.66	0.38	61.4	41	12.457	1969–1998
2	2	HEPATOLOGY	1.54	0.37	47.9	31	10.446	1980–2000
3	3	GUT	1.46	0.40	38.9	25	9.002	1959–2000
4	5	AM J GASTROENTEROL	1.34	0.37	28.9	19	5.608	1992–1994
5	10	DIS COLON RECTUM	1.31	0.41	24.4	17	2.442	1989–1995
6	4	J HEPATOL	1.29	0.37	24.8	16	6.073	1984–2000
7	8	ALIMENT PHARM THERAP	1.25	0.37	22.8	15	3.287	1992–1999
8	18	DIGEST DIS SCI	1.21	0.37	21.5	13	1.448	1991–1995
9	13	J PEDIATR GASTR NUTR	1.17	0.36	17.5	12	2.067	1993–1995
10	6	GASTROINTEST ENDOSC	1.17	0.48	21.0	11	4.825	1973–1999
11	15	SCAND J GASTROENTERO	1.09	0.43	17.2	10	1.869	1967–1998
12	16	DIGESTION	1.05	0.39	15.2	9	1.826	1967–2000
13	12	PANCREAS	1.04	0.36	13.2	9	2.121	1986–1998
14	11	J CLIN GASTROENTEROL	1.01	0.37	12.8	8	2.403	1978–1995
15	14	EUR J GASTROEN HEPAT	1.00	0.38	11.6	8	1.895	1996–1999
16	20	HEPATO-GASTROENTEROL	0.94	0.37	10.3	7	0.756	1979–1997
17	7	ENDOSCOPY	0.86	0.53	10.9	5	3.605	1995–2001
18	21	GASTROEN CLIN BIOL	0.83	0.37	7.7	5	0.749	1976–1995
19	17	J GASTROEN HEPATOL	0.83	0.44	9.2	5	1.785	1989–2000
20	19	Z GASTROENTEROL	0.63	0.43	5.6	3	1.293	1990–1999
21	22	ACTA GASTRO-ENT BELG	0.47	0.45	3.4	2	0.736	1991–2002
22	9	CURR OPIN GASTROEN	0.31	0.48	2.5	1	3.045	1990–2005

GENETICS & HEREDITY

ISI Category Description Genetics & Heredity includes resources that deal with the structure, functions, and properties of genes, and the characteristics of inheritance. This category also considers heritable traits, population genetics, frequency and distribution of polymorphism, as well as inherited diseases and disorders of the replicative process. The category is distinguishable from Biochemistry & Molecular Biology by its specific emphasis on the gene as a single functional unit, and on the gene's effect on the organism as a whole.

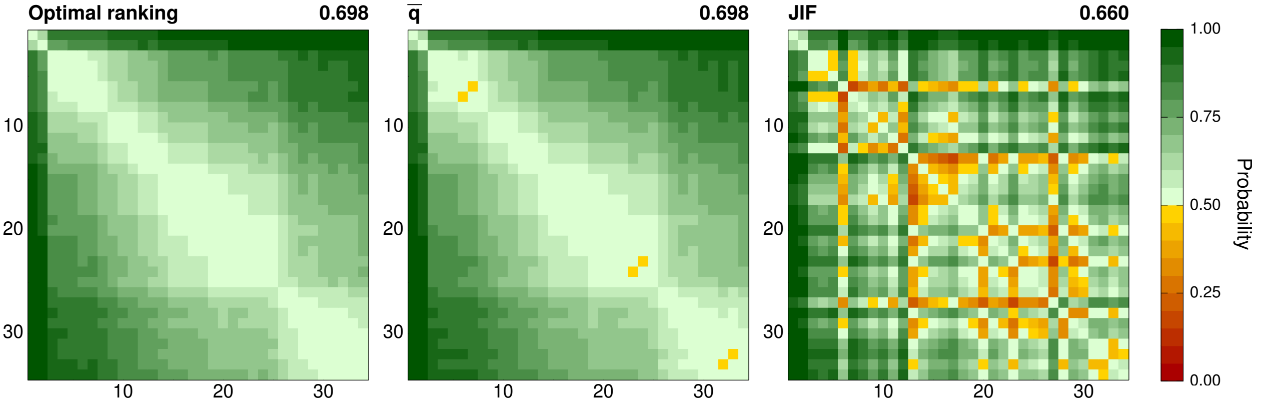

The color of each cell represents the probability of picking a paper with more citations from the higher-ranked journal. Green cells indicate adequate ranking, whereas red cells indicate that the ranking is inadequate (the probability is less than $\frac{1}{2}$). The value of M, the multi-class AUC statistic for each ranking scheme, is above each matrix.

**Rank**			$\mathbf{p_{ss}(q\|J)}$		**n**			**Steady-state**
**AUC**	**JIF**	**Journal abbreviation**	$\overline{\mathbf{q}}$	σ	$\overline{\mathbf{n}}$	**Q2**	**JIF**	**period**
1	1	NAT GENET	2.19	0.31	193.0	152	24.176	1991–1998
2	2	GENE DEV	2.04	0.30	143.5	106	15.050	1988–1996
3	7	EVOLUTION	1.67	0.35	69.8	43	4.292	1973–1993
4	5	ONCOGENE	1.66	0.36	60.4	41	6.582	1992–1995
5	3	AM J HUM GENET	1.66	0.35	60.1	40	12.629	1989–2001
6	4	MOL BIOL EVOL	1.60	0.35	72.9	35	6.726	1983–1998
7	12	GENOMICS	1.61	0.31	54.6	35	3.558	1987–1991
8	8	GENETICS	1.55	0.36	56.3	31	4.242	1986–1995
9	10	GENE CHROMOSOME CANC	1.48	0.33	35.4	27	3.900	1995–1998
10	17	GENE	1.48	0.40	63.8	25	2.721	1976–1990
11	27	DNA CELL BIOL	1.44	0.36	45.5	25	1.905	1989–1991
12	9	CHROMOSOMA	1.38	0.37	34.4	21	4.065	1955–1996
13	16	J MOL EVOL	1.37	0.38	40.4	20	2.767	1971–1999
14	15	IMMUNOGENETICS	1.32	0.37	27.7	18	2.852	1976–1995
15	11	HUM GENET	1.27	0.36	24.5	16	3.662	1975–1999
16	21	CURR GENET	1.28	0.37	23.4	16	2.220	1978–1992
17	18	THEOR APPL GENET	1.25	0.40	23.9	15	2.715	1985–1999
18	29	CANCER GENET CYTOGEN	1.24	0.39	25.2	14	1.544	1980–1990
19	19	ENVIRON MOL MUTAGEN	1.20	0.38	21.3	14	2.653	1985–1996
20	24	GENET RES	1.20	0.41	22.3	13	2.000	1966–1999
21	14	HEREDITY	1.20	0.36	19.8	13	2.872	1984–1998
22	6	J MED GENET	1.19	0.37	20.1	13	5.087	1970–1999

table continues on next page . . .

. . . table continued from previous page

**Rank**			$\mathbf{p_{ss}(q\|J)}$		$\mathbf{n}$			**Steady-state**
**AUC**	**JIF**	**Journal abbreviation**	$\overline{\mathbf{q}}$	σ	$\overline{\mathbf{n}}$	**Q2**	**JIF**	**period**
23	22	MUTAGENESIS	1.15	0.35	18.4	12	2.125	1985–1993
24	30	PRENATAL DIAG	1.15	0.34	17.1	12	1.514	1985–1995
25	25	GENOME	1.14	0.38	19.0	11	1.972	1992–1997
26	26	J HERED	1.06	0.39	16.6	9	1.942	1966–1998
27	33	HUM BIOL	1.00	0.38	13.2	8	1.132	1977–1999
28	13	CLIN GENET	0.97	0.35	10.7	7	3.140	1989–2001
29	20	MAMM GENOME	0.96	0.39	12.5	7	2.279	1997–2002
30	28	J INHERIT METAB DIS	0.92	0.39	10.5	6	1.574	1980–1998
31	23	HUM HERED	0.90	0.39	11.8	6	2.051	1971–2003
32	31	GENETICA	0.91	0.43	10.9	6	1.492	1960–1999
33	34	BIOCHEM GENET	0.90	0.40	9.4	6	0.876	1986–1997
34	32	HEREDITAS	0.86	0.38	8.3	5	1.269	1985–1998

GEOCHEMISTRY & GEOPHYSICS

ISI Category Description Resources in this category may focus on either Geochemistry or Geophysics or both. Geochemistry covers resources that deal with the chemical composition and chemical changes in the Earth or other planets or asteroids. Topics include research on related chemical and geological properties of substances, applied geochemistry, organic geochemistry, and biogeochemistry. Geophysics covers resources on the application of the methods and techniques of physics to the study of the structure of the Earth and the processes affecting it. Topics addressed include seismology, tectonics, tectonophysics, geomagnetism, radioactivity, and rock mechanics.

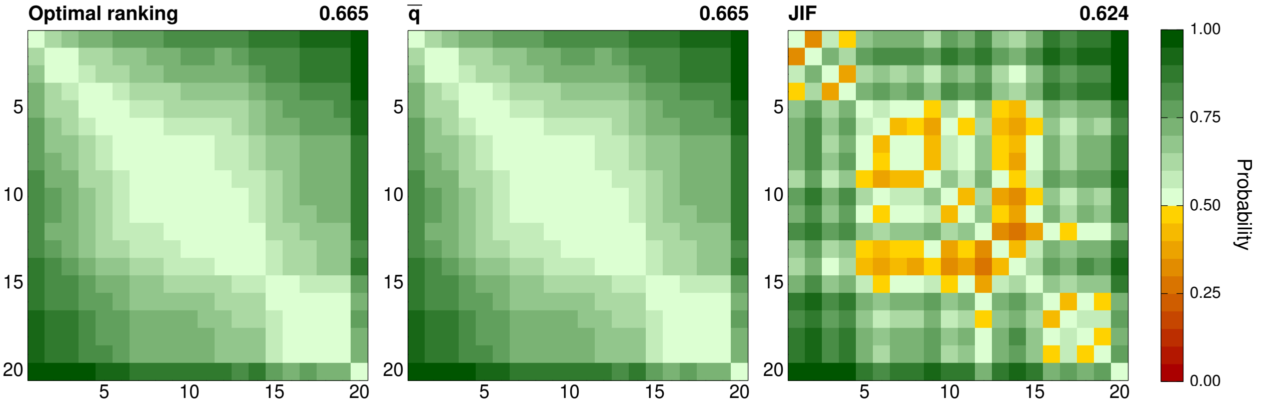

The color of each cell represents the probability of picking a paper with more citations from the higher-ranked journal. Green cells indicate adequate ranking, whereas red cells indicate that the ranking is inadequate (the probability is less than $\frac{1}{2}$). The value of M, the multi-class AUC statistic for each ranking scheme, is above each matrix.

Rank			$p_{ss}(q\|J)$		n			Steady-state
**AUC**	**JIF**	**Journal abbreviation**	$\overline{q}$	σ	$\overline{n}$	**Q2**	**JIF**	**period**
1	2	GEOCHIM COSMOCHIM AC	1.69	0.37	69.1	44	3.751	1975–1985
2	4	CONTRIB MINERAL PETR	1.51	0.35	46.7	28	2.754	1974–1993
3	1	EARTH PLANET SC LETT	1.46	0.34	36.3	25	3.887	1993–1997
4	3	TECTONICS	1.36	0.32	27.5	20	3.143	1991–1997
5	14	ECON GEOL	1.31	0.38	28.0	17	1.345	1971–1989
6	9	AM MINERAL	1.27	0.41	27.8	16	1.977	1954–1997
7	13	TECTONOPHYSICS	1.20	0.44	23.6	14	1.675	1965–1995
8	5	CHEM GEOL	1.19	0.41	23.4	13	2.716	1967–2000
9	7	GEOPHYS J INT	1.15	0.40	20.2	12	2.353	1984–1995
10	8	ORG GEOCHEM	1.13	0.36	16.8	11	2.331	1987–2000
11	11	B SEISMOL SOC AM	1.12	0.37	19.0	11	1.828	1994–1998
12	15	GEOPHYSICS	1.10	0.43	21.3	10	1.228	1963–1992
13	6	PHYS EARTH PLANET IN	1.07	0.44	18.6	9	2.440	1974–1996
14	10	APPL GEOCHEM	1.02	0.36	13.2	9	1.866	1990–2000
15	17	RADIO SCI	0.91	0.45	12.2	6	1.084	1965–1997
16	12	IEEE T GEOSCI REMOTE	0.83	0.49	11.6	5	1.752	1964–2006
17	19	J GEOCHEM EXPLOR	0.80	0.38	8.4	5	0.922	1974–1998
18	16	PURE APPL GEOPHYS	0.77	0.44	8.6	4	1.171	1989–2000
19	18	GEOPHYS PROSPECT	0.75	0.41	8.0	4	1.046	1977–2000
20	20	NUOVO CIMENTO C	0.25	0.48	3.3	1	0.294	1986–1999

GEOGRAPHY, PHYSICAL

ISI Category Description Geography, Physical covers resources dealing with the differentiation of areas of the Earth's surface as shown in the character, arrangement, and interrelations over the world of such elements as climate, elevation, soil, vegetation, population, land use, industries, or states, as well as the unit areas formed by the complex of these individual elements. Resources which focus on economic, human, and urban topics are covered in the SSCI GEOGRAPHY category.

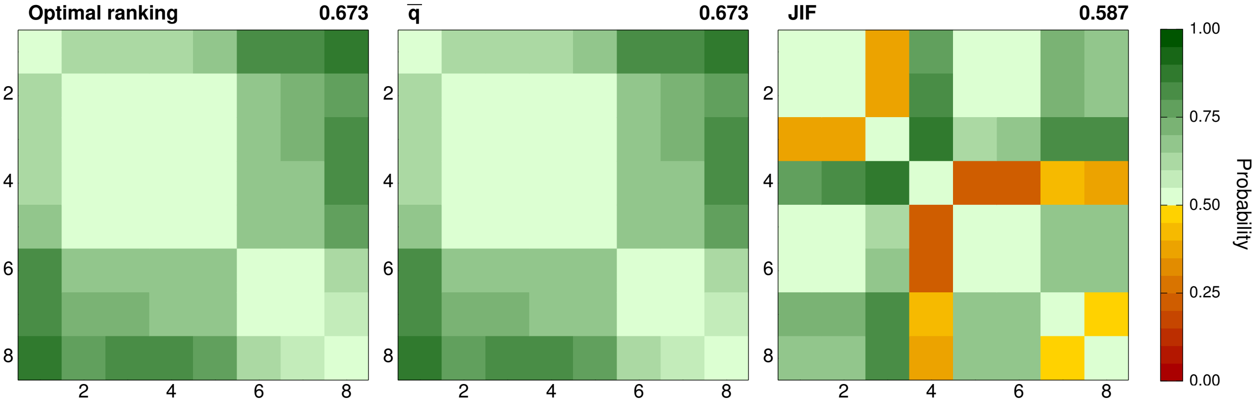

The color of each cell represents the probability of picking a paper with more citations from the higher-ranked journal. Green cells indicate adequate ranking, whereas red cells indicate that the ranking is inadequate (the probability is less than $\frac{1}{2}$). The value of M, the multi-class AUC statistic for each ranking scheme, is above each matrix.

Rank			$p_{ss}(q\|J)$		n			Steady-state
AUC	JIF	Journal abbreviation	$\overline{q}$	σ	$\overline{n}$	Q2	JIF	period
1	3	QUATERNARY RES	1.41	0.36	34.5	22	2.319	1973–1997
2	1	QUATERNARY SCI REV	1.21	0.40	20.0	14	4.113	1993–1999
3	2	J BIOGEOGR	1.21	0.36	20.4	13	2.878	1976–1998
4	5	PALAEOGEOGR PALAEOCL	1.19	0.38	20.4	13	1.822	1967–1997
5	6	J GLACIOL	1.15	0.41	17.4	12	1.446	1979–1999
6	8	J COASTAL RES	0.91	0.43	9.9	6	0.665	1989–1995
7	7	PHOTOGRAMM ENG REM S	0.83	0.51	11.1	5	1.284	1978–1999
8	4	LANDSCAPE URBAN PLAN	0.67	0.41	5.3	3	2.029	1985–2004

GEOGRAPHY

ISI Category Description Geography covers resources concerned with socio-cultural aspects of the Earth's surface emphasizing the human, economic, political, urban, and environmental issues of the discipline. The history of geography and the study of cartography are also covered in this category.

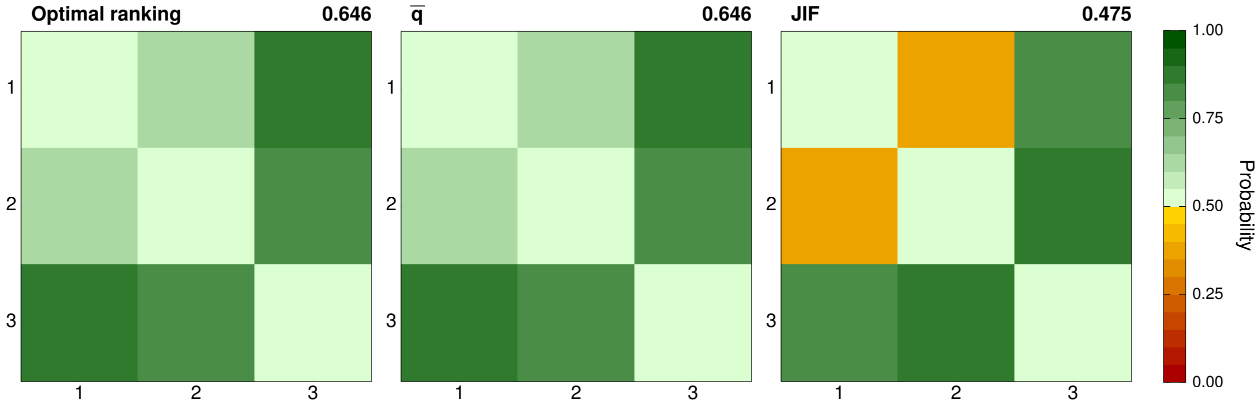

The color of each cell represents the probability of picking a paper with more citations from the higher-ranked journal. Green cells indicate adequate ranking, whereas red cells indicate that the ranking is inadequate (the probability is less than $\frac{1}{2}$). The value of M, the multi-class AUC statistic for each ranking scheme, is above each matrix.

Rank			$p_{ss}(q\|J)$		n			Steady-state
**AUC**	**JIF**	**Journal abbreviation**	$\overline{q}$	σ	$\overline{n}$	**Q2**	**JIF**	**period**
1	2	ENVIRON PLANN A	0.83	0.40	8.6	5	1.610	1972–2002
2	1	LANDSCAPE URBAN PLAN	0.67	0.41	5.3	3	2.029	1985–2004
3	3	J GEOGR	-0.05	0.35	0.8	0	0.320	2000–2006

GEOLOGY

ISI Category Description Geology covers resources that deal with the physical history of the Earth, the rock of which it is composed, and the physical changes (not the physics) that the Earth has undergone or is undergoing. Resources in this category cover sedimentology, stratigraphy, hydrogeology, ore geology, structural geology, regional geology, and petrology. These resources are somewhat narrow in scope and are not given to the interdisciplinary study of the Earth Sciences.

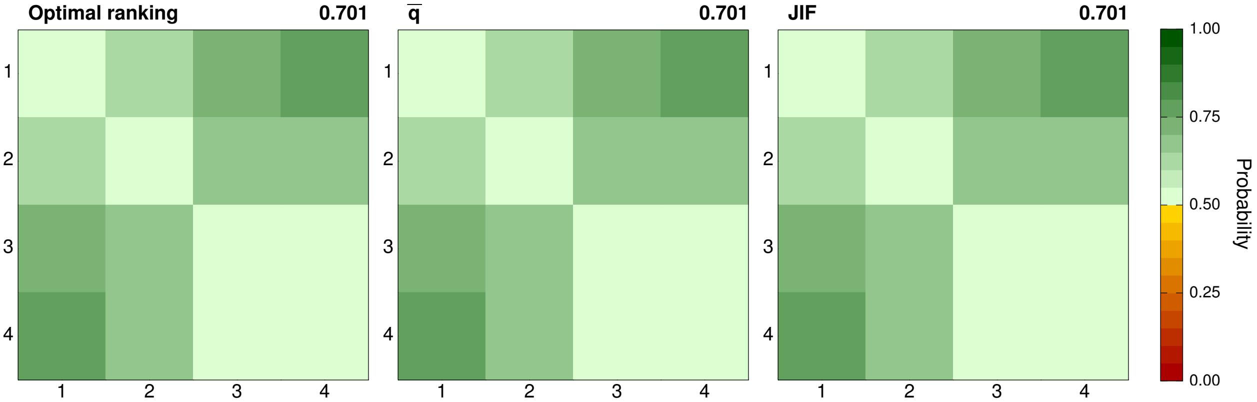

The color of each cell represents the probability of picking a paper with more citations from the higher-ranked journal. Green cells indicate adequate ranking, whereas red cells indicate that the ranking is inadequate (the probability is less than $\frac{1}{2}$). The value of M, the multi-class AUC statistic for each ranking scheme, is above each matrix.

Rank			$p_{ss}(q\|J)$		n			Steady-state
**AUC**	**JIF**	**Journal abbreviation**	$\overline{q}$	σ	$\overline{n}$	**Q2**	**JIF**	**period**
1	1	GEOLOGY	1.41	0.38	33.6	22	3.477	1975–1995
2	2	SEDIMENTOLOGY	1.26	0.29	19.7	15	1.746	1989–1997
3	3	SEDIMENT GEOL	1.03	0.37	13.6	9	1.313	1967–1999
4	4	NEW ZEAL J GEOL GEOP	0.99	0.40	11.5	8	0.822	1966–1996

GEOSCIENCES, MULTIDISCIPLINARY

ISI Category Description Geosciences, Multidisciplinary covers resources having a general or interdisciplinary approach to the study of the Earth and other planets. Relevant topics include geology, geochemistry/geophysics, hydrology, paleontology, oceanography, meteorology, mineralogy, geography, and energy and fuels. Resources having a primary focus on geology, or geochemistry & geophysics are placed in their own categories.

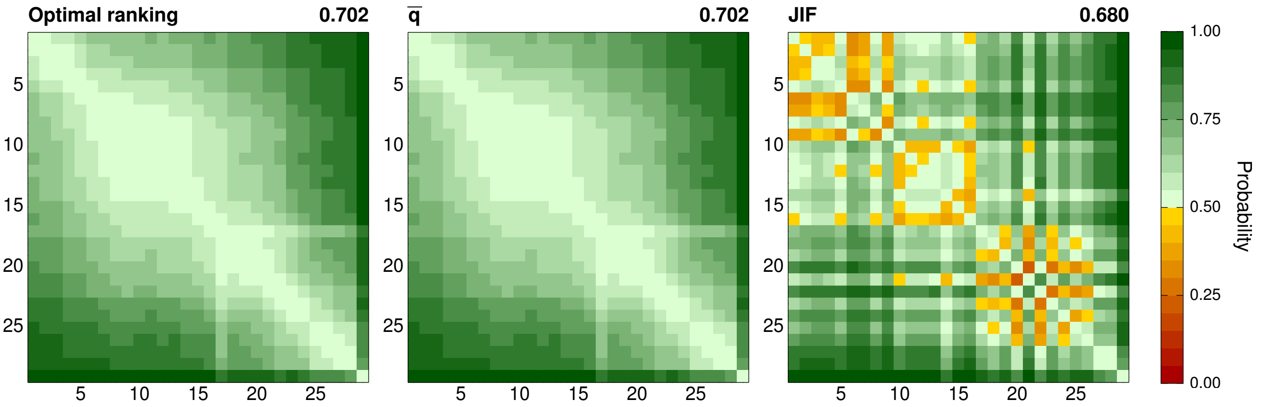

The color of each cell represents the probability of picking a paper with more citations from the higher-ranked journal. Green cells indicate adequate ranking, whereas red cells indicate that the ranking is inadequate (the probability is less than $\frac{1}{2}$). The value of M, the multi-class AUC statistic for each ranking scheme, is above each matrix.

Rank			$p_{ss}(q\|J)$		n			Steady-state
AUC	JIF	Journal abbreviation	$\overline{q}$	σ	$\overline{n}$	Q2	JIF	period
1	6	AM J SCI	1.50	0.43	52.0	28	2.453	1964–1994
2	9	J STRUCT GEOL	1.41	0.38	42.9	24	2.118	1978–1989
3	7	QUATERNARY RES	1.41	0.36	34.5	22	2.319	1973–1997
4	3	GEOL SOC AM BULL	1.37	0.44	35.3	20	2.820	1966–1989
5	4	J GEOPHYS RES	1.32	0.37	27.9	18	2.800	1988–1991
6	2	PRECAMBRIAN RES	1.27	0.35	21.6	15	2.949	1973–1999
7	16	CLAY CLAY MINER	1.22	0.32	20.1	15	1.423	1990–1994
8	1	QUATERNARY SCI REV	1.21	0.40	20.0	14	4.113	1993–1999
9	12	PALAEOGEOGR PALAEOCL	1.19	0.38	20.4	13	1.822	1967–1997
10	5	GEOPHYS RES LETT	1.19	0.37	19.2	13	2.602	1995–1997
11	8	J GEOL SOC LONDON	1.18	0.32	18.2	13	2.287	1992–1999
12	11	MAR GEOL	1.18	0.38	19.4	13	2.029	1964–1999
13	13	J VOLCANOL GEOTH RES	1.16	0.33	17.0	12	1.685	1986–1999
14	15	J GLACIOL	1.15	0.41	17.4	12	1.446	1979–1999
15	21	CAN J EARTH SCI	1.12	0.35	15.8	11	0.943	1985–1994
16	10	J HYDROL	1.07	0.37	13.5	10	2.117	1991–2000
17	14	AAPG BULL	1.04	0.68	21.0	8	1.553	1973–1994
18	23	NEW ZEAL J GEOL GEOP	0.99	0.40	11.5	8	0.822	1966–1996
19	19	GROUND WATER	0.97	0.41	12.6	7	1.117	1984–1998
20	17	GEOL MAG	0.95	0.40	11.9	7	1.376	1966–2001
21	25	J COASTAL RES	0.91	0.43	9.9	6	0.665	1989–1995
22	18	PHOTOGRAMM ENG REM S	0.83	0.51	11.1	5	1.284	1978–1999
23	26	CAN GEOTECH J	0.76	0.40	7.1	4	0.358	1990–1998

table continues on next page . . .

... table continued from previous page

Rank			$p_{ss}(q\|J)$		n			Steady-state
**AUC**	**JIF**	**Journal abbreviation**	$\overline{q}$	σ	$\overline{n}$	**Q2**	**JIF**	**period**
24	24	B SOC GEOL FR	0.68	0.41	6.1	3	0.753	1980–2001
25	20	J AFR EARTH SCI	0.59	0.46	5.1	2	0.982	1986–2000
26	22	ENG GEOL	0.50	0.43	4.0	2	0.900	1967–2004
27	27	J GEOL SOC INDIA	0.48	0.44	4.0	2	0.296	1969–1995
28	28	EARTH MOON PLANETS	0.38	0.45	3.0	1	0.252	1984–2003
29	29	GEOTIMES	-0.47	0.48	0.3	0	0.164	1981–2005

GERIATRICS & GERONTOLOGY

ISI Category Description Geriatrics & Gerontology covers resources on the aged and the aging process. This category includes the clinical, biochemical, histological, and psychological aspects of aging. Coverage also includes specific clinical problems in the treatment of elderly patients, as well as research on the cellular and animal correlates of age and senescence. Resources that focus on the psychological, social, and political aspects of aging are covered in the SSCI.

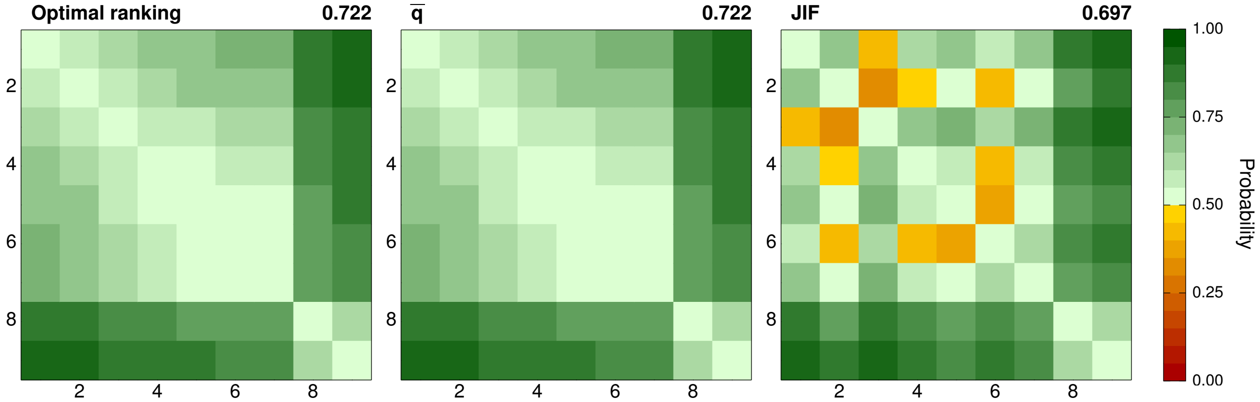

The color of each cell represents the probability of picking a paper with more citations from the higher-ranked journal. Green cells indicate adequate ranking, whereas red cells indicate that the ranking is inadequate (the probability is less than $\frac{1}{2}$). The value of M, the multi-class AUC statistic for each ranking scheme, is above each matrix.

Rank			$p_{ss}(q\|J)$		n			Steady-state
**AUC**	**JIF**	**Journal abbreviation**	$\overline{q}$	σ	$\overline{n}$	**Q2**	**JIF**	**period**
1	3	J AM GERIATR SOC	1.38	0.43	33.6	21	3.331	1984–1996
2	1	NEUROBIOL AGING	1.28	0.35	23.5	17	5.599	1997–2001
3	6	AGE AGEING	1.20	0.40	19.7	13	1.919	1987–1997
4	4	EXP GERONTOL	1.10	0.39	16.0	10	2.930	1967–2001
5	2	MECH AGEING DEV	1.04	0.35	14.0	9	3.846	1989–2002
6	5	INT J GERIATR PSYCH	1.02	0.39	12.9	8	1.930	1986–2001
7	7	GERONTOLOGY	0.99	0.41	13.0	8	1.439	1956–2001
8	8	GERIATRICS	0.50	0.45	4.3	2	1.147	1960–2004
9	9	ARCH GERONTOL GERIAT	0.24	0.64	2.8	0	1.136	1991–2002

GERONTOLOGY

ISI Category Description Gerontology covers resources that are concerned with the sociological and psychological issues of aging, including such areas as rehabilitation, aging and education, aging and work, aging and social policy as well as life span research. Geriatrics, which deals with the medical and clinical aspects of aging, is covered in the SCI.

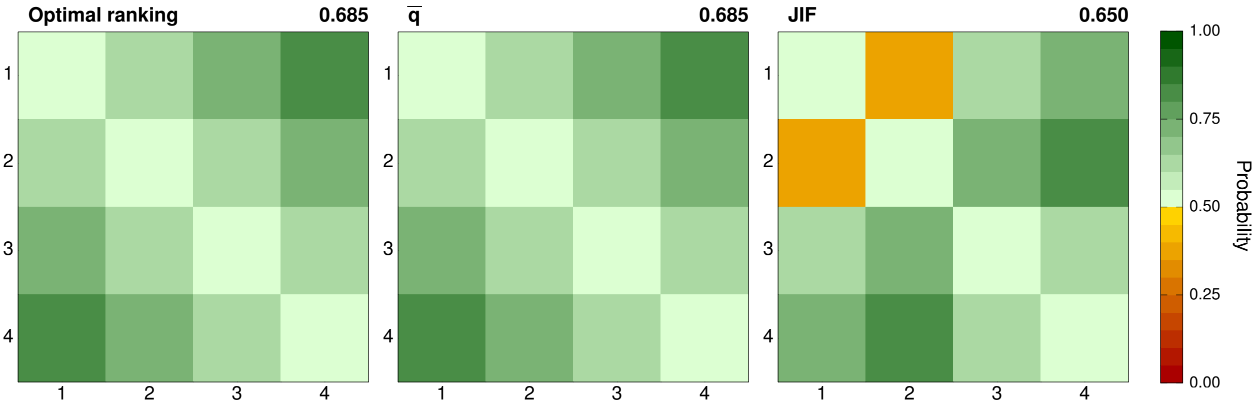

The color of each cell represents the probability of picking a paper with more citations from the higher-ranked journal. Green cells indicate adequate ranking, whereas red cells indicate that the ranking is inadequate (the probability is less than $\frac{1}{2}$). The value of M, the multi-class AUC statistic for each ranking scheme, is above each matrix.

Rank			$p_{ss}(q\|J)$		n			Steady-state
**AUC**	**JIF**	**Journal abbreviation**	$\overline{q}$	σ	$\overline{n}$	**Q2**	**JIF**	**period**
1	2	PSYCHOL AGING	1.52	0.34	42.1	30	2.828	1986–1994
2	1	J AM GERIATR SOC	1.38	0.43	33.6	21	3.331	1984–1996
3	3	GERONTOLOGIST	1.21	0.38	20.9	15	1.965	1994–1998
4	4	INT J GERIATR PSYCH	1.02	0.39	12.9	8	1.930	1986–2001

HEALTH CARE SCIENCES & SERVICES

ISI Category Description Health Care Sciences & Services covers resources on health services, hospital administration, health care management, health care financing, health policy and planning, health economics, health education, history of medicine, and palliative care.

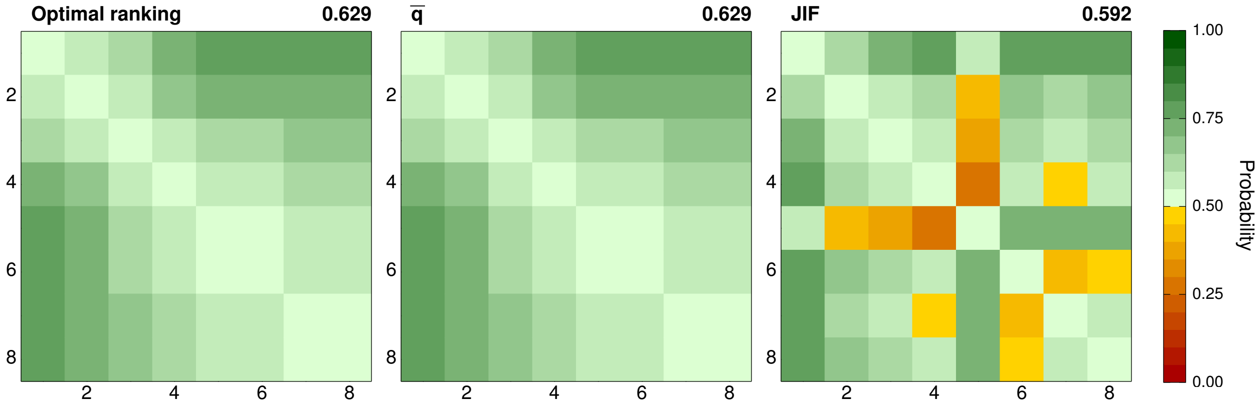

The color of each cell represents the probability of picking a paper with more citations from the higher-ranked journal. Green cells indicate adequate ranking, whereas red cells indicate that the ranking is inadequate (the probability is less than $\frac{1}{2}$). The value of M, the multi-class AUC statistic for each ranking scheme, is above each matrix.

Rank			$p_{ss}(q\|J)$		n			Steady-state
**AUC**	**JIF**	**Journal abbreviation**	$\overline{q}$	σ	$\overline{n}$	**Q2**	**JIF**	**period**
1	1	MED CARE	1.28	0.40	28.6	16	3.745	1994–2000
2	5	J PAIN SYMPTOM MANAG	1.18	0.42	19.5	13	2.437	1990–2000
3	2	HEALTH AFFAIR	1.03	0.46	15.2	8	3.680	1986–2002
4	3	ACAD MED	0.92	0.43	11.3	7	2.607	1988–1998
5	7	HEALTH POLICY	0.84	0.36	8.9	5	1.201	1991–2000
6	4	MED EDUC	0.81	0.43	8.4	5	2.467	1981–2002
7	8	J SCHOOL HEALTH	0.71	0.50	7.8	4	0.856	1983–1999
8	6	HASTINGS CENT REP	0.66	0.61	7.8	3	1.848	1992–1999

HEALTH POLICY & SERVICES

ISI Category Description Health Policy & Services covers resources on healthcare systems, including healthcare provision and management, financial analysis, healthcare ethics, health policy, and quality of care.

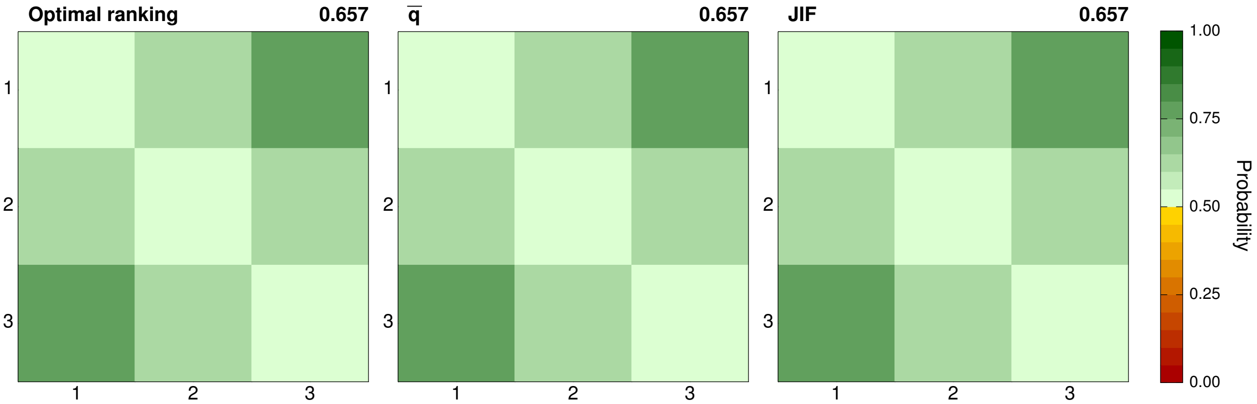

The color of each cell represents the probability of picking a paper with more citations from the higher-ranked journal. Green cells indicate adequate ranking, whereas red cells indicate that the ranking is inadequate (the probability is less than $\frac{1}{2}$). The value of M, the multi-class AUC statistic for each ranking scheme, is above each matrix.

**Rank**			$\mathbf{p_{ss}(q\|J)}$		**n**			**Steady-state**
**AUC**	**JIF**	**Journal abbreviation**	$\overline{\mathbf{q}}$	σ	$\overline{\mathbf{n}}$	**Q2**	**JIF**	**period**
1	1	MED CARE	1.28	0.40	28.6	16	3.745	1994–2000
2	2	HEALTH AFFAIR	1.03	0.46	15.2	8	3.680	1986–2002
3	3	HEALTH POLICY	0.84	0.36	8.9	5	1.201	1991–2000

HEMATOLOGY

ISI Category Description Hematology covers resources that deal with blood and blood-forming tissues, as well as the functions, diseases, and treatments of these systems. Topics included are hemophilia, neoplastic disorders of the blood or lymphoid tissues, and mechanisms and disorders of thrombosis.

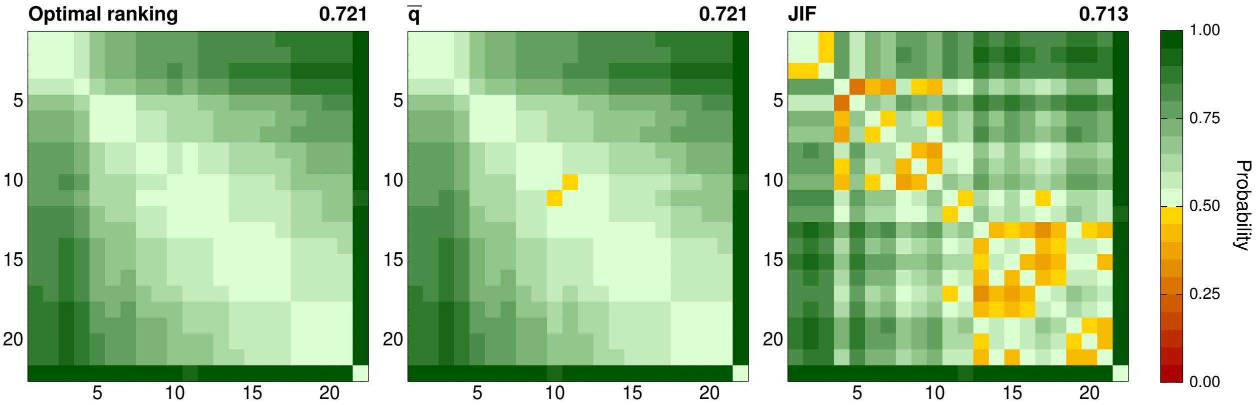

The color of each cell represents the probability of picking a paper with more citations from the higher-ranked journal. Green cells indicate adequate ranking, whereas red cells indicate that the ranking is inadequate (the probability is less than $\frac{1}{2}$). The value of M, the multi-class AUC statistic for each ranking scheme, is above each matrix.

Rank			$p_{ss}(q\|J)$		n			Steady-state
**AUC**	**JIF**	**Journal abbreviation**	$\overline{q}$	σ	$\overline{n}$	**Q2**	**JIF**	**period**
1	3	CIRC RES	1.72	0.46	75.8	47	9.854	1962–1994
2	1	CIRCULATION	1.71	0.46	75.4	45	10.940	1969–1997
3	2	BLOOD	1.68	0.35	63.9	43	10.370	1985–1995
4	5	J CEREBR BLOOD F MET	1.59	0.38	56.6	35	4.843	1982–1996
5	7	BRIT J HAEMATOL	1.41	0.38	36.8	22	4.498	1961–1990
6	10	THROMB HAEMOSTASIS	1.34	0.37	31.1	19	2.803	1980–1998
7	6	J LEUKOCYTE BIOL	1.34	0.34	27.8	18	4.572	1987–1999
8	9	TRANSFUSION	1.26	0.38	23.2	16	3.278	1993–1998
9	4	LEUKEMIA	1.26	0.37	22.5	15	6.146	1995–2000
10	8	EXP HEMATOL	1.18	0.38	20.3	12	3.408	1985–1998
11	12	BONE MARROW TRANSPL	1.21	0.51	20.5	14	2.621	1993–1995
12	17	AM J HEMATOL	1.14	0.36	17.8	12	1.882	1976–1995
13	11	SEMIN THROMB HEMOST	1.10	0.38	17.8	10	2.733	1982–2000
14	18	EUR J HAEMATOL	1.07	0.38	13.7	9	1.863	1986–1997
15	14	ANN HEMATOL	1.05	0.41	14.5	9	2.254	1990–1992
16	16	THROMB RES	1.04	0.39	14.1	9	2.058	1988–1995
17	21	BLOOD COAGUL FIBRIN	1.04	0.37	13.1	9	1.370	1990–1998
18	15	VOX SANG	0.94	0.38	11.1	7	2.111	1993–1999
19	20	LEUKEMIA LYMPHOMA	0.94	0.37	10.1	7	1.559	1992–1997
20	13	LEUKEMIA RES	0.91	0.33	9.4	6	2.483	1996–2000
21	19	ACTA HAEMATOL-BASEL	0.91	0.38	9.7	6	1.564	1960–1997
22	22	GEMATOL TRANSFUZIOL	-0.46	0.40	0.2	0	0.101	1982–2004

HORTICULTURE

ISI Category Description Horticulture covers resources concerning the cultivation of flowers, fruits, vegetables or ornamental plants, in gardens, orchards or nurseries.

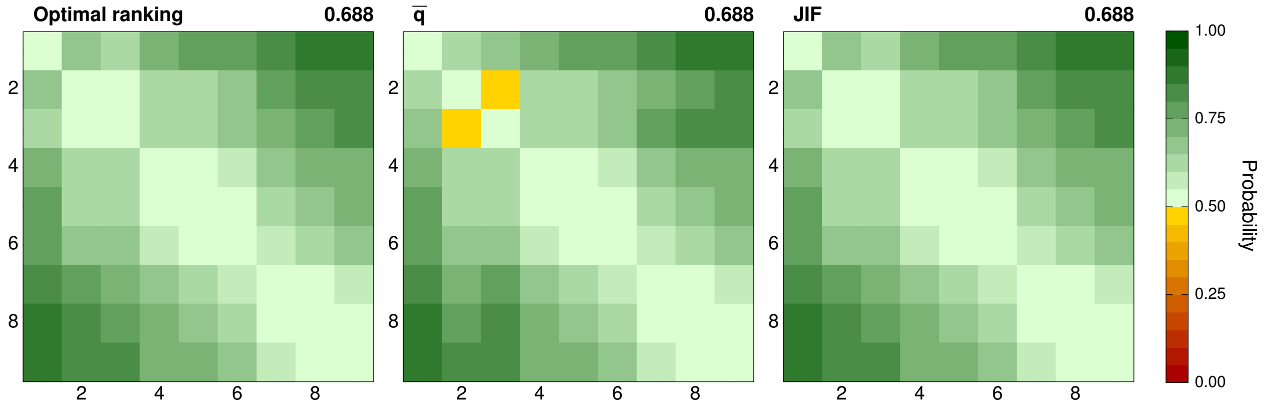

The color of each cell represents the probability of picking a paper with more citations from the higher-ranked journal. Green cells indicate adequate ranking, whereas red cells indicate that the ranking is inadequate (the probability is less than $\frac{1}{2}$). The value of M, the multi-class AUC statistic for each ranking scheme, is above each matrix.

Rank			$p_{ss}(q\|J)$		n			Steady-state
AUC	JIF	Journal abbreviation	$\overline{q}$	σ	$\overline{n}$	Q2	JIF	period
1	1	THEOR APPL GENET	1.25	0.40	23.9	15	2.715	1985–1999
2	2	J AM SOC HORTIC SCI	1.02	0.35	12.2	8	1.043	1968–1995
3	3	AM J ENOL VITICULT	1.03	0.42	13.9	8	1.009	1967–2000
4	4	EUPHYTICA	0.84	0.38	8.0	5	0.907	1984–1998
5	5	SCI HORTIC-AMSTERDAM	0.80	0.37	7.2	5	0.697	1975–1996
6	6	HORTSCIENCE	0.72	0.41	7.1	4	0.613	1975–1995
7	7	SEED SCI TECHNOL	0.55	0.44	4.6	2	0.410	1976–1998
8	8	SYNTHESE	0.46	0.42	3.8	2	0.364	1982–1996
9	9	J JPN SOC HORTIC SCI	0.43	0.40	3.0	2	0.337	1975–2000

IMAGING SCIENCE & PHOTOGRAPHIC TECHNOLOGY

ISI Category Description Imaging Science & Photographic Technology includes resources that cover pattern recognition, analog and digital signal processing, remote sensing, and optical technology. This category also covers resources on the photographic process (the engineering of photographic devices and the chemistry of photography) as well as machine-aided imaging, recording materials and media, and visual communication and image representation.

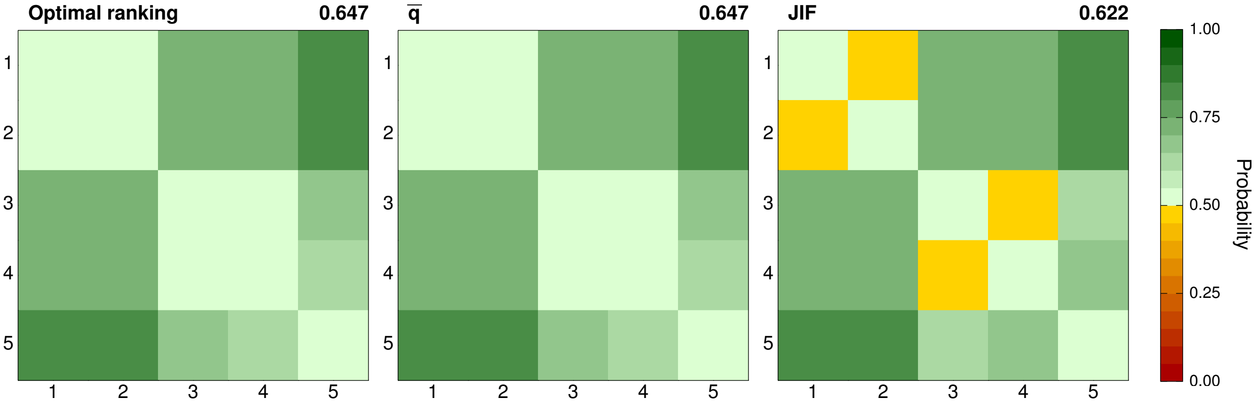

The color of each cell represents the probability of picking a paper with more citations from the higher-ranked journal. Green cells indicate adequate ranking, whereas red cells indicate that the ranking is inadequate (the probability is less than $\frac{1}{2}$). The value of M, the multi-class AUC statistic for each ranking scheme, is above each matrix.

Rank			$p_{ss}(q\|J)$		n			Steady-state
**AUC**	**JIF**	**Journal abbreviation**	$\overline{q}$	σ	$\overline{n}$	**Q2**	**JIF**	**period**
1	2	REMOTE SENS ENVIRON	1.27	0.40	26.7	16	3.064	1980–1998
2	1	IEEE T MED IMAGING	1.25	0.40	27.5	15	3.757	1985–1999
3	4	INT J REMOTE SENS	0.89	0.43	12.3	6	0.980	1982–2000
4	3	PHOTOGRAMM ENG REM S	0.83	0.51	11.1	5	1.284	1978–1999
5	5	J IMAGING SCI TECHN	0.53	0.45	5.0	2	0.622	1991–1992

IMMUNOLOGY

ISI Category Description Immunology covers resources dedicated to all aspects of immune response and regulation, at the cellular-molecular level as well as the clinical level. Other topics include studies of the interaction between pathogens and host immunity, as well as clinical immunology, emerging immunotherapies, and the immunologic contribution to disease course.

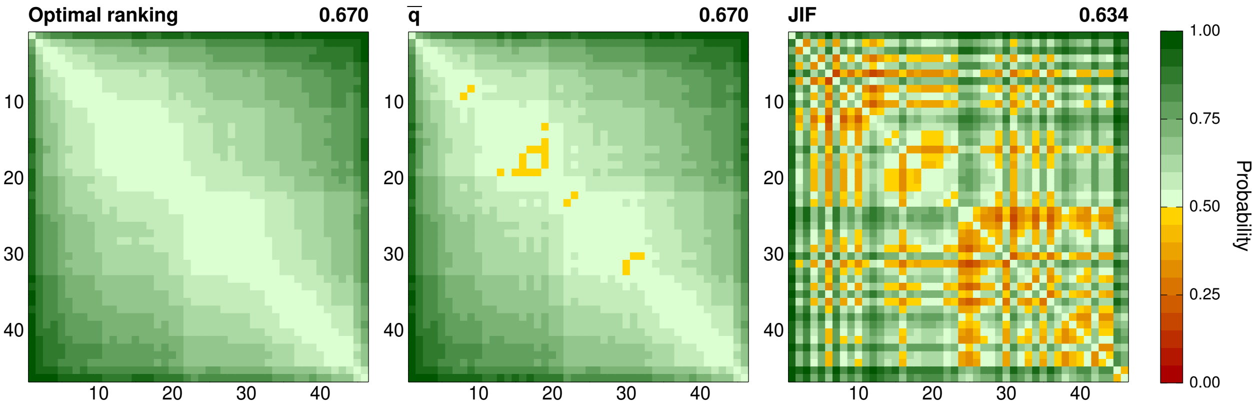

The color of each cell represents the probability of picking a paper with more citations from the higher-ranked journal. Green cells indicate adequate ranking, whereas red cells indicate that the ranking is inadequate (the probability is less than $\frac{1}{2}$). The value of M, the multi-class AUC statistic for each ranking scheme, is above each matrix.

Rank			$p_{ss}(q\|J)$		n			Steady-state
AUC	JIF	Journal abbreviation	$\bar{q}$	σ	$\bar{n}$	Q2	JIF	period
1	1	J EXP MED	1.97	0.36	132.9	86	14.484	1987–1995
2	3	J IMMUNOL	1.66	0.36	62.9	41	6.293	1988–1995
3	7	EUR J IMMUNOL	1.56	0.38	48.7	32	4.772	1988–1993
4	12	INFECT IMMUN	1.52	0.33	40.4	30	4.004	1985–1995
5	5	AIDS	1.49	0.38	37.7	27	5.632	1987–1999
6	13	TRANSPLANTATION	1.42	0.38	35.2	22	3.972	1981–1995
7	31	MICROB PATHOGENESIS	1.39	0.38	30.6	22	2.258	1985–1988
8	11	INT IMMUNOL	1.39	0.36	33.3	21	4.015	1989–1995
9	2	J ALLERGY CLIN IMMUN	1.39	0.40	30.8	21	8.829	1994–1999
10	9	J LEUKOCYTE BIOL	1.34	0.34	27.8	18	4.572	1987–1999
11	19	J NEUROIMMUNOL	1.32	0.38	27.4	18	2.880	1980–1999
12	20	IMMUNOGENETICS	1.32	0.37	27.7	18	2.852	1976–1995
13	29	CYTOKINE	1.25	0.36	26.1	16	2.355	1990–1994
14	21	CLIN EXP IMMUNOL	1.29	0.36	25.1	16	2.747	1980–1997
15	36	PARASITE IMMUNOL	1.29	0.33	22.5	17	2.009	1979–1995
16	34	ORAL MICROBIOL IMMUN	1.28	0.32	21.6	16	2.089	1987–1993
17	15	CLIN EXP ALLERGY	1.27	0.28	30.1	15	3.668	1988–1989
18	14	IMMUNOLOGY	1.27	0.39	26.3	16	3.674	1970–1997
19	17	PEDIATR INFECT DIS J	1.28	0.38	23.1	16	3.215	1990–1995
20	18	VACCINE	1.23	0.36	20.9	14	3.159	1987–1998
21	23	HUM IMMUNOL	1.22	0.38	22.9	14	2.605	1979–1995
22	4	CLIN INFECT DIS	1.21	0.46	23.6	13	6.186	1995–1998
23	22	BONE MARROW TRANSPL	1.21	0.51	20.5	14	2.621	1993–1995

table continues on next page . . .

... table continued from previous page

**Rank**			$\mathbf{p_{ss}(q\|J)}$		**n**			**Steady-state**
**AUC**	**JIF**	**Journal abbreviation**	$\overline{\mathbf{q}}$	σ	$\overline{\mathbf{n}}$	**Q2**	**JIF**	**period**
24	28	J IMMUNOL METHODS	1.16	0.41	28.4	11	2.402	1971–1997
25	41	CELL IMMUNOL	1.15	0.38	18.9	12	1.709	1979–2000
26	32	J AUTOIMMUN	1.14	0.36	17.0	12	2.154	1987–2001
27	8	MOL IMMUNOL	1.14	0.37	17.9	11	4.768	1978–1999
28	27	TISSUE ANTIGENS	1.14	0.40	20.2	11	2.462	1971–1999
29	44	J ANTIBIOT	1.13	0.37	18.6	11	1.262	1963–1992
30	10	CANCER IMMUNOL IMMUN	1.11	0.33	15.9	11	4.313	1982–2000
31	43	CAN J MICROBIOL	1.10	0.39	17.3	11	1.275	1954–1995
32	40	AM J REPROD IMMUNOL	1.11	0.39	16.1	10	1.743	1979–1999
33	37	VET IMMUNOL IMMUNOP	1.07	0.35	13.3	10	1.994	1979–1999
34	16	DEV COMP IMMUNOL	1.06	0.41	14.2	9	3.399	1976–2001
35	33	SCAND J IMMUNOL	1.03	0.37	13.2	8	2.090	1995–1999
36	6	ALLERGY	1.02	0.38	12.7	8	5.334	1995–2001
37	39	IMMUNOBIOLOGY	0.98	0.41	13.7	8	1.867	1980–1997
38	26	IMMUNOL CELL BIOL	0.97	0.40	11.8	7	2.482	1986–2002
39	35	AUTOIMMUNITY	0.96	0.36	10.8	7	2.033	1988–1999
40	30	IMMUNOL LETT	0.93	0.38	11.1	7	2.352	1978–2001
41	24	INT ARCH ALLERGY IMM	0.90	0.40	10.9	6	2.524	1991–1992
42	38	APMIS	0.86	0.40	8.8	5	1.875	1987–1999
43	42	MICROBIOL IMMUNOL	0.85	0.36	7.8	5	1.502	1996–2000
44	25	AIDS RES HUM RETROV	0.81	0.41	9.2	4	2.513	1985–1986
45	46	HYBRIDOMA	0.69	0.56	7.0	3	0.411	1986–2000
46	45	INDIAN J MED RES	0.55	0.37	3.8	2	1.224	1988–2000

INDUSTRIAL RELATIONS & LABOR

ISI Category Description Industrial Relations & Labor covers resources on arbitration, business and labor law, human resources, labor history, labor relations, and the sociology of work relations.

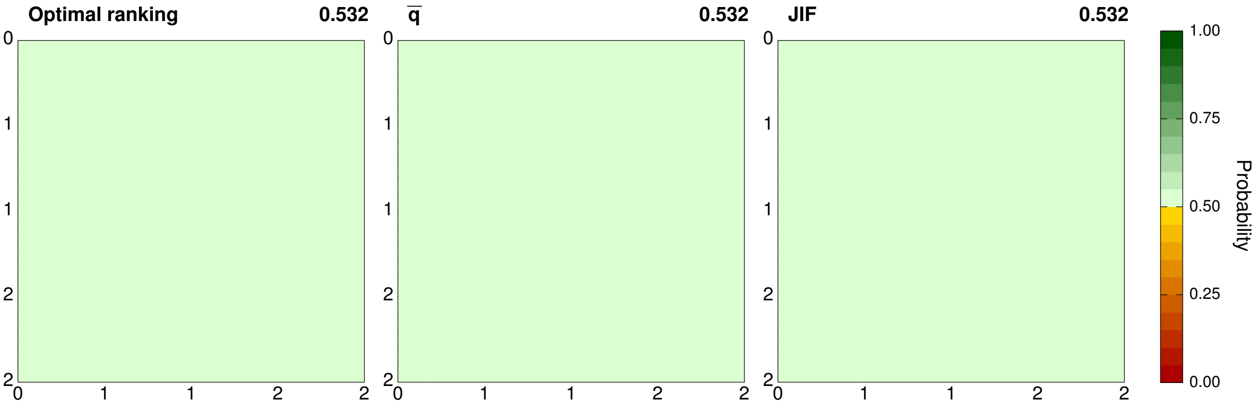

The color of each cell represents the probability of picking a paper with more citations from the higher-ranked journal. Green cells indicate adequate ranking, whereas red cells indicate that the ranking is inadequate (the probability is less than $\frac{1}{2}$). The value of M, the multi-class AUC statistic for each ranking scheme, is above each matrix.

Rank			$p_{ss}(q\|J)$		n			Steady-state
AUC	JIF	Journal abbreviation	$\overline{q}$	σ	$\overline{n}$	Q2	JIF	period
1	1	BUS LAWYER	0.32	0.40	2.6	1	0.685	1981–2003
2	2	MON LABOR REV	0.28	0.54	2.8	1	0.589	1971–2001

INFECTIOUS DISEASES

ISI Category Description Infectious Diseases covers resources on all aspects of the pathogenesis of clinically significant viral or bacterial diseases including HIV, AIDS, sexually transmitted diseases (STDs). This category is also concerned with resources on host-pathogen interactions, as well as the prevention, diagnosis, treatment, and epidemiology of infectious disease.

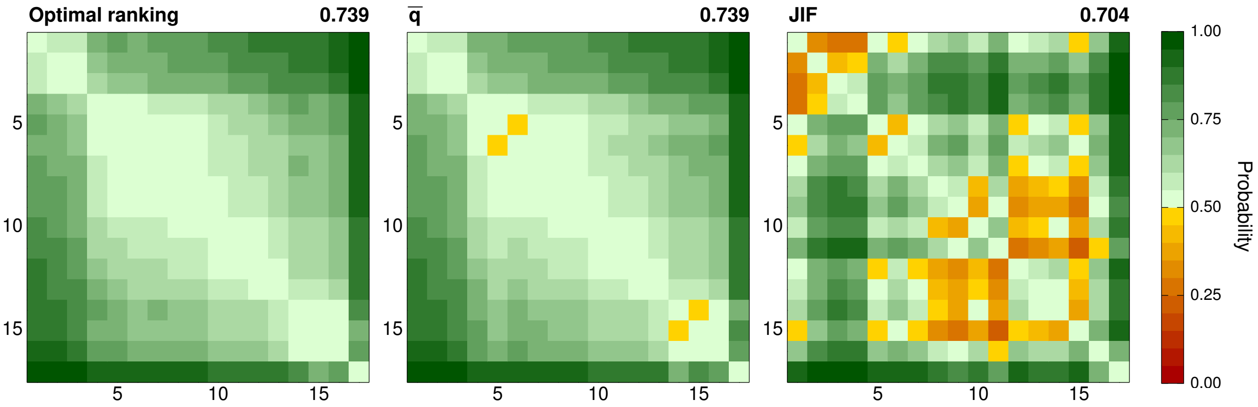

The color of each cell represents the probability of picking a paper with more citations from the higher-ranked journal. Green cells indicate adequate ranking, whereas red cells indicate that the ranking is inadequate (the probability is less than $\frac{1}{2}$). The value of M, the multi-class AUC statistic for each ranking scheme, is above each matrix.

**Rank**			$\mathbf{p_{ss}(q\|J)}$		**n**			**Steady-state**
**AUC**	**JIF**	**Journal abbreviation**	$\overline{\mathbf{q}}$	σ	$\overline{\mathbf{n}}$	**Q2**	**JIF**	**period**
1	3	J INFECT DIS	1.61	0.36	53.5	37	5.363	1978–1995
2	4	INFECT IMMUN	1.52	0.33	40.4	30	4.004	1985–1995
3	2	AIDS	1.49	0.38	37.7	27	5.632	1987–1999
4	6	PEDIATR INFECT DIS J	1.28	0.38	23.1	16	3.215	1990–1995
5	15	EPIDEMIOL INFECT	1.20	0.34	20.1	13	1.809	1988–1997
6	1	CLIN INFECT DIS	1.21	0.46	23.6	13	6.186	1995–1998
7	12	EUR J CLIN MICROBIOL	1.17	0.37	19.0	12	2.330	1982–1996
8	5	J ANTIMICROB CHEMOTH	1.16	0.37	18.3	12	3.891	1976–2000
9	7	SEX TRANSM DIS	1.14	0.39	17.2	11	2.577	1978–2000
10	13	INFECT CONT HOSP EP	1.08	0.45	17.6	10	2.236	1979–2000
11	10	J HOSP INFECT	1.05	0.39	13.4	9	2.442	1982–1999
12	14	J INFECTION	1.00	0.38	13.1	8	2.037	1978–1995
13	8	DIAGN MICR INFEC DIS	0.94	0.37	11.4	7	2.553	1982–2002
14	9	AIDS RES HUM RETROV	0.81	0.41	9.2	4	2.513	1985–1986
15	16	SCAND J INFECT DIS	0.84	0.67	9.6	5	1.560	1973–1996
16	11	INFECTION	0.75	0.46	7.5	4	2.368	1972–2000
17	17	MED MALADIES INFECT	0.12	0.46	1.5	0	0.267	1976–2002

INFORMATION SCIENCE & LIBRARY SCIENCE

ISI Category Description Information Science & Library Science covers resources on a wide variety of topics, including bibliographic studies, cataloguing, categorization, database construction and maintenance, electronic libraries, information ethics, information processing and management, interlending, preservation, scientometrics, serials librarianship, and special libraries.

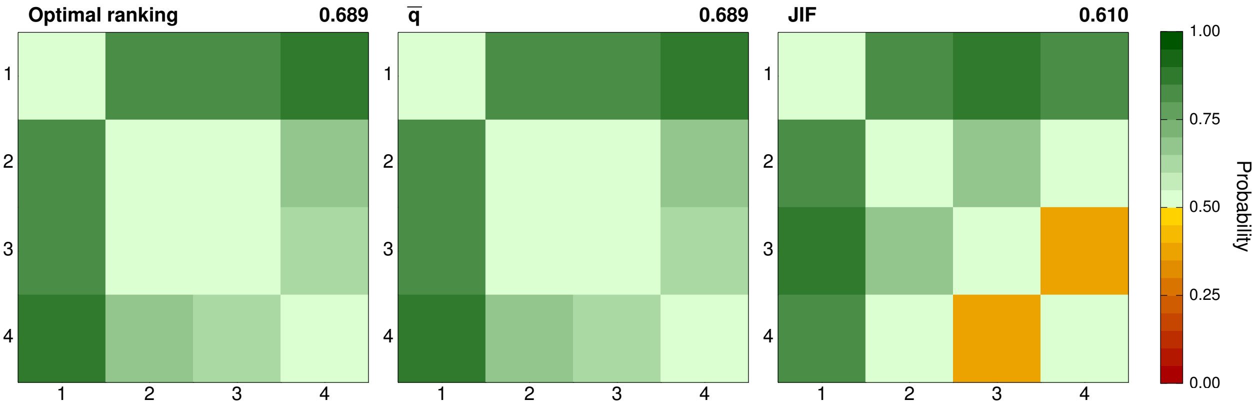

The color of each cell represents the probability of picking a paper with more citations from the higher-ranked journal. Green cells indicate adequate ranking, whereas red cells indicate that the ranking is inadequate (the probability is less than $\frac{1}{2}$). The value of M, the multi-class AUC statistic for each ranking scheme, is above each matrix.

Rank			$\mathbf{p_{ss}(q\|J)}$		n			Steady-state
**AUC**	**JIF**	**Journal abbreviation**	$\overline{\mathbf{q}}$	σ	$\overline{\mathbf{n}}$	**Q2**	**JIF**	**period**
1	1	SCIENTOMETRICS	0.72	0.39	6.2	4	1.363	1989–2002
2	2	ONLINE	0.05	0.43	1.2	0	0.484	1988–2005
3	4	LIBR J	0.04	0.47	1.2	0	0.271	1970–2000
4	3	SCIENTIST	-0.66	0.72	0.4	0	0.296	1992–1999

INSTRUMENTS & INSTRUMENTATION

ISI Category Description Instruments & Instrumentation includes resources on the application of instruments for observation, measurement, or control of physical and/or chemical systems. This category also includes materials on the development and manufacture of instruments.

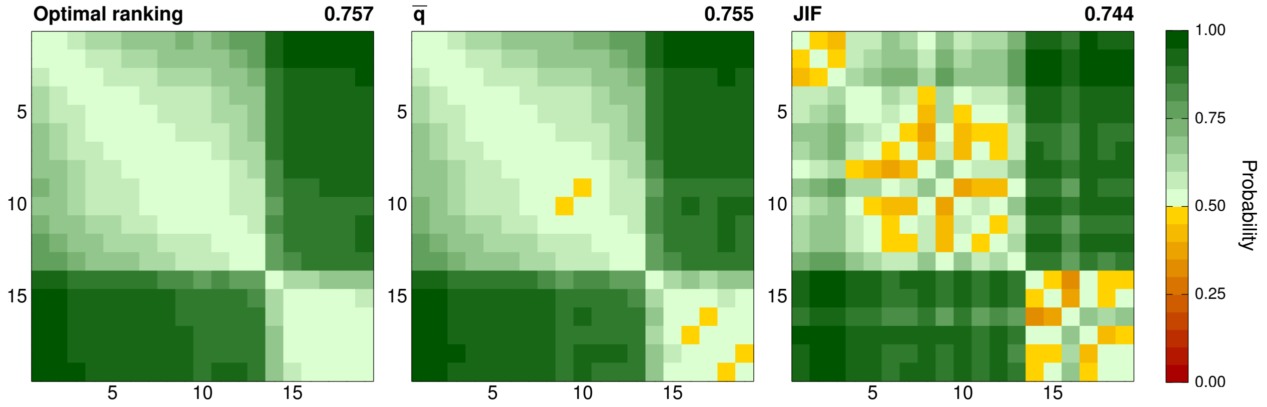

The color of each cell represents the probability of picking a paper with more citations from the higher-ranked journal. Green cells indicate adequate ranking, whereas red cells indicate that the ranking is inadequate (the probability is less than $\frac{1}{2}$). The value of M, the multi-class AUC statistic for each ranking scheme, is above each matrix.

Rank			$p_{ss}(q\|J)$		n			Steady-state
AUC	JIF	Journal abbreviation	$\bar{q}$	σ	$\bar{n}$	Q2	JIF	period
1	3	APPL SPECTROSC	1.08	0.38	16.2	10	1.879	1970–1998
2	2	SENSOR ACTUAT B-CHEM	1.02	0.38	12.2	8	2.331	1995–2001
3	1	CHEMOMETR INTELL LAB	0.96	0.42	14.8	8	2.450	1986–2002
4	8	RADIO SCI	0.91	0.45	12.2	6	1.084	1965–1997
5	4	REV SCI INSTRUM	0.88	0.45	12.9	5	1.541	1954–1993
6	10	NUCL INSTRUM METH B	0.82	0.39	8.7	5	0.946	1988–1997
7	5	SENSOR ACTUAT A-PHYS	0.80	0.38	7.7	5	1.434	1997–2001
8	12	IEEE T IND ELECTRON	0.76	0.43	7.7	4	0.590	1988–1998
9	7	NUCL INSTRUM METH A	0.73	0.44	8.3	4	1.185	1989–1994
10	11	J DYN SYST-T ASME	0.75	0.52	9.1	4	0.658	1982–1993
11	6	MEAS SCI TECHNOL	0.68	0.42	6.5	3	1.228	1989–2000
12	9	J GUID CONTROL DYNAM	0.62	0.39	5.0	3	0.986	1992–2000
13	13	IEEE T INSTRUM MEAS	0.55	0.44	4.8	2	0.572	1963–2001
14	16	AM LAB	-0.02	0.44	1.0	0	0.220	2000–2003
15	18	CONTROL ENG	-0.37	0.38	0.2	0	0.051	1966–2005
16	15	AUTOMAT REM CONTR+	-0.51	0.44	0.2	0	0.251	1990–2005
17	19	INTECH	-0.45	0.34	0.1	0	0.031	1985–2001
18	14	INSTRUM EXP TECH+	-0.70	0.53	0.1	0	0.329	1963–2005
19	17	MEAS TECH+	-0.70	0.38	0.0	0	0.129	1971–2003

INTERNATIONAL RELATIONS

ISI Category Description International Relations covers resources concerned with foreign policy, comparative world politics, world commerce and trade, international legal issues, peace studies and conflict resolution, military alliances, and strategic studies.

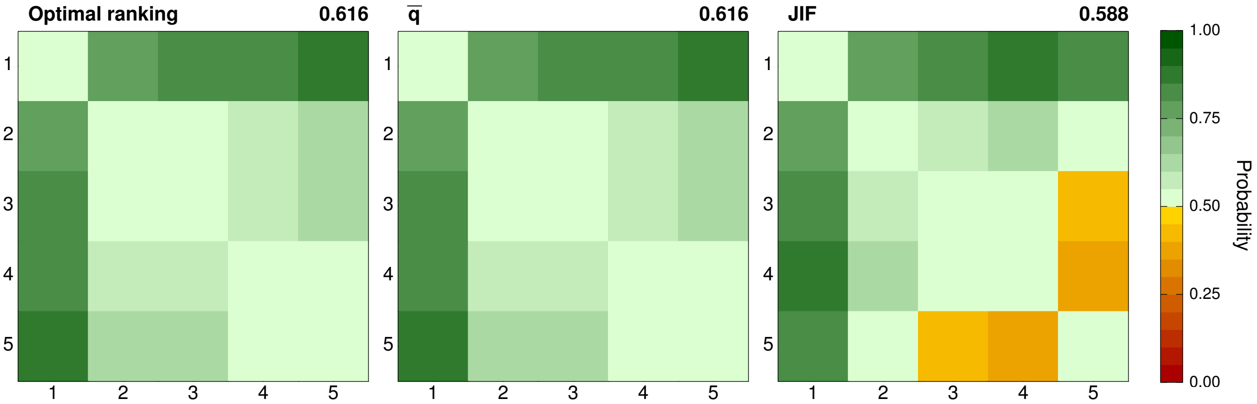

The color of each cell represents the probability of picking a paper with more citations from the higher-ranked journal. Green cells indicate adequate ranking, whereas red cells indicate that the ranking is inadequate (the probability is less than $\frac{1}{2}$). The value of M, the multi-class AUC statistic for each ranking scheme, is above each matrix.

Rank			$p_{ss}(q\|J)$		n			Steady-state
**AUC**	**JIF**	**Journal abbreviation**	$\overline{q}$	σ	$\overline{n}$	**Q2**	**JIF**	**period**
1	1	FOREIGN AFF	0.66	0.41	8.3	3	1.814	1992–1999
2	2	WASH QUART	0.03	0.40	1.0	0	0.775	1982–2005
3	5	CURR HIST	0.01	0.33	0.7	0	0.176	1993–2000
4	3	B ATOM SCI	-0.18	0.39	0.5	0	0.397	1978–2006
5	4	ISSUES STUD	-0.25	0.41	0.4	0	0.310	1967–2005

LAW

ISI Category Description Law covers resources from both general and specialized areas of national and international law, including comparative law, criminology, business law, banking, corporate and tax law, constitutional law, civil rights, copyright and intellectual property law, environmental law, family law, medicine and the law as well as psychology and the law.

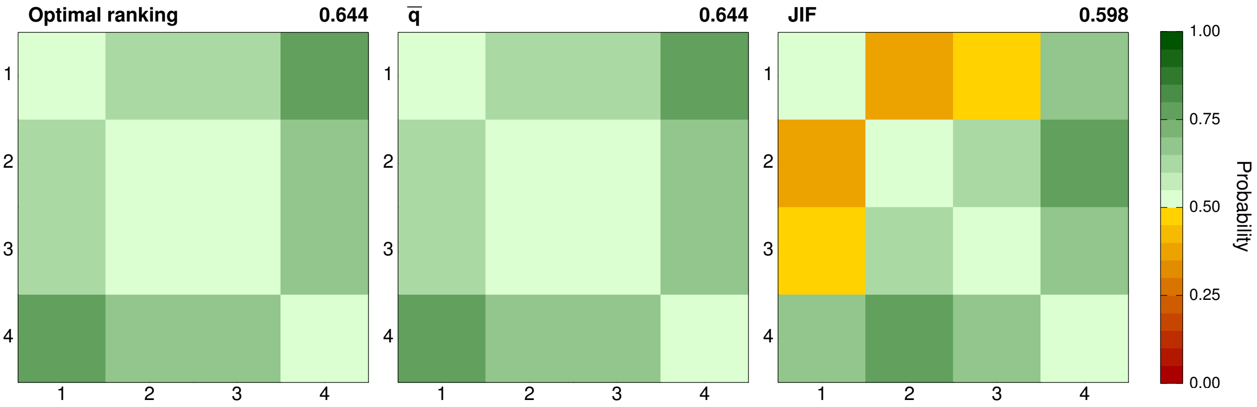

The color of each cell represents the probability of picking a paper with more citations from the higher-ranked journal. Green cells indicate adequate ranking, whereas red cells indicate that the ranking is inadequate (the probability is less than $\frac{1}{2}$). The value of M, the multi-class AUC statistic for each ranking scheme, is above each matrix.

**Rank**			$\mathbf{p_{ss}(q\|J)}$		**n**			**Steady-state**
**AUC**	**JIF**	**Journal abbreviation**	$\overline{\mathbf{q}}$	σ	$\overline{\mathbf{n}}$	**Q2**	**JIF**	**period**
1	2	BUS LAWYER	0.32	0.40	2.6	1	0.685	1981–2003
2	3	FOOD DRUG LAW J	0.04	0.48	1.2	0	0.397	1972–2005
3	1	WASH QUART	0.03	0.40	1.0	0	0.775	1982–2005
4	4	ABA J	-0.39	0.32	0.1	0	0.356	1999–2006

LIMNOLOGY

ISI Category Description Limnology covers resources concerning the study of the physical, chemical, meteorological, biological and ecological aspects of freshwaters.

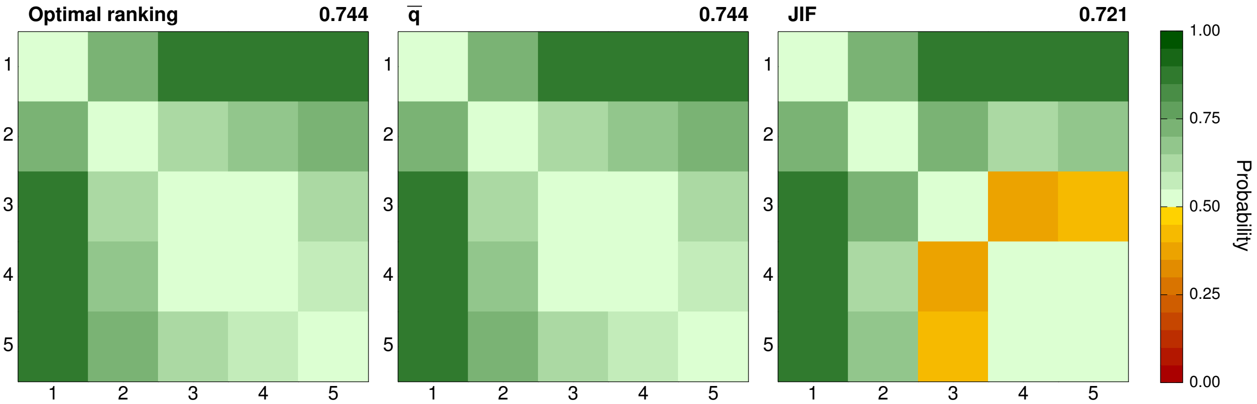

The color of each cell represents the probability of picking a paper with more citations from the higher-ranked journal. Green cells indicate adequate ranking, whereas red cells indicate that the ranking is inadequate (the probability is less than $\frac{1}{2}$). The value of M, the multi-class AUC statistic for each ranking scheme, is above each matrix.

Rank			$\mathbf{p_{ss}(q\|J)}$		n			Steady-state
**AUC**	**JIF**	**Journal abbreviation**	$\overline{\mathbf{q}}$	σ	$\overline{\mathbf{n}}$	**Q2**	**JIF**	**period**
1	1	LIMNOL OCEANOGR	1.71	0.36	65.0	47	3.287	1970–1992
2	2	WATER RESOUR RES	1.34	0.44	33.2	19	1.894	1981–1992
3	4	J GREAT LAKES RES	1.12	0.38	16.1	11	1.000	1982–1995
4	5	WATER ENVIRON RES	1.07	0.44	14.0	9	0.665	1991–1996
5	3	ARCH HYDROBIOL	0.95	0.44	12.0	7	1.362	1967–1999

MANAGEMENT

ISI Category Description Management covers resources on management science, organization studies, strategic planning and decision-making methods, leadership studies, and total quality management.

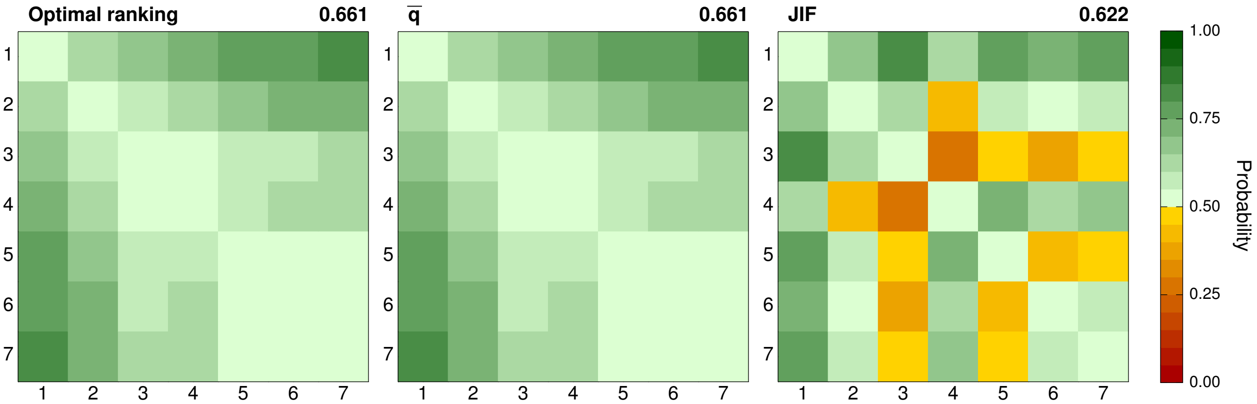

The color of each cell represents the probability of picking a paper with more citations from the higher-ranked journal. Green cells indicate adequate ranking, whereas red cells indicate that the ranking is inadequate (the probability is less than $\frac{1}{2}$). The value of M, the multi-class AUC statistic for each ranking scheme, is above each matrix.

Rank			$\mathbf{p_{ss}(q\|J)}$		n			Steady-state
**AUC**	**JIF**	**Journal abbreviation**	$\overline{\mathbf{q}}$	σ	$\overline{\mathbf{n}}$	**Q2**	**JIF**	**period**
1	1	MANAGE SCI	1.17	0.48	27.1	12	1.687	1957–1997
2	4	HUM RELAT	0.96	0.42	14.4	7	0.670	1955–2000
3	2	HARVARD BUS REV	0.79	0.60	18.3	4	1.505	1987–1997
4	6	J OPER RES SOC	0.74	0.42	7.8	4	0.597	1977–1997
5	7	INTERFACES	0.58	0.47	6.1	2	0.338	1973–2001
6	5	OMEGA-INT J MANAGE S	0.56	0.44	5.3	2	0.663	1974–2003
7	3	LONG RANGE PLANN	0.52	0.40	4.0	2	0.982	1989–2001

MARINE & FRESHWATER BIOLOGY

ISI Category Description Marine & Freshwater Biology covers resources concerning many aquatic sciences, including marine ecology and environmental research, aquatic biology, marine pollution and toxicology, aquatic botany and plant management, estuarine and coastal research, diseases of aquatic organisms, molluscan and shellfish research, fish biology and biofouling.

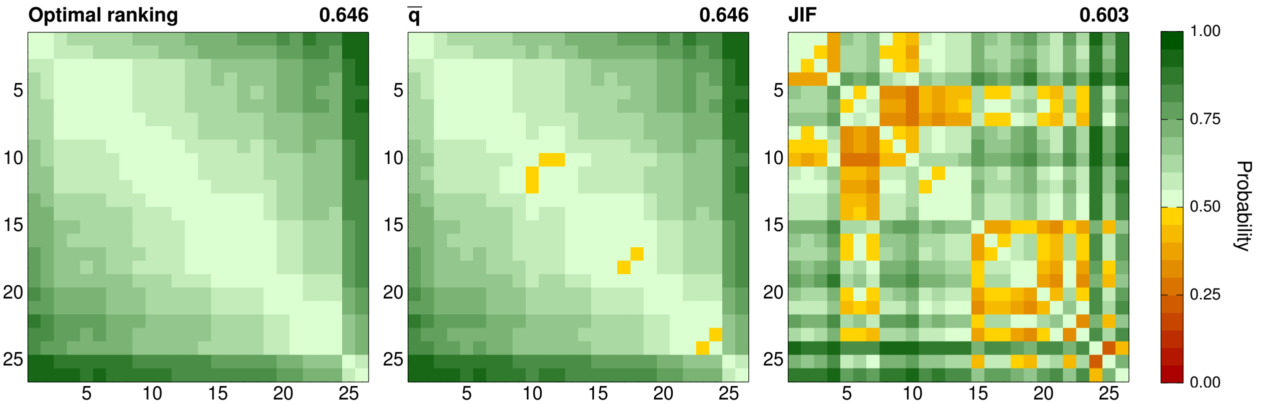

The color of each cell represents the probability of picking a paper with more citations from the higher-ranked journal. Green cells indicate adequate ranking, whereas red cells indicate that the ranking is inadequate (the probability is less than $\frac{1}{2}$). The value of M, the multi-class AUC statistic for each ranking scheme, is above each matrix.

Rank			$p_{ss}(q\|J)$		n			Steady-state
**AUC**	**JIF**	**Journal abbreviation**	$\overline{q}$	σ	$\overline{n}$	**Q2**	**JIF**	**period**
1	4	MAR ECOL-PROG SER	1.47	0.31	33.6	26	2.286	1991–1995
2	10	MAR BIOL	1.45	0.37	35.9	24	1.756	1973–1987
3	9	CAN J FISH AQUAT SCI	1.35	0.34	28.4	20	1.882	1988–1993
4	1	AQUAT TOXICOL	1.32	0.32	26.1	19	2.964	1982–1996
5	3	FRESHWATER BIOL	1.32	0.35	25.3	18	2.502	1972–1997
6	8	J EXP MAR BIOL ECOL	1.31	0.30	23.5	18	1.919	1988–1995
7	2	J PHYCOL	1.30	0.35	26.0	17	2.580	1964–1997
8	12	J PLANKTON RES	1.30	0.35	23.9	17	1.617	1985–1995
9	11	ESTUAR COAST SHELF S	1.25	0.35	21.2	15	1.733	1980–1997
10	13	ESTUARIES	1.24	0.36	21.7	14	1.563	1978–1997
11	14	J FISH BIOL	1.23	0.37	21.1	14	1.393	1968–1993
12	21	ENVIRON BIOL FISH	1.24	0.42	21.0	15	0.934	1981–1990
13	23	J MAR BIOL ASSOC UK	1.17	0.35	19.1	12	0.778	1983–1995
14	6	AQUACULTURE	1.16	0.33	16.0	12	2.081	1993–1999
15	20	J GREAT LAKES RES	1.12	0.38	16.1	11	1.000	1982–1995
16	17	B MAR SCI	1.12	0.39	17.9	11	1.093	1965–1995
17	16	AQUAT BOT	1.11	0.30	13.8	11	1.338	1994–1999
18	5	MAR ENVIRON RES	1.11	0.37	15.7	10	2.106	1977–1997
19	7	MAR POLLUT BULL	1.08	0.34	14.0	10	2.007	1995–2000
20	25	J CRUSTACEAN BIOL	1.03	0.35	12.1	9	0.608	1981–1995
21	18	HYDROBIOLOGIA	1.02	0.40	13.7	8	1.049	1984–1990
22	19	BOT MAR	0.97	0.34	9.6	7	1.009	1992–1996
23	22	NEW ZEAL J MAR FRESH	0.94	0.37	10.7	7	0.931	1976–1998

table continues on next page . . .

. . . table continued from previous page

Rank			$\mathbf{p_{ss}(q\|J)}$		n			Steady-state
**AUC**	**JIF**	**Journal abbreviation**	$\overline{\mathbf{q}}$	σ	$\overline{\mathbf{n}}$	**Q2**	**JIF**	**period**
24	15	ARCH HYDROBIOL	0.95	0.44	12.0	7	1.362	1967–1999
25	26	CRUSTACEANA	0.66	0.39	5.3	3	0.421	1975–1999
26	24	ACTA HYDROCH HYDROB	0.55	0.43	4.5	2	0.632	1991–2001

MATERIALS SCIENCE, BIOMATERIALS

ISI Category Description Materials Science, Biomaterials includes resources that analyze the physical characteristics of living tissue to aid in the development of synthetic replacements for repairs or augmentation of functions. Resources in this category cover the development, testing, performance, and biocompatibility of engineered biomaterials in vitro and in vivo for purposes such as medical implants, devices, and sensors.

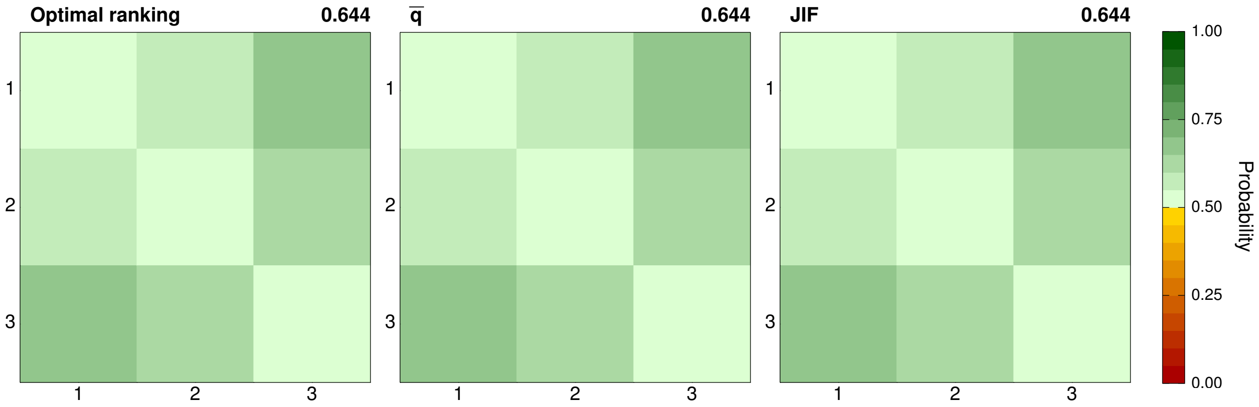

The color of each cell represents the probability of picking a paper with more citations from the higher-ranked journal. Green cells indicate adequate ranking, whereas red cells indicate that the ranking is inadequate (the probability is less than $\frac{1}{2}$). The value of M, the multi-class AUC statistic for each ranking scheme, is above each matrix.

**Rank**			$\mathbf{p_{ss}(q\|J)}$		**n**			**Steady-state**
**AUC**	**JIF**	**Journal abbreviation**	$\overline{\mathbf{q}}$	σ	$\overline{\mathbf{n}}$	**Q2**	**JIF**	**period**
1	1	BIOMATERIALS	1.31	0.35	25.7	18	5.196	1991–2000
2	2	DENT MATER	1.18	0.37	19.1	12	2.381	1987–2001
3	3	J MATER SCI-MATER M	1.01	0.41	14.1	8	1.562	1990–1999

MATERIALS SCIENCE, CERAMICS

ISI Category Description Materials Science, Ceramics covers resources that deal with inorganic materials with high-temperature melting points, including silicates and aluminosilicates, refractory metal oxides and metal nitrides, and borides. This category also includes resources discussing products such as earthenware, porcelain, brick, glass, and vitreous enamels.

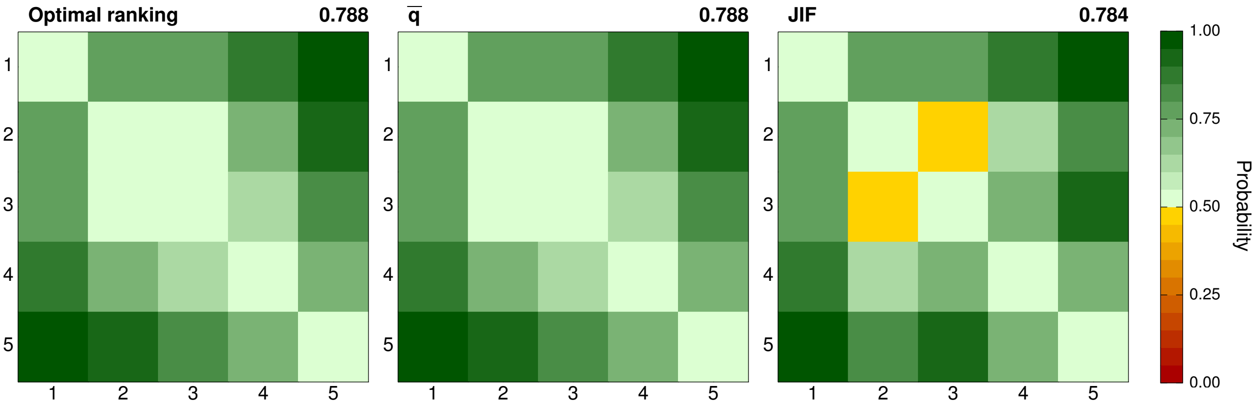

The color of each cell represents the probability of picking a paper with more citations from the higher-ranked journal. Green cells indicate adequate ranking, whereas red cells indicate that the ranking is inadequate (the probability is less than $\frac{1}{2}$). The value of M, the multi-class AUC statistic for each ranking scheme, is above each matrix.

Rank			$\mathbf{p_{ss}(q\|J)}$		n			Steady-state
**AUC**	**JIF**	**Journal abbreviation**	$\overline{\mathbf{q}}$	σ	$\overline{\mathbf{n}}$	**Q2**	**JIF**	**period**
1	1	J AM CERAM SOC	1.29	0.43	29.8	16	1.396	1956–1994
2	3	CERAM INT	0.75	0.36	6.7	4	1.128	1985–2000
3	2	J NON-CRYST SOLIDS	0.73	0.62	8.1	3	1.362	1995–1998
4	4	AM CERAM SOC BULL	0.27	0.66	4.8	0	0.210	1992–2000
5	5	GLASS CERAM+	-0.75	0.55	0.1	0	0.123	1999–2002

MATERIALS SCIENCE, CHARACTERIZATION & TESTING

ISI Category Description Materials Science, Characterization & Testing covers resources that focus on techniques used to evaluate and test materials. These techniques include nondestructive testing, diffraction analysis, electron microscopy, electron spectroscopy, ion beam analysis, mechanical testing, optical characterization, and scanning tunneling microscopy.

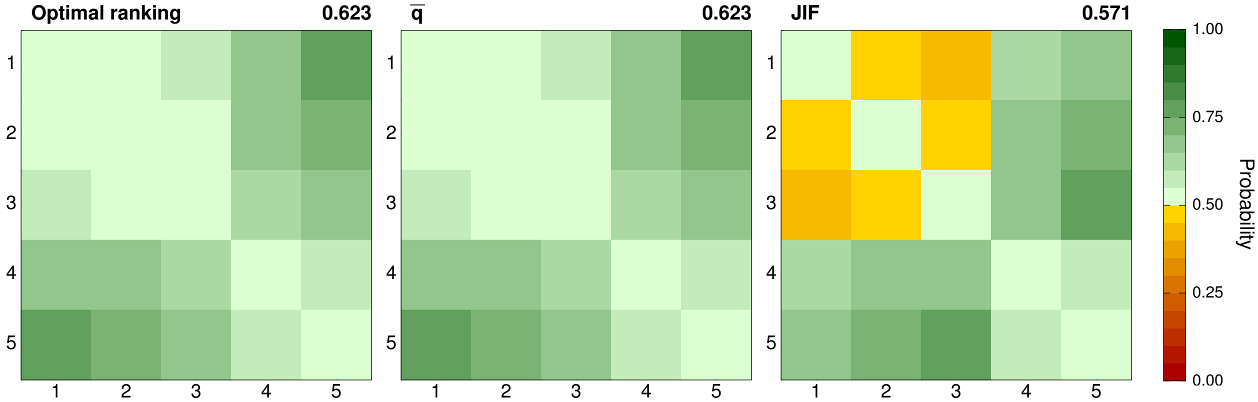

The color of each cell represents the probability of picking a paper with more citations from the higher-ranked journal. Green cells indicate adequate ranking, whereas red cells indicate that the ranking is inadequate (the probability is less than $\frac{1}{2}$). The value of M, the multi-class AUC statistic for each ranking scheme, is above each matrix.

**Rank**			$\mathbf{p_{ss}(q\|J)}$		**n**			**Steady-state**
**AUC**	**JIF**	**Journal abbreviation**	$\overline{\mathbf{q}}$	σ	$\overline{\mathbf{n}}$	**Q2**	**JIF**	**period**
1	3	J TEST EVAL	0.40	0.48	4.0	1	0.229	1973–2000
2	2	ARCH MECH	0.30	0.47	3.3	1	0.429	1965–1988
3	1	MATER EVAL	0.19	0.52	2.2	1	0.483	1964–2004
4	4	MATER PERFORMANCE	-0.06	0.43	0.9	0	0.160	1990–2005
5	5	RUSS J NONDESTRUCT+	-0.32	0.39	0.3	0	0.115	1999–2006

MATERIALS SCIENCE, COATINGS & FILMS

ISI Category Description Materials Science, Coatings & Films covers resources that concentrate on research in coatings and films applied to a base material (substrate). Metals, alloys, resin solutions, and solid/liquid suspensions are the coatings most commonly used in industry. Application methods include electrolysis, vapor deposition, vacuum, or mechanical means such as spraying, calendering, roller coating, extrusion, or thermosetting.

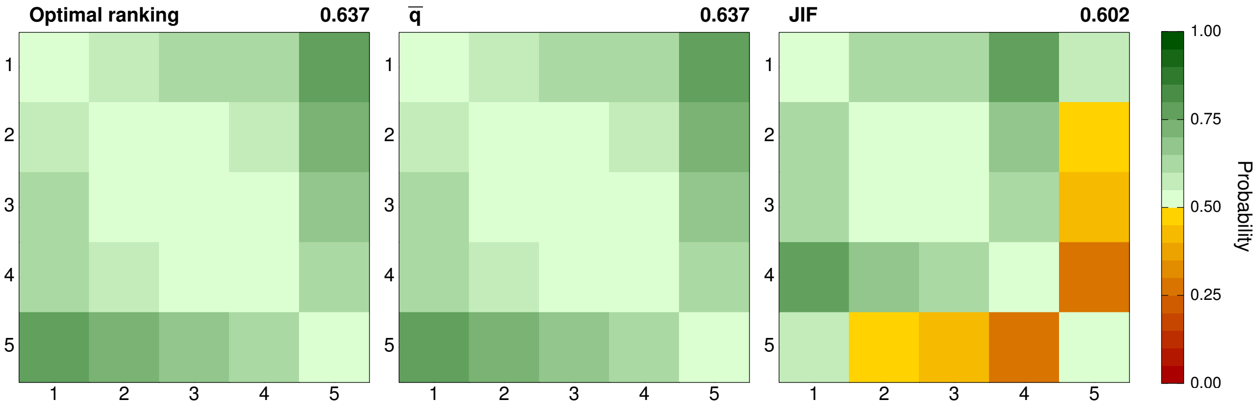

The color of each cell represents the probability of picking a paper with more citations from the higher-ranked journal. Green cells indicate adequate ranking, whereas red cells indicate that the ranking is inadequate (the probability is less than $\frac{1}{2}$). The value of M, the multi-class AUC statistic for each ranking scheme, is above each matrix.

Rank			$\mathbf{p_{ss}(q\|J)}$		n			
**AUC**	**JIF**	**Journal abbreviation**	$\overline{\mathbf{q}}$	σ	$\overline{\mathbf{n}}$	**Q2**	**JIF**	**Steady-state period**
1	1	J ELECTROCHEM SOC	1.13	0.41	20.5	11	2.387	1985–1998
2	5	J VAC SCI TECHNOL A	1.03	0.41	14.4	8	1.394	1992–1996
3	2	THIN SOLID FILMS	0.96	0.41	12.3	7	1.666	1989–1994
4	3	SURF COAT TECH	0.91	0.45	11.2	6	1.559	1986–1995
5	4	APPL SURF SCI	0.62	0.49	5.7	3	1.436	1996–1999

MATERIALS SCIENCE, COMPOSITES

ISI Category Description Materials Science, Composites covers resources that focus on mixtures or mechanical combinations of two or more materials that are solid in the finished state, are mutually insoluble, and differ in chemical nature. The major types of composites are 1) laminates of paper, fabric, or wood and a thermosetting material; 2) reinforced plastics; 3) cermets (ceramic and metal powders); 4) fabrics of natural and synthetic fibers; and 5) filled composites, in which a bonding material is loaded with filler in the form of flakes or small particles.

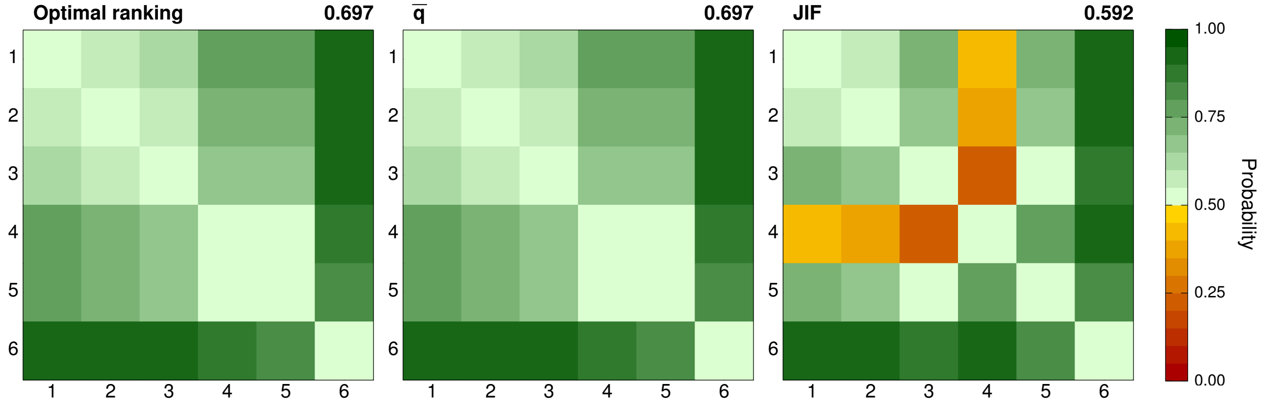

The color of each cell represents the probability of picking a paper with more citations from the higher-ranked journal. Green cells indicate adequate ranking, whereas red cells indicate that the ranking is inadequate (the probability is less than $\frac{1}{2}$). The value of M, the multi-class AUC statistic for each ranking scheme, is above each matrix.

Rank			$p_{ss}(q\|J)$		n			Steady-state
**AUC**	**JIF**	**Journal abbreviation**	$\overline{q}$	σ	$\overline{n}$	**Q2**	**JIF**	**period**
1	4	J COMPOS MATER	1.21	0.46	26.0	13	0.693	1967–1992
2	1	COMPOS SCI TECHNOL	1.12	0.41	17.2	11	2.027	1984–1993
3	2	POLYM COMPOSITE	0.99	0.41	13.5	8	1.163	1981–1997
4	3	COMPOS STRUCT	0.73	0.37	6.0	4	1.002	1994–2000
5	5	J REINF PLAST COMP	0.67	0.42	5.6	3	0.427	1987–1997
6	6	MECH COMPOS MATER	-0.13	0.42	0.5	0	0.275	2002–2006

MATERIALS SCIENCE, MULTIDISCIPLINARY

ISI Category Description Materials Science, Multidisciplinary covers resources having a general or multidisciplinary approach to the study of the nature, behavior, and use of materials. Relevant topics include ceramics, composites, alloys, metals and metallurgy, nanotechnology, nuclear materials, and adhesion and adhesives.

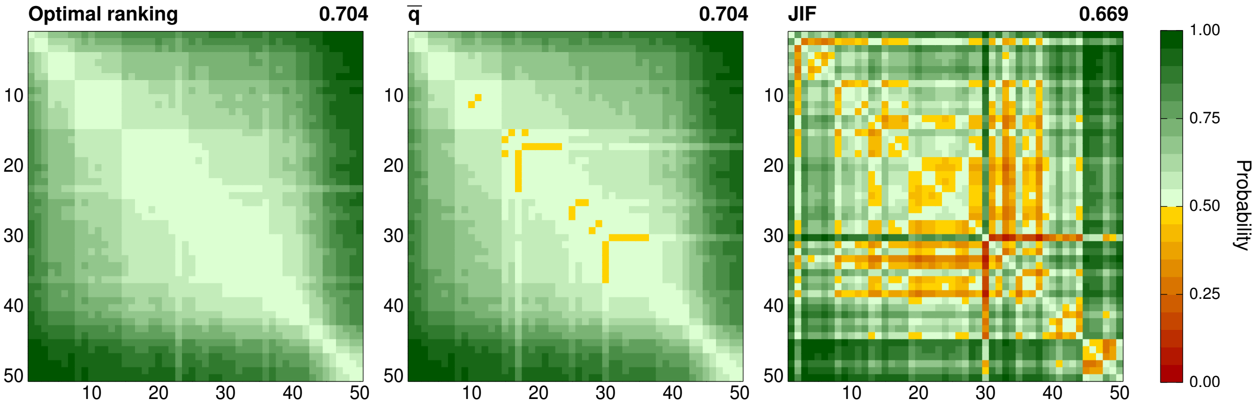

The color of each cell represents the probability of picking a paper with more citations from the higher-ranked journal. Green cells indicate adequate ranking, whereas red cells indicate that the ranking is inadequate (the probability is less than $\frac{1}{2}$). The value of M, the multi-class AUC statistic for each ranking scheme, is above each matrix.

Rank			$p_{ss}(q\|J)$		n			Steady-state
**AUC**	**JIF**	**Journal abbreviation**	$\overline{q}$	σ	$\overline{n}$	**Q2**	**JIF**	**period**
1	1	ADV MATER	1.47	0.43	39.9	25	7.896	1995–2001
2	3	CHEM MATER	1.33	0.38	29.0	18	5.104	1989–2000
3	6	J MECH PHYS SOLIDS	1.28	0.44	42.4	16	3.609	1954–1997
4	7	J MATER RES	1.14	0.41	22.8	11	2.354	1987–1995
5	5	CARBON	1.15	0.41	19.3	11	3.884	1963–1998
6	33	J MATER SCI	1.13	0.42	21.4	11	0.999	1968–1981
7	4	J MATER CHEM	1.11	0.36	15.9	10	4.287	1990–2001
8	38	CORROSION	1.03	0.41	13.8	9	0.687	1963–1994
9	9	CORROS SCI	1.01	0.34	11.5	8	1.885	1996–2000
10	10	APPL PHYS A-MATER	1.00	0.45	15.7	8	1.739	1980–1997
11	15	PHYS CHEM MINER	1.00	0.38	11.3	8	1.517	1996–2000
12	34	J ADHES SCI TECHNOL	0.97	0.40	11.2	7	0.868	1987–1997
13	31	J ADHESION	0.96	0.41	13.8	7	1.046	1968–1992
14	12	THIN SOLID FILMS	0.96	0.41	12.3	7	1.666	1989–1994
15	18	MATER RES BULL	0.87	0.41	9.7	6	1.383	1989–1998
16	11	SYNTHETIC MET	0.86	0.43	10.7	6	1.685	1994–1997
17	29	WEAR	0.86	0.43	10.3	5	1.180	1965–1996
18	36	ACI MATER J	0.87	0.44	8.8	6	0.764	1986–1998
19	17	MAT SCI ENG A-STRUCT	0.85	0.39	9.1	5	1.490	1998–2000
20	28	CEMENT CONCRETE RES	0.84	0.34	7.3	5	1.185	1996–2000
21	16	J ELECTRON MATER	0.84	0.41	9.4	5	1.504	1988–1999
22	8	SOL ENERG MAT SOL C	0.81	0.42	8.4	5	2.321	1991–2000
23	2	MRS BULL	0.86	0.67	14.1	5	5.671	1989–2002
24	24	J ALLOY COMPD	0.80	0.42	8.2	5	1.250	1990–1997

table continues on next page . . .

. . . table continued from previous page

Rank			$p_{ss}(q\|J)$		n			Steady-state
**AUC**	**JIF**	**Journal abbreviation**	$\overline{q}$	σ	$\overline{n}$	**Q2**	**JIF**	**period**
25	37	FATIGUE FRACT ENG M	0.78	0.38	7.3	4	0.756	1985–1999
26	25	J ENG MATER-T ASME	0.77	0.45	10.5	4	1.239	1976–1999
27	23	J NUCL MATER	0.79	0.50	8.1	4	1.261	1990–1994
28	14	SEMICOND SCI TECH	0.75	0.39	7.1	4	1.586	1991–1997
29	32	INT J FATIGUE	0.75	0.39	6.4	4	1.020	1982–2001
30	21	MATER LETT	0.71	0.40	6.7	4	1.353	1984–2000
31	22	MAT SCI ENG B-SOLID	0.71	0.40	6.5	4	1.331	1989–2000
32	44	FERROELECTRICS	0.70	0.50	9.0	3	0.389	1972–1993
33	39	ACI STRUCT J	0.69	0.43	6.7	4	0.657	1986–1999
34	13	MATER CHEM PHYS	0.69	0.41	6.3	3	1.657	1982–2002
35	26	PHYS STATUS SOLIDI A	0.69	0.41	6.4	3	1.221	1984–2000
36	19	J NON-CRYST SOLIDS	0.73	0.62	8.1	3	1.362	1995–1998
37	20	PHILOS MAG	0.66	0.39	6.0	3	1.354	1978–2003
38	27	J MAGN MAGN MATER	0.64	0.48	6.9	3	1.212	1997–2000
39	35	VACUUM	0.58	0.43	5.2	2	0.834	1965–2001
40	42	MATER STRUCT	0.57	0.47	5.7	2	0.459	1990–1996
41	40	J MATER PROCESS TECH	0.54	0.40	3.8	2	0.615	1989–2001
42	43	J MATER ENG PERFORM	0.46	0.40	3.9	2	0.417	1991–1992
43	48	ANN CHIM-SCI MAT	0.35	0.43	2.7	1	0.288	1977–2002
44	41	B MATER SCI	0.32	0.44	2.3	1	0.522	1985–2003
45	49	MATERIALWISS WERKST	0.16	0.44	1.5	1	0.264	1987–2003
46	30	KOVOVE MATER	0.06	0.40	1.0	0	1.138	1976–2001
47	47	ADV MATER PROCESS	-0.16	0.52	0.8	0	0.312	1991–2005
48	46	COMBUST EXPLO SHOCK+	-0.16	0.46	0.7	0	0.358	1972–2005
49	45	INORG MATER+	-0.31	0.46	0.4	0	0.374	1975–2005
50	50	IND DIAMOND REV	-0.43	0.53	0.4	0	0.034	1969–2000

MATERIALS SCIENCE, PAPER & WOOD

ISI Category Description Materials Science, Paper & Wood includes resources that cover all aspects of wood and/or paper production. Topics include cellulose chemistry and technology, pulp and paper science, paper fabrication techniques, and wood and fiber science and technology.

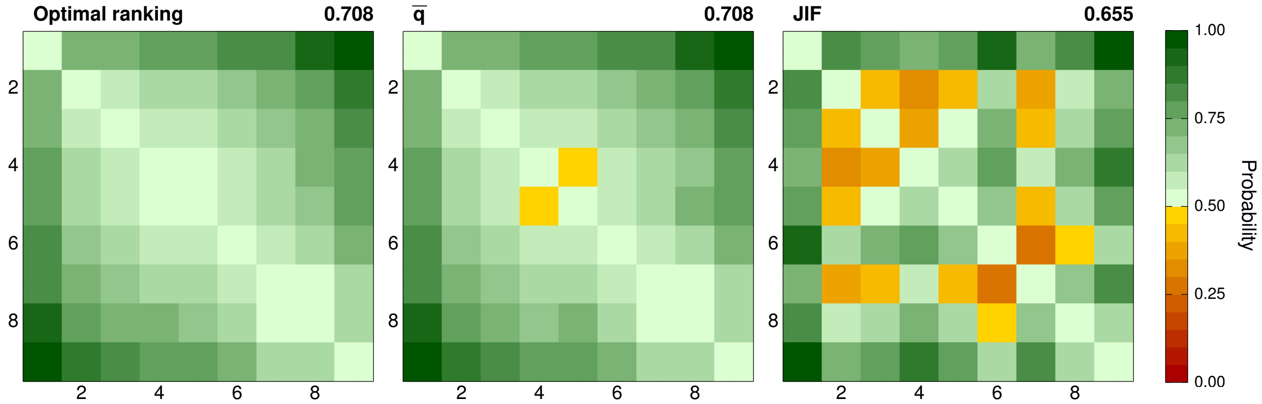

The color of each cell represents the probability of picking a paper with more citations from the higher-ranked journal. Green cells indicate adequate ranking, whereas red cells indicate that the ranking is inadequate (the probability is less than $\frac{1}{2}$). The value of M, the multi-class AUC statistic for each ranking scheme, is above each matrix.

Rank			$p_{ss}(q\|J)$		n			Steady-state
**AUC**	**JIF**	**Journal abbreviation**	$\bar{q}$	σ	$\bar{n}$	**Q2**	**JIF**	**period**
1	1	HOLZFORSCHUNG	0.94	0.41	11.5	7	1.014	1965–1999
2	4	FOREST PROD J	0.56	0.39	4.3	2	0.387	1975–2000
3	7	MOKUZAI GAKKAISHI	0.49	0.47	3.6	2	0.168	1984–1998
4	3	HOLZ ROH WERKST	0.37	0.44	2.7	1	0.514	1974–2001
5	5	APPITA J	0.38	0.52	3.2	1	0.301	1984–2000
6	2	TAPPI J	0.21	0.53	1.8	0	0.675	2001–2003
7	8	PAP PUU-PAP TIM	-0.01	0.78	2.0	0	0.065	1972–1997
8	6	PULP PAP-CANADA	-0.15	0.50	0.8	0	0.176	1971–2003
9	9	WOCHENBL PAPIERFABR	-0.53	0.46	0.2	0	0.012	1997–2006

MATERIALS SCIENCE, TEXTILES

ISI Category Description Materials Science, Textiles covers resources that focus on the manufacture of clothing and furniture from materials made of natural fibers (e.g., leather, cotton, wool, wood) and/or synthetic fibers (e.g., polyester, vinyl, nylon). Resources covering dyes and colors and fiber chemistry are also included.

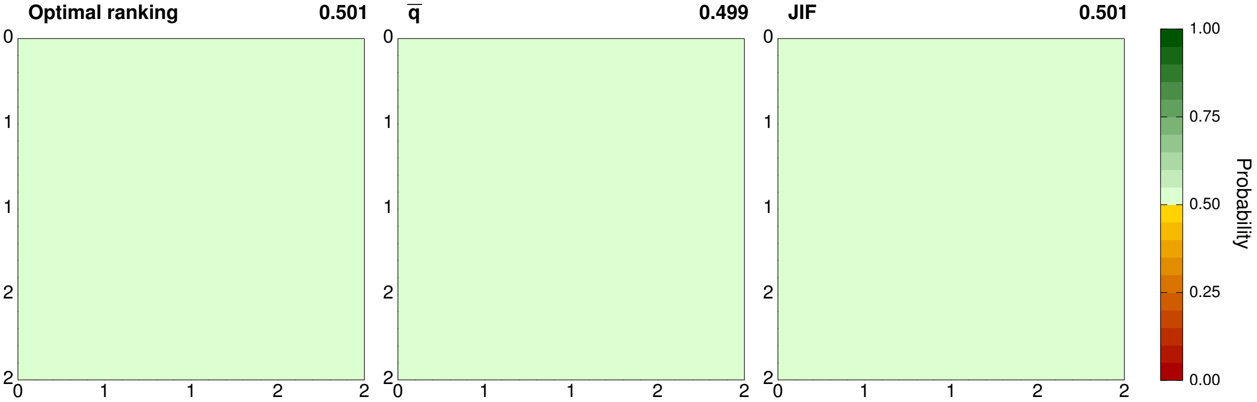

The color of each cell represents the probability of picking a paper with more citations from the higher-ranked journal. Green cells indicate adequate ranking, whereas red cells indicate that the ranking is inadequate (the probability is less than $\frac{1}{2}$). The value of M, the multi-class AUC statistic for each ranking scheme, is above each matrix.

**Rank**			$\mathbf{p_{ss}(q\|J)}$		$\mathbf{n}$			**Steady-state**
**AUC**	**JIF**	**Journal abbreviation**	$\overline{\mathbf{q}}$	σ	$\overline{\mathbf{n}}$	**Q2**	**JIF**	**period**
1	1	DYES PIGMENTS	0.69	0.36	5.9	3	1.909	1980–2003
2	2	TEXT RES J	0.69	0.38	5.8	4	0.578	1971–1999

MATHEMATICAL & COMPUTATIONAL BIOLOGY

ISI Category Description Mathematical and Computational Biology includes resources concerning the use of mathematical, statistical and computational methods to address data analysis, modeling, and information management in biological problems, processes and systems. Among the areas covered are biostatistics, bioinformatics, biometrics, modeling of biological systems, and computational biology.

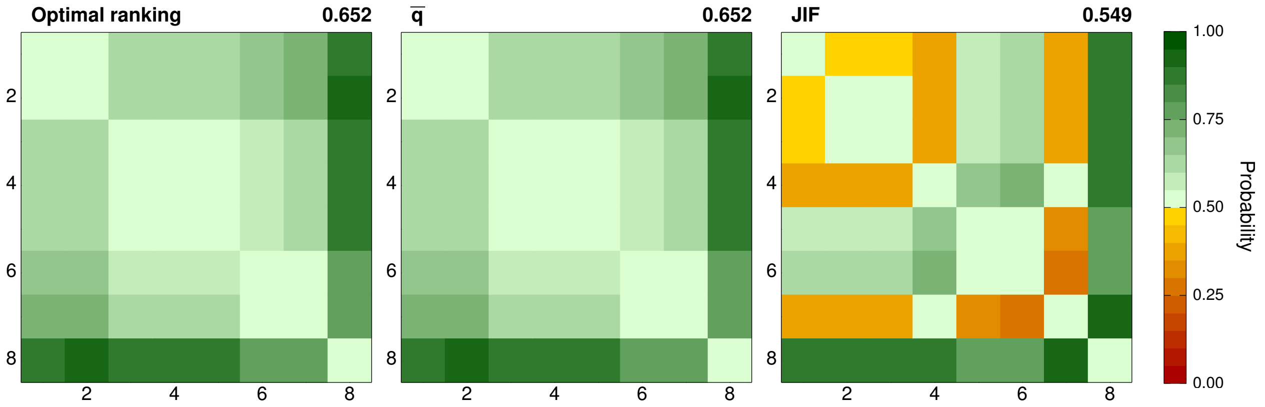

The color of each cell represents the probability of picking a paper with more citations from the higher-ranked journal. Green cells indicate adequate ranking, whereas red cells indicate that the ranking is inadequate (the probability is less than $\frac{1}{2}$). The value of M, the multi-class AUC statistic for each ranking scheme, is above each matrix.

**Rank**			$\mathbf{p_{ss}(q\|J)}$		**n**			**Steady-state**
**AUC**	**JIF**	**Journal abbreviation**	$\overline{\mathbf{q}}$	σ	$\overline{\mathbf{n}}$	**Q2**	**JIF**	**period**
1	4	BIOMETRICS	1.25	0.46	48.1	14	1.489	1954–1994
2	7	BIOMETRIKA	1.22	0.42	36.8	13	1.014	1955–1996
3	2	STAT MED	1.04	0.40	16.9	9	1.737	1994–1997
4	3	J MATH BIOL	1.04	0.41	16.5	9	1.664	1975–1998
5	1	J THEOR BIOL	1.03	0.41	16.3	9	2.264	1980–1998
6	5	MATH BIOSCI	0.89	0.44	12.4	6	1.126	1975–2001
7	6	MED BIOL ENG COMPUT	0.82	0.42	9.2	5	1.018	1976–1999
8	8	BIOMETRICAL J	0.27	0.44	2.3	1	0.612	1989–2005

MATHEMATICS, APPLIED

ISI Category Description Mathematics, Applied covers resources concerned with areas of mathematics that may be applied to other fields of science. It includes areas such as differential equations, numerical analysis, nonlinearity, control, software, systems analysis, computational mathematics and mathematical modeling. Resources that are concerned with mathematical methods and whose primary focus is on a specific non-mathematics discipline such as biology, psychology, history, economics etc., are covered in the MATHEMATICS, INTERDISCIPLINARY APPLICATIONS category.

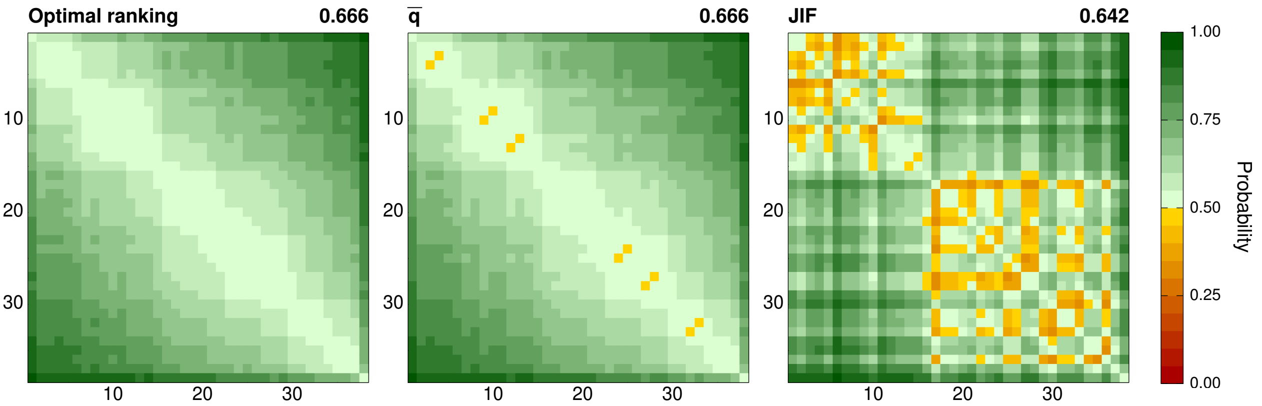

The color of each cell represents the probability of picking a paper with more citations from the higher-ranked journal. Green cells indicate adequate ranking, whereas red cells indicate that the ranking is inadequate (the probability is less than $\frac{1}{2}$). The value of M, the multi-class AUC statistic for each ranking scheme, is above each matrix.

Rank			$p_{ss}(q\|J)$		n			Steady-state
AUC	JIF	Journal abbreviation	$\overline{q}$	σ	$\overline{n}$	Q2	JIF	period
1	6	NONLINEARITY	1.23	0.37	25.2	16	1.288	1989–1991
2	8	FUZZY SET SYST	1.11	0.45	23.4	11	1.181	1980–1987
3	4	SIAM J NUMER ANAL	1.10	0.43	21.3	10	1.335	1967–1999
4	11	MATH PROGRAM	1.11	0.43	18.6	10	1.117	1976–1998
5	7	SIAM J CONTROL OPTIM	1.09	0.43	17.5	10	1.263	1976–1996
6	3	SIAM J COMPUT	1.02	0.40	16.1	8	1.361	1989–1997
7	12	NUMER MATH	0.98	0.44	16.7	8	1.116	1966–1997
8	1	PHYSICA D	0.98	0.39	12.6	8	1.674	1996–2000
9	9	MATH COMPUT	0.93	0.44	18.9	7	1.155	1963–1992
10	13	INFORM COMPUT	0.94	0.46	15.7	6	1.107	1983–1996
11	5	INVERSE PROBL	0.91	0.40	11.0	6	1.319	1985–1999
12	2	SIAM J APPL MATH	0.90	0.46	13.4	6	1.425	1965–2000
13	15	ALGORITHMICA	0.90	0.46	12.1	6	0.850	1988–1997
14	14	COMMUN PART DIFF EQ	0.88	0.43	11.2	5	1.094	1981–1997
15	10	SIAM J MATH ANAL	0.82	0.43	9.9	5	1.134	1973–2002
16	27	ANN PURE APPL LOGIC	0.75	0.43	7.3	4	0.582	1983–1996
17	21	NONLINEAR ANAL-THEOR	0.73	0.45	7.9	4	0.677	1980–1994
18	28	DISCRETE APPL MATH	0.72	0.45	7.8	4	0.577	1978–1996
19	16	APPL NUMER MATH	0.71	0.43	6.9	4	0.835	1984–1999
20	24	J OPTIMIZ THEORY APP	0.70	0.45	7.7	3	0.633	1974–1998
21	20	P ROY SOC EDINB A	0.66	0.44	6.7	3	0.684	1976–1998

table continues on next page . . .

... table continued from previous page

**Rank**			$\mathbf{p_{ss}(q\|J)}$		**n**			**Steady-state**
**AUC**	**JIF**	**Journal abbreviation**	$\overline{\mathbf{q}}$	σ	$\overline{\mathbf{n}}$	**Q2**	**JIF**	**period**
22	33	J PURE APPL ALGEBRA	0.63	0.41	5.5	3	0.470	1985–1997
23	18	J COMPUT APPL MATH	0.61	0.42	5.4	3	0.759	1982–2000
24	19	J MATH ANAL APPL	0.61	0.46	6.3	3	0.758	1983–1992
25	23	J SYMB COMPUT	0.61	0.41	5.0	3	0.641	1994–2002
26	36	TOPOL APPL	0.57	0.39	4.5	3	0.388	1980–1995
27	31	P AM MATH SOC	0.54	0.43	4.5	2	0.513	1967–1994
28	22	Z ANGEW MATH PHYS	0.54	0.48	4.8	2	0.652	1970–2002
29	32	MATH METHOD APPL SCI	0.53	0.42	4.2	2	0.473	1991–2001
30	26	LINEAR ALGEBRA APPL	0.44	0.42	3.4	2	0.585	1994–2001
31	25	COMPUT MATH APPL	0.42	0.45	3.7	1	0.611	1979–2002
32	29	APPL MATH LETT	0.37	0.44	3.0	1	0.546	1990–2002
33	17	APPL MATH COMPUT	0.37	0.47	3.4	1	0.816	1984–2001
34	34	MATH COMPUT MODEL	0.31	0.47	2.9	1	0.432	1987–2002
35	30	MATH COMPUT SIMULAT	0.26	0.44	2.3	1	0.534	1981–2003
36	35	INT J COMPUT MATH	0.26	0.48	2.6	1	0.428	1964–2001
37	37	SCI CHINA SER A	0.17	0.47	1.9	1	0.311	1984–2002
38	38	PMM-J APPL MATH MEC+	-0.34	0.52	0.6	0	0.191	1965–2003

MATHEMATICS, INTERDISCIPLINARY APPLICATIONS

ISI Category Description Mathematics, Interdisciplinary Applications includes resources concerned with mathematical methods whose primary focus is on a specific non-mathematics discipline such as biology, psychology, history, economics, etc. Resources that focus on specific mathematical topics such as differential equations, numerical analysis, nonlinearity, etc., are covered in the MATHEMATICS, APPLIED category.

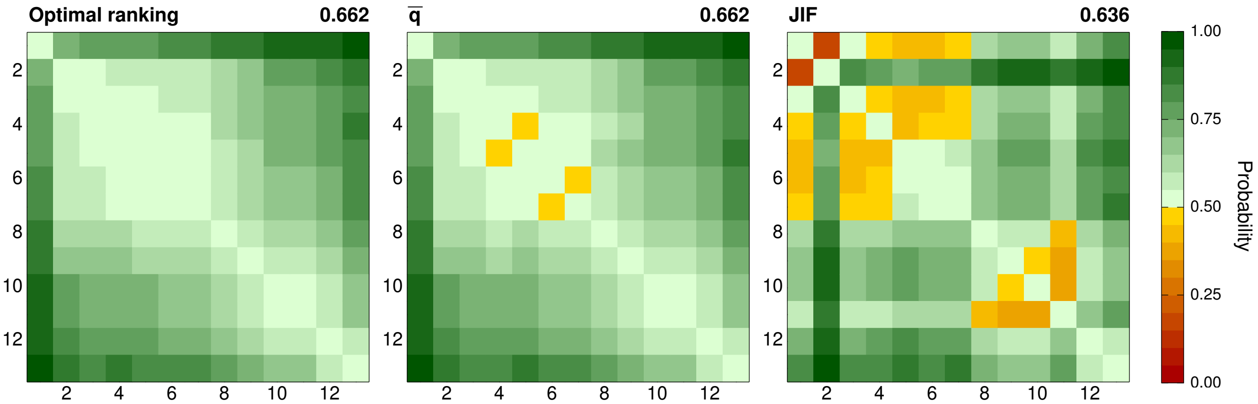

The color of each cell represents the probability of picking a paper with more citations from the higher-ranked journal. Green cells indicate adequate ranking, whereas red cells indicate that the ranking is inadequate (the probability is less than $\frac{1}{2}$). The value of M, the multi-class AUC statistic for each ranking scheme, is above each matrix.

Rank			$\mathbf{p_{ss}(q\|J)}$		n			Steady-state
**AUC**	**JIF**	**Journal abbreviation**	$\bar{q}$	σ	$\bar{n}$	**Q2**	**JIF**	**period**
1	2	ECONOMETRICA	1.58	0.45	88.6	35	2.402	1980–1994
2	5	J ECONOMETRICS	1.13	0.45	28.1	11	1.669	1979–1996
3	6	ARCH RATION MECH AN	1.08	0.47	21.4	9	1.650	1956–1999
4	7	INT J NUMER METH ENG	1.03	0.39	16.3	9	1.497	1990–1996
5	4	RISK ANAL	1.04	0.45	16.2	9	1.938	1983–1999
6	1	CHEMOMETR INTELL LAB	0.96	0.42	14.8	8	2.450	1986–2002
7	3	COMPUT METHOD APPL M	0.97	0.47	17.8	7	2.015	1976–1998
8	11	INT J NUMER METH FL	0.85	0.43	11.5	5	0.870	1980–1998
9	8	INT J QUANTUM CHEM	0.77	0.52	8.9	4	1.182	1992–1998
10	10	ENG ANAL BOUND ELEM	0.62	0.40	5.1	3	0.883	1984–2002
11	9	EDUC PSYCHOL MEAS	0.61	0.44	6.6	3	0.921	1964–2000
12	12	APPL MATH MODEL	0.47	0.43	4.0	2	0.583	1977–2002
13	13	J FRANKLIN I	0.31	0.45	2.3	1	0.362	1985–2004

MATHEMATICS

ISI Category Description Mathematics covers resources having a broad, general approach to the field. The category also includes resources focusing on specific fields of basic research in Mathematics such as topology, algebra, functional analysis, combinatorial theory, differential geometry and number theory.

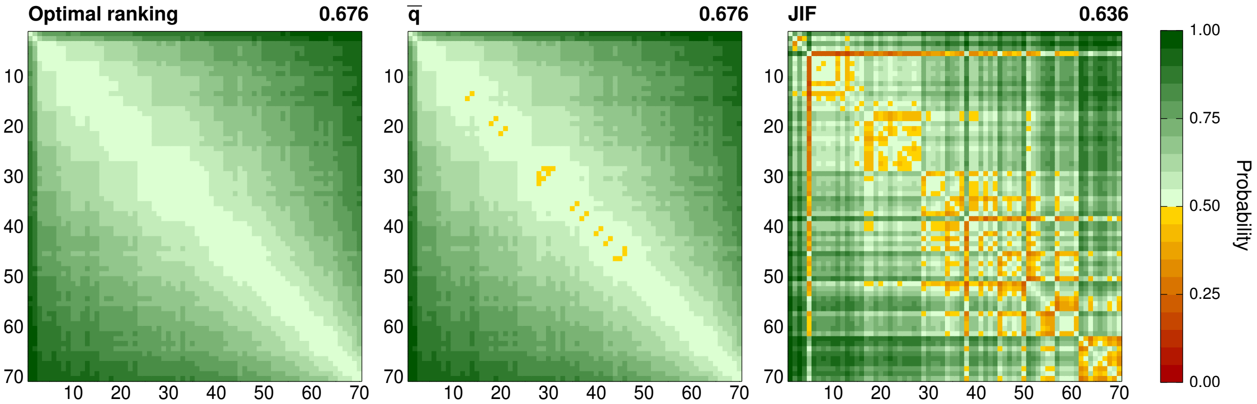

The color of each cell represents the probability of picking a paper with more citations from the higher-ranked journal. Green cells indicate adequate ranking, whereas red cells indicate that the ranking is inadequate (the probability is less than $\frac{1}{2}$). The value of M, the multi-class AUC statistic for each ranking scheme, is above each matrix.

Rank			$p_{ss}(q\|J)$		n			Steady-state
**AUC**	**JIF**	**Journal abbreviation**	$\overline{q}$	σ	$\overline{n}$	**Q2**	**JIF**	**period**
1	1	ANN MATH	1.45	0.44	45.2	25	2.426	1954–1995
2	3	INVENT MATH	1.22	0.35	21.4	14	1.659	1987–1993
3	13	J FUNCT ANAL	1.00	0.43	15.4	8	0.866	1975–1995
4	11	P LOND MATH SOC	0.96	0.42	12.9	7	0.902	1964–1996
5	4	DUKE MATH J	0.96	0.40	12.8	7	1.409	1987–1996
6	2	B AM MATH SOC	0.94	0.40	17.9	6	2.385	1978–2002
7	6	J DIFFER EQUATIONS	0.90	0.40	9.9	6	1.166	1992–1998
8	8	COMMUN PART DIFF EQ	0.88	0.43	11.2	5	1.094	1981–1997
9	14	J REINE ANGEW MATH	0.87	0.40	10.0	5	0.860	1980–1996
10	7	ADV MATH	0.85	0.45	11.1	5	1.143	1992–1998
11	9	INDIANA U MATH J	0.85	0.46	12.3	5	1.029	1969–1996
12	16	J COMB THEORY B	0.84	0.42	10.1	5	0.792	1974–1995
13	10	AM J MATH	0.83	0.38	8.7	5	0.933	1988–1999
14	12	MATH ANN	0.84	0.44	10.3	5	0.902	1954–1993
15	22	J COMB THEORY A	0.81	0.41	9.4	5	0.677	1974–1994
16	19	ANN I FOURIER	0.79	0.45	9.7	4	0.698	1966–1995
17	27	MATH Z	0.76	0.43	8.3	4	0.570	1961–1995
18	15	T AM MATH SOC	0.75	0.41	8.0	4	0.820	1989–1998
19	23	COMPOS MATH	0.75	0.44	8.4	4	0.675	1967–1997
20	28	J ALGEBRA	0.74	0.42	7.7	4	0.568	1981–1991
21	26	ANN PURE APPL LOGIC	0.75	0.43	7.3	4	0.582	1983–1996
22	21	NONLINEAR ANAL-THEOR	0.73	0.45	7.9	4	0.677	1980–1994
23	25	J LOND MATH SOC	0.73	0.41	6.8	4	0.617	1977–1996
24	51	DISCRETE MATH	0.67	0.44	7.1	3	0.347	1974–1989

table continues on next page . . .

. . . table continued from previous page

Rank			$p_{ss}(q\|J)$		n			Steady-state
AUC	JIF	Journal abbreviation	$\overline{q}$	σ	$\overline{n}$	Q2	JIF	period
25	20	P ROY SOC EDINB A	0.66	0.44	6.7	3	0.684	1976–1998
26	24	J SYMBOLIC LOGIC	0.66	0.44	6.7	3	0.664	1965–1993
27	37	J PURE APPL ALGEBRA	0.63	0.41	5.5	3	0.470	1985–1997
28	30	MATH PROC CAMBRIDGE	0.63	0.40	5.2	3	0.536	1987–1999
29	39	CAN J MATH	0.63	0.44	6.0	3	0.464	1971–1997
30	40	PAC J MATH	0.62	0.43	5.9	3	0.411	1969–1996
31	18	EUR J COMBIN	0.63	0.44	5.0	3	0.710	1985–1996
32	17	J MATH ANAL APPL	0.61	0.46	6.3	3	0.758	1983–1992
33	42	J GRAPH THEOR	0.59	0.38	4.5	3	0.403	1992–2000
34	33	J APPROX THEORY	0.58	0.43	5.0	2	0.500	1974–1998
35	44	TOPOL APPL	0.57	0.39	4.5	3	0.388	1980–1995
36	31	STUD MATH	0.58	0.44	4.9	2	0.515	1978–1999
37	32	P AM MATH SOC	0.54	0.43	4.5	2	0.513	1967–1994
38	52	ACTA ARITH	0.54	0.42	4.1	2	0.345	1980–1997
39	57	COMMUN ALGEBRA	0.50	0.41	4.0	2	0.268	1982–1994
40	47	MANUSCRIPTA MATH	0.48	0.38	3.4	2	0.376	1991–2001
41	36	J NUMBER THEORY	0.49	0.40	3.3	2	0.479	1993–2000
42	29	B LOND MATH SOC	0.44	0.36	3.2	2	0.556	1994–2002
43	35	INTEGR EQUAT OPER TH	0.44	0.39	3.1	2	0.481	1996–2003
44	43	FUND MATH	0.43	0.41	3.0	2	0.398	1980–2001
45	61	AM MATH MON	0.44	0.50	5.5	1	0.237	1960–1995
46	49	SEMIGROUP FORUM	0.43	0.44	3.2	2	0.361	1978–1998
47	53	ARCH MATH	0.40	0.44	3.2	1	0.341	1965–1996
48	41	GEOMETRIAE DEDICATA	0.38	0.40	3.0	1	0.408	1990–2002
49	46	MATH NACHR	0.38	0.44	3.1	1	0.382	1964–2001
50	48	ILLINOIS J MATH	0.35	0.40	2.5	1	0.374	1996–2002
51	34	OSAKA J MATH	0.33	0.43	2.3	1	0.485	1994–2001
52	58	B AUST MATH SOC	0.30	0.46	2.6	1	0.266	1980–2001
53	59	P JPN ACAD A-MATH	0.28	0.40	2.0	1	0.256	1983–2000
54	60	CAN MATH BULL	0.27	0.41	1.8	1	0.246	1994–2002
55	5	LECT NOTES MATH	0.25	0.54	3.0	1	1.206	1980–2002
56	70	FIBONACCI QUART	0.23	0.44	1.8	1	0.096	1974–1999
57	64	CZECH MATH J	0.20	0.41	1.7	1	0.197	1988–2001
58	54	SCI CHINA SER A	0.17	0.47	1.9	1	0.311	1984–2002
59	45	ACTA MATH HUNG	0.16	0.43	1.7	1	0.384	1988–2002
60	68	ARS COMBINATORIA	0.12	0.42	1.5	0	0.142	1997–2001
61	50	HOUSTON J MATH	0.09	0.47	1.2	0	0.354	1999–2004
62	38	CHINESE ANN MATH B	0.06	0.47	1.4	0	0.470	1980–2005
63	56	PUBL MATH-DEBRECEN	0.05	0.43	1.1	0	0.279	1985–2002
64	55	RUSS MATH SURV+	0.04	0.49	1.6	0	0.303	1993–2001
65	69	INDIAN J PURE AP MAT	-0.03	0.44	0.9	0	0.109	1979–2002
66	65	ROCKY MT J MATH	-0.09	0.49	0.8	0	0.183	1998–2006
67	67	ACTA MATH SCI	-0.17	0.45	0.6	0	0.169	1989–2005

table continues on next page . . .

...table continued from previous page

Rank			$\mathbf{p_{ss}(q\|J)}$		n			Steady-state
**AUC**	**JIF**	**Journal abbreviation**	$\overline{\mathbf{q}}$	σ	$\overline{\mathbf{n}}$	**Q2**	**JIF**	**period**
68	63	SIBERIAN MATH J+	-0.22	0.49	0.6	0	0.206	1974–2001
69	66	MATH NOTES+	-0.24	0.45	0.5	0	0.172	1974–2002
70	62	DIFF EQUAT+	-0.49	0.55	0.4	0	0.215	1979–1999

MECHANICS

ISI Category Description Mechanics includes resources that cover the study of the behavior of physical systems under the action of forces. Relevant topics in this category include fluid mechanics, solid mechanics, gas mechanics, mathematical modeling (chaos and fractals, finite element analysis), thermal engineering, fracture mechanics, heat and mass flow and transfer, phase equilibria studies, plasticity, adhesion, rheology, gravity effects, vibration effects, and wave motion analysis.

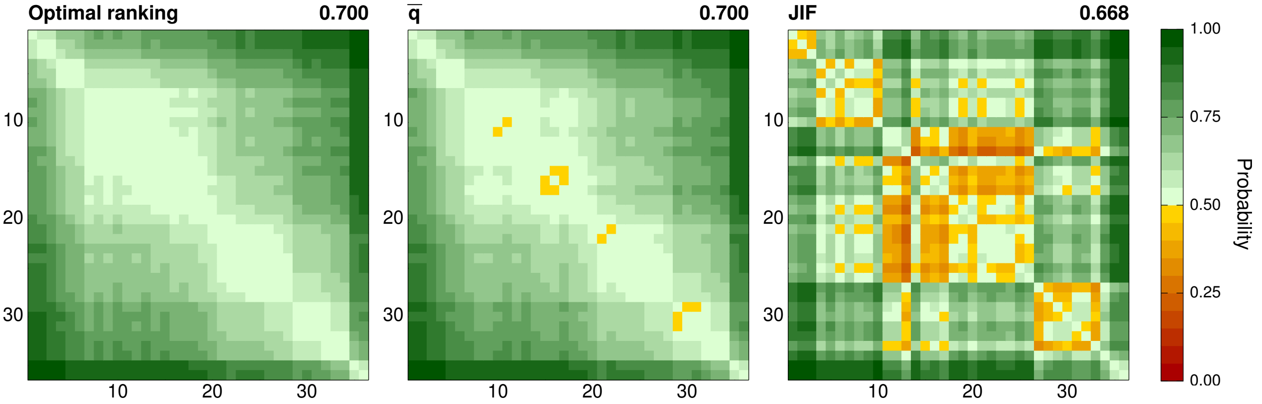

The color of each cell represents the probability of picking a paper with more citations from the higher-ranked journal. Green cells indicate adequate ranking, whereas red cells indicate that the ranking is inadequate (the probability is less than $\frac{1}{2}$). The value of M, the multi-class AUC statistic for each ranking scheme, is above each matrix.

Rank			$p_{ss}(q\|J)$		n			Steady-state
**AUC**	**JIF**	**Journal abbreviation**	$\bar{q}$	σ	$\bar{n}$	**Q2**	**JIF**	**period**
1	3	J FLUID MECH	1.42	0.45	43.0	23	2.022	1956–1991
2	2	J RHEOL	1.34	0.35	25.9	19	2.082	1993–1996
3	1	J MECH PHYS SOLIDS	1.28	0.44	42.4	16	3.609	1954–1997
4	10	J NON-NEWTON FLUID	1.15	0.39	18.7	12	1.449	1975–1998
5	5	PHYS FLUIDS	1.10	0.44	20.4	10	1.697	1966–1996
6	7	ARCH RATION MECH AN	1.08	0.47	21.4	9	1.650	1956–1999
7	4	COMPUT METHOD APPL M	0.97	0.47	17.8	7	2.015	1976–1998
8	25	J ADHES SCI TECHNOL	0.97	0.40	11.2	7	0.868	1987–1997
9	21	J APPL MECH-T ASME	0.96	0.47	17.1	7	0.943	1976–1993
10	14	INT J MULTIPHAS FLOW	0.95	0.35	11.0	7	1.274	1993–1999
11	19	J ADHESION	0.96	0.41	13.8	7	1.046	1968–1992
12	8	INT J SOLIDS STRUCT	0.95	0.43	14.8	7	1.529	1975–1997
13	9	INT J HEAT MASS TRAN	0.93	0.34	10.4	7	1.482	1991–1997
14	6	RHEOL ACTA	0.91	0.43	12.3	6	1.653	1974–2001
15	22	NUMER HEAT TR A-APPL	0.88	0.34	8.8	6	0.936	1988–1992
16	18	EXP FLUIDS	0.88	0.42	10.8	6	1.112	1983–1994
17	26	INT J THERMOPHYS	0.88	0.39	9.2	6	0.793	1985–1997
18	23	J SOUND VIB	0.87	0.43	10.7	6	0.884	1966–1994
19	24	INT J NUMER METH FL	0.85	0.43	11.5	5	0.870	1980–1998
20	20	INT J MECH SCI	0.80	0.45	9.4	4	0.950	1968–1997
21	16	INT J IMPACT ENG	0.70	0.43	6.9	3	1.195	1987–2000
22	33	J VIB ACOUST	0.73	0.49	7.0	4	0.565	1984–1997

table continues on next page . . .

...table continued from previous page

Rank			$\mathbf{p_{ss}(q\|J)}$		n			Steady-state
**AUC**	**JIF**	**Journal abbreviation**	$\overline{\mathbf{q}}$	σ	$\overline{\mathbf{n}}$	**Q2**	**JIF**	**period**
23	11	ENG FRACT MECH	0.68	0.40	6.0	3	1.390	1987–2003
24	15	INT J NONLINEAR MECH	0.68	0.42	6.4	3	1.205	1976–2002
25	12	PHILOS MAG	0.66	0.39	6.0	3	1.354	1978–2003
26	30	INT J FRACTURE	0.63	0.46	5.7	3	0.685	1993–2001
27	17	EXP MECH	0.60	0.46	5.9	3	1.133	1966–2002
28	29	ACTA MECH	0.59	0.44	6.1	2	0.690	1968–1998
29	32	APPL MATH MODEL	0.47	0.43	4.0	2	0.583	1977–2002
30	28	INT COMMUN HEAT MASS	0.46	0.38	3.2	2	0.708	1982–2002
31	31	J WIND ENG IND AEROD	0.47	0.43	3.4	2	0.586	1994–1999
32	13	ENERG CONVERS MANAGE	0.40	0.45	3.1	1	1.325	1987–2006
33	27	MECH RES COMMUN	0.34	0.47	3.0	1	0.788	1975–2002
34	34	ARCH MECH	0.30	0.47	3.3	1	0.429	1965–1988
35	35	MECH COMPOS MATER	-0.13	0.42	0.5	0	0.275	2002–2006
36	36	PMM-J APPL MATH MEC+	-0.34	0.52	0.6	0	0.191	1965–2003

MEDICAL INFORMATICS

ISI Category Description Medical Informatics covers resources on health care information in clinical studies and medical research. This category includes resources on the evaluation, assessment, and use of health care technology, its consequences for patients, and its impact on society.

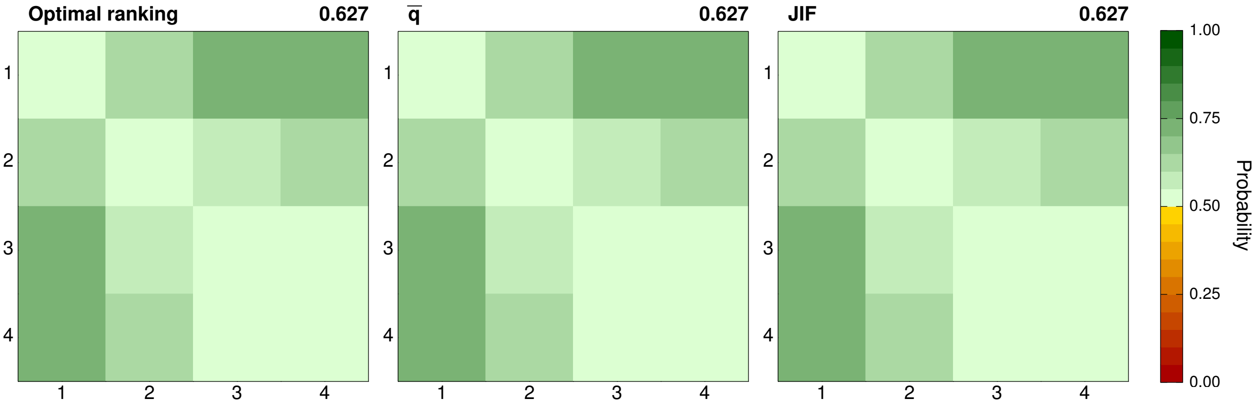

The color of each cell represents the probability of picking a paper with more citations from the higher-ranked journal. Green cells indicate adequate ranking, whereas red cells indicate that the ranking is inadequate (the probability is less than $\frac{1}{2}$). The value of M, the multi-class AUC statistic for each ranking scheme, is above each matrix.

**Rank**			$\mathbf{p_{ss}(q\|J)}$		$\mathbf{n}$			**Steady-state**
**AUC**	**JIF**	**Journal abbreviation**	$\overline{\mathbf{q}}$	σ	$\overline{\mathbf{n}}$	**Q2**	**JIF**	**period**
1	1	STAT MED	1.04	0.40	16.9	9	1.737	1994–1997
2	2	MED BIOL ENG COMPUT	0.82	0.42	9.2	5	1.018	1976–1999
3	3	IEEE ENG MED BIOL	0.67	0.53	6.7	3	0.940	1995–1999
4	4	COMPUT METH PROG BIO	0.61	0.40	6.4	3	0.624	1984–2002

MEDICAL LABORATORY TECHNOLOGY

ISI Category Description Medical Laboratory Technology covers resources on the testing, methods, and equipment used in clinical, medical, hospital, and pathology laboratories, including clinical chemistry and biochemical analysis of laboratory samples. Resources on the development and refinement of the diagnostic technologies used in these laboratories are also covered.

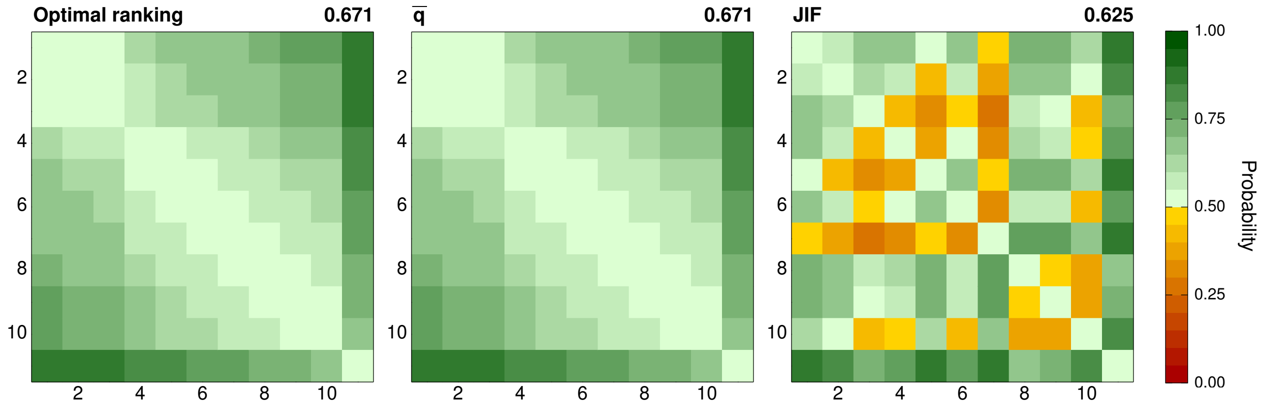

The color of each cell represents the probability of picking a paper with more citations from the higher-ranked journal. Green cells indicate adequate ranking, whereas red cells indicate that the ranking is inadequate (the probability is less than $\frac{1}{2}$). The value of M, the multi-class AUC statistic for each ranking scheme, is above each matrix.

Rank			$p_{ss}(q\|J)$		n			Steady-state
**AUC**	**JIF**	**Journal abbreviation**	$\overline{q}$	σ	$\overline{n}$	**Q2**	**JIF**	**period**
1	7	ARCH PATHOL LAB MED	1.23	0.38	23.0	14	1.605	1975–1989
2	1	CLIN CHEM	1.21	0.40	23.8	13	5.454	1985–2001
3	5	J LAB CLIN MED	1.17	0.38	17.9	13	1.812	1994–1997
4	2	THER DRUG MONIT	1.06	0.36	14.0	9	3.032	1980–2000
5	10	DIAGN CYTOPATHOL	1.02	0.37	11.5	8	0.786	1986–1995
6	4	CLIN CHIM ACTA	0.96	0.37	12.7	7	2.328	1990–1996
7	6	ANN CLIN BIOCHEM	0.91	0.39	11.1	7	1.741	1972–1997
8	3	CLIN BIOCHEM	0.88	0.38	10.3	6	2.331	1967–2002
9	9	J CLIN LAB ANAL	0.81	0.35	8.1	5	1.117	1986–1999
10	8	ANN CLIN LAB SCI	0.78	0.42	8.2	4	1.227	1973–2000
11	11	ANN BIOL CLIN-PARIS	0.42	0.46	3.6	1	0.342	1964–1997

MEDICINE, GENERAL & INTERNAL

ISI Category Description Medicine, General & Internal covers resources on medical specialties such as general medicine, family medicine, internal medicine, clinical physiology, pain management, and military and hospital medicine.

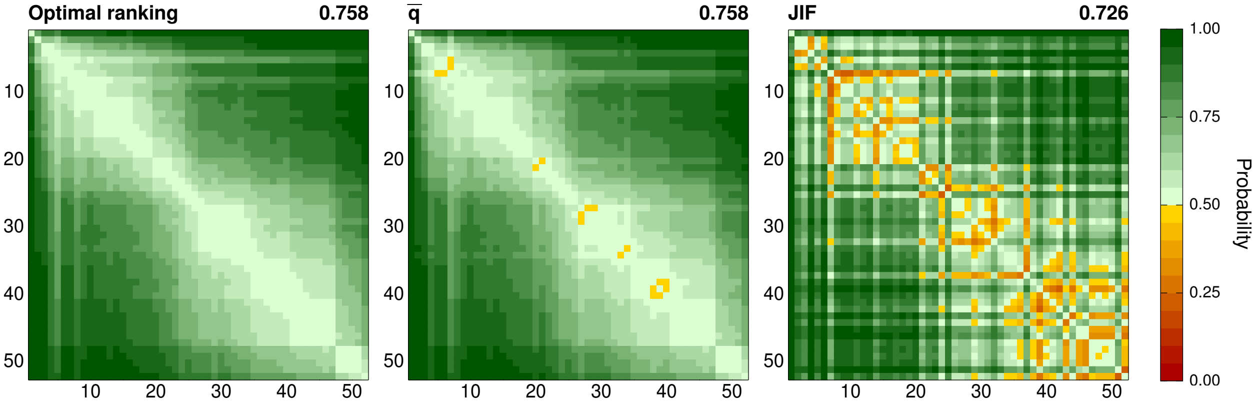

The color of each cell represents the probability of picking a paper with more citations from the higher-ranked journal. Green cells indicate adequate ranking, whereas red cells indicate that the ranking is inadequate (the probability is less than $\frac{1}{2}$). The value of M, the multi-class AUC statistic for each ranking scheme, is above each matrix.

Rank			$p_{ss}(q\|J)$		n			Steady-state
**AUC**	**JIF**	**Journal abbreviation**	$\overline{q}$	σ	$\overline{n}$	**Q2**	**JIF**	**period**
1	1	NEW ENGL J MED	2.23	0.46	268.7	175	51.296	1989–1993
2	4	ANN INTERN MED	1.84	0.50	92.9	62	14.780	1970–1997
3	6	ARCH INTERN MED	1.56	0.39	49.0	32	7.920	1989–1999
4	2	LANCET	1.53	0.64	60.3	31	25.800	2000–2002
5	3	JAMA-J AM MED ASSOC	1.34	0.75	76.3	26	23.175	1995–1997
6	9	AM J MED	1.37	0.44	35.8	20	4.518	1982–2000
7	10	MAYO CLIN PROC	1.34	0.44	32.7	19	4.022	1967–1995
8	5	BRIT MED J	1.28	0.59	38.0	16	9.245	1985–1998
9	16	PREV MED	1.28	0.45	26.2	16	2.390	1979–1998
10	13	EUR J CLIN INVEST	1.25	0.37	24.7	15	2.847	1983–1994
11	12	J GEN INTERN MED	1.22	0.40	22.3	14	2.964	1985–2000
12	8	J INTERN MED	1.19	0.39	22.0	13	4.730	1988–1994
13	15	J PAIN SYMPTOM MANAG	1.18	0.42	19.5	13	2.437	1990–2000
14	20	J LAB CLIN MED	1.17	0.38	17.9	13	1.812	1994–1997
15	18	BRIT J GEN PRACT	1.15	0.38	16.6	12	1.938	1989–1996
16	19	BRIT MED BULL	1.12	0.42	22.4	11	1.881	1986–1999
17	11	AM J PREV MED	1.09	0.41	16.4	10	3.497	1986–2002
18	17	MED CLIN N AM	1.06	0.41	15.8	9	2.054	1969–2000
19	25	J FAM PRACTICE	1.03	0.40	13.9	9	1.278	1999–2001
20	23	AM J MED SCI	0.93	0.37	10.5	7	1.355	1980–1997
21	22	FAM PRACT	0.95	0.44	10.5	6	1.558	1986–2000
22	14	MED J AUSTRALIA	0.90	0.45	11.6	6	2.582	1985–1998
23	32	AVIAT SPACE ENVIR MD	0.83	0.43	9.1	5	0.830	1974–1996
24	7	CAN MED ASSOC J	0.80	0.53	10.3	4	6.862	1962–2004

table continues on next page . . .

... table continued from previous page

**Rank**			$\mathbf{p_{ss}(q\|J)}$		$\mathbf{n}$			**Steady-state**
**AUC**	**JIF**	**Journal abbreviation**	$\overline{\mathbf{q}}$	σ	$\overline{\mathbf{n}}$	**Q2**	**JIF**	**period**
25	37	DEUT MED WOCHENSCHR	0.69	0.41	6.6	4	0.584	1959–1985
26	21	AM FAM PHYSICIAN	0.68	0.45	5.1	3	1.616	1989–2002
27	28	NETH J MED	0.57	0.39	4.5	2	1.162	1973–2001
28	31	TOHOKU J EXP MED	0.55	0.38	4.3	2	1.012	1975–2000
29	26	J NATL MED ASSOC	0.57	0.47	4.9	2	1.255	1991–2001
30	27	INDIAN J MED RES	0.55	0.37	3.8	2	1.224	1988–2000
31	33	WIEN KLIN WOCHENSCHR	0.51	0.40	3.7	2	0.804	1995–2002
32	42	SCOT MED J	0.49	0.45	4.2	2	0.367	1976–1998
33	30	MT SINAI J MED	0.48	0.47	4.2	2	1.063	1969–2003
34	24	MED CLIN-BARCELONA	0.49	0.46	3.5	2	1.327	1996–2000
35	44	PRESSE MED	0.41	0.50	3.5	1	0.338	1963–1996
36	52	BRIT J HOSP MED	0.38	0.47	3.1	1	0.022	1973–1993
37	34	MIL MED	0.35	0.47	3.2	1	0.747	1960–1994
38	41	IRISH J MED SCI	0.32	0.43	2.5	1	0.400	1970–2001
39	29	POSTGRAD MED J	0.33	0.50	2.7	1	1.093	1996–2002
40	35	CAN FAM PHYSICIAN	0.34	0.55	2.9	1	0.701	1995–1998
41	47	MED KLIN	0.26	0.43	2.1	1	0.289	1973–2002
42	49	MEDICINA-BUENOS AIRE	0.23	0.43	2.1	1	0.272	1971–2002
43	40	REV MED CHILE	0.21	0.40	1.7	1	0.405	1971–2002
44	48	INTERNIST	0.21	0.43	1.7	1	0.277	1983–2000
45	36	CHINESE MED J-PEKING	0.18	0.43	1.7	1	0.615	1963–2003
46	38	REV MED INTERNE	0.18	0.50	1.7	0	0.478	1984–2003
47	50	REV INVEST CLIN	0.17	0.43	1.6	1	0.243	1971–2002
48	45	B ACAD NAT MED PARIS	-0.09	0.47	0.9	0	0.323	1976–2003
49	43	ANN SAUDI MED	-0.09	0.38	0.6	0	0.360	1998–2005
50	39	REV CLIN ESP	-0.16	0.39	0.5	0	0.430	2000–2005
51	46	SAUDI MED J	-0.21	0.42	0.4	0	0.300	2003–2006
52	51	TERAPEVT ARKH	-0.43	0.38	0.2	0	0.099	1973–2005

MEDICINE, LEGAL

ISI Category Description Medicine, Legal covers resources on all aspects of medical legal issues, including government regulations and policies, malpractice, toxicological and pharmacological regulations, clinical therapeutic patents and other critical legal issues at the interface of law, medicine, and healthcare. The category also covers resources dealing with the various branches of forensic science.

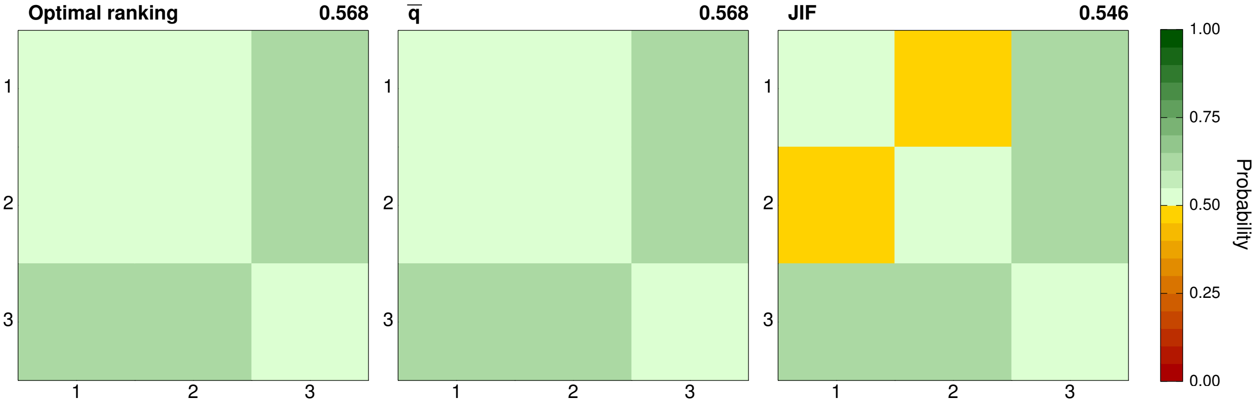

The color of each cell represents the probability of picking a paper with more citations from the higher-ranked journal. Green cells indicate adequate ranking, whereas red cells indicate that the ranking is inadequate (the probability is less than $\frac{1}{2}$). The value of M, the multi-class AUC statistic for each ranking scheme, is above each matrix.

Rank			$p_{ss}(q\|J)$		n			Steady-state
**AUC**	**JIF**	**Journal abbreviation**	$\overline{q}$	σ	$\overline{n}$	**Q2**	**JIF**	**period**
1	2	J FORENSIC SCI	0.85	0.39	9.0	5	0.846	1977–1998
2	1	FORENSIC SCI INT	0.83	0.40	7.9	5	1.397	1980–2000
3	3	AM J FOREN MED PATH	0.65	0.44	5.1	3	0.745	1979–1999

MEDICINE, RESEARCH & EXPERIMENTAL

ISI Category Description Medicine, Research & Experimental includes resources describing general medical research with a particular emphasis on extremely novel techniques and clinical interventions in a broad range of medical specializations and applications, including vaccine development, tissue replacement, immunotherapies, and other experimental therapeutic strategies. Resources in this category reflect clinical interventions that are in early stages of development, using in vitro or animal models, and small-scale clinical trials.

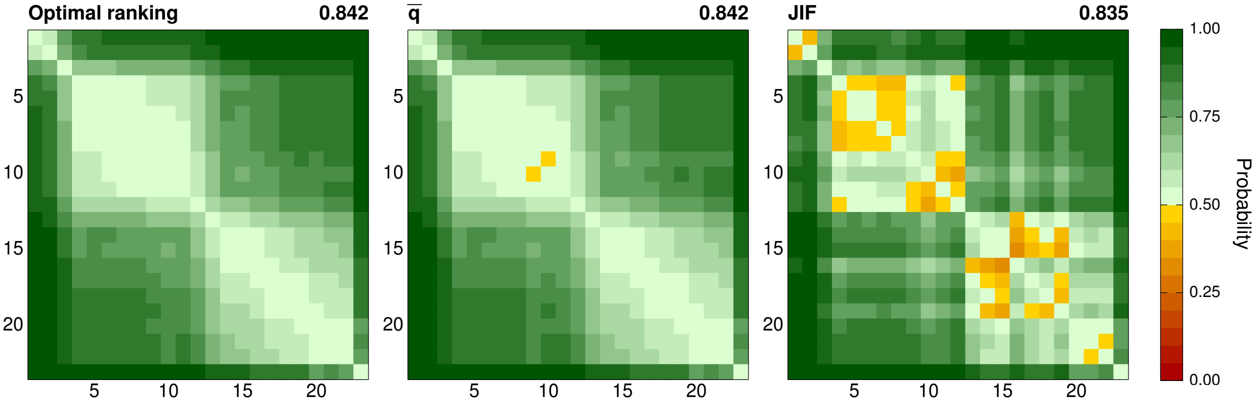

The color of each cell represents the probability of picking a paper with more citations from the higher-ranked journal. Green cells indicate adequate ranking, whereas red cells indicate that the ranking is inadequate (the probability is less than $\frac{1}{2}$). The value of M, the multi-class AUC statistic for each ranking scheme, is above each matrix.

Rank			$p_{ss}(q\|J)$		n			Steady-state
AUC	JIF	Journal abbreviation	$\overline{q}$	σ	$\overline{n}$	Q2	JIF	period
1	2	J EXP MED	1.97	0.36	132.9	86	14.484	1987–1995
2	1	J CLIN INVEST	1.85	0.35	94.3	65	15.754	1978–1997
3	3	LAB INVEST	1.55	0.40	49.5	31	4.453	1963–1993
4	8	LIFE SCI	1.29	0.43	31.6	16	2.389	1963–1986
5	7	EUR J CLIN INVEST	1.25	0.37	24.7	15	2.847	1983–1994
6	5	CLIN SCI	1.24	0.36	23.9	14	3.263	1985–1994
7	12	ARCH PATHOL LAB MED	1.23	0.38	23.0	14	1.605	1975–1989
8	6	VACCINE	1.23	0.36	20.9	14	3.159	1987–1998
9	4	EXP HEMATOL	1.18	0.38	20.3	12	3.408	1985–1998
10	11	LARYNGOSCOPE	1.19	0.42	19.4	13	1.736	1979–1994
11	9	J LAB CLIN MED	1.17	0.38	17.9	13	1.812	1994–1997
12	10	STAT MED	1.04	0.40	16.9	9	1.737	1994–1997
13	16	SCAND J CLIN LAB INV	0.88	0.45	10.7	6	1.216	1983–1998
14	13	BIOMED PHARMACOTHER	0.72	0.40	6.8	4	1.526	1981–2003
15	19	J INT MED RES	0.72	0.41	6.9	4	0.665	1973–2001
16	17	BRAZ J MED BIOL RES	0.66	0.38	5.4	3	1.075	1981–2000
17	14	MED HYPOTHESES	0.62	0.41	5.6	3	1.299	1976–2000
18	18	TOHOKU J EXP MED	0.55	0.38	4.3	2	1.012	1975–2000
19	15	INDIAN J MED RES	0.55	0.37	3.8	2	1.224	1988–2000
20	20	BIOMED RES-TOKYO	0.47	0.50	4.1	2	0.493	1988–2001
21	22	CURR THER RES CLIN E	0.45	0.44	3.8	2	0.319	1984–2002
22	21	ANN BIOL CLIN-PARIS	0.42	0.46	3.6	1	0.342	1964–1997
23	23	B EXP BIOL MED+	-0.51	0.48	0.2	0	0.190	1960–2005

METALLURGY & METALLURGICAL ENGINEERING

ISI Category Description Metallurgy & Metallurgical Engineering includes resources that cover the numerous chemical and physical processes used to isolate a metallic element from its naturally occurring state, refine it, and convert it into a useful alloy or product. Topics in this category include corrosion prevention and control, hydrometallurgy, pyrometallurgy, electrometallurgy, phase equilibria, iron-making, steel-making, oxidation, plating and finishing, powder metallurgy, and welding.

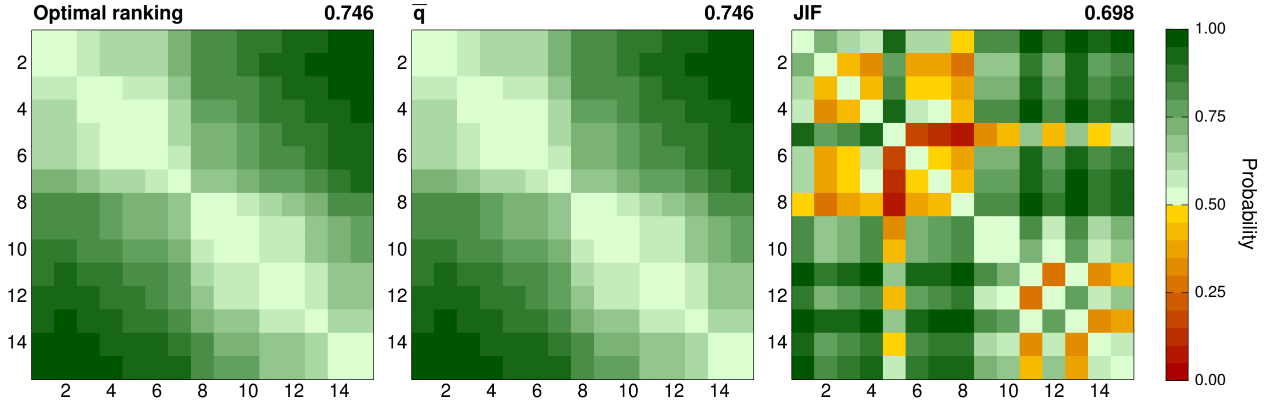

The color of each cell represents the probability of picking a paper with more citations from the higher-ranked journal. Green cells indicate adequate ranking, whereas red cells indicate that the ranking is inadequate (the probability is less than $\frac{1}{2}$). The value of M, the multi-class AUC statistic for each ranking scheme, is above each matrix.

Rank			$p_{ss}(q\|J)$		n			Steady-state
AUC	JIF	Journal abbreviation	$\bar{q}$	σ	$\bar{n}$	Q2	JIF	period
1	8	CORROSION	1.03	0.41	13.8	9	0.687	1963–1994
2	1	CORROS SCI	1.01	0.34	11.5	8	1.885	1996–2000
3	4	HYDROMETALLURGY	0.91	0.33	9.2	6	1.227	1976–2001
4	7	ISIJ INT	0.87	0.39	8.6	6	0.741	1990–1997
5	6	Z METALLKD	0.84	0.42	9.5	5	0.866	1966–1997
6	3	J ALLOY COMPD	0.80	0.42	8.2	5	1.250	1990–1997
7	2	PHILOS MAG	0.66	0.39	6.0	3	1.354	1978–2003
8	9	J JPN I MET	0.39	0.41	2.9	1	0.434	1975–2001
9	10	WELD J	0.30	0.56	3.3	1	0.350	1964–2000
10	12	TETSU TO HAGANE	0.25	0.46	2.1	1	0.237	1984–2002
11	14	STAHL EISEN	0.08	0.49	1.3	0	0.112	1981–1997
12	5	KOVOVE MATER	0.06	0.40	1.0	0	1.138	1976–2001
13	15	T INDIAN I METALS	-0.13	0.43	0.6	0	0.078	1997–2004
14	11	PROT MET+	-0.57	0.52	0.2	0	0.309	1985–2005
15	13	MET SCI HEAT TREAT+	-0.71	0.48	0.1	0	0.131	1973–2004

METEOROLOGY & ATMOSPHERIC SCIENCES

ISI Category Description Meteorology & Atmospheric Sciences covers those resources that deal with the atmosphere and its phenomena, especially weather and weather forecasting. Resources in this category are concerned with the atmosphere's temperature, density, winds, clouds, precipitation and other characteristics, as well as the structure and evolution of the atmosphere in terms of external influences and the basic laws of physics. This category also includes resources dealing with climatology.

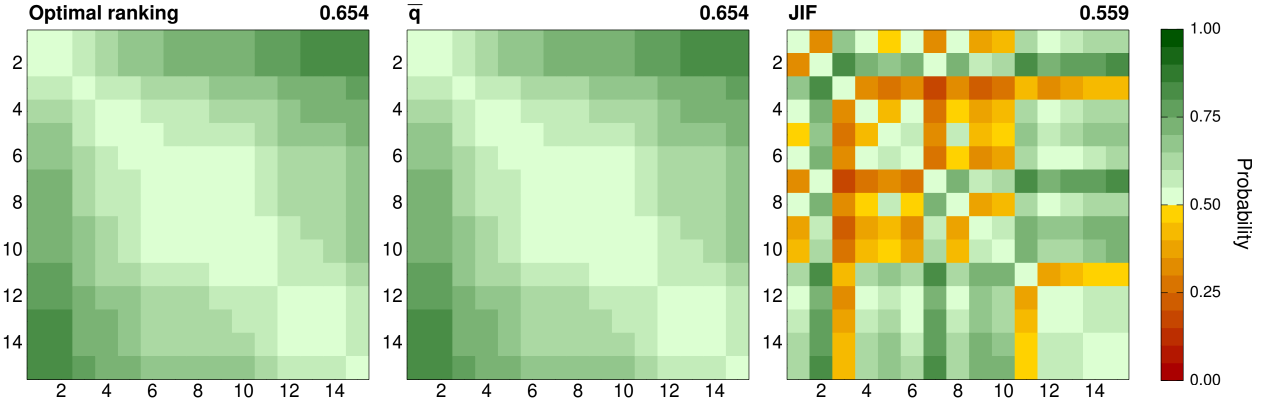

The color of each cell represents the probability of picking a paper with more citations from the higher-ranked journal. Green cells indicate adequate ranking, whereas red cells indicate that the ranking is inadequate (the probability is less than $\frac{1}{2}$). The value of M, the multi-class AUC statistic for each ranking scheme, is above each matrix.

Rank			$p_{ss}(q\|J)$		n			Steady-state
AUC	JIF	Journal abbreviation	$\overline{q}$	σ	$\overline{n}$	Q2	JIF	period
1	2	J CLIMATE	1.47	0.40	43.0	26	3.419	1988–1998
2	7	J ATMOS SCI	1.47	0.42	45.6	26	2.163	1969–1989
3	9	Q J ROY METEOR SOC	1.35	0.46	37.1	20	2.045	1954–1995
4	10	MON WEATHER REV	1.25	0.39	24.5	15	1.927	1991–1997
5	5	ATMOS ENVIRON	1.21	0.40	21.1	14	2.630	1989–1997
6	1	B AM METEOROL SOC	1.11	0.53	42.0	11	3.728	1989–1997
7	8	BOUND-LAY METEOROL	1.10	0.41	19.4	10	2.054	1978–1997
8	4	AGR FOREST METEOROL	1.09	0.39	17.3	10	2.903	1983–2002
9	6	INT J CLIMATOL	1.07	0.38	16.0	10	2.332	1988–2001
10	12	J ATMOS OCEAN TECH	1.05	0.42	15.8	9	1.298	1988–1996
11	13	WATER AIR SOIL POLL	1.00	0.41	13.9	8	1.205	1974–1995
12	14	RADIO SCI	0.91	0.45	12.2	6	1.084	1965–1997
13	15	J METEOROL SOC JPN	0.87	0.42	11.8	6	0.797	1980–1999
14	11	J AIR WASTE MANAGE	0.87	0.44	11.0	5	1.441	1989–2000
15	3	J AEROSOL SCI	0.77	0.50	9.6	4	2.952	1979–1999

MICROBIOLOGY

ISI Category Description Microbiology includes resources dealing with all aspects of fundamental and applied studies of microorganisms, including bacteria, viruses, and fungi. This category also considers resources on the clinical aspects of the occurrence and treatment of microbial pathogens, basic science studies of microbial biochemistry and function, environmental microbiology, and bacterial/viral uses in biotechnology.

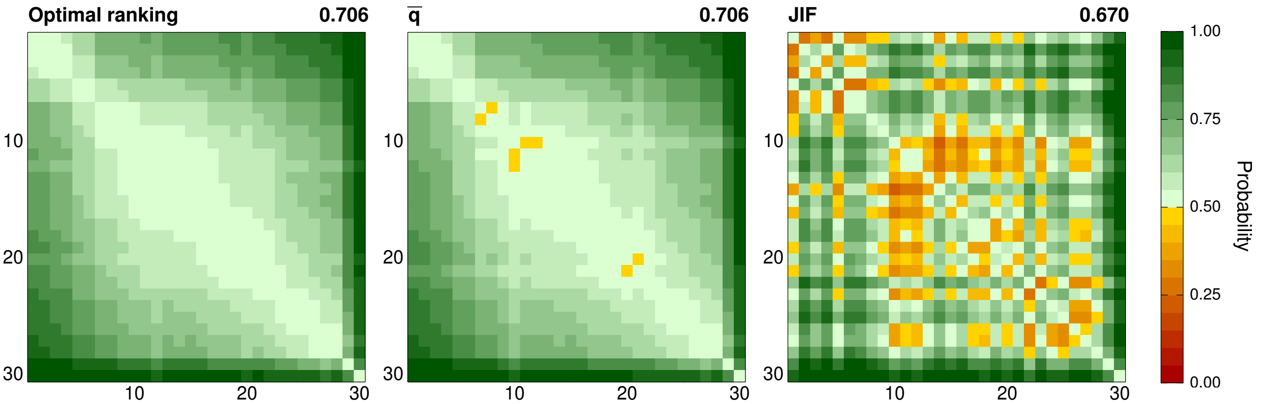

The color of each cell represents the probability of picking a paper with more citations from the higher-ranked journal. Green cells indicate adequate ranking, whereas red cells indicate that the ranking is inadequate (the probability is less than $\frac{1}{2}$). The value of M, the multi-class AUC statistic for each ranking scheme, is above each matrix.

Rank			$p_{ss}(q\|J)$		n			Steady-state
AUC	JIF	Journal abbreviation	$\overline{q}$	σ	$\overline{n}$	Q2	JIF	period
1	2	MOL MICROBIOL	1.57	0.32	43.8	33	5.634	1988–1999
2	4	J BACTERIOL	1.54	0.33	42.4	31	3.993	1984–1993
3	6	APPL ENVIRON MICROB	1.51	0.35	42.7	28	3.532	1990–1993
4	7	J CLIN MICROBIOL	1.49	0.36	41.6	27	3.445	1991–1995
5	14	MICROB PATHOGENESIS	1.39	0.38	30.6	22	2.258	1985–1988
6	3	ANTIMICROB AGENTS CH	1.38	0.38	32.7	21	4.153	1971–1998
7	16	ORAL MICROBIOL IMMUN	1.28	0.32	21.6	16	2.089	1987–1993
8	8	FEMS MICROBIOL ECOL	1.28	0.34	21.8	16	3.157	1984–2000
9	19	SYST APPL MICROBIOL	1.24	0.39	24.7	14	2.037	1985–1994
10	21	YEAST	1.20	0.34	25.4	14	1.955	1992–1994
11	9	INT J FOOD MICROBIOL	1.20	0.37	19.5	13	2.608	1983–2000
12	1	CLIN INFECT DIS	1.21	0.46	23.6	13	6.186	1995–1998
13	23	ARCH MICROBIOL	1.18	0.35	18.7	13	1.820	1988–1999
14	13	EUR J CLIN MICROBIOL	1.17	0.37	19.0	12	2.330	1982–1996
15	15	J MED MICROBIOL	1.16	0.35	18.2	12	2.180	1982–1995
16	5	J ANTIMICROB CHEMOTH	1.16	0.37	18.3	12	3.891	1976–2000
17	27	J ANTIBIOT	1.13	0.37	18.6	11	1.262	1963–1992
18	26	CAN J MICROBIOL	1.10	0.39	17.3	11	1.275	1954–1995
19	17	VET MICROBIOL	1.09	0.35	14.3	10	2.073	1975–2000
20	20	ANTON LEEUW INT J G	1.06	0.38	21.1	10	1.964	1994–1998
21	11	RES MICROBIOL	1.07	0.35	14.3	10	2.504	1990–2001
22	18	FEMS MICROBIOL LETT	1.05	0.38	14.7	9	2.068	1976–1998
23	12	J MICROBIOL METH	1.01	0.38	12.2	8	2.442	1998–2001

table continues on next page . . .

. . . table continued from previous page

Rank			$\mathbf{p_{ss}(q\|J)}$		n			Steady-state
**AUC**	**JIF**	**Journal abbreviation**	$\overline{\mathbf{q}}$	σ	$\overline{\mathbf{n}}$	**Q2**	**JIF**	**period**
24	10	DIAGN MICR INFEC DIS	0.94	0.37	11.4	7	2.553	1982–2002
25	24	LETT APPL MICROBIOL	0.93	0.38	11.5	7	1.593	1984–2000
26	28	CURR MICROBIOL	0.92	0.39	10.0	6	1.007	1984–1998
27	22	APMIS	0.86	0.40	8.8	5	1.875	1987–1999
28	25	MICROBIOL IMMUNOL	0.85	0.36	7.8	5	1.502	1996–2000
29	29	FOLIA MICROBIOL	0.53	0.37	3.4	2	0.963	1985–2002
30	30	MICROBIOLOGY+	-0.21	0.48	0.6	0	0.543	1973–2005

MICROSCOPY

ISI Category Description Microscopy covers those resources that focus on the interpretative application of microscope magnification to the study of materials that cannot be seen properly by the unaided eye. The instruments used in microscopy may be either optical in nature, or use radiation other than light for making enlarged images of minute objects (e.g., an electron microscope).

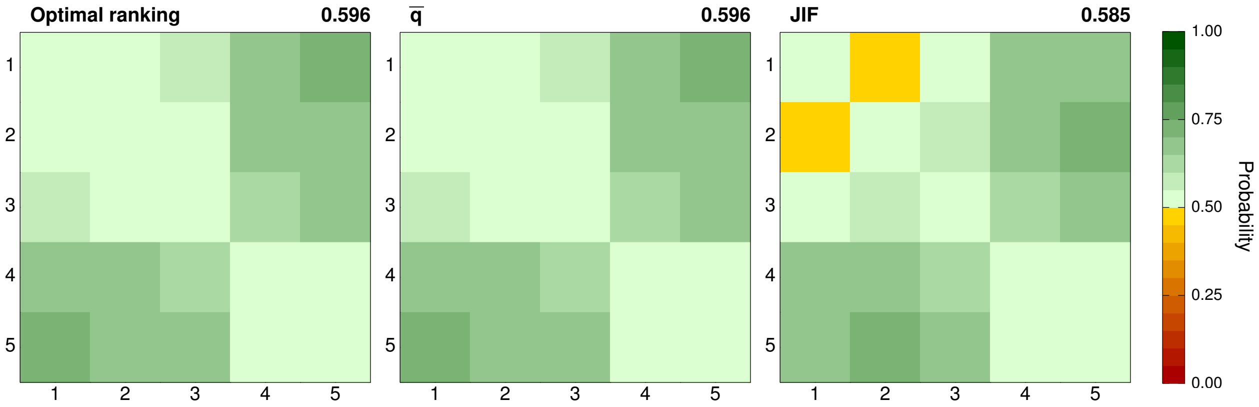

The color of each cell represents the probability of picking a paper with more citations from the higher-ranked journal. Green cells indicate adequate ranking, whereas red cells indicate that the ranking is inadequate (the probability is less than $\frac{1}{2}$). The value of M, the multi-class AUC statistic for each ranking scheme, is above each matrix.

**Rank**			$\mathbf{p_{ss}(q\|J)}$		**n**			**Steady-state**
**AUC**	**JIF**	**Journal abbreviation**	$\overline{\mathbf{q}}$	σ	$\overline{\mathbf{n}}$	**Q2**	**JIF**	**period**
1	2	ULTRAMICROSCOPY	1.07	0.41	18.4	10	1.706	1974–1998
2	1	J MICROSC-OXFORD	1.05	0.42	19.0	9	1.947	1967–2000
3	3	MICROSC RES TECHNIQ	1.00	0.41	12.1	8	1.680	1991–2001
4	4	ULTRASTRUCT PATHOL	0.79	0.34	6.7	5	0.835	1995–2001
5	5	J ELECTRON MICROSC	0.72	0.44	6.7	4	0.777	1955–1999

MINERALOGY

ISI Category Description Mineralogy includes resources that deal with the science of minerals, their crystallography, physical and chemical properties, classification, and the ways of distinguishing them.

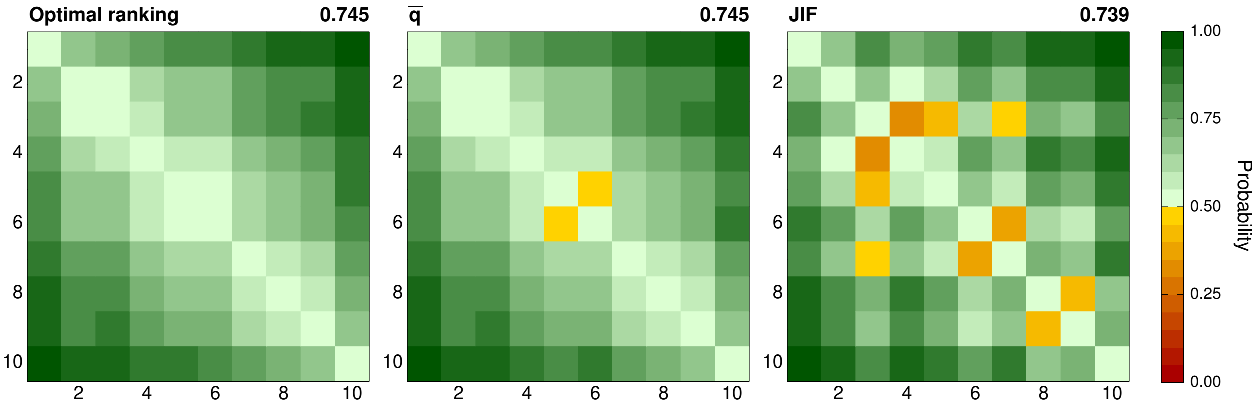

The color of each cell represents the probability of picking a paper with more citations from the higher-ranked journal. Green cells indicate adequate ranking, whereas red cells indicate that the ranking is inadequate (the probability is less than $\frac{1}{2}$). The value of M, the multi-class AUC statistic for each ranking scheme, is above each matrix.

Rank			$p_{ss}(q\|J)$		n			Steady-state
**AUC**	**JIF**	**Journal abbreviation**	$\overline{q}$	σ	$\overline{n}$	**Q2**	**JIF**	**period**
1	1	CONTRIB MINERAL PETR	1.51	0.35	46.7	28	2.754	1974–1993
2	2	AM MINERAL	1.27	0.41	27.8	16	1.977	1954–1997
3	4	CLAY CLAY MINER	1.22	0.32	20.1	15	1.423	1990–1994
4	5	EUR J MINERAL	1.09	0.39	16.9	10	1.219	1988–1992
5	7	CAN MINERAL	1.00	0.36	13.4	8	0.972	1980–1997
6	3	PHYS CHEM MINER	1.00	0.38	11.3	8	1.517	1996–2000
7	6	MINERAL MAG	0.84	0.37	8.1	5	1.064	1993–2001
8	9	INT J MINER PROCESS	0.75	0.37	5.9	4	0.884	1991–2002
9	8	MINER ENG	0.63	0.37	4.6	3	0.942	1987–2000
10	10	NEUES JB MINER MONAT	0.35	0.40	2.3	1	0.487	1990–2003

MINING & MINERAL PROCESSING

ISI Category Description Mining & Mineral Processing includes resources on locating and evaluating mineral deposits; designing and constructing mines; developing mining equipment; supervising mining operations and safety; and extracting, cleaning, sizing, and dressing mined material. Relevant topics in this category include exploration and mining geology, rock mechanics, geophysics, and mining science and technology.

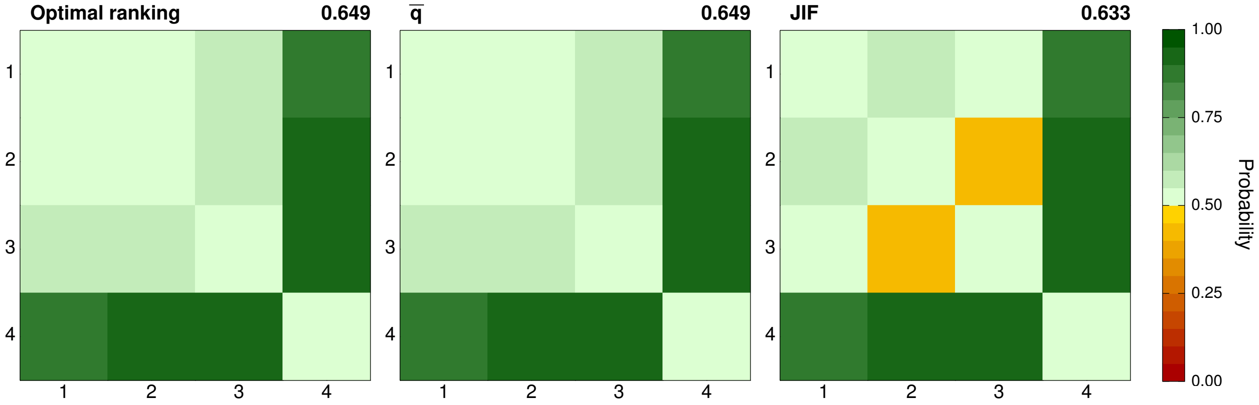

The color of each cell represents the probability of picking a paper with more citations from the higher-ranked journal. Green cells indicate adequate ranking, whereas red cells indicate that the ranking is inadequate (the probability is less than $\frac{1}{2}$). The value of M, the multi-class AUC statistic for each ranking scheme, is above each matrix.

Rank			$\mathbf{p_{ss}(q\|J)}$		n			Steady-state
**AUC**	**JIF**	**Journal abbreviation**	$\overline{\mathbf{q}}$	σ	$\overline{\mathbf{n}}$	**Q2**	**JIF**	**period**
1	1	J NUCL MATER	0.79	0.50	8.1	4	1.261	1990–1994
2	3	INT J MINER PROCESS	0.75	0.37	5.9	4	0.884	1991–2002
3	2	MINER ENG	0.63	0.37	4.6	3	0.942	1987–2000
4	4	ENG MIN J	-1.32	0.71	0.0	0	0.008	1998–2006

MULTIDISCIPLINARY SCIENCES

ISI Category Description Multidisciplinary Sciences includes resources of a very broad or general character in the sciences. It covers the spectrum of major scientific disciplines such as Physics, Chemistry, Mathematics, Biology, etc. Nature and Science are the preeminent resources in this category and serve as typical examples. The Web site of the National Science Foundation is a good example of a web resource included in this category. Some specialized resources that have a wide range of applications in the sciences also may fall under this category. The journal Fractals—Complex Geometry Patterns and Scaling in Nature and Society would be an example of such a resource.

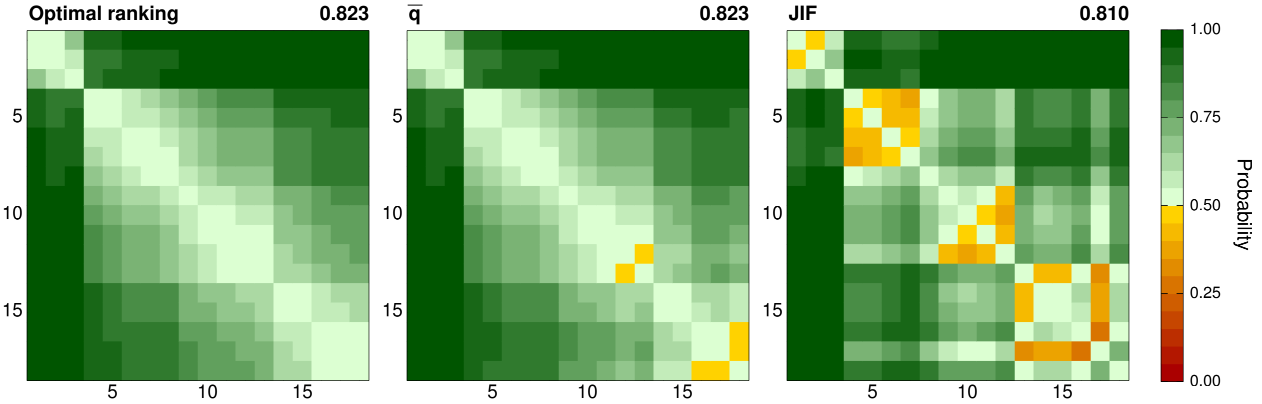

The color of each cell represents the probability of picking a paper with more citations from the higher-ranked journal. Green cells indicate adequate ranking, whereas red cells indicate that the ranking is inadequate (the probability is less than $\frac{1}{2}$). The value of M, the multi-class AUC statistic for each ranking scheme, is above each matrix.

Rank			$p_{ss}(q\|J)$		n			Steady-state
**AUC**	**JIF**	**Journal abbreviation**	$\bar{q}$	σ	$\bar{n}$	**Q2**	**JIF**	**period**
1	2	NATURE	2.13	0.51	216.7	131	26.681	1992–1997
2	1	SCIENCE	2.07	0.58	208.9	112	30.028	1987–1995
3	3	P NATL ACAD SCI USA	1.90	0.39	114.9	72	9.643	1985–1991
4	7	IBM J RES DEV	0.97	0.47	20.0	7	1.483	1956–2001
5	6	SCI AM	0.94	0.56	18.5	6	1.560	1963–1995
6	5	ANN NY ACAD SCI	0.79	0.53	10.6	4	1.930	1979–2000
7	4	NATURWISSENSCHAFTEN	0.74	0.52	10.7	4	2.021	1954–2002
8	8	P JPN ACAD B-PHYS	0.62	0.43	6.9	3	0.808	1976–2002
9	12	S AFR J SCI	0.51	0.54	5.1	2	0.602	1976–2000
10	9	CURR SCI INDIA	0.34	0.46	2.9	1	0.737	1960–2001
11	11	CHINESE SCI BULL	0.28	0.41	2.1	1	0.722	1998–2003
12	10	AN ACAD BRAS CIENC	0.21	0.46	2.1	1	0.737	1965–2004
13	17	J SCI IND RES INDIA	0.21	0.53	2.2	1	0.178	1960–1998
14	14	TECHNOL REV	-0.11	0.40	0.7	0	0.262	1977–2005
15	15	NATL ACAD SCI LETT	-0.18	0.43	0.5	0	0.210	1979–2005
16	13	SCIENTIST	-0.66	0.72	0.4	0	0.296	1992–1999
17	16	NEW SCI	-0.45	0.34	0.1	0	0.193	1998–2006
18	18	R&D MAG	-0.54	0.35	0.1	0	0.041	1990–2005

MYCOLOGY

ISI Category Description Mycology includes resources on topics that range from the general biology of fungi to fungal diseases of humans, animals and plants.

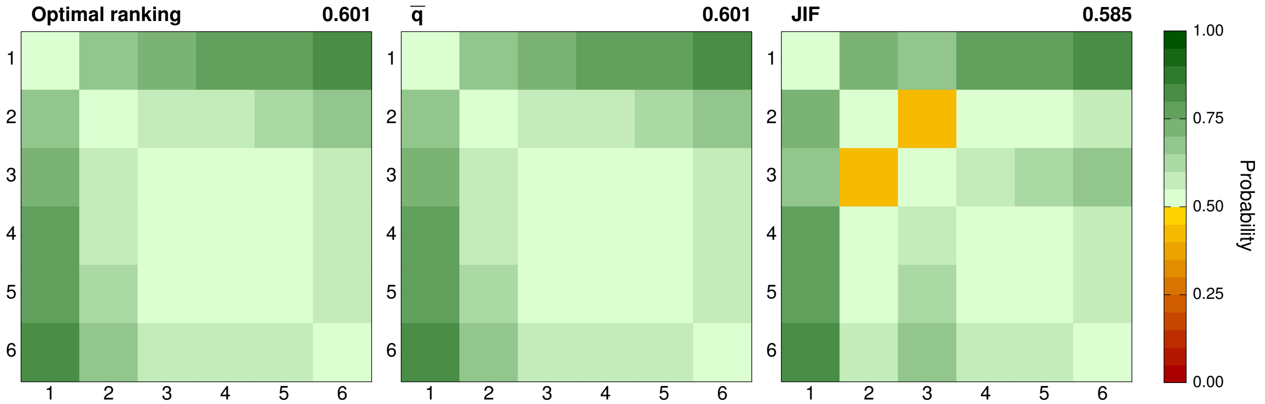

The color of each cell represents the probability of picking a paper with more citations from the higher-ranked journal. Green cells indicate adequate ranking, whereas red cells indicate that the ranking is inadequate (the probability is less than $\frac{1}{2}$). The value of M, the multi-class AUC statistic for each ranking scheme, is above each matrix.

Rank			$\mathbf{p_{ss}(q\|J)}$		n			Steady-state
**AUC**	**JIF**	**Journal abbreviation**	$\overline{\mathbf{q}}$	σ	$\overline{\mathbf{n}}$	**Q2**	**JIF**	**period**
1	1	YEAST	1.20	0.34	25.4	14	1.955	1992–1994
2	3	MYCOLOGIA	0.96	0.38	12.3	7	1.574	1955–2000
3	2	MYCOL RES	0.85	0.40	8.6	5	1.860	1996–2001
4	4	MYCOSES	0.81	0.36	8.0	5	0.959	1983–1999
5	5	MYCOPATHOLOGIA	0.79	0.39	7.9	5	0.915	1974–2000
6	6	MYCOTAXON	0.68	0.38	6.5	3	0.486	1977–1993

NANOSCIENCE & NANOTECHNOLOGY

ISI Category Description Nanoscience & Nanotechnology includes resources that focus on basic and applied research at the micro and nano level across a variety of disciplines including chemistry, biology, bioengineering, physics, electronics, clinical and medical science, chemical engineering and materials science.

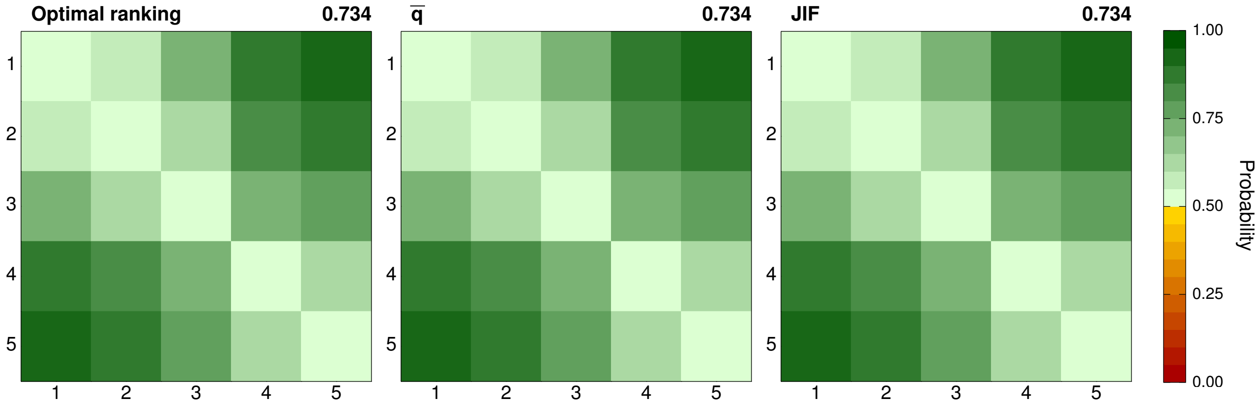

The color of each cell represents the probability of picking a paper with more citations from the higher-ranked journal. Green cells indicate adequate ranking, whereas red cells indicate that the ranking is inadequate (the probability is less than $\frac{1}{2}$). The value of M, the multi-class AUC statistic for each ranking scheme, is above each matrix.

Rank			$p_{ss}(q\|J)$		n			Steady-state
**AUC**	**JIF**	**Journal abbreviation**	$\overline{q}$	σ	$\overline{n}$	**Q2**	**JIF**	**period**
1	1	BIOSENS BIOELECTRON	1.25	0.41	21.1	15	4.132	1990–2000
2	2	J VAC SCI TECHNOL B	1.09	0.41	17.5	10	1.597	1987–1990
3	3	MAT SCI ENG A-STRUCT	0.85	0.39	9.1	5	1.490	1998–2000
4	4	MICROELECTRON ENG	0.48	0.44	3.9	2	1.398	1992–2003
5	5	MICROELECTRON RELIAB	0.27	0.44	2.2	1	0.815	1965–2004

NEUROIMAGING

ISI Category Description Neuroimaging covers resources on the mapping technologies used to treat, diagnose, or monitor brain lesions and mental disorders.

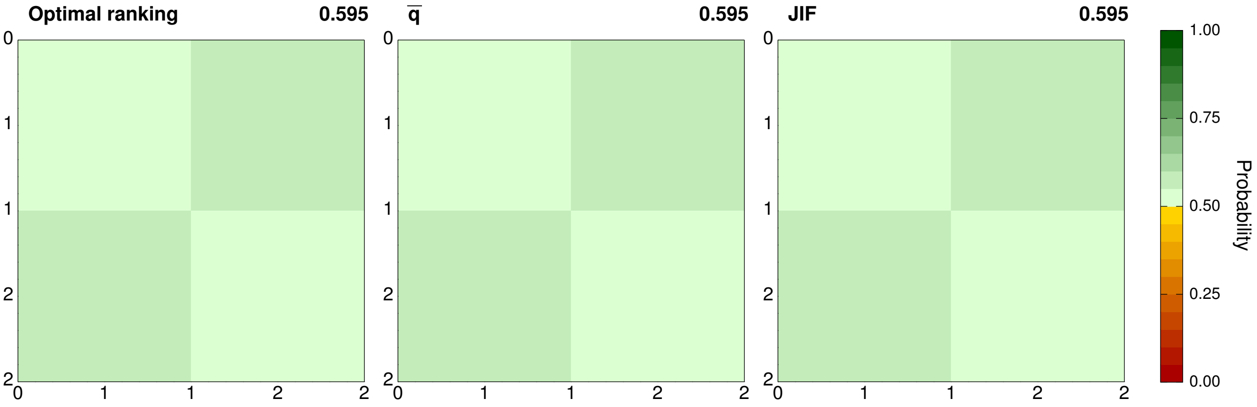

The color of each cell represents the probability of picking a paper with more citations from the higher-ranked journal. Green cells indicate adequate ranking, whereas red cells indicate that the ranking is inadequate (the probability is less than $\frac{1}{2}$). The value of M, the multi-class AUC statistic for each ranking scheme, is above each matrix.

**Rank**			$\mathbf{p_{ss}(q\|J)}$		$\mathbf{n}$			**Steady-state**
**AUC**	**JIF**	**Journal abbreviation**	$\overline{\mathbf{q}}$	σ	$\overline{\mathbf{n}}$	**Q2**	**JIF**	**period**
1	1	AM J NEURORADIOL	1.23	0.39	22.3	14	2.279	1979–2000
2	2	NEURORADIOLOGY	1.08	0.41	15.8	10	1.625	1971–1994

NEUROSCIENCES

ISI Category Description Neurosciences covers resources on all areas of basic research on the brain, neural physiology, and function in health and disease. The areas of focus include neurotransmitters, neuropeptides, neurochemistry, neural development, and neural behavior. Coverage also includes resources in neuro-endocrine and neuro-immune systems, somatosensory system, motor system and sensory motor integration, autonomic system as well as diseases of the nervous system.

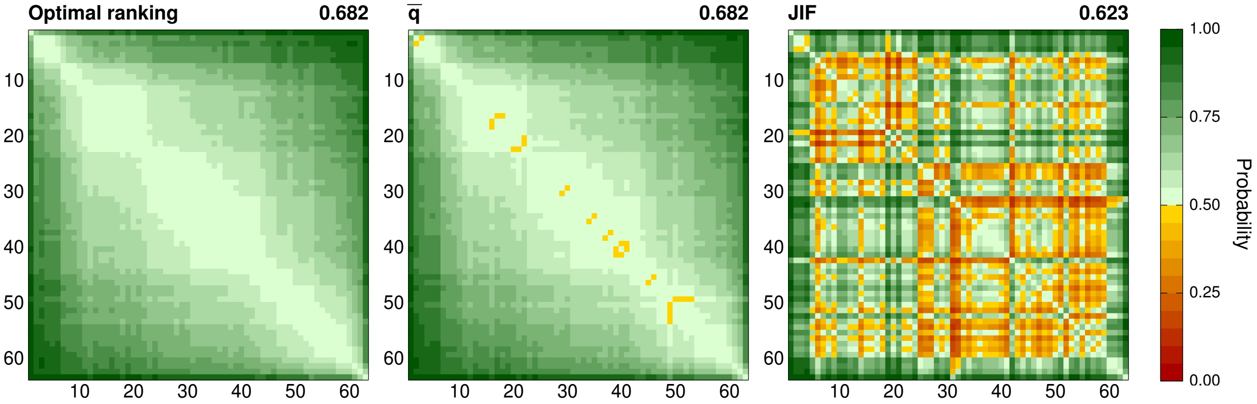

The color of each cell represents the probability of picking a paper with more citations from the higher-ranked journal. Green cells indicate adequate ranking, whereas red cells indicate that the ranking is inadequate (the probability is less than $\frac{1}{2}$). The value of M, the multi-class AUC statistic for each ranking scheme, is above each matrix.

Rank			$p_{ss}(q\|J)$		n			Steady-state
AUC	JIF	Journal abbreviation	$\bar{q}$	σ	$\bar{n}$	Q2	JIF	period
1	1	NEURON	2.08	0.31	150.1	118	13.894	1987–1997
2	19	J COMP NEUROL	1.82	0.40	105.9	63	3.831	1969–1980
3	4	J NEUROSCI	1.82	0.34	88.9	60	7.453	1980–1996
4	2	ANN NEUROL	1.77	0.38	83.5	55	8.051	1984–1993
5	3	BRAIN	1.78	0.40	82.8	55	7.617	1954–1996
6	21	J NEUROPHYSIOL	1.74	0.36	70.6	50	3.652	1981–1989
7	42	BRAIN RES	1.63	0.39	65.4	38	2.341	1974–1980
8	10	J CEREBR BLOOD F MET	1.59	0.38	56.6	35	4.843	1982–1996
9	11	PAIN	1.57	0.35	52.5	34	4.836	1979–1996
10	24	NEUROSCIENCE	1.55	0.37	47.7	31	3.427	1986–1993
11	54	J NEUROCYTOL	1.49	0.37	43.7	27	1.695	1971–1990
12	17	NEUROPSYCHOLOGIA	1.48	0.41	48.6	27	3.924	1964–1995
13	56	MOL BRAIN RES	1.47	0.33	37.2	26	1.670	1985–1995
14	13	J NEUROCHEM	1.47	0.36	41.2	26	4.260	1957–1997
15	51	EXP BRAIN RES	1.47	0.36	38.4	26	1.959	1984–1991
16	8	GLIA	1.46	0.31	36.6	26	5.013	1993–1998
17	28	BEHAV NEUROSCI	1.45	0.36	39.2	25	2.907	1984–1996
18	12	J NEUROPATH EXP NEUR	1.47	0.44	41.1	25	4.371	1956–1999
19	20	EUR J NEUROSCI	1.44	0.31	32.9	25	3.709	1995–1998
20	30	SYNAPSE	1.41	0.38	38.6	24	2.870	1987–1994
21	5	BIOL PSYCHIAT	1.42	0.33	34.0	23	7.154	2000–2002
22	58	HEARING RES	1.41	0.38	34.2	22	1.584	1980–1989

table continues on next page . . .

...table continued from previous page

Rank AUC	JIF	Journal abbreviation	$p_{ss}(q\|J)$ $\overline{q}$	σ	n $\overline{n}$	Q2	JIF	Steady-state period
23	23	J NEUROSCI RES	1.37	0.38	33.0	20	3.476	1987–1993
24	53	J COMP PHYSIOL A	1.37	0.31	26.4	21	1.751	1984–1989
25	22	PSYCHOPHARMACOLOGY	1.36	0.36	31.1	20	3.625	1975–1997
26	16	EXP NEUROL	1.35	0.36	30.5	19	4.156	1990–1999
27	15	J NEUROBIOL	1.35	0.36	30.6	20	4.209	1971–1999
28	45	VISION RES	1.35	0.40	31.6	19	2.167	1960–1994
29	36	NEUROENDOCRINOLOGY	1.33	0.34	27.6	18	2.680	1989–1995
30	57	DEV BRAIN RES	1.34	0.35	27.2	19	1.598	1982–1993
31	50	NEUROSCI LETT	1.31	0.40	30.2	17	2.092	1977–1990
32	29	J NEUROIMMUNOL	1.32	0.38	27.4	18	2.880	1980–1999
33	18	NEUROPHARMACOLOGY	1.31	0.37	28.2	17	3.860	1969–1999
34	59	VISUAL NEUROSCI	1.30	0.34	25.5	17	1.484	1990–1997
35	9	PSYCHONEUROENDOCRINO	1.30	0.36	25.2	17	4.850	1974–2001
36	35	ACTA NEUROPATHOL	1.29	0.39	27.6	17	2.694	1965–1996
37	7	NEUROBIOL AGING	1.28	0.35	23.5	17	5.599	1997–2001
38	37	BEHAV BRAIN RES	1.28	0.40	26.4	16	2.591	1979–1998
39	43	BRAIN LANG	1.25	0.33	22.9	16	2.317	1992–1997
40	33	J NEUROENDOCRINOL	1.25	0.30	22.2	16	2.774	1994–1999
41	38	MUSCLE NERVE	1.27	0.39	23.6	16	2.456	1977–1995
42	39	J NEUROL SCI	1.25	0.38	25.2	15	2.412	1977–1994
43	55	BRAIN RES BULL	1.24	0.40	25.4	14	1.684	1975–1990
44	48	NEUROREPORT	1.20	0.40	22.5	13	2.137	1990–1991
45	46	NEUROTOXICOL TERATOL	1.19	0.35	20.8	12	2.143	1979–1997
46	40	BEHAV PHARMACOL	1.20	0.34	17.9	13	2.388	1990–1998
47	49	PHARMACOL BIOCHEM BE	1.14	0.35	17.0	11	2.092	1991–1997
48	14	J PINEAL RES	1.14	0.36	17.3	12	4.228	1983–2002
49	44	J NEUROSCI METH	1.13	0.42	28.2	11	2.243	1980–1994
50	34	NEUROTOXICOLOGY	1.12	0.40	17.1	11	2.718	1980–1997
51	27	INT J DEV NEUROSCI	1.10	0.36	15.4	10	2.924	1983–2000
52	26	J NEURAL TRANSM	1.10	0.40	16.7	10	2.938	1985–1998
53	6	CEPHALALGIA	1.13	0.55	18.3	11	6.049	1988–1997
54	52	NEUROSCI RES	1.05	0.41	14.2	9	1.953	1986–2000
55	47	NEUROCHEM RES	1.03	0.37	14.0	9	2.139	1978–1997
56	41	NEUROPSYCHOBIOLOGY	1.02	0.38	13.6	8	2.367	1976–2000
57	25	NEUROCHEM INT	1.02	0.36	12.9	8	3.159	1981–2002
58	61	EUR NEUROL	0.97	0.38	12.3	8	1.031	1967–1997
59	60	BIOL CYBERN	0.98	0.37	11.2	7	1.474	1995–2002
60	32	NEUROPEPTIDES	0.92	0.34	9.7	7	2.789	1993–2002
61	62	INT J NEUROSCI	0.85	0.43	9.9	5	0.831	1971–1995
62	31	BRAIN COGNITION	0.73	0.61	9.1	3	2.858	1995–1999
63	63	ZH VYSSH NERV DEYAT+	0.38	0.42	2.6	1	0.379	1961–1998

NUCLEAR SCIENCE & TECHNOLOGY

ISI Category Description Nuclear Science & Technology covers resources on nuclear energy (fission and fusion processes), nuclear energy and fuel, nuclear power, and nuclear electric power generation. This category also includes resources on nuclear engineering (the branch of technology that applies the nuclear fission process to power generation), nuclear safety, radiation effects, and radioactive waste management. Note: Resources on nuclear physics (low-energy physics) appear in the category PHYSICS, NUCLEAR.

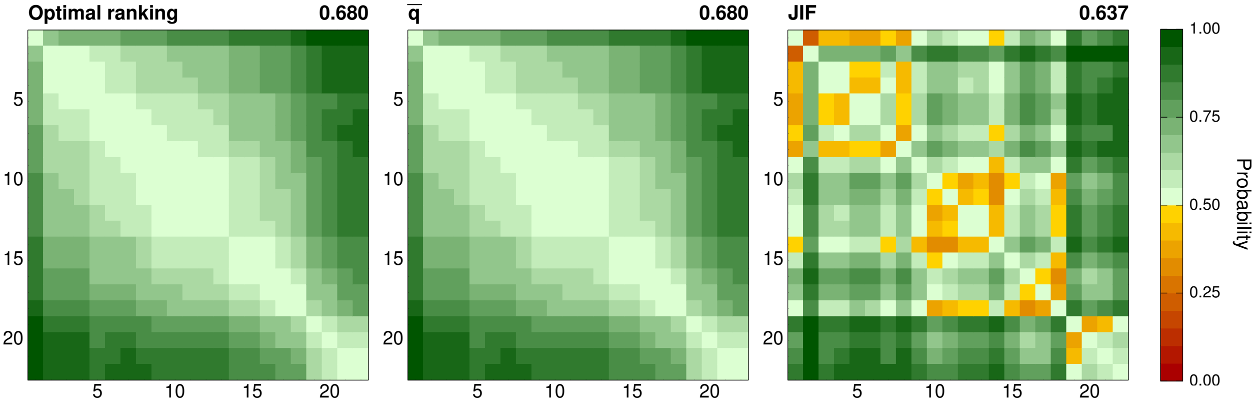

The color of each cell represents the probability of picking a paper with more citations from the higher-ranked journal. Green cells indicate adequate ranking, whereas red cells indicate that the ranking is inadequate (the probability is less than $\frac{1}{2}$). The value of M, the multi-class AUC statistic for each ranking scheme, is above each matrix.

Rank			$p_{ss}(q\|J)$		n			Steady-state
**AUC**	**JIF**	**Journal abbreviation**	$\overline{q}$	σ	$\overline{n}$	**Q2**	**JIF**	**period**
1	2	INT J RADIAT BIOL	1.16	0.38	20.4	12	1.312	1972–1999
2	8	HEALTH PHYS	0.89	0.43	10.5	6	0.902	1964–1994
3	5	RADIOCHIM ACTA	0.83	0.38	8.4	5	1.068	1965–2000
4	6	NUCL INSTRUM METH B	0.82	0.39	8.7	5	0.946	1988–1997
5	3	J NUCL MATER	0.79	0.50	8.1	4	1.261	1990–1994
6	4	NUCL INSTRUM METH A	0.73	0.44	8.3	4	1.185	1989–1994
7	14	NUCL SCI ENG	0.70	0.38	6.0	4	0.578	1985–1996
8	7	APPL RADIAT ISOTOPES	0.65	0.40	5.0	3	0.924	1993–2000
9	1	IEEE T NUCL SCI	0.63	0.48	6.8	3	1.497	1981–1998
10	9	RADIAT PHYS CHEM	0.60	0.44	5.1	2	0.868	1992–2000
11	18	RADIAT PROT DOSIM	0.57	0.43	4.6	2	0.446	1983–1999
12	12	J NUCL SCI TECHNOL	0.55	0.41	4.2	2	0.637	1970–1997
13	13	FUSION ENG DES	0.53	0.42	4.1	2	0.598	1987–2000
14	11	ANN NUCL ENERGY	0.44	0.39	2.9	2	0.690	1983–2001
15	15	NUCL TECHNOL	0.42	0.40	3.2	2	0.537	1986–2001
16	10	INT J ENERG RES	0.35	0.41	2.6	1	0.718	1976–2002
17	17	NUCL ENG DES	0.28	0.46	2.6	1	0.461	1981–2001
18	16	RADIAT EFF DEFECT S	0.19	0.41	1.6	1	0.497	1997–2005
19	20	KERNTECHNIK	-0.08	0.40	0.8	0	0.062	1998–2005
20	21	J ATOM ENERG SOC JPN	-0.26	0.53	0.5	0	0.043	1976–2003
21	19	NUCL ENG INT	-0.45	0.43	0.2	0	0.074	1969–2005
22	22	ATOM ENERGY+	-0.57	0.38	0.1	0	0.033	1999–2006

NURSING

ISI Category Description Nutrition & Dietetics covers resources concerning many aspects of nutrition, including general nutrition, nutrition and metabolism, nutrition science, clinical nutrition, vitamin research and nutritional biochemistry. Dietetics, the application of nutritional principles, is also included in this category.

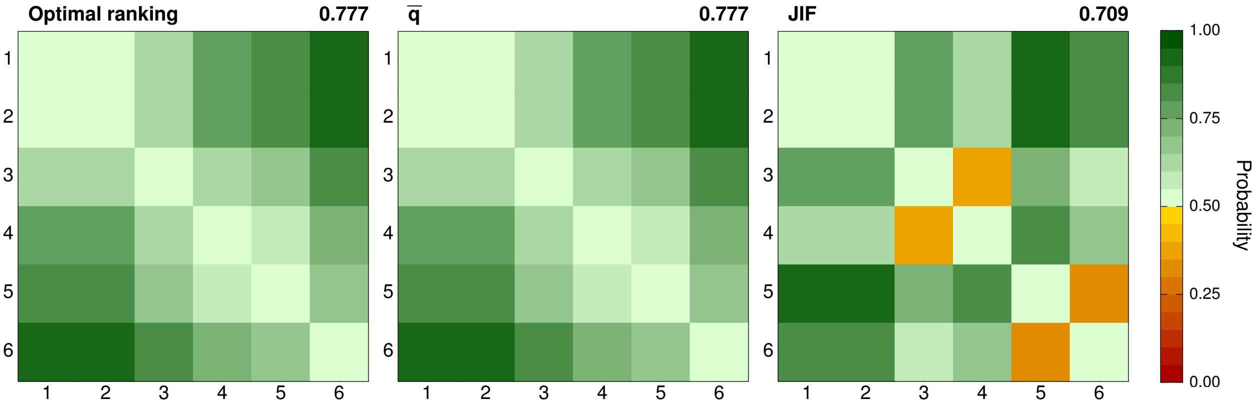

The color of each cell represents the probability of picking a paper with more citations from the higher-ranked journal. Green cells indicate adequate ranking, whereas red cells indicate that the ranking is inadequate (the probability is less than $\frac{1}{2}$). The value of M, the multi-class AUC statistic for each ranking scheme, is above each matrix.

Rank			$p_{ss}(q\|J)$		n			Steady-state
**AUC**	**JIF**	**Journal abbreviation**	$\overline{q}$	σ	$\overline{n}$	**Q2**	**JIF**	**period**
1	1	NURS RES	0.99	0.35	12.8	8	1.604	1995–2000
2	2	J ADV NURS	0.99	0.37	11.1	8	1.342	1986–1997
3	4	HEART LUNG	0.79	0.47	7.9	4	0.955	1986–2001
4	3	J NURS ADMIN	0.51	0.41	3.9	2	1.090	1981–2003
5	6	NURS CLIN N AM	0.37	0.40	2.8	1	0.432	1977–2002
6	5	AM J NURS	-0.05	0.48	1.1	0	0.711	1956–2005

NUTRITION & DIETETICS

ISI Category Description Nutrition & Dietetics covers resources concerning many aspects of nutrition, including general nutrition, nutrition and metabolism, nutrition science, clinical nutrition, vitamin research and nutritional biochemistry. Dietetics, the application of nutritional principles, is also included in this category.

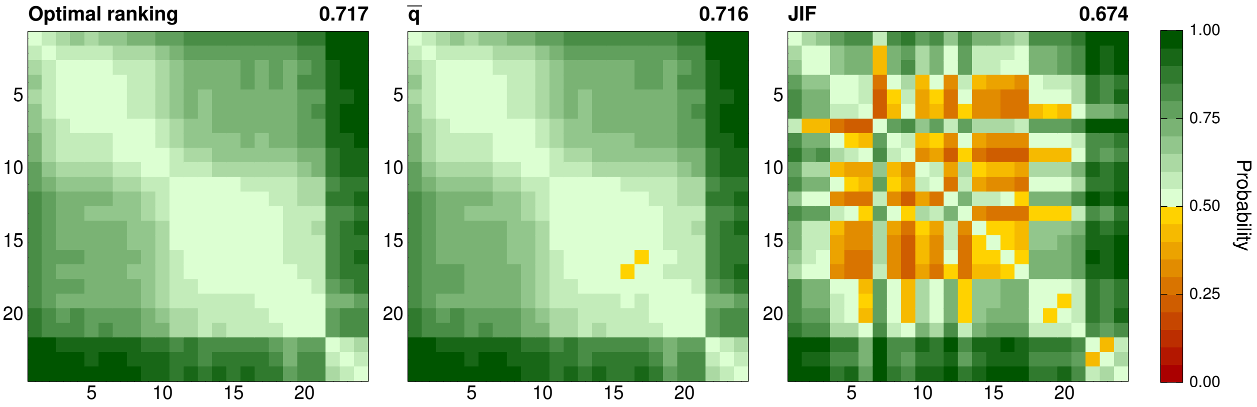

The color of each cell represents the probability of picking a paper with more citations from the higher-ranked journal. Green cells indicate adequate ranking, whereas red cells indicate that the ranking is inadequate (the probability is less than $\frac{1}{2}$). The value of M, the multi-class AUC statistic for each ranking scheme, is above each matrix.

Rank			$p_{ss}(q\|J)$		n			Steady-state
AUC	JIF	Journal abbreviation	$\overline{q}$	σ	$\overline{n}$	Q2	JIF	period
1	1	AM J CLIN NUTR	1.49	0.40	42.6	27	6.562	1978–1996
2	7	BRIT J NUTR	1.36	0.38	31.2	20	2.708	1955–1991
3	2	INT J OBESITY	1.28	0.39	25.0	16	4.055	1985–1999
4	3	J NUTR	1.26	0.36	22.6	15	4.009	1995–2000
5	17	INT J EAT DISORDER	1.24	0.40	25.8	15	1.839	1980–1997
6	12	NUTR CANCER	1.23	0.36	22.9	14	2.289	1987–2000
7	16	LIPIDS	1.20	0.35	19.3	13	1.935	1990–1995
8	15	J PEDIATR GASTR NUTR	1.17	0.36	17.5	12	2.067	1993–1995
9	14	EUR J CLIN NUTR	1.14	0.42	18.2	11	2.116	1987–1998
10	10	J AM COLL NUTR	1.07	0.39	14.3	9	2.452	1981–2002
11	4	P NUTR SOC	0.97	0.47	13.0	7	3.411	1970–2002
12	11	FOOD CHEM	0.91	0.37	9.9	6	2.433	1976–2002
13	8	J AM DIET ASSOC	0.90	0.48	12.7	6	2.564	1973–2000
14	5	J NUTR BIOCHEM	0.87	0.37	9.1	6	2.945	1989–2002
15	18	REPROD NUTR DEV	0.87	0.40	9.1	6	1.817	1979–2000
16	20	J NUTR SCI VITAMINOL	0.86	0.35	8.7	5	0.758	1972–1998
17	19	INT J VITAM NUTR RES	0.86	0.40	9.1	5	0.862	1970–2000
18	13	NUTRITION	0.82	0.45	9.5	5	2.229	1988–2002
19	6	NUTR REV	0.80	0.62	10.8	4	2.937	1977–2006
20	9	CLIN NUTR	0.74	0.41	7.0	4	2.474	1983–2003
21	21	NUTR RES	0.71	0.40	6.2	4	0.728	1987–1997
22	23	ARCH LATINOAM NUTR	0.21	0.40	1.6	1	0.258	1975–2001
23	22	FOOD DRUG LAW J	0.04	0.48	1.2	0	0.397	1972–2005
24	24	ERNAHRUNGS-UMSCHAU	-0.11	0.46	0.8	0	0.190	1976–2003

OBSTETRICS & GYNECOLOGY

ISI Category Description Obstetrics & Gynecology covers resources on the medical fields concerned with female reproductive function and reproductive organs. Obstetrics covers resources on pregnancy, fetal health, labor, and puerperium. Gynecology covers resources on the health and diseases of female sex organs and their impact on women's overall health. This category also includes resources on fertility, infertility, and contraception.

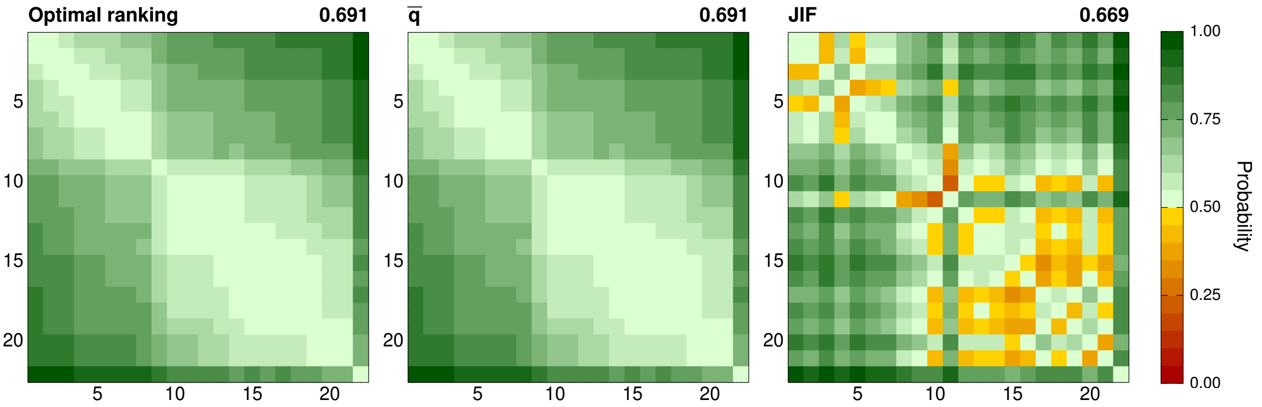

The color of each cell represents the probability of picking a paper with more citations from the higher-ranked journal. Green cells indicate adequate ranking, whereas red cells indicate that the ranking is inadequate (the probability is less than $\frac{1}{2}$). The value of M, the multi-class AUC statistic for each ranking scheme, is above each matrix.

Rank			$p_{ss}(q\|J)$		n			Steady-state
AUC	JIF	Journal abbreviation	$\overline{q}$	σ	$\overline{n}$	Q2	JIF	period
1	3	FERTIL STERIL	1.39	0.38	32.0	21	3.277	1978–1993
2	5	AM J OBSTET GYNECOL	1.33	0.40	30.2	18	2.805	1971–1996
3	1	OBSTET GYNECOL	1.30	0.40	27.3	17	3.813	1979–1995
4	2	HUM REPROD	1.24	0.40	23.0	15	3.769	1988–1997
5	6	GYNECOL ONCOL	1.23	0.37	20.9	14	2.319	1990–1995
6	7	ULTRASOUND OBST GYN	1.15	0.35	18.5	12	2.288	1995–1997
7	11	PRENATAL DIAG	1.15	0.34	17.1	12	1.514	1985–1995
8	4	PLACENTA	1.15	0.37	17.9	11	2.969	1980–2000
9	8	CONTRACEPTION	0.99	0.41	12.6	8	1.882	1970–1999
10	17	CLIN OBSTET GYNECOL	0.90	0.40	9.4	6	0.928	1985–1997
11	9	EARLY HUM DEV	0.91	0.39	9.7	6	1.738	1991–2000
12	19	J REPROD MED	0.87	0.39	9.9	6	0.867	1976–1997
13	21	AM J PERINAT	0.84	0.36	7.6	5	0.720	1986–1998
14	13	ACTA OBSTET GYN SCAN	0.82	0.49	9.1	5	1.327	1961–2000
15	18	GYNECOL OBSTET INVES	0.77	0.37	7.1	4	0.874	1976–2000
16	14	EUR J OBSTET GYN R B	0.76	0.41	7.2	4	1.273	1972–2000
17	10	CURR OPIN OBSTET GYN	0.76	0.36	6.4	4	1.634	1992–2003
18	12	INT J GYNECOL CANCER	0.74	0.39	6.3	4	1.469	1990–2002
19	16	J PERINAT MED	0.70	0.42	6.5	4	1.000	1972–2001
20	20	AUST NZ J OBSTET GYN	0.63	0.41	5.1	3	0.835	1968–2000
21	15	INT J GYNECOL OBSTET	0.63	0.43	5.1	3	1.078	1973–2001
22	22	GEBURTSH FRAUENHEILK	0.15	0.42	1.6	1	0.693	1996–2000

OCEANOGRAPHY

ISI Category Description Oceanography covers resources concerning the scientific study and exploration of the oceans and seas in all their aspects, including the delimitation of their extent and depth, the physics and chemistry of their waters, and the exploration of their resources.

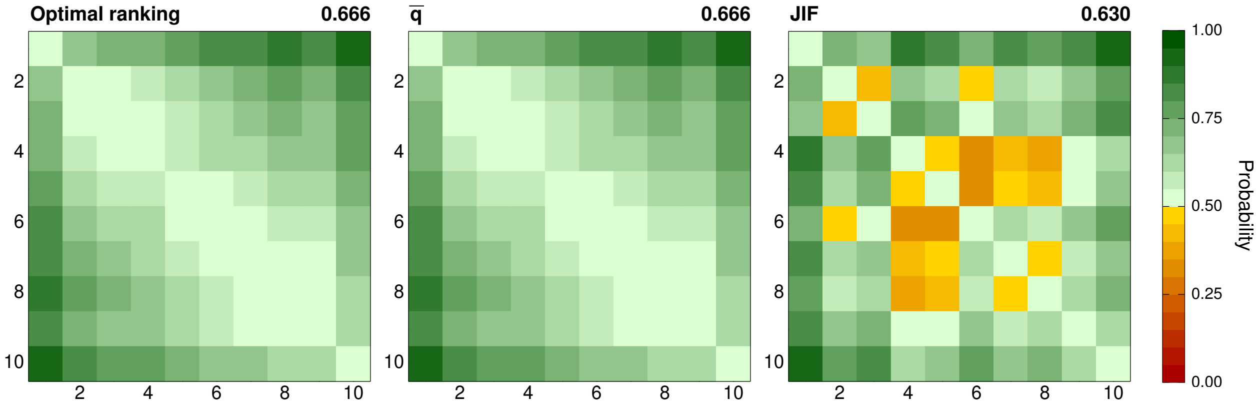

The color of each cell represents the probability of picking a paper with more citations from the higher-ranked journal. Green cells indicate adequate ranking, whereas red cells indicate that the ranking is inadequate (the probability is less than $\frac{1}{2}$). The value of M, the multi-class AUC statistic for each ranking scheme, is above each matrix.

Rank			$p_{ss}(q\|J)$		n			Steady-state
**AUC**	**JIF**	**Journal abbreviation**	$\bar{q}$	σ	$\bar{n}$	**Q2**	**JIF**	**period**
1	1	LIMNOL OCEANOGR	1.71	0.36	65.0	47	3.287	1970–1992
2	3	MAR ECOL-PROG SER	1.47	0.31	33.6	26	2.286	1991–1995
3	6	J PHYS OCEANOGR	1.40	0.37	36.4	22	1.838	1973–1994
4	2	MAR CHEM	1.38	0.36	31.1	20	2.663	1976–1996
5	8	J PLANKTON RES	1.30	0.35	23.9	17	1.617	1985–1995
6	7	ESTUAR COAST SHELF S	1.25	0.35	21.2	15	1.733	1980–1997
7	5	MAR GEOL	1.18	0.38	19.4	13	2.029	1964–1999
8	4	CONT SHELF RES	1.13	0.35	14.9	11	2.030	1996–2000
9	9	B MAR SCI	1.12	0.39	17.9	11	1.093	1965–1995
10	10	NEW ZEAL J MAR FRESH	0.94	0.37	10.7	7	0.931	1976–1998

ONCOLOGY

ISI Category Description Oncology covers resources on the mechanisms, causes, and treatments of cancer including environmental and genetic risk factors, and cellular and molecular carcinogenesis. Aspects of clinical oncology covered include surgical, radiological, chemical, and palliative care. This category is also concerned with resources on cancers of specific systems and organs.

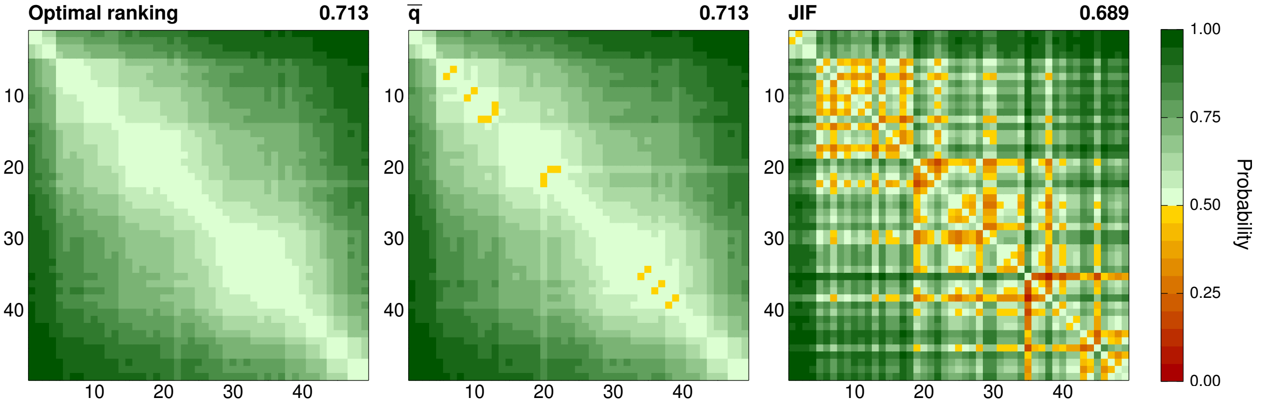

The color of each cell represents the probability of picking a paper with more citations from the higher-ranked journal. Green cells indicate adequate ranking, whereas red cells indicate that the ranking is inadequate (the probability is less than $\frac{1}{2}$). The value of M, the multi-class AUC statistic for each ranking scheme, is above each matrix.

Rank			$p_{ss}(q\|J)$		n			Steady-state
**AUC**	**JIF**	**Journal abbreviation**	$\overline{q}$	σ	$\overline{n}$	**Q2**	**JIF**	**period**
1	2	J CLIN ONCOL	1.82	0.34	85.9	61	13.598	1987–1993
2	1	J NATL CANCER I	1.80	0.40	90.9	57	15.271	1989–2000
3	3	CANCER RES	1.69	0.35	63.1	43	7.656	1997–1999
4	4	ONCOGENE	1.66	0.36	60.4	41	6.582	1992–1995
5	17	GENE CHROMOSOME CANC	1.48	0.33	35.4	27	3.900	1995–1998
6	10	CANCER	1.44	0.37	36.3	24	4.582	1983–1997
7	14	CANCER EPIDEM BIOMAR	1.46	0.35	32.9	25	4.289	1991–2000
8	12	BRIT J CANCER	1.44	0.35	34.3	24	4.459	1993–1995
9	6	CARCINOGENESIS	1.38	0.35	31.1	21	5.366	1979–2000
10	8	INT J CANCER	1.39	0.36	28.3	21	4.693	1996–1999
11	22	MOL CARCINOGEN	1.35	0.36	29.1	20	2.743	1988–1994
12	11	INT J RADIAT ONCOL	1.37	0.41	31.2	20	4.463	1982–1995
13	18	EXP CELL RES	1.35	0.38	33.9	19	3.777	1955–1996
14	9	BREAST CANCER RES TR	1.28	0.40	26.2	16	4.671	1983–1998
15	5	LEUKEMIA	1.26	0.37	22.5	15	6.146	1995–2000
16	38	CANCER GENET CYTOGEN	1.24	0.39	25.2	14	1.544	1980–1990
17	30	NUTR CANCER	1.23	0.36	22.9	14	2.289	1987–2000
18	16	RADIOTHER ONCOL	1.22	0.38	22.5	14	3.970	1983–2000
19	29	GYNECOL ONCOL	1.23	0.37	20.9	14	2.319	1990–1995
20	15	EUR J CANCER	1.20	0.39	21.9	13	4.167	1968–2000
21	21	SEMIN ONCOL	1.19	0.37	19.1	13	3.064	1993–1996
22	23	BONE MARROW TRANSPL	1.21	0.51	20.5	14	2.621	1993–1995
23	7	ANN ONCOL	1.15	0.38	18.2	11	5.179	1998–2000

table continues on next page . . .

. . . table continued from previous page

Rank			$p_{ss}(q\|J)$		n			Steady-state
**AUC**	**JIF**	**Journal abbreviation**	$\overline{q}$	σ	$\overline{n}$	**Q2**	**JIF**	**period**
24	13	CANCER IMMUNOL IMMUN	1.11	0.33	15.9	11	4.313	1982–2000
25	27	CANCER CHEMOTH PHARM	1.09	0.36	16.0	10	2.363	1988–1999
26	34	J NEURO-ONCOL	1.08	0.36	14.0	10	1.848	1983–2000
27	20	CANCER LETT	1.04	0.36	12.7	9	3.277	1997–2001
28	26	J CANCER RES CLIN	1.01	0.38	12.9	8	2.469	1978–2001
29	24	INT J ONCOL	0.98	0.39	11.6	8	2.556	1997–2000
30	45	AM J CLIN ONCOL-CANC	0.96	0.35	11.8	7	1.224	1981–1998
31	40	ANTICANCER RES	0.95	0.37	11.2	7	1.479	1980–1999
32	31	ANTI-CANCER DRUG	0.95	0.35	10.9	7	2.245	1990–2001
33	37	LEUKEMIA LYMPHOMA	0.94	0.37	10.1	7	1.559	1992–1997
34	33	ACTA ONCOL	0.91	0.38	11.2	6	1.856	1984–2003
35	25	LEUKEMIA RES	0.91	0.33	9.4	6	2.483	1996–2000
36	32	J SURG ONCOL	0.93	0.43	9.9	7	2.183	1988–1999
37	19	STRAHLENTHER ONKOL	0.91	0.32	8.1	6	3.682	2000–2004
38	28	CANCER INVEST	0.87	0.44	10.5	6	2.335	1982–2001
39	36	CANCER DETECT PREV	0.88	0.42	9.3	5	1.622	1989–2000
40	39	CHEMOTHERAPY	0.81	0.38	8.3	5	1.511	1963–2000
41	42	JPN J CLIN ONCOL	0.77	0.41	7.3	4	1.376	1982–1998
42	41	INT J GYNECOL CANCER	0.74	0.39	6.3	4	1.469	1990–2002
43	48	TUMORI	0.69	0.39	6.1	4	0.701	1975–1998
44	47	J EXP CLIN CANC RES	0.62	0.48	4.8	3	0.869	1996–2000
45	44	NEOPLASMA	0.56	0.38	4.1	2	1.247	1962–2001
46	49	FOLIA BIOL-PRAGUE	0.55	0.40	4.1	2	0.387	1973–2000
47	46	B CANCER	0.42	0.43	3.2	1	0.906	1984–1999
48	43	J CHEMOTHERAPY	0.41	0.53	3.4	1	1.374	1988–2003
49	35	ONKOLOGIE	0.36	0.45	2.7	1	1.724	1977–2003

OPERATIONS RESEARCH & MANAGEMENT SCIENCE

ISI Category Description Operations Research & Management Science includes resources on the definition, analysis, and solution of complex problems. Relevant topics in this category include mathematical modeling, stochastic modeling, decision theory and systems, optimization theory, logistics, and control theory.

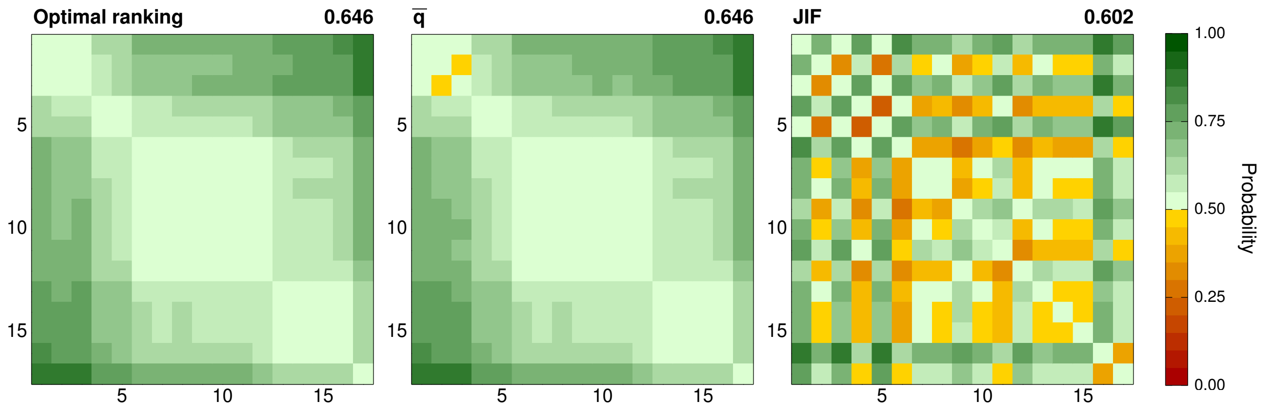

The color of each cell represents the probability of picking a paper with more citations from the higher-ranked journal. Green cells indicate adequate ranking, whereas red cells indicate that the ranking is inadequate (the probability is less than $\frac{1}{2}$). The value of M, the multi-class AUC statistic for each ranking scheme, is above each matrix.

Rank			$p_{ss}(q\|J)$		n			Steady-state
AUC	JIF	Journal abbreviation	$\overline{q}$	σ	$\overline{n}$	Q2	JIF	period
1	1	MANAGE SCI	1.17	0.48	27.1	12	1.687	1957–1997
2	3	OPER RES	1.10	0.45	22.1	10	1.234	1958–1997
3	5	MATH PROGRAM	1.11	0.43	18.6	10	1.117	1976–1998
4	9	INT J PROD RES	0.96	0.49	13.4	7	0.799	1986–1992
5	12	IIE TRANS	0.89	0.44	11.3	6	0.637	1981–1997
6	7	EUR J OPER RES	0.77	0.44	8.6	4	0.918	1977–1999
7	15	NAV RES LOG	0.76	0.47	8.3	4	0.362	1971–1994
8	14	J OPER RES SOC	0.74	0.42	7.8	4	0.597	1977–1997
9	10	OPER RES LETT	0.73	0.44	7.3	4	0.767	1982–1999
10	2	SYST CONTROL LETT	0.71	0.41	7.6	4	1.683	1994–2000
11	8	COMPUT OPER RES	0.72	0.42	7.1	3	0.893	1975–2000
12	13	J OPTIMIZ THEORY APP	0.70	0.45	7.7	3	0.633	1974–1998
13	17	INTERFACES	0.58	0.47	6.1	2	0.338	1973–2001
14	4	INT J PROD ECON	0.58	0.44	4.2	2	1.183	1990–2002
15	11	OMEGA-INT J MANAGE S	0.56	0.44	5.3	2	0.663	1974–2003
16	6	RELIAB ENG SYST SAFE	0.52	0.42	4.0	2	0.920	1981–2002
17	16	INT J SYST SCI	0.28	0.43	2.3	1	0.343	1986–2001

OPHTHALMOLOGY

ISI Category Description Ophthalmology covers resources on the eye, its diseases, and refractive errors. Coverage includes research on the cornea, retina, and eye diseases. This category also includes resources on physiological optics and optometry as well as reconstructive surgery.

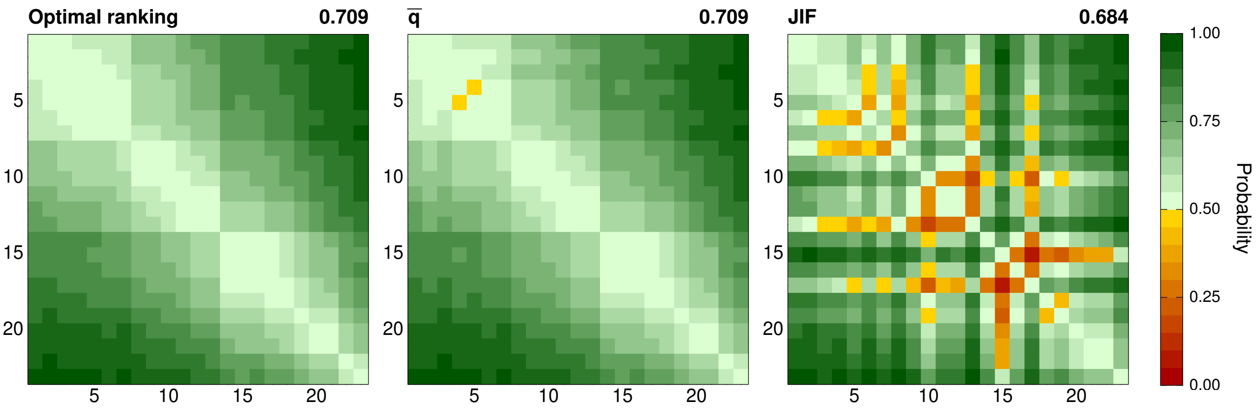

The color of each cell represents the probability of picking a paper with more citations from the higher-ranked journal. Green cells indicate adequate ranking, whereas red cells indicate that the ranking is inadequate (the probability is less than $\frac{1}{2}$). The value of M, the multi-class AUC statistic for each ranking scheme, is above each matrix.

Rank			$p_{ss}(q\|J)$		n			Steady-state
**AUC**	**JIF**	**Journal abbreviation**	$\overline{q}$	σ	$\overline{n}$	**Q2**	**JIF**	**period**
1	1	OPHTHALMOLOGY	1.39	0.39	32.6	21	4.031	1981–1994
2	2	INVEST OPHTH VIS SCI	1.35	0.34	27.3	19	3.766	1979–1999
3	8	VISION RES	1.35	0.40	31.6	19	2.167	1960–1994
4	13	VISUAL NEUROSCI	1.30	0.34	25.5	17	1.484	1990–1997
5	6	AM J OPHTHALMOL	1.30	0.38	25.9	17	2.468	1970–1995
6	3	ARCH OPHTHALMOL-CHIC	1.27	0.40	25.5	16	3.206	1961–1992
7	4	EXP EYE RES	1.25	0.36	23.1	15	2.776	1961–1996
8	17	CURR EYE RES	1.12	0.35	15.7	11	1.208	1983–1995
9	5	BRIT J OPHTHALMOL	1.12	0.39	16.9	11	2.524	1963–2000
10	7	J CATARACT REFR SURG	1.10	0.39	15.6	10	2.285	1985–2000
11	9	EYE	1.03	0.38	13.5	9	2.084	1986–1996
12	11	CORNEA	0.98	0.38	11.3	7	1.708	1986–2001
13	12	GRAEF ARCH CLIN EXP	0.95	0.38	11.6	7	1.609	1982–1995
14	14	OPHTHAL PHYSL OPT	0.78	0.43	7.1	4	1.483	1980–2002
15	16	OPTOMETRY VISION SCI	0.76	0.45	7.6	4	1.371	1988–2001
16	19	JPN J OPHTHALMOL	0.74	0.40	7.7	4	0.770	1972–1996
17	10	DOC OPHTHALMOL	0.68	0.42	6.7	3	1.712	1965–1999
18	18	OPHTHALMOLOGICA	0.64	0.40	5.5	3	1.051	1963–2001
19	20	CAN J OPHTHALMOL	0.56	0.44	4.2	2	0.701	1995–2001
20	21	KLIN MONATSBL AUGENH	0.44	0.40	3.2	2	0.679	1973–2002
21	22	J FR OPHTALMOL	0.39	0.38	2.4	1	0.291	1977–2000
22	15	RETINA-J RET VIT DIS	0.17	0.52	2.1	0	1.403	2003–2006
23	23	ANN OPHTHALMOL	-0.04	0.61	1.5	0	0.075	1993–2005

OPTICS

ISI Category Description Optics includes resources that deal with the genesis and propagation of light, the changes that it undergoes and produces, and other phenomena closely associated with it. Resources in this category cover subject areas such as lasers and laser technology, infrared physics and technology, microwave technology, quantum optics, lightwave technology, fiber optics, opto-electronics, and photonics. Resources on photometry and luminescence are also included in this category.

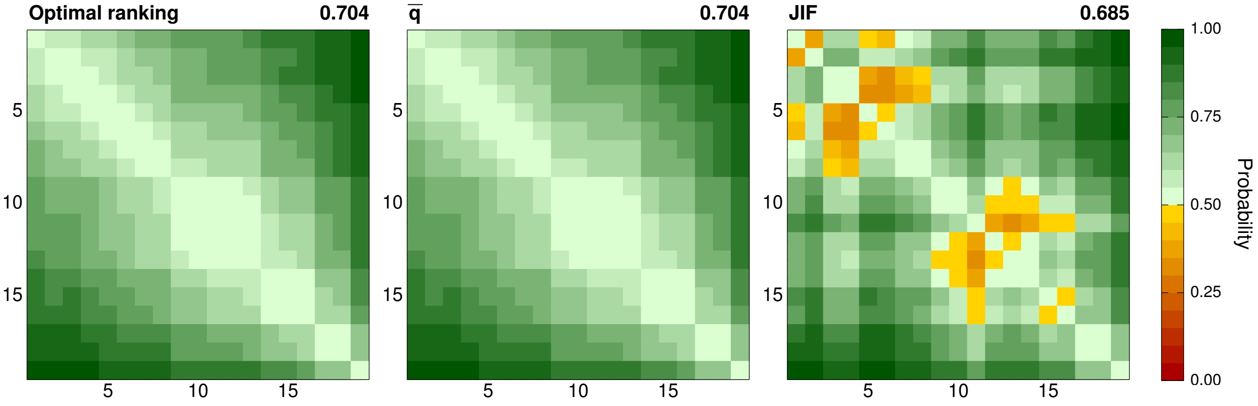

The color of each cell represents the probability of picking a paper with more citations from the higher-ranked journal. Green cells indicate adequate ranking, whereas red cells indicate that the ranking is inadequate (the probability is less than $\frac{1}{2}$). The value of M, the multi-class AUC statistic for each ranking scheme, is above each matrix.

**Rank**			$\mathbf{p_{ss}(q\|J)}$		**n**			**Steady-state**
**AUC**	**JIF**	**Journal abbreviation**	$\overline{\mathbf{q}}$	σ	$\overline{\mathbf{n}}$	**Q2**	**JIF**	**period**
1	2	PHYS REV A	1.32	0.42	33.2	18	3.047	1981–1986
2	6	J OPT SOC AM B	1.24	0.43	28.6	14	2.002	1983–1990
3	5	J PHYS B-AT MOL OPT	1.20	0.37	20.4	13	2.024	1981–1986
4	1	OPT LETT	1.14	0.40	20.0	11	3.598	1995–1998
5	7	J OPT SOC AM A	1.11	0.43	21.5	10	2.002	1985–1995
6	8	APPL OPTICS	1.03	0.44	17.9	8	1.717	1962–1987
7	3	J LIGHTWAVE TECHNOL	0.97	0.41	14.2	7	2.824	1983–2000
8	4	IEEE PHOTONIC TECH L	0.93	0.37	10.5	7	2.353	1994–2000
9	13	IMAGE VISION COMPUT	0.79	0.40	8.2	5	1.171	1984–2001
10	9	OPT COMMUN	0.79	0.41	7.9	5	1.480	1995–1998
11	12	J MOD OPTIC	0.73	0.43	8.4	4	1.189	1986–2000
12	14	OPT ENG	0.72	0.46	8.6	4	0.897	1972–1995
13	10	J LUMIN	0.71	0.45	7.2	4	1.441	1993–2002
14	16	OPTIK	0.56	0.45	6.2	2	0.585	1963–1995
15	15	OPT QUANT ELECTRON	0.51	0.41	4.3	2	0.644	1992–2002
16	11	MICROELECTRON ENG	0.48	0.44	3.9	2	1.398	1992–2003
17	17	MICROW OPT TECHN LET	0.24	0.45	2.4	1	0.568	1987–2002
18	18	INT J INFRARED MILLI	0.17	0.46	1.7	1	0.326	1994–2002
19	19	LASER FOCUS WORLD	-0.27	0.50	0.6	0	0.249	1983–2005

ORNITHOLOGY

ISI Category Description Ornithology covers resources concerning many aspects of the study of birds, including avian biology, field ornithology, avian biochemistry and physiology, avian systematics and taxonomy, raptor research, bird behavior and migration.

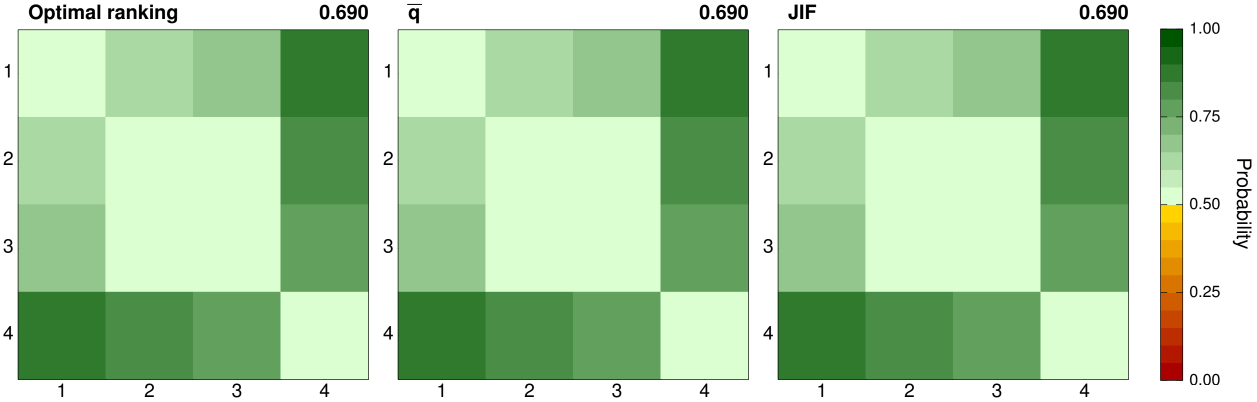

The color of each cell represents the probability of picking a paper with more citations from the higher-ranked journal. Green cells indicate adequate ranking, whereas red cells indicate that the ranking is inadequate (the probability is less than $\frac{1}{2}$). The value of M, the multi-class AUC statistic for each ranking scheme, is above each matrix.

Rank			$\mathbf{p_{ss}(q\|J)}$		**n**			**Steady-state**
**AUC**	**JIF**	**Journal abbreviation**	$\overline{\mathbf{q}}$	σ	$\overline{\mathbf{n}}$	**Q2**	**JIF**	**period**
1	1	AUK	1.46	0.37	36.1	25	2.056	1977–1987
2	2	CONDOR	1.26	0.36	22.8	15	1.604	1965–1994
3	3	IBIS	1.22	0.38	24.5	14	1.595	1966–1996
4	4	J FIELD ORNITHOL	0.76	0.38	6.5	4	0.660	1985–1999

ORTHOPEDICS

ISI Category Description Orthopedics covers resources on surgery and medical appliances as a means to preserve or restore function or alleviate pain in the musculoskeletal system, particularly the bones and joints.

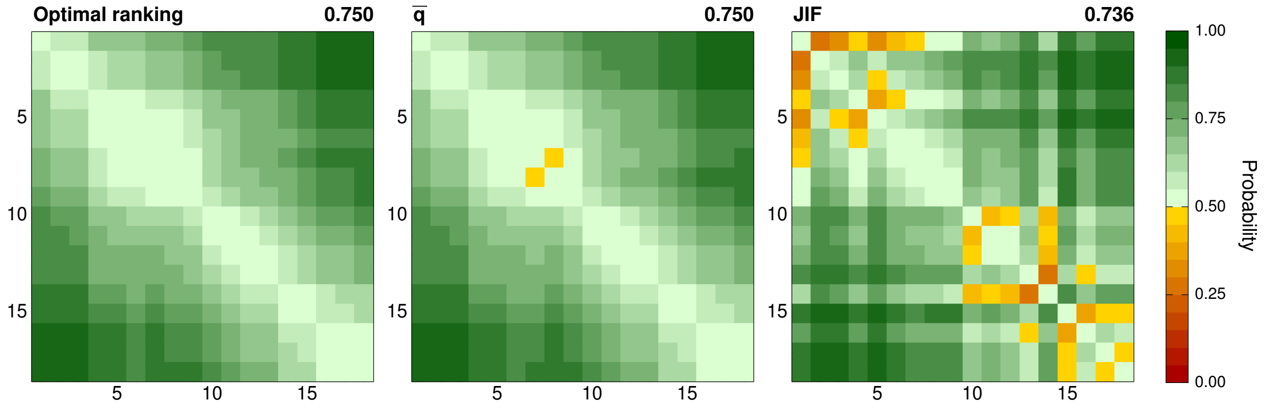

The color of each cell represents the probability of picking a paper with more citations from the higher-ranked journal. Green cells indicate adequate ranking, whereas red cells indicate that the ranking is inadequate (the probability is less than $\frac{1}{2}$). The value of M, the multi-class AUC statistic for each ranking scheme, is above each matrix.

Rank			$p_{ss}(q\|J)$		n			Steady-state
AUC	JIF	Journal abbreviation	$\overline{q}$	σ	$\overline{n}$	Q2	JIF	period
1	2	J BONE JOINT SURG AM	1.46	0.45	43.3	25	2.444	1965–1995
2	5	J BONE JOINT SURG BR	1.36	0.39	30.6	19	1.790	1955–1995
3	3	SPINE	1.34	0.39	29.8	19	2.351	1978–1995
4	6	ARTHROSCOPY	1.20	0.43	18.6	15	1.574	1991–1992
5	4	CLIN ORTHOP RELAT R	1.16	0.38	17.6	12	2.161	1995–1998
6	7	PHYS THER	1.14	0.44	18.4	11	1.510	1989–1997
7	1	ORTHOP CLIN N AM	1.09	0.33	12.9	10	2.500	1995–1997
8	8	J HAND SURG-AM	1.10	0.38	13.8	11	1.286	1993–1995
9	9	J PEDIATR ORTHOPED	1.04	0.35	12.3	9	1.152	1980–1997
10	14	HAND CLIN	0.91	0.35	9.0	6	0.661	1985–1996
11	11	J HAND SURG-BRIT EUR	0.85	0.37	8.3	5	0.844	1983–1996
12	12	ARCH ORTHOP TRAUM SU	0.77	0.39	7.3	4	0.793	1978–1999
13	10	INT ORTHOP	0.75	0.38	7.0	4	0.977	1976–1999
14	16	ORTHOPEDICS	0.63	0.47	5.9	3	0.583	1977–1999
15	13	ORTHOPADE	0.58	0.40	4.2	3	0.707	1977–2001
16	18	REV CHIR ORTHOP	0.43	0.41	3.2	2	0.344	1971–2001
17	17	J AM PODIAT MED ASSN	0.41	0.46	3.3	1	0.549	1988–2001
18	15	Z ORTHOP GRENZGEB	0.35	0.43	2.6	1	0.630	1971–2002

OTORHINOLARYNGOLOGY

ISI Category Description Otorhinolaryngology covers resources on the basic and clinical research and medicine of the ears, nose, and throat. This category also includes voice and audiology resources.

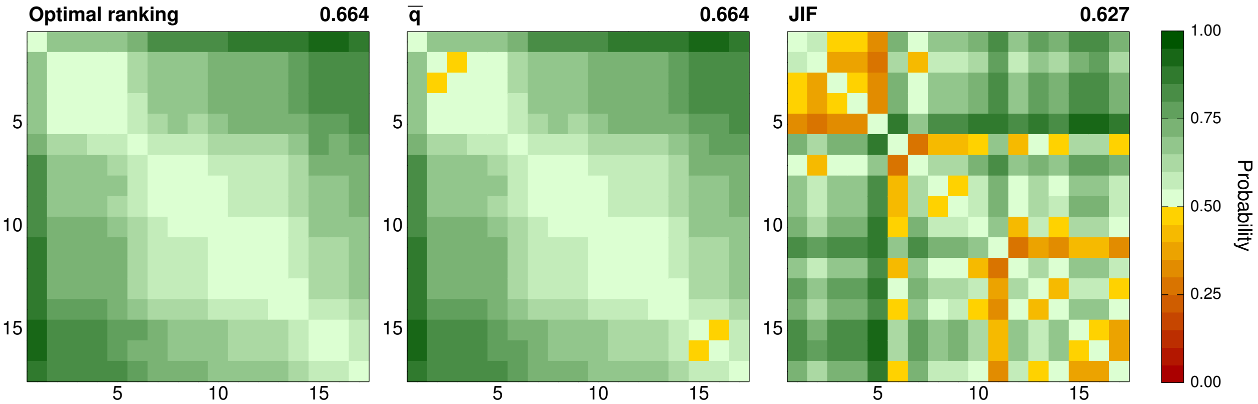

The color of each cell represents the probability of picking a paper with more citations from the higher-ranked journal. Green cells indicate adequate ranking, whereas red cells indicate that the ranking is inadequate (the probability is less than $\frac{1}{2}$). The value of M, the multi-class AUC statistic for each ranking scheme, is above each matrix.

Rank			$p_{ss}(q\|J)$		n			Steady-state
AUC	JIF	Journal abbreviation	$\overline{q}$	σ	$\overline{n}$	Q2	JIF	period
1	5	HEARING RES	1.41	0.38	34.2	22	1.584	1980–1989
2	4	ARCH OTOLARYNGOL	1.18	0.40	19.6	13	1.734	1973–1995
3	3	LARYNGOSCOPE	1.19	0.42	19.4	13	1.736	1979–1994
4	1	HEAD NECK-J SCI SPEC	1.15	0.37	17.0	11	1.961	1988–1999
5	7	ANN OTO RHINOL LARYN	1.13	0.42	17.6	11	1.096	1960–1995
6	2	EAR HEARING	1.01	0.41	14.0	8	1.858	1981–2000
7	9	INT J PEDIATR OTORHI	0.91	0.37	9.2	6	0.846	1978–1995
8	12	AM J OTOLARYNG	0.88	0.38	8.7	6	0.737	1988–1997
9	8	OTOLARYNG CLIN N AM	0.88	0.40	9.1	6	0.911	1973–1998
10	14	J LARYNGOL OTOL	0.82	0.39	7.8	5	0.561	1979–1995
11	10	EUR ARCH OTO-RHINO-L	0.78	0.39	6.7	4	0.822	1989–1998
12	17	J OTOLARYNGOL	0.78	0.42	7.2	4	0.368	1975–1998
13	6	OTOLARYNG HEAD NECK	0.75	0.40	6.9	4	1.338	1998–2002
14	13	ORL J OTO-RHINO-LARY	0.68	0.39	5.6	4	0.704	1973–2001
15	16	LARYNGO RHINO OTOL	0.57	0.38	3.9	2	0.400	1992–1999
16	15	HNO	0.57	0.41	3.9	2	0.533	1987–1998
17	11	ACTA OTO-LARYNGOL	0.44	0.84	4.7	0	0.738	1994–1997

PALEONTOLOGY

ISI Category Description Paleontology includes resources that focus on the study of life and physical conditions, such as climate and geography, of past geological periods as recorded by fossil remains.

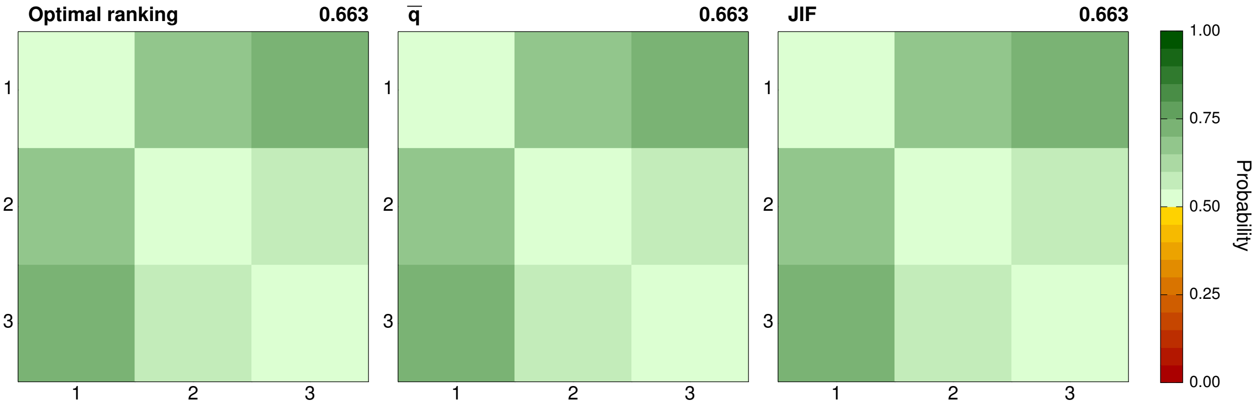

The color of each cell represents the probability of picking a paper with more citations from the higher-ranked journal. Green cells indicate adequate ranking, whereas red cells indicate that the ranking is inadequate (the probability is less than $\frac{1}{2}$). The value of M, the multi-class AUC statistic for each ranking scheme, is above each matrix.

Rank			$\mathbf{p_{ss}(q\|J)}$		n			Steady-state
**AUC**	**JIF**	**Journal abbreviation**	$\overline{\mathbf{q}}$	σ	$\overline{\mathbf{n}}$	**Q2**	**JIF**	**period**
1	1	PALAEOGEOGR PALAEOCL	1.19	0.38	20.4	13	1.822	1967–1997
2	2	REV PALAEOBOT PALYNO	0.93	0.36	10.5	7	1.170	1967–2001
3	3	J PALEONTOL	0.81	0.37	7.8	5	0.834	1954–2001

PARASITOLOGY

ISI Category Description Parasitology covers resources concerning many aspects of the study of parasites, organisms that live in or on other living organisms, deriving benefits for themselves and often causing harm to their hosts.

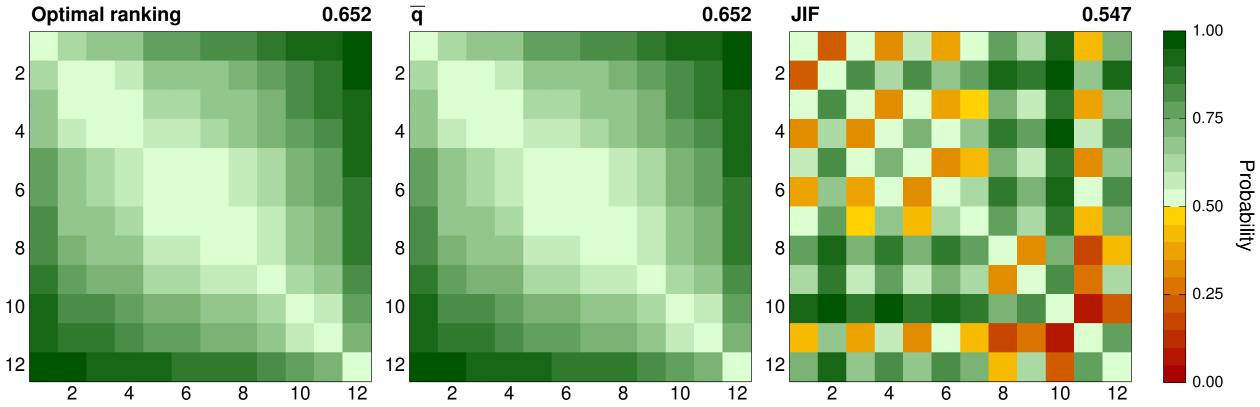

The color of each cell represents the probability of picking a paper with more citations from the higher-ranked journal. Green cells indicate adequate ranking, whereas red cells indicate that the ranking is inadequate (the probability is less than $\frac{1}{2}$). The value of M, the multi-class AUC statistic for each ranking scheme, is above each matrix.

Rank			$p_{ss}(q\|J)$		n			Steady-state
AUC	JIF	Journal abbreviation	$\overline{q}$	σ	$\overline{n}$	Q2	JIF	period
1	2	MOL BIOCHEM PARASIT	1.44	0.28	31.1	24	2.641	1991–1994
2	4	PARASITE IMMUNOL	1.29	0.33	22.5	17	2.009	1979–1995
3	6	PARASITOLOGY	1.24	0.36	21.8	15	1.786	1963–1994
4	11	EXP PARASITOL	1.20	0.35	19.6	13	1.108	1960–1997
5	1	INT J PARASITOL	1.07	0.36	14.0	10	3.337	1974–2002
6	7	J PARASITOL	1.06	0.38	15.1	9	1.300	1956–1995
7	3	ACTA TROP	1.02	0.39	12.8	9	2.211	1977–1997
8	5	VET PARASITOL	0.97	0.35	11.4	7	1.900	1975–2001
9	9	ANN TROP MED PARASIT	0.89	0.38	9.1	6	1.191	1987–2000
10	12	SYST PARASITOL	0.69	0.38	5.9	3	0.856	1980–1999
11	8	MEM I OSWALDO CRUZ	0.60	0.39	4.5	3	1.208	1971–2001
12	10	PARASITOL RES	0.21	0.39	2.4	1	1.140	1986–1987

PATHOLOGY

ISI Category Description Pathology includes resources specializing in the techniques, causes, and developmental effect of disease on living tissue. This category also considers the medical and biomedical applications of histological and cytogenetic methods, the development and use of novel techniques and diagnostic applications, and the pathologic study of specific tissues or diseases.

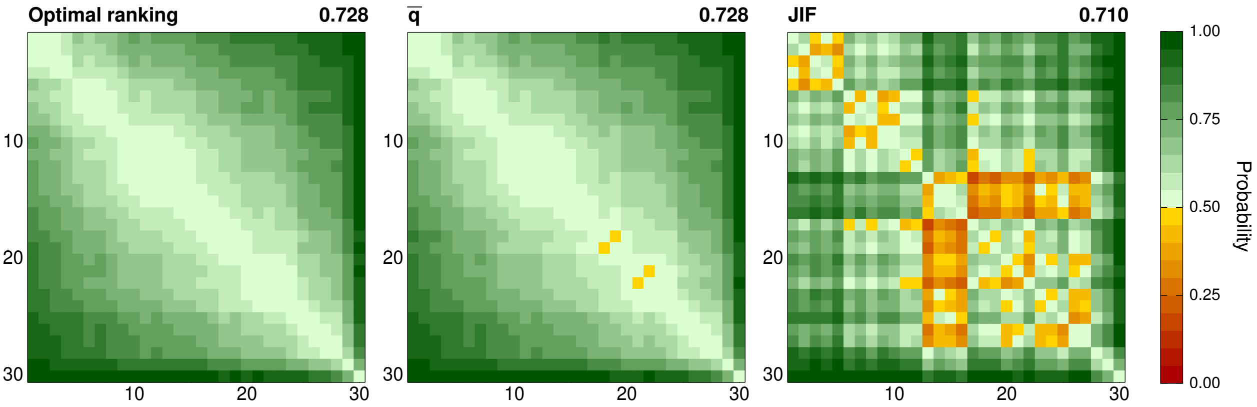

The color of each cell represents the probability of picking a paper with more citations from the higher-ranked journal. Green cells indicate adequate ranking, whereas red cells indicate that the ranking is inadequate (the probability is less than $\frac{1}{2}$). The value of M, the multi-class AUC statistic for each ranking scheme, is above each matrix.

Rank			$p_{ss}(q\|J)$		n			Steady-state
**AUC**	**JIF**	**Journal abbreviation**	$\overline{q}$	σ	$\overline{n}$	**Q2**	**JIF**	**period**
1	5	AM J SURG PATHOL	1.59	0.36	49.0	34	4.144	1976–1995
2	3	LAB INVEST	1.55	0.40	49.5	31	4.453	1963–1993
3	1	AM J PATHOL	1.53	0.43	49.9	29	5.917	1954–1992
4	4	J NEUROPATH EXP NEUR	1.47	0.44	41.1	25	4.371	1956–1999
5	2	J PATHOL	1.38	0.36	27.7	21	5.759	1995–1998
6	9	HUM PATHOL	1.36	0.43	35.4	20	2.810	1971–1992
7	7	HISTOPATHOLOGY	1.33	0.34	28.8	18	3.216	1976–1994
8	10	ACTA NEUROPATHOL	1.29	0.39	27.6	17	2.694	1965–1996
9	17	ARCH PATHOL LAB MED	1.23	0.38	23.0	14	1.605	1975–1989
10	6	MODERN PATHOL	1.22	0.37	19.1	14	3.753	1987–1999
11	8	AM J CLIN PATHOL	1.20	0.41	22.7	13	2.939	1969–1997
12	22	VET PATHOL	1.19	0.36	17.8	13	1.188	1970–1993
13	12	J CLIN PATHOL	1.16	0.42	23.6	12	2.245	1954–1994
14	11	TISSUE ANTIGENS	1.14	0.40	20.2	11	2.462	1971–1999
15	19	J ORAL PATHOL MED	1.12	0.36	16.6	11	1.530	1972–1995
16	18	J CUTAN PATHOL	1.08	0.36	13.6	10	1.582	1973–1998
17	26	ACTA CYTOL	1.06	0.38	14.6	9	0.793	1961–1995
18	21	ORAL SURG ORAL MED O	1.01	0.41	13.5	8	1.221	1970–1995
19	27	DIAGN CYTOPATHOL	1.02	0.37	11.5	8	0.786	1986–1995
20	24	PATHOL RES PRACT	0.95	0.39	11.2	7	0.892	1977–1998
21	20	EXP MOL PATHOL	0.92	0.34	10.0	7	1.377	1989–2002
22	14	TOXICOL PATHOL	0.92	0.45	11.4	6	2.092	1986–2000
23	23	J COMP PATHOL	0.91	0.37	9.4	6	0.939	1981–1999

table continues on next page . . .

...table continued from previous page

Rank			$p_{ss}(q\|J)$		n			Steady-state
**AUC**	**JIF**	**Journal abbreviation**	$\overline{q}$	σ	$\overline{n}$	**Q2**	**JIF**	**period**
24	15	APMIS	0.86	0.40	8.8	5	1.875	1987–1999
25	25	ULTRASTRUCT PATHOL	0.79	0.34	6.7	5	0.835	1995–2001
26	16	PATHOLOGY	0.71	0.36	5.9	4	1.643	1994–2002
27	13	HISTOL HISTOPATHOL	0.70	0.37	5.6	3	2.182	1985–2001
28	28	AM J FOREN MED PATH	0.65	0.44	5.1	3	0.745	1979–1999
29	29	PATHOL BIOL	0.37	0.46	3.0	1	0.667	1979–2003
30	30	ZH NEVROPATOL PSIKH	-0.20	0.39	0.4	0	0.129	1956–1995

PEDIATRICS

ISI Category Description Pediatrics covers resources on basic and clinical research in pediatrics. Numerous pediatric specialties are covered including, cardiology and respiratory systems, dentistry, dermatology, developmental behavior, gastroenterology, hematology, immunology and infectious diseases, neurology, nutrition, oncology, psychiatry, surgery, tropical medicine, urology, and nephrology. Coverage also includes perinatology, neonatology, and adolescent medicine.

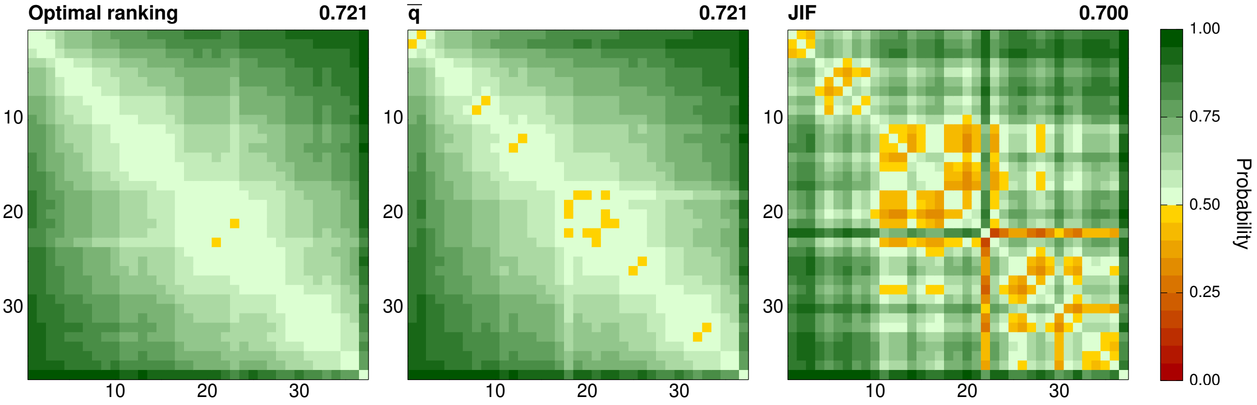

The color of each cell represents the probability of picking a paper with more citations from the higher-ranked journal. Green cells indicate adequate ranking, whereas red cells indicate that the ranking is inadequate (the probability is less than $\frac{1}{2}$). The value of M, the multi-class AUC statistic for each ranking scheme, is above each matrix.

**Rank**			$\mathbf{p_{ss}(q\|J)}$		**n**			**Steady-state**
**AUC**	**JIF**	**Journal abbreviation**	$\bar{q}$	σ	$\bar{n}$	**Q2**	**JIF**	**period**
1	3	J PEDIATR	1.52	0.38	42.3	30	3.991	1966–1995
2	2	J AM ACAD CHILD PSY	1.52	0.43	43.4	30	4.767	1987–1997
3	1	PEDIATRICS	1.45	0.45	38.9	25	5.012	1962–1993
4	7	ARCH DIS CHILD	1.34	0.39	30.1	19	2.090	1959–1988
5	4	PEDIATR INFECT DIS J	1.28	0.38	23.1	16	3.215	1990–1995
6	6	PEDIATR RES	1.25	0.37	22.5	15	2.619	1982–1996
7	9	DEV MED CHILD NEUROL	1.25	0.41	22.6	15	2.008	1976–1995
8	8	J PEDIATR GASTR NUTR	1.17	0.36	17.5	12	2.067	1993–1995
9	5	J ADOLESCENT HEALTH	1.17	0.39	16.4	12	2.710	1993–1999
10	20	J PEDIATR SURG	1.13	0.36	15.7	11	1.109	1989–1995
11	10	PEDIATR NEPHROL	1.07	0.39	14.3	10	2.007	1986–1995
12	23	PEDIATR CLIN N AM	1.06	0.38	15.4	9	1.048	1970–1997
13	14	PEDIATR NEUROL	1.06	0.34	12.7	9	1.542	1986–1997
14	18	J PEDIATR ORTHOPED	1.04	0.35	12.3	9	1.152	1980–1997
15	19	EUR J PEDIATR	1.02	0.38	12.9	8	1.137	1974–1998
16	21	PEDIATR RADIOL	0.99	0.34	11.1	8	1.076	1972–1994
17	15	J CHILD NEUROL	0.95	0.40	11.4	7	1.350	1985–2000
18	28	INT J PEDIATR OTORHI	0.91	0.37	9.2	6	0.846	1978–1995
19	13	BIOL NEONATE	0.89	0.35	9.2	6	1.681	1987–1999
20	12	EARLY HUM DEV	0.91	0.39	9.7	6	1.738	1991–2000
21	24	PEDIATR DERMATOL	0.89	0.37	8.7	6	1.014	1987–1998
22	16	ACTA PAEDIATR	0.90	0.44	9.8	6	1.297	1996–1999
23	11	PEDIATR PULM	0.93	0.62	12.0	7	1.965	1985–1997

table continues on next page . . .

...table continued from previous page

**Rank**			$\mathbf{p_{ss}(q\|J)}$		**n**			**Steady-state**
**AUC**	**JIF**	**Journal abbreviation**	$\overline{\mathbf{q}}$	σ	$\overline{\mathbf{n}}$	**Q2**	**JIF**	**period**
24	17	CHILD NERV SYST	0.86	0.38	7.6	6	1.257	1995–2000
25	32	AM J PERINAT	0.84	0.36	7.6	5	0.720	1986–1998
26	27	J PAEDIATR CHILD H	0.84	0.37	7.7	5	0.931	1989–1999
27	29	PEDIATR CARDIOL	0.79	0.40	7.5	5	0.826	1978–2000
28	31	CLIN PEDIATR	0.77	0.44	7.6	4	0.726	1961–1998
29	25	J PERINAT MED	0.70	0.42	6.5	4	1.000	1972–2001
30	36	EUR J PEDIATR SURG	0.70	0.37	5.4	4	0.579	1990–2000
31	26	ANN TROP PAEDIATR	0.67	0.37	5.2	3	0.934	1985–1998
32	35	J TROP PEDIATRICS	0.63	0.38	4.6	3	0.592	1979–2000
33	33	PEDIATR EMERG CARE	0.66	0.47	4.9	3	0.700	1991–2001
34	34	PEDIATR SURG INT	0.62	0.34	4.0	3	0.653	1999–2001
35	30	PEDIATR ANN	0.51	0.45	3.9	2	0.741	1978–1999
36	22	KLIN PADIATR	0.47	0.38	3.6	2	1.055	1971–1995
37	37	MONATSSCHR KINDERH	-0.11	0.44	0.8	0	0.161	1994–2005

PERIPHERAL VASCULAR DISEASE

ISI Category Description Peripheral Vascular Disease covers resources on arterial occlusive disease (atherosclerosis or hardening of the arteries), venous obstruction and clotting, venous incompetence/insufficiency, cerebrovascular disease, aneurysms, vasospastic disorders, and other vascular disorders. This category also covers hypertension, circulation, and stroke. Resources on the diagnosis, treatment, and prevention of heart diseases are covered in the Cardiac & Cardiovascular Systems category.

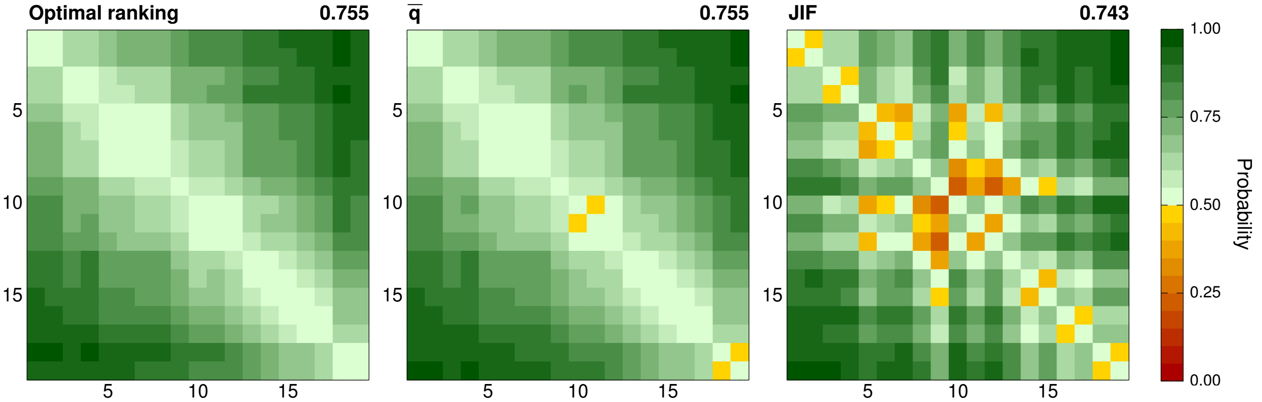

The color of each cell represents the probability of picking a paper with more citations from the higher-ranked journal. Green cells indicate adequate ranking, whereas red cells indicate that the ranking is inadequate (the probability is less than $\frac{1}{2}$). The value of M, the multi-class AUC statistic for each ranking scheme, is above each matrix.

Rank			$p_{ss}(q\|J)$		n			Steady-state
**AUC**	**JIF**	**Journal abbreviation**	$\overline{q}$	σ	$\overline{n}$	**Q2**	**JIF**	**period**
1	2	CIRC RES	1.72	0.46	75.8	47	9.854	1962–1994
2	1	CIRCULATION	1.71	0.46	75.4	45	10.940	1969–1997
3	4	STROKE	1.52	0.42	48.5	30	5.391	1976–1993
4	3	HYPERTENSION	1.49	0.34	39.1	26	6.007	1995–1999
5	7	J VASC SURG	1.40	0.40	32.7	22	3.311	1983–1995
6	10	THROMB HAEMOSTASIS	1.34	0.37	31.1	19	2.803	1980–1998
7	6	ATHEROSCLEROSIS	1.34	0.40	30.9	18	3.811	1969–1997
8	12	MICROVASC RES	1.32	0.38	30.7	18	2.477	1970–1994
9	5	J HYPERTENS	1.20	0.37	21.7	13	4.021	1996–1999
10	11	SEMIN THROMB HEMOST	1.10	0.38	17.8	10	2.733	1982–2000
11	8	AM J HYPERTENS	1.10	0.38	16.3	10	3.116	1988–2000
12	13	THROMB RES	1.04	0.39	14.1	9	2.058	1988–1995
13	15	CLIN EXP HYPERTENS	0.91	0.35	9.6	6	1.147	1982–1999
14	9	J HUM HYPERTENS	0.86	0.40	9.4	5	2.960	1986–2000
15	14	CORONARY ARTERY DIS	0.80	0.41	8.6	5	1.507	1989–2003
16	17	ANGIOLOGY	0.71	0.42	7.4	4	0.655	1960–1998
17	16	INT ANGIOL	0.64	0.42	5.2	3	0.678	1984–2002
18	19	ARCH MAL COEUR VAISS	0.43	0.39	3.0	2	0.519	1979–1997
19	18	VASA-J VASCULAR DIS	0.43	0.52	3.7	1	0.568	1972–2000

PHARMACOLOGY & PHARMACY

ISI Category Description Pharmacology & Pharmacy covers resources on the discovery and testing of bioactive substances, including animal research, clinical experience, delivery systems, and dispensing of drugs. This category also includes resources on the biochemistry, metabolism, and toxic or adverse effects of drugs.

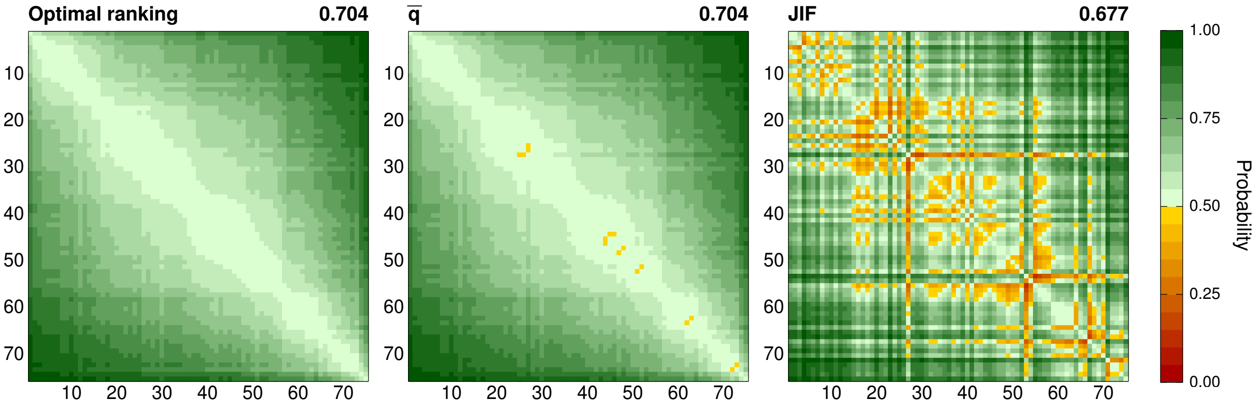

The color of each cell represents the probability of picking a paper with more citations from the higher-ranked journal. Green cells indicate adequate ranking, whereas red cells indicate that the ranking is inadequate (the probability is less than $\frac{1}{2}$). The value of M, the multi-class AUC statistic for each ranking scheme, is above each matrix.

**Rank**			$\mathbf{p_{ss}(q\|J)}$		**n**			**Steady-state**
**AUC**	**JIF**	**Journal abbreviation**	$\overline{\mathbf{q}}$	σ	$\overline{\mathbf{n}}$	**Q2**	**JIF**	**period**
1	4	MOL PHARMACOL	1.57	0.37	52.6	32	4.469	1965–1998
2	7	J PHARMACOL EXP THER	1.46	0.37	42.0	25	3.956	1984–1989
3	23	PEPTIDES	1.45	0.38	35.4	26	2.701	1979–1983
4	10	BRIT J PHARMACOL	1.41	0.40	39.3	22	3.825	1967–1991
5	1	CLIN PHARMACOL THER	1.39	0.39	33.3	22	8.066	1982–1998
6	5	ANTIMICROB AGENTS CH	1.38	0.38	32.7	21	4.153	1971–1998
7	12	PSYCHOPHARMACOLOGY	1.36	0.36	31.1	20	3.625	1975–1997
8	25	EUR J PHARMACOL	1.36	0.41	34.4	19	2.522	1969–1988
9	20	N-S ARCH PHARMACOL	1.35	0.41	33.9	19	2.779	1972–1990
10	2	TOXICOL APPL PHARM	1.34	0.36	27.8	19	4.722	1979–1995
11	9	NEUROPHARMACOLOGY	1.31	0.37	28.2	17	3.860	1969–1999
12	29	LIFE SCI	1.29	0.43	31.6	16	2.389	1963–1986
13	6	J CONTROL RELEASE	1.28	0.35	22.8	16	4.012	1997–2000
14	13	BIOCHEM PHARMACOL	1.26	0.36	24.8	15	3.581	1984–1994
15	14	ALIMENT PHARM THERAP	1.25	0.37	22.8	15	3.287	1992–1999
16	3	DRUGS	1.23	0.39	22.7	14	4.472	1991–2001
17	11	DRUG METAB DISPOS	1.19	0.37	20.1	13	3.638	1987–2002
18	30	BEHAV PHARMACOL	1.20	0.34	17.9	13	2.388	1990–1998
19	39	ALCOHOL	1.18	0.36	20.1	12	2.020	1987–1993
20	41	CHEM-BIOL INTERACT	1.15	0.35	17.8	12	1.800	1985–1995
21	8	J ANTIMICROB CHEMOTH	1.16	0.37	18.3	12	3.891	1976–2000
22	26	TOXICON	1.15	0.37	16.9	11	2.509	1966–1997
23	36	PHARMACOL BIOCHEM BE	1.14	0.35	17.0	11	2.092	1991–1997
24	55	J ANTIBIOT	1.13	0.37	18.6	11	1.262	1963–1992

table continues on next page . . .

. . . table continued from previous page

Rank			$p_{ss}(q\|J)$		n			Steady-state
AUC	JIF	Journal abbreviation	$\overline{q}$	σ	$\overline{n}$	Q2	JIF	period
25	28	J NAT PROD	1.12	0.31	15.2	11	2.418	1978–1996
26	15	INT CLIN PSYCHOPHARM	1.12	0.41	18.1	10	3.080	1985–2002
27	21	NEUROTOXICOLOGY	1.12	0.40	17.1	11	2.718	1980–1997
28	19	ANTIVIR RES	1.11	0.41	16.6	11	2.878	1980–1999
29	31	CANCER CHEMOTH PHARM	1.09	0.36	16.0	10	2.363	1988–1999
30	22	BRIT J CLIN PHARMACO	1.09	0.46	15.8	10	2.718	1994–2000
31	24	TOXICOLOGY	1.06	0.34	13.7	9	2.685	1994–2000
32	17	THER DRUG MONIT	1.06	0.36	14.0	9	3.032	1980–2000
33	35	CHIRALITY	1.06	0.34	13.7	9	2.165	1990–1997
34	45	J CARDIOVASC PHARM	1.05	0.34	14.0	9	1.625	1993–1997
35	52	CAN J PHYSIOL PHARM	1.05	0.37	14.5	9	1.380	1985–1995
36	38	EUR J CLIN PHARMACOL	1.03	0.38	13.6	9	2.029	1988–2000
37	56	CHEM PHARM BULL	1.01	0.34	12.5	8	1.262	1961–1990
38	44	J ETHNOPHARMACOL	0.99	0.33	10.8	8	1.625	1978–2001
39	34	INT J PHARM	0.99	0.35	10.9	8	2.212	1994–1998
40	46	XENOBIOTICA	0.98	0.36	12.3	7	1.613	1990–2001
41	16	J CLIN PHARMACOL	0.98	0.38	11.7	7	3.058	1986–1999
42	18	CLIN THER	0.97	0.39	13.2	7	2.893	1994–1999
43	67	Z NATURFORSCH C	0.96	0.35	11.6	7	0.720	1987–1991
44	57	PHARMACOLOGY	0.95	0.37	12.1	7	1.240	1968–1996
45	33	ANTI-CANCER DRUG	0.95	0.35	10.9	7	2.245	1990–2001
46	32	ANN PHARMACOTHER	0.96	0.38	10.8	7	2.259	1991–1998
47	37	J PHARMACEUT BIOMED	0.94	0.34	9.6	7	2.032	1984–2000
48	43	PLANTA MED	0.94	0.44	11.0	7	1.746	1977–2000
49	58	CLIN EXP HYPERTENS	0.91	0.35	9.6	6	1.147	1982–1999
50	40	PHARMACOTHERAPY	0.90	0.41	9.8	6	1.900	1986–2000
51	64	DRUG DEVELOP RES	0.89	0.47	12.0	6	0.752	1980–1999
52	47	BIOMED CHROMATOGR	0.90	0.39	8.3	6	1.611	1988–1999
53	48	J PHARM PHARMACOL	0.87	0.37	9.0	6	1.533	1995–1999
54	42	CLIN EXP PHARMACOL P	0.87	0.42	9.4	6	1.780	1976–2001
55	51	CARDIOVASC DRUG THER	0.84	0.40	9.4	5	1.396	1988–1998
56	50	CHEMOTHERAPY	0.81	0.38	8.3	5	1.511	1963–2000
57	70	ARZNEIMITTEL-FORSCH	0.76	0.42	8.7	4	0.596	1955–1996
58	59	PHYTOTHER RES	0.73	0.34	6.0	4	1.144	1998–2001
59	49	BIOMED PHARMACOTHER	0.72	0.40	6.8	4	1.526	1981–2003
60	68	J INT MED RES	0.72	0.41	6.9	4	0.665	1973–2001
61	54	INT J CLIN PHARM TH	0.71	0.40	6.9	4	1.361	1971–2000
62	61	DRUG EXP CLIN RES	0.67	0.40	6.3	3	1.030	1983–2001
63	27	PHARMACOL RES	0.67	0.40	5.7	3	2.421	1984–2004
64	60	ARCH PHARM	0.66	0.32	4.8	3	1.076	1968–2001
65	62	METHOD FIND EXP CLIN	0.62	0.43	5.2	3	0.847	1981–1999
66	63	DRUG DEV IND PHARM	0.61	0.34	4.1	3	0.821	1999–2002
67	69	PHARMAZIE	0.56	0.37	4.0	2	0.606	1974–2001

table continues on next page . . .

. . . table continued from previous page

Rank			$\mathbf{p_{ss}(q\|J)}$		n			Steady-state
**AUC**	**JIF**	**Journal abbreviation**	$\overline{\mathbf{q}}$	σ	$\overline{\mathbf{n}}$	**Q2**	**JIF**	**period**
68	74	YAKUGAKU ZASSHI	0.53	0.41	4.0	2	0.225	1968–1998
69	65	J LABELLED COMPD RAD	0.52	0.38	4.0	2	0.746	1978–2003
70	73	CURR THER RES CLIN E	0.45	0.44	3.8	2	0.319	1984–2002
71	72	THERAPIE	0.44	0.41	3.6	2	0.381	1970–2002
72	66	AM J PHARM EDUC	0.40	0.42	2.7	1	0.743	1966–2001
73	53	J CHEMOTHERAPY	0.41	0.53	3.4	1	1.374	1988–2003
74	75	PHARM IND	0.20	0.45	2.1	1	0.153	1980–2001
75	71	FOOD DRUG LAW J	0.04	0.48	1.2	0	0.397	1972–2005

PHYSICS, APPLIED

ISI Category Description Physics, Applied covers those resources dealing with the applications of condensed matter, optics, vacuum science, lasers, electronics, cryogenics, magnets and magnetism, acoustical physics, and mechanics. This category also may include resources on physics applications to other sciences, engineering, and industry.

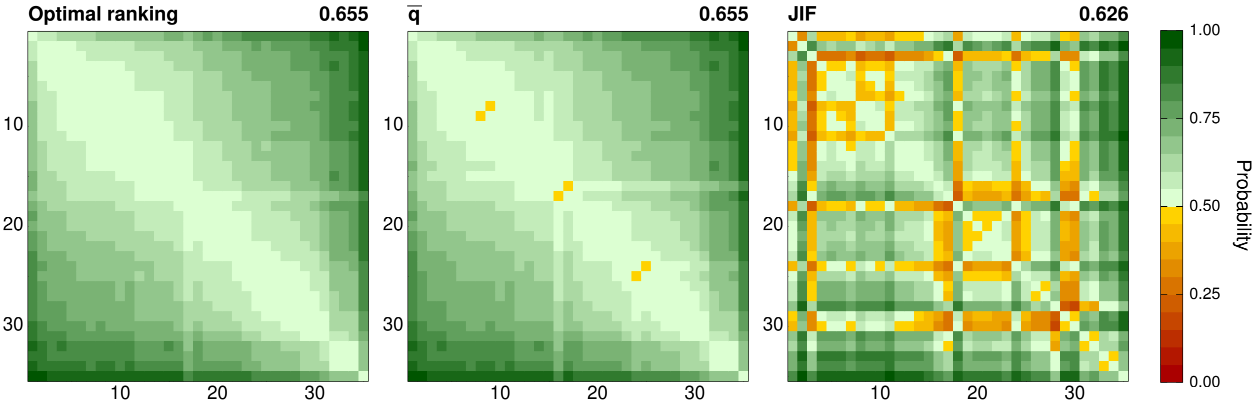

The color of each cell represents the probability of picking a paper with more citations from the higher-ranked journal. Green cells indicate adequate ranking, whereas red cells indicate that the ranking is inadequate (the probability is less than $\frac{1}{2}$). The value of M, the multi-class AUC statistic for each ranking scheme, is above each matrix.

Rank			$p_{ss}(q\|J)$		n			Steady-state
AUC	JIF	Journal abbreviation	$\overline{q}$	σ	$\overline{n}$	Q2	JIF	period
1	2	APPL PHYS LETT	1.21	0.40	24.8	13	3.977	1992–1995
2	11	J VAC SCI TECHNOL B	1.09	0.41	17.5	10	1.597	1987–1990
3	8	IEEE T ELECTRON DEV	1.05	0.44	18.1	9	2.052	1973–1994
4	18	J VAC SCI TECHNOL A	1.03	0.41	14.4	8	1.394	1992–1996
5	5	J APPL PHYS	1.01	0.41	14.8	8	2.316	1993–1999
6	9	APPL PHYS A-MATER	1.00	0.45	15.7	8	1.739	1980–1997
7	24	J LOW TEMP PHYS	0.96	0.43	15.1	7	0.978	1972–1991
8	6	IEEE J QUANTUM ELECT	0.95	0.39	12.3	7	2.262	1994–2002
9	10	THIN SOLID FILMS	0.96	0.41	12.3	7	1.666	1989–1994
10	30	PHYSICA C	0.95	0.45	14.0	7	0.792	1991–1993
11	4	IEEE PHOTONIC TECH L	0.93	0.37	10.5	7	2.353	1994–2000
12	12	SURF COAT TECH	0.91	0.45	11.2	6	1.559	1986–1995
13	7	J PHYS D APPL PHYS	0.90	0.42	11.4	6	2.077	1969–1996
14	13	REV SCI INSTRUM	0.88	0.45	12.9	5	1.541	1954–1993
15	29	INT J THERMOPHYS	0.88	0.39	9.2	6	0.793	1985–1997
16	14	J ELECTRON MATER	0.84	0.41	9.4	5	1.504	1988–1999
17	1	MRS BULL	0.86	0.67	14.1	5	5.671	1989–2002
18	15	SUPERCOND SCI TECH	0.78	0.39	7.7	5	1.440	1992–1998
19	25	IEEE T MAGN	0.74	0.45	8.4	4	0.938	1965–1991
20	21	JPN J APPL PHYS	0.74	0.47	8.7	4	1.222	1974–1982
21	20	MATER LETT	0.71	0.40	6.7	4	1.353	1984–2000
22	22	PHYS STATUS SOLIDI A	0.69	0.41	6.4	3	1.221	1984–2000
23	19	PHILOS MAG	0.66	0.39	6.0	3	1.354	1978–2003
24	23	SOLID STATE ELECTRON	0.62	0.42	5.2	3	1.159	1993–2000

table continues on next page . . .

. . . table continued from previous page

Rank			$p_{ss}(q\|J)$		n			Steady-state
**AUC**	**JIF**	**Journal abbreviation**	$\overline{q}$	σ	$\overline{n}$	**Q2**	**JIF**	**period**
25	16	APPL SURF SCI	0.62	0.49	5.7	3	1.436	1996–1999
26	27	VACUUM	0.58	0.43	5.2	2	0.834	1965–2001
27	26	CRYOGENICS	0.56	0.43	5.3	2	0.927	1977–1999
28	3	LASER PART BEAMS	0.55	0.41	4.6	2	3.958	1986–2005
29	32	INT J MOD PHYS B	0.50	0.49	5.8	2	0.437	1989–1998
30	17	MICROELECTRON ENG	0.48	0.44	3.9	2	1.398	1992–2003
31	31	J ELECTROMAGNET WAVE	0.42	0.46	3.1	1	0.524	1995–1999
32	28	MICROELECTRON RELIAB	0.27	0.44	2.2	1	0.815	1965–2004
33	34	SOLID STATE TECHNOL	0.21	0.53	2.7	1	0.293	1992–1999
34	33	INT J INFRARED MILLI	0.17	0.46	1.7	1	0.326	1994–2002
35	35	HIGH TEMP+	-0.23	0.44	0.5	0	0.198	1971–2005

PHYSICS, ATOMIC, MOLECULAR & CHEMICAL

ISI Category Description Physics, Atomic, Molecular & Chemical includes resources concerned with the physics of atoms and molecules. Topics covered in this category include the structure of atoms and molecules, atomic and molecular interactions with radiation, magnetic resonances and relaxation, Mossbauer effect, and atomic and molecular collision processes and interactions.

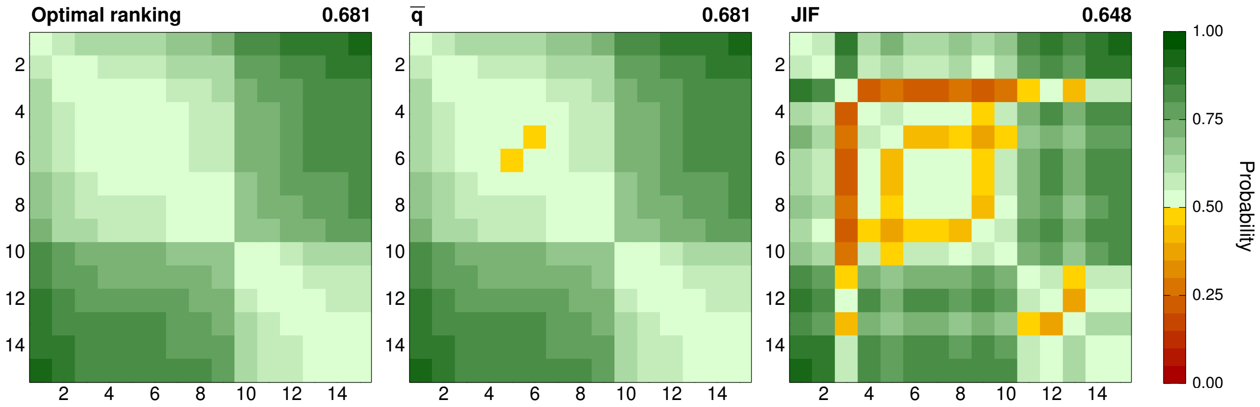

The color of each cell represents the probability of picking a paper with more citations from the higher-ranked journal. Green cells indicate adequate ranking, whereas red cells indicate that the ranking is inadequate (the probability is less than $\frac{1}{2}$). The value of M, the multi-class AUC statistic for each ranking scheme, is above each matrix.

Rank			$p_{ss}(q\|J)$		n			Steady-state
**AUC**	**JIF**	**Journal abbreviation**	$\bar{q}$	σ	$\bar{n}$	**Q2**	**JIF**	**period**
1	1	J CHEM PHYS	1.43	0.39	38.4	23	3.166	1989–1991
2	2	PHYS REV A	1.32	0.42	33.2	18	3.047	1981–1986
3	9	MOL PHYS	1.26	0.40	28.7	15	1.690	1980–1987
4	4	CHEM PHYS LETT	1.21	0.39	23.1	14	2.462	1972–1993
5	6	J MAGN RESON	1.19	0.40	30.0	13	2.076	1970–1992
6	7	J PHYS B-AT MOL OPT	1.20	0.37	20.4	13	2.024	1981–1986
7	8	CHEM PHYS	1.15	0.38	17.7	12	1.984	1986–1995
8	10	J MOL SPECTROSC	1.12	0.35	16.2	11	1.260	1987–1993
9	5	INT J MASS SPECTROM	1.08	0.42	18.0	10	2.337	1974–1994
10	13	NUCL INSTRUM METH B	0.82	0.39	8.7	5	0.946	1988–1997
11	11	INT J QUANTUM CHEM	0.77	0.52	8.9	4	1.182	1992–1998
12	3	INT J HYDROGEN ENERG	0.70	0.42	6.7	4	2.612	1975–2004
13	12	J MOL LIQ	0.65	0.42	5.6	3	1.106	1982–2002
14	14	RADIAT PHYS CHEM	0.60	0.44	5.1	2	0.868	1992–2000
15	15	HYPERFINE INTERACT	0.56	0.43	4.7	2	0.267	1981–2000

PHYSICS, CONDENSED MATTER

ISI Category Description Physics, Condensed Matter covers resources that deal with the study of the structure and the thermal, mechanical, electrical, magnetic, and optical properties of condensed matter. Topics covered in this category include superconductivity, surfaces, interfaces, thin films, dielectrics, ferroelectrics, and semiconductors. This category also includes resources from the former category of Solid State Physics as well as resources on condensed fluids.

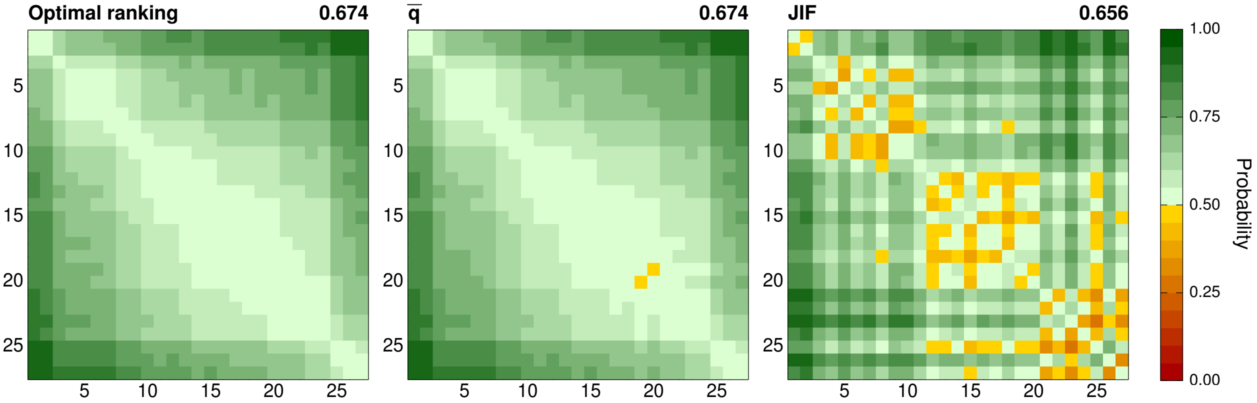

The color of each cell represents the probability of picking a paper with more citations from the higher-ranked journal. Green cells indicate adequate ranking, whereas red cells indicate that the ranking is inadequate (the probability is less than $\frac{1}{2}$). The value of M, the multi-class AUC statistic for each ranking scheme, is above each matrix.

Rank			$p_{ss}(q\|J)$		n			Steady-state
AUC	JIF	Journal abbreviation	$\overline{q}$	σ	$\overline{n}$	Q2	JIF	period
1	2	PHYS REV B	1.32	0.43	37.1	18	3.107	1984–1990
2	1	J MECH PHYS SOLIDS	1.28	0.44	42.4	16	3.609	1954–1997
3	5	SURF SCI	1.12	0.47	18.5	11	1.880	1989–1995
4	3	SOLID STATE IONICS	1.02	0.42	13.3	8	2.190	1998–2000
5	9	SOLID STATE COMMUN	1.00	0.41	15.5	8	1.556	1982–1988
6	10	PHIL MAG LETT	0.99	0.41	13.5	7	1.539	1986–1996
7	7	THIN SOLID FILMS	0.96	0.41	12.3	7	1.666	1989–1994
8	4	J PHYS-CONDENS MAT	0.87	0.40	10.0	6	2.038	1992–1999
9	6	SYNTHETIC MET	0.86	0.43	10.7	6	1.685	1994–1997
10	11	SUPERCOND SCI TECH	0.78	0.39	7.7	5	1.440	1992–1998
11	18	J PHYS CHEM SOLIDS	0.76	0.41	7.5	4	1.164	1996–2000
12	8	SEMICOND SCI TECH	0.75	0.39	7.1	4	1.586	1991–1997
13	14	MAT SCI ENG B-SOLID	0.71	0.40	6.5	4	1.331	1989–2000
14	25	FERROELECTRICS	0.70	0.50	9.0	3	0.389	1972–1993
15	16	PHYS STATUS SOLIDI A	0.69	0.41	6.4	3	1.221	1984–2000
16	13	PHILOS MAG	0.66	0.39	6.0	3	1.354	1978–2003
17	20	PHYS STATUS SOLIDI B	0.63	0.41	5.7	3	0.967	1985–1999
18	17	J MAGN MAGN MATER	0.64	0.48	6.9	3	1.212	1997–2000
19	19	SOLID STATE ELECTRON	0.62	0.42	5.2	3	1.159	1993–2000
20	12	APPL SURF SCI	0.62	0.49	5.7	3	1.436	1996–1999
21	27	HYPERFINE INTERACT	0.56	0.43	4.7	2	0.267	1981–2000
22	15	SUPERLATTICE MICROST	0.54	0.44	4.8	2	1.259	1989–2001
23	22	PHASE TRANSIT	0.51	0.46	4.8	2	0.830	1986–2005

table continues on next page . . .

. . . table continued from previous page

Rank			$\mathbf{p_{ss}(q\|J)}$		$\mathbf{n}$			Steady-state
**AUC**	**JIF**	**Journal abbreviation**	$\overline{\mathbf{q}}$	σ	$\overline{\mathbf{n}}$	**Q2**	**JIF**	**period**
24	24	INT J MOD PHYS B	0.50	0.49	5.8	2	0.437	1989–1998
25	21	PHYSICA B	0.31	0.48	2.6	1	0.872	1998–2000
26	26	SOLID STATE TECHNOL	0.21	0.53	2.7	1	0.293	1992–1999
27	23	RADIAT EFF DEFECT S	0.19	0.41	1.6	1	0.497	1997–2005

PHYSICS, FLUIDS & PLASMAS

ISI Category Description Physics, Fluids & Plasmas covers resources on the kinetic and transport theory of fluids, the physical properties of gases, and the physics of plasmas and electric discharges. This category may include resources on nuclear fusion.

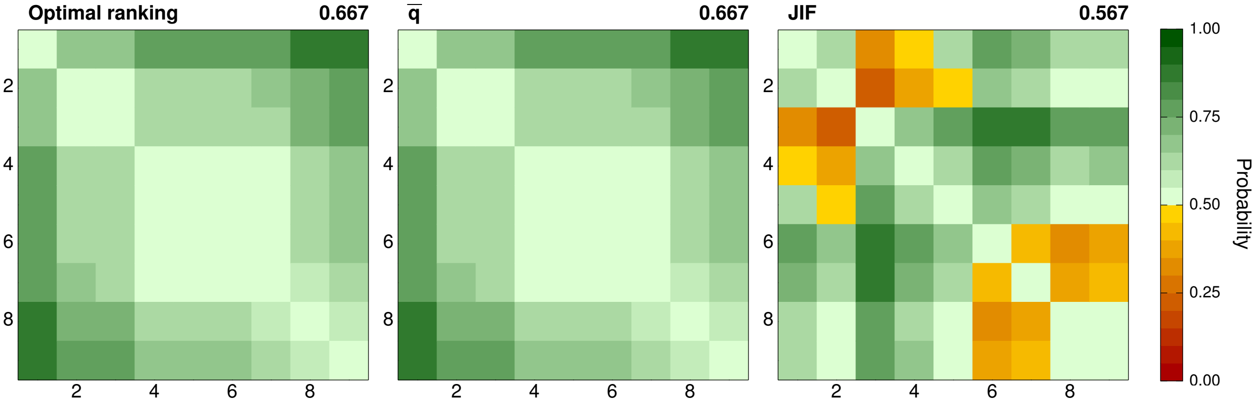

The color of each cell represents the probability of picking a paper with more citations from the higher-ranked journal. Green cells indicate adequate ranking, whereas red cells indicate that the ranking is inadequate (the probability is less than $\frac{1}{2}$). The value of M, the multi-class AUC statistic for each ranking scheme, is above each matrix.

Rank			$p_{ss}(q\|J)$		n			Steady-state
AUC	JIF	Journal abbreviation	$\overline{q}$	σ	$\overline{n}$	Q2	JIF	period
1	3	J FLUID MECH	1.42	0.45	43.0	23	2.022	1956–1991
2	4	PHYS FLUIDS	1.10	0.44	20.4	10	1.697	1966–1996
3	1	NUCL FUSION	1.06	0.46	17.0	9	2.839	1959–2001
4	5	IEEE T PLASMA SCI	0.86	0.43	10.9	5	1.144	1978–1998
5	2	PLASMA PHYS CONTR F	0.85	0.43	9.9	5	2.820	1972–2002
6	8	INT J NUMER METH FL	0.85	0.43	11.5	5	0.870	1980–1998
7	9	J PLASMA PHYS	0.80	0.45	10.8	4	0.748	1967–1992
8	7	EXP THERM FLUID SCI	0.65	0.36	5.9	3	0.894	1987–2000
9	6	CONTRIB PLASM PHYS	0.55	0.45	4.5	2	1.113	1984–2001

PHYSICS, MATHEMATICAL

ISI Category Description Physics, Mathematical includes resources that focus on mathematical methods in physics. It includes resources on logic, set theory, algebra, group theory, function theory, analysis, geometry, topology, and probability theory that have applications in physics.

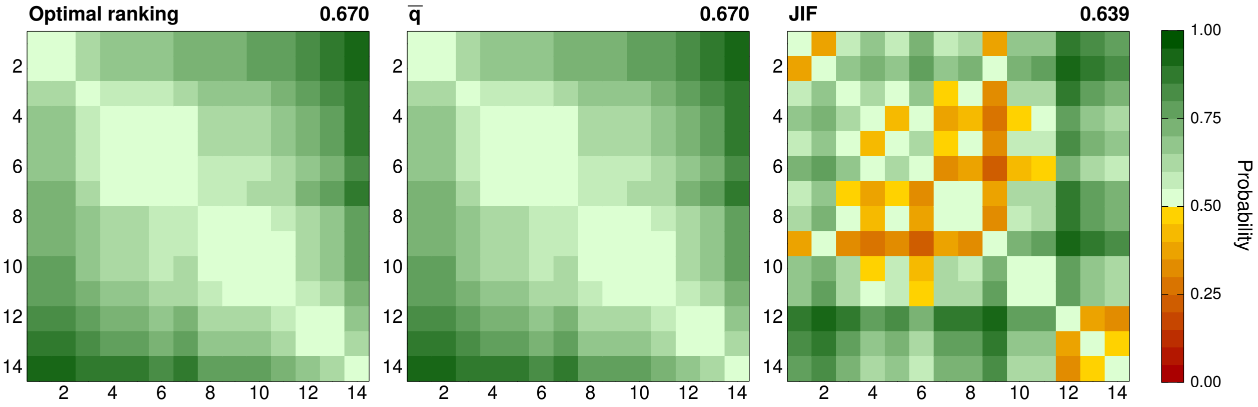

The color of each cell represents the probability of picking a paper with more citations from the higher-ranked journal. Green cells indicate adequate ranking, whereas red cells indicate that the ranking is inadequate (the probability is less than $\frac{1}{2}$). The value of M, the multi-class AUC statistic for each ranking scheme, is above each matrix.

Rank			$p_{ss}(q\|J)$		n			Steady-state
**AUC**	**JIF**	**Journal abbreviation**	$\overline{q}$	σ	$\overline{n}$	**Q2**	**JIF**	**period**
1	2	COMMUN MATH PHYS	1.26	0.44	35.7	16	2.077	1968–1993
2	9	NONLINEARITY	1.23	0.37	25.2	16	1.288	1989–1991
3	1	J COMPUT PHYS	1.08	0.41	19.6	10	2.328	1994–1999
4	7	J STAT PHYS	0.99	0.45	16.8	7	1.437	1985–1996
5	3	PHYSICA D	0.98	0.39	12.6	8	1.674	1996–2000
6	5	J PHYS A-MATH GEN	0.91	0.46	14.7	6	1.577	1971–1993
7	8	INVERSE PROBL	0.91	0.40	11.0	6	1.319	1985–1999
8	10	J MATH PHYS	0.79	0.43	9.1	4	1.018	1985–1996
9	4	COMPUT PHYS COMMUN	0.75	0.52	17.4	4	1.595	1970–2000
10	11	LETT MATH PHYS	0.72	0.43	9.4	4	0.986	1974–1999
11	6	MOD PHYS LETT A	0.67	0.48	10.8	3	1.564	1987–1992
12	14	INT J MOD PHYS B	0.50	0.49	5.8	2	0.437	1989–1998
13	13	J ELECTROMAGNET WAVE	0.42	0.46	3.1	1	0.524	1995–1999
14	12	THEOR MATH PHYS+	0.10	0.50	2.0	0	0.626	1974–2002

PHYSICS, MULTIDISCIPLINARY

ISI Category Description Physics, Multidisciplinary covers resources having a general or interdisciplinary approach to physics. This category also includes theoretical and experimental physics as well as special topics that have relevance to many areas of physics.

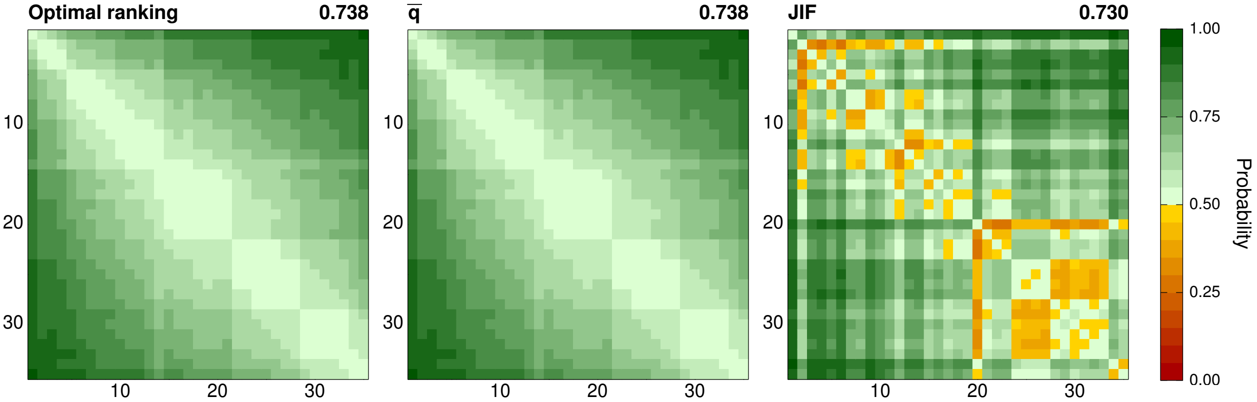

The color of each cell represents the probability of picking a paper with more citations from the higher-ranked journal. Green cells indicate adequate ranking, whereas red cells indicate that the ranking is inadequate (the probability is less than $\frac{1}{2}$). The value of M, the multi-class AUC statistic for each ranking scheme, is above each matrix.

**Rank**			$\mathbf{p_{ss}(q\|J)}$		**n**			**Steady-state**
**AUC**	**JIF**	**Journal abbreviation**	$\overline{\mathbf{q}}$	σ	$\overline{\mathbf{n}}$	**Q2**	**JIF**	**period**
1	1	PHYS REV LETT	1.40	0.48	44.3	22	7.072	1969–2006
2	4	ANN PHYS-NEW YORK	1.23	0.51	43.1	14	3.019	1960–1991
3	6	EUROPHYS LETT	1.16	0.43	22.9	12	2.229	1986–1994
4	3	PHYS LETT B	1.11	0.45	22.3	10	5.043	1967–2006
5	9	PHYSICA D	0.98	0.39	12.6	8	1.674	1996–2000
6	5	CLASSICAL QUANT GRAV	0.92	0.41	12.0	6	2.773	1983–1998
7	10	J PHYS A-MATH GEN	0.91	0.46	14.7	6	1.577	1971–1993
8	14	PHYSICA A	0.86	0.44	11.3	5	1.311	1975–1997
9	13	PHYS LETT A	0.83	0.43	10.7	5	1.468	1966–1996
10	7	J PHYS SOC JPN	0.78	0.42	9.3	4	1.926	1992–1999
11	8	PROG THEOR PHYS	0.76	0.43	8.6	4	1.712	1988–2001
12	11	GEN RELAT GRAVIT	0.72	0.43	8.1	4	1.531	1974–1998
13	16	PHYS SCRIPTA	0.72	0.47	7.9	4	1.161	1990–1993
14	2	PHYS TODAY	0.70	0.64	16.4	3	5.839	1968–2004
15	18	AM J PHYS	0.58	0.42	6.3	2	0.919	1960–1999
16	19	Z NATURFORSCH A	0.58	0.43	4.7	2	0.904	1991–2001
17	15	JETP LETT+	0.57	0.45	6.6	2	1.251	1971–2001
18	12	CHINESE PHYS	0.52	0.45	4.1	2	1.497	1992–2003
19	23	CAN J PHYS	0.51	0.46	4.7	2	0.756	1987–2002
20	22	FOUND PHYS	0.51	0.42	3.9	2	0.854	1995–2003
21	17	CHINESE PHYS LETT	0.48	0.35	3.2	2	1.135	1997–2003
22	29	INT J THEOR PHYS	0.41	0.46	3.9	1	0.389	1974–2000
23	21	ACTA PHYS POL B	0.39	0.45	3.9	1	0.882	1974–1999
24	32	NUOVO CIMENTO B	0.36	0.44	3.1	1	0.351	1986–1998

table continues on next page . . .

. . . table continued from previous page

Rank			$\mathbf{p_{ss}(q\|J)}$		n			Steady-state
**AUC**	**JIF**	**Journal abbreviation**	$\overline{\mathbf{q}}$	σ	$\overline{\mathbf{n}}$	**Q2**	**JIF**	**period**
25	28	PRAMANA-J PHYS	0.34	0.41	2.5	1	0.417	1989–1996
26	31	ACTA PHYS POL A	0.31	0.42	2.3	1	0.371	1982–1999
27	33	J PHYS IV	0.28	0.45	2.4	1	0.315	1993–2000
28	30	INDIAN J PURE AP PHY	0.27	0.44	2.1	1	0.380	1978–2002
29	24	COMMUN THEOR PHYS	0.15	0.49	1.8	0	0.726	1984–2004
30	26	PROG THEOR PHYS SUPP	0.12	0.56	2.5	0	0.568	1985–2001
31	25	THEOR MATH PHYS+	0.10	0.50	2.0	0	0.626	1974–2002
32	27	CZECH J PHYS	0.08	0.47	1.4	0	0.568	1990–2005
33	35	CHINESE J PHYS	0.05	0.42	1.0	0	0.238	2001–2005
34	20	PHYS WORLD	-0.13	0.65	2.0	0	0.886	1992–2005
35	34	REV MEX FIS	-0.15	0.45	0.6	0	0.265	1996–2006

PHYSICS, NUCLEAR

ISI Category Description Physics, Nuclear includes resources on the study of nuclear structure, decay, radioactivity, reactions, and scattering. Resources in this category focus on low-energy physics. High-energy physics is covered in the PHYSICS, PARTICLES & FIELDS category.

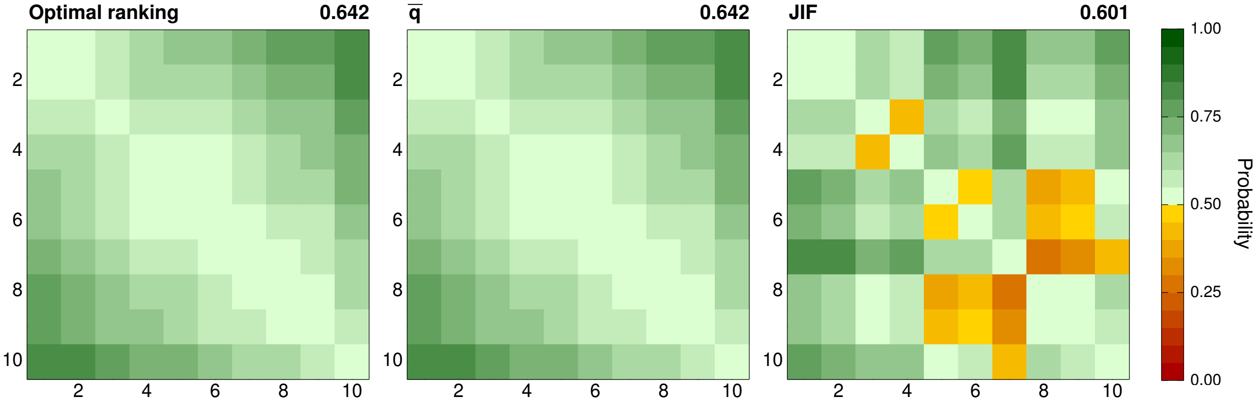

The color of each cell represents the probability of picking a paper with more citations from the higher-ranked journal. Green cells indicate adequate ranking, whereas red cells indicate that the ranking is inadequate (the probability is less than $\frac{1}{2}$). The value of M, the multi-class AUC statistic for each ranking scheme, is above each matrix.

Rank			$p_{ss}(q\|J)$		n			Steady-state
**AUC**	**JIF**	**Journal abbreviation**	$\overline{q}$	σ	$\overline{n}$	**Q2**	**JIF**	**period**
1	1	PHYS REV C	1.10	0.38	17.6	10	3.327	1987–1995
2	2	NUCL FUSION	1.06	0.46	17.0	9	2.839	1959–2001
3	4	NUCL PHYS A	0.96	0.47	14.0	7	2.155	1990–1995
4	3	PLASMA PHYS CONTR F	0.85	0.43	9.9	5	2.820	1972–2002
5	8	NUCL INSTRUM METH B	0.82	0.39	8.7	5	0.946	1988–1997
6	9	INT J MOD PHYS A	0.77	0.50	11.5	4	0.914	1986–1994
7	6	MOD PHYS LETT A	0.67	0.48	10.8	3	1.564	1987–1992
8	5	J PHYS G NUCL PARTIC	0.59	0.40	4.8	3	1.781	1995–2004
9	10	HYPERFINE INTERACT	0.56	0.43	4.7	2	0.267	1981–2000
10	7	ENERG CONVERS MANAGE	0.40	0.45	3.1	1	1.325	1987–2006

PHYSICS, PARTICLES & FIELDS

ISI Category Description Physics, Particles & Fields includes resources on the study of the structure and properties of elementary particles and resonances and their interactions. Resources in this category focus on high-energy physics. Low-energy physics is covered in the PHYSICS, NUCLEAR category.

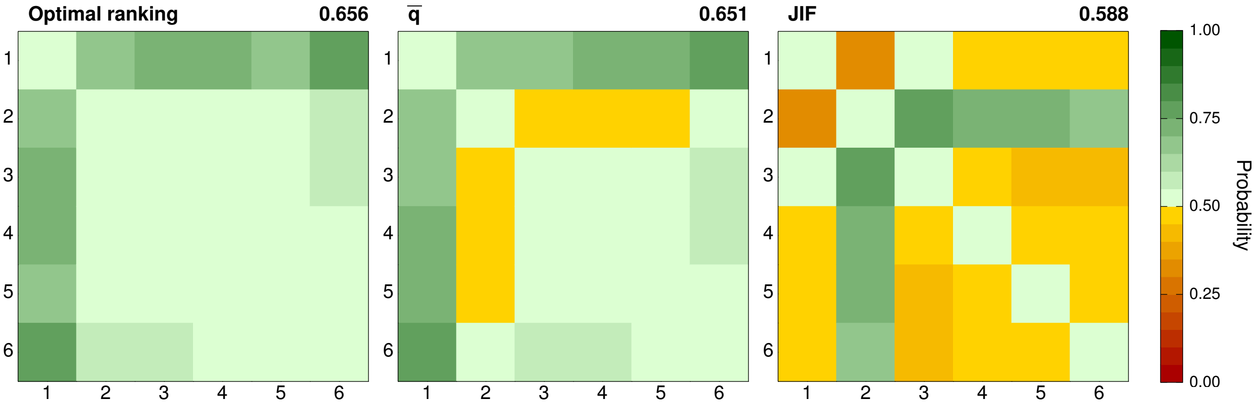

The color of each cell represents the probability of picking a paper with more citations from the higher-ranked journal. Green cells indicate adequate ranking, whereas red cells indicate that the ranking is inadequate (the probability is less than $\frac{1}{2}$). The value of M, the multi-class AUC statistic for each ranking scheme, is above each matrix.

**Rank**			$\mathbf{p_{ss}(q\|J)}$		**n**			**Steady-state**
**AUC**	**JIF**	**Journal abbreviation**	$\overline{\mathbf{q}}$	σ	$\overline{\mathbf{n}}$	**Q2**	**JIF**	**period**
1	2	PHYS REV D	1.17	0.47	27.1	12	4.896	1982–1995
2	6	INT J MOD PHYS A	0.77	0.50	11.5	4	0.914	1986–1994
3	5	NUCL INSTRUM METH A	0.73	0.44	8.3	4	1.185	1989–1994
4	4	MOD PHYS LETT A	0.67	0.48	10.8	3	1.564	1987–1992
5	1	NUCL PHYS B	0.79	0.99	19.9	3	5.199	1993–1995
6	3	J PHYS G NUCL PARTIC	0.59	0.40	4.8	3	1.781	1995–2004

PHYSIOLOGY

ISI Category Description Physiology includes resources concerned with the normal and pathologic functioning of living cells, tissues, and organisms. Topics include comparative physiology, molecular biochemistry of cell function, applied physiology, and pharmacological intervention in pathophysiological processes.

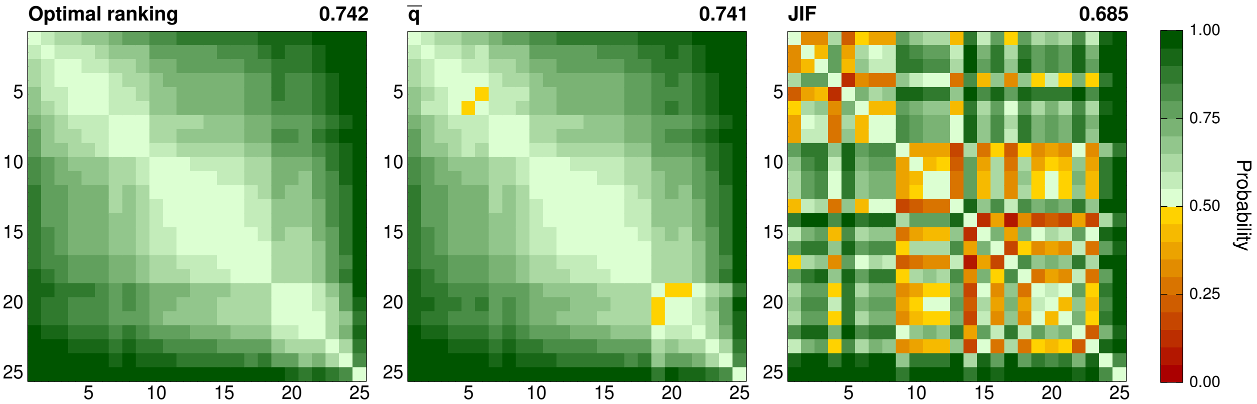

The color of each cell represents the probability of picking a paper with more citations from the higher-ranked journal. Green cells indicate adequate ranking, whereas red cells indicate that the ranking is inadequate (the probability is less than $\frac{1}{2}$). The value of M, the multi-class AUC statistic for each ranking scheme, is above each matrix.

Rank			$p_{ss}(q\|J)$		n			Steady-state
**AUC**	**JIF**	**Journal abbreviation**	$\overline{q}$	σ	$\overline{n}$	**Q2**	**JIF**	**period**
1	5	J NEUROPHYSIOL	1.74	0.36	70.6	50	3.652	1981–1989
2	3	J PHYSIOL-LONDON	1.63	0.39	60.3	37	4.407	1978–1992
3	2	J GEN PHYSIOL	1.55	0.33	44.2	33	4.685	1989–1997
4	7	J APPL PHYSIOL	1.53	0.41	47.6	30	3.178	1975–1980
5	8	PSYCHOPHYSIOLOGY	1.47	0.36	41.8	27	3.159	1985–1995
6	13	J MEMBRANE BIOL	1.47	0.38	41.2	27	2.112	1973–1988
7	6	J CELL PHYSIOL	1.39	0.41	35.4	21	3.638	1965–1993
8	17	J COMP PHYSIOL A	1.37	0.31	26.4	21	1.751	1984–1989
9	1	PFLUG ARCH EUR J PHY	1.32	0.40	34.8	18	4.807	1969–1990
10	15	J INSECT PHYSIOL	1.24	0.34	20.2	15	2.019	1970–1995
11	23	FISH PHYSIOL BIOCHEM	1.20	0.36	19.1	14	0.558	1986–1995
12	19	ARCH INSECT BIOCHEM	1.18	0.35	16.9	13	1.474	1982–1994
13	21	PESTIC BIOCHEM PHYS	1.16	0.38	18.2	12	1.189	1975–1993
14	4	J PINEAL RES	1.14	0.36	17.3	12	4.228	1983–2002
15	11	ACTA PHYSIOL SCAND	1.11	0.39	16.7	11	2.230	1987–1994
16	12	CRYOBIOLOGY	1.11	0.39	17.1	10	2.162	1963–1997
17	20	CAN J PHYSIOL PHARM	1.05	0.37	14.5	9	1.380	1985–1995
18	10	EXP PHYSIOL	1.02	0.34	13.0	8	2.339	1989–1997
19	18	COMP BIOCHEM PHYS A	0.87	0.36	8.6	6	1.553	1986–2002
20	16	CLIN EXP PHARMACOL P	0.87	0.42	9.4	6	1.780	1976–2001
21	9	REGUL PEPTIDES	0.88	0.57	10.4	5	2.442	1994–1998
22	22	JPN J PHYSIOL	0.78	0.37	7.1	5	1.081	1984–1998
23	14	PHYSIOL RES	0.64	0.38	4.3	3	2.093	1990–2003
24	24	ZH VYSSH NERV DEYAT+	0.38	0.42	2.6	1	0.379	1961–1998
25	25	J EVOL BIOCHEM PHYS+	-0.76	0.61	0.2	0	0.206	1983–2003

PLANNING & DEVELOPMENT

ISI Category Description Planning & Development is concerned with resources on the economics and social development of both underdeveloped and industrialized areas. The resources in this category focus on subjects such as economic forecasting, development studies, policy-making strategies, theories of planning, and the growth of the third world.

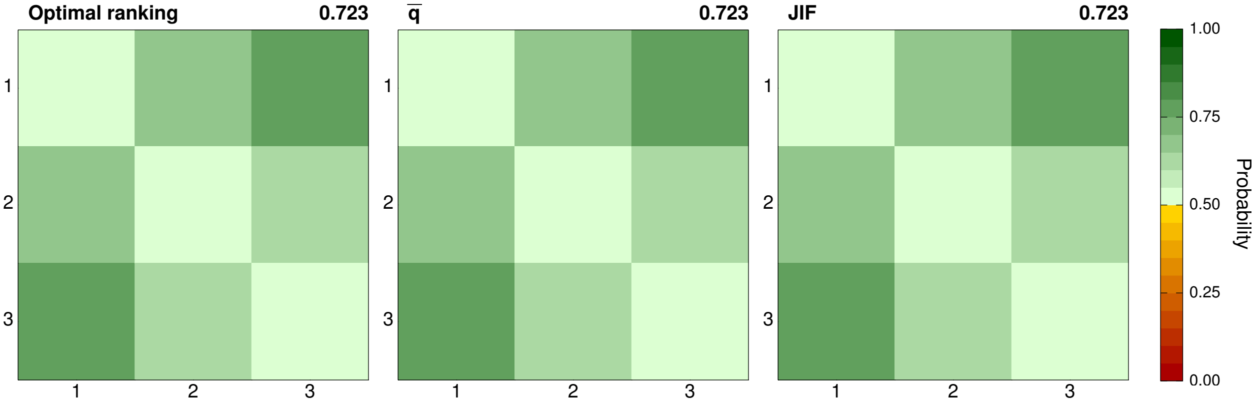

The color of each cell represents the probability of picking a paper with more citations from the higher-ranked journal. Green cells indicate adequate ranking, whereas red cells indicate that the ranking is inadequate (the probability is less than $\frac{1}{2}$). The value of M, the multi-class AUC statistic for each ranking scheme, is above each matrix.

**Rank**			$\mathbf{p_{ss}(q\|J)}$		$\mathbf{n}$			**Steady-state**
**AUC**	**JIF**	**Journal abbreviation**	$\overline{\mathbf{q}}$	σ	$\overline{\mathbf{n}}$	**Q2**	**JIF**	**period**
1	1	WORLD DEV	0.80	0.39	8.2	5	1.298	1981–2001
2	2	LONG RANGE PLANN	0.52	0.40	4.0	2	0.982	1989–2001
3	3	FUTURES	0.23	0.44	2.4	1	0.738	1967–2004

PLANT SCIENCES

ISI Category Description Plant Sciences covers resources concerning many aspects of the study of plants including systematic, biochemical, agricultural, and pharmaceutical topics. This category includes materials on higher and lower plants, terrestrial and aquatic plants, plant cells, entire plants, and plant assemblages.

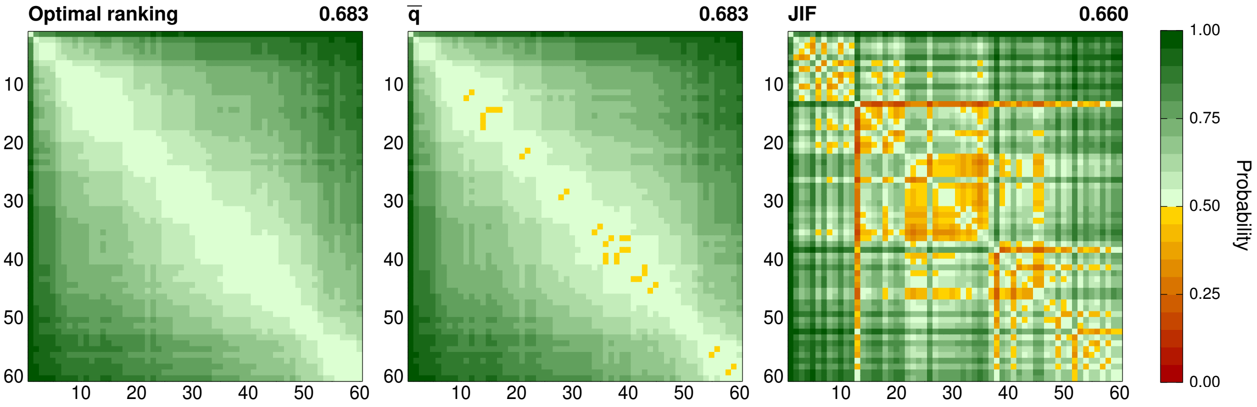

The color of each cell represents the probability of picking a paper with more citations from the higher-ranked journal. Green cells indicate adequate ranking, whereas red cells indicate that the ranking is inadequate (the probability is less than $\frac{1}{2}$). The value of M, the multi-class AUC statistic for each ranking scheme, is above each matrix.

Rank			$p_{ss}(q\|J)$		n			Steady-state
AUC	JIF	Journal abbreviation	$\overline{q}$	σ	$\overline{n}$	Q2	JIF	period
1	1	PLANT CELL	1.87	0.34	92.4	72	9.868	1988–1995
2	5	MOL PLANT MICROBE IN	1.52	0.31	41.0	34	3.936	1988–1993
3	2	PLANT PHYSIOL	1.48	0.35	38.0	27	6.125	1954–1995
4	7	PLANT MOL BIOL	1.47	0.33	34.9	26	3.577	1989–1994
5	10	PLANTA	1.43	0.33	34.0	24	2.963	1970–1995
6	4	PLANT CELL ENVIRON	1.35	0.36	28.5	19	4.135	1979–2000
7	12	J PHYCOL	1.30	0.35	26.0	17	2.580	1964–1997
8	3	NEW PHYTOL	1.28	0.38	25.7	16	4.245	1963–1995
9	17	J VEG SCI	1.28	0.36	22.4	16	2.382	1989–1999
10	20	PHOTOSYNTH RES	1.26	0.38	26.5	15	2.193	1990–1993
11	9	AM J BOT	1.25	0.36	22.9	15	2.969	1955–1998
12	11	THEOR APPL GENET	1.25	0.40	23.9	15	2.715	1985–1999
13	35	PROTOPLASMA	1.22	0.35	19.8	14	1.333	1971–1992
14	16	PHYTOCHEMISTRY	1.21	0.30	18.7	14	2.417	1981–1986
15	6	J EXP BOT	1.21	0.32	18.3	13	3.630	1991–1998
16	21	PHYSIOL PLANTARUM	1.21	0.36	23.8	13	2.169	1954–1997
17	26	PLANT CELL REP	1.21	0.35	19.1	13	1.727	1983–1993
18	8	PLANT CELL PHYSIOL	1.18	0.36	18.7	13	3.324	1958–1998
19	19	PHYTOPATHOLOGY	1.16	0.36	18.6	12	2.195	1956–1991
20	36	PHYSIOL MOL PLANT P	1.13	0.34	14.7	11	1.288	1992–2000
21	15	J NAT PROD	1.12	0.31	15.2	11	2.418	1978–1996
22	32	PLANT SOIL	1.12	0.40	17.1	11	1.495	1988–1991
23	34	AQUAT BOT	1.11	0.30	13.8	11	1.338	1994–1999
24	14	ANN BOT-LONDON	1.10	0.37	15.9	10	2.448	1970–1996

table continues on next page . . .

...table continued from previous page

Rank			$p_{ss}(q\|J)$		n			Steady-state
**AUC**	**JIF**	**Journal abbreviation**	$\overline{q}$	σ	$\overline{n}$	**Q2**	**JIF**	**period**
25	45	PLANT CELL TISS ORG	1.06	0.35	13.1	9	0.951	1985–1994
26	46	AUST J BOT	1.05	0.32	12.6	9	0.940	1992–1998
27	31	J PLANT PHYSIOL	1.04	0.37	13.2	9	1.521	1983–1996
28	33	WEED SCI	1.03	0.37	13.3	9	1.476	1967–1999
29	18	PLANT PATHOL	1.04	0.37	12.8	9	2.198	1981–1993
30	39	CAN J BOT	1.02	0.37	13.1	8	1.193	1988–1997
31	28	PLANT SCI	1.00	0.32	11.0	8	1.631	1993–2001
32	29	J ETHNOPHARMACOL	0.99	0.33	10.8	8	1.625	1978–2001
33	30	INT J PLANT SCI	0.98	0.32	10.3	7	1.622	1991–2001
34	22	PLANT PHYSIOL BIOCH	0.96	0.34	10.3	7	1.847	1990–2000
35	42	BOT MAR	0.97	0.34	9.6	7	1.009	1992–1996
36	27	WEED RES	0.94	0.37	12.4	7	1.705	1965–1998
37	24	PLANT DIS	0.93	0.37	10.4	7	1.795	1983–1995
38	40	REV PALAEOBOT PALYNO	0.93	0.36	10.5	7	1.170	1967–2001
39	25	PLANTA MED	0.94	0.44	11.0	7	1.746	1977–2000
40	37	PLANT SYST EVOL	0.93	0.43	10.8	7	1.239	1973–2000
41	23	ENVIRON EXP BOT	0.88	0.37	9.4	6	1.820	1975–2003
42	44	PLANT BREEDING	0.89	0.39	9.2	6	0.954	1985–1996
43	50	BOT J LINN SOC	0.89	0.43	10.6	6	0.805	1970–1998
44	48	PLANT GROWTH REGUL	0.86	0.35	9.1	5	0.903	1981–2000
45	43	CAN J PLANT PATHOL	0.87	0.41	9.2	6	0.971	1983–1996
46	47	EUPHYTICA	0.84	0.38	8.0	5	0.907	1984–1998
47	53	WEED TECHNOL	0.82	0.40	8.2	5	0.626	1986–2000
48	51	PHOTOSYNTHETICA	0.80	0.35	7.1	5	0.782	1979–2000
49	56	J PLANT NUTR	0.78	0.38	7.9	4	0.441	1978–1999
50	55	CAN J PLANT SCI	0.75	0.41	6.3	4	0.484	1988–1997
51	58	COMMUN SOIL SCI PLAN	0.72	0.41	8.9	4	0.302	1973–1995
52	41	BRYOLOGIST	0.71	0.40	6.8	4	1.010	1979–2001
53	49	J PHYTOPATHOL	0.65	0.38	4.8	3	0.817	1993–2000
54	54	NOVA HEDWIGIA	0.61	0.41	4.7	3	0.603	1976–1997
55	38	BIOL PLANTARUM	0.57	0.38	4.3	3	1.198	1960–2002
56	13	TAXON	0.58	0.49	6.4	2	2.519	1975–2003
57	57	SEED SCI TECHNOL	0.55	0.44	4.6	2	0.410	1976–1998
58	59	Z PFLANZENK PFLANZEN	0.54	0.43	4.1	2	0.239	1975–1999
59	60	NORD J BOT	0.55	0.47	4.3	2	0.129	1993–1997
60	52	S AFR J BOT	0.48	0.34	3.1	2	0.648	1993–1999

POLITICAL SCIENCE

ISI Category Description Political Science covers resources concerned with political studies, military studies, the electoral and legislative processes, political theory, history of political science, comparative studies of political systems, and the interaction of politics and other areas of science and social science.

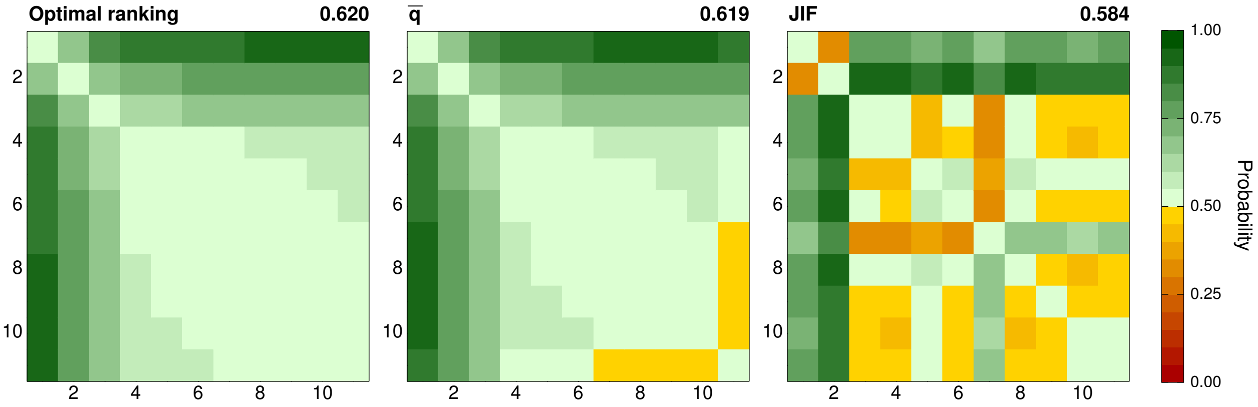

The color of each cell represents the probability of picking a paper with more citations from the higher-ranked journal. Green cells indicate adequate ranking, whereas red cells indicate that the ranking is inadequate (the probability is less than $\frac{1}{2}$). The value of M, the multi-class AUC statistic for each ranking scheme, is above each matrix.

Rank			$\mathbf{p_{ss}(q\|J)}$		n			Steady-state
**AUC**	**JIF**	**Journal abbreviation**	$\overline{\mathbf{q}}$	σ	$\overline{\mathbf{n}}$	**Q2**	**JIF**	**period**
1	2	PUBLIC CHOICE	0.73	0.43	8.1	4	0.446	1968–1995
2	1	ANN AM ACAD POLIT SS	0.34	0.47	3.5	1	0.736	1964–1995
3	7	CURR HIST	0.01	0.33	0.7	0	0.176	1993–2000
4	5	ISSUES STUD	-0.25	0.41	0.4	0	0.310	1967–2005
5	10	DISSENT	-0.31	0.43	0.4	0	0.118	1955–2003
6	11	PENSEE	-0.32	0.34	0.2	0	0.022	1966–2000
7	9	COMMENTARY	-1.34	1.30	0.2	0	0.133	2000–2003
8	3	NATION	-0.42	0.34	0.1	0	0.418	1978–2006
9	6	OSTEUROPA	-0.44	0.36	0.1	0	0.181	1969–2005
10	4	POLIT EKON	-0.50	0.35	0.1	0	0.363	1969–2005
11	8	NEW REPUBLIC	-0.72	0.46	0.1	0	0.148	1967–2005

POLYMER SCIENCE

ISI Category Description Polymer Science includes all resources dealing with the study, production, and technology of natural or synthetic polymers. Resources on polymeric materials are also covered in this category.

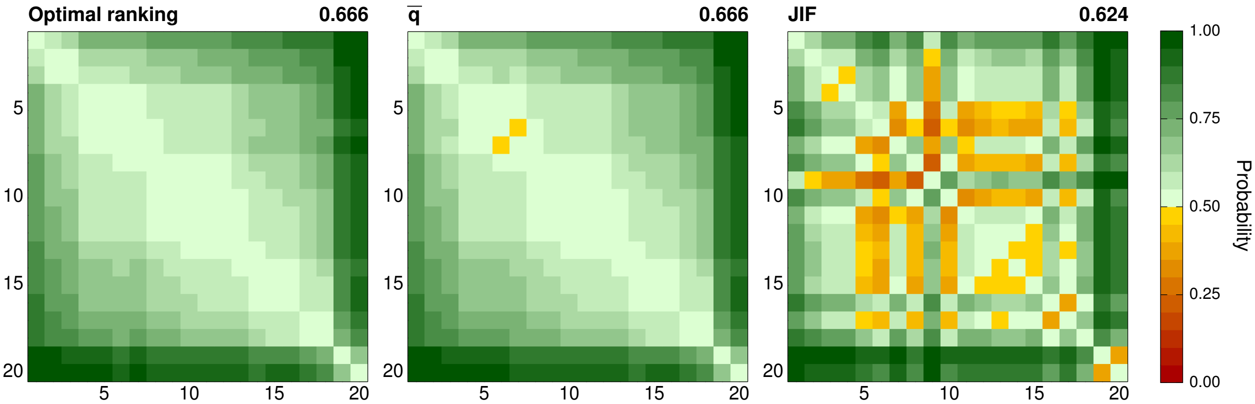

The color of each cell represents the probability of picking a paper with more citations from the higher-ranked journal. Green cells indicate adequate ranking, whereas red cells indicate that the ranking is inadequate (the probability is less than $\frac{1}{2}$). The value of M, the multi-class AUC statistic for each ranking scheme, is above each matrix.

Rank			$p_{ss}(q\|J)$		n			Steady-state
**AUC**	**JIF**	**Journal abbreviation**	$\bar{q}$	σ	$\bar{n}$	**Q2**	**JIF**	**period**
1	1	MACROMOLECULES	1.45	0.38	39.8	24	4.277	1968–1992
2	9	J POLYM SCI POL PHYS	1.32	0.39	30.6	18	1.622	1971–1987
3	2	J MEMBRANE SCI	1.25	0.40	23.6	15	3.442	1975–1994
4	4	POLYMER	1.13	0.38	17.2	11	2.773	1991–1994
5	3	J POLYM SCI POL CHEM	1.11	0.38	18.5	10	3.405	1971–1995
6	11	POLYM ENG SCI	1.11	0.42	19.9	10	1.414	1968–1992
7	7	CARBOHYD POLYM	1.11	0.34	14.6	10	1.784	1993–2000
8	15	POLYM J	1.03	0.38	15.0	9	1.146	1971–1993
9	12	J APPL POLYM SCI	1.02	0.39	13.2	8	1.306	1988–1994
10	14	POLYM COMPOSITE	0.99	0.41	13.5	8	1.163	1981–1997
11	17	RUBBER CHEM TECHNOL	0.98	0.41	12.8	7	0.569	1976–1997
12	13	COLLOID POLYM SCI	0.97	0.41	13.3	7	1.249	1973–1999
13	5	POLYM DEGRAD STABIL	0.93	0.38	10.0	7	2.174	1984–2000
14	8	SYNTHETIC MET	0.86	0.43	10.7	6	1.685	1994–1997
15	10	POLYM INT	0.84	0.39	8.2	5	1.475	1990–2000
16	6	EUR POLYM J	0.82	0.36	7.3	5	2.113	1991–2000
17	16	POLYM BULL	0.73	0.38	6.6	4	0.969	1994–2000
18	18	J REINF PLAST COMP	0.67	0.42	5.6	3	0.427	1987–1997
19	20	KOBUNSHI RONBUNSHU	0.21	0.41	1.6	1	0.169	1993–2001
20	19	MECH COMPOS MATER	-0.13	0.42	0.5	0	0.275	2002–2006

PSYCHIATRY

ISI Category Description Psychiatry covers resources on clinical, therapeutic, research, and community aspects of human mental, emotional, and behavioral disorders.

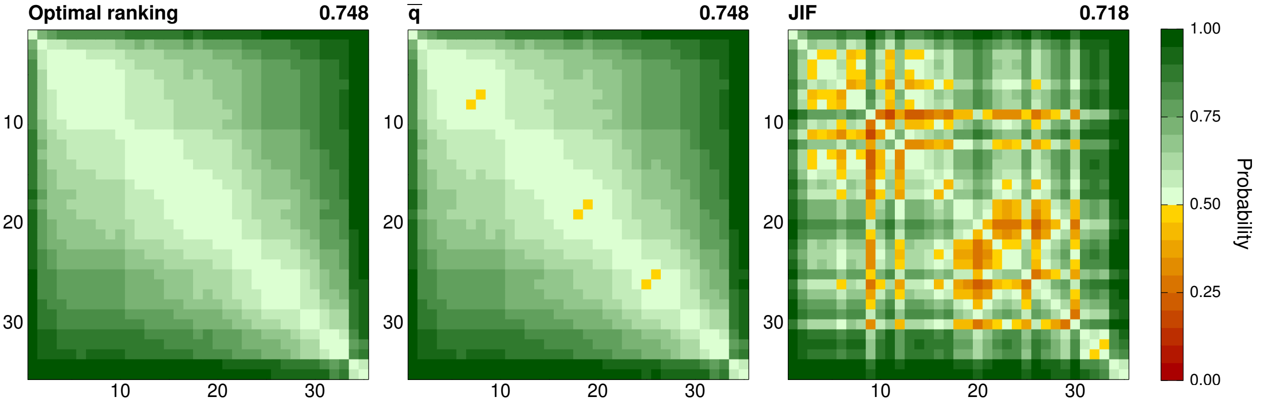

The color of each cell represents the probability of picking a paper with more citations from the higher-ranked journal. Green cells indicate adequate ranking, whereas red cells indicate that the ranking is inadequate (the probability is less than $\frac{1}{2}$). The value of M, the multi-class AUC statistic for each ranking scheme, is above each matrix.

**Rank**			$\mathbf{p_{ss}(q\|J)}$		**n**			**Steady-state**
**AUC**	**JIF**	**Journal abbreviation**	$\overline{\mathbf{q}}$	σ	$\overline{\mathbf{n}}$	**Q2**	**JIF**	**period**
1	1	ARCH GEN PSYCHIAT	1.97	0.36	141.5	94	13.936	1983–1996
2	2	AM J PSYCHIAT	1.67	0.43	62.0	41	8.250	1984–1999
3	11	PSYCHOSOM MED	1.54	0.39	49.8	31	3.857	1968–1997
4	7	J AM ACAD CHILD PSY	1.52	0.43	43.4	30	4.767	1987–1997
5	4	J CLIN PSYCHIAT	1.49	0.41	37.0	27	5.533	1989–1998
6	13	PSYCHOL MED	1.48	0.42	46.8	26	3.816	1970–1996
7	8	J CHILD PSYCHOL PSYC	1.45	0.40	40.1	25	4.404	1983–1994
8	5	BRIT J PSYCHIAT	1.46	0.40	41.3	25	5.436	1977–1998
9	14	J NEUROL NEUROSUR PS	1.45	0.40	40.5	25	3.630	1954–1995
10	3	BIOL PSYCHIAT	1.42	0.33	34.0	23	7.154	2000–2002
11	15	PSYCHOPHARMACOLOGY	1.36	0.36	31.1	20	3.625	1975–1997
12	17	J AFFECT DISORDERS	1.35	0.35	28.7	19	3.138	1982–1998
13	26	J NERV MENT DIS	1.35	0.39	32.0	19	1.957	1980–1995
14	10	SCHIZOPHR RES	1.33	0.37	25.8	19	4.264	1987–1999
15	6	SLEEP	1.30	0.37	26.8	17	5.126	1992–1999
16	23	PSYCHIAT RES	1.28	0.37	26.1	16	2.310	1987–1995
17	16	DRUG ALCOHOL DEPEN	1.27	0.36	21.6	16	3.213	1992–1999
18	30	INT J EAT DISORDER	1.24	0.40	25.8	15	1.839	1980–1997
19	24	COMPR PSYCHIAT	1.25	0.38	21.7	14	2.181	1984–1998
20	22	J PSYCHOSOM RES	1.22	0.40	24.8	14	2.322	1955–1999
21	27	AM J ORTHOPSYCHIAT	1.20	0.42	22.7	13	1.954	1974–1996
22	12	ACTA PSYCHIAT SCAND	1.16	0.42	21.1	12	3.857	1974–1997
23	18	INT CLIN PSYCHOPHARM	1.12	0.41	18.1	10	3.080	1985–2002
24	19	EUR ARCH PSY CLIN N	1.09	0.39	15.9	10	3.042	1983–2001
25	21	NEUROPSYCHOBIOLOGY	1.02	0.38	13.6	8	2.367	1976–2000

table continues on next page . . .

... table continued from previous page

Rank			$p_{ss}(q\|J)$		n			Steady-state
AUC	JIF	Journal abbreviation	$\overline{q}$	σ	$\overline{n}$	Q2	JIF	period
26	28	INT J GERIATR PSYCH	1.02	0.39	12.9	8	1.930	1986–2001
27	25	PSYCHOSOMATICS	1.00	0.43	13.3	8	2.015	1977–2001
28	9	PSYCHOTHER PSYCHOSOM	0.98	0.43	11.9	7	4.333	1979–2003
29	20	CAN J PSYCHIAT	0.88	0.41	9.7	6	2.531	1978–2002
30	29	AUST NZ J PSYCHIAT	0.85	0.40	9.1	5	1.900	1974–2001
31	31	J BEHAV THER EXP PSY	0.71	0.35	6.4	4	1.432	1996–2003
32	33	PSYCHIAT ANN	0.65	0.44	6.2	3	0.469	1982–1998
33	32	NERVENARZT	0.63	0.38	5.0	3	0.711	1971–2001
34	34	NERVENHEILKUNDE	-0.02	0.44	1.1	0	0.396	1982–2003
35	35	ZH NEVROPATOL PSIKH	-0.20	0.39	0.4	0	0.129	1956–1995

PSYCHOLOGY, APPLIED

ISI Category Description Psychology, Applied covers resources on organizational psychology, including selection, training, performance, and evaluation; organizational behavior; counseling and development; as well as aviation psychology and sports psychology.

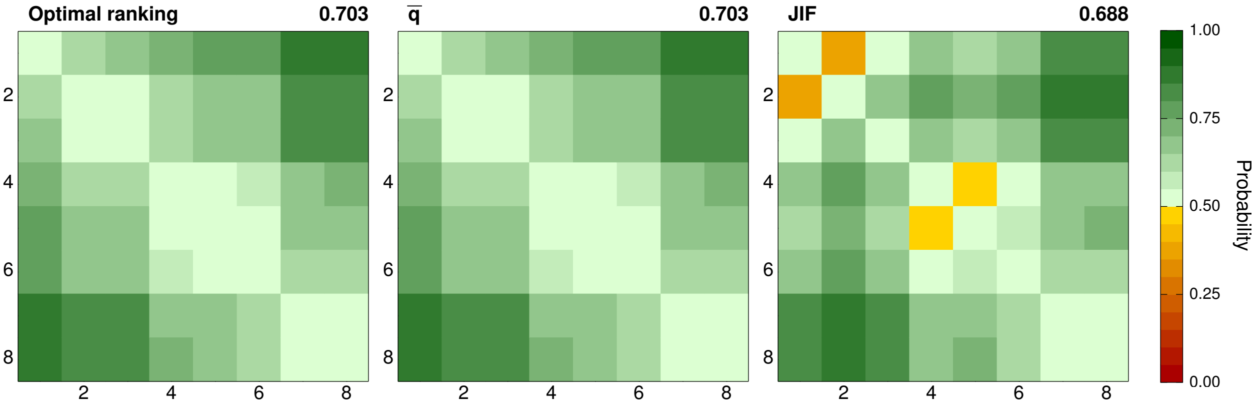

The color of each cell represents the probability of picking a paper with more citations from the higher-ranked journal. Green cells indicate adequate ranking, whereas red cells indicate that the ranking is inadequate (the probability is less than $\frac{1}{2}$). The value of M, the multi-class AUC statistic for each ranking scheme, is above each matrix.

Rank			$p_{ss}(q\|J)$		n			Steady-state
**AUC**	**JIF**	**Journal abbreviation**	$\bar{q}$	σ	$\bar{n}$	**Q2**	**JIF**	**period**
1	2	J APPL PSYCHOL	1.50	0.40	44.9	27	2.851	1974–1997
2	1	J COUNS PSYCHOL	1.27	0.34	23.7	16	2.924	1976–1998
3	3	J VOCAT BEHAV	1.27	0.32	22.7	16	2.263	1984–1997
4	5	HUM FACTORS	1.08	0.40	15.6	10	0.861	1980–1996
5	4	RES Q EXERCISE SPORT	1.03	0.41	14.4	8	0.982	1979–1999
6	6	ERGONOMICS	0.96	0.46	13.1	7	0.826	1957–1994
7	7	J COUNS DEV	0.74	0.43	7.3	4	0.564	1984–2001
8	8	J COLL STUDENT DEV	0.68	0.42	6.0	3	0.537	1978–2000

PSYCHOLOGY, BIOLOGICAL

ISI Category Description Psychology, Biological includes resources concerned with the biological basis of psychological states and processes. Biopsychology, psychophysiology, psychopharmacology, and comparative psychology resources are covered in this category.

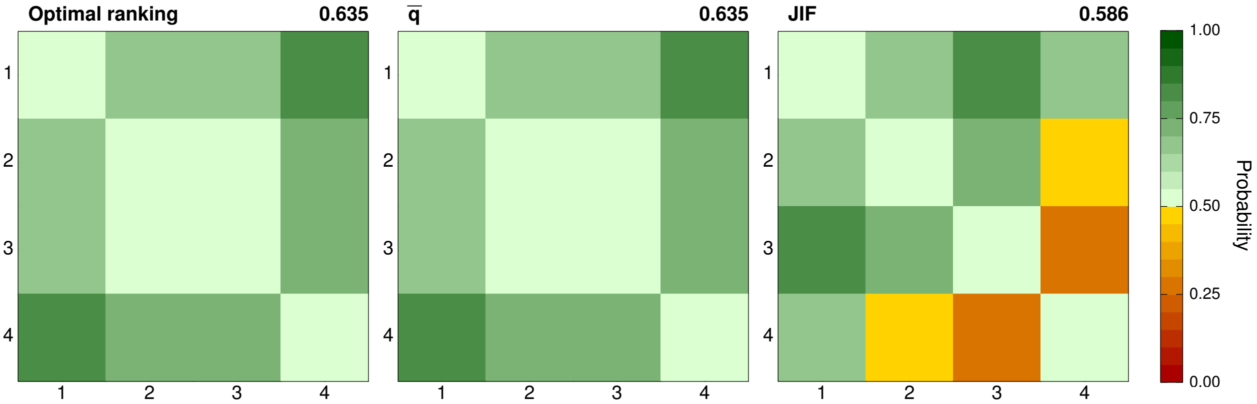

The color of each cell represents the probability of picking a paper with more citations from the higher-ranked journal. Green cells indicate adequate ranking, whereas red cells indicate that the ranking is inadequate (the probability is less than $\frac{1}{2}$). The value of M, the multi-class AUC statistic for each ranking scheme, is above each matrix.

**Rank**			$\mathbf{p_{ss}(q\|J)}$		**n**			**Steady-state**
**AUC**	**JIF**	**Journal abbreviation**	$\overline{\mathbf{q}}$	σ	$\overline{\mathbf{n}}$	**Q2**	**JIF**	**period**
1	1	PSYCHOPHYSIOLOGY	1.47	0.36	41.8	27	3.159	1985–1995
2	4	J EXP ANAL BEHAV	1.22	0.38	23.8	14	1.221	1970–1991
3	2	PHYSIOL BEHAV	1.20	0.37	20.5	13	2.445	1975–1991
4	3	BEHAV PROCESS	0.87	0.39	9.0	5	1.478	1975–2000

PSYCHOLOGY, CLINICAL

ISI Category Description Psychology, Clinical covers resources concerned with the combination of psychological therapy and clinical treatment such as behavior research and therapy, cognitive therapy, family therapy, marital and sexual therapy, psychotherapy, and rehabilitation psychology.

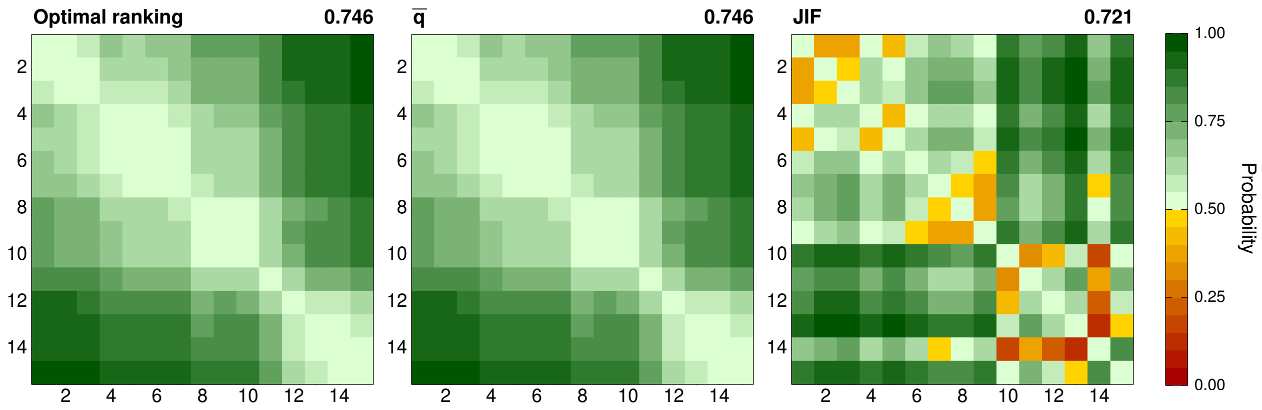

The color of each cell represents the probability of picking a paper with more citations from the higher-ranked journal. Green cells indicate adequate ranking, whereas red cells indicate that the ranking is inadequate (the probability is less than $\frac{1}{2}$). The value of M, the multi-class AUC statistic for each ranking scheme, is above each matrix.

**Rank**			$\mathbf{p_{ss}(q \mid J)}$		**n**			**Steady-state**
**AUC**	**JIF**	**Journal abbreviation**	$\overline{\mathbf{q}}$	σ	$\overline{\mathbf{n}}$	**Q2**	**JIF**	**period**
1	3	J CONSULT CLIN PSYCH	1.70	0.42	68.6	46	4.026	1980–1995
2	2	J ABNORM PSYCHOL	1.62	0.38	61.1	40	4.372	1977–1997
3	5	HEALTH PSYCHOL	1.56	0.34	49.6	34	3.693	1983–1996
4	1	J CLIN PSYCHIAT	1.49	0.41	37.0	27	5.533	1989–1998
5	4	PSYCHOL MED	1.48	0.42	46.8	26	3.816	1970–1996
6	9	J CLIN EXP NEUROPSYC	1.42	0.36	37.2	23	1.590	1984–1993
7	6	BEHAV RES THER	1.39	0.39	35.8	22	2.887	1962–1996
8	8	INT J EAT DISORDER	1.24	0.40	25.8	15	1.839	1980–1997
9	14	J APPL BEHAV ANAL	1.24	0.37	22.5	15	0.491	1983–1997
10	7	ADDICT BEHAV	1.22	0.36	24.0	14	1.849	1974–1998
11	11	J PERS ASSESS	1.06	0.40	17.1	9	1.175	1984–1997
12	12	J CLIN PSYCHOL	0.81	0.40	9.1	5	1.048	1955–2000
13	10	J BEHAV THER EXP PSY	0.71	0.35	6.4	4	1.432	1996–2003
14	15	AM BEHAV SCI	0.64	0.45	6.5	3	0.466	1965–2001
15	13	PSYCHOTHER PSYCH MED	0.56	0.40	4.4	2	1.043	1982–2002

PSYCHOLOGY, DEVELOPMENTAL

ISI Category Description Psychology, Developmental covers resources concerned with the study of developmental changes in social and cognitive abilities. Key areas include adult development and aging, child and adolescent psychology, cognitive, perceptual, motor and language development as well as psychosocial and personality development.

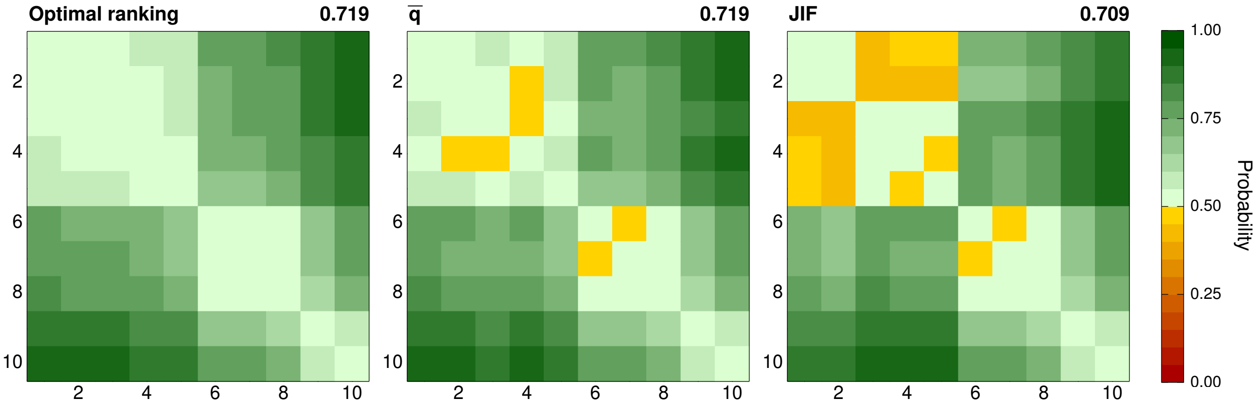

The color of each cell represents the probability of picking a paper with more citations from the higher-ranked journal. Green cells indicate adequate ranking, whereas red cells indicate that the ranking is inadequate (the probability is less than $\frac{1}{2}$). The value of M, the multi-class AUC statistic for each ranking scheme, is above each matrix.

Rank			$p_{ss}(q\|J)$		n			Steady-state
**AUC**	**JIF**	**Journal abbreviation**	$\overline{q}$	σ	$\overline{n}$	**Q2**	**JIF**	**period**
1	3	CHILD DEV	1.60	0.37	54.4	35	3.893	1984–1995
2	5	PSYCHOL AGING	1.52	0.34	42.1	30	2.828	1986–1994
3	4	DEV PSYCHOL	1.53	0.35	45.8	30	3.556	1985–1994
4	1	J AM ACAD CHILD PSY	1.52	0.43	43.4	30	4.767	1987–1997
5	2	J CHILD PSYCHOL PSYC	1.45	0.40	40.1	25	4.404	1983–1994
6	7	J EXP CHILD PSYCHOL	1.15	0.39	20.0	12	2.062	1963–1999
7	6	J ADOLESCENT HEALTH	1.17	0.39	16.4	12	2.710	1993–1999
8	8	SEX ROLES	1.08	0.38	15.4	10	0.942	1974–1992
9	9	ADOLESCENCE	0.85	0.42	8.6	5	0.768	1984–1995
10	10	J GENET PSYCHOL	0.66	0.40	5.9	3	0.731	1969–1998

PSYCHOLOGY, EDUCATIONAL

ISI Category Description Psychology, Educational includes resources on educational psychology, educational measurement, creative behavior, instructional science, reading research, and school psychology.

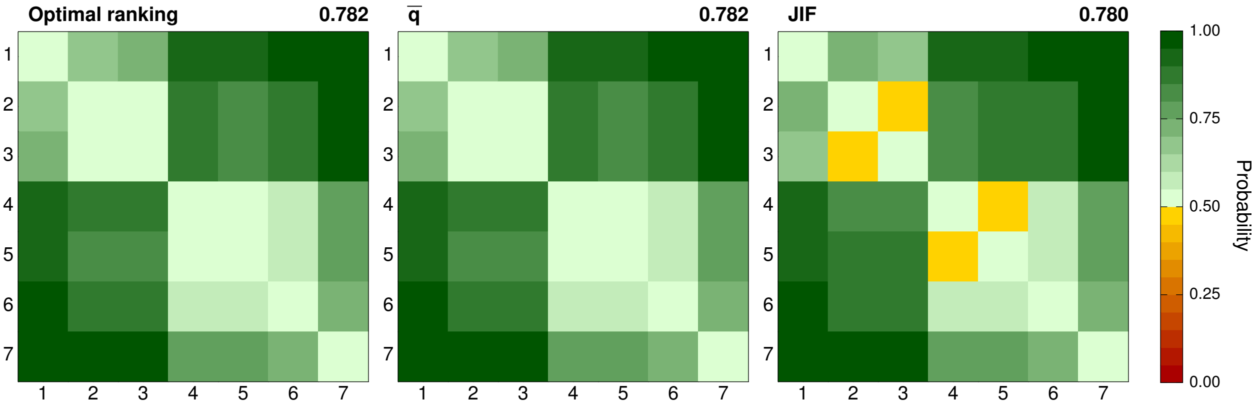

The color of each cell represents the probability of picking a paper with more citations from the higher-ranked journal. Green cells indicate adequate ranking, whereas red cells indicate that the ranking is inadequate (the probability is less than $\frac{1}{2}$). The value of M, the multi-class AUC statistic for each ranking scheme, is above each matrix.

Rank			$\mathbf{p_{ss}(q\|J)}$		n			Steady-state
**AUC**	**JIF**	**Journal abbreviation**	$\overline{\mathbf{q}}$	σ	$\overline{\mathbf{n}}$	**Q2**	**JIF**	**period**
1	1	CHILD DEV	1.60	0.37	54.4	35	3.893	1984–1995
2	3	J EDUC PSYCHOL	1.31	0.40	30.9	18	2.025	1978–1997
3	2	J COUNS PSYCHOL	1.27	0.34	23.7	16	2.924	1976–1998
4	5	PSYCHOL SCHOOLS	0.62	0.40	4.8	3	0.538	1963–2002
5	4	EDUC PSYCHOL MEAS	0.61	0.44	6.6	3	0.921	1964–2000
6	6	J EXP EDUC	0.45	0.44	3.8	2	0.484	1956–2002
7	7	VOP PSIKHOL+	-0.02	0.45	0.9	0	0.268	1965–2005

PSYCHOLOGY, EXPERIMENTAL

ISI Category Description Psychology, Experimental covers resources concerned with consciousness; cognition and memory; visual, auditory, and speech perception; and ecological psychology.

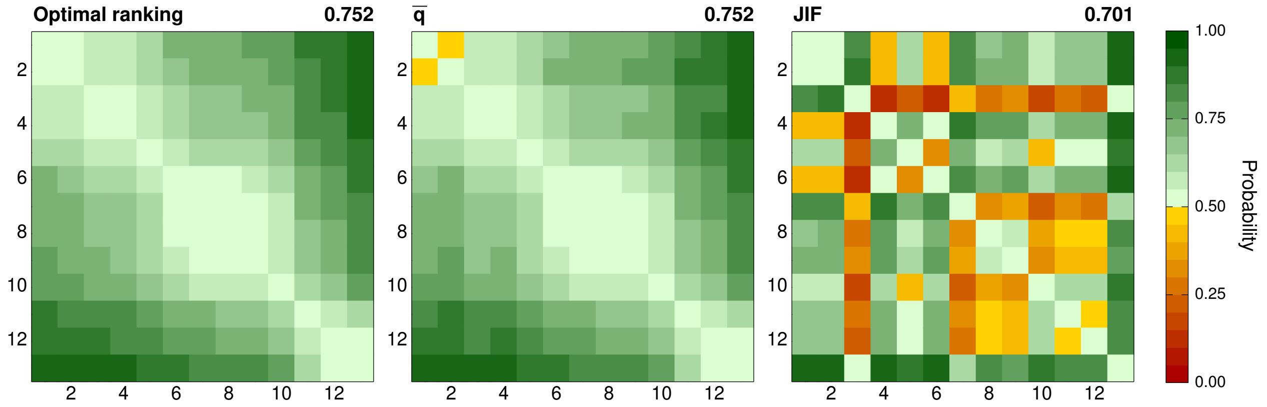

The color of each cell represents the probability of picking a paper with more citations from the higher-ranked journal. Green cells indicate adequate ranking, whereas red cells indicate that the ranking is inadequate (the probability is less than $\frac{1}{2}$). The value of M, the multi-class AUC statistic for each ranking scheme, is above each matrix.

Rank			$p_{ss}(q\|J)$		n			Steady-state
AUC	JIF	Journal abbreviation	$\overline{q}$	σ	$\overline{n}$	Q2	JIF	period
1	4	J EXP PSYCHOL LEARN	1.55	0.35	47.5	34	2.601	1992–1995
2	6	J EXP PSYCHOL HUMAN	1.56	0.38	52.1	32	2.261	1974–1995
3	2	PSYCHOPHYSIOLOGY	1.47	0.36	41.8	27	3.159	1985–1995
4	1	NEUROPSYCHOLOGIA	1.48	0.41	48.6	27	3.924	1964–1995
5	10	MEM COGNITION	1.38	0.40	34.0	21	1.512	1977–1997
6	5	BRAIN LANG	1.25	0.33	22.9	16	2.317	1992–1997
7	12	J EXP ANAL BEHAV	1.22	0.38	23.8	14	1.221	1970–1991
8	11	PERCEPT PSYCHOPHYS	1.20	0.41	23.5	13	1.482	1965–1996
9	8	J EXP CHILD PSYCHOL	1.15	0.39	20.0	12	2.062	1963–1999
10	9	PERCEPTION	1.07	0.45	17.7	9	1.585	1973–1995
11	7	ACTA PSYCHOL	0.84	0.55	13.2	5	2.094	1955–2001
12	3	BRAIN COGNITION	0.73	0.61	9.1	3	2.858	1995–1999
13	13	PERCEPT MOTOR SKILL	0.54	0.42	4.5	2	0.333	1970–1995

PSYCHOLOGY, MULTIDISCIPLINARY

ISI Category Description Psychology, Multidisciplinary covers resources with a general or interdisciplinary approach to the field. Resources on philosophical psychology, psychobiology, and the history of psychology are included in this category.

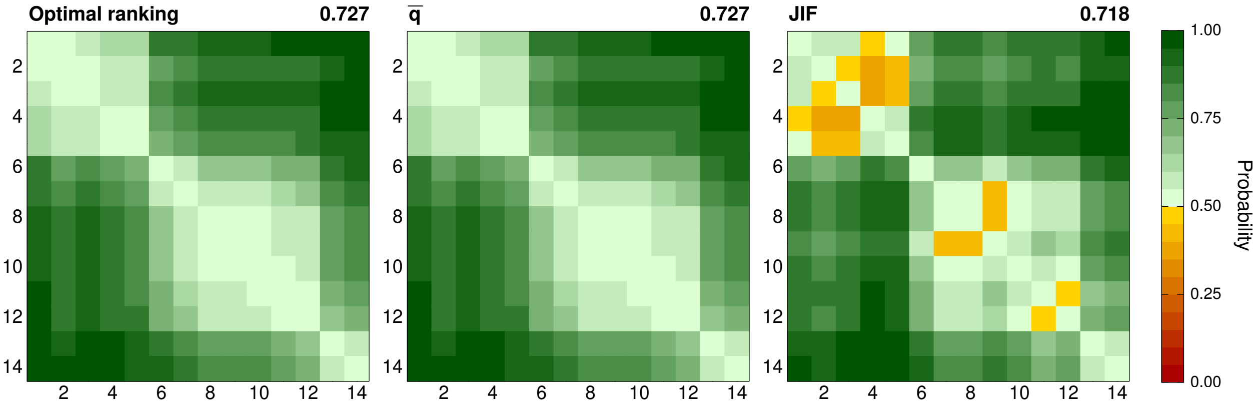

The color of each cell represents the probability of picking a paper with more citations from the higher-ranked journal. Green cells indicate adequate ranking, whereas red cells indicate that the ranking is inadequate (the probability is less than $\frac{1}{2}$). The value of M, the multi-class AUC statistic for each ranking scheme, is above each matrix.

Rank			$p_{ss}(q\|J)$		n			Steady-state
**AUC**	**JIF**	**Journal abbreviation**	$\overline{q}$	σ	$\overline{n}$	**Q2**	**JIF**	**period**
1	4	J ABNORM PSYCHOL	1.62	0.38	61.1	40	4.372	1977–1997
2	1	PSYCHOL BULL	1.56	0.53	79.9	32	12.725	1954–1999
3	5	PSYCHOSOM MED	1.54	0.39	49.8	31	3.857	1968–1997
4	3	PSYCHOL SCI	1.43	0.43	38.1	26	4.571	1989–1998
5	2	AM PSYCHOL	1.39	0.52	54.3	21	7.829	1972–1998
6	6	PROF PSYCHOL-RES PR	0.94	0.39	11.3	7	1.083	1982–1998
7	9	AM J PSYCHOL	0.83	0.45	10.5	5	0.698	1964–2000
8	7	PSYCHOL REC	0.68	0.41	6.5	3	0.937	1956–2002
9	8	J GENET PSYCHOL	0.66	0.40	5.9	3	0.731	1969–1998
10	10	J PSYCHOL	0.64	0.40	5.8	3	0.589	1964–1999
11	12	PSYCHOL REP	0.56	0.41	4.8	2	0.364	1972–1995
12	11	J GEN PSYCHOL	0.51	0.42	4.6	2	0.577	1956–2003
13	13	CESK PSYCHOL	0.16	0.45	1.3	1	0.279	1993–1999
14	14	PSIKHOL ZH	-0.01	0.39	0.8	0	0.202	1982–2001

PSYCHOLOGY, PSYCHOANALYSIS

ISI Category Description Psychology, Psychoanalysis includes resources concerned with psychoanalysis as a form of diagnosis and treatment that emphasizes the gradual integration of repressed memories into the total structure of the personality.

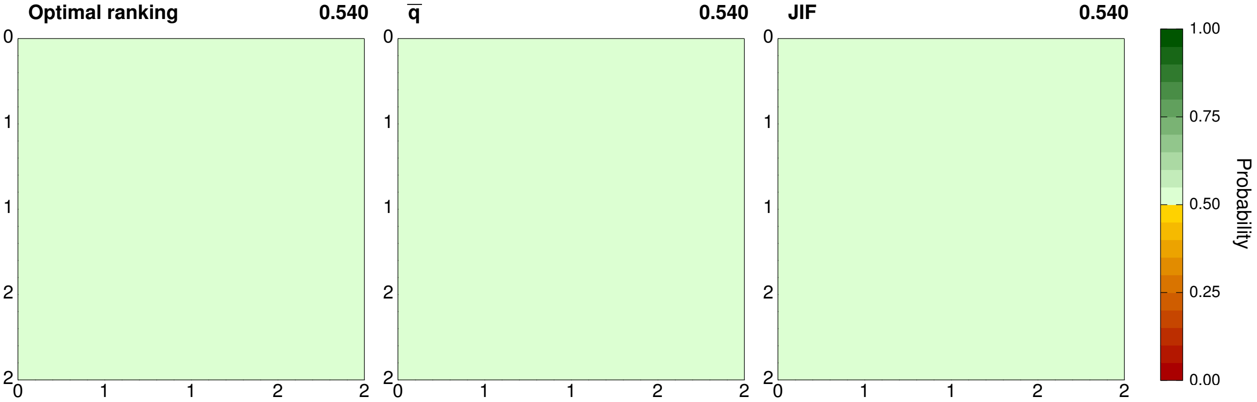

The color of each cell represents the probability of picking a paper with more citations from the higher-ranked journal. Green cells indicate adequate ranking, whereas red cells indicate that the ranking is inadequate (the probability is less than $\frac{1}{2}$). The value of M, the multi-class AUC statistic for each ranking scheme, is above each matrix.

**Rank**			$\mathbf{p_{ss}(q \mid J)}$		**n**			**Steady-state**
**AUC**	**JIF**	**Journal abbreviation**	$\overline{\mathbf{q}}$	σ	$\overline{\mathbf{n}}$	**Q2**	**JIF**	**period**
1	1	PSYCHOTHER PSYCHOSOM	0.98	0.43	11.9	7	4.333	1979–2003
2	2	INT J PSYCHOANAL	0.90	0.45	13.2	6	0.976	1955–1998

PSYCHOLOGY, SOCIAL

ISI Category Description Psychology, Social covers resources on the behavior of the individual in a social context. Areas included are group processes, interpersonal processes, intercultural relations, personality, social roles, persuasion, compliance, conformity, sex roles, and sexual orientation.

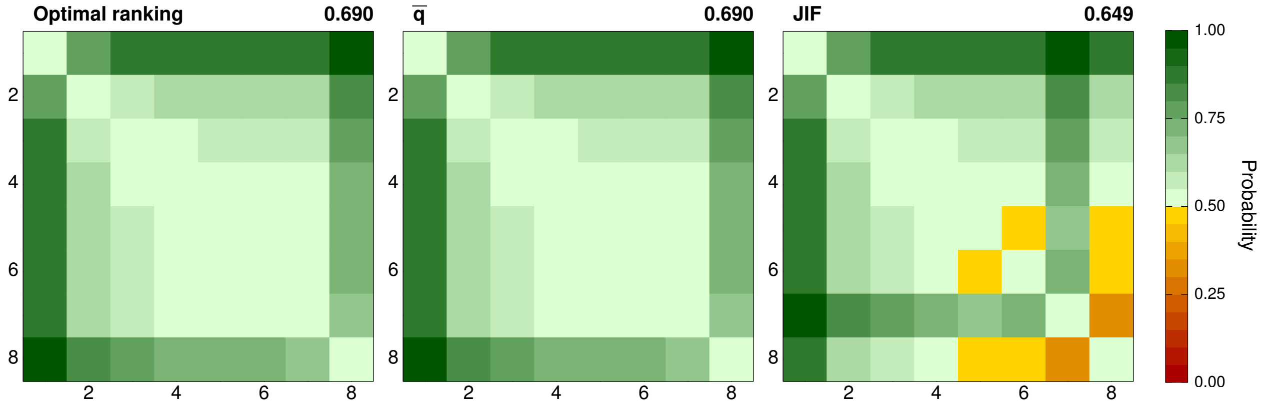

The color of each cell represents the probability of picking a paper with more citations from the higher-ranked journal. Green cells indicate adequate ranking, whereas red cells indicate that the ranking is inadequate (the probability is less than $\frac{1}{2}$). The value of M, the multi-class AUC statistic for each ranking scheme, is above each matrix.

Rank			$\mathbf{p_{ss}(q\|J)}$		n			Steady-state
**AUC**	**JIF**	**Journal abbreviation**	$\overline{\mathbf{q}}$	σ	$\overline{\mathbf{n}}$	**Q2**	**JIF**	**period**
1	1	J PERS SOC PSYCHOL	1.71	0.33	69.3	48	4.223	1990–1995
2	2	PERS SOC PSYCHOL B	1.29	0.38	25.0	16	2.419	1983–1998
3	3	CHILD ABUSE NEGLECT	1.14	0.31	18.3	12	1.623	1994–1996
4	4	PERS INDIV DIFFER	1.11	0.38	17.5	10	1.423	1979–1995
5	8	J APPL SOC PSYCHOL	1.09	0.40	17.2	10	0.566	1970–1996
6	6	SEX ROLES	1.08	0.38	15.4	10	0.942	1974–1992
7	5	J PERS ASSESS	1.06	0.40	17.1	9	1.175	1984–1997
8	7	J SOC PSYCHOL	0.75	0.40	7.1	4	0.655	1955–1996

PSYCHOLOGY

ISI Category Description Psychology is concerned with resources on the study of human behavior and mental processes. This category covers the biological and neurological underpinnings of perception, thought, and behavior; psychological development and change over the life span; in addition to emotional and mental disturbances and diseases and their treatment. Resources that report on animal behavior to illuminate human behavior and mental processes are also covered.

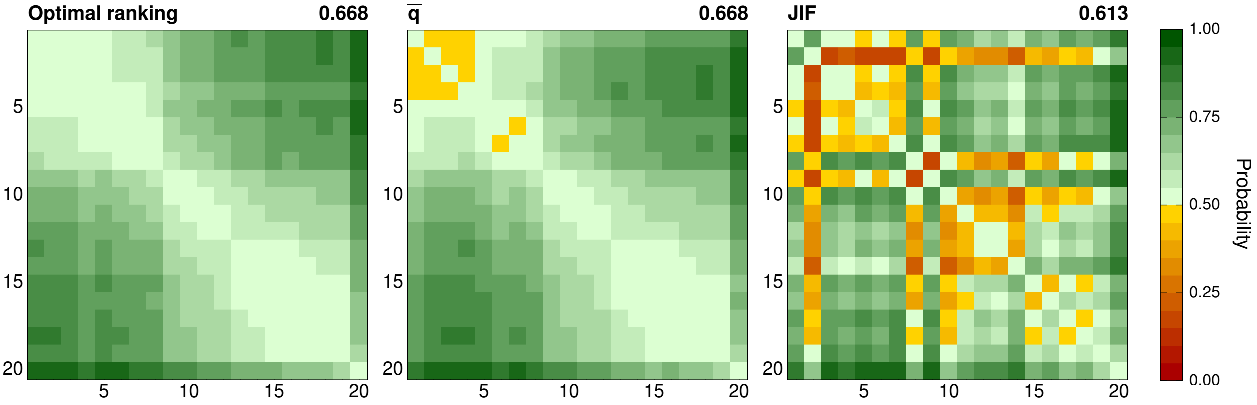

The color of each cell represents the probability of picking a paper with more citations from the higher-ranked journal. Green cells indicate adequate ranking, whereas red cells indicate that the ranking is inadequate (the probability is less than $\frac{1}{2}$). The value of M, the multi-class AUC statistic for each ranking scheme, is above each matrix.

**Rank**			$\mathbf{p_{ss}(q\|J)}$		**n**			**Steady-state**
**AUC**	**JIF**	**Journal abbreviation**	$\overline{\mathbf{q}}$	σ	$\overline{\mathbf{n}}$	**Q2**	**JIF**	**period**
1	7	J EXP PSYCHOL LEARN	1.55	0.35	47.5	34	2.601	1992–1995
2	5	HEALTH PSYCHOL	1.56	0.34	49.6	34	3.693	1983–1996
3	9	J EXP PSYCHOL HUMAN	1.56	0.38	52.1	32	2.261	1974–1995
4	1	PSYCHOL BULL	1.56	0.53	79.9	32	12.725	1954–1999
5	3	PSYCHOSOM MED	1.54	0.39	49.8	31	3.857	1968–1997
6	6	PSYCHOPHYSIOLOGY	1.47	0.36	41.8	27	3.159	1985–1995
7	4	PSYCHOL MED	1.48	0.42	46.8	26	3.816	1970–1996
8	14	J CLIN EXP NEUROPSYC	1.42	0.36	37.2	23	1.590	1984–1993
9	12	J STUD ALCOHOL	1.31	0.42	27.4	17	1.884	1974–1998
10	13	INT J EAT DISORDER	1.24	0.40	25.8	15	1.839	1980–1997
11	16	PERCEPT PSYCHOPHYS	1.20	0.41	23.5	13	1.482	1965–1996
12	11	DEV PSYCHOBIOL	1.17	0.37	18.6	12	1.946	1971–1997
13	18	HUM FACTORS	1.08	0.40	15.6	10	0.861	1980–1996
14	15	PERCEPTION	1.07	0.45	17.7	9	1.585	1973–1995
15	8	NEUROPSYCHOBIOLOGY	1.02	0.38	13.6	8	2.367	1976–2000
16	17	RES Q EXERCISE SPORT	1.03	0.41	14.4	8	0.982	1979–1999
17	10	PSYCHOSOMATICS	1.00	0.43	13.3	8	2.015	1977–2001
18	2	PSYCHOTHER PSYCHOSOM	0.98	0.43	11.9	7	4.333	1979–2003
19	19	ERGONOMICS	0.96	0.46	13.1	7	0.826	1957–1994
20	20	J GENET PSYCHOL	0.66	0.40	5.9	3	0.731	1969–1998

PUBLIC, ENVIRONMENTAL & OCCUPATIONAL HEALTH

ISI Category Description Public, Environmental & Occupational Health covers resources dealing with epidemiology, hygiene, and health; parasitic diseases and parasitology; tropical medicine; industrial medicine; occupational medicine; infection control; and preventive medicine. Also included are resources on environmental health; cancer causes and control; aviation, aerosol, and wilderness medicine.

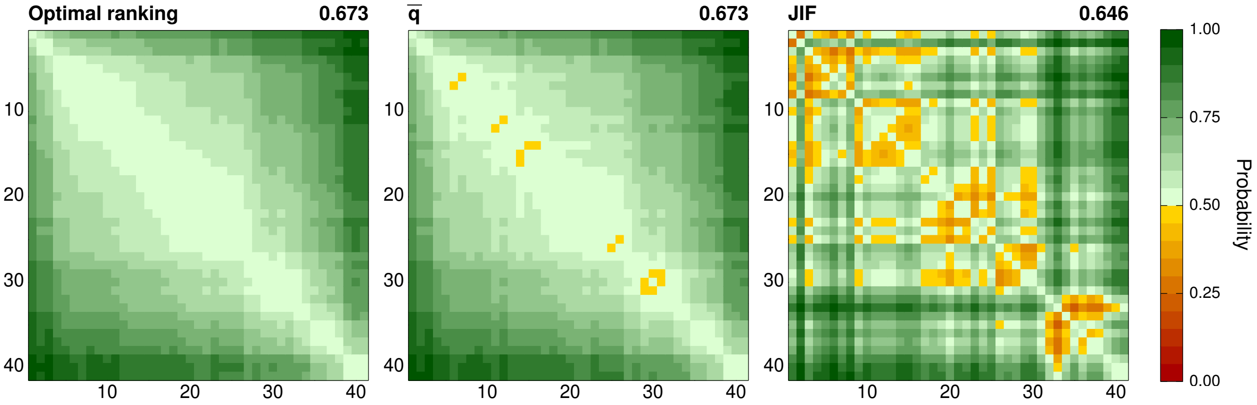

The color of each cell represents the probability of picking a paper with more citations from the higher-ranked journal. Green cells indicate adequate ranking, whereas red cells indicate that the ranking is inadequate (the probability is less than $\frac{1}{2}$). The value of M, the multi-class AUC statistic for each ranking scheme, is above each matrix.

**Rank**			**$p_{ss}(q\|J)$**		**n**			**Steady-state**
**AUC**	**JIF**	**Journal abbreviation**	**$\overline{q}$**	**σ**	**$\overline{n}$**	**Q2**	**JIF**	**period**
1	2	AM J EPIDEMIOL	1.61	0.41	57.6	36	5.241	1976–1995
2	8	AM J PUBLIC HEALTH	1.51	0.42	44.9	28	3.698	1986–1996
3	6	CANCER EPIDEM BIOMAR	1.46	0.35	32.9	25	4.289	1991–2000
4	5	EPIDEMIOLOGY	1.33	0.39	27.8	18	4.339	1990–2001
5	15	J CLIN EPIDEMIOL	1.33	0.44	32.7	18	2.440	1987–1998
6	7	MED CARE	1.28	0.40	28.6	16	3.745	1994–2000
7	16	PREV MED	1.28	0.45	26.2	16	2.390	1979–1998
8	14	AM J TROP MED HYG	1.25	0.37	22.2	15	2.546	1964–1996
9	10	J EPIDEMIOL COMMUN H	1.25	0.39	23.5	15	2.805	1977–1996
10	4	INT J EPIDEMIOL	1.25	0.40	23.3	15	4.517	1973–1998
11	23	EPIDEMIOL INFECT	1.20	0.34	20.1	13	1.809	1988–1997
12	25	SCAND J WORK ENV HEA	1.21	0.41	20.0	13	1.735	1976–1998
13	1	ENVIRON HEALTH PERSP	1.19	0.45	23.1	13	5.861	1985–2001
14	11	SOC SCI MED	1.17	0.39	20.7	12	2.749	1989–1999
15	13	ENVIRON RES	1.15	0.40	19.4	12	2.556	1966–1998
16	12	J ADOLESCENT HEALTH	1.17	0.39	16.4	12	2.710	1993–1999
17	29	INT ARCH OCC ENV HEA	1.13	0.38	16.3	11	1.520	1975–1995
18	30	AM J IND MED	1.12	0.36	15.1	11	1.433	1991–1998
19	18	T ROY SOC TROP MED H	1.10	0.40	17.0	10	2.030	1964–1998
20	9	AM J PREV MED	1.09	0.41	16.4	10	3.497	1986–2002
21	17	INFECT CONT HOSP EP	1.08	0.45	17.6	10	2.236	1979–2000
22	3	B WORLD HEALTH ORGAN	1.08	0.49	19.5	10	5.029	1960–1997
23	21	COMMUNITY DENT ORAL	1.06	0.35	13.3	9	1.870	1975–1999

table continues on next page . . .

. . . table continued from previous page

Rank			$p_{ss}(q\|J)$		n			Steady-state
AUC	JIF	Journal abbreviation	$\overline{q}$	σ	$\overline{n}$	Q2	JIF	period
24	24	STAT MED	1.04	0.40	16.9	9	1.737	1994–1997
25	22	J TOXICOL ENV HEALTH	1.03	0.38	14.6	8	1.811	1994–1996
26	19	J OCCUP ENVIRON MED	1.04	0.40	14.4	9	1.942	1987–1998
27	28	PUBLIC HEALTH REP	1.00	0.51	14.4	7	1.523	1987–1994
28	27	ACCIDENT ANAL PREV	0.95	0.38	11.2	7	1.587	1974–2001
29	20	ANN OCCUP HYG	0.89	0.40	10.4	6	1.919	1977–2001
30	31	ANN TROP MED PARASIT	0.89	0.38	9.1	6	1.191	1987–2000
31	35	HEALTH PHYS	0.89	0.43	10.5	6	0.902	1964–1994
32	26	EUR J EPIDEMIOL	0.88	0.38	8.8	6	1.605	1986–2000
33	37	AVIAT SPACE ENVIR MD	0.83	0.43	9.1	5	0.830	1974–1996
34	38	J ENVIRON SCI HEAL B	0.78	0.38	7.2	4	0.604	1975–1999
35	36	J SCHOOL HEALTH	0.71	0.50	7.8	4	0.856	1983–1999
36	34	PUBLIC HEALTH	0.66	0.40	5.7	3	0.926	1987–2000
37	32	CAN J PUBLIC HEALTH	0.62	0.46	5.5	3	1.157	1972–2001
38	39	RADIAT PROT DOSIM	0.57	0.43	4.6	2	0.446	1983–1999
39	40	REV SAUDE PUBL	0.40	0.40	2.7	1	0.343	1981–2001
40	41	TROP DOCT	0.32	0.42	2.2	1	0.291	1977–2000
41	33	SOZ PRAVENTIV MED	0.33	0.44	2.7	1	1.013	1987–2002

RADIOLOGY, NUCLEAR MEDICINE & MEDICAL IMAGING

ISI Category Description Radiology, Nuclear Medicine & Medical Imaging covers resources on radiation research in biology and biophysics. Resources in this category focus on interventional radiology, investigative radiology, neuroradiology, radiotherapy, and oncology. Nuclear Medicine resources are concerned with the diagnostic, therapeutic, and investigative use of radionuclides. Medical Imaging resources are concerned with computerized medical imaging and graphics.

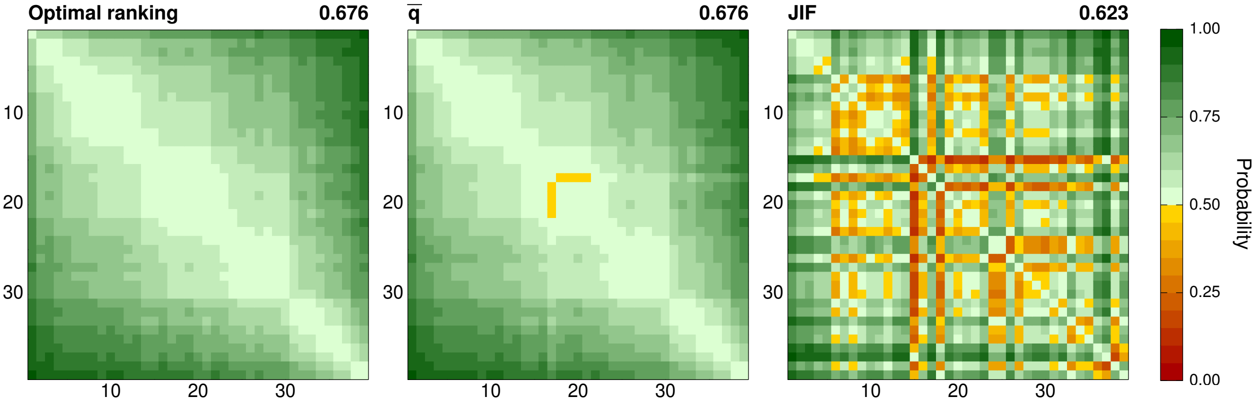

The color of each cell represents the probability of picking a paper with more citations from the higher-ranked journal. Green cells indicate adequate ranking, whereas red cells indicate that the ranking is inadequate (the probability is less than $\frac{1}{2}$). The value of M, the multi-class AUC statistic for each ranking scheme, is above each matrix.

**Rank**			$\mathbf{p_{ss}(q\|J)}$		**n**			**Steady-state**
**AUC**	**JIF**	**Journal abbreviation**	$\bar{q}$	σ	$\bar{n}$	**Q2**	**JIF**	**period**
1	1	RADIOLOGY	1.54	0.42	45.9	31	5.251	1986–1995
2	2	J NUCL MED	1.36	0.43	34.5	20	4.986	1977–1995
3	3	INT J RADIAT ONCOL	1.37	0.41	31.2	20	4.463	1982–1995
4	17	AM J ROENTGENOL	1.31	0.43	27.5	18	2.117	1975–1992
5	5	IEEE T MED IMAGING	1.25	0.40	27.5	15	3.757	1985–1999
6	14	AM J NEURORADIOL	1.23	0.39	22.3	14	2.279	1979–2000
7	4	RADIOTHER ONCOL	1.22	0.38	22.5	14	3.970	1983–2000
8	23	J COMPUT ASSIST TOMO	1.19	0.37	22.6	13	1.530	1981–1995
9	10	RADIAT RES	1.17	0.40	21.8	12	2.602	1954–1998
10	13	ULTRASOUND OBST GYN	1.15	0.35	18.5	12	2.288	1995–1997
11	26	INT J RADIAT BIOL	1.16	0.38	20.4	12	1.312	1972–1999
12	19	ULTRASOUND MED BIOL	1.15	0.41	19.6	12	2.011	1979–1998
13	11	RADIOL CLIN N AM	1.14	0.38	17.3	11	2.533	1964–2000
14	7	MED PHYS	1.09	0.44	18.6	9	3.571	1979–1998
15	21	NEURORADIOLOGY	1.08	0.41	15.8	10	1.625	1971–1994
16	30	SKELETAL RADIOL	1.07	0.35	12.2	9	1.176	1991–1995
17	9	PHYS MED BIOL	1.04	0.41	16.1	9	2.873	1955–2000
18	22	MAGN RESON IMAGING	1.04	0.39	14.6	9	1.580	1987–2000
19	29	J ULTRAS MED	1.04	0.36	13.2	9	1.189	1981–1995
20	28	BRIT J RADIOL	1.02	0.43	15.8	8	1.279	1954–1994
21	12	RADIOGRAPHICS	1.06	0.52	14.1	9	2.344	1989–2000
22	32	PEDIATR RADIOL	0.99	0.34	11.1	8	1.076	1972–1994

table continues on next page . . .

. . . table continued from previous page

Rank			$p_{ss}(q\|J)$		n			Steady-state
AUC	JIF	Journal abbreviation	$\overline{q}$	σ	$\overline{n}$	Q2	JIF	period
23	20	CLIN RADIOL	0.97	0.38	11.4	7	1.665	1973–1997
24	38	J CLIN ULTRASOUND	0.92	0.36	10.5	6	0.573	1983–1996
25	35	ACTA RADIOL	0.92	0.38	9.5	7	0.884	1962–1999
26	8	INVEST RADIOL	0.91	0.42	11.0	6	3.398	1987–2003
27	6	STRAHLENTHER ONKOL	0.91	0.32	8.1	6	3.682	2000–2004
28	16	NUCL MED BIOL	0.90	0.41	10.3	6	2.121	1985–2003
29	31	CARDIOVASC INTER RAD	0.89	0.39	10.0	6	1.149	1979–2000
30	34	HEALTH PHYS	0.89	0.43	10.5	6	0.902	1964–1994
31	27	NUCL MED COMMUN	0.76	0.40	7.8	4	1.283	1982–2002
32	24	EUR J RADIOL	0.72	0.38	6.4	4	1.332	1981–2003
33	25	ULTRASONICS	0.71	0.42	7.6	4	1.322	1965–2001
34	33	APPL RADIAT ISOTOPES	0.65	0.40	5.0	3	0.924	1993–2000
35	18	ULTRASCHALL MED	0.62	0.37	4.5	3	2.103	1982–2003
36	39	RADIAT PROT DOSIM	0.57	0.43	4.6	2	0.446	1983–1999
37	36	RADIOLOGE	0.52	0.39	3.7	2	0.696	1971–2001
38	15	CLIN NUCL MED	0.38	0.43	3.1	1	2.217	1995–2004
39	37	J RADIOL	0.32	0.40	2.1	1	0.600	1978–2000

REHABILITATION

ISI Category Description Rehabilitation covers resources on therapy to aid in the recovery or enhancement of physical, cognitive, or social abilities diminished by birth defect, disease, injury, or aging.

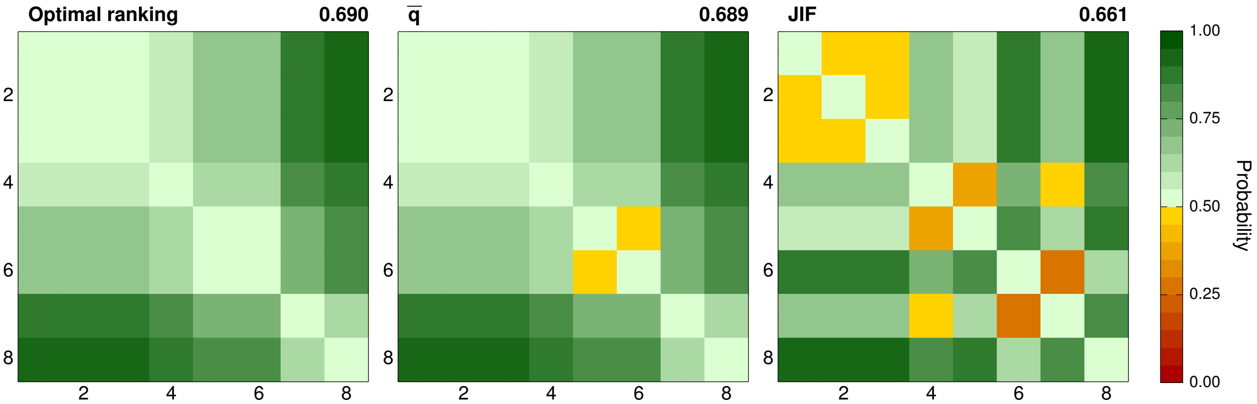

The color of each cell represents the probability of picking a paper with more citations from the higher-ranked journal. Green cells indicate adequate ranking, whereas red cells indicate that the ranking is inadequate (the probability is less than $\frac{1}{2}$). The value of M, the multi-class AUC statistic for each ranking scheme, is above each matrix.

Rank			$\mathbf{p_{ss}(q\|J)}$		n			Steady-state
**AUC**	**JIF**	**Journal abbreviation**	$\overline{\mathbf{q}}$	σ	$\overline{\mathbf{n}}$	**Q2**	**JIF**	**period**
1	3	PHYS THER	1.14	0.44	18.4	11	1.510	1989–1997
2	2	ARCH PHYS MED REHAB	1.13	0.44	18.5	11	1.826	1973–1999
3	1	EXCEPT CHILDREN	1.11	0.39	16.6	10	3.226	1985–2000
4	5	AM J PHYS MED REHAB	0.98	0.43	13.0	7	1.300	1972–1998
5	7	AM J OCCUP THER	0.80	0.41	8.0	5	0.713	1976–1996
6	4	MENT RETARD	0.81	0.45	7.5	4	1.373	1971–1998
7	6	J REHABIL	0.32	0.44	2.4	1	0.729	1974–2003
8	8	J VISUAL IMPAIR BLIN	0.09	0.43	1.2	0	0.413	1997–2006

REMOTE SENSING

ISI Category Description Remote Sensing includes resources on the technique of remote observation and of obtaining reliable information about physical objects and the environment through the process of recording, measuring, and interpreting photographic images and patterns of electromagnetic radiation from space. This category also covers resources on the applications of remote sensing in environmental, atmospheric, meteorological, geographic, and geoscientific observations. Resources on geographic information systems that deal in large part with remote sensing are also included.

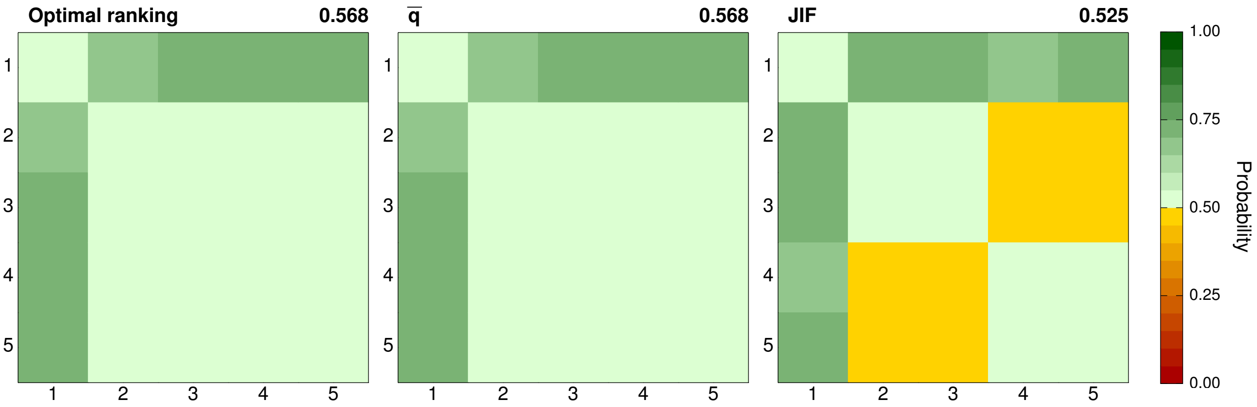

The color of each cell represents the probability of picking a paper with more citations from the higher-ranked journal. Green cells indicate adequate ranking, whereas red cells indicate that the ranking is inadequate (the probability is less than $\frac{1}{2}$). The value of M, the multi-class AUC statistic for each ranking scheme, is above each matrix.

Rank			$\mathbf{p_{ss}(q\|J)}$		n			Steady-state
**AUC**	**JIF**	**Journal abbreviation**	$\overline{\mathbf{q}}$	σ	$\overline{\mathbf{n}}$	**Q2**	**JIF**	**period**
1	1	REMOTE SENS ENVIRON	1.27	0.40	26.7	16	3.064	1980–1998
2	4	RADIO SCI	0.91	0.45	12.2	6	1.084	1965–1997
3	5	INT J REMOTE SENS	0.89	0.43	12.3	6	0.980	1982–2000
4	2	IEEE T GEOSCI REMOTE	0.83	0.49	11.6	5	1.752	1964–2006
5	3	PHOTOGRAMM ENG REM S	0.83	0.51	11.1	5	1.284	1978–1999

REPRODUCTIVE BIOLOGY

ISI Category Description Reproductive Biology includes resources that cover reproduction in humans, animals, and plants. This category ranges from the molecular biology of reproduction through reproductive nutrition, immunology, and toxicology.

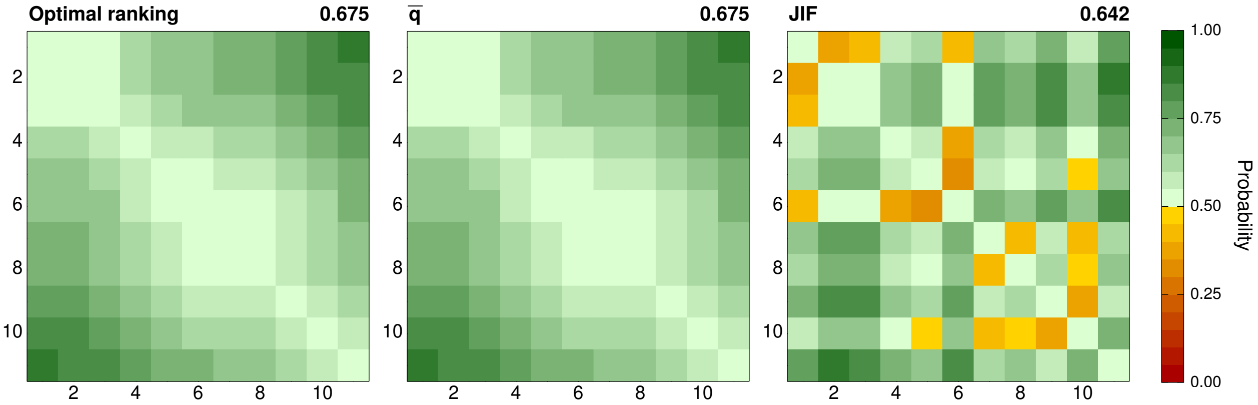

The color of each cell represents the probability of picking a paper with more citations from the higher-ranked journal. Green cells indicate adequate ranking, whereas red cells indicate that the ranking is inadequate (the probability is less than $\frac{1}{2}$). The value of M, the multi-class AUC statistic for each ranking scheme, is above each matrix.

Rank			$p_{ss}(q\|J)$		n			Steady-state
**AUC**	**JIF**	**Journal abbreviation**	$\overline{q}$	σ	$\overline{n}$	**Q2**	**JIF**	**period**
1	2	BIOL REPROD	1.39	0.36	31.9	21	3.498	1970–1997
2	3	FERTIL STERIL	1.39	0.38	32.0	21	3.277	1978–1993
3	6	MOL REPROD DEV	1.34	0.37	26.9	18	2.379	1989–1995
4	1	HUM REPROD	1.24	0.40	23.0	15	3.769	1988–1997
5	4	PLACENTA	1.15	0.37	17.9	11	2.969	1980–2000
6	10	AM J REPROD IMMUNOL	1.11	0.39	16.1	10	1.743	1979–1999
7	5	REPROD FERT DEVELOP	1.07	0.37	14.5	10	2.541	1988–1998
8	8	THERIOGENOLOGY	1.06	0.37	14.5	9	1.898	1994–2000
9	7	ANIM REPROD SCI	0.95	0.37	11.2	7	2.186	1979–2001
10	9	REPROD NUTR DEV	0.87	0.40	9.1	6	1.817	1979–2000
11	11	EUR J OBSTET GYN R B	0.76	0.41	7.2	4	1.273	1972–2000

RESPIRATORY SYSTEM

ISI Category Description Respiratory System covers resources on all aspects of respiratory and lung diseases, including their relation to cardiovascular and thoracic surgery and diseases.

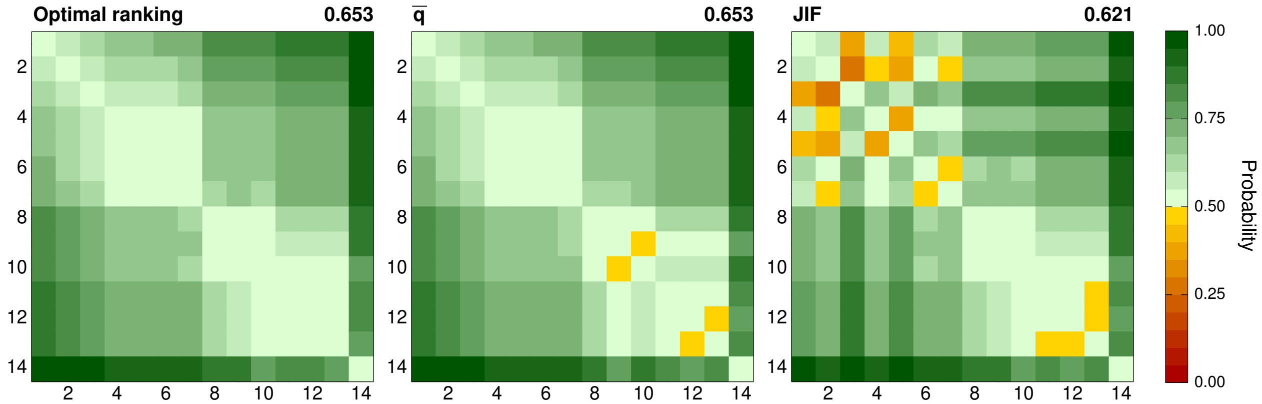

The color of each cell represents the probability of picking a paper with more citations from the higher-ranked journal. Green cells indicate adequate ranking, whereas red cells indicate that the ranking is inadequate (the probability is less than $\frac{1}{2}$). The value of M, the multi-class AUC statistic for each ranking scheme, is above each matrix.

Rank AUC	Rank JIF	Journal abbreviation	$p_{ss}(q\|J)$ $\overline{q}$	σ	n $\overline{n}$	n Q2	JIF	Steady-state period
1	3	AM J RESP CELL MOL	1.48	0.33	38.3	26	4.593	1988–1998
2	5	J THORAC CARDIOV SUR	1.42	0.39	34.0	23	3.560	1976–1995
3	1	THORAX	1.31	0.40	28.0	17	6.064	1976–2000
4	4	CHEST	1.22	0.45	24.3	14	3.924	1974–1996
5	7	ANN THORAC SURG	1.21	0.42	22.1	14	2.342	1973–1995
6	2	EUR RESPIR J	1.19	0.37	19.9	12	5.076	1994–2000
7	6	J HEART LUNG TRANSPL	1.15	0.37	18.6	12	2.830	1990–1998
8	8	EUR J CARDIO-THORAC	0.96	0.38	11.0	7	2.106	1989–2000
9	9	RESP MED	0.92	0.39	9.7	6	2.086	1995–2001
10	10	PEDIATR PULM	0.93	0.62	12.0	7	1.965	1985–1997
11	13	THORAC CARDIOV SURG	0.79	0.39	7.1	4	0.814	1978–2000
12	11	RESPIRATION	0.78	0.40	7.7	4	1.649	1968–2000
13	12	HEART LUNG	0.79	0.47	7.9	4	0.955	1986–2001
14	14	REV MAL RESPIR	0.13	0.45	1.3	0	0.436	1998–2001

RHEUMATOLOGY

ISI Category Description Rheumatology covers resources on clinical, therapeutic, and laboratory research about arthritis and rheumatism, the chronic degenerative autoimmune inflammatory diseases that primarily affect joints and connective tissue.

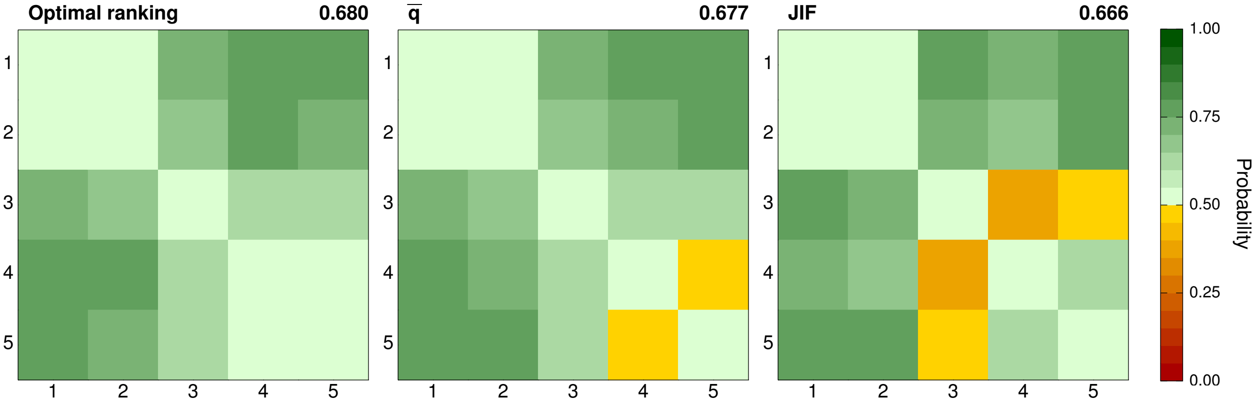

The color of each cell represents the probability of picking a paper with more citations from the higher-ranked journal. Green cells indicate adequate ranking, whereas red cells indicate that the ranking is inadequate (the probability is less than $\frac{1}{2}$). The value of M, the multi-class AUC statistic for each ranking scheme, is above each matrix.

**Rank**			$\mathbf{p_{ss}(q\|J)}$		**n**			**Steady-state**
**AUC**	**JIF**	**Journal abbreviation**	$\overline{\mathbf{q}}$	σ	$\overline{\mathbf{n}}$	**Q2**	**JIF**	**period**
1	1	ANN RHEUM DIS	1.29	0.37	27.1	17	5.767	1982–1989
2	2	J RHEUMATOL	1.25	0.38	23.7	15	2.940	1973–1999
3	4	CLIN EXP RHEUMATOL	0.98	0.39	12.2	8	2.189	1982–1999
4	5	CLIN RHEUMATOL	0.81	0.38	7.6	5	1.459	1981–2000
5	3	SCAND J RHEUMATOL	0.82	0.65	10.2	5	2.273	1973–1999

SOCIAL ISSUES

ISI Category Description Social Issues covers resources in a wide variety of topics addressing social problems for the individual, family, or society. Resources included in this category deal with death studies, issues in science and technology, gender studies, ethical studies, media studies, race and class, and the interaction of technology and society.

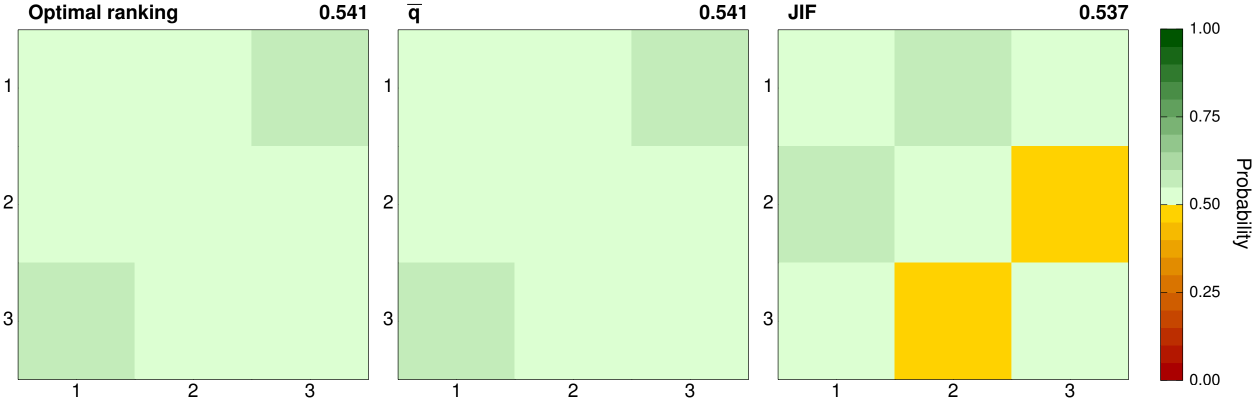

The color of each cell represents the probability of picking a paper with more citations from the higher-ranked journal. Green cells indicate adequate ranking, whereas red cells indicate that the ranking is inadequate (the probability is less than $\frac{1}{2}$). The value of M, the multi-class AUC statistic for each ranking scheme, is above each matrix.

Rank			$p_{ss}(q\|J)$		n			Steady-state
AUC	JIF	Journal abbreviation	$\overline{q}$	σ	$\overline{n}$	Q2	JIF	period
1	1	B ATOM SCI	-0.18	0.39	0.5	0	0.397	1978–2006
2	3	DISSENT	-0.31	0.43	0.4	0	0.118	1955–2003
3	2	COMMENTARY	-1.34	1.30	0.2	0	0.133	2000–2003

SOCIAL SCIENCES, BIOMEDICAL

ISI Category Description Social Sciences, Biomedical includes resources on the political and social effects of biomedical research. Areas covered include family planning, healthcare ethics, psycho-oncology, and sexual health.

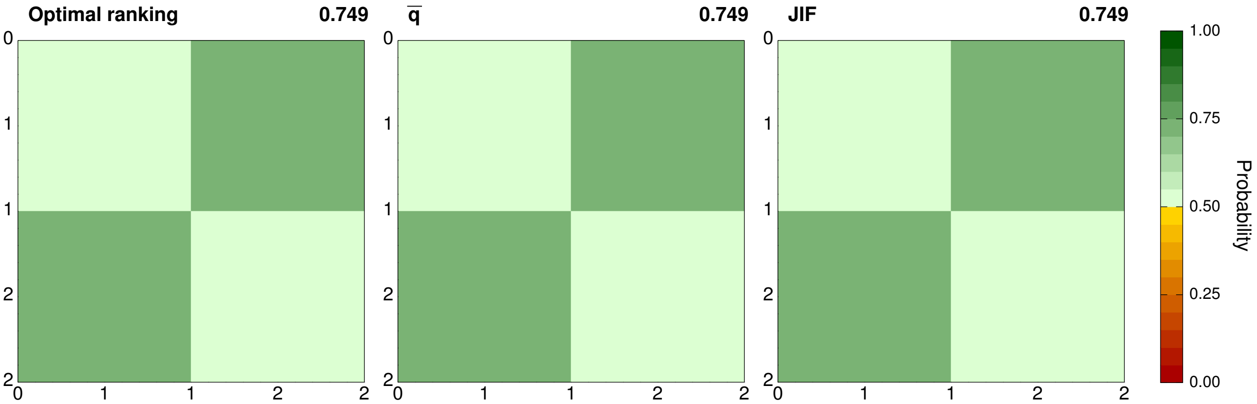

The color of each cell represents the probability of picking a paper with more citations from the higher-ranked journal. Green cells indicate adequate ranking, whereas red cells indicate that the ranking is inadequate (the probability is less than $\frac{1}{2}$). The value of M, the multi-class AUC statistic for each ranking scheme, is above each matrix.

Rank			$\mathbf{p_{ss}(q\|J)}$		n			Steady-state
**AUC**	**JIF**	**Journal abbreviation**	$\overline{\mathbf{q}}$	σ	$\overline{\mathbf{n}}$	**Q2**	**JIF**	**period**
1	1	SOC SCI MED	1.17	0.39	20.7	12	2.749	1989–1999
2	2	HASTINGS CENT REP	0.66	0.61	7.8	3	1.848	1992–1999

SOCIAL SCIENCES, INTERDISCIPLINARY

ISI Category Description Social Sciences, Interdisciplinary includes resources with an interdisciplinary approach to the field such as studies on social sciences and computers, time and society, evaluation practice, black studies, information science and society, homosexuality studies, childhood studies, and death studies.

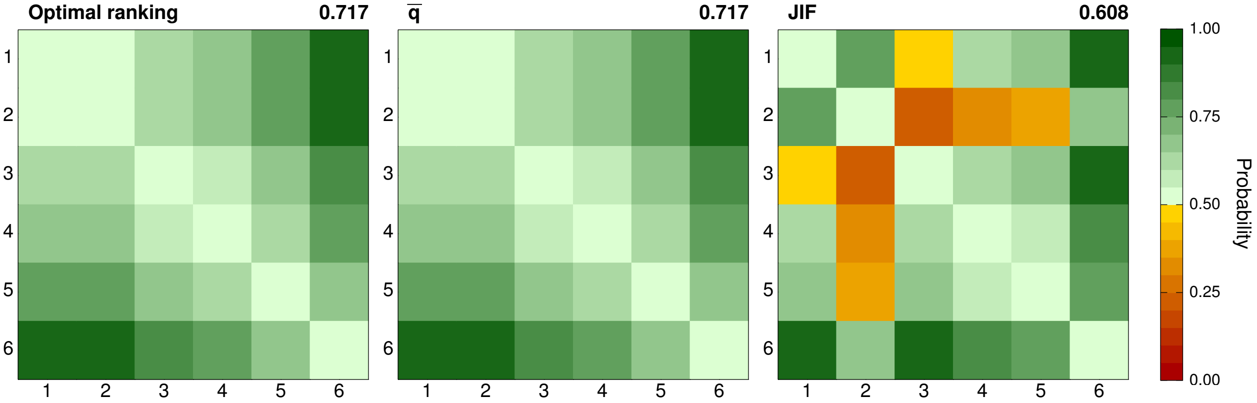

The color of each cell represents the probability of picking a paper with more citations from the higher-ranked journal. Green cells indicate adequate ranking, whereas red cells indicate that the ranking is inadequate (the probability is less than $\frac{1}{2}$). The value of M, the multi-class AUC statistic for each ranking scheme, is above each matrix.

Rank			$p_{ss}(q\|J)$		n			Steady-state
**AUC**	**JIF**	**Journal abbreviation**	$\overline{q}$	σ	$\overline{n}$	**Q2**	**JIF**	**period**
1	3	HUM RELAT	0.96	0.42	14.4	7	0.670	1955–2000
2	1	ACCIDENT ANAL PREV	0.95	0.38	11.2	7	1.587	1974–2001
3	4	SOC SCI QUART	0.75	0.43	7.6	4	0.641	1969–2000
4	5	AM BEHAV SCI	0.64	0.45	6.5	3	0.466	1965–2001
5	2	ANN AM ACAD POLIT SS	0.34	0.47	3.5	1	0.736	1964–1995
6	6	SOCIETY	-0.06	0.44	0.9	0	0.049	1973–2004

SOCIAL SCIENCES, MATHEMATICAL METHODS

ISI Category Description Social Sciences, Mathematical Methods covers resources concerned with the quantitative methodologies used for research in social sciences such as mathematical modeling and statistical techniques for psychological, sociological, and economic data evaluation.

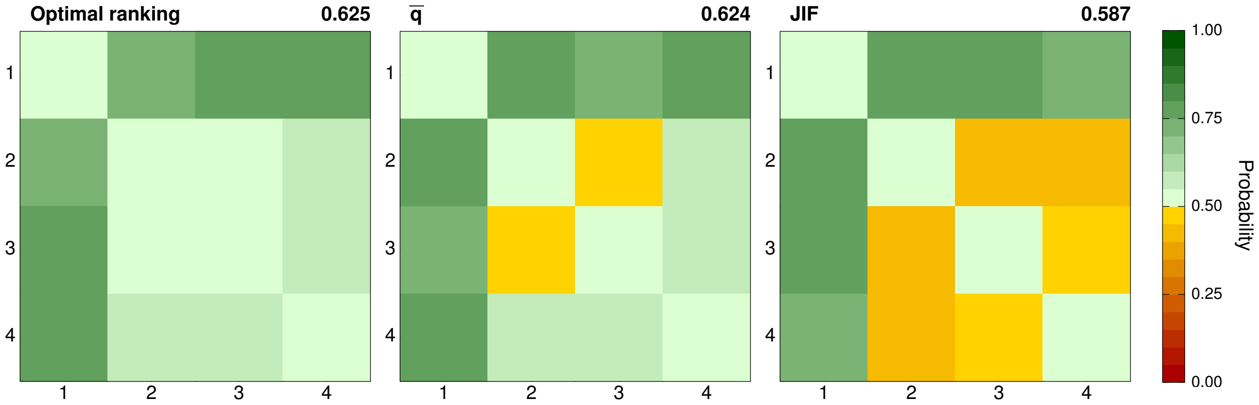

The color of each cell represents the probability of picking a paper with more citations from the higher-ranked journal. Green cells indicate adequate ranking, whereas red cells indicate that the ranking is inadequate (the probability is less than $\frac{1}{2}$). The value of M, the multi-class AUC statistic for each ranking scheme, is above each matrix.

Rank			$p_{ss}(q\|J)$		n			Steady-state
**AUC**	**JIF**	**Journal abbreviation**	$\overline{q}$	σ	$\overline{n}$	**Q2**	**JIF**	**period**
1	1	ECONOMETRICA	1.58	0.45	88.6	35	2.402	1980–1994
2	4	J ECONOMETRICS	1.13	0.45	28.1	11	1.669	1979–1996
3	3	REV ECON STAT	1.13	0.42	21.4	11	1.766	1964–1999
4	2	RISK ANAL	1.04	0.45	16.2	9	1.938	1983–1999

SOCIAL WORK

ISI Category Description Social Work covers resources concerned with homelessness, social casework, social services, social work education, public welfare, family counseling, child welfare and abuse, social work administration, social work with groups, and gerontological social work.

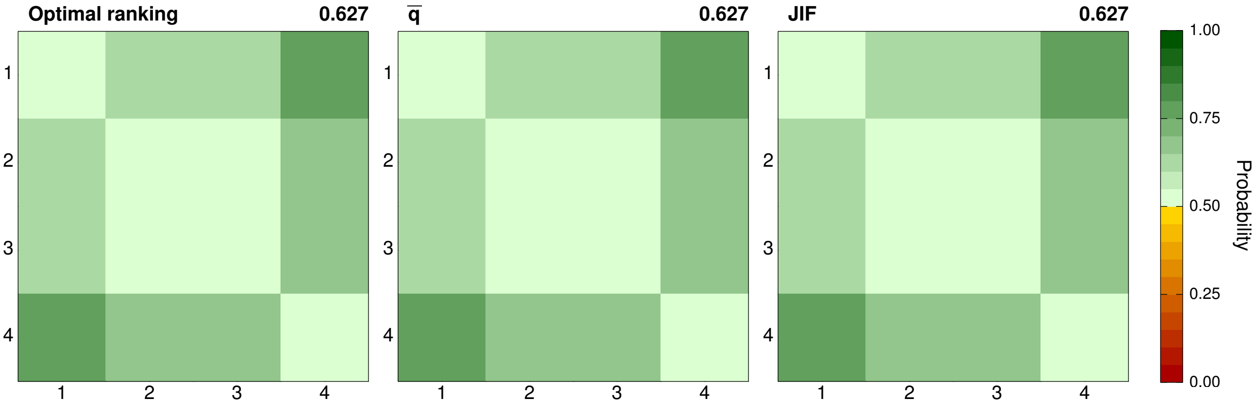

The color of each cell represents the probability of picking a paper with more citations from the higher-ranked journal. Green cells indicate adequate ranking, whereas red cells indicate that the ranking is inadequate (the probability is less than $\frac{1}{2}$). The value of M, the multi-class AUC statistic for each ranking scheme, is above each matrix.

Rank			$p_{ss}(q\|J)$		n			Steady-state
**AUC**	**JIF**	**Journal abbreviation**	$\overline{q}$	σ	$\overline{n}$	**Q2**	**JIF**	**period**
1	1	CHILD ABUSE NEGLECT	1.14	0.31	18.3	12	1.623	1994–1996
2	2	SOC WORK	0.98	0.35	11.0	8	0.779	1979–1999
3	3	FAM RELAT	0.96	0.38	11.6	7	0.731	1981–1998
4	4	CHILD WELFARE	0.71	0.41	6.3	4	0.500	1977–2001

SOCIOLOGY

ISI Category Description Sociology covers resources that focus on the study of human society, social structures, and social change as well as human behavior as it is shaped by social forces. Areas covered in this category include community studies, socio-ethnic problems, leisure and tourism, rural sociology, sociobiology, social deviance, gender studies, the sociology of law, the sociology of religion, and comparative sociology.

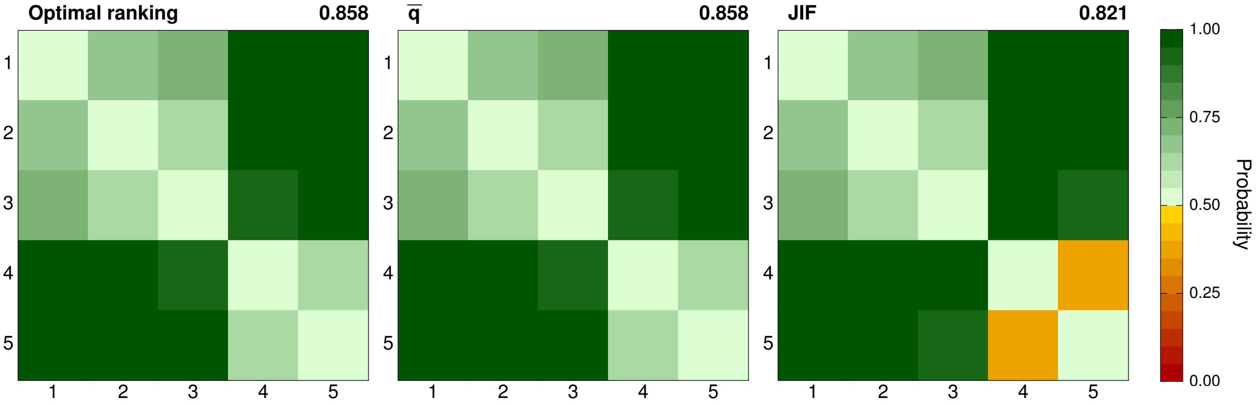

The color of each cell represents the probability of picking a paper with more citations from the higher-ranked journal. Green cells indicate adequate ranking, whereas red cells indicate that the ranking is inadequate (the probability is less than $\frac{1}{2}$). The value of M, the multi-class AUC statistic for each ranking scheme, is above each matrix.

Rank			$p_{ss}(q\|J)$		n			Steady-state
**AUC**	**JIF**	**Journal abbreviation**	$\overline{q}$	σ	$\overline{n}$	**Q2**	**JIF**	**period**
1	1	AM SOCIOL REV	1.60	0.43	61.1	36	3.205	1955–1996
2	2	J MARRIAGE FAM	1.34	0.41	31.6	19	1.763	1975–1994
3	3	SOC FORCES	1.16	0.43	20.5	11	1.214	1971–1998
4	5	SOCIETY	-0.06	0.44	0.9	0	0.049	1973–2004
5	4	SOTSIOL ISSLED+	-0.36	0.38	0.2	0	0.227	1982–2004

SOIL SCIENCE

ISI Category Description Soil Science covers resources concerning many aspects of the formation, nature, distribution, and utilization of soils including soil biology and fertility, soil conservation and tillage research, soil contamination and reclamation, soil biochemistry, and soil chemistry and physics.

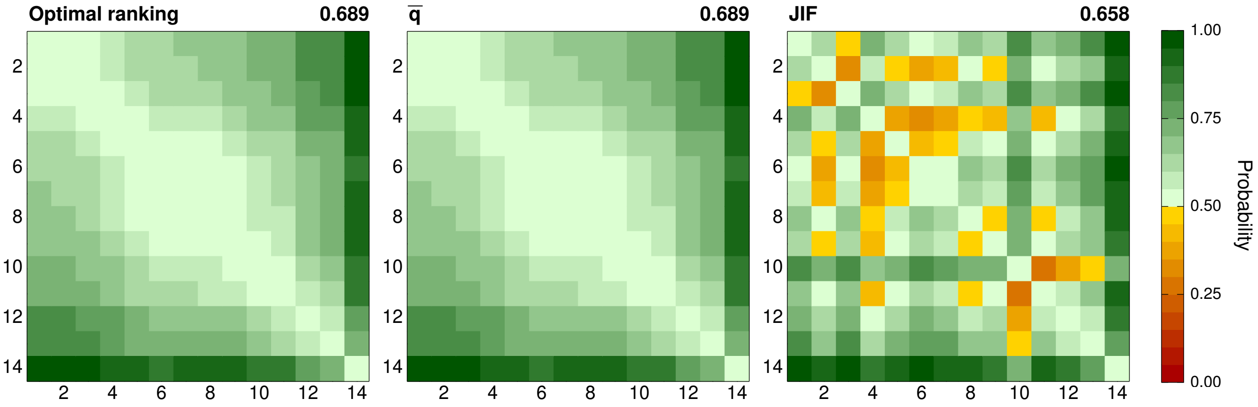

The color of each cell represents the probability of picking a paper with more citations from the higher-ranked journal. Green cells indicate adequate ranking, whereas red cells indicate that the ranking is inadequate (the probability is less than $\frac{1}{2}$). The value of M, the multi-class AUC statistic for each ranking scheme, is above each matrix.

Rank			$p_{ss}(q\|J)$		n			Steady-state
AUC	JIF	Journal abbreviation	$\overline{q}$	σ	$\overline{n}$	Q2	JIF	period
1	3	SOIL SCI SOC AM J	1.30	0.39	28.5	17	1.869	1971–1994
2	1	SOIL BIOL BIOCHEM	1.29	0.38	26.7	16	2.623	1987–1996
3	6	CLAY CLAY MINER	1.22	0.32	20.1	15	1.423	1990–1994
4	7	BIOL FERT SOILS	1.18	0.37	18.7	12	1.405	1984–1995
5	5	PLANT SOIL	1.12	0.40	17.1	11	1.495	1988–1991
6	9	SOIL SCI	1.08	0.42	18.0	10	0.956	1965–1997
7	2	GEODERMA	1.05	0.39	15.3	9	2.124	1967–2000
8	11	CAN J SOIL SCI	1.04	0.38	14.9	9	0.761	1964–1997
9	8	AUST J SOIL RES	1.00	0.33	12.1	8	1.072	1992–1999
10	4	SOIL TILL RES	0.95	0.36	11.0	7	1.619	1979–2001
11	12	SOIL SCI PLANT NUTR	0.88	0.37	9.7	6	0.443	1978–1994
12	13	COMMUN SOIL SCI PLAN	0.72	0.41	8.9	4	0.302	1973–1995
13	10	J SOIL WATER CONSERV	0.65	0.49	7.0	3	0.949	1966–2002
14	14	AGROCHIMICA	0.13	0.43	1.5	0	0.231	1965–2003

SPECTROSCOPY

ISI Category Description Spectroscopy covers resources concerned with the production, measurement, and interpretation of electromagnetic spectra arising from either emission or absorption of radiant energy by various sources. This category includes resources that report on any of several techniques for analyzing the spectra of beams of particles or for determining mass spectra.

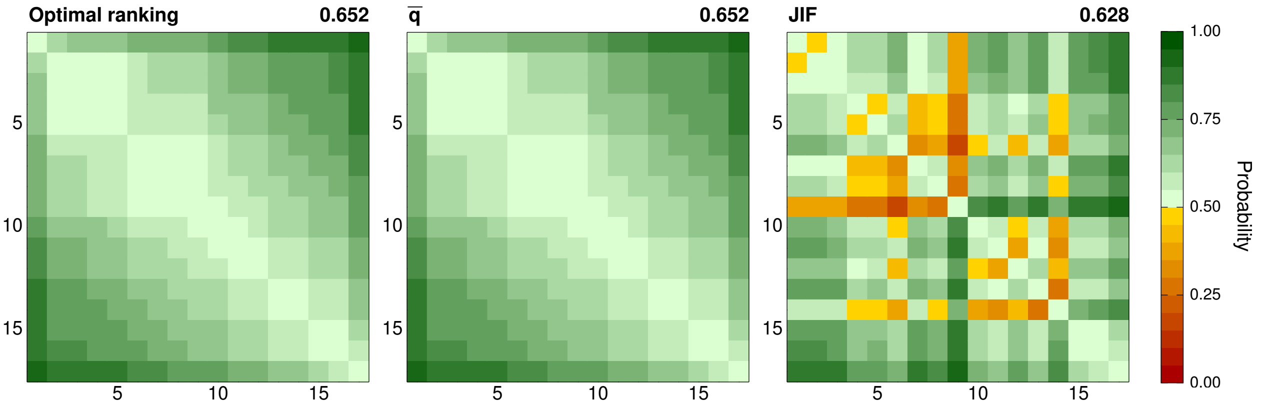

The color of each cell represents the probability of picking a paper with more citations from the higher-ranked journal. Green cells indicate adequate ranking, whereas red cells indicate that the ranking is inadequate (the probability is less than $\frac{1}{2}$). The value of M, the multi-class AUC statistic for each ranking scheme, is above each matrix.

**Rank**			$\mathbf{p_{ss}(q\|J)}$		**n**			**Steady-state**
**AUC**	**JIF**	**Journal abbreviation**	$\overline{\mathbf{q}}$	σ	$\overline{\mathbf{n}}$	**Q2**	**JIF**	**period**
1	9	J BIOMOL NMR	1.46	0.37	73.5	25	1.791	1991–1995
2	2	J AM SOC MASS SPECTR	1.26	0.37	25.5	15	3.307	1989–2000
3	1	J ANAL ATOM SPECTROM	1.25	0.33	19.6	14	3.630	1998–2000
4	3	SPECTROCHIM ACTA B	1.23	0.38	22.1	14	3.092	1966–1996
5	7	J MAGN RESON	1.19	0.40	30.0	13	2.076	1970–1992
6	14	J MOL SPECTROSC	1.12	0.35	16.2	11	1.260	1987–1993
7	8	APPL SPECTROSC	1.08	0.38	16.2	10	1.879	1970–1998
8	5	INT J MASS SPECTROM	1.08	0.42	18.0	10	2.337	1974–1994
9	4	RAPID COMMUN MASS SP	1.05	0.44	18.4	9	2.680	1989–1997
10	12	J ANAL APPL PYROL	1.00	0.37	12.8	8	1.412	1979–1998
11	10	MAGN RESON CHEM	0.93	0.35	9.9	6	1.610	1984–1994
12	6	J RAMAN SPECTROSC	0.91	0.38	10.0	6	2.133	1983–1999
13	11	J QUANT SPECTROSC RA	0.82	0.37	8.8	5	1.599	1998–2000
14	13	SPECTROCHIM ACTA A	0.82	0.36	7.4	5	1.270	1995–2001
15	15	NUCL INSTRUM METH A	0.73	0.44	8.3	4	1.185	1989–1994
16	16	J ELECTRON SPECTROSC	0.69	0.41	6.2	4	1.172	1995–2002
17	17	SPECTROSC LETT	0.53	0.39	3.7	2	0.772	1985–1998

SPORT SCIENCES

ISI Category Description Sport Sciences covers resources on the applied physiology of human performance, physical conditioning for sports participation, optimal nutrition for sports performance, and the prevention and treatment of sports-related injuries and diseases. This category also includes resources on sport psychology and sociology.

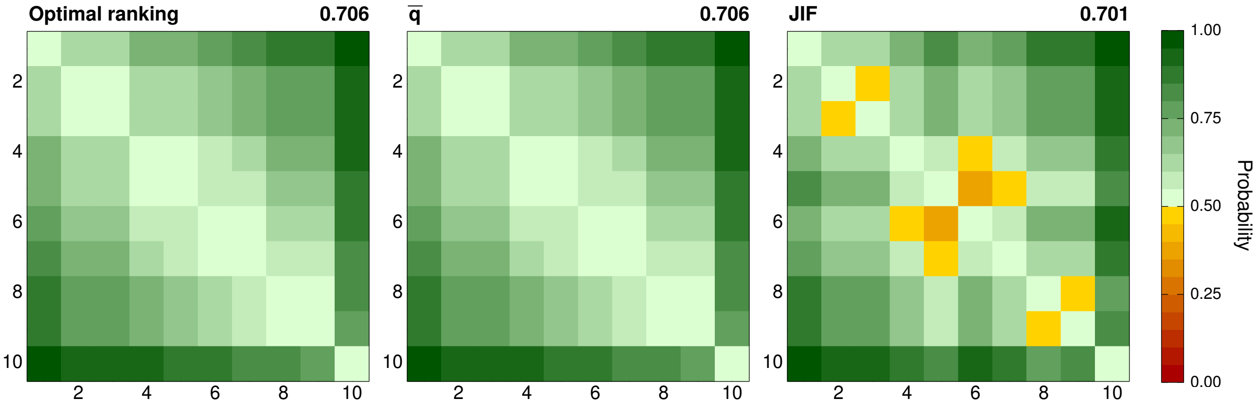

The color of each cell represents the probability of picking a paper with more citations from the higher-ranked journal. Green cells indicate adequate ranking, whereas red cells indicate that the ranking is inadequate (the probability is less than $\frac{1}{2}$). The value of M, the multi-class AUC statistic for each ranking scheme, is above each matrix.

Rank			$p_{ss}(q\|J)$		n			Steady-state
**AUC**	**JIF**	**Journal abbreviation**	$\overline{q}$	σ	$\overline{n}$	**Q2**	**JIF**	**period**
1	1	J APPL PHYSIOL	1.53	0.41	47.6	30	3.178	1975–1980
2	3	AM J SPORT MED	1.37	0.42	30.6	20	2.694	1981–1999
3	2	MED SCI SPORT EXER	1.35	0.40	31.3	19	2.909	1972–1995
4	6	INT J SPORTS MED	1.18	0.38	19.8	12	1.240	1981–1996
5	4	ARCH PHYS MED REHAB	1.13	0.44	18.5	11	1.826	1973–1999
6	7	RES Q EXERCISE SPORT	1.03	0.41	14.4	8	0.982	1979–1999
7	5	AM J PHYS MED REHAB	0.98	0.43	13.0	7	1.300	1972–1998
8	9	J SPORT MED PHYS FIT	0.86	0.38	8.3	6	0.568	1990–1999
9	8	AVIAT SPACE ENVIR MD	0.83	0.43	9.1	5	0.830	1974–1996
10	10	PHYSICIAN SPORTSMED	0.19	0.50	2.3	1	0.322	1988–2004

STATISTICS & PROBABILITY

ISI Category Description Statistics & Probability covers resources concerned with methods of obtaining, analyzing, summarizing, and interpreting numerical or quantitative data. Resources on the study of the mathematical structures and constructions used to analyze the probability of a given set of events from a family of outcomes are also covered.

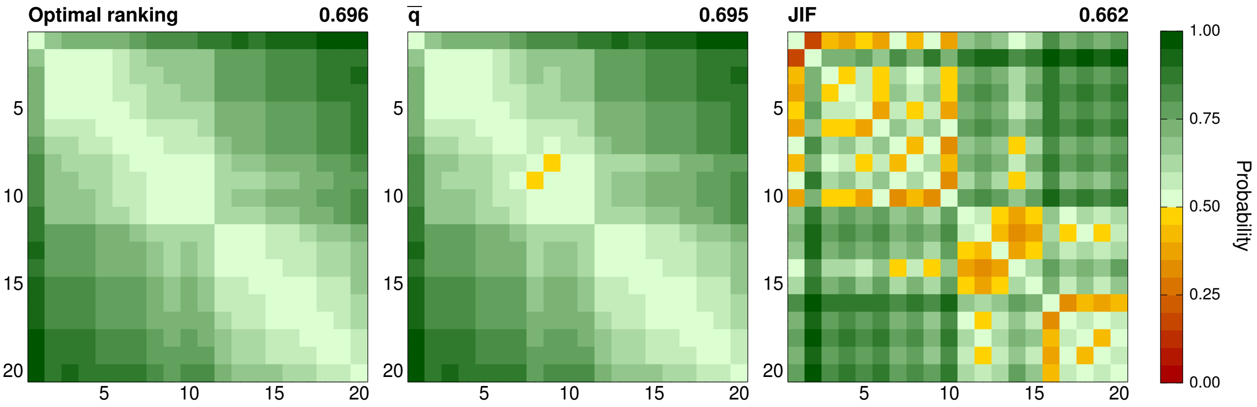

The color of each cell represents the probability of picking a paper with more citations from the higher-ranked journal. Green cells indicate adequate ranking, whereas red cells indicate that the ranking is inadequate (the probability is less than $\frac{1}{2}$). The value of M, the multi-class AUC statistic for each ranking scheme, is above each matrix.

Rank			$p_{ss}(q\|J)$		n			Steady-state
AUC	JIF	Journal abbreviation	$\bar{q}$	σ	$\bar{n}$	Q2	JIF	period
1	2	ECONOMETRICA	1.58	0.45	88.6	35	2.402	1980–1994
2	6	BIOMETRICS	1.25	0.46	48.1	14	1.489	1954–1994
3	10	BIOMETRIKA	1.22	0.42	36.8	13	1.014	1955–1996
4	4	ANN STAT	1.21	0.44	30.1	14	1.902	1972–1994
5	3	J AM STAT ASSOC	1.16	0.48	37.9	12	2.171	1955–1998
6	8	FUZZY SET SYST	1.11	0.45	23.4	11	1.181	1980–1987
7	5	STAT MED	1.04	0.40	16.9	9	1.737	1994–1997
8	1	CHEMOMETR INTELL LAB	0.96	0.42	14.8	8	2.450	1986–2002
9	14	ADV APPL PROBAB	0.98	0.51	13.8	7	0.789	1975–1992
10	7	ANN PROBAB	0.95	0.38	10.8	7	1.301	1987–1999
11	9	PROBAB THEORY REL	0.89	0.38	10.1	6	1.180	1985–1999
12	15	J MULTIVARIATE ANAL	0.73	0.40	7.3	4	0.763	1975–2000
13	13	STOCH PROC APPL	0.66	0.41	5.8	3	0.802	1983–1999
14	11	AM STAT	0.66	0.47	11.0	3	0.976	1964–2001
15	17	J APPL PROBAB	0.57	0.40	4.5	2	0.504	1993–2001
16	19	ANN I STAT MATH	0.56	0.44	6.2	2	0.355	1956–1998
17	12	COMPUT STAT DATA AN	0.51	0.41	4.3	2	0.928	1982–2003
18	18	J STAT PLAN INFER	0.44	0.41	3.4	2	0.497	1992–2000
19	20	STAT PROBABIL LETT	0.40	0.42	2.7	1	0.286	1994–2000
20	16	BIOMETRICAL J	0.27	0.44	2.3	1	0.612	1989–2005

SUBSTANCE ABUSE

ISI Category Description Substance Abuse covers resources on the behavior, education, treatment, and research of alcohol, drug, and other substances of addiction.

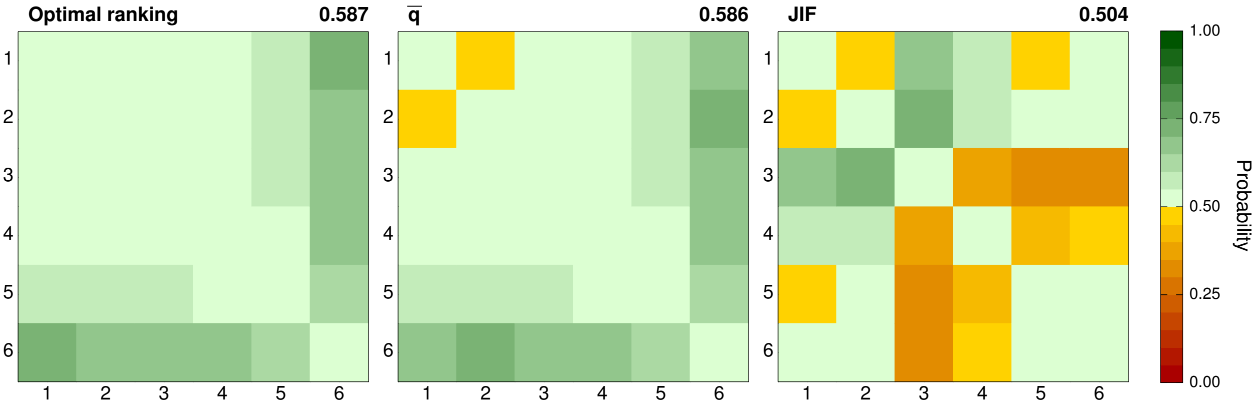

The color of each cell represents the probability of picking a paper with more citations from the higher-ranked journal. Green cells indicate adequate ranking, whereas red cells indicate that the ranking is inadequate (the probability is less than $\frac{1}{2}$). The value of M, the multi-class AUC statistic for each ranking scheme, is above each matrix.

**Rank**			$\mathbf{p_{ss}(q\|J)}$		**n**			**Steady-state**
**AUC**	**JIF**	**Journal abbreviation**	$\overline{\mathbf{q}}$	σ	$\overline{\mathbf{n}}$	**Q2**	**JIF**	**period**
1	2	ALCOHOL CLIN EXP RES	1.30	0.37	25.8	17	2.933	1981–1995
2	5	J STUD ALCOHOL	1.31	0.42	27.4	17	1.884	1974–1998
3	1	DRUG ALCOHOL DEPEN	1.27	0.36	21.6	16	3.213	1992–1999
4	6	ADDICT BEHAV	1.22	0.36	24.0	14	1.849	1974–1998
5	4	ALCOHOL	1.18	0.36	20.1	12	2.020	1987–1993
6	3	ALCOHOL ALCOHOLISM	0.98	0.42	12.3	8	2.061	1982–2000

SURGERY

ISI Category Description Surgery covers resources on general surgical topics including the different types of surgery (cardiovascular, neurosurgery, orthopedic, pediatric, or vascular); allied disciplines of surgery (surgical oncology, pathology, or radiology); and surgical techniques (arthroscopy, microscopy, or endoscopy).

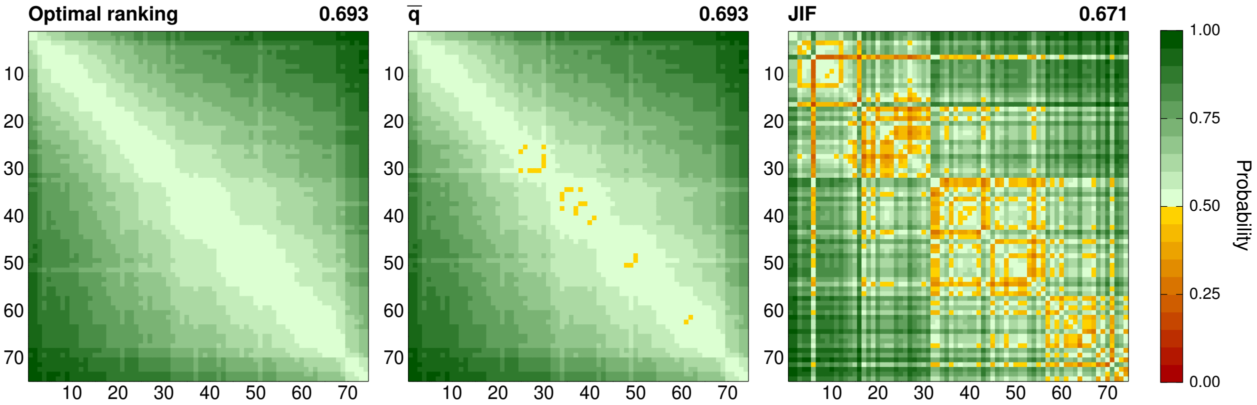

The color of each cell represents the probability of picking a paper with more citations from the higher-ranked journal. Green cells indicate adequate ranking, whereas red cells indicate that the ranking is inadequate (the probability is less than $\frac{1}{2}$). The value of M, the multi-class AUC statistic for each ranking scheme, is above each matrix.

Rank			$p_{ss}(q\|J)$		n			Steady-state
AUC	JIF	Journal abbreviation	$\overline{q}$	σ	$\overline{n}$	Q2	JIF	period
1	1	ANN SURG	1.62	0.39	56.4	37	7.678	1979–1999
2	2	AM J SURG PATHOL	1.59	0.36	49.0	34	4.144	1976–1995
3	16	J NEUROSURG	1.50	0.39	44.0	28	2.242	1974–1992
4	12	J BONE JOINT SURG AM	1.46	0.45	43.3	25	2.444	1965–1995
5	5	J NEUROL NEUROSUR PS	1.45	0.40	40.5	25	3.630	1954–1995
6	4	TRANSPLANTATION	1.42	0.38	35.2	22	3.972	1981–1995
7	7	J THORAC CARDIOV SUR	1.42	0.39	34.0	23	3.560	1976–1995
8	8	J VASC SURG	1.40	0.40	32.7	22	3.311	1983–1995
9	10	SURGERY	1.38	0.35	27.7	20	2.977	1993–1995
10	9	ARCH SURG-CHICAGO	1.37	0.40	31.5	20	3.058	1976–1997
11	27	J BONE JOINT SURG BR	1.36	0.39	30.6	19	1.790	1955–1995
12	11	NEUROSURGERY	1.33	0.42	27.8	18	2.692	1977–1997
13	3	BRIT J SURG	1.32	0.40	27.8	18	4.092	1979–1995
14	13	DIS COLON RECTUM	1.31	0.41	24.4	17	2.442	1989–1995
15	20	AM J SURG	1.28	0.42	25.4	16	2.101	1974–1996
16	24	J TRAUMA	1.27	0.41	25.6	16	2.035	1989–1993
17	28	WORLD J SURG	1.26	0.43	23.1	15	1.765	1983–1998
18	14	ANN THORAC SURG	1.21	0.42	22.1	14	2.342	1973–1995
19	31	ARTHROSCOPY	1.20	0.43	18.6	15	1.574	1991–1992
20	29	ARCH OTOLARYNGOL	1.18	0.40	19.6	13	1.734	1973–1995
21	26	PLAST RECONSTR SURG	1.17	0.46	22.5	12	1.864	1973–1993
22	18	CLIN ORTHOP RELAT R	1.16	0.38	17.6	12	2.161	1995–1998
23	25	HEAD NECK-J SCI SPEC	1.15	0.37	17.0	11	1.961	1988–1999
24	43	J PEDIATR SURG	1.13	0.36	15.7	11	1.109	1989–1995

table continues on next page ...

. . . table continued from previous page

Rank			$\mathbf{p_{ss}(q\|J)}$		n			Steady-state
**AUC**	**JIF**	**Journal abbreviation**	$\overline{\mathbf{q}}$	σ	$\overline{\mathbf{n}}$	**Q2**	**JIF**	**period**
25	15	J CATARACT REFR SURG	1.10	0.39	15.6	10	2.285	1985–2000
26	54	CLEFT PALATE-CRAN J	1.03	0.37	16.3	9	0.724	1990–1992
27	35	J HAND SURG-AM	1.10	0.38	13.8	11	1.286	1993–1995
28	30	SURG CLIN N AM	1.07	0.41	16.1	10	1.656	1971–1999
29	21	LASER SURG MED	1.07	0.36	14.1	9	2.077	1992–2000
30	23	J SURG RES	1.05	0.37	14.7	9	2.038	1978–1998
31	22	CLIN TRANSPLANT	1.03	0.32	11.7	9	2.051	1997–2000
32	37	ORAL SURG ORAL MED O	1.01	0.41	13.5	8	1.221	1970–1995
33	44	CLIN PLAST SURG	0.99	0.39	12.3	8	1.080	1978–1998
34	40	BRIT J PLAST SURG	0.97	0.41	13.0	8	1.182	1973–1997
35	34	AM SURGEON	0.97	0.38	10.8	7	1.299	1983–1998
36	19	EUR J CARDIO-THORAC	0.96	0.38	11.0	7	2.106	1989–2000
37	39	ACTA NEUROCHIR	0.98	0.48	11.8	8	1.212	1970–1995
38	56	J CARDIAC SURG	0.95	0.39	11.6	7	0.709	1990–1996
39	46	SURG NEUROL	0.97	0.41	10.0	7	1.057	1993–1998
40	53	HEPATO-GASTROENTEROL	0.94	0.37	10.3	7	0.756	1979–1997
41	41	J CRANIO MAXILL SURG	0.95	0.37	9.8	7	1.171	1986–2000
42	17	J SURG ONCOL	0.93	0.43	9.9	7	2.183	1988–1999
43	38	INT J ORAL MAX SURG	0.90	0.36	9.7	6	1.212	1985–1999
44	47	ANN PLAS SURG	0.89	0.39	9.9	6	1.027	1982–1995
45	49	MICROSURG	0.87	0.39	8.7	6	0.882	1989–1995
46	60	BRIT J ORAL MAX SURG	0.87	0.38	8.6	6	0.654	1983–1998
47	71	SCAND J PLAST RECONS	0.87	0.41	9.1	5	0.353	1980–1996
48	50	J HAND SURG-BRIT EUR	0.85	0.37	8.3	5	0.844	1983–1996
49	36	CHILD NERV SYST	0.86	0.38	7.6	6	1.257	1995–2000
50	55	ANN ROY COLL SURG	0.85	0.44	8.9	5	0.720	1973–1996
51	6	ENDOSCOPY	0.86	0.53	10.9	5	3.605	1995–2001
52	67	J RECONSTR MICROSURG	0.85	0.40	7.6	5	0.467	1989–1997
53	42	BURNS	0.82	0.37	7.5	5	1.139	1988–2001
54	58	EUR SURG RES	0.81	0.39	8.2	5	0.684	1968–2000
55	51	THORAC CARDIOV SURG	0.79	0.39	7.1	4	0.814	1978–2000
56	52	ARCH ORTHOP TRAUM SU	0.77	0.39	7.3	4	0.793	1978–1999
57	32	CLIN NEUROL NEUROSUR	0.77	0.38	6.6	4	1.506	1977–1998
58	33	OTOLARYNG HEAD NECK	0.75	0.40	6.9	4	1.338	1998–2002
59	65	CAN J SURG	0.74	0.42	7.0	4	0.515	1967–1999
60	66	CHIRURG	0.73	0.37	6.4	4	0.489	1990–1997
61	48	J CARDIOVASC SURG	0.72	0.41	6.7	4	1.020	1960–1997
62	45	INJURY	0.72	0.43	6.5	4	1.067	1973–1996
63	63	EUR J PEDIATR SURG	0.70	0.37	5.4	4	0.579	1990–2000
64	62	UNFALLCHIRURG	0.67	0.38	5.5	3	0.645	1988–1998
65	69	NEUROCHIRURGIE	0.66	0.39	5.2	3	0.386	1964–1999
66	74	INT SURG	0.66	0.39	5.1	3	0.273	1980–1999
67	59	AESTHET PLAST SURG	0.64	0.40	5.8	3	0.657	1975–2000

table continues on next page . . .

. . . table continued from previous page

**Rank**			$\mathbf{p_{ss}(q\|J)}$		$\mathbf{n}$			**Steady-state**
**AUC**	**JIF**	**Journal abbreviation**	$\overline{\mathbf{q}}$	σ	$\overline{\mathbf{n}}$	**Q2**	**JIF**	**period**
68	61	PEDIATR SURG INT	0.62	0.34	4.0	3	0.653	1999–2001
69	57	SURG TODAY	0.58	0.36	4.5	3	0.698	1991–1992
70	64	ZBL CHIR	0.48	0.39	3.1	2	0.548	1995–1997
71	68	ANN CHIR	0.45	0.40	3.1	2	0.442	1991–2000
72	73	REV CHIR ORTHOP	0.43	0.41	3.2	2	0.344	1971–2001
73	70	J CHIR-PARIS	0.35	0.39	2.2	1	0.365	1975–1999
74	72	ACTA CHIR BELG	0.35	0.52	2.3	1	0.348	1993–1999

TELECOMMUNICATIONS

ISI Category Description Telecommunications covers resources on the technical and engineering aspects of communications over long distances via telephone, television, cable, fiber optics, radio, computer networks, telegraph, satellites, and so on. Other relevant topics include electronics, opto-electronics, radar and sonar navigation, communications systems, microwaves, antennas, and wave propagation.

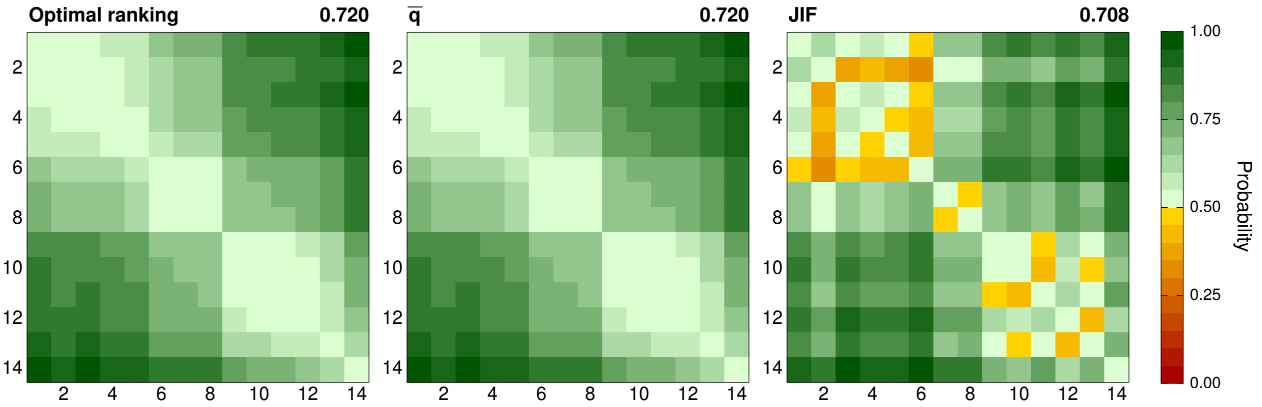

The color of each cell represents the probability of picking a paper with more citations from the higher-ranked journal. Green cells indicate adequate ranking, whereas red cells indicate that the ranking is inadequate (the probability is less than $\frac{1}{2}$). The value of M, the multi-class AUC statistic for each ranking scheme, is above each matrix.

Rank			$p_{ss}(q\|J)$		n			Steady-state
**AUC**	**JIF**	**Journal abbreviation**	$\overline{q}$	σ	$\overline{n}$	**Q2**	**JIF**	**period**
1	6	IEEE T VEH TECHNOL	1.01	0.42	23.4	8	1.071	1989–1993
2	1	IEEE J SEL AREA COMM	0.98	0.47	17.5	7	1.816	1988–2000
3	3	IEEE T ANTENN PROPAG	0.97	0.42	14.5	7	1.480	1969–1998
4	5	RADIO SCI	0.91	0.45	12.2	6	1.084	1965–1997
5	4	IEEE T COMMUN	0.86	0.48	14.0	5	1.208	1972–2001
6	2	IEEE COMMUN MAG	0.69	0.51	8.8	3	1.678	1996–2002
7	8	IEEE T ELECTROMAGN C	0.60	0.44	6.1	3	0.770	1966–2002
8	7	IEEE T AERO ELEC SYS	0.59	0.47	6.8	2	0.836	1971–1999
9	11	IEICE T COMMUN	0.25	0.44	2.3	1	0.290	1991–2000
10	9	IEEE T CONSUM ELECTR	0.18	0.45	1.9	1	0.727	1974–2002
11	13	ANN TELECOMMUN	0.15	0.47	1.6	0	0.168	1971–2001
12	10	COMPUT COMMUN	0.09	0.46	1.5	0	0.444	1990–2006
13	12	MICROWAVE J	-0.09	0.51	1.0	0	0.223	1976–1998
14	14	MICROWAVES RF	-0.57	0.46	0.2	0	0.045	1981–2005

THERMODYNAMICS

ISI Category Description Thermodynamics includes resources that focus on the areas of physics examining the transformations of matter and energy in physical and chemical processes, particularly those processes that involve the transfer of heat and changes in temperature. Relevant topics in this category include cooling and heating systems, cryogenics, refrigeration, combustion, energy conversion, and thermal stresses.

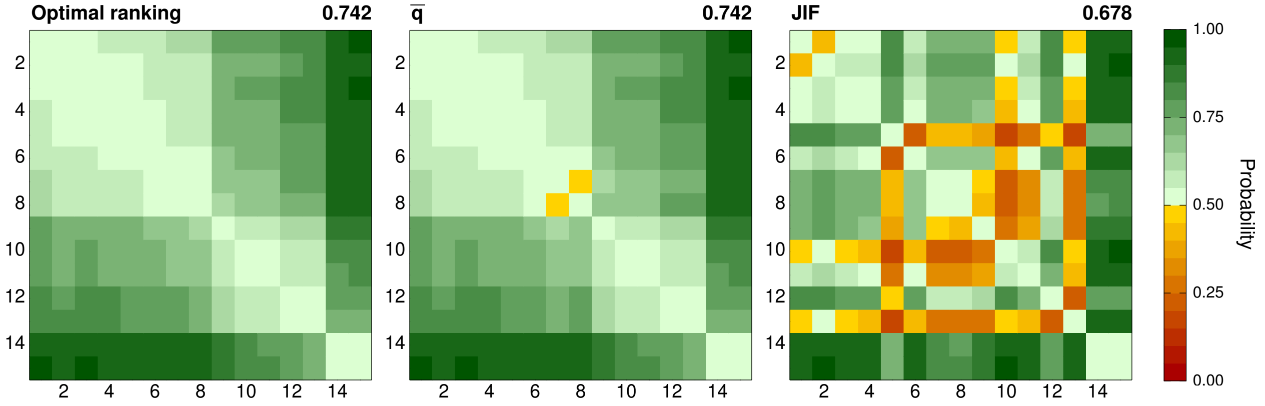

The color of each cell represents the probability of picking a paper with more citations from the higher-ranked journal. Green cells indicate adequate ranking, whereas red cells indicate that the ranking is inadequate (the probability is less than $\frac{1}{2}$). The value of M, the multi-class AUC statistic for each ranking scheme, is above each matrix.

Rank			$p_{ss}(q\|J)$		n			Steady-state
**AUC**	**JIF**	**Journal abbreviation**	$\overline{q}$	σ	$\overline{n}$	**Q2**	**JIF**	**period**
1	2	COMBUST FLAME	1.09	0.40	16.2	10	1.828	1957–1999
2	13	COMBUST SCI TECHNOL	1.04	0.42	15.0	9	0.651	1969–1992
3	10	J HEAT TRANS-T ASME	1.02	0.37	13.1	8	0.886	1978–1994
4	1	J CHEM THERMODYN	1.00	0.35	12.1	8	1.842	1983–1994
5	3	FLUID PHASE EQUILIBR	0.99	0.39	12.3	7	1.680	1987–1995
6	4	INT J HEAT MASS TRAN	0.93	0.34	10.4	7	1.482	1991–1997
7	6	NUMER HEAT TR A-APPL	0.88	0.34	8.8	6	0.936	1988–1992
8	11	INT J THERMOPHYS	0.88	0.39	9.2	6	0.793	1985–1997
9	9	EXP THERM FLUID SCI	0.65	0.36	5.9	3	0.894	1987–2000
10	7	ENERGY	0.58	0.43	4.7	2	0.935	1991–2001
11	8	CRYOGENICS	0.56	0.43	5.3	2	0.927	1977–1999
12	12	INT COMMUN HEAT MASS	0.46	0.38	3.2	2	0.708	1982–2002
13	5	ENERG CONVERS MANAGE	0.40	0.45	3.1	1	1.325	1987–2006
14	14	COMBUST EXPLO SHOCK+	-0.16	0.46	0.7	0	0.358	1972–2005
15	15	ASHRAE J	-0.21	0.46	0.6	0	0.297	1971–2004

TOXICOLOGY

ISI Category Description Toxicology covers resources that focus on the identification, biochemistry, and effects of harmful substances, including the side effects of drugs, in animals, humans, and the environment.

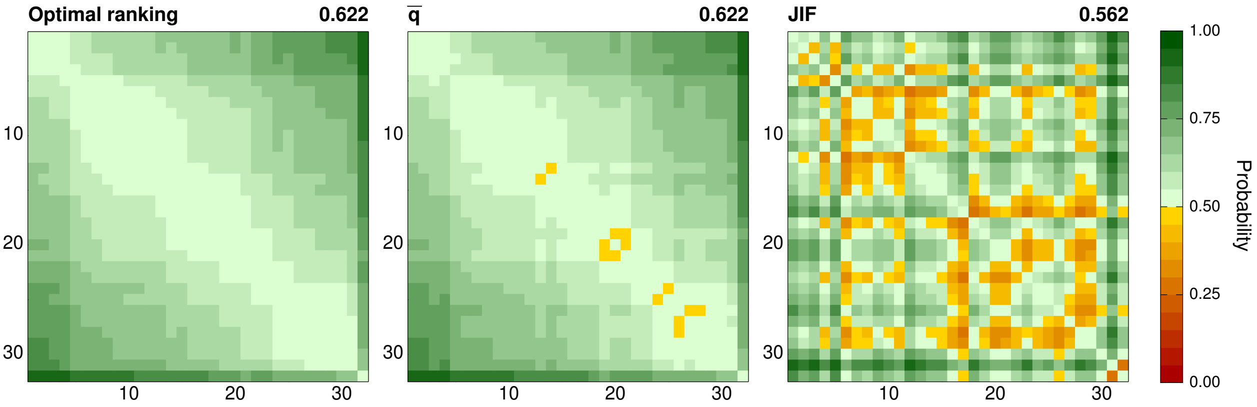

The color of each cell represents the probability of picking a paper with more citations from the higher-ranked journal. Green cells indicate adequate ranking, whereas red cells indicate that the ranking is inadequate (the probability is less than $\frac{1}{2}$). The value of M, the multi-class AUC statistic for each ranking scheme, is above each matrix.

Rank			$p_{ss}(q\|J)$		n			Steady-state
AUC	JIF	Journal abbreviation	$\overline{q}$	σ	$\overline{n}$	Q2	JIF	period
1	1	TOXICOL APPL PHARM	1.34	0.36	27.8	19	4.722	1979–1995
2	5	AQUAT TOXICOL	1.32	0.32	26.1	19	2.964	1982–1996
3	3	CHEM RES TOXICOL	1.29	0.30	23.4	16	3.162	1997–1999
4	12	ENVIRON TOXICOL CHEM	1.31	0.36	25.0	18	2.202	1983–1995
5	2	DRUGS	1.23	0.39	22.7	14	4.472	1991–2001
6	9	ENVIRON MOL MUTAGEN	1.20	0.38	21.3	14	2.653	1985–1996
7	13	NEUROTOXICOL TERATOL	1.19	0.35	20.8	12	2.143	1979–1997
8	18	ALCOHOL	1.18	0.36	20.1	12	2.020	1987–1993
9	28	ARCH ENVIRON CON TOX	1.17	0.36	17.5	12	1.419	1975–1995
10	23	CHEM-BIOL INTERACT	1.15	0.35	17.8	12	1.800	1985–1995
11	14	MUTAGENESIS	1.15	0.35	18.4	12	2.125	1985–1993
12	10	TOXICON	1.15	0.37	16.9	11	2.509	1966–1997
13	29	J ANAL TOXICOL	1.12	0.34	15.9	11	1.242	1977–1996
14	7	NEUROTOXICOLOGY	1.12	0.40	17.1	11	2.718	1980–1997
15	15	MAR ENVIRON RES	1.11	0.37	15.7	10	2.106	1977–1997
16	8	TOXICOLOGY	1.06	0.34	13.7	9	2.685	1994–2000
17	4	THER DRUG MONIT	1.06	0.36	14.0	9	3.032	1980–2000
18	19	ECOTOX ENVIRON SAFE	1.06	0.37	13.7	9	2.000	1976–1999
19	22	J TOXICOL ENV HEALTH	1.03	0.38	14.6	8	1.811	1994–1996
20	11	FOOD CHEM TOXICOL	1.04	0.37	14.3	9	2.393	1981–1999
21	24	ARCH TOXICOL	1.04	0.49	14.9	9	1.787	1973–1999
22	27	XENOBIOTICA	0.98	0.36	12.3	7	1.613	1990–2001
23	25	FOOD ADDIT CONTAM	0.97	0.37	11.4	8	1.780	1985–2001
24	6	TOXICOL LETT	0.92	0.38	10.6	7	2.784	1976–2001
25	16	TOXICOL PATHOL	0.92	0.45	11.4	6	2.092	1986–2000

table continues on next page . . .

. . . table continued from previous page

Rank			$\mathbf{p_{ss}(q\|J)}$		n			Steady-state
**AUC**	**JIF**	**Journal abbreviation**	$\overline{\mathbf{q}}$	σ	$\overline{\mathbf{n}}$	**Q2**	**JIF**	**period**
26	20	COMP BIOCHEM PHYS C	0.89	0.37	9.5	6	1.991	1985–2002
27	21	ANN OCCUP HYG	0.89	0.40	10.4	6	1.919	1977–2001
28	30	HUM EXP TOXICOL	0.90	0.40	8.6	6	1.122	1989–2000
29	32	B ENVIRON CONTAM TOX	0.87	0.36	8.4	6	0.505	1987–1991
30	26	J APPL TOXICOL	0.87	0.39	8.7	5	1.625	1983–2000
31	17	TOXICOL IN VITRO	0.81	0.39	7.9	5	2.045	1986–2002
32	31	VET HUM TOXICOL	0.53	0.37	3.7	2	0.660	1997–2000

TRANSPLANTATION

ISI Category Description Transplantation covers resources that focus on the assimilation of grafted tissue and the reconstitution of removed organs or parts of organs. The coverage focuses on transplantation procedures and the maintenance of transplanted tissues or organs. Specific transplantation coverage focuses on heart, lung, kidney, and bone marrow.

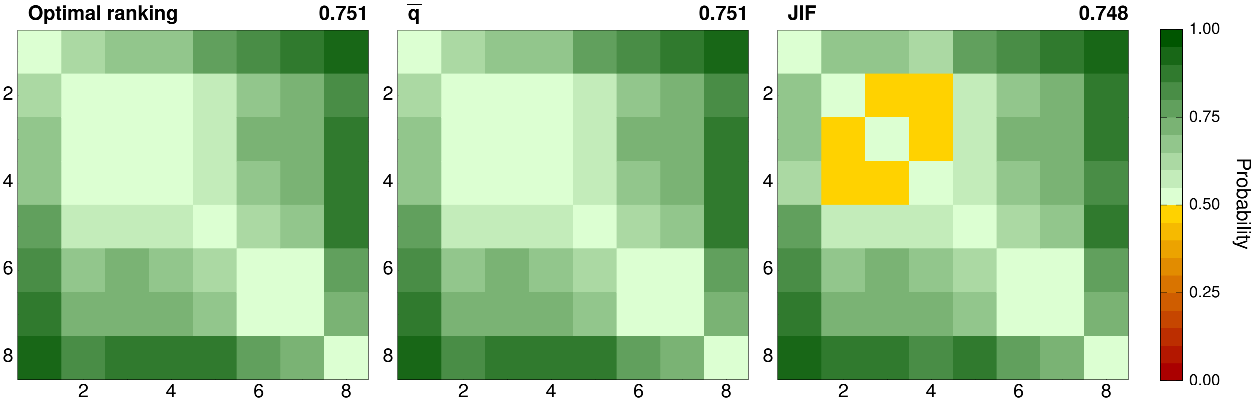

The color of each cell represents the probability of picking a paper with more citations from the higher-ranked journal. Green cells indicate adequate ranking, whereas red cells indicate that the ranking is inadequate (the probability is less than $\frac{1}{2}$). The value of M, the multi-class AUC statistic for each ranking scheme, is above each matrix.

Rank			$\mathbf{p_{ss}(q\|J)}$		**n**			**Steady-state**
**AUC**	**JIF**	**Journal abbreviation**	$\overline{\mathbf{q}}$	σ	$\overline{\mathbf{n}}$	**Q2**	**JIF**	**period**
1	1	TRANSPLANTATION	1.42	0.38	35.2	22	3.972	1981–1995
2	4	BONE MARROW TRANSPL	1.21	0.51	20.5	14	2.621	1993–1995
3	3	J HEART LUNG TRANSPL	1.15	0.37	18.6	12	2.830	1990–1998
4	2	NEPHROL DIAL TRANSPL	1.12	0.42	17.1	11	3.154	1987–1995
5	5	CLIN TRANSPLANT	1.03	0.32	11.7	9	2.051	1997–2000
6	6	ARTIF ORGANS	0.83	0.41	8.3	5	1.903	1978–1999
7	7	INT J ARTIF ORGANS	0.74	0.40	7.0	4	1.253	1977–2001
8	8	DIALYSIS TRANSPLANT	0.24	0.51	2.6	1	0.248	1977–2002

TRANSPORTATION SCIENCE & TECHNOLOGY

ISI Category Description Transportation Science & Technology covers resources on all aspects of the movement of goods and peoples as well as the design and maintenance of transportation systems. Topics covered in this category include logistics, vehicular design and technology, and transportation science and technology. Note: Resources that concentrate on transportation safety, policy, economics, and planning appear under the TRANSPORTATION category in the SSCI.

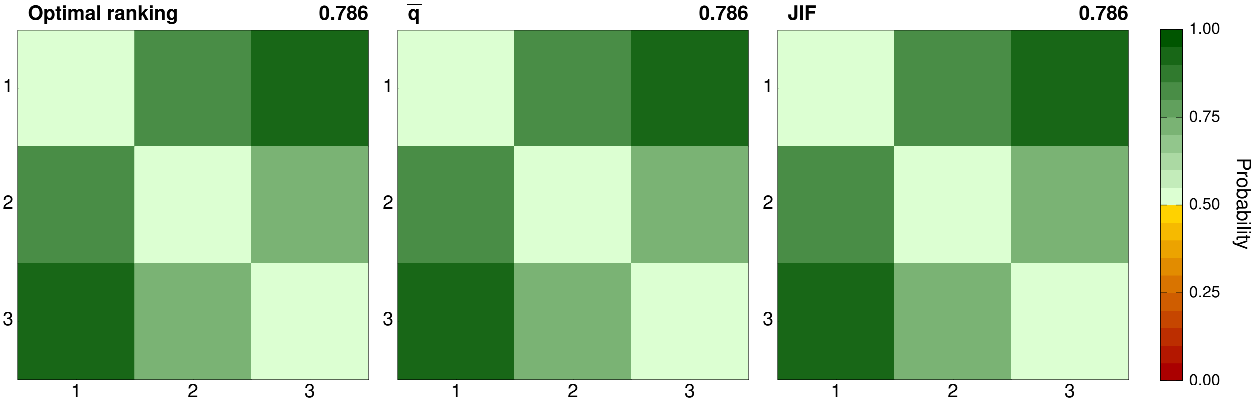

The color of each cell represents the probability of picking a paper with more citations from the higher-ranked journal. Green cells indicate adequate ranking, whereas red cells indicate that the ranking is inadequate (the probability is less than $\frac{1}{2}$). The value of M, the multi-class AUC statistic for each ranking scheme, is above each matrix.

**Rank**			$\mathbf{p_{ss}(q\|J)}$		**n**			**Steady-state**
**AUC**	**JIF**	**Journal abbreviation**	$\overline{\mathbf{q}}$	σ	$\overline{\mathbf{n}}$	**Q2**	**JIF**	**period**
1	1	IEEE T VEH TECHNOL	1.01	0.42	23.4	8	1.071	1989–1993
2	2	J TRANSP ENG-ASCE	0.31	0.42	2.3	1	0.410	1983–2002
3	3	ITE J	-0.33	0.47	0.4	0	0.132	1977–2004

TROPICAL MEDICINE

ISI Category Description Tropical Medicine covers resources on the study and treatment of disease, parasites, and other medical conditions unique to or originating in tropical regions.

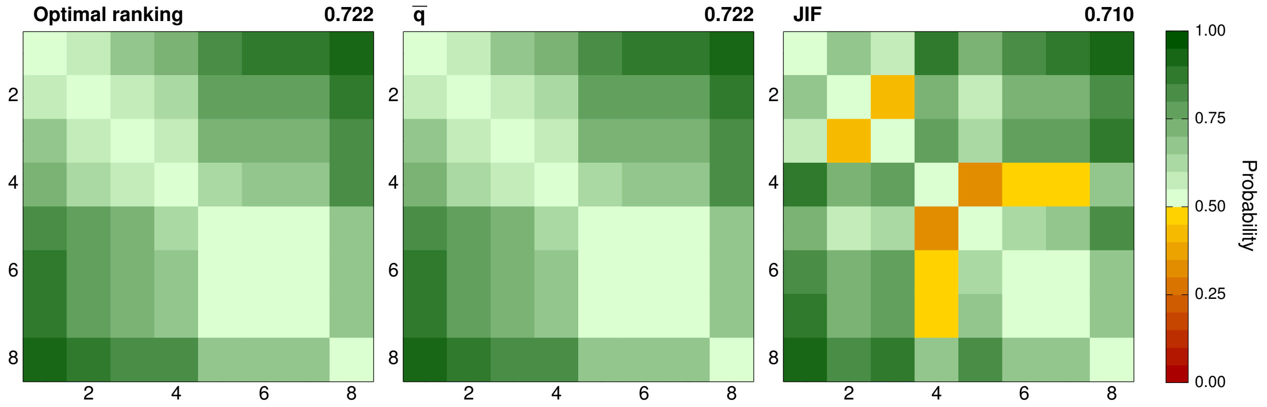

The color of each cell represents the probability of picking a paper with more citations from the higher-ranked journal. Green cells indicate adequate ranking, whereas red cells indicate that the ranking is inadequate (the probability is less than $\frac{1}{2}$). The value of M, the multi-class AUC statistic for each ranking scheme, is above each matrix.

**Rank**			$\mathbf{p_{ss}(q\|J)}$		**n**			**Steady-state**
**AUC**	**JIF**	**Journal abbreviation**	$\overline{\mathbf{q}}$	σ	$\overline{\mathbf{n}}$	**Q2**	**JIF**	**period**
1	1	AM J TROP MED HYG	1.25	0.37	22.2	15	2.546	1964–1996
2	3	T ROY SOC TROP MED H	1.10	0.40	17.0	10	2.030	1964–1998
3	2	ACTA TROP	1.02	0.39	12.8	9	2.211	1977–1997
4	5	ANN TROP MED PARASIT	0.89	0.38	9.1	6	1.191	1987–2000
5	6	ANN TROP PAEDIATR	0.67	0.37	5.2	3	0.934	1985–1998
6	7	J TROP PEDIATRICS	0.63	0.38	4.6	3	0.592	1979–2000
7	4	MEM I OSWALDO CRUZ	0.60	0.39	4.5	3	1.208	1971–2001
8	8	TROP DOCT	0.32	0.42	2.2	1	0.291	1977–2000

URBAN STUDIES

ISI Category Description Urban Studies covers resources concerned with the social aspects of city planning and urban design. Topics covered include the effects of the urban environment on the individual, the effects of urbanization on the natural environment, urban economics, urban technology, housing planning, urban education, and urban law.

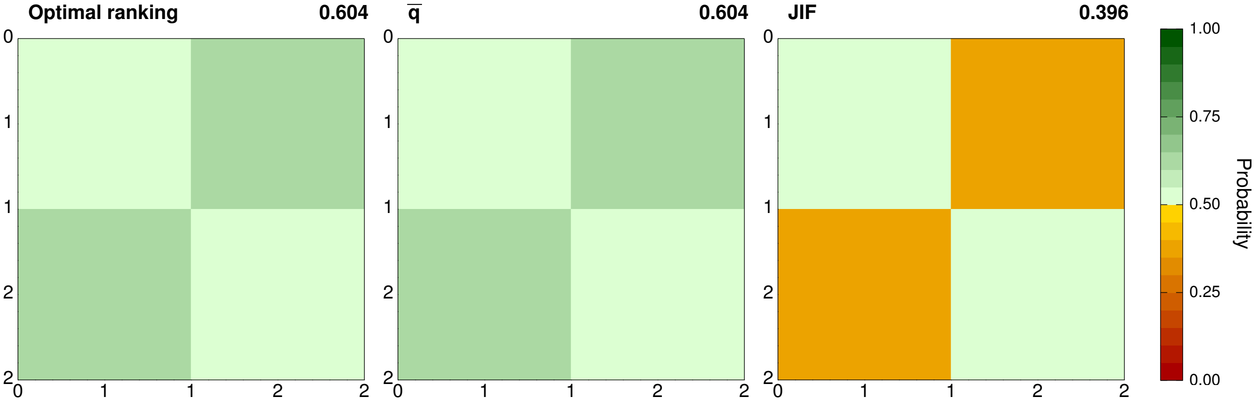

The color of each cell represents the probability of picking a paper with more citations from the higher-ranked journal. Green cells indicate adequate ranking, whereas red cells indicate that the ranking is inadequate (the probability is less than $\frac{1}{2}$). The value of M, the multi-class AUC statistic for each ranking scheme, is above each matrix.

Rank			$\mathbf{p_{ss}(q\|J)}$		n			Steady-state
**AUC**	**JIF**	**Journal abbreviation**	$\overline{\mathbf{q}}$	σ	$\overline{\mathbf{n}}$	**Q2**	**JIF**	**period**
1	2	URBAN STUD	0.83	0.39	8.7	5	0.992	1963–2002
2	1	LANDSCAPE URBAN PLAN	0.67	0.41	5.3	3	2.029	1985–2004

UROLOGY & NEPHROLOGY

ISI Category Description Urology & Nephrology covers resources on the diagnosis and treatment of diseases of the genitourinary tract and kidneys. This category includes general urology and nephrology as well as specialty resources on the prostate, dialysis and other blood purification techniques, transplantation, and renal failure.

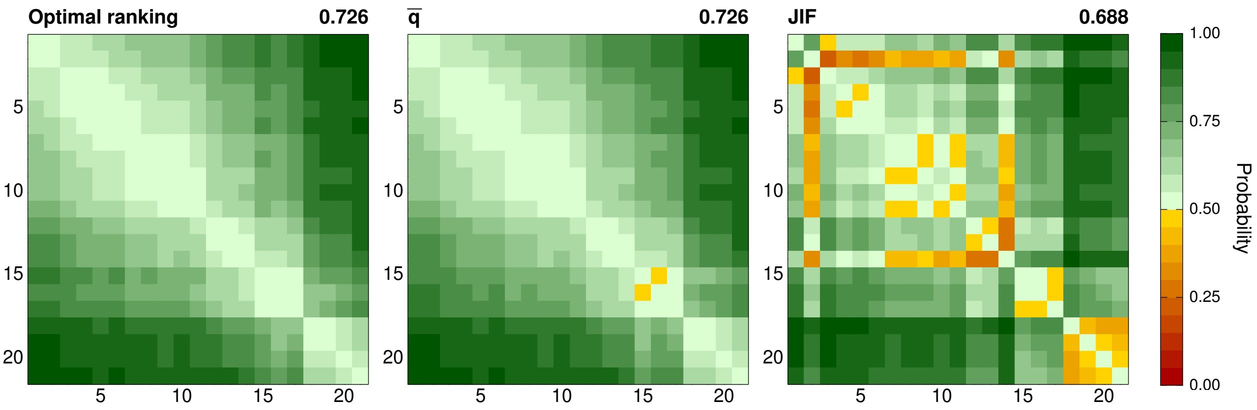

The color of each cell represents the probability of picking a paper with more citations from the higher-ranked journal. Green cells indicate adequate ranking, whereas red cells indicate that the ranking is inadequate (the probability is less than $\frac{1}{2}$). The value of M, the multi-class AUC statistic for each ranking scheme, is above each matrix.

Rank			$p_{ss}(q\|J)$		n			Steady-state
**AUC**	**JIF**	**Journal abbreviation**	$\overline{q}$	σ	$\overline{n}$	**Q2**	**JIF**	**period**
1	3	KIDNEY INT	1.42	0.44	40.2	23	4.773	1987–1991
2	1	J AM SOC NEPHROL	1.38	0.35	29.8	21	7.371	1989–2002
3	5	J UROLOGY	1.30	0.40	28.9	17	3.956	1989–1995
4	4	AM J KIDNEY DIS	1.28	0.41	27.3	16	4.072	1995–1998
5	6	PROSTATE	1.25	0.45	23.7	15	3.724	1986–2000
6	14	CLIN NEPHROL	1.22	0.37	20.6	14	1.418	1973–1994
7	9	UROLOGY	1.17	0.45	21.6	12	2.130	1995–1998
8	11	UROL CLIN N AM	1.14	0.39	18.2	12	1.819	1977–2001
9	7	NEPHROL DIAL TRANSPL	1.12	0.42	17.1	11	3.154	1987–1995
10	8	AM J NEPHROL	1.11	0.39	16.2	10	2.879	1981–1995
11	10	PEDIATR NEPHROL	1.07	0.39	14.3	10	2.007	1986–1995
12	2	EUR UROL	0.95	0.39	10.8	7	4.850	1989–2003
13	13	UROL RES	0.87	0.38	8.8	6	1.449	1972–2000
14	12	J ENDOUROL	0.85	0.40	9.0	5	1.536	1991–2000
15	17	RENAL FAILURE	0.71	0.38	5.7	4	0.699	1986–2000
16	15	SCAND J UROL NEPHROL	0.71	0.61	7.6	3	1.089	1971–1994
17	16	UROL INT	0.63	0.39	5.3	3	0.709	1963–1999
18	21	DIALYSIS TRANSPLANT	0.24	0.51	2.6	1	0.248	1977–2002
19	20	ANN UROL	0.22	0.39	1.6	1	0.338	1971–2000
20	19	AKTUEL UROL	0.11	0.47	1.5	0	0.400	1973–2000
21	18	NEFROLOGIA	-0.07	0.44	0.7	0	0.609	2002–2005

VETERINARY SCIENCES

ISI Category Description Veterinary Sciences covers resources concerning both the research and clinical aspects of animal health, diseases, injuries, nutrition, reproduction, and public health. This category includes materials on companion, farm, zoo, laboratory, wild, and aquatic animals.

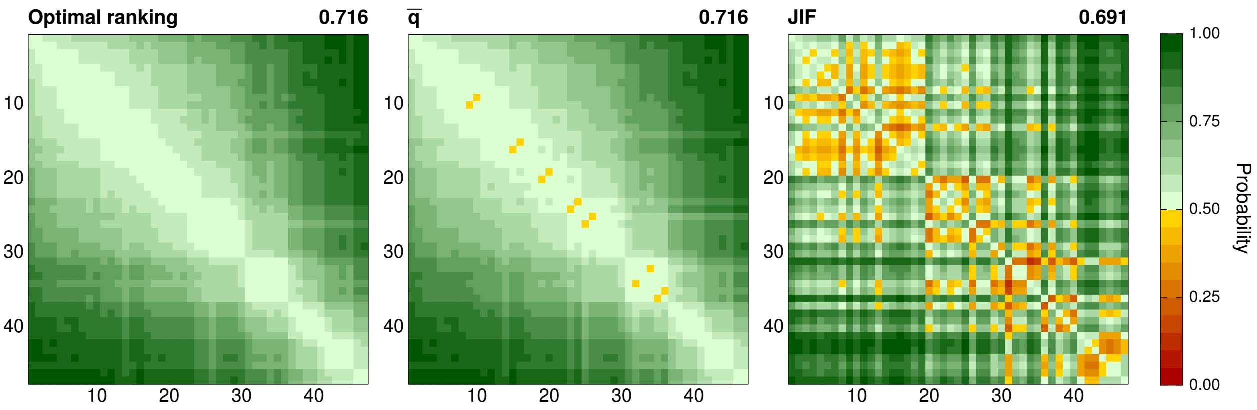

The color of each cell represents the probability of picking a paper with more citations from the higher-ranked journal. Green cells indicate adequate ranking, whereas red cells indicate that the ranking is inadequate (the probability is less than $\frac{1}{2}$). The value of M, the multi-class AUC statistic for each ranking scheme, is above each matrix.

Rank			$p_{ss}(q\|J)$		n			Steady-state
AUC	JIF	Journal abbreviation	$\overline{q}$	σ	$\overline{n}$	Q2	JIF	period
1	1	VACCINE	1.23	0.36	20.9	14	3.159	1987–1998
2	16	VET PATHOL	1.19	0.36	17.8	13	1.188	1970–1993
3	11	DIS AQUAT ORGAN	1.15	0.35	16.3	11	1.509	1984–1999
4	9	EQUINE VET J	1.14	0.35	15.9	11	1.730	1975–1998
5	17	APPL ANIM BEHAV SCI	1.11	0.38	15.3	11	1.177	1983–1994
6	2	VET MICROBIOL	1.09	0.35	14.3	10	2.073	1975–2000
7	15	AM J VET RES	1.09	0.33	14.0	10	1.241	1987–1996
8	4	VET IMMUNOL IMMUNOP	1.07	0.35	13.3	10	1.994	1979–1999
9	7	THERIOGENOLOGY	1.06	0.37	14.5	9	1.898	1994–2000
10	19	VET SURG	1.06	0.35	12.3	9	1.121	1981–1998
11	12	AVIAN DIS	1.04	0.38	13.9	9	1.369	1960–1998
12	14	RES VET SCI	1.03	0.35	12.7	8	1.258	1984–1995
13	3	MED VET ENTOMOL	1.02	0.37	12.3	8	2.033	1987–1998
14	18	VET REC	1.01	0.48	14.2	8	1.168	1970–1995
15	6	VET PARASITOL	0.97	0.35	11.4	7	1.900	1975–2001
16	25	J AM ANIM HOSP ASSOC	0.97	0.33	9.9	7	0.818	1991–1997
17	5	J MED ENTOMOL	0.96	0.39	11.5	7	1.950	1966–1998
18	28	J SMALL ANIM PRACT	0.95	0.41	10.4	7	0.717	1967–1998
19	22	J COMP PATHOL	0.91	0.37	9.4	6	0.939	1981–1999
20	27	VET CLIN N AM-SMALL	0.91	0.40	9.1	6	0.732	1979–2000
21	10	PREV VET MED	0.88	0.38	9.9	6	1.533	1983–2002
22	34	AUST VET J	0.89	0.37	9.0	6	0.571	1988–1995
23	8	AVIAN PATHOL	0.84	0.34	7.6	5	1.809	1994–2001
24	24	VET RADIOL ULTRASOUN	0.85	0.46	7.7	5	0.821	1991–1997

table continues on next page ...

... table continued from previous page

**Rank**			$\mathbf{p_{ss}(q\|J)}$		**n**			**Steady-state**
**AUC**	**JIF**	**Journal abbreviation**	$\overline{\mathbf{q}}$	σ	$\overline{\mathbf{n}}$	**Q2**	**JIF**	**period**
25	35	COMP CONT EDUC PRACT	0.81	0.42	7.1	5	0.550	1981–1997
26	37	ACTA VET SCAND	0.81	0.52	8.5	5	0.375	1964–1998
27	21	J WILDLIFE DIS	0.80	0.41	6.8	5	0.987	1998–2001
28	13	J VET MED B	0.78	0.38	6.9	4	1.356	1985–1999
29	23	J VET MED SCI	0.74	0.35	6.1	4	0.827	1990–1993
30	30	J VET MED A	0.71	0.39	5.5	4	0.627	1986–1998
31	40	J ZOO WILDLIFE MED	0.61	0.39	4.8	3	0.322	1984–1999
32	32	BERL MUNCH TIERARZTL	0.55	0.41	4.2	2	0.611	1975–2001
33	20	J ANIM PHYSIOL AN N	0.56	0.39	3.7	3	1.075	1995–2001
34	33	CAN VET J	0.57	0.51	5.0	2	0.606	1986–1997
35	29	VET HUM TOXICOL	0.53	0.37	3.7	2	0.660	1997–2000
36	26	SCHWEIZ ARCH TIERH	0.55	0.44	3.9	2	0.789	1976–2000
37	39	DEUT TIERARZTL WOCH	0.39	0.43	2.8	1	0.350	1979–2001
38	45	VET MED-US	0.34	0.43	2.8	1	0.145	1984–1994
39	46	KLEINTIERPRAXIS	0.27	0.42	1.9	1	0.080	1977–1999
40	44	REV MED VET-TOULOUSE	0.27	0.43	2.0	1	0.154	1975–1997
41	38	J EQUINE VET SCI	0.19	0.51	1.8	0	0.366	1993–2003
42	36	WIEN TIERARZTL MONAT	0.08	0.45	1.1	0	0.409	2000–2005
43	41	PRAKT TIERARZT	0.04	0.44	1.1	0	0.194	1976–2004
44	47	INDIAN VET J	0.04	0.38	0.9	0	0.036	1979–1997
45	31	VET MED-CZECH	0.02	0.52	1.6	0	0.624	2002–2006
46	43	MAGY ALLATORVOSOK	-0.16	0.42	0.5	0	0.155	1982–2005
47	42	TIJDSCHR DIERGENEESK	-0.19	0.41	0.5	0	0.188	1995–2005

VIROLOGY

ISI Category Description Virology includes resources dealing with all aspects of viral organisms and host-virus interactions. Resources in this category cover the molecular, biochemical, and cellular studies of plant-, animal-, and human-specific viruses, as well as bacteriophages. This category also contains materials on medical virology and pathogenesis and treatment of viral diseases.

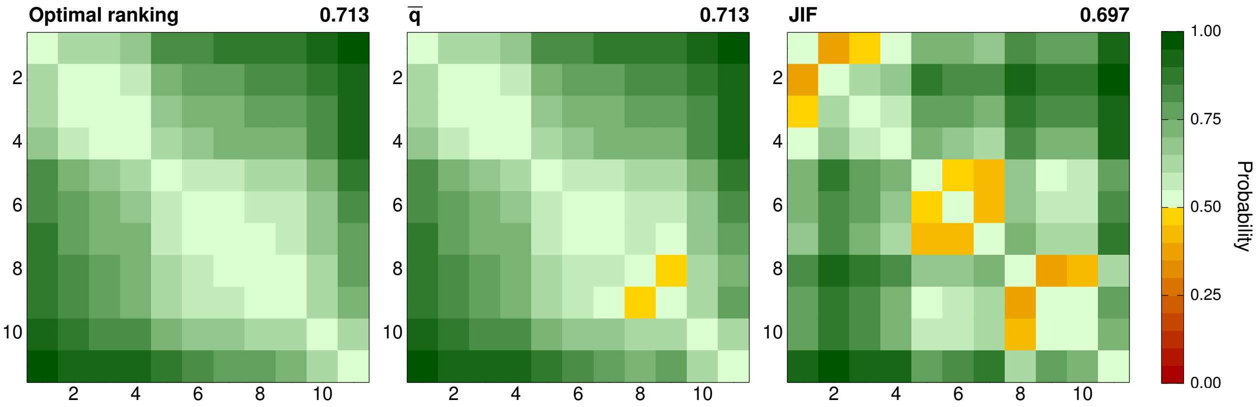

The color of each cell represents the probability of picking a paper with more citations from the higher-ranked journal. Green cells indicate adequate ranking, whereas red cells indicate that the ranking is inadequate (the probability is less than $\frac{1}{2}$). The value of M, the multi-class AUC statistic for each ranking scheme, is above each matrix.

Rank			$p_{ss}(q\|J)$		n			Steady-state
AUC	JIF	Journal abbreviation	$\overline{q}$	σ	$\overline{n}$	Q2	JIF	period
1	2	J VIROL	1.67	0.32	57.9	42	5.341	1991–1994
2	3	VIROLOGY	1.53	0.35	41.1	30	3.525	1986–1992
3	1	AIDS	1.49	0.38	37.7	27	5.632	1987–1999
4	4	J GEN VIROL	1.42	0.37	34.8	22	3.110	1985–1991
5	7	J MED VIROL	1.23	0.38	21.2	14	2.779	1976–1999
6	6	VIRUS RES	1.13	0.34	16.8	11	2.783	1989–1996
7	5	ANTIVIR RES	1.11	0.41	16.6	11	2.878	1980–1999
8	9	J VIROL METHODS	1.03	0.37	14.1	9	2.097	1979–2000
9	10	ARCH VIROL	1.03	0.47	14.0	9	1.850	1974–1997
10	8	AIDS RES HUM RETROV	0.81	0.41	9.2	4	2.513	1985–1986
11	11	ACTA VIROL	0.59	0.36	4.1	3	0.788	1984–2001

WATER RESOURCES

ISI Category Description Water Resources covers resources concerning a number of water-related topics. These include desalination, ground water monitoring and remediation, hydrology, irrigation and drainage science and technology, water quality, hydraulic engineering, ocean and coastal management, river research and management, waterways and ports.

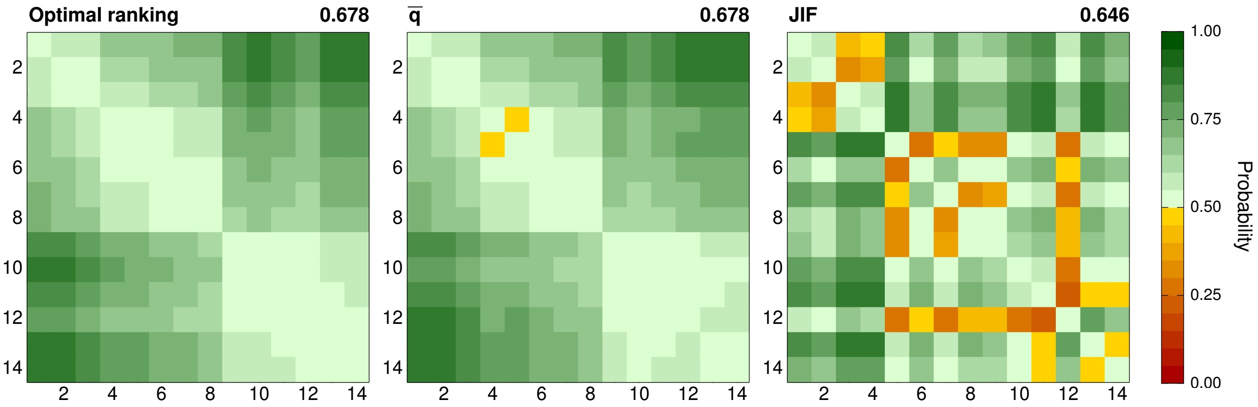

The color of each cell represents the probability of picking a paper with more citations from the higher-ranked journal. Green cells indicate adequate ranking, whereas red cells indicate that the ranking is inadequate (the probability is less than $\frac{1}{2}$). The value of M, the multi-class AUC statistic for each ranking scheme, is above each matrix.

Rank			$p_{ss}(q\|J)$		n			Steady-state
**AUC**	**JIF**	**Journal abbreviation**	$\overline{q}$	σ	$\overline{n}$	**Q2**	**JIF**	**period**
1	3	WATER RESOUR RES	1.34	0.44	33.2	19	1.894	1981–1992
2	4	CLAY CLAY MINER	1.22	0.32	20.1	15	1.423	1990–1994
3	1	WATER RES	1.18	0.37	20.5	13	2.459	1966–1998
4	2	J HYDROL	1.07	0.37	13.5	10	2.117	1991–2000
5	12	WATER ENVIRON RES	1.07	0.44	14.0	9	0.665	1991–1996
6	6	WATER AIR SOIL POLL	1.00	0.41	13.9	8	1.205	1974–1995
7	8	GROUND WATER	0.97	0.41	12.6	7	1.117	1984–1998
8	9	J HYDRAUL ENG-ASCE	0.94	0.45	12.2	7	1.004	1982–1995
9	7	AGR WATER MANAGE	0.71	0.40	6.0	4	1.122	1977–2002
10	5	J IRRIG DRAIN E-ASCE	0.63	0.39	5.0	3	1.250	1982–2002
11	10	J SOIL WATER CONSERV	0.65	0.49	7.0	3	0.949	1966–2002
12	14	J AM WATER WORKS ASS	0.68	0.65	9.8	3	0.431	1977–1994
13	13	ACTA HYDROCH HYDROB	0.55	0.43	4.5	2	0.632	1991–2001
14	11	DESALINATION	0.53	0.46	5.3	2	0.917	1975–1997

ZOOLOGY

ISI Category Description Zoology covers resources concerning a broad range of topics on the study of animals. This category ranges from animal behavior and animal physiology to some aspects of animal ecology. The category does not include veterinary medicine, ornithology, or most aspects of entomology.

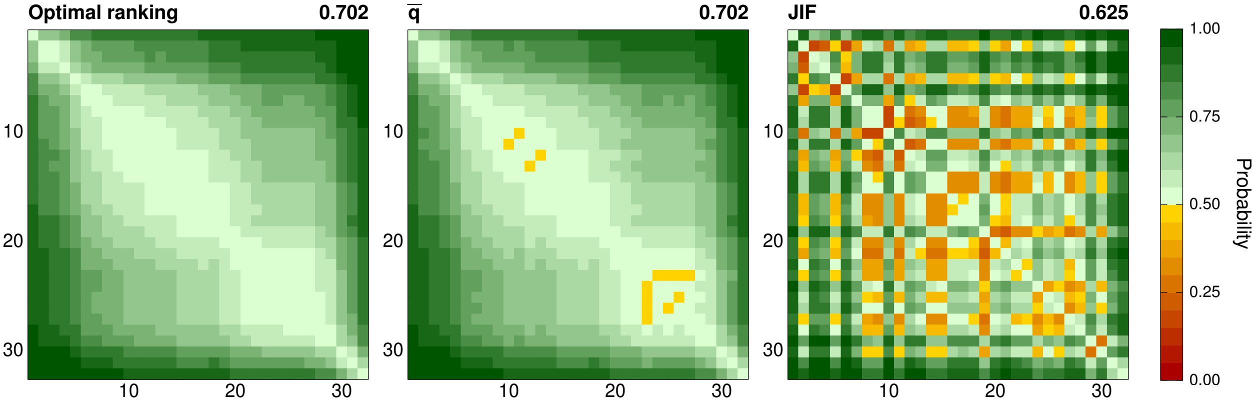

The color of each cell represents the probability of picking a paper with more citations from the higher-ranked journal. Green cells indicate adequate ranking, whereas red cells indicate that the ranking is inadequate (the probability is less than $\frac{1}{2}$). The value of M, the multi-class AUC statistic for each ranking scheme, is above each matrix.

Rank			$p_{ss}(q\|J)$		n			Steady-state
AUC	JIF	Journal abbreviation	$\overline{q}$	σ	$\overline{n}$	Q2	JIF	period
1	1	J COMP NEUROL	1.82	0.40	105.9	63	3.831	1969–1980
2	6	BEHAV ECOL SOCIOBIOL	1.60	0.31	44.4	36	2.316	1978–1990
3	3	J ANIM ECOL	1.57	0.34	47.5	33	3.390	1954–1996
4	4	ANIM BEHAV	1.51	0.33	38.6	28	2.711	1975–1993
5	10	J COMP PHYSIOL A	1.37	0.31	26.4	21	1.751	1984–1989
6	12	J MAMMAL	1.27	0.34	22.3	16	1.549	1965–1988
7	7	ETHOLOGY	1.25	0.34	19.7	15	2.245	1985–1993
8	21	BEHAVIOUR	1.21	0.33	18.4	13	1.165	1988–1998
9	13	J WILDLIFE MANAGE	1.19	0.34	18.3	13	1.538	1984–1995
10	17	J ZOOL	1.17	0.36	18.0	12	1.413	1986–1991
11	16	AM J PRIMATOL	1.18	0.43	17.4	13	1.429	1980–1998
12	20	J INVERTEBR PATHOL	1.12	0.35	15.7	11	1.235	1965–1995
13	27	COPEIA	1.12	0.39	16.8	11	0.840	1961–1994
14	18	CAN J ZOOL	1.11	0.37	15.7	10	1.393	1980–1996
15	23	HERPETOLOGICA	1.07	0.38	15.1	9	1.019	1977–1997
16	5	REPROD FERT DEVELOP	1.07	0.37	14.5	10	2.541	1988–1998
17	2	DEV COMP IMMUNOL	1.06	0.41	14.2	9	3.399	1976–2001
18	22	WILDLIFE RES	1.05	0.32	11.6	9	1.032	1990–1997
19	28	J HERPETOL	1.01	0.36	11.6	8	0.795	1976–1995
20	25	AUST J ZOOL	0.98	0.40	10.9	7	0.933	1975–1995
21	30	J NEMATOL	0.93	0.36	10.1	7	0.771	1968–1998
22	8	COMP BIOCHEM PHYS C	0.89	0.37	9.5	6	1.991	1985–2002
23	11	COMP BIOCHEM PHYS A	0.87	0.36	8.6	6	1.553	1986–2002
24	14	COMP BIOCHEM PHYS B	0.87	0.35	8.6	6	1.532	1987–2001

table continues on next page . . .

...table continued from previous page

Rank			$p_{ss}(q\|J)$		n			Steady-state
**AUC**	**JIF**	**Journal abbreviation**	$\overline{q}$	σ	$\overline{n}$	**Q2**	**JIF**	**period**
25	9	REPROD NUTR DEV	0.87	0.40	9.1	6	1.817	1979–2000
26	15	BEHAV PROCESS	0.87	0.39	9.0	5	1.478	1975–2000
27	26	WILDLIFE SOC B	0.87	0.40	8.7	6	0.843	1983–1998
28	24	J THERM BIOL	0.80	0.39	7.6	5	0.950	1976–2000
29	19	ZOOL SCI	0.76	0.40	6.9	4	1.240	1984–2000
30	29	ZOOL ANZ	0.55	0.40	4.2	2	0.775	1975–2003
31	31	REV SUISSE ZOOL	0.39	0.42	2.8	1	0.474	1976–2002
32	32	ZOOL ZH	0.11	0.42	1.2	0	0.219	1995–2003
